# A complete logical approach to resolve the evolution and dynamics of mitochondrial genome in bilaterians

**DOI:** 10.1371/journal.pone.0194334

**Published:** 2018-03-16

**Authors:** Laurent Oxusoff, Pascal Préa, Yvan Perez

**Affiliations:** 1 Laboratoire des Sciences de l’Information et des Systèmes UMR, Aix Marseille Université, Université de Toulon, CNRS, ENSAM, Marseille, France; 2 Laboratoire d’Informatique Fondamentale de Marseille UMR, Aix Marseille Université, CNRS, Ecole Centrale Marseille, Technopole de Château-Gombert, Marseille, France; 3 Institut Méditerranéen de Biodiversité et d’Ecologie marine et continentale UMR, Aix Marseille Université, Avignon Université, CNRS, IRD, Marseille, France; Tel Aviv University, ISRAEL

## Abstract

Investigating how recombination might modify gene order during the evolution has become a routine part of mitochondrial genome analysis. A new method of genomic maps analysis based on formal logic is described. The purpose of this method is to 1) use mitochondrial gene order of current taxa as datasets 2) calculate rearrangements between all mitochondrial gene orders and 3) reconstruct phylogenetic relationships according to these calculated rearrangements within a tree under the assumption of maximum parsimony. Unlike existing methods mainly based on the probabilistic approach, the main strength of this new approach is that it calculates all the exact tree solutions with completeness and provides logical consequences as highly robust results. Moreover, this method infers all possible hypothetical ancestors and reconstructs character states for all internal nodes of the trees. We started by testing our method using the deuterostomes as a study case. Then, with sponges as an outgroup, we investigated the evolutionary history of mitochondrial genomes of 47 bilaterian phyla and emphasised the peculiar case of chaetognaths. This pilot work showed that the use of formal logic in a hypothetico-deductive background such as phylogeny (where experimental testing of hypotheses is impossible) is very promising to explore mitochondrial gene order in deuterostomes and should be applied to many other bilaterian clades.

## Introduction

Unlike nuclear genome, mitochondrial genome (mtDNA) is rather small and simply structured. In Metazoa, it consists of circular DNA about 16 kb in size that, as a result of ancient intracellular symbiosis, has only retained a few well-characterized genes coding for: 13 protein subunits (nad1-6, nad4L, cox1-3, cob and atp6/8), 2 ribosomal RNAs (rRNAs) (rrnL, rrnS) and a maximum of 22 transfer RNAs (tRNAs) [1]. Recently, the emergence of next-generation sequencing techniques has significantly increased the amount of mtDNAs available in public databases. The comparative analysis of this growing amount of data has helped to broaden our understanding of the metazoan mtDNA evolution. Because it is assumed that nuclear genomes underwent similar evolutionary processes, it has been proposed that comparative analysis of mtDNAs could shed a new light on the mechanisms and selective forces driving whole-genome evolution in genomic data that are more tractable [2].

Besides the primary sequence information which has been proven valuable for evolutionary studies [1, 3–5], the mitochondrial (mt) gene order is also a reliable marker for phylogenetic inferences at many taxonomic levels for several reasons [4, 6–8]. First, the gene content is almost invariant and provides a unique and universal dataset. Second, stable structural gene rearrangements are assumed to be rare because functional genomes must be maintained, which limits the level of homoplasy [8]. Several studies successfully used mt gene orders to support phylogenetic hypotheses, for instance in crustaceans and insects [9–12], echinoderms [13] and annelids [14, 15]. However, relying on gene order to make phylogenetic inferences has, at times, been disappointing because no evolutionary significant changes could be identified in some lineages [16]. Indeed, mtDNA can strongly differ in tunicates, molluscs, brachiopods, platyhelminthes, bryozoans and nematodes and these high evolutionary rates lead to homoplasious gene orders (for a review see [6]). It is noteworthy that these problematic phyla appear as long-branched leaves in sequence-based phylogenetic analyses [16, 17], confirming that their rates of molecular evolution are unusually high. Nearly 80% of the rearrangements affect only tRNA genes. In the majority of these cases, only a single tRNA is affected [18]. The study of rearrangements of tRNA genes in the Hymenoptera suggests that the position of mt tRNA genes is selectively neutral [19], meaning that changes in their position must be considered as non-adaptive and therefore not informative to infer the evolutionary potential of species.

Changes in mt gene order can be assigned to three main models of intrachromosomal recombination:

- Change in the position of a genome segment containing one or several genes by transposition, inversion, reverse transposition and gain/loss [20],
- Tandem Duplication followed by Random Loss of genes (TDRL) [3],
- A variant of the latter which consists of tandem duplication followed by non-random loss [21].

In the first model, changes in mt gene number involve lineage-specific gains and losses, the losses being sometimes associated with mitochondria-to-nucleus gene relocation. Contrary to other rearrangement events, mt gene loss is rare, and gain is negligible in metazoans. Gene number variations have been reported more frequently in nonbilaterian compared with bilaterian animals (for a review of mt gene gain/loss see [22]). Losses of protein-coding mt genes have been reported, including losses of *atp*8 in placozoans, some sponges, most nematodes, some molluscs and platyhelminthes and losses of *atp*6 and *atp*8 in chaetognaths and ctenophores (in ctenophores atp6 has been transferred to the nucleus). The gain of novel protein-coding mt genes, including *atp*9, *tat*C, *mut*S and *Pol*B, has been reported in some sponges (*atp*9 has been transferred to the nucleus in some demosponges), placozoans, and cnidarians. It has been also suggested that *atp*9 and *tat*C were likely inherited vertically in sponges from choanoflagellates and lost in other animals while *mut*S and *pol*B were novelties acquired by horizontal gene transfer in some cnidarians from prokaryotes. Besides few variations in protein-coding gene content, many animal mtDNAs repeatedly lost tRNA genes, sometimes all but one or two, for instance in sponges, cnidarians, ctenophores and chaetognaths.

Intrachromosomal recombination often involves the replication origins [23], but other hot spots of rearrangements have been proposed [24]. TDRLs mostly occur across vertebrate lineages [25] and can easily describe local transposition. However, inversion and long-range transposition which are common in invertebrate mtDNAs [26] are more consistent with transposition, inversion, and reverse transposition model [20, 25]. In protostomes, TDRLs represent about 10% of the rearrangements [27]. Similar frequencies have been observed in the reconstructed rearrangements of metazoan mtDNAs and may suggest that TDRL plays a marginal role [25]. Mao et al. [25] proposed a model of recombination based on the coexistence of minicircular mtDNAs containing an origin of replication. This model accommodates the coexistence of nontandem repeat fragments and two or three copies of the control region. Consequentially, it is reasonable to consider only transposition, inversion, reverse transposition and gain/loss as elementary rearrangements to the evolution of mtDNAs in Metazoa, even though some transpositions may mechanistically result from TDRLs.

In the course of evolution, rearrangements are rare so that evolutionary scenarios minimizing their number are more likely to be close to reality. This allows the connection with combinatorial optimisation because the optimisation principle meets the parsimony criterion [28]. In general terms, this approach corresponds to the *genome rearrangement problem*: considering a set of genomes and potential rearrangements, search for the most parsimonious phylogenetic tree describing the rearrangement scenario(s) for multiple genomes [28]. One important aim of the *genome rearrangement problem* is to infer gene order in hypothetical ancestral species from extant species (the so-called *median problem*, see [28–30]). The situation we are faced with (hereinafter PHYLO problem) is to find the tree(s) *T* with a minimum number of rearrangements between all the mt gene orders of a given taxonomic dataset, and that verifies additional constraints imposing the existence of monophyletic groups. In this tree *T*, each node represents a mt gene order from extant organisms or from hypothetical ancestors, while each edge represents a rearrangement step between two linked nodes. Formally speaking, PHYLO corresponds to two known problems which were proven to be NP-hard [31]. If the phylogeny is fixed, PHYLO corresponds to the *small parsimony problem*; otherwise, it corresponds to the *large parsimony problem* [28, 31–33]. However, a simpler version of the *small parsimony problem* can be efficiently handled (see for instance [32] where the authors studied the two versions of the *genome rearrangement problem* under the Single-Cut-or-Join distance).

The inference of evolutionary relationships is one of the central problems of bioinformatics. Numerous software tools implementing methods for comparative analysis of gene order have been developed to infer phylogenies and genome evolution (for a review see [28, 32, 34, 35]). Breakpoint and reversal phylogenies (*e*.*g*., using the breakpoint or the reversal distance respectively) have been widely used (among others Blanchette et al. [36] and Sankoff and Blanchette [37] for the breakpoint phylogeny, and Moret et al. [38] and Bourque and Pevzner [29] for the reversal phylogeny) and studies using other variants of the *large parsimony problem* are scarce (for an exhaustive review see [28]). Another more realistic rearrangement model, with reversals, transpositions, translocations, fusions and fissions is modelled by the popular Double-Cut-and-Join operation [39]. The web-based program CREx considers transpositions, inversions, reverse transpositions, and TDRLs [27]. Formal logic provides an elegant way to represent and solve such a problem. It has the benefit of correctness, completeness and allows the understanding of the logical consequences (*i*.*e*., results that are true for all solutions found). First, PHYLO must be defined (axiomatisation) with a set of logic formulas or constraints. Second, a model generator calculates all the models, each model is a solution of PHYLO. Several complete model generators are available but a recurring difficulty is the computation time when the data set increases. When the search for a solution takes exponential time, two computing strategies are conceivable. First, an incomplete but fast algorithm that does not provide the optimal solution (for example, use local improvements from an initial random solution); or, second, a complete–and thus not efficient–algorithm on a smaller tractable dataset. While a large amount of genes found in the nuclear genome strongly limits our possibility to use formal logic with any conventional computer, we show in this paper that, for bilaterian mtDNAs, all the correct solutions can be found in a reasonable time due to the small number of genes.

Here, we present the first logical study of bilaterian mtDNAs for reconstruction of hypothetical ancestral gene orders that provides optimal solutions, including transposition, inversion, reverse transposition and gain/loss events. This new approach aims to reveal the evolutionary history from several mtDNAs and to infer their common plesiomorphic states (ground patterns). First, we used deuterostome mtDNAs as a study case. Second, we extended the analysis to the bilaterians and emphasised the peculiar case of chaetognaths.

## Methods

### Some definitions and properties

We will give in this section some formal definitions of usual biological notions and two useful properties (the Shared Block and Lower Bound Properties) used as heuristic tests.

For our purpose, a (unsigned) **gene** can be seen as an elementary item; a **signed gene** is a gene with (or without) the sign '-' before it. Given a signed gene *s*, we define -*s* by -*s =* -*g* if *s = g* (*g* is an unsigned gene) and -*s = g* if *s =* -*g*. A **genome** is a sequence of signed genes, represented by [*s*_0_ *s*_1_ … *s*_*n*-1_]. From a mathematical point of view, a genome comprising *n* genes *g*_0_, *g*_1_, …, *g*_*n*-1_ is represented by a **signed permutation** of {*g*_0_, *g*_1_, …, *g*_*n*-1_} (see [28] for a precise definition of a signed permutation). A genome can be **linear** or **circular**. In a circular genome, the last gene of the sequence is linked with the first one. Any genome with *n* genes, linear or circular, [*s*_0_ *s*_1_ … *s*_*n*-1_] also admits [-*s*_*n*-1_ -*s*_*n*-2_ … -*s*_1_ -*s*_0_] as a representation. For a linear genome, these are the only representations. A circular genome admits 2(*n*-1) other representations, the circular permutations of [*s*_0_ *s*_1_ … *s*_*n*-1_] and of [-*s*_*n*-1_ -*s*_*n*-2_ … -*s*_1_ -*s*_0_], *i*.*e*., [*s*_*i*_ *s*_*i+*1_ … *s*_*n*-1_ *s*_0_ … *s*_*i*-1_] for *i* in {1, …, *n*-1} and [-*s*_*i*_ -*s*_*i*-1_ … -*s*_0_ -*s*_*n*-1_ … -*s*_*i*+1_] for *i* in {1, …, *n*-1}.

In this study, we consider only circular genomes. By a little abuse of notation, we will say that all the representations of the same genome are equal and use the sign ' = '. For instance [1 2 -3] = [2 -3 1] = [-3 1 2] = [-1 3 -2] = [3 -2 -1] = [-2 -1 3].

By convention, a mtDNA is generally represented with *cox*1 as first signed gene. We call this representation **Canonical Linear Representation** (CLR). Every mtDNA has a unique CLR. For instance, the mtDNA for *Homo sapiens* (without the tRNA genes) is represented in CLR by [cox1 cox2 atp8 atp6 cox3 nad3 nad4L nad4 nad5 -nad6 cob rrnS rrnL nad1 nad2].

Given a genome [*s*_0_ *s*_1_ … *s*_*n*-1_] and *i*, *j* in {0, …, *n*-1}, we will say that the signed gene *s*_*j*_ is **at the right of** *s*_*i*_, that *s*_*i*_ is **at the left of** *s*_*j*_, or that *s*_*i*_*s*_*j*_ are **successive** if *j = i* + 1 (mod *n*). Notice that if *s*_*j*_ is at the right of *s*_*i*_, then -*s*_*i*_ is at the right of -*s*_*j*_.

Given a genome *G =* [*s*_0_ *s*_1_ … *s*_*n*-1_], a **block** (of genes) of *G* (or present in *G*) is a sequence [*s'*_0_ *s'*_1_ … *s'*_*k*_], with *0 ≤ k < n*-1, of signed genes, successive in *G*: for all *i* in {1, …, *k*}, *s'*_*i*_ is at the right of *s'*_*i*-1_. Notice that a block may contain only one signed gene. Conversely, a whole genome is not a block.

For example, if *G =* [1 2 3 4 5 6 7 8 9], then [3 4 5] and [7 8 9 1] are blocks of *G*, as [-5 -4 -3 -2] and [-1 -9 -8] (*G* is also represented by [-9 -8 … -2 -1]).

The notions **at the right**, **at the left** and **successive** naturally extend from genes to blocks.

If *B =* [*s*_*i*_ *s*_*i*+1_ … *s*_*j*_] is a block of *G*, we define the **inverted** block -*B* by [-*s*_*j*_ … -*s*_*i*+1_ -*s*_*i*_]. Notice that -*B* is also a block of *G* and that, as for genes, if a block *B*_1_ is at the right of a block *B*_2_, -*B*_2_ is at the right of -*B*_1_.

Let *G* and *G'* be two genomes having exactly the same genes and *s*_1_*s*_2_ be two successive (signed) genes in *G*. The position (in *G*) between *s*_1_ and *s*_2_ is a **breakpoint** of *G* and *G'* if *s*_1_*s*_2_ are not successive genes in *G'*. The number of breakpoints of *G* and *G'* is denoted by **nb_breakpoints(*G*, *G'*)**.

There exist five (elementary) rearrangements:

- **Inversion**: a block *B* of genes separates from the genome and is re-inserted, in the opposite direction at the same place. Equivalently, *B* is replaced by -*B*.
- **Transposition**: a block of genes separates from the genome and is re-inserted between two successive genes, at a different position.
- **Reverse transposition**: a block of genes separates from the genome and is re-inserted in the opposite direction between two successive genes, at a different position.
- **Loss**: a block *B* is removed from the genome.
- **Gain**: a block *B* is inserted in the genome.

In what follows, we will consider only the three first rearrangements (inversion, transposition and reverse transposition). This will be justified within the description of the algorithm Genome_Comparison.c.

Given two genomes *G*_0_ and *G*_*k*_, a **path *P*** between *G*_0_ and *G*_*k*_ is a sequence (*G*_0_, *G*_1_, …, *G*_*k*_) of *k*+1 genomes such that, for all *i* in {0, …, *k*-1}, it is possible to transform *G*_*i*_ into *G*_*i*+1_ with one rearrangement (transposition, inversion or reverse transposition). We will denote the path *P* by *G*_0_ → *G*_1_ → … → *G*_*k*_.

The **length** of a path *P* is the number of its rearrangements. A path of length *k* is called a ***k*-path** (a *k*-path is made of *k*+1 genomes). A path between two genomes *G* and *G'* is **minimal** if there exist no shorter paths between *G* and *G'*. The **distance** *d*(*G*,*G*’) between two genomes *G* and *G*’ is the length of a minimal path between them. This distance between two genomes is a metric since:

- *d*(*G*, *G'*) = 0 if and only if *G = G'*.
- *d*(*G*, *G'*) *= d*(*G'*, *G*) (if *G*_0_ → *G*_1_ → … → *G*_*k*_ is a path from *G*_0_ to *G*_*k*_, then *G*_*k*_ → *G*_*k*-1_ → … *→ G*_1_ → *G*_0_ is a path from *G*_*k*_ to *G*_0_).
- *d*(*G*_0_, *G*_2_) ≤ *d*(*G*_0_, *G*_1_) + *d*(*G*_1_, *G*_2_) (the concatenation of a path from *G*_0_ to *G*_1_ and of a path from *G*_1_ to *G*_2_ is a path from *G*_0_ to *G*_2_, not necessarily minimal).

From a biological point of view, a path is an evolutionary scenario.

Given a path *G*_0_ → *G*_1_ → … → *G*_*k*_, the rearrangement *G*_*i*_ → *G*_*i*+1_ is a **cut** if there exists a block present in *G*_0_, *G*_*i*_ and *G*_*k*_ but not in *G*_*i*+1_.

**Property 1.** *Let G*_0_ *and G*_*k*_ *be two genomes having exactly the same genes*. *Then there exist a minimal path P*_*min*_ *= G*_0_ → *G*_1_ → … → *G*_*k*_ *with no cuts*. *That is to say that*, *if a block of genes is present both in G*_0_ *and G*_*k*_ *(possibly inverted)*, *then it is present in G*_*i*_, *for all i in* {0, …, *k*}.

*Proof*. Let *P = G*_0_ → *G*_1_ → … → *G*_*k*_ be a *k*-path from *G*_0_ to *G*_*k*_. We will transform *P* into a path, not longer than *P*, which has no cut. Let us suppose that *P* has at least one cut and that the last cut occurs at *G*_*i*_ → *G*_*i*+1_; let *B* = [*B*_1_ *B*_2_] be the relevant block: *B*_1_ and *B*_2_ are successive in *G*_*i*_ and *G*_*k*_, but not in *G*_*i*+1_.

Let *G*_*k'*_ be the first genome occurring after *G*_*i*+1_ in which *B* is present (*B* is present in all *G*_*j*_ for *k' < j < k*). Notice that *B*_1_ and *B*_2_ are blocks of genes which are present in all *G*_*i'*_ for *i < i’ < k* (they are present in *G*_0_, *G*_*i*+1_ and *G*_*k*_, and the last cut occurs at *G*_*i*_ → *G*_*i*+1_). We construct a path *P' = G*_0_ → *G'*_1_ → *G'*_2_ → … → *G'*_*k*-1_ → *G*_*k*_ as follows:

- For 0 < *j* ≤ *i* and *k’ ≤ j < k*, *G'*_*j*_ = *G*_*j*_.
- For *i* < *j* < *k'*, we construct *G'*_*j*_ by replacing, in *G*_*j*_, *B*_2_ by *B* and *B*_1_ by the empty block. Considering the rearrangements *μ*_*j*_ at *G*_*j*_ → *G*_*j*+1_ for *j* ≥ *i*, that is to say, that rearrangements *μ’*_*j*_ at *G’*_*j*_ → *G’*_*j*+1_ is defined by:
  - If *μ*_*j*_ moves *B*_1_ (possibly with other blocks), then *μ*'_*j*_ moves only the other blocks (if there are no other blocks moved by *μ*_*j*_, *μ*_*j*_ moves the empty block; this is equivalent to removing a step in the path).
  - If *μ*_*j*_ moves *B*_2_ (possibly with other blocks), then *μ'*_*j*_ moves [*B*_1_ *B*_2_] (with the same blocks). Remark that if *μ*_*j*_ moves [*C*_1_ *B*_1_ *C*_2_ *B*_2_ *C*_3_], where C_1_, C_2_, C_3_ are other blocks of G_*j*_, then *μ'*_*j*_ moves [*C*_1_ *C*_2_ *B*_1_ *B*_2_ *C*_3_].
  - If *μ*_*j*_ moves a block *C* (not containing *B*_2_) to the left of *B*_2_, *μ'*_*j*_ moves *C* to the left of *B*_1_ (*B*_1_ is at the left of *B*_2_ in *G'*_*j*_ and *G'*_*j*+1_).
  - If *μ*_*j*_ moves a block *C* to the right of *B*_1_ and *B* is already cut (so *j* > *i* and *μ*_*j*_ does not move *C* to the left of *B*_2_ but to the left of another block *B*_3_), then *μ'*_*j*_ moves *C* to the left of *B*_3_.
  - If *μ*_*j*_ moves a block *C* to the left of *B*_1_, then *μ*_*j*_ moves *C* to the right of a block *B*_3_; in this case, *μ'*_*j*_ moves *C* to the right of *B*_3_.
  - The other rearrangements are unchanged.

Remark that, if we denote *B* = [*B*_1_ *B*_2_] by *B’*_2_ and the empty block by *B’*_1_, then the paths *P*_*i*,*k’*_ = *G*_*i*_ → *G*_*i*+1_ → … → *G*_*k’*_ and *P’*_*i*,*k’*_ = *G’*_*i*_ → *G’*_*i*+1_ → … → *G’*_*k’*_ are similar: for every *l* in {*i*, …, *k’*}, the only difference between *G’*_*l*_ and *G*_*l*_ is that *B*_2_ is replaced by *B’*_2_ and *B*_1_ by *B’*_1_. As [*B’*_1_ *B’*_2_] = [*B*_1_ *B*_2_], *G’*_*k’*_ = *G*_*k’*_, and thus *G’*_*k*_ = *G*_*k*_.

The path *P'* is shorter than or has the same length as *P*, transforms *G*_0_ into *G*_*k*_ and has fewer cuts than *P*. By repeating this construction, we transform *P* into a path with no cut.

QED

If *G*_0_ → *G*_1_ → … → *G*_*k*_ is a shortest path from *G*_0_ to *G*_*k*_, then for every *i*, *j* with 0 ≤ *i* ≤ *j*≤ *k*, *G*_*i*_ → *G*_*i*+1_ → … → *G*_*j*_ is a shortest path from *G*_*i*_ to *G*_*j*_.

It is thus possible to strengthen Property 1.

**Property 2 (Shared Block Property).** *Let G*_*0*_ *and G*_*k*_ *be two genomes having exactly the same genes*. *Then there exist a minimal path P*_*min*_ *= G*_*0*_ *→ G*_*1*_ *→* … *→ G*_*k*_ *such that*, *for every i*, *j with 0 ≤ i ≤ j ≤ k*, *if a block of genes is present in G*_*i*_ *and G*_*j*_, *then it is present in G*_*i'*_ *for all i' in {i*, …, *j}*.

Properties 1 and 2 say that, among all the minimal paths between two genomes, some are without cuts. We conjecture that *all* the minimal paths between two circular genomes are without cuts. This conjecture is false for linear genomes, as shown by the following example: [1 -2 -3 -5 -4] → [1 -2 5 3 -4] → [1 4 -3 -2 5] → [1 2 3 4 5] has a cut (between -5 and -4) but is a minimal path between [1 -2 -3 -5 -4] and [1 2 3 4 5] when considered as linear genomes. If we consider these two genomes as circular ones, then:

[1 -2 -3 -5 -4] → [1 -2 -3 4 5] = [-2 -3 4 5 1] → [-2 -1 -5 -4 -3] = [1 2 3 4 5] is a 2-path between [1 -2 -3 -5 -4] and [1 2 3 4 5]. For this example, there exist seven other minimal paths (of length 2), all without cuts.

**Property 3 (Lower Bound Property—**Bafna and Pevzner [40] and Fertin et al. [28]**)**. *If G and G’ are two genomes at distance d one from the other then 3 × d ≥ nb_breakpoints(G*, *G’)*.

This property links the distance *d*(*G*, *G’*) with *nb_breakpoints*(*G*, *G’*). Although this link is rather tight (it is a lower bound), it is useful because *nb_breakpoints(G*, *G’)* is easy to calculate.

### Sketch of the method

Solving the PHYLO problem with completeness consists of enumerating all the equiparsimonious trees that explain the paths between distinct mtDNAs with the minimum number of rearrangements. In order to calculate these trees, the reconstruction method is organised along four main procedures. First, a pairwise genome comparison program, which is called Genome_Comparison.c, calculates the distances between all mtDNAs. Second, a complete finite model generator for first-order logic calculates all the most parsimonious trees that respect the distance matrix and clades defined by Primary Phylogenetic Hypotheses (hereinafter PPHs). Third, plesiomorphic gene orders (or Hypothetical Taxonomic Units, hereinafter HTUs) are defined at all internal nodes. Fourth, in the solutions computed during step 2, if it exists a tree in which there is no possible gene order for some HTUs, this tree is not a solution and must be rejected (step 3). Thus, steps 2 and 3 are reiterated until all the incorrect solutions are excluded.

### Step 1—Genome comparison algorithm

Let *G* and *G’* be two genomes having exactly the same genes. We want to find a minimum path between *G* and *G’*. This problem is equivalent to the problem of sorting signed permutations using inversions, transpositions, and reverse transposition [28].

The program Genome_Comparison.c (S1 Appendix) uses the backtracking framework [41]: it enumerates all the possible paths of length *k* starting from *G* through a depth-first exploration of a search tree. Each path of length *k* leading to *G’* is a solution while any other path (not ending to *G’*) is a deadlock which causes backtracking in the search tree and exploration of another branch. The backtracking algorithm to find the paths from *G* to *G’* in *k* steps is given below.

**Backtracking algorithm: Computation of all the paths between *G* and *G’* in *k* steps**

**Main variables**

*state*        // current state of the automaton

*GL*                // data structure encoding the current path and associated problem

                        // *GL* includes the path from ***G*** to a current genome ***X*** in *r* steps

                        // the associated problem is the search of the paths from ***X*** to ***G’*** in (*k*-*r*) steps

**begin**

initialisations                        // reading and encoding of genomes ***G*** and ***G’***

                                                                        // initialisation of *GL* with a path of length 0

*number_of_solutions* ← 0

*state* ← 0                        // progression state (initial state)

*finished* ← false

**while** (*finished* = false)

**do                        case** (*state*) **of**

                                      0:                        // progression state

                                                **if** (the search tree has been totally explored)

                                                **then** *state* ← 1 // final state—the search tree has been totally explored

                                                **else** APPLY A HEURISTIC TEST to the current associated problem (encoded by *GL*)

                                                                        **if** (the heuristic test returns "YES") // *GL* is a solution

                                                                        **then** *state* ← 2 // success state—a solution has been found

                                                                        **else if** (the heuristic test returns "INDETERMINATE")

                                                                                                **then** calculate next possible rearrangement *M* to apply to ***X***

                                                                                                                        ***X'*** ← *M*(***X***)

                                                                                                                        *extend GL* with the last step from ***X*** to ***X'***

                                                                                                                                                                        // *GL* = new current path (and associated problem)

                                                                                                                        *state* ← 0 // progression state

                                                                                                **else** // the heuristic test returns "NO":

                                                                                                                                                // *GL* is not a solution, and cannot lead to a solution

                                                                                                                                                *state* ← 3 // backtrack state

                                                              1: // final state—the search tree has been totally explored

                                                              *finished* ← true

                                                              **if** (*number_of_solutions* = 0)

                                                              **then** print "no solution"

                                                              **else** print "no other solutions"

                                                              2: // success state—a solution has been found

                                                              print the solution

                                                              *number_of_solutions* ← *number_of_solutions* +1

                                                              **if** (*want*_*all_solutions* = true)

                                                              **then** *state* ← 3 // backtrack state

                                                              **else** *finished* ← true

                                                              3: // backtrack state—backtracking in the search tree

                                                              remove the last step of *GL*

                                                              *state* ← 0 // progression state

**end**

In state 0 (progression state), in order to calculate the next rearrangement to be applied to the current genome ***X***, all possible rearrangements must be enumerated. The program does not consider the gain/loss and calculates the paths between two mt gene orders reduced to their common genes (with the same number of genes) only using transposition, reverse transposition and inversion. Then, and still with completeness, the model generator calculates the tree solutions that fit with the distances (Step 2). Because gain/loss are rare and obvious, the solutions trees are determined discarding these rearrangements which are inserted *a posteriori*.

**Fig 1:**
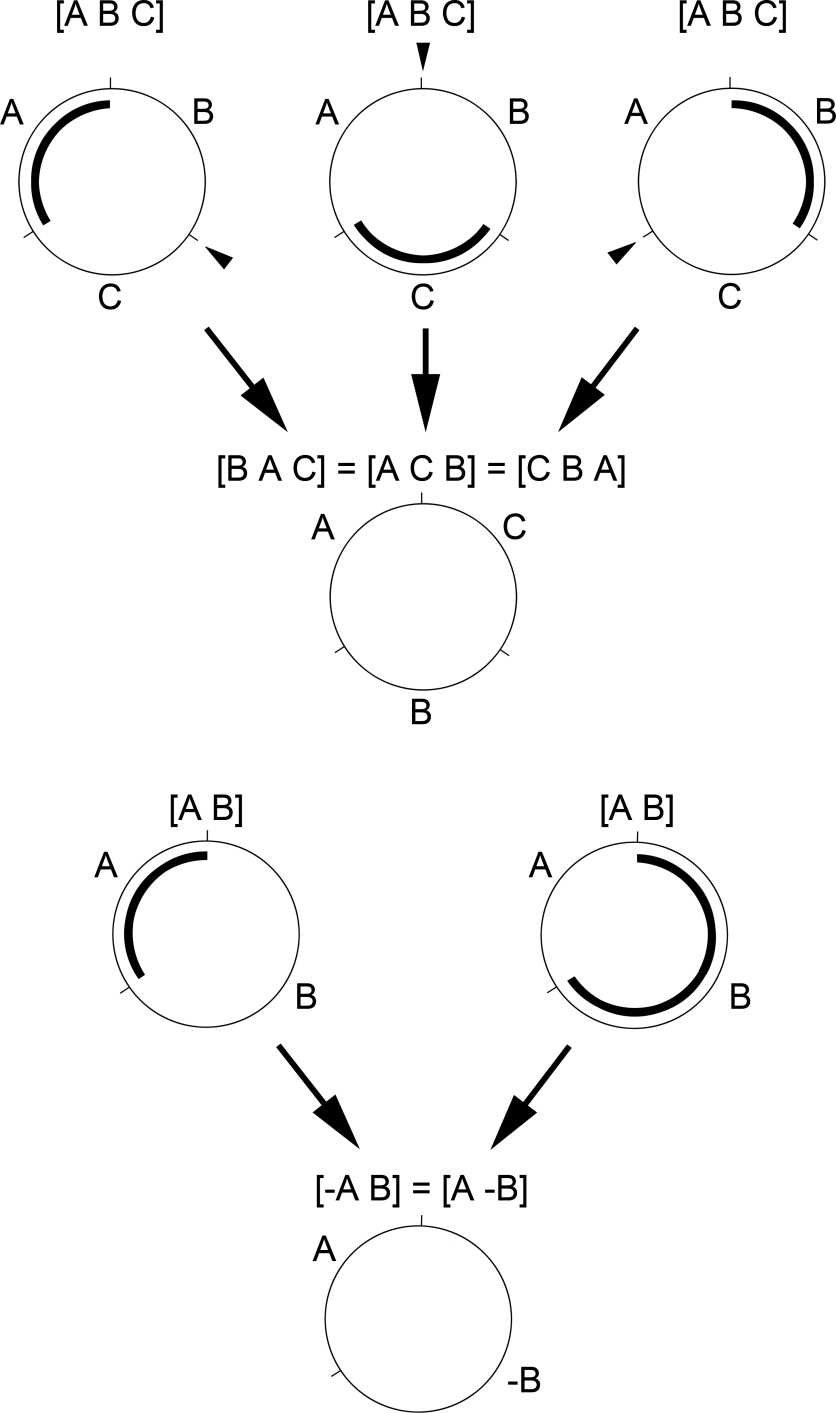
**Diagram of three possible transpositions (top) and two possible inversions (bottom) in a circular genome leading to the same gene order. A.** Because of the circularity of the genome, there are always three possible transpositions leading to a similar gene order ([*B A C*] = [*A C B*] = [*C B A*]) from a given gene order ([*A B C*]). Thus, it is not possible to determine which block of genes is concerned by a transposition. **B.** Similarly, there are always two possible inversions leading to a similar gene order ([-*A B*] = [*A* -*B*]) from a given gene order ([*A B*]). Thus, it is not possible to determine which block of genes is inverted (*A* or *B*). In each example, the transposed or inverted block is underlined. The black arrowheads indicate where the transposed block is inserted. By convention, the circular genomes are read clockwise.

Due to genome circularity, there always exists, from a given gene order, three distinct transpositions or two distinct inversions leading to a single gene order (Fig 1). A formal proof of these equivalent transpositions is given by Hartmann et al. [42]. Equivalent inversions have been studied by Meidanis et al. [43]. Therefore, the program arbitrarily chooses one possibility among them when it enumerates a succession of equivalent transpositions or inversions. The sorting problem solved by the backtracking algorithm presented here has already been studied by several authors using the Integer Linear Programming framework [44, 45].

In state 0 (progression state), we apply a heuristic test as a function that returns:

- YES if the path encoded in *GL* is a solution, *i*.*e*., a path from *G* to *G’* in *k* steps. In this case, the solution is displayed (State 2).
- NO if the path encoded in *GL* is not a solution and cannot be extended to construct a solution. In this case, the algorithm backtracks (State 3) and does not need to explore the search subtree from the current path *GL* because it cannot lead to a solution.
- INDETERMINATE otherwise. In this case, the algorithm extends the current path *GL* by listing all the possible rearrangements it can apply to the current genome *X*.

Because of the exponential complexity of the backtracking algorithm, the computation time can be very long for paths with numerous rearrangement steps. The computation time can, however, be strongly reduced when using two mathematical properties as heuristic tests, the shared block and lower bound properties.

The shared block property was used in the program Genome_Comparison.c as follows. At each step of the computation of the shortest path between a genome *G* and a genome *G’*, the blocks that are present in the current genome *X* and the genome *G’* are calculated first; the only rearrangements that are considered to be potentially applied to the current genome *X* are those that do not intersect the blocks present in *X* and *G’*.

The lower bound property was used in the program Genome_Comparison.c on the basis of the following contrapositive: let *G* and *G’* be two distinct genomes. Let *k* > 0. If *nb_breakpoints*(*G*, *G’*) > 3 × *k*, then there is no *k*-path between *G* and *G’*. Indeed, for each current genome *X* explored in the state 0 of the automaton (progression state), the function HEURISTIC_TEST returns NO if *nb_breakpoints*(*X*, *G’*) > 3 × (number of steps remaining between *X* and *G’*). In this case, the algorithm does not need to explore the search subtree from the current path, as it never leads to *G’* with the remaining number of steps.

To illustrate the influence of the two previous properties used as heuristic test, we carried out a comparison of the computation times with and without these properties (S2 Appendix).

This comparison shows that the program Genome_comparison.c is efficient only if the shared block property (HT1) and the lower bound property (HT2) are used as heuristic tests. Note that these two properties are complementary: HT1 is very efficient for couples of mt gene orders with a small number of breakpoints, while HT2 is very efficient for couples of mt gene orders with a large number of breakpoints. Without HT1 and HT2, it is not possible to calculate minimal distances greater than 3 in a reasonable time. All computations were done on a laptop with a 2.5 GHz processor and 4 Go of RAM.

### Step 2—Tree computation

Formal logic allows studying a problem with a hypothetico-deductive approach that permits the enumeration of all the solutions of the problem under study, to then assess working hypotheses or answer specific questions, all with the same program [46]. A finite model generator is a program that computes all the solutions of a set of first-order logical formulas representing the problem. Several model generators exist [47], and their common characteristics are correctness (the solutions are correct), completeness (all the solutions are listed), and decidability (all computations end, an obvious property for finite domains). PHYLO can be axiomatised by writing a set of first-order logic formulas that defines a connected and acyclic graph *T* (dendrogram or tree) with a minimum number of rearrangements between all the distinct mtDNAs of a given taxonomic dataset. In this tree *T*, each node represents a mtDNA, while each edge represents a rearrangement between two linked nodes.

To define *T*, we use a relation *R*(*x*, *y*) such that: *R*(*x*, *y*) is true if and only if there is an edge in *T* between the nodes *x* and *y*. The set of all possible values for the variables *x* and *y* is called the domain (the node set of the tree).

Finally, PHYLO consists in finding the most parsimonious tree *T* (*i*.*e*., defined on the smallest possible domain containing at least the complete taxonomic dataset) which satisfy the following properties:

**Property P1**—*T* is simple (the relation *R* is not reflexive, *i*.*e*., *R*(*x*, *x*) is false).

**Property P2**—*T* is non-oriented (*R* is symmetrical, *i*.*e*., *R*(*x*, *y*) implies *R*(*y*, *x*)).

**Property P3**—*T* is connected and acyclic (*T* is a tree).

**Property P4**—*T* respects the distance matrix: for each pair of genomes *G* and *G’* belonging to the taxonomic dataset, the path length between the nodes corresponding to *G* and *G’* in *T* is always greater than or equal to the distance between *G* and *G’*.

**Property P5**—*T* verifies additional constraints (PPHs), conditional on choosing a root node to define the hierarchical levels in the tree. In other words, the PPH imposes the existence of given monophyletic groups. For this study, 32 PPHs were used (S3 Appendix). They all are well-admitted phylogenetic hypotheses associated to well-known taxa.

**Property P6**—The possible mtDNA organisations are calculated for each HTU in *T* (the ancestral states that are not represented in the dataset).

The model generator computes all the trees *T* that satisfy the properties P1 to P5 considered as axioms of PHYLO. Property P6 is verified *a posteriori* for each tree.

Extra-logical constraints can be implemented in model generators to improve the performance. These constraints can replace a group of logical formulas having exactly the same meaning and are defined by an algorithmic process [48]. This feature is supported by the Davis and Putnam model generator [49, 50]. For instance, the property P3 (the graph is connected and acyclic) can be replaced by a constraint which verifies that *(i)* the graph is connected and *(ii)* the number of nodes equals the number of edges plus 1. Similarly, the properties P4 and P5 are preferably expressed in the form of constraints rather than logical formulas.

In the present work, we used an experimental model generator belonging to the Davis and Putnam type with symmetry breaking techniques [51, 52]. This model generator is correct, complete and decidable and supports constraints; any model generator with similar characteristics may also be suitable to solve PHYLO.

### Step 3—Calculating the hypothetical common ancestors (verifying P6)

We analyse each tree got at Step 2 to infer hypothetical ancestral genomes (also called ground patterns). In each tree with *V* nodes, there are *N* nodes (*N* < *V*) representing the *N* genomes belonging to the taxonomic dataset (Operational Taxonomic Units, OTUs), and *M* additional nodes that represent ancestral states (Hypothetical Taxonomic Units, HTUs), *i*.*e*., *V* = *N* + *M*. Determining hypothetical ancestral genomes consists of enumerating all possible gene orders for the *M* HTUs of the tree (and thus proving that the tree verifies property P6), or on the contrary by proving that there is at least one HTU in the tree for which no gene order can be found (in this case the tree does not verify property P6, and therefore is not valid). To enumerate all the possible gene orders of the *M* HTUs, all the paths of length *k* linking two OTUs *G* and *G’* must be recalculated such that the path between *G* and *G’* is of length *k* and passes only through HTUs (the program Genome_Comparison.c enumerates all the paths with the shared block property disabled). It appears that two cases are possible for each HTU *X*:

1. There is a unique OTU *G* such that *X* appears in all the recalculated paths between *G* and other mtDNAs and only in these paths. In this case, all possible gene orders for *X* appear in the branch which leads to *G*.
2. Otherwise, there exist at least three OTUs such that *X* appears as an intermediate step in the recalculated paths between them. *X* is at a branching node or between two branching nodes. A lack of common gene orders for *X* means that the tree is not a valid solution because it contains a subtree *S* that does not verify P6. Any other tree solution containing this subtree is excluded.

### Step 4—Determining the complete set of tree solutions

For each invalid subtree *S*, an additional constraint is programmed into the model generator to rule out the solutions containing *S*. Trees are recalculated (Step 2) and verified (Step 3), possibly leading to the discovery of other invalid minimum subtrees and thus to the addition of new constraints to recalculate the solutions. The complete set of solutions is determined by iterating this process and eliminating all the trees that do not verify P6. A result verified by all the solutions is called a logical consequence. In contrast to existing methods used to analyse gene orders that only provide incomplete results, a complete logical approach will enumerate all the solutions and highlight the logical consequences. In practice, calculating all these solutions is possible only for a small taxonomic dataset (model generation is NP-hard). However, a broader taxonomic dataset can be used by combining all the solutions for smaller subdatasets. In the present study, the solutions for the Bilateria have been obtained by the combination of 36 computations (see S4–S8 Appendices).

## Results and discussion

Using the shared block and lower bound properties as heuristic tests, it was possible to calculate the exact distances between all pairs of mtDNAs present in the taxonomic dataset (Table 1) from the order of protein-coding and ribosomal RNA mt genes considering transposition, inversion, reverse transposition and gain/loss (S4 Appendix).

**Table 1:** Species, systematic position, and accession number of mitochondrial genomes used for gene order comparisons. *Cucumaria miniata* has the same order of protein-coding and ribosomal RNA genes as *Strongylocentrotus purpuratus* and is only used for comparisons including the transfer RNA genes.

Species	Taxonomy	Accession no.
*Tethya actinia*	Porifera	AY_320033
*Sipunculus nudus*	Sipunculida	FJ_422961
*Urechis caupo*	Echiura	NC_006379
*Platynereis dumerilii*	Annelida/Polychaeta	NC_000931
*Phoronis architecta (*syn. *psammophila)*	Phoronida	AY368231
*Terebratalia transversa*	Brachiopoda	NC_003086
*Terebratulina retusa*	Brachiopoda	NC_000941
*Laqueus rubellus*	Brachiopoda	NC_002322
*Lingula anatina*	Brachiopoda	AB178773
*Gyrodactylus derjavinoides*	Platyhelminthes/Trematoda	NC_010976
*Schistosoma mansoni*	Platyhelminthes/Trematoda	NC_002545
*Katharina tunicata*	Mollusca/Polyplacophora	NC_001636
*Biomphalaria glabrata*	Mollusca/Gastropoda	NC_005439
*Cepaea nemoralis*	Mollusca/Gastropoda	NC_001816
*Albinaria caerulea*	Mollusca/Gastropoda	NC_001761
*Nautilus macromphalus*	Mollusca/Cephalopoda	NC_007980
*Loligo bleekeri*	Mollusca/Cephalopoda	NC_002507
*Siphonodentalium lobatum*	Mollusca/Scaphopoda	NC_005840
*Graptacme eborea*	Mollusca/Scaphopoda	NC_006162
*Venerupis philippinarum*	Mollusca/Bivalvia	NC_003354
*Mytilus edulis*	Mollusca/Bivalvia	NC_006161
*Lampsilis ornata*	Mollusca/Bivalvia	NC_005335
*Inversidens japanensis*	Mollusca/Bivalvia	AB055624
*Loxocorone allax*	Entoprocta	NC_010431
*Flustrellidra hispida*	Bryozoa/Ectoprocta	NC_008192
*Watersipora subtorquata*	Bryozoa/Ectoprocta	NC_011820
*Bugula neritina*	Bryozoa/Ectoprocta	NC_010197
*Paraspadella gotoi*	Chaetognatha	NC_006083
*Spadella cephaloptera*	Chaetognatha	NC_006386
*Sagitta enflata*	Chaetognatha	NC_013814
*Sagitta nagae*	Chaetognatha	NC_013810
*Leptorhynchoides thecatus*	Rotifera/Acanthocephala	NC_006892
*Caenorhabditis elegans*	Nematoda	NC_001328
*Trichinella spiralis*	Nematoda	NC_002681
*Priapulus caudatus*	Priapulida	NC_008557
*Epiperipatus biolleyi*	Onychophora	NC_009082
*Limulus polyphemus*	Arthropoda/Xiphosura	NC_003057
*Steganacarus magnus*	Arthropoda/Arachnida	NC_011574
*Dermatophagoides pteronyssinus*	Arthropoda/Arachnida	NC_012218
*Leptotrombidium akamushi*	Arthropoda/Arachnida	NC_007601
*Nymphon gracile*	Arthropoda/Pycnogonida	NC_008572
*Narceus annularis*	Arthropoda/Myriapoda	NC_003343
*Ligia oceanica*	Arthropoda/Crustacea	NC_008412
*Argulus americanus*	Arthropoda/Crustacea	NC_005935
*Speleonectes tulumensis*	Arthropoda/Crustacea	NC_005938
*Tigriopus japonicus*	Arthropoda/Crustacea	NC_003979
*Vargula hilgendorfii*	Arthropoda/Crustacea	NC_005306
*Eriocheir sinensis*	Arthropoda/Crustacea	NC_006992
*Megabalanus volcano*	Arthropoda/Crustacea	NC_006293
*Cherax destructor*	Arthropoda/Crustacea	NC_011243
*Pagurus longicarpus*	Arthropoda/Crustacea	NC_003058
*Chinkia crosnieri*	Arthropoda/Crustacea	NC_011013
*Balanoglossus carnosus*	Enteropneusta	NC_001887
*Xenoturbella bocki*	Xenoturbellida	NC_008556
*Florometra serratissima*	Echinodermata/Crinoidea	NC_001878
*Antedon mediterranea*	Echinodermata/Crinoidea	NC_010692
*Gymnocrinus richeri*	Echinodermata/Crinoidea	NC_007689
*Asterina pectinifera*	Echinodermata/Asteroidea	NC_001627
*Ophiura lukteni*	Echinodermata/Ophiuroidea	NC_005930
*Ophiopholis aculeata*	Echinodermata/Ophiuroidea	NC_005334
*Cucumaria miniata*	Echinodermata/Holothuroidea	NC_005929
*Strongylocentrotus purpuratus*	Echinodermata/Echinoidea	NC_001453
*Doliolum nationalis*	Chordata/Tunicata	NC_006627
*Phallusia fumigata*	Chordata/Tunicata	NC_009834
*Phallusia mammillata*	Chordata/Tunicata	NC_009833
*Ciona savignyi*	Chordata/Tunicata	NC_004570
*Ciona intestinalis*	Chordata/Tunicata	NC_004447
*Halocynthia roretzi*	Chordata/Tunicata	NC_002177
*Asymmetron inferum*	Chordata/Cephalochordata	NC_009774
*Homo sapiens*	Chordata/Craniata	NC_012920

Nine phyla included in this dataset exhibit highly variable mt gene orders, *e*.*g*., Hemichordata, Annelida, Brachiopoda, Chaetognatha, Bryozoa, Entoprocta, Rotifera, Mollusca and Porifera as well as in subtaxa belonging to phyla with conservation of mt gene order, *e*.*g*., Tunicata within Chordata, Bivalvia within Mollusca, Myriapoda within Arthropoda, and Enoplea within Nematoda [53]. Fast-evolving taxa have often been recognized as problematic in phylogenetic studies based on primary sequences because of long-branch attraction artefact [54]. Although the bias introduced by these taxa in gene order analysis has not been thoroughly addressed in previous studies, an increase in the number of rearrangements necessarily increases the risk of homoplasy with subsequent loss of phylogenetic signal. One of the most obvious strategies is the removal of the fast-evolving species (or characters) from the analysis. Interestingly, the distances computed with the program Genome_Comparison.c between the mtDNAs of these shuffled genes phyla were usually greater than 5 (S4 Appendix). To highlight this putative threshold, we carried out empirical tests. A first simulation was made with 30,944 pairwise comparisons of randomly generated gene orders with 15 genes. The distances were 4 in 4 cases, 5 in 260 cases (0.84%), 6 in 6158 cases (19.90%), 7 in 24,499 (79.17%) cases and 8 in 23 cases. In a second simulation with random genomes containing 14 genes we obtained a distance 4 in 32 cases, 5 in 1607 cases (4.56%), 6 in 18,910 cases (53.64%) and 7 in 14,704 cases (41.71%). The high probability of obtaining a distance greater than or equal to 6 for pairwise comparisons of random genomes means that when the distance between two mtDNAs is greater than 5, the risk of underestimating the true number of rearrangements is high. So we removed from the analysis twenty one mtDNAs which were at distance greater than 5 from more than 95% of the other mtDNAs: all tunicates (*Ciona intestinalis*, *Ciona savignyi*, *Doliolum nationalis*, *Halocynthia roretzi*, *Phallusia fumigata*, *Phallusia mammillata*), one copepod (*Tigriopus japonicus*), one nematode (*Caenorhabditis elegans*), all scaphopods (*Graptacme eborea*, *Siphonodentalium lobatum*), all lamellibranchs (*Inversidens japanensis*, *Lampsilis ornata*, *Mytilus edulis*, *Venerupis philippinarum*), all platyhelminthes (*Gyrodactylus derjavinoides*, *Schistosoma mansoni*), one acanthocephalan (*Leptorhynchoides thecatus*), two bryozoans (*Flustrellidra hispida*, *Watersipora subtorquata*) and two brachiopods (*Laqueus rubellus*, *Lingula anatina*).

### Reconstruction of mtDNA evolutionary history: The deuterostomes as a study case

**Fig 2:**
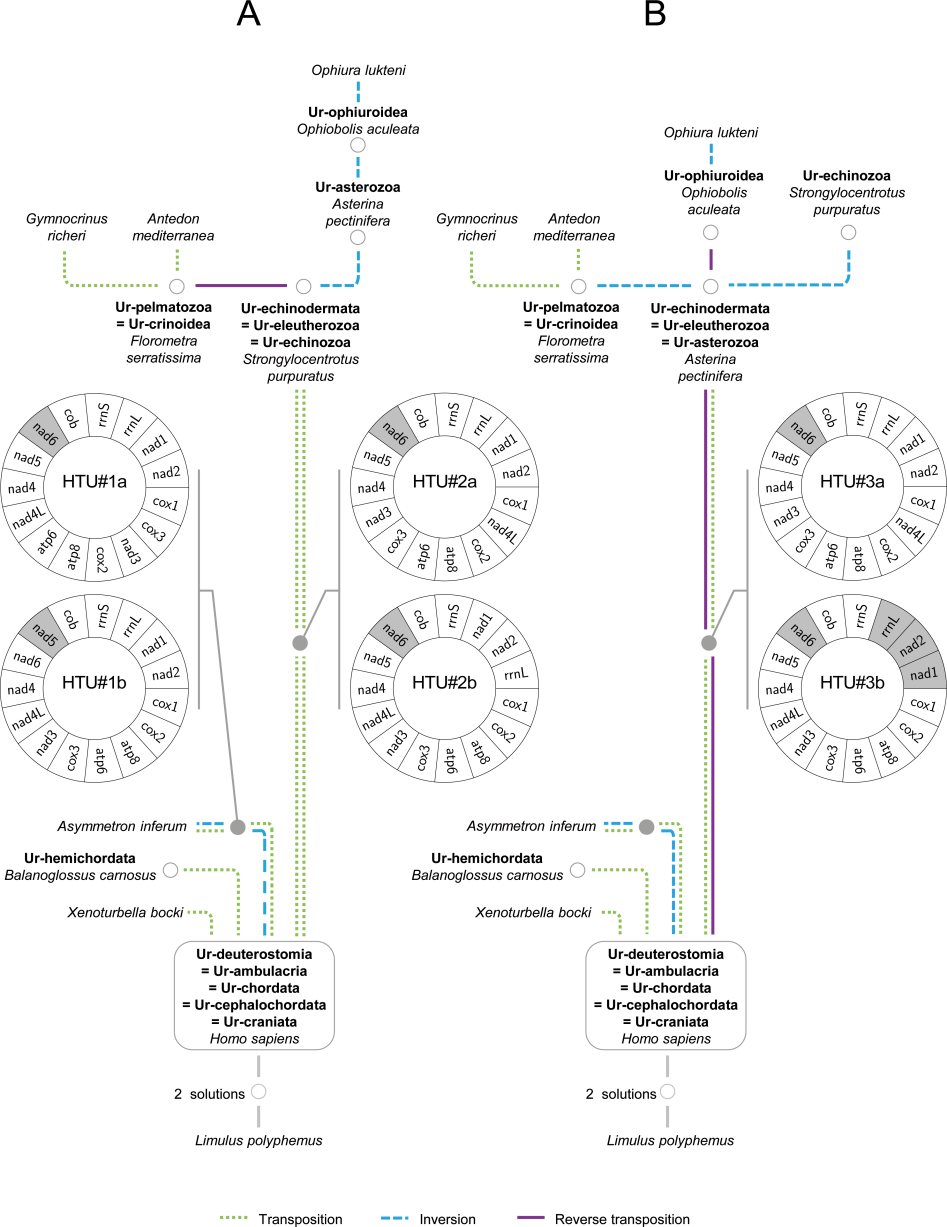
The two most parsimonious trees for deuterostomes (12 evolutionary steps) deduced from the order of protein-coding and ribosomal RNA mitochondrial (mt) genes. The maximum parsimony rearrangement events among the trees are indicated by different lines (blue dashed line, inversion; green dashed line, transposition; purple solid line, reverse transposition). Hypothetical ancestral mtDNAs (HTUs) are indicated by grey shaded dots. Grey-circled white dots indicate HTUs that correspond to ground patterns of clades. Ur-echinodermata is represented by the mtDNA of either *Strongylocentrotus purpuratus* (A) or *Asterina pectinifera* (B). Grey-shaded boxes on diagrammatic representations of hypothetical ancestral mtDNAs (HTU#1a to 3b) highlight genes transcribed from the opposite strand.

Different combinations of outgroups were assessed to explore their effects on the attachment point to the root and examine whether the rooting choice would affect the topology of optimal tree(s). First, we computed the deuterostome trees rooted by *Limulus polyphemus* (Arthropoda, Ecdysozoa). Then, we performed analyses rooted with one or two additional taxa, (*Limulus polyphemus*, *Katharina tunicata–*Mollusca, Lophotrochozoa) and (*Limulus polyphemus*, *Katharina tunicata*, *Tethya actinia–*Porifera). All the computations are detailed in the logbook 1 (S5 Appendix). As the nature of the outgroup did not change the topologies of the optimal tree solutions, we only present the trees obtained from the first computation rooted on *Limulus polyphemus*. The computation resulted in only six distinct solutions (S6 Appendix, section ‘deuterostomes_taxA_v2_6sol’) in which the internal relationships of deuterostomes were identical except some variations within Echinodermata. These variations included three topologies for the Crinoidea combined with two topologies for the rest of the Echinodermata (Fig 2) which consisted of different positioning of *Asterina pectinifera* and *Strongylocentrotus purpuratus*, for which the mtDNAs corresponded to Ur-echinodermata in all solutions.

In an attempt to reduce the number of solutions and work out the ground pattern of Echinodermata, we used three additional alternative PPHs:

- PPH#30a (Echinoidea and Asteroidea) [55]. In this first hypothesis, the Asteroidea are placed as sister group to the clade Echinoidea and Holothuroidea.
- PPH#30b (Ophiuroidea and Echinoidea): Cryptosyringid [56]. In this second hypothesis, the Ophiuroidea are placed as sister group to the clade Echinoidea and Holothuroidea.
- PPH#30c (Asteroidea and Ophiuroidea): Asterozoa [57]. In this third hypothesis, the Asteroidea are placed as sister group to Ophiuroidea.

When computations were constrained with PPH#30a or PPH#30b we still found three topologies corresponding to three different relationships within Crinoidea, but *Asterina pectinifera* always appeared as Ur-echinodermata (Fig 2B). With PPH#30c, either *Asterina pectinifera* or *Strongylocentrotus purpuratus* represents Ur-echinodermata within distinct but equiparsimonious trees (Fig 2). These results contradict the conclusions drawn by Scouras & Smith [13] and Perseke et al. [55] who proposed that the mtDNA ground pattern of the Ophiuroidea, Crinoidea, and the group of Echinoidea, Holothuroidea, and Asteroidea could be derived from a hypothetical ancestral crinoid gene order. Previous analyses based on mt gene order have also suggested that the ground pattern of echinoderms most likely resembles the echinoid mtDNA [12, 58]. However, here we showed that either Echinoidea or Asteroidea might represent the echinoderm ground pattern.

Three topologies exhibited different relationships within Crinoidea, specifically between *Antedon mediterranea* and *Gymnocrinus richeri* with *Florometra serratissima* basal to Crinoidea (S6 Appendix, section ‘deuterostomes_taxA_v2_6sol’). For a local analysis of Crinoidea, tRNA genes have been included for computations. There are up to 22 tRNA genes added to the 15 protein-coding and rRNA genes. Therefore, including the tRNAs into the path computation between two mtDNAs increases the computation time to a point that makes the procedure unfeasible, unless the mtDNAs compared are very close. For example, the path computation between *Florometra serratissima* and *Antedon mediterranea* is fast, as the distance is 3. However, in the path between *Asterina pectinifera* and *Homo sapiens*, the distance is at least 11, whereas it is only 2 when considering only protein-coding and ribosomal genes. When *Asterina pectinifera* or *Strongylocentrotus purpuratus* are used as outgroups, the analysis including the tRNA genes yielded a single unique topology for the Crinoidea (S6 Appendix, sections ‘with_tRNA_crinoids_taxA_1sol’ and ‘with_tRNA_crinoids_taxB_1sol’), as represented in Fig 2.

Finally, we performed computations regarding several phylogenetic hypotheses on *Xenoturbella bocki* relationships. Acoelomorph flatworms related to *Xenoturbella bocki* were initially placed within deuterostomes [59] but several conflicting hypotheses are still under debate. A first study based on the analysis of newly sequenced mtDNAs [60] provided no support for a sister group relationship between Xenoturbellida and Acoela or Acoelomorpha and suggested an unstable phylogenetic position of *Xenoturbella bocki* as sister group to or part of the deuterostomes. More recently, two phylogenomic analyses have grouped *Xenoturbella* with acoelomorphs = Xenacoelomorpha) and suggested that Xenacoelomorpha could be the sister group of Nephrozoa [61, 62] or Protostomia [61]. In our contribution, whatever the PPH used to constrain the position of *Xenoturbella bocki* (*i*.*e*., whether it is sister group to or part of the deuterostomes), its mt gene order is always derived from an ancestor exhibiting a mtDNA identical to that of *Homo sapiens* (S6 Appendix). Hence our results corroborate the conclusion that the arrangement of protein-coding and rRNA genes in the mtDNA of *Xenoturbella bocki* is plesiomorphic [63] and therefore does not contain relevant signal to assess the phylogenetic relationships of this species.

As a result of using PPHs to constrain the relationships within echinoderms and tRNA genes to decipher Crinoidea relationships, only two trees were finally validated for deuterostomes (Fig 2). The major advantage of a complete method is that all the values of HTUs that are by definition not present in the taxonomic dataset are enumerated. Such a comprehensive and correct enumeration is not possible in traditional probabilistic approaches or by manual pairwise comparisons. In the case of the deuterostomes, the solutions contained only two HTUs, the first in the lineage leading to cephalochordates and the second in the one leading to the echinoderms (Fig 2). Each HTU has two possible gene orders because of the commutative property of both paths described. For each path, the HTUs represent a ground pattern that characterizes an ancestor or a current mtDNA that has not been sequenced yet. Interestingly, the gene orders of HTU#2a and HTU#3a (see Fig 2) that stand for two distinct paths between Craniata and Echinodermata have already been characterised in a previous study and were considered as the echinoderm consensus [58].

To summarise, below are listed the key results that are logical consequences of PHYLO:

- The monophyly of Chordata, Echinodermata, Ophiurida and Crinoidea are always verified.
- There is only one subtree for Cephalochordata; this subtree has *Homo sapiens* mtDNA as Ur-cephalochordata.
- There is only one subtree for Ophiuroidea; this subtree has *Ophiobolis aculeata* mtDNA as Ur-ophiuroidea.
- There is only one subtree for Hemichordata and *Xenoturbella bocki*.
- There is only one subtree for Crinoidea (when using tRNA genes); in this subtree, *Florometra serratissima* mtDNA represents Ur-crinoidea.
- *Homo sapiens* mtDNA represents Ur-deuterostomia, Ur-chordata, Ur-cephalochordata and Ur-ambulacria.

### The use of tRNA genes to solve the PHYLO problem

**Fig 3:**
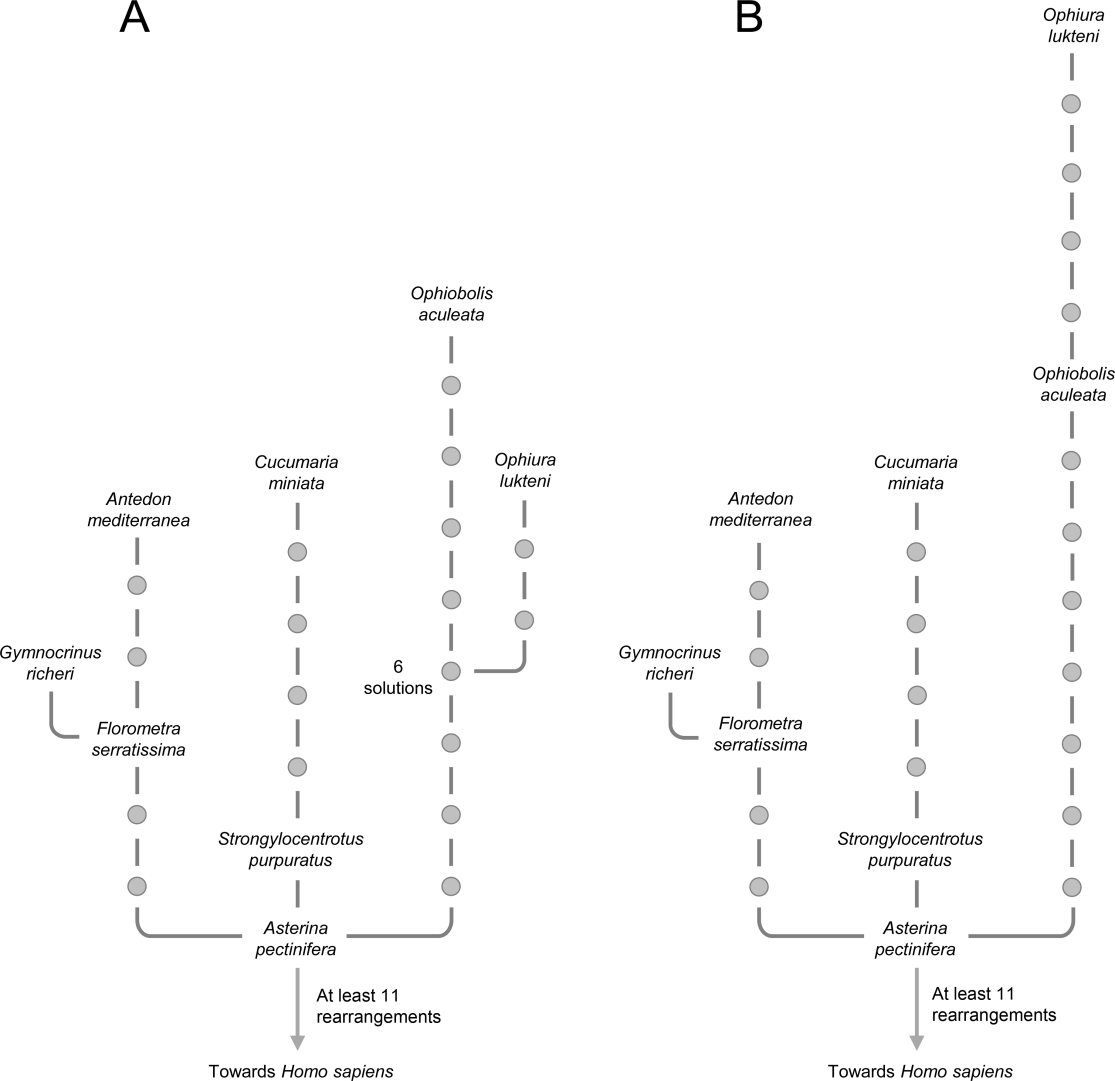
Two trees among extant Echinodermata as deduced from the order of protein-coding, ribosomal RNA(rRNA) and transfer RNA (tRNA) mitochondrial genes. (A) One tree solution for the whole Echinodermata group calculated with mitochondrial genes (including tRNA genes). Among the 25 necessary steps, more than 6 involved the mitochondrial protein-coding and rRNA genes. (B) Tree solution calculated with mitochondrial protein-coding and rRNA genes with *Asterina pectinifera* as Ur-echinodermata (Fig 2B) on which 20 necessary rearrangements of tRNA genes have been added *a posteriori*. Among the 26 steps, 6 involved the mitochondrial protein-coding and rRNA genes.

The tRNA genes are often omitted in the comparison of mt gene orders due to their high evolutionary rate. However, the order of tRNAs does contain phylogenetic information in some contexts [19, 64, 65] and should be considered in rearrangement models to decrease the number of solutions, as for instance in Crinoidea. The computation of the Echinodermata trees with all mt genes was possible but could not be held with completeness. The reconstruction of one tree with all mt genes with *Asterina pectinifera* as Ur-echinodermata (S6 Appendix, sections ‘with_tRNA_eleutherozoa_taxA_2sol’ and ‘with_tRNA_ophiurida_taxA_1sol’) was tractable and required 25 evolutionary steps (Fig 3A). This topology was slightly different (different branching within Ophiuroidea) from the topology obtained with the protein-coding and rRNA genes on which specific rearrangement of tRNA genes have been added *a posteriori* (Fig 3B). The ancestral state of Ophiuroidea has been shown to be difficult to infer and remains unresolved [64, 65] but it has been suggested that *Ophiura lutkeni* has a more derived gene order than *Ophiobolis aculeata* [64]. While the scenarios computed with protein-coding and rRNA genes always favoured the gene order of *Ophiobolis aculeata* as the Ophiuroidea ground pattern (Figs 2 and 3B), the topology obtained with the inclusion of tRNA genes proposed 6 additional ground patterns (Fig 3A) with a more derived position for *Ophiobolis aculeata* than *Ophiura lutkeni*. All of the 14,641 possible paths between *Strongylocentrotus purpuratus* and *Cucumaria miniata* are represented by a succession of five tRNA transpositions (Fig 3). A TDRL encompassing the control region, the tRNA cluster, NADH dehydrogenase subunits 1 and 2, the large rRNA, the cytochrome oxidase subunit I and tRNA Arg has been previously proposed in the path between Echinoidea and Holothuroidea [65, 66]. Only both copies of the putative control region sequence have been maintained. For reducing the two cluster copies to a single set of functional genes this hypothesis needs at least 9 rearrangements for tRNA genes (1 tandem duplication and eight independent random losses), a scenario which is less parsimonious than the rearrangement based on transposition only. Nevertheless, whatever the hypothesis selected, the topology and HTUs of the tree solutions will be the same because both the TDRL and tRNA transpositions constitute autapomorphic rearrangements for Holothuroidea.

The computation including tRNA genes (Fig 3A) raised interesting remarks. Indeed, in this last analysis, the total number of rearrangements was the most parsimonious (25 rearrangements instead of 26 obtained from a computation with protein-coding and rRNA genes only, see Fig 3B). However, the total number of rearrangements concerning the protein-coding and rRNA genes increased within the most parsimonious tree computed with all mt genes (Fig 3A, more than 6 rearrangements) when compared with the tree computed without tRNA genes (Fig 3B, 6 rearrangements). Parsimony is the principle according to which, all other things being equal, the best hypothesis to consider is the one that requires the fewest evolutionary steps. However, the reasonableness of the parsimony assumption in a given context may have nothing to do with its reasonableness in another one. In other words, when using the parsimony principle to decipher evolutionary hypotheses, the outcome depends on the set of characters considered. Nearly 80% of all the rearrangements that have happened involve tRNA genes. Given this high percentage and in an attempt to minimize the global number of rearrangements (*i*.*e*., if we are looking for parsimonious trees that takes all mt genes into account), the influence of larger protein-coding and rRNA genes is negligible when compared to those of smaller tRNA genes. Hence, the trees obtained with the larger genes are expected to be significantly different than those obtained with all the genes (which should be very similar to the parsimonious trees obtained when using only tRNA genes). This suggests that even if the use of tRNA genes can be relevant for local resolutions, it is reasonable to rely predominantly on the larger mt genes with a lower evolutionary rate when calculating the tree solutions corresponding to deep and ancient lineages like in the case of deuterostomes or bilaterians.

### Towards logical analysis of mt gene orders in bilaterians

There were too many OTUs (47 bilaterians and 1 poriferan) to compute a single global analysis, but smaller computations that verify the convergence of results at each step were tractable. Using known monophyletic groups as PPHs (S3 Appendix), computations were carried out on taxa and subtaxa by recombining the resulting solutions in the hierarchical structure of the bilaterian phylogeny. The chronological description of all the computations is given in the Logbook 1 (S5 Appendix). Many equiparsimonious trees were obtained. Even though a unique representation of these topologies is not possible, the whole set of solutions can be enumerated (S6–S9 Appendices). These results constitute a database of all the possible solutions. Moreover, the number of possible solutions can be reduced, possibly down to a single one, by adding new PPHs.

In the case of Ecdysozoa, seven computations had to be carried out (S7 Appendix). After the recombination of these computations, 4212 equiparsimonious trees were obtained corresponding to 3 subtrees for Decapoda combined with 39 subtrees for the rest of Mandibulata, (3×39 = 117 subtrees for all Mandibulata), 9 subtrees for Chelicerata (comprising 6 subtrees for Acari, meaning 117×9 = 1053 subtrees for Arthropoda), 1 subtree for Onychophora (1×1053 = 1053 subtrees for Panarthropoda) and 4 subtrees for Introverta (4×1053 = 4212 trees for Ecdysozoa).

Concerning Lophotrochozoa, 12 computations were needed, leading to 81 tree solutions (S8 Appendix), including 3 subtrees for Gastropoda combined with 1 subtree for the rest of Mollusca (1×3 = 3 subtrees for Mollusca), 3 subtrees for the rest of Eutrochozoa, (3×3 = 9 for Eutrochozoa), 9 subtrees for Lophophorata (9×9 = 81 subtrees for Lophotrochozoa).

**Fig 4:**
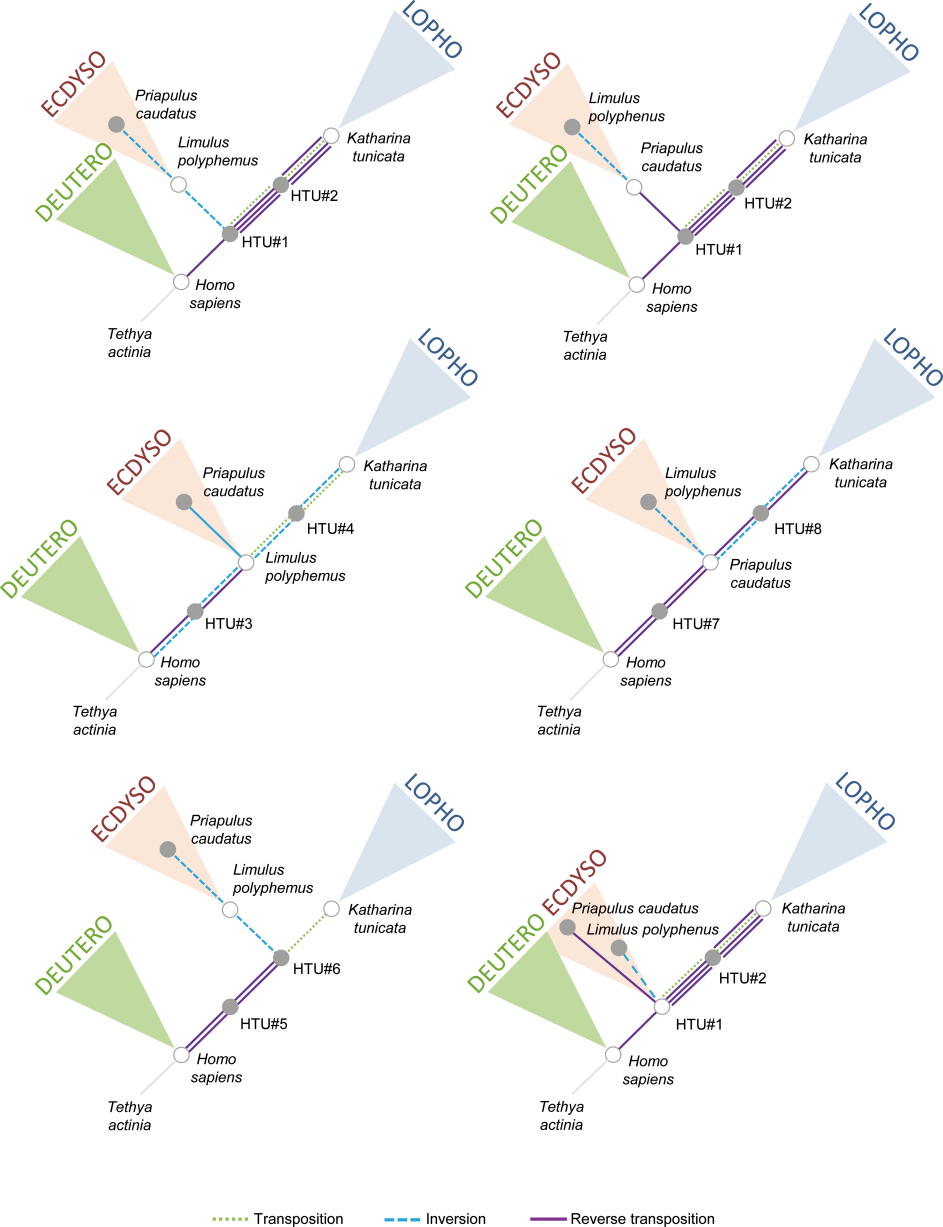
The six most parsimonious trees deduced from the order of protein-coding and ribosomal RNA mitochondrial (mt) genes in bilaterians. The rearrangements are indicated by different lines (blue dashed line, inversion; green dashed line, transposition; purple solid line, reverse transposition). Hypothetical ancestral mtDNAs (HTUs) are indicated at each node of the trees by grey shaded dots. Grey-circled white dots indicate HTUs that correspond to ground patterns of deuterostomes, ecdysozoans and lophotrochozoans. Gene orders of HTUs are indicated in Table 2.

The large amount of equiparsimonious trees obtained for the two main protostomian clades does not allow a single representation but the analyses provided the following logical consequences that are important results from a biological perspective:

- The monophyly of Acari, of Panarthropoda, and of Annelida are always verified.
- *Limulus polyphemus* mtDNA represents Ur-panarthropoda, Ur-arthropoda, Ur-mandibulata and Ur-chelicerata.
- There is only one solution for the set of rearrangements which links the mtDNA of Ur-panarthropoda (*Limulus polyphemus*) and the mtDNA of *Eriocheir sinensis* (transposition), *Narceus annularis* (transposition) and *Epiperipatus biolleyi* (6 possible paths, each with 3 rearrangements).
- Three ground patterns have been found for Ur-ecdysozoa which correspond either to the mtDNA of *Limulus polyphemus* or to *Priapulus caudatus* or to a hypothetical ancestor (see HTU#1 of Fig 4).
- There is only one solution for the cephalopods, with *Katharina tunicata* mtDNA as Ur-cephalopods linking *Nautilus macrocephalus* mtDNA (transposition) and *Loligo bleekeri* mtDNA (4 possible paths, each with 2 rearrangements).
- *Cepaea nemoralis* represents Ur-gastropoda.
- There is only one solution for the position of *Loxocorone allax* mtDNA (10 possible paths, each with 2 rearrangements) and *Phoronis architecta* mtDNA (transposition) with respect to *Katharina tunicata*.
- *Sipunculus nudus* (Sipunculida) is always the sister group of annelids (*Platynereis dumerilii* and *Urechis caupo*).
- *Katharina tunicata* mtDNA represents Ur-lophotrochozoa, Ur-eutrochozoa, Ur-mollusca, Ur-lophophorata and Ur-cephalopoda.

To give more insight into the deep branching of bilaterians, we carried out a computation rooted on *Tethya actinia* and using the respective gene order ground patterns of protein-coding and rRNA genes of ecdysozoans (*Limulus polyphemus*, *Priapulus caudatus* or HTU#1), lophotrochozoans (*Katharina tunicata*) and deuterostomes (*Homo sapiens*) as the representative of the three main bilaterian lineages. This strategy allowed enumerating with completeness only 6 equiparsimonious trees for Bilateria (Fig 4) and to highlight the following logical consequence: *Homo sapiens* mtDNA represents Ur-bilateria. Gene orders of protein-coding and rRNA genes of HTU#1 to 8 of Fig 4 are given in Table 2.

**Table 2:** Putative organisation of protein-coding and ribosomal RNA genes of hypothetical ancestral mitochondrial genomes represented on Fig 4.

#	Hypothetical mitochondrial gene orders														
**1, 3, 5, 7**	cox1	cox2	atp8	atp6	cox3	nad3	-nad5	-nad4	-nad4L	nad6	cob	rrnS	rrnL	nad1	nad2
**2**	cox1	cox2	atp8	atp6	-nad5	-nad4	-nad4L	nad6	cob	rrnS	rrnL	nad1	cox3	nad3	nad2
**2**	cox1	cox2	atp8	atp6	-nad5	-nad4	-nad4L	nad6	cob	-nad3	-cox3	rrnS	rrnL	nad1	nad2
**2**	cox1	cox2	atp8	atp6	nad6	cob	nad4L	nad4	nad5	-nad3	-cox3	rrnS	rrnL	nad1	nad2
**2, 4, 6**	cox1	cox2	atp8	atp6	cox3	nad3	-nad5	-nad4	-nad4L	-cob	-nad6	-nad1	-rrnL	-rrnS	nad2
**3**	cox1	cox2	atp8	atp6	cox3	nad3	nad4L	nad4	nad5	-nad6	cob	-nad1	-rrnL	-rrnS	nad2
**4, 8**	cox1	cox2	atp8	atp6	-nad5	-nad4	-nad4L	nad6	cob	-nad1	-rrnL	-rrnS	cox3	nad3	nad2
**5**	cox1	cox2	atp8	atp6	cox3	nad3	-nad5	-nad4	-nad4L	-cob	nad6	rrnS	rrnL	nad1	nad2
**7**	cox1	cox2	atp8	atp6	cox3	nad3	rrnS	rrnL	nad1	-cob	nad6	-nad5	-nad4	-nad4L	nad2
**8**	cox1	cox2	atp8	atp6	cox3	nad3	rrnS	rrnL	nad1	nad6	cob	nad4L	nad4	nad5	nad2

Ground patterns in Bilateria have been previously studied in Lophotrochozoa [35, 67], Ecdysozoa [68] and Deuterostomia [63]. It is noteworthy that these studies usually considered all the mt genes to draw their conclusions, which could explain some incongruence with the present results. Notably, it has been suggested that the ancestral gene order in Lophotrochozoa and Deuterosotomia cannot be found in extant species but rather represent a consensus between ingroup and outgroup mtDNAs [35]. Considering the protein-coding and rRNA genes, we showed that the ground patterns of Deuterostomia and Lophotrochozoa are realized in the respective mtDNAs of two extant species, *Homo sapiens* and *Katharina tunicata*. In Ecdysozoa, Ur-arthropoda is always realized in the mtDNA of *Limulus polyphemus* like it has been previously proposed [69]. In addition, the mtDNA of *Limulus polyphemus* should also be considered as the ground pattern of Panarthropoda and Ecdysozoa but our results also demonstrated that Ur-ecdysozoa could correspond to the mtDNA of *Priapulus caudatus* or to an inferred ancestral gene order (HTU#1) that is not realized in extant species. Priapulids have been described as an ancient clade and seem likely to adhere closely to the predicted ecdysozoan ground pattern [68]. Finally, the ground pattern of Bilateria was previously hypothesised and the order of the protein-coding and rRNA mt genes of *Homo sapiens* has been considered as Ur-bilateria [70]. It has been also suggested that the differences previously observed between vertebrate and arthropod mtDNAs are due mainly to gene rearrangements within the protostome lineages, a conclusion corroborated by our study.

Additional computations were carried out with four chaetognath mtDNAs added to the dataset described above (S10 and S11 Appendices). The position of chaetognaths was either basal to protostomes, ecdysozoans, or lophotrochozoans (34 possible topologies, S8 and S9 Appendices) and three logical consequences are emphasised:

- Chaetognatha mtDNAs were always grouped together (monophyly of Chaetognatha).
- Among the chaetognaths, the Sagittidae family is valid with *Flaccisagitta enflata* mtDNA as Ur-sagittidae.
- Chaetognatha mtDNAs cannot be basal to all bilaterians (the mt gene order of chaetognaths never derived directly from that of *Homo sapiens*).

Although it was possible to assert that chaetognaths were not the sister group of bilaterians, the topologies obtained are another reminder that the phylogenetic position of Chaetognatha is still one of the most problematic issues of bilaterian phylogeny [71].

### Conclusion

We presented for the first time a logical method to infer the evolution of mtDNA gene order and hypothetical ancestral configurations. This method has the benefit of both correctness and completeness, which is impossible by manual inspection when the distances between genomes are greater than one. At first, exploring all the possible trees might not seem to be a very elegant method, as it provides numerous solutions to the same problem. However, an understanding of the logical consequences can only be obtained through a complete enumeration of solutions and these logical consequences are, in themselves, extremely robust results. In our study of the bilaterian mtDNAs, we used the broadest and most indisputable PPHs which lead us to a high number of equiparsimonious trees. Our results showed that 8 among these 29 PPHs were logical consequences, *i*.*e*., they were always verified even when not previously imposed. The 21 PPHs imposing the monophyly of the following taxa were necessary: Bilateria, Deuterostomia, Ambulacria, Eleutherozoa, Ecdysozoa, Arthropoda, Mandibulata, Crustacea, Decapoda, Chelicerata, Introverta, Lophotrochozoa, Mollusca, Polyplacophora, Cephalopoda, Gastropoda, Eutrochozoa, Polychaeta, Echiura, Lophophorata, and Brachiopoda. By adding more PPHs for higher-level bilaterian taxa, the number of trees will decrease. Such a hypothetico-deductive approach was particularly fruitful to study the evolution of deuterostome mt gene order and should be applied to many other clades of bilaterians.

## Supporting information

S1 Appendix Source code of the program Genome_Comparison.c. (RAR)

S2 Appendix Influence of the shared block property and the lower bound for distance property used as heuristic tests (HT1 and HT2 respectively) on the computation time. (DOC)

S3 Appendix List of primary phylogenetic hypotheses (PPHs). (DOC)

S4 Appendix Distance matrix calculated with Genome_Comparison.c program. (TXT)

S5 Appendix Logbook 1—Chronological description of computations for Bilateria. (DOC)

S6 Appendix Axioms and solutions for Deuterostomia. (DOC)

S7 Appendix Axioms and solutions for Ecdysozoa. (DOC)

S8 Appendix Axioms and solutions for Lophotrochozoa. (DOC)

S9 Appendix Axioms and solutions for Bilateria. (DOC)

S10 Appendix Logbook 2—Chronological description of computations for Bilateria—Annex for Chaetognatha. (DOC)

S11 Appendix Axioms and solutions for Chaetognatha. (DOC)
