# Supplementary material for: A complete logical approach to resolve the evolution and dynamics of mitochondrial genome in bilaterians
Source: PLoS One. 2018 Mar 16;13(3):e0194334. doi: 10.1371/journal.pone.0194334 (PMC5856267; doi:10.1371/journal.pone.0194334)
Supplement: S2 Appendix — (DOC) [file pone.0194334.s002.doc]

**S2 appendix.** The distance computation (Table 1, standard program with HT1 and HT2) is faster than the computation of all the paths (Table 2, standard program with HT2) because it stops when the first path is found while the second computation enumerate all the paths, and also because the heuristic test HT1 is used in the first computation but not in the second one. The computation of all the paths (Table 2, standard program with HT2) is faster for couple of genomes with a high number of breakpoints (for a same number of steps *k*), because HT2 applies more frequently in this case.

**Table 1. Influence of the shared block property and the lower bound for distance property used as heuristic tests (HT1 and HT2 respectively) on the computation time of the distance *dist* between two mitochondrial genomes *G* and *G’* with the program Genome_Comparison.c. For each example and for each version of the program, the table gives the computation time and the number of nodes explored in the search tree (* = no result after 5 hours).**

| **Pairwise genome comparisons** | **Standard program (HT1 and HT2)** | **Program with HT2 only** | **Program with HT1 only** | **Program without HT1 and HT2** |
| --- | --- | --- | --- | --- |
| *Ophiura lukteni - Homo sapiens* *dist* = 3 (7 breakpoints) | < 1 sec 5 nd | < 1 sec 1.937 nd | < 1 sec 49 nd | 21 sec 15.667.212 nd |
| *Asterina pectinifera - Xenoturbella bocki* *dist* = 3 (8 breakpoints) | < 1 sec 19 nd | < 1 sec 3.195 nd | < 1 sec 435 nd | 24 sec 17.264.670 nd |
| *Homo sapiens - Nautilus macromphallus* *dist* = 4 (8 breakpoints) | < 1 sec 3.310 nd | < 1 sec 191.673 nd | 38 sec 88.574.474 nd | * |
| *Homo sapiens - Trichinella spiralis* *dist* = 4 (9 breakpoints) | < 1 sec 1.628 nd | < 1 sec 41.701 nd | 1 sec 1.798.253 nd | * |
| *Xenoturbella bocki - Pagurus longicarpus* *dist* = 5 (9 breakpoints) | < 1 sec 8.846 nd | 35 sec 23.718.487 nd | 1 min 6 sec 113.242.168 nd | * |
| *Homo sapiens - Bugula neritina* *dist* = 5 (11 breakpoints) | < 1 sec 118.992 nd | 3 sec 841.125 nd | * | * |
| *Homo sapiens - Cepaea nemoralis* *dist* = 6 (13 breakpoints) | 8 sec 8.955.069 nd | 37 sec 27.057.931 nd | * | * |
| *Antedon mediterranea - Ciona intestinalis* *dist* = 6 (14 breakpoints) | 4 sec 3.679.950 nd | 13 sec 9.035.144 nd | * | * |
| *Antedon mediterranea - Ciona savignyi* *dist* = 7 (14 breakpoints) | 5 sec 4.040.699 nd | 13 sec 8.964.821 nd | * | * |
| *Homo sapiens - Flustrellidra hispida* *dist* = 7 (15 breakpoints) | 8 min 9 sec 396.395.742 nd | 24 min 15 sec 981.322.106 nd | * | * |
| *Doliolum nationalis - Urechis caupo* *dist* = 8 (15 breakpoints) | 3 min 34 sec 164.672.795 nd | 8 min 52 sec 370.534.741 nd | * | * |
| *Phallusia mammillata - Trichinella spiralis* *dist* = 8 (15 breakpoints) | 32 min 9 sec 1.462.784.016 nd | 2h 24 min 32 sec 6.937.456.322 nd | * | * |

**Table 2. Influence of the lower bound for distance property used as heuristic test (HT2) on the computation time of all the paths in *k* steps between two mitochondrial genomes *G* and *G’* with the program Genome_Comparison.c.** Note that HT1 cannot be used here because the program calculates *all* the paths, and the paths are not necessarily minimum (the shared block property is not valid here). For each pairwise comparison and for each version of the program, the table gives the computation time and the number of nodes explored in the search tree (* = no result after 5 hours).

| **Pairwise genome comparisons** | **Standard program with HT2** | **Program without HT2** |
| --- | --- | --- |
| *Ophiura lukteni - Homo sapiens* *k* = 3 (7 breakpoints) (nb paths = 30) | < 1 sec 152.075 nd | 2 h 49 min 36 sec 7.133.330.050 nd |
| *Asterina pectinifera - Xenoturbella bocki* *k* = 3 (8 breakpoints) (nb paths = 9) | < 1 sec 26.950 nd | 2 h 51 min 36 sec 7.133.330.050 nd |
| *Asterina pectinifera - Xenoturbella bocki* *k* = 4 (8 breakpoints) (nb paths = 4051 , not minimum) | 27 sec 18.938.150 nd | * (only 16 paths found after 5 hours) |
| *Homo sapiens - Nautilus macromphallus* *k* = 4 (8 breakpoints) (nb paths = 976) | 14 sec 9.773.225 nd | * |
| *Homo sapiens - Trichinella spiralis* *k* = 4 (9 breakpoints) (nb paths = 26) | 2 sec 1.591.975 nd | * |
| *Homo sapiens - Trichinella spiralis* *k* = 5 (9 breakpoints) (nb paths = 129.972 , not minimum) | 35 min 17 sec 1.594.631.500 nd | * (only 1 path found after 5 hours) |
| *Xenoturbella bocki - Pagurus longicarpus* *k* = 5 (9 breakpoints) (nb paths = 183.732) | 49 min 32 sec 2.223.407.725 nd | * |
| *Homo sapiens - Bugula neritina k* = 5 (11 breakpoints) (nb paths = 2.782) | 1 min 23 sec 62.273.750 nd | * |
| *Homo sapiens - Cepaea nemoralis* *k* = 6 (13 breakpoints) (nb paths = 17.293) | 32 min 21 sec 1.439.934.650 nd | * |
| *Antedon mediterranea - Ciona intestinalis* *k* = 6 (14 breakpoints) (nb paths = 904) | 2 min 35 sec 116.435.550 nd | * |
