# Supplementary material for: A complete logical approach to resolve the evolution and dynamics of mitochondrial genome in bilaterians
Source: PLoS One. 2018 Mar 16;13(3):e0194334. doi: 10.1371/journal.pone.0194334 (PMC5856267; doi:10.1371/journal.pone.0194334)
Supplement: S3 Appendix — (DOC) [file pone.0194334.s003.doc]

**S3 appendix. List of primary phylogenetic hypotheses (PPHs).**

PPH#1 monophyly of Bilateria

PPH#2 monophyly of Deuterostomia

PPH#3 monophyly of Chordata

PPH#4 monophyly of Ambulacria

PPH#5 monophyly of Echinodermata

PPH#6 monophyly of Eleutherozoa

PPH#7 monophyly of Ophiurida

PPH#8 monophyly of Crinoidea

PPH#9 monophyly of Ecdysozoa

PPH#10 monophyly of Arthropoda

PPH#11 monophyly of Mandibulata

PPH#12 monophyly of Crustacea

PPH#13 monophyly of Decapoda

PPH#14 monophyly of Chelicerata

PPH#15 monophyly of Acari

PPH#16 monophyly of Panarthropoda

PPH#17 monophyly of Introverta

PPH#18 monophyly of Lophotrochozoa

PPH#19 monophyly of Mollusca

PPH#20 monophyly of Polyplacophora

PPH#21 monophyly of Cephalopoda

PPH#22 monophyly of Gastropoda

PPH#23 monophyly of Eutrochozoa

PPH#24 monophyly of Annelida

PPH#25 monophyly of Polychaeta

PPH#26 monophyly of Echiura

PPH#27 monophyly of Lophophorata

PPH#28 monophyly of Brachiopoda

PPH#29 monophyly of Chaetognatha

PPH#30a monophyly of Echinoidea + Asteroidea

PPH#30b monophyly of Ophiuroidea + Echinoidea (Cryptosyringid hypothesis)

PPH#30c monophyly of Asteroidea + Ophiuroidea (Asterozoa hypothesis)
