# Supplementary material for: A complete logical approach to resolve the evolution and dynamics of mitochondrial genome in bilaterians
Source: PLoS One. 2018 Mar 16;13(3):e0194334. doi: 10.1371/journal.pone.0194334 (PMC5856267; doi:10.1371/journal.pone.0194334)
Supplement: S5 Appendix — (DOC) [file pone.0194334.s005.doc]

S5 Appendix. Log book 1 - Chronological description of computations for Bilaterians.

PRELIMINARY NOTE:

The notions of "OTU" (operational taxon unit), "HTU" (hypothetical taxon unit), and "Ur-<monophyletic group>" are slightly different when they are used for dendrograms or for phylogenetic trees (cladograms).

In a phylogenetic tree, the vertices (nodes) of degree > 1 are always HTUs (ancestral states).

And "Ur-pelmatozoa" (for example) is always an HTU, because, by construction, "Ur-pelmatozoa" is always a node of degree > 1

But in a dendrogram (where each node is associated to a distinct mtDNA), nodes of degree > 1 can be represented either by HTUs or OTUs:

-> an OTU is a node associated to a fixed mtDNA (among those of the taxonomic dataset)

-> an HTU is an additional node (necessarily of degree > 1 in the most parsimonious dendrograms), with an associated mtDNA determined by calculation.

This log-book describes computations of *dendrograms* (from which one can obtain *cladograms* or *phylogenetic trees*). So, in this log-book:

-> "Ur-pelmatozoa" (for example) makes reference to the node of the dendrogram which is at the base of the pelmatozoa group. This node can be either an HTU or an OTU.

and it is correct to say:

"Ur-pelmatozoa = florometra_serratissima" (for example)

(which means:

"the node of the dendrogram which is at the base of the pelmatozoa group is the OTU florometra_serratissima"

and:

"the mtDNA associated to the base of the pelmatozoa group is equal to the mtDNA of the OTU florometra_serratissima"

)

=================

DEUTEROSTOMES

=================

----------------------------------------------------------

(1) computation of the DEUTEROSTOMIA tree with 1 outgroup:

----------------------------------------------------------

CHOICE OF THE TAXONOMIC DATASET:

All the deuterostomes (17) and the outgroup limulus_polyphemus (ecdysozoan)

homo_sapiens (Chordata)

asymmetron_inferum (Chordata)

xenoturbella_bocki (Xenoturbellidae)

balanoglossus_carnosus (Hemichordata)

strongylocentrotus_purpuratus (Echinodermata)

asterina_pectinifera (Echinodermata)

ophiura_lukteni (Echinodermata)

ophiobolis_aculeata (Echinodermata)

florometra_serratissima (Echinodermata)

gymnocrinus_richeri (Echinodermata)

antedon_mediterranea (Echinodermata)

doliolum_nationalis (Urochordata) ***

ciona_savignyi (Urochordata) ***

ciona_intestinalis (Urochordata) ***

phallusia_mammillata (Urochordata) ***

phallusia_fumigata (Urochordata) ***

halocynthia_roretzi (Urochordata) ***

limulus_polyphemus (Ecdysozoa) = OUTGROUP

but the OTUs marked with "***" have a saturation coefficient close to 100%, and must be excluded from the taxonomic dataset.

We finally keep the following list of OTUs:

LIST OF OTUs for the computation "deuterostomes_taxA":

homo_sapiens = 0 (Chordata)

asymmetron_inferum = 1 (Chordata)

limulus_polyphemus = 2 (Ecdysozoa) = OUTGROUP

xenoturbella_bocki = 3 (Xenoturbellidae)

balanoglossus_carnosus = 4 (Hemichordata)

strongylocentrotus_purpuratus = 5 (Echinodermata)

ophiura_lukteni = 6 (Echinodermata)

ophiobolis_aculeata = 7 (Echinodermata)

asterina_pectinifera = 8 (Echinodermata)

florometra_serratissima = 9 (Echinodermata)

gymnocrinus_richeri = 10 (Echinodermata)

antedon_mediterranea = 11 (Echinodermata)

PRIMARY PHYLOGENETIC HYPOTHESYS (PPH) used:

- monophyly of Deuterostomia = (0,1,3,4,5,6,7,8,9,10,11)

- monophyly of Chordata = (0,1)

- monophyly of Ambulacria = (4,5,6,7,8,9,10,11)

- monophyly of Echinodermata = (5,6,7,8,9,10,11)

- monophyly of Ophiurida = (6,7)

- monophyly of Crinoidea = (9,10,11)

SOLUTIONS:

On domain D=[0,14], we obtain:

63 possible solutions (OK for property P6) (4 impossible subtrees)

(see S6 appendix - section 'deuterostomes_taxA_63sol')

The analysis of all solutions shows the following results:

-> Ur-deuterostomia = homo_sapiens

(homo_sapiens is always at the base of the deuterostomia group, in the 63 solutions)

-> the deuterostomia tree is always equal to:

R(homo_sapiens,xenoturbella_bocki) and

R(homo_sapiens,y) and

R(y,asymmetron_inferum) and

R(homo_sapiens,balanoglossus_carnosus) and

R(homo_sapiens,z) and

z linked with the echinodermata group (63 forms)

-> there is 1 possible form for the ophiurida group (OTUs 6,7), and ophiobolis_aculeata (= 7) is at the base of the group

-> there are 3 possible forms for the crinoidea group (OTUs 9,10,11), and florometra_serratissima (= 9) is always at the base of the group

-> there are 21 possible forms for the rest of the echinodermata group (with florometra_serratissima and ophiobolis_aculeata)

Those possible forms can freely combine (the total number of solutions is multiplied consequently), so there are:

1x3x21 = 63 possibles forms for the complete echinodermata group

-> the outgroup limulus_polyphemus is always linked with homo_sapiens by a 2-edges branch: R(limulus_polyphemus,x) and R(x,homo_sapiens)

COMPUTATION with additionnal PPH Eleutherozoa:

With the same list of OTUs, and with one additionnal hypothesis: PPH Eleutherozoa (= 5,6,7,8), we obtain on domain D=[0,14]:

6 possible solutions (OK for property P6) (4 impossible subtrees)

(see S6 appendix - section 'deuterostomes_taxA_v2_6sol')

The analysis of the 6 solutions (which are a subset of the 63 solutions previously obtained) shows the same results for the base of the deuterostomia tree:

-> the outgroup limulus_polyphemus is always linked with homo_sapiens by a 2-edges branch: R(limulus_polyphemus,x) and R(x,homo_sapiens)

-> Ur-deuterostomia = homo_sapiens

-> the deuterostomia tree is always equal to:

R(homo_sapiens,xenoturbella_bocki) and

R(homo_sapiens,y) and

R(y,asymmetron_inferum) and

R(homo_sapiens,balanoglossus_carnosus) and

R(homo_sapiens,z) and

z linked with the echinodermata group

and for echinodermata:

-> there is 1 possible form for the ophiurida group (OTUs 6,7), and ophiobolis_aculeata (= 7) is at the base of the group

-> florometra_serratissima (= 9) is always at the base of the crinoidea group (OTUs 9,10,11)

but also with new results for echinodermata:

-> there are only 6 possible forms for the echinodermata group:

3 possible forms for the crinoidea group (OTUs 9,10,11) combining with

2 possible forms (among 21 previously obtained) for the rest of the tree

(one form with Ur-echinodermata = strongylocentrotus_purpuratus, one form with Ur-echinodermata = asterina_pectinifera)

Those possible forms can freely combine (the total number of solutions is multiplied consequently: 3x2 = 6 possible forms for the complete echinodermata group)

COMPUTATIONS with more PPHs:

(A) if we add the 2 additionnal PPHs:

- monophyly of Eleutherozoa (OTUs 5,6,7,8)

- monophyly of (Asteroidea+Echinoidea) group (OTUs 5,8)

we obtain only 3 solutions (see S6 appendix - section 'deuterostomes_taxA_v3_3sol').

In this computation, the 3 solutions correspond to 3 possible forms for the crinoidea group, with only one possible form for the rest of the tree (with Ur-echinodermata = asterina_pectinifera)

But a local analysis of the crinoidea group with tRNAs genes (with asterina_pectinifera as outgroup) shows that only one solution remains for the crinoidea group (see the next computations and the file "with_tRNAs_crinoids_taxA_(1sol)").

So, finally we have 1 solution for deuterostomes (model 1 in "deuterostomes_taxA_v3_(3sol).rtf").

(B) instead, if we add the 2 additionnal PPHs:

- monophyly of Eleutherozoa (OTUs 5,6,7,8)

- monophyly of Cryptosyringid (OTUs 5,6,7)

we obtain 3 solutions.

(see S6 appendix - section 'deuterostomes_taxA_v4_3sol')

The 3 solutions are identical to the 3 solutions founded in S6 appendix - section 'deuterostomes_taxA_v3_3sol', and they correspond to 3 possible forms for the crinoidea group combining with 1 possible form for the rest of the tree (with Ur-echinodermata = asterina_pectinifera).

The local analysis of the crinoidea group with tRNAs genes (with asterina_pectinifera as outgroup) has already shown that there is only one solution for the crinoidea group (see the next computations and S6 appendix - section 'with_tRNAs_crinoids_taxA_1sol').

So, finally we have 1 solution for deuterostomes satisfying the Cryptosyringid hypothesis (model 1 in S6 appendix - section 'deuterostomes_taxA_v4_3sol')

(C) instead, if we add the 2 additionnal PPHs:

- monophyly of Eleutherozoa (OTUs 5,6,7,8)

- monophyly of Asterozoa (OTUs 6,7,8)

we obtain 6 solutions (see S6 appendix - section 'deuterostomes_taxA_v5_6sol').

In this computation, the 6 solutions correspond to 3 possible forms for the crinoidea group combining with 2 possible forms for the rest of the tree (one form with Ur-echinodermata = strongylocentrotus_purpuratus, one form with Ur-echinodermata = asterina_pectinifera)

Those 6 solutions are exactly the same solutions previously obtained in S6 appendix - section 'deuterostomes_taxA_v2_6sol'

A new local analysis of the crinoidea group with tRNAs genes (with strongylocentrotus_purpuratus as outgroup) shows that only one solution remains for the crinoidea group (see the next computations and S6 appendix - section 'with_tRNAs_crinoids_taxB_1sol')

So, finally we have 2 solutions for deuterostomes satisfying the Asterozoa hypothesis:

-> one solution with Ur-echinodermata = strongylocentrotus_purpuratus

(model 1 in S6 appendix - section 'deuterostomes_taxA_v5_6sol')

-> one solution with Ur-echinodermata = asterina_pectinifera

(model 4 in S6 appendix - section 'deuterostomes_taxA_v5_6sol')

ADDITIONNAL COMPUTATIONS:

-> if no PPH are imposed, we obtain 378 solutions: the previous 63 solutions, plus 378-63 = 315 solutions which do not respect the PPHs. For example, the following model (OK for property P6) does not respect the PPH ambulacria:

R(0,3) R(0,4) R(0,n1) R(0,n2) R(1,n2) R(2,n3) R(3,0) R(4,0) R(4,n3) R(5,9) R(5,n1) R(6,7) R(7,6) R(7,n1) R(8,n1) R(9,5) R(9,10) R(9,11) R(10,9) R(11,9) R(n1,0) R(n1,5) R(n1,7) R(n1,8) R(n2,0) R(n2,1) R(n3,2) R(n3,4)

-> if we only impose the PPH Ambulacria, we obtain the 63 possible solutions (OK for P6) previously obtained, which respect *all* the PPHs. So the other PPHs (PPHs chordata, echinodermata, ophiurida and crinoidea) are logical consequence of the problem: they are always verified, even if they are not imposed. However, the PPH Deuterostomia is (implicitly) imposed, because of the choice of the taxonomic dataset and the outgroup.

-> if we fix the crinoidea group (with one of its 3 possible forms), we only obtain 63/3 = 21 possible solutions (OK for P6) (4 impossible subtrees).

(see S6 appendix - section 'deuterostomes_taxA_v6_21sol')

-----------------------------------------------------------

(2) computation of the CRINOIDEA tree with tRNAs

(and with asterina_pectinifera as outgroup)

-----------------------------------------------------------

Here, all the genomes contains 37 genes (15 coding genes + 22 tRNAs genes)

Previously, 3 possible forms were found for the crinoidea group

(see S6 appendix - sections 'deuterostomes_taxA_v3_3sol' and 'deuterostomes_taxA_v4_3sol')

This computation allow to determine the best form for crinoidea tree, when asterina_pectinifera is linked with Ur-pelmatozoa

(with PPH (Asteroidea+Echinoidea) or with PHH Cryptosyringid)

CHOICE OF THE TAXONOMIC DATASET:

All the crinoids, with the outgroup asterina_pectinifera

LIST OF OTUs for the computation "with_tRNAs_crinoids_taxA ":

asterina_pectinifera = 0 = OUTGROUP

florometra_serratissima = 1 (Crinoidea)

gymnocrinus_richeri = 2 (Crinoidea)

antedon_mediterranea = 3 (Crinoidea)

PRIMARY PHYLOGENETIC HYPOTHESYS (PPH) used:

- monophyly of Crinoidea = (1,2,3)

SOLUTIONS:

On domain D=[0,7], we obtain:

1 possible solutions (OK for property P6) (0 impossible subtree)

(see S6 appendix - section 'with_tRNAs_crinoids_taxA_1sol')

The analysis of the solution shows the following results:

-> there is 1 possible form for the crinoidea group:

R(asterina_pectinifera,x1) and R(x1,x2) and R(x2,florometra_serratissima)

R(florometra_serratissima, gymnocrinus_richeri) and

R(florometra_serratissima,x3) and R(x3,x4) and R(x4,antedon_mediterranea)

-> florometra_serratissima is at the base of the crinoidea group

(Ur-pelmatozoa = florometra_serratissima)

-----------------------------------------------------------

(3) computation of the CRINOIDEA tree with tRNAs

(and with strongylocentrotus_purpuratus as outgroup)

-----------------------------------------------------------

Here, all the genomes contains 37 genes (15 coding genes + 22 tRNAs genes)

Previously, 3 possible forms were found for the crinoidea group

(see files "deuterostomes_taxA_v5_(6sol).rtf")

This computation allow to determine the best form for crinoidea tree, when strongylocentrotus_purpuratus is linked with Ur-pelmatozoa

(one of the 2 solutions with PPH Asterozoa)

CHOICE OF THE TAXONOMIC DATASET:

All the crinoids, with the outgroup strongylocentrotus_purpuratus

LIST OF OTUs for the computation "with_tRNAs_crinoids_taxB ":

strongylocentrotus_purpuratus = 0 = OUTGROUP

florometra_serratissima = 1 (Crinoidea)

gymnocrinus_richeri = 2 (Crinoidea)

antedon_mediterranea = 3 (Crinoidea)

PRIMARY PHYLOGENETIC HYPOTHESYS (PPH) used:

- monophyly of Crinoidea = (1,2,3)

SOLUTIONS:

On domain D=[0,7], we obtain:

1 possible solutions (OK for property P6) (0 impossible subtree)

(see S6 appendix - section 'with_tRNAs_crinoids_taxB_1sol')

The analysis of the solution shows the following results:

-> there is 1 possible form for the crinoidea group:

R(strongylocentrotus_purpuratus,x1) and R(x1,x2) and R(x2,florometra_serratissima)

R(florometra_serratissima, gymnocrinus_richeri) and

R(florometra_serratissima,x3) and R(x3,x4) and R(x4,antedon_mediterranea)

-> florometra_serratissima is at the base of the crinoidea group

(Ur-pelmatozoa = florometra_serratissima)

Conclusion:

There is always only one possible form for the crinoidea group, identical in S6 appendix - section 'with_tRNAs_crinoids_taxA_1sol' and section 'with_tRNAs_crinoids_taxB_1sol'

-----------------------------------------------------------

(4) computation of the ELEUTHEROZOA tree with tRNAs

(and with florometra_serratissima as outgroup)

-----------------------------------------------------------

Here, all the genomes contains 37 genes (15 coding genes + 22 tRNAs genes)

With the Asterozoa hypothesis, 2 solutions were founded for the eleutherozoa tree (see S6 appendix - section 'deuterostomes_taxA_v5_6sol'):

-> one solution with Ur-echinodermata = strongylocentrotus_purpuratus

(model 1 in S6 appendix - section 'deuterostomes_taxA_v5_6sol')

-> one solution with Ur-echinodermata = asterina_pectinifera

(model 4 in S6 appendix - section 'deuterostomes_taxA_v5_6sol')

We make this computation to determine the best form for eleutherozoa tree (using PPH Asterozoa) using tRNA genes

Note: it was not possible to make a computation with all the eleutherozoans (with the ophiurids), because the distance matrix was impossible to calculate in a reasonnable time (with such a taxonomic dataset and with tRNAs, some minimal distance are greater than 8). That is why we have only selected asterina_pectinifera (= Ur-asterozoa) to represent all the asterozoans.

CHOICE OF THE TAXONOMIC DATASET:

strongylocentrotus_purpuratus, cucumaria_miniata, asterina_pectinifera (= Ur-asterozoa), and the outgroup florometra_serratissima

Note: if we consider only coding genes, the mtDNA of cucumaria_miniata is identical to the mtDNA of strongylocentrotus_purpuratus. But considering also tRNAs, these two mtDNA are distincts. That is why we now include cucumaria_miniata on this computation.

LIST OF OTUs for the computation "with_tRNAs_eleutherozoa_taxA ":

florometra_serratissima = 0 (Crinoidea) = OUTGROUP

strongylocentrotus_purpuratus = 1 (Eleutherozoa)

asterina_pectinifera = 2 (Eleutherozoa) (= Ur-asterozoa)

cucumaria_miniata = 3 (Eleutherozoa)

PRIMARY PHYLOGENETIC HYPOTHESYS (PPH) used:

- monophyly of Eleutherozoa = (1,2,3)

SOLUTIONS:

On domain D=[0,4], we obtain:

2 possible solutions (OK for property P6) (1 impossible subtree)

(see S6 appendix - section 'with_tRNAs_eleutherozoa_taxA_2sol')

The analysis of the solution shows the following results:

-> cucumaria_miniata is linked with strongylocentrotus_purpuratus by a 5-edge branch (5 transpositions of tRNAs genes)

-> there is 2 possible form for the eleutherozoa group, corresponding exactly to the 2 possible forms founded in S6 appendix - section 'deuterostomes_taxA_v5_6sol'

conclusion:

in the case of the Asterozoa hypothesis, a local analysis of eleutherozoa group with tRNAs is not discriminant: 2 forms remain possible for the eleutherozoa group

-----------------------------------------------------------

(5) computation of the OPHIURIDA tree with tRNAs

(and with strongylocentrotus_purpuratus as outgroup)

-----------------------------------------------------------

Here, all the genomes contains 37 genes (15 coding genes + 22 tRNAs genes)

We make this computation to compute the ophiurida tree when we also consider tRNAs genes. With the previous computations, we shall obtain the whole echinodermata tree (with tRNAs genes).

In this computation (5) and the previous ones (2,3,4), considering genomes with tRNAs genes, the *total* number of mutations is minimised by the parsimony hypothesis in the dendrograms solution.

(considering genomes with only coding genes, the number of mutations *modifying coding genes order* is minimised by the parsimony hypothesis in the dendrograms solution, to which we can add *a posteriori* some mutations proper to tRNAs genes if we want finally to consider all the genes)

Note:

Here, it has not been possible to compute ALL the solutions, because verifying property P6 was untractable for most of the solutions obtained, except one.

So this computation is NOT COMPLETE: only one solution has been founded, and probably there are other possible solutions.

CHOICE OF THE TAXONOMIC DATASET:

strongylocentrotus_purpuratus (= outgroup), asterina_pectinifera, and the ophiurids

LIST OF OTUs for the computation "with_tRNAs_ophiura_taxA ":

asterina_pectinifera = 0 (Eleutherozoa)

ophiobolis_aculeata = 1 (Ophiurida)

ophiura_lukteni = 2 (Ophiurida)

strongylocentrotus_purpuratus = 3 (Eleutherozoa) = OUTGROUP

PRIMARY PHYLOGENETIC HYPOTHESYS (PPH) used:

- monophyly of Ophiurida = (1,2)

SOLUTIONS:

On domain D=[0,13], we obtain:

1 solution (OK for property P6) (8 impossible subtrees)

(see S6 appendix - section 'with_tRNAs_ophiurida_taxA_1sol')

BUT probably there are other possible solutions (it was untractable to verify property P6 for them)

-> the solution obtained here for the ophiurida tree, with the others solutions obtained with tRNAs, allow to represent the whole echinodermata tree.

this dendrogram solution (computed for echinodermata with tRNAs) contains 25 mutations: it is the minimal number of mutations within the clade.

-> instead, the dendrogram solution previously computed for echinodermata without tRNAs contains 6 mutations (modifying coding genes), to which we must add 20 mutations proper to tRNAs genes (computed with the program genome_comparison.c). All together, we have 6 + 20 = 26 mutations within the clade (but with a minimal number of mutations modifying coding genes = 6)

We can see here that the parsimony hypothesis has a different meaning and consequently leads to different results when we consider - or not - the tRNAS genes in the genomes.

-----------------------------------------------------------

(6) computation of the DEUTEROSTOMIA tree with 2 outgroups:

-----------------------------------------------------------

CHOICE OF THE TAXONOMIC DATASET:

All the OTUs already used in "deuterostomes_taxA", with the 2 outgroups limulus_polyphemus (ecdysozoa) and katharina_tunicata (lophotrochozoa)

LIST OF OTUs for the computation "deuterostomes_taxB":

homo_sapiens = 0 (Chordata Craniata)

asymmetron_inferum = 1 (Chordata Cephalochordata)

limulus_polyphemus = 2 (Ecdysozoa) = OUTGROUP1

xenoturbella_bocki = 3 (Xenoturbellidea)

balanoglossus_carnosus = 4 (Hemichordata)

strongylocentrotus_purpuratus = 5 (Echinodermata)

ophiura_lukteni = 6 (Echinodermata Ophiurida)

ophiobolis_aculeata = 7 (Echinodermata Ophiurida)

asterina_pectinifera = 8 (Echinodermata)

florometra_serratissima = 9 (Echinodermata Crinoidea)

gymnocrinus_richeri = 10 (Echinodermata Crinoidea)

antedon_mediterranea = 11 (Echinodermata Crinoidea)

katharina_tunicata = 12 (Lophotrochozoa) = OUTGROUP2

(same OTUs as in "deuterostomes_taxA", plus katharina_tunicata)

PRIMARY PHYLOGENETIC HYPOTHESYS (PPH) used:

- monophyly of Deuterostomia = (0,1,3,4,5,6,7,8,9,10,11)

- monophyly of Chordata = (0,1)

- monophyly of Ambulacria = (4,5,6,7,8,9,10,11)

- monophyly of Echinodermata = (5,6,7,8,9,10,11)

- monophyly of Ophiurida = (6,7)

- monophyly of Crinoidea = (9,10,11)

ADDITIONAL HYPOTHESIS:

First, to divide by 3 the number of solutions obtained, we fix one of the 3 possible forms for the crinoidea group:

R(florometra_serratissima, gymnocrinus_richeri) and

R(florometra_serratissima, antedon_mediterranea) and

florometra_serratissima linked with the rest of the tree

(Ur-pelmatozoa = florometra_serratissima)

SOLUTIONS:

On domain D=[0,16], we obtain:

63 possible solutions (OK for property P6) (5 impossible subtrees)

(see S6 appendix - section 'deuterostomes_taxB_63sol')

The analysis of all solutions shows the following results:

-> Ur-deuterostomia = homo_sapiens

(homo_sapiens is always at the base of the deuterostomia group)

-> the deuterostomia tree is always equal to:

R(homo_sapiens,xenoturbella_bocki) and

R(homo_sapiens,y) and

R(y,asymmetron_inferum) and

R(homo_sapiens,balanoglossus_carnosus) and

R(homo_sapiens,z) and

z linked with the echinodermata group (21 forms, with crinoidea fixed)

-> (as in computation "deuterostomes_taxA") there is 1 possible form for the ophiurida group (OTUs 6,7), and ophiobolis_aculeata is at the base of the group:

R(ophiobolis_aculeata,ophiura_lukteni) and

ophiobolis_aculeata linked with the echinodermata group

-> (as in "deuterostomes_taxA") there are 21 possible forms for the echinodermata group with the crinoidea group fixed.

Therefore, with the additionnal PPH eleutherozoa, we would obtain only 2 possible forms for the echinodermata group with the crinoidea group fixed.

-> there are 3 possible forms for the subtree "HOLIKAT" restricted to homo_sapiens, limulus_polyphemus and katharina_tunicata:

form outgroup SOL1:

R(homo_sapiens,x1) and

R(x1,limulus_polyphemus) and

R(x1,x2) and

R(x2,katharina_tunicata)

form outgroup SOL2:

R(homo_sapiens,x1) and

R(x1,limulus_polyphemus) and

R(limulus_polyphemus,x2) and

R(x2,katharina_tunicata)

form outgroup SOL3:

R(homo_sapiens,x1) and

R(x1,x2) and

R(x2,limulus_polyphemus) and

R(x2,katharina_tunicata)

ADDITIONNAL COMPUTATIONS:

-> if we do not fix the crinoidea group, we obtain (12h of computation): 63x3 = 189 solutions (OK for P6): each of the 63 solutions previously obtained now combines with the 3 possible forms for the crinoidea group.

-> if we only impose the PPH ambulacria (and without fixing the crinoidea group), we obtain the same 189 solutions (OK for P6) previously obtained, which verify all the PPHs. So the other PPHs are *logical consequences* of the problem: they are always verified, even if they are not imposed.

-----------------------------------------------------------

(7) computation of the DEUTEROSTOMIA tree with 3 outgroups:

-----------------------------------------------------------

CHOICE OF THE TAXONOMIC DATASET:

All the OTUs already used in "deuterostomes_taxA", with the 3 outgroups limulus_polyphemus (ecdysozoa), katharina_tunicata (lophotrochozoa) and tethya_actinia (demospongiae).

LIST OF OTUs for the computation "deuterostomes_taxC":

homo_sapiens = 0 (Chordata Craniata)

asymmetron_inferum = 1 (Chordata Cephalochordata)

limulus_polyphemus = 2 (Ecdysozoa) = OUTGROUP1

xenoturbella_bocki = 3 (Xenoturbellidea)

balanoglossus_carnosus = 4 (Hemichordata)

strongylocentrotus_purpuratus = 5 (Echinodermata)

ophiura_lukteni = 6 (Echinodermata Ophiurida)

ophiobolis_aculeata = 7 (Echinodermata Ophiurida)

asterina_pectinifera = 8 (Echinodermata)

florometra_serratissima = 9 (Echinodermata Crinoidea)

gymnocrinus_richeri = 10 (Echinodermata Crinoidea)

antedon_mediterranea = 11 (Echinodermata Crinoidea)

katharina_tunicata = 12 (Lophotrochozoa) = OUTGROUP2

tethya_actinia = 13 (Demospongiae) = OUTGROUP3

(same OTUs as in "deuterostomes_taxB", plus tethya_actinia)

PRIMARY PHYLOGENETIC HYPOTHESYS (PPH) used:

- monophyly of Deuterostomia = (0,1,3,4,5,6,7,8,9,10,11)

- monophyly of Chordata = (0,1)

- monophyly of Ambulacria = (4,5,6,7,8,9,10,11)

- monophyly of Echinodermata = (5,6,7,8,9,10,11)

- monophyly of Ophiurida = (6,7)

- monophyly of Crinoidea = (9,10,11)

ADDITIONAL HYPOTHESIS:

First, to divide the number of solutions obtained, we fix one of the 63 possible forms for the echinodermata group.

A PARTICULAR CASE: THE LOSS OF GENE atp9 for tethya_actinia

To calculate the minimal distance between tethya_actinia (16 genes = 15 genes + atp9) and an other mtDNA (15 genes) of the taxonomic dataset, we only consider the 15 genes common to the 2 mtDNAs, in order to compute minimal paths between mtDNA of same size. Then we add 1 step for the loss of gene atp9.

But, to compute the tree solutions in "deuterostomes_taxC", it is simpler to code this minimal distance in the distance matrix of the model generator *without adding 1* (for the loss of genes atp9), as if we "forgot" the loss step. This is done in order to simplify the computation. Then we can insert *a posteriori* in the tree solutions the necessary loss step (at any position *between tethya_actinia and homo_sapiens*), and easily reconstruct the values of the eventual ancestral states (with 16 genes) between tethya_actinia and the loss step.

SOLUTIONS:

On domain D=[0,20], we obtain:

3 possible solutions (OK for P6) (15 impossible subtrees).

(see S6 appendix - section 'deuterostomes_taxC_3sol')

The analysis of all solutions shows the following results:

-> Ur-deuterostomia = homo_sapiens

-> the deuterostomia tree is always equal to:

R(homo_sapiens,xenoturbella_bocki) and

R(homo_sapiens,y) and

R(y,asymmetron_inferum) and

R(homo_sapiens,balanoglossus_carnosus) and

R(homo_sapiens,z) and

z linked with the echinodermata group (1 form fixed)

-> (as in computation "deuterostomes_taxB") there are 3 possible forms SOL1, SOL2, SOL3 for the subtree HOLIKAT restricted to homo_sapiens, limulus_polyphemus and katharina_tunicata:

form SOL1:

R(homo_sapiens,x1) and

R(x1,limulus_polyphemus) and

R(x1,x2) and

R(x2,katharina_tunicata)

form SOL2:

R(homo_sapiens,x1) and

R(x1,limulus_polyphemus) and

R(limulus_polyphemus,x2) and

R(x2,katharina_tunicata)

form SOL3:

R(homo_sapiens,x1) and

R(x1,x2) and

R(x2,limulus_polyphemus) and

R(x2,katharina_tunicata)

-> there is 1 possible form for the outgroup tethya_actinia (linked with homo_sapiens by a 5-edges branch: 4 + 1 loss atp9)

ADDITIONNAL COMPUTATIONS:

-> if we do not fix all the echinodermata group, but only the crinoidea group, we obtain 3x21 = 63 possible solutions (OK for P6) (5 days of computation). There are 21 possible forms for the echinodermata group with crinoidea fixed, which combines with the 3 outgroup forms (SOL1, SOL2, SOL3).

CONCLUSION (for deuterostomes):

-> there is convergence of the results in computations "deuterostomes_taxA", "deuterostomes_taxC" and "deuterostomes_taxC"

-> Ur-deuterostomia = homo_sapiens

-> Ur-ambulacria = Ur-chordata = homo_sapiens

-> the deuterostomia tree is always equal to:

R(homo_sapiens,xenoturbella_bocki) and

R(homo_sapiens,y) and

R(y,asymmetron_inferum) and

R(homo_sapiens,balanoglossus_carnosus) and

R(homo_sapiens,z) and

z linked with the echinodermata group

for echinodermata:

-> 1 possible form for the ophiurida group

(and Ur-ophiurida = ophiobolis_aculeata)

-> 3 possible forms for the crinoidea group

(and Ur-pelmatozoa = florometra_serratissima)

-> 21 possible forms for the rest of the echinodermata group

(with ophiobolis_aculeata and florometra_serratissima)

Those possible forms can freely combine (the total number of solutions is multiplied consequently), so there are:

1x3x21 = 63 possibles forms for the complete echinodermata group

-> but only 1 form (among 3) is possible for the crinoidea group after making a local analysis of the crinoidea with the tRNAs genes

-> with additional primary phylogenetic hypothesis:

monophyly of Eleutherozoa

only 2 forms are possible for the echinodermata group (and thus for the deuterostomia group)

-> with the additional primary phylogenetic hypothesis:

monophyly of Eleutherozoa

monophyly of (Asteroidea+Echinoidea)

only 1 form (among 63) is possible for the deuterostomia group

-> with the additional primary phylogenetic hypothesis:

monophyly of Eleutherozoa

monophyly of Cryptosyringid

only 1 form (among 63) is possible for the deuterostomia group

-> with the additional primary phylogenetic hypothesis:

monophyly of Eleutherozoa

monophyly of Asterozoa

only 2 form (among 63) are possible for the deuterostomia group

outgroup:

-> 1 possible form for the outgroup tethya_actinia (linked with homo_sapiens by a 5-edges branch: 4 + 1 loss atp9)

-> 3 possible forms SOL1, SOL2, and SOL3 for the subtree HOLIKAT restricted to homo_sapiens, limulus_polyphemus and katharina_tunicata

-> necessaries PPHs to obtain the results:

* PPH Ambulacria

* PPH Eleutherozoa

plus: (PPHs implicitly used)

* PPH Deuterostomia

=================

ECDYSOZOANS

=================

There are too many ecdysozoans (20) to make one global computation. But we can make several smaller computations, verifying at each step the convergence of results.

-----------------------------------------------------------------------

(1) PRELIMINARY computation of the MANDIBULATA tree with 2 outgroups:

-----------------------------------------------------------------------

CHOICE OF THE TAXONOMIC DATASET:

All the arthropods mandibulates (11)

+ limulus_polyphemus (chelicerata, but with a mtDNA equal to the mtDNA of several mandibulates: for example fenneropenaeus_chinensis: mandibulata crustacea)

+ the outgroups nymphon_gracile (chelicerata) and homo_sapiens (Ur-deuterostomia)

narceus_annularis (Mandibulata Myriapoda)

speleonectes_tulumensis (Mandibulata Crustacea Remipedia)

argulus_americanus (Mandibulata Crustacea Branchiura)

megabalanus_volcano (Mandibulata Crustacea Cirripedia)

eriocheir_sinensis (Mandibulata Crustacea Decapoda)

chinkia_crosnieri (Mandibulata Crustacea Decapoda)

pagurus_longicarpus (Mandibulata Crustacea Decapoda)

cherax_destructor (Mandibulata Crustacea Decapoda)

vargula_hilgendorfi (Mandibulata Crustacea Ostracoda)

ligia_oceanica (Mandibulata Crustacea Isopoda)

tigriopus_japonicus (Mandibulata Crustacea Copepoda) ***

limulus_polyphemus; (Chelicerata)

nymphon_gracile (Cheliceratea Pycnogonida) = OUTGROUP1

homo_sapiens (Ur-deuterostomia) = OUTGROUP2

but the OTU marked with "***" has a saturation coefficient equal to 100%, and must be excluded from the taxonomic dataset.

First, we also exclude from the taxonomic dataset the 2 OTUs ligia_oceanica (crustacea isopoda) and cherax_destructor (crustacea decapoda) (rapidly evolving taxa). So we can make a preliminary computation - in a reasonnable time - of the base of the mandibulata tree.

LIST OF OTUs for the computation "ecdysozoans_taxA":

narceus_annularis=0; (Mandibulata Myriapoda)

speleonectes_tulumensis=1; (Mandibulata Crustacea Remipedia)

argulus_americanus=2; (Mandibulata Crustacea Branchiura)

megabalanus_volcano=3; (Mandibulata Crustacea Cirripedia)

eriocheir_sinensis=4; (Mandibulata Crustacea Decapoda)

chinkia_crosnieri=5; (Mandibulata Crustacea Decapoda)

pagurus_longicarpus=6; (Mandibulata Crustacea Decapoda)

vargula_hilgendorfi=7; (Mandibulata Crustacea Ostracoda)

limulus_polyphemus=8; (Chelicerata)

nymphon_gracile=9; (Chelicerata Pycnogonidea) = OUTGROUP1

homo_sapiens=10; (Ur-deuterostomia) = OUTGROUP2

PRIMARY PHYLOGENETIC HYPOTHESYS (PPH) used:

- monophyly of Arthropoda = (0,1,2,3,4,5,6,7,8,9)

- monophyly of Mandibulata = (0,1,2,3,4,5,6,7)

- monophyly of Crustacea = (1,2,3,4,5,6,7)

- monophyly of Decapoda = (4,5,6)

- monophyly of Chelicerata = (8,9)

SOLUTIONS:

On domain D=[0,14], we obtain:

9 possible solutions (OK for P6) (4 impossible subtrees)

(see S7 appendix - section 'ecdysozoans_taxA_9sol')

The analysis of all solutions shows the following results:

-> Ur-mandibulata = limulus_polyphemus

-> there is always (in the 9 solutions):

R(limulus_polyphemus,narceus_annularis) and

R(limulus_polyphemus,nymphon_gracile)

-> there is 1 possible form for the crustacea decapoda group (not yet complete: OTUs 4,5,6):

R(limulus_polyphemus,eriocheir_sinensis) and

R(limulus_polyphemus,y) and

R(y,chinkia_crosnieri) and

R(y,pagurus_longicarpus)

-> there are 9 possible forms for the rest of the crustacea group (OTUs 1,2,3,7)

-> the best solution is model 8 (score=18)

-----------------------------------------------------------------------

(2) PRELIMINARY computation of the MANDIBULATA tree with 3 outgroups:

-----------------------------------------------------------------------

CHOICE OF THE TAXONOMIC DATASET:

All the OTUs already used in "ecdysozoans_taxA", with the 3 outgroups nymphon_gracile (chelicerata), homo_sapiens (Ur-deuterostomia), and katharina_tunicata (lophotrochozoa)

LIST OF OTUs for the computation " ecdysozoans_taxB":

narceus_annularis=0; (Mandibulata Myriapoda)

speleonectes_tulumensis=1; (Mandibulata Crustacea Remipedia)

argulus_americanus=2; (Mandibulata Crustacea Branchiura)

megabalanus_volcano=3; (Mandibulata Crustacea Cirripedia)

eriocheir_sinensis=4; (Mandibulata Crustacea Decapoda)

chinkia_crosnieri=5; (Mandibulata Crustacea Decapoda)

pagurus_longicarpus=6; (Mandibulata Crustacea Decapoda)

vargula_hilgendorfi=7; (Mandibulata Crustacea Ostracoda)

limulus_polyphemus=8; (Chelicerata)

nymphon_gracile=9; (Chelicerata Pycnogonida) = OUTGROUP1

homo_sapiens=10; (Ur-deuterostomia) = OUTGROUP2

katharina_tunicata = 11 (Lophotrochozoa) = OUTGROUP3

(same OTUs as in "ecdysozoans_taxA", plus katharina_tunicata)

PRIMARY PHYLOGENETIC HYPOTHESYS (PPH) used:

- monophyly of Arthropoda = (0,1,2,3,4,5,6,7,8,9)

- monophyly of Mandibulata = (0,1,2,3,4,5,6,7)

- monophyly of Crustacea = (1,2,3,4,5,6,7)

- monophyly of Decapoda = (4,5,6)

- monophyly of Chelicerata = (8,9)

SOLUTIONS:

On domain D=[0,16], we obtain:

27 possible solutions (OK for P6) (4 impossible subtrees)

(see S7 appendix - section 'ecdysozoans_taxB_27sol')

The analysis of all solutions shows the following results:

-> Ur-mandibulata = limulus_polyphemus

-> there is always (in the 27 solutions):

R(limulus_polyphemus,narceus_annularis) and

R(limulus_polyphemus,nymphon_gracile)

-> (as in computation "ecdysozoans_taxA") there is 1 possible form for the crustacea decapoda group (uncomplete: OTUs 4,5,6):

R(limulus_polyphemus,eriocheir_sinensis) and

R(limulus_polyphemus,y) and

R(y,chinkia_crosnieri) and

R(y,pagurus_longicarpus)

-> (as in "ecdysozoans_taxA") there are 9 possible forms for the rest of the crustacea group (OTUs 1,2,3,7)

-> the best solution is model 19 (score=23)

-> there are 3 possible forms SOL1, SOL2, and SOL3 for the subtree HOLIKAT restricted to homo_sapiens, limulus_polyphemus and katharina_tunicata (the same forms as in "deuterostomes_taxB" and "deuterostomes_taxC"):

form SOL1:

R(homo_sapiens,x1) and

R(x1,limulus_polyphemus) and

R(x1,x2) and

R(x2,katharina_tunicata)

form SOL2:

R(homo_sapiens,x1) and

R(x1,limulus_polyphemus) and

R(limulus_polyphemus,x2) and

R(x2,katharina_tunicata)

form SOL3:

R(homo_sapiens,x1) and

R(x1,x2) and

R(x2,limulus_polyphemus) and

R(x2,katharina_tunicata)

------------------------------------------------------------------------

(3) computation of the MANDIBULATA tree (except crustacea decapoda):

------------------------------------------------------------------------

CHOICE OF THE TAXONOMIC DATASET:

All the OTUs already used in "ecdysozoans_taxB", plus ligia_oceanica (mandibulata crustacea isopoda)

LIST OF OTUs for the computation " ecdysozoans_taxC":

narceus_annularis=0; (Mandibulata Myriapoda)

speleonectes_tulumensis=1; (Mandibulata Crustacea Remipedia)

argulus_americanus=2; (Mandibulata Crustacea Branchiura)

megabalanus_volcano=3; (Mandibulata Crustacea Cirripedia)

eriocheir_sinensis=4; (Mandibulata Crustacea Decapoda)

chinkia_crosnieri=5; (Mandibulata Crustacea Decapoda)

pagurus_longicarpus=6; (Mandibulata Crustacea Decapoda)

vargula_hilgendorfi=7; (Mandibulata Crustacea Ostracoda)

limulus_polyphemus=8; (Chelicerata)

nymphon_gracile=9; (Chelicerata Pycnogonida) = OUTGROUP1

homo_sapiens=10; (Ur-deuterostomia) = OUTGROUP2

katharina_tunicata = 11 (Lophotrochozoa) = OUTGROUP3

ligia_oceanica=12; (Mandibulata Crustacea Isopoda)

PRIMARY PHYLOGENETIC HYPOTHESYS (PPH) used:

- monophyly of Arthropoda = (0,1,2,3,4,5,6,7,8,9,12)

- monophyly of Mandibulata = (0,1,2,3,4,5,6,7,12)

- monophyly of Crustacea = (1,2,3,4,5,6,7,12)

- monophyly of Decapoda = (4,5,6)

- monophyly of Chelicerata = (8,9)

ADDITIONNAL HYPOTHESIS:

First, to divide (by 3) the number of solutions obtained, we arbitrairly fix the form SOL3 for the subtree HOLIKAT restricted to homo_sapiens, limulus_polyphemus and katharina_tunicata.

We also fix the unique possible form previously obtained for the crustacea decapoda group (uncomplete: OTUs 4,5,6):

R(limulus_polyphemus,eriocheir_sinensis) and

R(limulus_polyphemus,G1) and

R(G1,chinkia_crosnieri) and

R(G1,pagurus_longicarpus)

(all the possible forms for the - complete - crustacea decapoda group will be studied in the next computation "ecdysozoans_taxD")

SOLUTIONS:

On domain D=[0,19], we obtain:

39 possible solutions (OK for P6) (15 impossible subtrees)

(see S7 appendix - section 'ecdysozoans_taxC_39sol')

The analysis of all solutions shows the following results:

-> Ur-mandibulata = limulus_polyphemus

-> there is always (in the 39 solutions):

R(limulus_polyphemus,narceus_annularis) and

R(limulus_polyphemus,nymphon_gracile)

-> there are 39 possible forms for the mandibulata group (OTUs 1,2,3,7,12) (with the decapoda group fixed)

-> the best solution is model 31 (score=147)

ADDITIONNAL COMPUTATIONS:

-> if no PPH are imposed, we obtain 614 solutions on domain D=[0,19]. For example, the model (OK for P6):

R(0,3) R(0,8) R(0,n1) R(1,8) R(1,n2) R(1,n3) R(2,n1) R(3,0) R(4,8) R(5,13) R(6,13) R(7,n2) R(8,0) R(8,1) R(8,4) R(8,9) R(8,13) R(8,15) R(9,8) R(10,14) R(11,15) R(12,n4) R(13,5) R(13,6) R(13,8) R(14,10) R(14,15) R(15,8) R(15,11) R(15,14) R(n1,0) R(n1,2) R(n2,1) R(n2,7) R(n3,1) R(n3,n4) R(n4,12) R(n4,n3)

which do not respect the PPH crustacea,

or the model (OK for P6):

R(0,15) R(1,n1) R(1,n2) R(2,n4) R(3,8) R(3,n4) R(4,8) R(5,13) R(6,13) R(7,n2) R(8,3) R(8,4) R(8,9) R(8,13) R(8,15) R(8,n2) R(9,8) R(10,14) R(11,15) R(12,n3) R(13,5) R(13,6) R(13,8) R(14,10) R(14,15) R(15,0) R(15,8) R(15,11) R(15,14) R(n1,1) R(n1,n3) R(n2,1) R(n2,7) R(n2,8) R(n3,12) R(n3,n1) R(n4,2) R(n4,3)

which do not respect the PPH mandibulata.

-> if we only impose the 2 PPHs Crustacea and Mandibulata, we obtain the same 39 possible solutions (OK for P6) previously obtained, which respect *all* the PPHs. The other PPHs are logical consequence of the problem. However, the PPH Decapoda is (implicitly) imposed, because we fix the decapoda group.

-> if we do not fix the form SOL3 for the HOLIKAT subtree, we obtain (2 days of computation) 117 possible solutions (OK for P6): each of the 39 solutions previously obtained now combines with the 3 possible forms SOL1, SOL2, SOL3 for the subtree HOLIKAT (3x39 = 117).

------------------------------------------------

(4) computation of the CRUSTACEA DECAPODA tree:

------------------------------------------------

CHOICE OF THE TAXONOMIC DATASET:

All the OTUs already used in "ecdysozoans_taxC", plus cherax_destructor (mandibulata crustacea decapoda)

LIST OF OTUs for the computation "ecdysozoans_taxD":

narceus_annularis=0; (Mandibulata Myriapoda)

speleonectes_tulumensis=1; (Mandibulata Crustacea Remipedia)

argulus_americanus=2; (Mandibulata Crustacea Branchiura)

megabalanus_volcano=3; (Mandibulata Crustacea Cirripedia)

eriocheir_sinensis=4; (Mandibulata Crustacea Decapoda)

chinkia_crosnieri=5; (Mandibulata Crustacea Decapoda)

pagurus_longicarpus=6; (Mandibulata Crustacea Decapoda)

vargula_hilgendorfi=7; (Mandibulata Crustacea Ostracoda)

limulus_polyphemus=8; (Chelicerata)

nymphon_gracile=9; (Chelicerata Pycnogonida) = OUTGROUP1

homo_sapiens=10; (Ur-deuterostomia) = OUTGROUP2

katharina_tunicata = 11 (Lophotrochozoa) = OUTGROUP3

ligia_oceanica=12; (Mandibulata Crustacea Isopoda)

cherax_destructor=13; (Mandibulata Crustacea Decapoda)

PRIMARY PHYLOGENETIC HYPOTHESYS (PPH) used:

- monophyly of Arthropoda = (0,1,2,3,4,5,6,7,8,9,12,13)

- monophyly of Mandibulata = (0,1,2,3,4,5,6,7,12,13)

- monophyly of Crustacea = (1,2,3,4,5,6,7,12,13)

- monophyly of Decapoda = (4,5,6,13)

- monophyly of Chelicerata = (8,9)

ADDITIONNAL HYPOTHESIS:

We fix the best possible form previously obtained for the crustacea group (except decapoda) (OTUs 1,2,3,7,12):

R(limulus_polyphemus,speleonectes_tulumensis) and

R(limulus_polyphemus,megabalanus_volcano) and

R(limulus_polyphemus,G1) and R(G1,argulus_americanus) and

R(limulus_polyphemus,G2) and R(G2,vargula_hilgendorfi) and

R(limulus_polyphemus,G3) and R(G3,G4) and R(G4,ligia_oceanica)

SOLUTIONS:

On domain D=[0,22], we obtain:

9 possible solutions (OK for P6) (36 impossible subtrees)

(see S7 appendix - section 'ecdysozoans_taxD_9sol')

The analysis of all solutions shows the following results:

-> Ur-mandibulata = limulus_polyphemus

-> there is always (in the 9 solutions):

R(limulus_polyphemus,narceus_annularis) and

R(limulus_polyphemus,nymphon_gracile) and

R(limulus_polyphemus,eriocheir_sinensis)

-> there are 3 possible forms for the decapoda group (OTUs 4,5,6,13)

-> there are the 3 possible forms SOL1, SOL2, SOL3, for the subtree HOLIKAT restricted to homo_sapiens, limulus_polyphemus and katharina_tunicata (as in "deuterostomes_taxB" and "deuterostomes_taxC")

-> the best solution is model 7 (score=204)

ADDITIONNAL COMPUTATIONS:

-> if no PPH are imposed, we obtain 1.096 solutions on domain D=[0,22]. For example, the model (OK for P6):

R(0,8) R(0,n1) R(0,n2) R(1,8) R(2,14) R(3,8) R(4,8) R(5,n3) R(6,n3) R(7,15) R(8,0) R(8,1) R(8,3) R(8,4) R(8,9) R(8,14) R(8,15) R(8,16) R(8,n3) R(9,8) R(10,n1) R(11,n2) R(12,17) R(13,n4) R(14,2) R(14,8) R(15,7) R(15,8) R(16,8) R(16,17) R(17,12) R(17,16) R(n1,0) R(n1,10) R(n2,0) R(n2,11) R(n3,5) R(n3,6) R(n3,8) R(n3,n5) R(n4,13) R(n4,n5) R(n5,n3) R(n5,n4)

which do not respect the PPH Mandibulata

or the model (OK for P6):

R(0,8) R(1,8) R(2,14) R(3,8) R(4,8) R(5,n1) R(6,n1) R(7,15) R(8,0) R(8,1) R(8,3) R(8,4) R(8,9) R(8,14) R(8,15) R(8,16) R(8,n1) R(9,8) R(9,n4) R(10,n4) R(11,n5) R(12,17) R(13,n3) R(14,2) R(14,8) R(15,7) R(15,8) R(16,8) R(16,17) R(17,12) R(17,16) R(n1,5) R(n1,6) R(n1,8) R(n1,n2) R(n2,n1) R(n2,n3) R(n3,13) R(n3,n2) R(n4,9) R(n4,10) R(n4,n5) R(n5,11) R(n5,n4)

which do not respect the PPH Chelicerata.

-> if we only impose the 2 PPHs Mandibulata and Chelicerata, we obtain the same 9 possible solutions (OK for P6) previously obtained, which respect *all* the PPHs. The other PPHs are logical consequence of the problem.

----------------------------------------

(5) computation of the CHELICERATA tree:

----------------------------------------

CHOICE OF THE TAXONOMIC DATASET:

All the arthropods chelicerates (5), plus the 3 outgroups narceus_annularis (mandibulata), homo_sapiens (Ur-deuterostomia), and katharina_tunicata (lophotrochozoa)

LIST OF OTUs for the computation " ecdysozoans_taxE":

limulus_polyphemus=0; (Arthropoda Chelicerata)

nymphon_gracile=1: (Arthropoda Chelicerata Pycnogonida)

dermatophagoides_pteronyssinus=2; (Arthropoda Chelicerata Acari)

steganacarus_magnus=3; (Arthropoda Chelicerata Acari)

leptotrombidium_akamushi=4; (Arthropoda Chelicerata Acari)

narceus_annularis=5; (Arthropoda Mandibulata) = OUTGROUP1

homo_sapiens=6; (Ur-deuterostomia) = OUTGROUP2

katharina_tunicata=7; (Lophotrochozoa) = OUTGROUP3

PRIMARY PHYLOGENETIC HYPOTHESYS (PPH) used:

- monophyly of Ecdysozoa = (0,1,2,3,4,5)

- monophyly of Arthropoda = (0,1,2,3,4,5)

- monophyly of Chelicerata = (0,1,2,3,4)

- monophyly of Acari = (2,3,4)

ADDITIONNAL HYPOTHESIS:

First, to divide (by 3) the number of solutions obtained, we arbitrairly fix the form SOL2 for the subtree HOLIKAT restricted to homo_sapiens, limulus_polyphemus and katharina_tunicata.

SOLUTIONS:

On domain D=[0,15], we obtain:

9 possible solutions (OK for P6) (17 impossible subtrees)

(see S7 appendix - section 'ecdysozoans_taxE_9sol')

The analysis of all solutions shows the following results:

-> Ur-chelicerata = limulus_polyphemus

-> there is always (in the 9 solutions):

R(limulus_polyphemus,narceus_annularis)

-> there are 9 possible forms for the chelicerata group (OTUs 1,2,3,4)

(containing 6 distinct possible forms for the acari group: OTUs 2,3,4)

-> the best solutions are model 4 and 6 (score=28)

ADDITIONNAL COMPUTATIONS:

-> if we do not fix the form SOL2 for the HOLIKAT subtree, we obtain 27 solutions (OK for P6) on domain D=[0,15]:

Each of the 9 solutions previously obtained now combines with the 3 possible forms SOL1, SOL2, SOL3 for the HOLIKAT subtree (3x9 = 27).

The best solutions are models 11 and 13 (score=28), which correspond to model 4 and 6 previously obtained with SOL2 fixed.

(see S7 appendix - section 'ecdysozoans_taxF_27sol')

-> if no PPH are imposed, we obtain 1.141 solutions on domain D=[0,15]. For example, the model (OK for P6):

R(0,5) R(0,n5) R(1,n5) R(1,n7) R(2,n3) R(3,n4) R(4,n8) R(5,0) R(6,n7) R(7,n6) R(n1,n2) R(n1,n8) R(n2,n1) R(n2,n4) R(n3,2) R(n3,n4) R(n3,n5) R(n4,3) R(n4,n2) R(n4,n3) R(n5,0) R(n5,1) R(n5,n3) R(n6,7) R(n6,n7) R(n7,1) R(n7,6) R(n7,n6) R(n8,4) R(n8,n1)

which do not respect the PPH Chelicerata

-> if we only impose the PPH Chelicerata, we obtain the 27 possible solutions (OK for P6) previously obtained, which respect *all* the PPHs. The other PPHs are logical consequence of the problem.

--------------------------------------------------------------

(6) computation of the PANARTHROPODA and INTROVERTA tree:

--------------------------------------------------------------

CHOICE OF THE TAXONOMIC DATASET:

All the OTUs already used in "ecdysozoans_taxE", plus:

epiperipatus_biolleyi (Panarthropoda Onychophora)

priapulus_caudatus (Introverta Priapulida)

trichinella_spiralis (Introverta Nematoda)

caenorhabditis_elegans (Introverta Nematoda) ***

but the OTU marked with "***" has a saturation coefficient close to 100%, and must be excluded from the taxonomic dataset.

LIST OF OTUs for the computation " ecdysozoans_taxG":

limulus_polyphemus=0; (Arthropoda Chelicerata)

nymphon_gracile=1: (Arthropoda Chelicerata Pycnogonida)

dermatophagoides_pteronyssinus=2; (Arthropoda Chelicerata Acari)

steganacarus_magnus=3; (Arthropoda Chelicerata Acari)

leptotrombidium_akamushi=4; (Arthropoda Chelicerata Acari)

narceus_annularis=5; (Arthropoda Mandibulata) = OUTGROUP1

homo_sapiens=6; (Ur-deuterostomia) = OUTGROUP2

katharina_tunicata=7; (Lophotrochozoa) = OUTGROUP3

epiperipatus_biolleyi=8; (Panarthropoda Onychophora)

priapulus_caudatus=9; (Introverta Priapulida)

trichinella_spiralis=10; (Introverta Nematoda)

PRIMARY PHYLOGENETIC HYPOTHESYS (PPH) used:

- monophyly of Ecdysozoa = (0,1,2,3,4,5,8,9,10)

- monophyly of Arthropoda = (0,1,2,3,4,5)

- monophyly of Chelicerata = (0,1,2,3,4)

- monophyly of Acari = (2,3,4)

- monophyly of Panarthropoda = (0,1,2,3,4,5,8)

- monophyly of Introverta = (9,10)

ADDITIONNAL HYPOTHESIS:

We fix the best possible form previously obtained for the arthropoda group (OTUs 0,1,2,3,4,5):

R(limulus_polyphemus,narceus_annularis) and

R(limulus_polyphemus,nymphon_gracile) and

R(limulus_polyphemus,G1) and R(G1,steganacarus_magnus) and

R(G1,G2) and R(G2,G3) and R(G3,dermatophagoides_pteronyssinus) and

R(G2,G4) and R(G4,G5) and R(G5,G6) and R(G6,leptotrombidium_akamushi)

SOLUTIONS:

On domain D=[0,22], we obtain:

7 possible solutions (OK for P6) (36 impossible subtrees)

(see S7 appendix - section 'ecdysozoans_taxG_7sol')

The analysis of all solutions shows the following results:

-> Ur-panarthropoda = limulus_polyphemus

-> there is 1 possible form for epiperipatus_biolleyi (onychophora) (OTU 8):

R(limulus_polyphemus,x1) and R(x1,x2) and R(x2, epiperipatus_biolleyi)

so there are 1053 possible forms for the complete panarthropoda group:

3 possible forms for the crustacea decapoda group

39 possible forms for the rest of the mandibulata group

9 possible forms for the chelicerata group

1 possible form for epiperipatus_biolleyi (onychophora)

(3x39x9x1 = 1053)

-> the best solutions is model 1 (score=380)

-> there are 3 possible forms SOL1, SOL2, SOL3, for the subtree HOLIKAT restricted to homo_sapiens, limulus_polyphemus and katharina_tunicata (as in computations "ecdysozoans_taxD" and "ecdysozoans_taxF")

*plus* 2 new possible forms:

* form SOL1_ALTER:

R(homo_sapiens,x1) and

R(x1,priapulus_caudatus) and

R(x1,x2) and

R(x2,katharina_tunicata) and

R(priapulus_caudatus,limulus_polyphemus)

(Ur-ecdysozoa = priapulus_caudatus).

the form SOL1_ALTER is equivalent to SOL1 in which we substitute limulus_polyphemus by priapulus_caudatus

* form SOL2_ALTER:

R(homo_sapiens,x1) and

R(x1,priapulus_caudatus) and

R(priapulus_caudatus,x2) and

R(x2,katharina_tunicata) and

R(priapulus_caudatus,limulus_polyphemus)

(Ur-ecdysozoa = priapulus_caudatus).

the form SOL2_ALTER is equivalent to SOL2 in which we substitute limulus_polyphemus by priapulus_caudatus

-> there are 4 possible forms for the introverta group (OTUs 9,10):

1 possible form for SOL1, SOL2, SOL3 (models 1,2,3):

R(limulus_polyphemus,priapulus_caudatus) and

R(priapulus_caudatus,x1) and R(x1,x2) and R(x2,trichinella_spiralis)

*PLUS* 2 other possible forms (*only for SOL1*) (models 4,5)

*PLUS* 1 other possible form (for SOL1_ALTER and SOL2_ALTER) (model 6,7)

-> there are 4x1053 = 4212 possible forms for the ecdysozoa group:

1053 possible forms for the panarthropoda group

4 possible forms for the introverta group

ADDITIONNAL COMPUTATIONS:

-> if no PPH are imposed, we obtain 185 solutions on domain D=[0,22]. For example, the model (OK for P6):

R(0,1) R(0,5) R(0,9) R(0,11) R(0,n1) R(0,n2) R(1,0) R(2,13) R(3,11) R(4,16) R(5,0) R(6,n1) R(7,n3) R(8,n4) R(9,0) R(10,n5) R(11,0) R(11,3) R(11,12) R(12,11) R(12,13) R(12,14) R(13,2) R(13,12) R(14,12) R(14,15) R(15,14) R(15,16) R(16,4) R(16,15) R(n1,0) R(n1,6) R(n1,n3) R(n1,n6) R(n2,0) R(n2,n4) R(n3,7) R(n3,n1) R(n4,8) R(n4,n2) R(n5,10) R(n5,n6) R(n6,n1) R(n6,n5)

which do not respect the PPH Introverta

or the model (OK for P6):

R(0,1) R(0,5) R(0,11) R(0,n1) R(0,n2) R(0,n3) R(1,0) R(2,13) R(3,11) R(4,16) R(5,0) R(6,n1) R(7,n2) R(8,n6) R(9,n1) R(10,n5) R(11,0) R(11,3) R(11,12) R(12,11) R(12,13) R(12,14) R(13,2) R(13,12) R(14,12) R(14,15) R(15,14) R(15,16) R(16,4) R(16,15) R(n1,0) R(n1,6) R(n1,9) R(n1,n4) R(n2,0) R(n2,7) R(n3,0) R(n3,n6) R(n4,n1) R(n4,n5) R(n5,10) R(n5,n4) R(n6,8) R(n6,n3)

which do not respect the PPH Ecdysozoa

-> if we only impose the 2 PPHs Ecdysozoa and Introverta, we obtain the same 7 possible solutions (OK for P6) previously obtained, which respect *all* the PPHs. The other PPHs are logical consequence of the problem.

CONCLUSION (for the ecdysozoans):

-> Ur-panarthropoda = limulus_polyphemus

-> there are 2 possible values for Ur-introverta:

Ur-introverta = priapulus_caudatus

(see models 1,2,3,4,6,7, in section 'ecdysozoans_taxG_7sol')

Ur-introverta = UR1 =

[cox1 cox2 atp8 atp6 cox3 nad3 -nad5 -nad4 -nad4L nad6 cob rrnS rrnL nad1 nad2]

(see model 5, in section 'ecdysozoans_taxG_7sol')

-> there are 3 possible values for Ur-ecdysozoa:

Ur-ecdysozoa = limulus_polyphemus

(see models 1,2,3, in section 'ecdysozoans_taxG_7sol')

Ur-ecdysozoa = priapulus_caudatus

(see models 6,7, in section 'ecdysozoans_taxG_7sol')

Ur-ecdysozoa = UR1 =

[cox1 cox2 atp8 atp6 cox3 nad3 -nad5 -nad4 -nad4L nad6 cob rrnS rrnL nad1 nad2]

(see models 4,5, in section 'ecdysozoans_taxG_7sol')

-> there is always:

R(limulus_polyphemus,eriocheir_sinensis) and

R(limulus_polyphemus,narceus_annularis) and

R(limulus_polyphemus,x1) and R(x1,x2) and R(x2, epiperipatus_biolleyi)

-> there are:

3 possible forms for the crustacea decapoda group

39 possible forms for the rest of the mandibulata group

(3x39 = 117 possible forms for the mandibulata group)

9 possible forms for the chelicerata group

(containing 6 distinct forms for the acari group)

(117x9 = 1053 possible forms for the arthropoda group)

1 possible form for the onychophora group (epiperipatus_biolleyi)

(1x1053 = 1053 possible forms for the panarthropoda group)

4 possible forms for the introverta group

(4x1053= 4212 possible forms for the ecdysozoa group)

-> there are:

3 possible forms SOL1, SOL2, SOL3, for the subtree HOLIKAT restricted to homo_sapiens, limulus_polyphemus and katharina_tunicata

*PLUS* 2 new forms:

* form SOL1_ALTER:

R(homo_sapiens,x1) and

R(x1,priapulus_caudatus) and

R(x1,x2) and

R(x2,katharina_tunicata) and

R(priapulus_caudatus,limulus_polyphemus)

* form SOL2_ALTER:

R(homo_sapiens,x1) and

R(x1,priapulus_caudatus) and

R(priapulus_caudatus,x2) and

R(x2,katharina_tunicata) and

R(priapulus_caudatus,limulus_polyphemus)

-> necessaries PPHs to obtain the results:

* PPH Crustacea

* PPH Mandibulata

* PPH Chelicerata

* PPH Ecdysozoa

* PPH Introverta

plus: (PPHs implicitly used)

* PPH Decapoda

* PPH Arthropoda

NOTE for the next computations:

All the possible configurations at the base of the ecdysozoa tree (with the 3 possible values for Ur-ecdysozoa) can be described using the 2 OTUS: limulus_polyphemus and priapulus_caudatus

Therefore, 2 series of computations are now necessary for studying all the other groups (lophotrochozoa, deuterostomia, chaetognatha):

-> with the outgroup limulus_polyphemus

(which appears in the outgroup forms SOL1, SOL2, SOL3)

-> with the outgroup priapulus_caudatus

(which appears in the outgroup forms SOL1_ALTER, SOL2_ALTER)

=====================

LOPHOTROCHOZOANS

=====================

There are too many lophotrochozoans (27) to make one global computation. But we can make several smaller computations, verifying at each step the convergence of results.

=======================================================

LOPHOTROCHOZOANS - PART A (with limulus_polyphemus)

=======================================================

Using the outgroup limulus_polyphemus (ecdysozoa)

---------------------------------------

(1) computation of the MOLLUSCA tree:

---------------------------------------

CHOICE OF THE TAXONOMIC DATASET:

All the molluscs (12), plus the 2 outgroups homo_sapiens (Ur-deuterostomia) and limulus_polyphemus (Ecdysozoa):

katharina_tunicata (Mollusca Polyplacophorea)

nautilus_macromphallus (Mollusca Cephalopoda)

loligo_bleekeri (Mollusca Cephalopoda)

albinaria_soerulea (Mollusca Gastropoda)

cepaea_nemoralis (Mollusca Gastropoda)

biomphalaria_glabrata (Mollusca Gastropoda)

siphonodentalium_lobatum (Mollusca Scaphopoda) ***

graptacme_eborea (Mollusca Scaphopoda) ***

mytilus_edulis (Mollusca Bivalvia) ***

lampsilis_ornata (Mollusca Bivalvia) ***

venerupis_philippinarum (Mollusca Bivalvia) ***

inversidens_japanensis (Mollusca Bivalvia) ***

limulus_polyphemus (Ecdysozoa) = OUTGROUP1

homo_sapiens (Ur-deuterostomia) = OUTGROUP2

but the OTUs marked with "***" have a saturation coefficient equal to 100%, and must be excluded from the taxonomic dataset.

As an experiment, we keep in the taxonomic dataset the 3 molluscs gastropods: albinaria_soerulea, cepaea_nemoralis, and biomphalaria_glabrata, which have a very high saturation coefficient (> 95%), but are also very close together.

We finally keep the following list:

LIST OF OTUs for the computation "lophotrochozoans_taxA":

katharina_tunicata = 0; (Mollusca Polyplacophora)

nautilus_macromphallus = 1; (Mollusca Cephalopoda)

loligo_bleekeri = 2; (Mollusca Cephalopoda)

albinaria_soerulea = 3; (Mollusca Gastropoda)

cepaea_nemoralis = 4; (Mollusca Gastropoda)

biomphalaria_glabrata = 5; (Mollusca Gastropoda)

limulus_polyphemus = 6; (Ecdysozoa) = OUTGROUP1

homo_sapiens = 7; (Ur-deuterostomia) = OUTGROUP2

PRIMARY PHYLOGENETIC HYPOTHESYS (PPH) used:

- monophyly of Lophotrochozoa = (0,1,2,3,4,5)

- monophyly of Mollusca = (0,1,2,3,4,5)

- monophyly of Polyplacophora = (0)

- monophyly of Cephalopoda = (0,1,2)

- monophyly of Gastropoda = (0,3,4,5)

Notes:

1- katharina_tunicata mtDNA is identical to octopus_vulgaris mtDNA (mollusc *Cephalopoda*), considering only the 15 protein-coding genes and rRNA genes. Thus, katharina_tunicata must be also element of Cephalopoda clade in this computation.

2- katharina_tunicata mtDNA is also identical to haliotis_rubra mtDNA (mollusca *Gastropoda*). Thus, katharina_tunicata must be also element of Gastropoda clade in this computation.

SOLUTIONS:

On domain D=[0,15], we obtain:

9 possible solutions (OK for P6) (0 impossible subtrees)

(see S8 appendix - section 'lophotrochozoans_taxA_9sol')

The analysis of all solutions shows the following results:

-> Ur-mollusca = katharina_tunicata

-> the mollusca tree is always equal to (in the 9 solutions):

R(katharina_tunicata,nautilus_macromphallus) and

R(katharina_tunicata,y) and R(y,loligo_bleekeri) and

katharina_tunicata linked with the gastropods group (3 forms)

there are 3 possible forms for the Gastropoda group (OTUs 3,4,5), and cepaea_nemoralis (= 4) is always at the base of this group, linked with katharina_tunicata by a 6-edges branch.

-> there are the 3 possible forms SOL1, SOL2, SOL3 (previously obtained) for the subtree HOLIKAT restricted to homo_sapiens, limulus_polyphemus and katharina_tunicata

ADDITIONNAL COMPUTATIONS:

-> if the PPH Cephalopoda is not imposed, we obtain many other solutions (OK for P6). For example, the following model, which do not respect PPH Cephalopoda:

R(0,n1) R(0,n2) R(1,n3) R(2,n2) R(3,4) R(3,5) R(4,3) R(4,n4) R(5,3) R(6,n6) R(7,n6) R(n1,0) R(n1,n8) R(n2,0) R(n2,2) R(n2,n3) R(n2,n6) R(n3,1) R(n3,n2) R(n4,4) R(n4,n5) R(n5,n4) R(n5,n7) R(n6,6) R(n6,7) R(n6,n2) R(n7,n5) R(n7,n8) R(n8,n1) R(n8,n7)

-> if the PPH Gastropoda is not imposed, we obtain many other solutions (OK for P6). For example, the following model, which do not respect PPH Gastropoda:

R(0,1) R(0,n1) R(0,n2) R(1,0) R(2,n2) R(3,4) R(3,5) R(4,3) R(4,n3) R(5,3) R(6,n1) R(6,n6) R(6,n8) R(7,n6) R(n1,0) R(n1,6) R(n2,0) R(n2,2) R(n3,4) R(n3,n4) R(n4,n3) R(n4,n5) R(n5,n4) R(n5,n7) R(n6,6) R(n6,7) R(n7,n5) R(n7,n8) R(n8,6) R(n8,n7)

-> if we only impose the 3 PPHs Polyplacophora, Cephalopoda and Gastropoda (but no other PPHs), we obtain the 9 solutions (OK for P6) previously obtained, which respects all the PPHs.

--------------------------------------------

(2) computation of the EUTROCHOZOA tree:

--------------------------------------------

CHOICE OF THE TAXONOMIC DATASET:

All the eutrochozoans (13), plus the nearest lophophorates (2), plus the 2 outgroups homo_sapiens (Ur-deuterostomia) and limulus_polyphemus (ecdysozoa).

katharina_tunicata (Eutrochozoa Mollusca Polyplacophora)

nautilus_macromphallus (Eutrochozoa Mollusca Cephalopoda)

loligo_bleekeri (Eutrochozoa Mollusca Cephalopoda)

albinaria_soerulea (Eutrochozoa Mollusca Gastropoda)

cepaea_nemoralis (Eutrochozoa Mollusca Gastropoda)

biomphalaria_glabrata (Eutrochozoa Mollusca Gastropoda)

platynereis_dumerilii (Eutrochozoa Annelida Polychaeta)

urechis_caupo (Eutrochozoa Annelida Echiura)

sipunculus_nudus (Eutrochozoa Sipunculida)

loxocorone_allax (Eutrochozoa Entoprocta)

gyrodactylus_derjavinoides (Eutrochozoa Platyhelminthes) ***

schistosoma_mansoni (Eutrochozoa Platyhelminthes) ***

leptorhynchoides_thecatus (Eutrochozoa Acanthocephala) ***

terebratulina_retusa (Lophophorata Brachiopoda)

phoronis_architecta (Lophophorata Phoronida)

limulus_polyphemus (Ecdysozoa) = OUTGROUP1

homo_sapiens (Ur-deuterostomia) = OUTGROUP2

but the OTUs marked with "***" have a saturation coefficient close to 100%, and must be excluded from the taxonomic dataset.

To simplify the computation, we also exclude from the taxonomic dataset the 3 molluscs Gastropoda, for which the solutions have been already computed, and we will also fix the unique form obtained for the mollusca cephalopoda group.

We finally keep the following list:

LIST OF OTUs for the computation "lophotrochozoans_taxB":

katharina_tunicata=0; (Eutrochozoa Mollusca Polyplacophora)

nautilus_macromphallus=1; (Eutrochozoa Mollusca Cephalopoda)

loligo_bleekeri=2; (Eutrochozoa Mollusca Cephalopoda)

platynereis_dumerilii=3; (Eutrochozoa Annelida Polychaeta)

urechis_caupo=4; (Eutrochozoa Annelida Echiura)

sipunculus_nudus=5; (Eutrochozoa Sipunculida)

limulus_polyphemus=6; (Ecdysozoa) = OUTGROUP1

homo_sapiens=7; (Ur-deuterostomia) = OUTGROUP2

loxocorone_allax=8; (Eutrochozoa Entoprocta)

terebratulina_retusa=9; (Lophophorata Brachiopoda)

phoronis_architecta=10; (Lophophorata Phoronida)

PRIMARY PHYLOGENETIC HYPOTHESYS (PPH) used:

- monophyly of Lophotrochozoa = (0,1,2,3,4,5,8,9,10)

- monophyly of Eutrochozoa = (0,1,2,3,4,5,8)

- monophyly of Mollusca = (0,1,2)

- monophyly of Polyplacophora = (0)

- monophyly of Cephalopoda = (0,1,2)

- monophyly of Annelida = (3,4)

- monophyly of Echiura = (3,4)

- monophyly of Polychaeta = (3)

- monophyly of Lophophorata = (9,10)

Notes:

1- katharina_tunicata mtDNA is identical to octopus_vulgaris mtDNA (mollusc *Cephalopoda*), considering only the 15 protein-coding genes and rRNA genes. Thus, katharina_tunicata must be also element of Cephalopoda clade in this computation.

2- platynereis_dumerilii mtDNA is identical to clymenella_torquata mtDNA (annelid *Echiura*). Thus, platynereis_dumerilii must be also element of *Echiura* clade in this computation.

ADDITIONNAL HYPOTHESIS:

To accelerate the computation, we fix the only possible form for the mollusca cephalopoda group (OTUs 0,1,2):

R(katharina_tunicata,nautilus_macromphallus) and

R(katharina_tunicata,y) and R(y,loligo_bleekeri) and

katharina_tunicata linked with the rest of the tree

We can also decompose the problem logically:

If we consider the subtree "HOLI" restricted to homo_sapiens and limulus_polyphemus, only 4 cases are possible:

CASE 1:

2 edges between homo_sapiens and limulus_polyphemus, 1st possible form:

R(homo_sapiens,G1) and

R(G1,limulus_polyphemus) and

G1 linked with the rest of the tree

CASE 2:

2 edges between homo_sapiens and limulus_polyphemus, 2nd possible form:

R(homo_sapiens,G1) and

R(G1,limulus_polyphemus) and

limulus_polyphemus linked with the rest of the tree

CASE 3:

3 edges between homo_sapiens and limulus_polyphemus, 1st possible form:

R(homo_sapiens,G1) and

R(G1,G2) and

R(G2,limulus_polyphemus) and

G2 linked with the rest of the tree

CASE 4:

3 edges between homo_sapiens and limulus_polyphemus, 2nd possible form:

R(homo_sapiens,G1) and

R(G1,G2) and

R(G2,limulus_polyphemus) and

G1 linked with the rest of the tree

but the computation shows that case 4 leads to less parsimonious solutions

All the other cases *necessarily* leads to less parsimonious solutions. So we can make 3 separate computations to solve the only 3 possible cases (CASE1, CASE2, CASE3).

SOLUTIONS:

-------

CASE 1:

-------

On domain D=[0,20], we obtain:

3 possible solutions (OK for P6) (146 impossible subtrees)

(see S8 appendix - section 'lophotrochozoans_taxB1_3sol')

The analysis of all solutions shows the following results:

-> Ur-eutrochozoa = katharina_tunicata

-> there is always a group "annelida+sipunculida" (OTUs 3,4,5) inside the eutrochozoa group, linked with katharina_tunicata.

-> the entoproct loxocorone_allax (OTU 8) is always linked with katharina_tunicata by a 2-edges branch:

R(katharina_tunicata,y) and

R(y,loxocorone_allax)

-> the lophophorata group (uncomplete: OTUs 9,10) is always equal to:

R(katharina_tunicata,terebratulina_retusa) and

R(katharina_tunicata,phoronis_architecta)

-> there are 3 possible forms for the eutrochozoa group (OTUs 0,1,2,3,4,5,8) with mollusca group fixed (OTUs 0,1,2)

-> the best solution is model 2 (score=65).

-> there is 1 possible form (SOL1) for the subtree HOLIKAT restricted to homo_sapiens, limulus_polyphemus and katharina_tunicata

-------

CASE 2:

-------

On domain D=[0,20], we obtain:

3 possible solutions (OK for P6, with 139 impossible subtrees)

(see S8 appendix - section 'lophotrochozoans_taxB2_3sol')

The analysis of all solutions shows the following results:

-> Ur-eutrochozoa = katharina_tunicata

-> there are 3 possible forms for the eutrochozoa group (OTUs 0,1,2,3,4,5,8) with mollusca group fixed (OTUs 0,1,2). They are exactly the same 3 forms previously obtained for CASE1

-> the lophophorata group (uncomplete: OTUs 9,10) is always equal to:

R(katharina_tunicata,terebratulina_retusa) and

R(katharina_tunicata,phoronis_architecta)

-> the best solution is model 2 (score=65)

-> there is 1 possible form (SOL2) for the subtree HOLIKAT restricted to homo_sapiens, limulus_polyphemus and katharina_tunicata

-------

CASE 3:

-------

On domain D=[0,20], we obtain:

3 possible solutions (OK for P6, with 139 impossible subtrees)

(see S8 appendix - section 'lophotrochozoans_taxB3_3sol')

The analysis of all solutions shows the following results:

-> Ur-eutrochozoa = katharina_tunicata

-> there are 3 possible forms for the eutrochozoa group (OTUs 0,1,2,3,4,5,8) with mollusca group fixed (OTUs 0,1,2). They are exactly the same 3 forms previously obtained for CASE1 and CASE2

-> the lophophorata group (uncomplete: OTUs 9,10) is always equal to:

R(katharina_tunicata,terebratulina_retusa) and

R(katharina_tunicata,phoronis_architecta)

-> the best solution is model 2 (score=57).

-> there is 1 possible form (SOL3) for the subtree HOLIKAT restricted to homo_sapiens, limulus_polyphemus and katharina_tunicata

SYNTHESIS of the 3 CASES:

-> there is convergence for the results obtained in CASE1, CASE2, and CASE3.

-> Ur-eutrochozoa = katharina_tunicata

-> there is always a group "annelida+sipunculida" (OTUs 3,4,5) inside the eutrochozoa group, linked with katharina_tunicata.

-> the entoproct loxocorone_allax (OTU 8) is always linked with katharina_tunicata by a 2-edges branch:

R(katharina_tunicata,y) and

R(y,loxocorone_allax)

-> there are 3 possible forms (SOL1, SOL2, SOL3) for the subtree HOLIKAT restricted to homo_sapiens, limulus_polyphemus and katharina_tunicata

ADDITIONNAL COMPUTATIONS:

-> if no PPH are imposed, we obtain many other solutions which do not respect the PPH. For example, the model (OK for P6):

R(0,1) R(0,9) R(0,10) R(0,12) R(0,n1) R(0,n8) R(1,0) R(2,12) R(3,n2) R(4,n5) R(5,n7) R(6,11) R(7,11) R(8,n1) R(9,0) R(10,0) R(11,6) R(11,7) R(11,n8) R(12,0) R(12,2) R(n1,0) R(n1,8) R(n2,3) R(n2,n3) R(n3,n2) R(n3,n4) R(n3,n8) R(n4,n3) R(n4,n6) R(n4,n7) R(n5,4) R(n5,n6) R(n6,n4) R(n6,n5) R(n7,5) R(n7,n4) R(n8,0) R(n8,11) R(n8,n3)

which do not respect the PPH Eutrochozoa

or the model (OK for P6):

R(0,1) R(0,10) R(0,12) R(0,n1) R(0,n2) R(0,n8) R(1,0) R(2,12) R(3,n3) R(4,n6) R(5,n7) R(6,11) R(7,11) R(8,n2) R(9,n8) R(10,0) R(11,6) R(11,7) R(11,n8) R(12,0) R(12,2) R(n1,0) R(n1,n3) R(n2,0) R(n2,8) R(n3,3) R(n3,n1) R(n3,n4) R(n3,n5) R(n4,n3) R(n4,n6) R(n5,n3) R(n5,n7) R(n6,4) R(n6,n4) R(n7,5) R(n7,n5) R(n8,0) R(n8,9) R(n8,11)

which do not respect the PPH Lophophorata

-> if we only impose the 4 PPHs Eutrochozoa, Lophophorata, Echiura and Polychaeta, we obtain - in the 3 cases - the 3 solutions (OK for P6) previously obtained which respect all the PPHs.

------------------------------------------

(3) computation of the LOPHOPHORATA tree:

------------------------------------------

CHOICE OF THE TAXONOMIC DATASET:

All the eutrochozoans in "lophotrochozoans_taxB" (7), plus all the lophophorates (8), plus the 2 outgroups homo_sapiens (Ur-deuterostomia) and limulus_polyphemus (ecdysozoa).

katharina_tunicata (Eutrochozoa Mollusca Polyplacophora)

nautilus_macromphallus (Eutrochozoa Mollusca Cephalopoda)

loligo_bleekeri (Eutrochozoa Mollusca Cephalopoda)

platynereis_dumerilii (Eutrochozoa Annelida Polychaeta)

urechis_caupo (Eutrochozoa Annelida Echiura)

sipunculus_nudus (Eutrochozoa Sipunculida)

loxocorone_allax (Eutrochozoa Entoprocta)

phoronis_architecta (Lophophorata Phoronida)

bugula_neritina (Lophophorata Bryozoa)

flustrellidra_hispida (Lophophorata Bryozoa) ***

watersipora_subtorquata (Lophophorata Bryozoa) ***

terebratulina_retusa (Lophophorata Brachiopoda)

terebratalia_transversa (Lophophorata Brachiopoda)

laqueus_rubellus (Lophophorata Brachiopoda) ***

lingula_anatina (Lophophorata Brachiopoda) ***

limulus_polyphemus (Ecdysozoa) = OUTGROUP1

homo_sapiens (Ur-deuterostomia) = OUTGROUP2

but the OTUs marked with "***" have a saturation coefficient close to 100%, and must be excluded from the taxonomic dataset.

We finally keep the following list:

LIST OF OTUs for the computation "lophotrochozoans_taxC":

katharina_tunicata=0; (Eutrochozoa Mollusca Polyplacophora)

nautilus_macromphallus=1; (Eutrochozoa Mollusca Cephalopoda)

loligo_bleekeri=2; (Eutrochozoa Mollusca Cephalopoda)

platynereis_dumerilii=3; (Eutrochozoa Annelida Polychaeta)

urechis_caupo=4; (Eutrochozoa Annelida Echiura)

sipunculus_nudus=5; (Eutrochozoa Sipunculida)

limulus_polyphemus=6; (Ecdysozoa) = OUTGROUP1

homo_sapiens=7; (Ur-deuterostomia) = OUTGROUP2

loxocorone_allax=8; (Eutrochozoa Entoprocta)

terebratulina_retusa=9; (Lophophorata Brachiopoda)

phoronis_architecta=10; (Lophophorata Phoronida)

bugula_neritina=11; (Lophophorata Bryozoa)

terebratalia_transversa=12; (Lophophorata Brachiopoda)

PRIMARY PHYLOGENETIC HYPOTHESYS (PPH) used:

- monophyly of Lophotrochozoa = (0,1,2,3,4,5,8,9,10,11,12)

- monophyly of Eutrochozoa = (0,1,2,3,4,5,8)

- monophyly of Mollusca = (0,1,2)

- monophyly of Polyplacophora = (0)

- monophyly of Cephalopoda = (0,1,2)

- monophyly of Annelida = (3,4)

- monophyly of Echiura = (3,4)

- monophyly of Polychaeta = (3)

- monophyly of Lophophorata = (9,10,11,12)

- monophyly of Brachiopoda = (9,12)

Notes:

1- katharina_tunicata mtDNA is identical to octopus_vulgaris mtDNA (mollusc *Cephalopoda*), considering only the 15 protein-coding genes and rRNA genes. Thus, katharina_tunicata must be also element of Cephalopoda clade in this computation.

2- platynereis_dumerilii mtDNA is identical to clymenella_torquata mtDNA (annelid *Echiura*). Thus, platynereis_dumerilii must be also element of *Echiura* clade in this computation.

ADDITIONNAL HYPOTHESIS:

To simplify the computation, we fix the eutrochozoa group (OTUs 0,1,2,3,4,5,8) with one of its 3 possible forms:

R(katharina_tunicata,nautilus_macromphallus) and

R(katharina_tunicata,G1) and R(G1,loligo_bleekeri) and

R(katharina_tunicata,G2) and R(G2,G3) and R(G3,platynereis_dumerilii) and

R(platynereis_dumerilii,G4) and R(G4,G5) and R(G5,urechis_caupo) and

R(G3,G6) and R(G6,G7) and R(G7,sipunculus_nudus) and

R(katharina_tunicata,G8) and R(G8,loxocorone_allax)

We also use the logical decomposition of the problem in 3 cases:

CASE 1:

2 edges between homo_sapiens and limulus_polyphemus, 1st possible form:

R(homo_sapiens,G1) and

R(G1,limulus_polyphemus) and

G1 linked with the rest of the tree

CASE 2:

2 edges between homo_sapiens and limulus_polyphemus, 2nd possible form:

R(homo_sapiens,G1) and

R(G1,limulus_polyphemus) and

limulus_polyphemus linked with the rest of the tree

CASE 3:

3 edges between homo_sapiens and limulus_polyphemus, 1st possible form:

R(homo_sapiens,G1) and

R(G1,G2) and

R(G2,limulus_polyphemus) and

G2 linked with the rest of the tree

SOLUTIONS:

------

CASE 1:

------

On domain D=[0,29], we obtain:

9 possible solutions (OK for P6) (164 impossible subtrees)

(see S8 appendix - section 'lophotrochozoans_taxC1_9sol')

The analysis of all solutions shows the following results:

-> Ur-lophophorata = katharina_tunicata

-> there are 9 possible forms for the lophophorata group (OTUs 9,10,11,12)

-> the best solution is model 1 (score=100)

-> there is 1 possible form (SOL1) for the subtree HOLIKAT restricted to homo_sapiens, limulus_polyphemus and katharina_tunicata

-------

CASE 2:

-------

On domain D=[0,29], we obtain:

9 possible solutions (OK for P6, with 167 impossible subtrees)

(see S8 appendix - section 'lophotrochozoans_taxC2_9sol')

The analysis of all solutions shows the following results:

-> Ur-lophophorata = katharina_tunicata

-> there are 9 possible forms for the lophophorata group (OTUs 9,10,11,12). They are exactly the same 9 forms previously obtained for CASE1

-> the best solution is model 1 (score=100).

-> there is 1 possible form (SOL2) for the subtree HOLIKAT restricted to homo_sapiens, limulus_polyphemus and katharina_tunicata

-------

CASE 3:

-------

On domain D=[0,29], we obtain:

9 possible solutions (OK for P6, with 167 impossible subtrees)

(see S8 appendix - section 'lophotrochozoans_taxC3_9sol')

The analysis of all solutions shows the following results:

-> Ur-lophophorata = katharina_tunicata

-> there are 9 possible forms for the lophophorata group (OTUs 9,10,11,12). They are exactly the same 9 forms previously obtained for CASE1 and CASE2

-> the best solution is model 1 (score=90).

-> there is 1 possible form (SOL3) for the subtree HOLIKAT restricted to homo_sapiens, limulus_polyphemus and katharina_tunicata

SYNTHESIS of the 3 CASES:

-> there is convergence for the results obtained in CASE1, CASE2, and CASE3.

-> Ur-lophophorata = Ur-lophotrochozoa = katharina_tunicata

-> there are 3 possible forms (SOL1, SOL2, SOL3) for the subtree HOLIKAT restricted to homo_sapiens, limulus_polyphemus and katharina_tunicata

ADDITIONNAL COMPUTATIONS:

-> if no PPH are imposed, we obtain other solutions which do not respect the PPH. For example, the model (OK for P6):

R(0,1) R(0,10) R(0,13) R(0,14) R(0,20) R(0,n1) R(0,n2) R(1,0) R(2,13) R(3,15) R(3,16) R(4,17) R(5,19) R(6,21) R(6,n1) R(7,21) R(8,20) R(9,n1) R(9,n3) R(10,0) R(11,n6) R(12,n8) R(13,0) R(13,2) R(14,0) R(14,15) R(15,3) R(15,14) R(15,18) R(16,3) R(16,17) R(17,4) R(17,16) R(18,15) R(18,19) R(19,5) R(19,18) R(20,0) R(20,8) R(21,6) R(21,7) R(n1,0) R(n1,6) R(n1,9) R(n2,0) R(n2,n7) R(n3,9) R(n3,n4) R(n4,n3) R(n4,n5) R(n5,n4) R(n5,n8) R(n6,11) R(n6,n7) R(n7,n2) R(n7,n6) R(n8,12) R(n8,n5)

which do not respect the PPH Lophophorata

or the model (OK for P6):

R(0,1) R(0,9) R(0,13) R(0,14) R(0,20) R(0,n1) R(0,n2) R(0,n3) R(1,0) R(2,13) R(3,15) R(3,16) R(4,17) R(5,19) R(6,21) R(6,n1) R(7,21) R(8,20) R(9,0) R(10,n3) R(11,n4) R(12,n6) R(13,0) R(13,2) R(14,0) R(14,15) R(15,3) R(15,14) R(15,18) R(16,3) R(16,17) R(17,4) R(17,16) R(18,15) R(18,19) R(19,5) R(19,18) R(20,0) R(20,8) R(21,6) R(21,7) R(n1,0) R(n1,6) R(n2,0) R(n2,n5) R(n3,0) R(n3,10) R(n3,n7) R(n4,11) R(n4,n5) R(n5,n2) R(n5,n4) R(n6,12) R(n6,n8) R(n7,n3) R(n7,n8) R(n8,n6) R(n8,n7)

which do not respect the PPH Brachiopoda

-> if we only impose the 2 PPHs Lophophorata and Brachiopoda, we obtain - in the 3 cases - the 9 solutions (OK for P6) previously obtained which respect all the PPHs.

=======================================================

LOPHOTROCHOZOANS - PART B (with priapulus_caudatus)

=======================================================

Using the outgroup priapulus_caudatus (ecdysozoa)

(we make again the computations of part A, but with the outgroup priapulus_caudatus instead of limulus_polyphemus)

---------------------------------------

(1) computation of the MOLLUSCA tree:

---------------------------------------

LIST OF OTUs for the computation "lophotrochozoans_taxD":

katharina_tunicata = 0; (Mollusca Polyplacophora)

nautilus_macromphallus = 1; (Mollusca Cephalopoda)

loligo_bleekeri = 2; (Mollusca Cephalopoda)

albinaria_soerulea = 3; (Mollusca Gastropoda)

cepaea_nemoralis = 4; (Mollusca Gastropoda)

biomphalaria_glabrata = 5; (Mollusca Gastropoda)

priapulus_caudatus = 6; (Ecdysozoa) = OUTGROUP1

homo_sapiens = 7; (Ur-deuterostomia) = OUTGROUP2

(the same OTUs as in computation "lophotrochozoans_taxA", but with priapulus_caudatus instead of limulus_polyphemus)

PRIMARY PHYLOGENETIC HYPOTHESYS (PPH) used:

- monophyly of Lophotrochozoa = (0,1,2,3,4,5)

- monophyly of Mollusca = (0,1,2,3,4,5)

- monophyly of Polyplacophora = (0)

- monophyly of Cephalopoda = (0,1,2)

- monophyly of Gastropoda = (0,3,4,5)

Notes:

1- katharina_tunicata mtDNA is identical to octopus_vulgaris mtDNA (mollusc *Cephalopoda*), considering only the 15 protein-coding genes and rRNA genes. Thus, katharina_tunicata must be also element of Cephalopoda clade in this computation.

2- katharina_tunicata mtDNA is also identical to haliotis_rubra mtDNA (mollusc *Gastropoda*). Thus, katharina_tunicata must be also element of Gastropoda clade in this computation.

SOLUTIONS:

On domain D=[0,15], we obtain:

6 possible solutions (OK for P6) (1 impossible subtree)

(see S8 appendix - section 'lophotrochozoans_taxD_6sol')

The analysis of all solutions shows the following results:

-> Ur-mollusca = katharina_tunicata

-> the mollusca tree is always equal to:

R(katharina_tunicata,nautilus_macromphallus) and

R(katharina_tunicata,y) and R(y,loligo_bleekeri) and

katharina_tunicata linked with the gastropods group (3 forms)

there are 3 possible forms for the Gastropoda group (OTUs 3,4,5), and cepaea_nemoralis (= 4) is always at the base of this group, linked with katharina_tunicata by a 6-edges branch.

Thus, the possible forms for the mollusca tree are identical to the forms previously obtained in computation "lophotrochozoans_taxA" (with the outgroup limulus_polyphemus)

-> there are 2 possible forms SOL1_ALTER and SOL2_ALTER (previously obtained in the computations for ecdysozoans) for the subtree "HOPRIAKAT" restricted to homo_sapiens, priapulus_caudatus and katharina_tunicata

* form SOL1_ALTER:

R(homo_sapiens,x1) and

R(x1,priapulus_caudatus) and

R(x1,x2) and

R(x2,katharina_tunicata)

* form SOL2_ALTER:

R(homo_sapiens,x1) and

R(x1,priapulus_caudatus) and

R(priapulus_caudatus,x2) and

R(x2,katharina_tunicata)

ADDITIONNAL COMPUTATIONS:

-> if we only impose the 3 PPHs Polyplacophora, Cephalopoda and Gastropoda (but no other PPHs), we obtain the same 6 solutions (OK for P6) previously obtained, which respects all the PPHs.

--------------------------------------------

(2) computation of the EUTROCHOZOA tree:

--------------------------------------------

LIST OF OTUs for the computation "lophotrochozoans_taxE":

katharina_tunicata=0; (Eutrochozoa Mollusca Polyplacophora)

nautilus_macromphallus=1; (Eutrochozoa Mollusca Cephalopoda)

loligo_bleekeri=2; (Eutrochozoa Mollusca Cephalopoda)

platynereis_dumerilii=3; (Eutrochozoa Annelida Polychaeta)

urechis_caupo=4; (Eutrochozoa Annelida Echiura)

sipunculus_nudus=5; (Eutrochozoa Sipunculida)

priapulus_caudatus=6; (Ecdysozoa) = OUTGROUP1

homo_sapiens=7; (Ur-deuterostomia) = OUTGROUP2

loxocorone_allax=8; (Eutrochozoa Entoprocta)

terebratulina_retusa=9; (Lophophorata Brachiopoda)

phoronis_architecta=10; (Lophophorata Phoronida)

(the same OTUs as in "lophotrochozoans_taxB", but with priapulus_caudatus instead of limulus_polyphemus)

PRIMARY PHYLOGENETIC HYPOTHESYS (PPH) used:

- monophyly of Lophotrochozoa = (0,1,2,3,4,5,8,9,10)

- monophyly of Eutrochozoa = (0,1,2,3,4,5,8)

- monophyly of Mollusca = (0,1,2)

- monophyly of Polyplacophora = (0)

- monophyly of Cephalopoda = (0,1,2)

- monophyly of Annelida = (3,4)

- monophyly of Echiura = (3,4)

- monophyly of Polychaeta = (3)

- monophyly of Lophophorata = (9,10)

Notes:

1- katharina_tunicata mtDNA is identical to octopus_vulgaris mtDNA (mollusc *Cephalopoda*), considering only the 15 protein-coding genes and rRNA genes. Thus, katharina_tunicata must be also element of Cephalopoda clade in this computation.

2- platynereis_dumerilii mtDNA is identical to clymenella_torquata mtDNA (annelid *Echiura*). Thus, platynereis_dumerilii must be also element of *Echiura* clade in this computation.

ADDITIONNAL HYPOTHESIS:

To accelerate the computation, we fix the unique possible form for the mollusca cephalopoda group (OTUs 0,1,2):

R(katharina_tunicata,nautilus_macromphallus) and

R(katharina_tunicata,G2) and

R(G2,loligo_bleekeri) and

katharina_tunicata linked with the rest of the tree

We can also decompose the problem logically:

If we consider the subtree "HOPRIA" restricted to homo_sapiens and priapulus_caudatus, only 2 cases are possible:

CASE 1:

2 edges between homo_sapiens and priapulus_caudatus, 1st possible form:

R(homo_sapiens,G1) and

R(G1,priapulus_caudatus) and

G1 linked with the rest of the tree

CASE 2:

2 edges between homo_sapiens and priapulus_caudatus, 2nd possible form:

R(homo_sapiens,G1) and

R(G1,priapulus_caudatus) and

priapulus_caudatus linked with the rest of the tree

All the other cases necessarily lead to less parsimonious solutions. So we can make 2 separate computations to solve the only 2 possible cases (CASE1, CASE2).

SOLUTIONS:

-------

CASE 1:

-------

On domain D=[0,20], we obtain:

3 possible solutions (OK for P6) (99 impossible subtrees)

(see S8 appendix - section 'lophotrochozoans_taxE1_3sol')

The analysis of all solutions shows the following results:

-> Ur-eutrochozoa = katharina_tunicata

-> there are 3 possible forms for the eutrochozoa group (OTUs 0,1,2,3,4,5,8) with mollusca group fixed (OTUs 0,1,2). The possible forms for the eutrochozoa tree are identical to the forms previously obtained in "lophotrochozoans_taxB" (with the outgroup limulus_polyphemus)

-> the best solution is model 2 (score=66).

-> the lophophorata group (uncomplete: OTUs 9,10) is always equal to:

R(katharina_tunicata,terebratulina_retusa) and

R(katharina_tunicata,phoronis_architecta)

-> there is 1 possible form (SOL1_ALTER) for the subtree HOPRIAKAT restricted to homo_sapiens, priapulus_caudatus and katharina_tunicata

-------

CASE 2:

-------

On domain D=[0,20], we obtain:

3 possible solutions (OK for P6) (108 impossible subtrees)

see file "lophotrochozoans_taxE2_(3sol).rtf"

The analysis of all solutions shows the following results:

-> Ur-eutrochozoa = katharina_tunicata

-> there are 3 possible forms for the eutrochozoa group (OTUs 0,1,2,3,4,5,8) with mollusca group fixed (OTUs 0,1,2). They are exactly the same 3 forms previously obtained for CASE1 and for "lophotrochozoans_taxB" (with the outgroup limulus_polyphemus)

-> the lophophorata group (uncomplete: OTUs 9,10) is always equal to:

R(katharina_tunicata,terebratulina_retusa) and

R(katharina_tunicata,phoronis_architecta)

-> the best solution is model 2 (score=66)

-> there is 1 possible form (SOL2_ALTER) for the subtree HOPRIAKAT restricted to homo_sapiens, priapulus_caudatus and katharina_tunicata

ADDITIONNAL COMPUTATIONS:

-> if we only impose the 4 PPHs Eutrochozoa, Lophophorata, Echiura and Polychaeta, we obtain - in the 2 cases - the 3 solutions (OK for P6) previously obtained which respect all the PPHs.

------------------------------------------

(3) computation of the LOPHOPHORATA tree:

------------------------------------------

LIST OF OTUs for the computation "lophotrochozoans_taxF":

katharina_tunicata=0; (Eutrochozoa Mollusca Polyplacophora)

nautilus_macromphallus=1; (Eutrochozoa Mollusca Cephalopoda)

loligo_bleekeri=2; (Eutrochozoa Mollusca Cephalopoda)

platynereis_dumerilii=3; (Eutrochozoa Annelida Polychaeta)

urechis_caupo=4; (Eutrochozoa Annelida Echiura)

sipunculus_nudus=5; (Eutrochozoa Sipunculida)

priapulus_caudatus=6; (Ecdysozoa) = OUTGROUP1

homo_sapiens=7; (Ur-deuterostomia) = OUTGROUP2

loxocorone_allax=8; (Eutrochozoa Entoprocta)

terebratulina_retusa=9; (Lophophorata Brachiopoda)

phoronis_architecta=10; (Lophophorata Phoronida)

bugula_neritina=11; (Lophophorata Bryozoa)

terebratalia_transversa=12; (Lophophorata Brachiopoda)

(the same OTUs as in "lophotrochozoans_taxC", but with priapulus_caudatus instead of limulus_polyphemus)

PRIMARY PHYLOGENETIC HYPOTHESYS (PPH) used:

- monophyly of Lophotrochozoa = (0,1,2,3,4,5,8,9,10,11,12)

- monophyly of Eutrochozoa = (0,1,2,3,4,5,8)

- monophyly of Mollusca = (0,1,2)

- monophyly of Polyplacophora = (0)

- monophyly of Cephalopoda = (0,1,2)

- monophyly of Annelida = (3,4)

- monophyly of Echiura = (3,4)

- monophyly of Polychaeta = (3)

- monophyly of Lophophorata = (9,10,11,12)

- monophyly of Brachiopoda = (9,12)

Notes:

1- katharina_tunicata mtDNA is identical to octopus_vulgaris mtDNA (mollusc *Cephalopoda*), considering only the 15 protein-coding genes and rRNA genes. Thus, katharina_tunicata must be also element of Cephalopoda clade in this computation.

2- platynereis_dumerilii mtDNA is identical to clymenella_torquata mtDNA (annelid *Echiura*). Thus, platynereis_dumerilii must be also element of *Echiura* clade in this computation.

ADDITIONNAL HYPOTHESIS:

To simplify the computation, we fix the eutrochozoa group (OTUs 0,1,2,3,4,5,8) with one of its 3 possible forms:

R(katharina_tunicata,nautilus_macromphallus) and

R(katharina_tunicata,G1) and R(G1,loligo_bleekeri) and

R(katharina_tunicata,G2) and R(G2,G3) and R(G3,platynereis_dumerilii) and

R(platynereis_dumerilii,G4) and R(G4,G5) and R(G5,urechis_caupo) and

R(G3,G6) and R(G6,G7) and R(G7,sipunculus_nudus) and

R(katharina_tunicata,G8) and R(G8,loxocorone_allax)

We also use the logical decomposition of the problem in 2 cases:

CASE 1:

2 edges between homo_sapiens and priapulus_caudatus, 1st possible form:

R(homo_sapiens,G1) and

R(G1,priapulus_caudatus) and

G1 linked with the rest of the tree

CASE 2:

2 edges between homo_sapiens and priapulus_caudatus, 2nd possible form:

R(homo_sapiens,G1) and

R(G1,priapulus_caudatus) and

priapulus_caudatus linked with the rest of the tree

SOLUTIONS:

-------

CASE 1:

-------

On domain D=[0,29], we obtain:

9 possible solutions (OK for P6) (128 impossible subtrees)

(see S8 appendix - section 'lophotrochozoans_taxF1_9sol')

The analysis of all solutions shows the following results:

-> Ur-lophophorata = katharina_tunicata

-> there are 9 possible forms for the lophophorata group (OTUs 9,10,11,12). The possible forms for the lophophorata tree are identical to the forms previously obtained in computation "lophotrochozoans_taxC" (with the outgroup limulus_polyphemus)

-> the best solution is model 1 (score=101)

-> there is 1 possible form (SOL1_ALTER) for the subtree HOPRIAKAT restricted to homo_sapiens, priapulus_caudatus and katharina_tunicata

-------

CASE 2:

-------

On domain D=[0,29], we obtain:

9 possible solutions (OK for P6, with 143 impossible subtrees)

(see S8 appendix - section 'lophotrochozoans_taxF2_9sol')

The analysis of all solutions shows the following results:

-> Ur-lophophorata = katharina_tunicata

-> there are 9 possible forms for the lophophorata group (OTUs 9,10,11,12). They are exactly the same 9 forms previously obtained for CASE1 and for computation "lophotrochozoans_taxC" (with outgroup = limulus_polyphemus)

-> the best solution is model 2 (score=101)

-> there is 1 possible form (SOL2_ALTER) for the subtree HOPRIAKAT restricted to homo_sapiens, priapulus_caudatus and katharina_tunicata

ADDITIONNAL COMPUTATIONS:

-> if we only impose the 2 PPHs Lophophorata and Brachiopoda, we obtain in the 2 cases the 9 solutions (OK for P6) previously obtained which respect all the PPHs.

CONCLUSION:

the solutions obtained for the *complete lophotrochozoa group* (part B: with outgroup = priapulus_caudatus) are identical to those previously obtained (part A: with outgroup = limulus_polyphemus), and always with Ur-lophotrochozoa = katharina_tunicata

For the deuterostomes too, one last computation is necessary:

=======================================================

DEUTEROSTOMES - PART B (with priapulus_caudatus)

=======================================================

With the outgroup priapulus_caudatus instead of limulus_polyphemus

------------------------------------------------------------

(1) computation of the DEUTEROSTOMIA tree with 2 outgroups:

------------------------------------------------------------

CHOICE OF THE TAXONOMIC DATASET:

All the OTUs already in "deuterostomes_taxB", but with the outgroup priapulus_caudatus (Ecdysozoa) instead of limulus_polyphemus

LIST OF OTUs for the computation "deuterostomes_taxD":

homo_sapiens = 0 Chordata Craniata

asymmetron_inferum = 1 Chordata Cephalochordata

priapulus_caudatus = 2 Ecdysozoa = OUTGROUP1

xenoturbella_bocki = 3 Xenoturbellidea

balanoglossus_carnosus = 4 Hemichordata

strongylocentrotus_purpuratus = 5 Echinodermata

ophiura_lukteni = 6 Echinodermata Ophiurida

ophiobolis_aculeata = 7 Echinodermata Ophiurida

asterina_pectinifera = 8 Echinodermata

florometra_serratissima = 9 Echinodermata Crinoidea

gymnocrinus_richeri = 10 Echinodermata Crinoidea

antedon_mediterranea = 11 Echinodermata Crinoidea

katharina_tunicata = 12 Lophotrochozoa = OUTGROUP2

(same OTUs as in computation "deuterostomes_taxB", but with priapulus_caudatus instead of limulus_polyphemus)

PRIMARY PHYLOGENETIC HYPOTHESYS (PPH) used:

- monophyly of Deuterostomia = (0,1,3,4,5,6,7,8,9,10,11)

- monophyly of Chordata = (0,1)

- monophyly of Ambulacria = (4,5,6,7,8,9,10,11)

- monophyly of Echinodermata = (5,6,7,8,9,10,11)

- monophyly of Ophiurida = (6,7)

- monophyly of Crinoidea = (9,10,11)

ADDITIONAL HYPOTHESIS:

First, to divide by 3 the number of solutions obtained, we fix one of the 3 possible forms for the crinoidea group:

R(florometra_serratissima, gymnocrinus_richeri) and

R(florometra_serratissima, antedon_mediterranea) and

florometra_serratissima linked with the rest of the tree

(Ur-pelmatozoa = florometra_serratissima)

SOLUTIONS:

On domain D=[0,16], we obtain:

42 possible solutions (OK for property P6) (10 impossible subtrees)

(see S6 appendix - section 'deuterostomes_taxD_42sol')

The analysis of all solutions shows the following results:

-> Ur-deuterostomia = homo_sapiens

-> the deuterostomia tree is always equal to:

R(homo_sapiens,xenoturbella_bocki) and

R(homo_sapiens,y) and

R(y,asymmetron_inferum) and

R(homo_sapiens,balanoglossus_carnosus) and

R(homo_sapiens,z) and

z linked with the echinodermata group (21 forms, with crinoidea fixed)

-> there is 1 possible form for the ophiurida group (OTUs 6,7), and ophiobolis_aculeata is at the base of the group:

R(ophiobolis_aculeata,ophiura_lukteni) and

ophiobolis_aculeata linked with the echinodermata group

-> there are 21 possible forms for the echinodermata group with the crinoidea group fixed (the same solutions previously obtained in "deuterostomes_taxA")

-> there are 2 possible forms for the subtree HOPRIAKAT restricted to homo_sapiens, priapulus_caudatus and katharina_tunicata:

form outgroup SOL1_ALTER:

R(homo_sapiens,x1) and

R(x1,priapulus_caudatus) and

R(x1,x2) and

R(x2,katharina_tunicata)

form outgroup SOL2_ALTER:

R(homo_sapiens,x1) and

R(x1,priapulus_caudatus) and

R(priapulus_caudatus,x2) and

R(x2,katharina_tunicata)

ADDITIONNAL COMPUTATIONS:

-> if we do not fix the crinoidea group, we obtain (12h of computation): 42x3 = 126 solutions (OK for P6): each of the 42 solutions previously obtained now combines with the 3 possible forms for the crinoidea group.

-> if we only impose the PPH Ambulacria (and without fixing the crinoidea group), we obtain the same 126 solutions (OK for P6) previously obtained which verify all the PPHs. The other PPHs are logical consequences of the problem.

CONCLUSION:

the solutions obtained here for the deuterostomes (with outgroup priapulus_caudatus) are identical to those previously obtained (with outgroup limulus_polyphemus) in computation "deuterostomes_taxB".

============================================

the base of the BILATERIAN TREE

============================================

-------------------------------------------------------------------------

(1) computation of the BILATERIAN tree with tethya_actinia as outgroup

-------------------------------------------------------------------------

CHOICE OF THE TAXONOMIC DATASET:

homo_sapiens (= Ur-deuterostomia), limulus_polyphemus and priapulus_caudatus (ecdysozoa), katharina_tunicata (= Ur-lophotrochozoa), and the outgroup tethya_actinia (demospongiae)

LIST OF OTUs for the computation "bilaterians_taxA":

homo_sapiens=0; (Ur-deuterostomia)

priapulus_caudatus=1; (Ecdysozoa)

limulus_polyphemus=2; (Ecdysozoa)

katharina_tunicata=3; (Lophotrochozoa)

tethya_actinia=4; (Demospongiae) = OUTGROUP

PRIMARY PHYLOGENETIC HYPOTHESYS (PPH) used:

- monophyly of Deuterostomia = (0,1,2,3)

- monophyly of Ecdysozoa = (1,2)

SOLUTIONS:

On domain D=[0,9], we obtain:

6 possible solutions (OK for P6) (8 impossible subtrees).

(see S9 appendix - section 'bilaterians_taxA_6sol')

The analysis of all solutions shows the following results:

-> Ur-bilateria = Ur-deuterostomia = homo_sapiens

-> 1 possible form for the outgroup tethya_actinia (demospongiae), linked with homo_sapiens by a 5-edges branch (4 + 1 loss of gene atp9)

-> for the subtree "BASE" restricted to homo_sapiens, priapulus_caudatus, limulus_polyphemus and katharina_tunicata, there are 6 possible forms:

form BASE1: ( = SOL1 + R(limulus_polyphemus,priapulus_caudatus) )

R(homo_sapiens,x1) and

R(x1,limulus_polyphemus) and

R(x1,x2) and

R(x2,katharina_tunicata) and

R(limulus_polyphemus,priapulus_caudatus)

form BASE2: ( = SOL2 + R(limulus_polyphemus,priapulus_caudatus) )

R(homo_sapiens,x1) and

R(x1,limulus_polyphemus) and

R(limulus_polyphemus,x2) and

R(x2,katharina_tunicata) and

R(limulus_polyphemus,priapulus_caudatus)

form BASE3: ( = SOL3 + R(limulus_polyphemus,priapulus_caudatus) )

R(homo_sapiens,x1) and

R(x1,x2) and

R(x2,limulus_polyphemus) and

R(x2,katharina_tunicata) and

R(limulus_polyphemus,priapulus_caudatus)

form BASE4: ( = SOL1_ALTER + R(priapulus_caudatus,limulus_polyphemus) )

R(homo_sapiens,x1) and

R(x1,priapulus_caudatus) and

R(x1,x2) and

R(x2,katharina_tunicata) and

R(priapulus_caudatus,limulus_polyphemus)

form BASE5: ( = SOL2_ALTER + R(priapulus_caudatus,limulus_polyphemus) )

R(homo_sapiens,x1) and

R(x1,priapulus_caudatus) and

R(priapulus_caudatus,x2) and

R(x2,katharina_tunicata) and

R(priapulus_caudatus,limulus_polyphemus)

form BASE6: ( = SOL1 + SOL1_ALTER)

R(homo_sapiens,x1) and

R(x1,limulus_polyphemus) and

R(x1,priapulus_caudatus) and

R(x1,x2) and

R(x2,katharina_tunicata)

==============================================

BILATERIANS - SYNTHESIS OF ALL THE SOLUTIONS

==============================================

DEUTEROSTOMES: (63 possible forms)

-> Ur-deuterostomia = homo_sapiens

-> Ur-ambulacria = Ur-chordata = homo_sapiens

-> the deuterostomia tree is always equal to:

R(homo_sapiens,xenoturbella_bocki) and

R(homo_sapiens,y) and

R(y,asymmetron_inferum) and

R(homo_sapiens,balanoglossus_carnosus) and

R(homo_sapiens,z) and

z linked with the echinodermata group

echinodermata:

-> 1 possible form for the ophiurida group

(and Ur-ophiurida = ophiobolis_aculeata)

-> 3 possible forms for the crinoidea group

(and Ur-pelmatozoa = florometra_serratissima)

-> 21 possible forms for the rest of the echinodermata group

(with ophiobolis_aculeata and florometra_serratissima)

Those possible forms can freely combine (the total number of solutions is multiplied consequently), so there are:

1x3x21 = 63 possibles forms for the complete echinodermata group

-> but only 1 form (among 3) is possible for the crinoidea group after making a local analysis of the crinoids with the tRNAs genes

-> with the additional primary phylogenetic hypothesis:

monophyly of Eleutherozoa

only 2 forms (among 21) are possible for the echinodermata group (and thus for the deuterostomia group)

-> with the additional primary phylogenetic hypothesis:

monophyly of Eleutherozoa

monophyly of (Asteroidea+Echinoidea)

only 1 form (among 63) is possible for the deuterostomia group

-> with the additional primary phylogenetic hypothesis:

monophyly of Eleutherozoa

monophyly of Cryptosyringid

only 1 form (among 63) is possible for the deuterostomia group

-> with the additional primary phylogenetic hypothesis:

monophyly of Eleutherozoa

monophyly of Asterozoa

only 2 form (among 63) are possible for the deuterostomia group

ECDYSOZOANS: (4212 possible forms)

-> Ur-panarthropoda = limulus_polyphemus

-> Ur-chelicerata = Ur-mandibulata = Ur-arthropoda = limulus_polyphemus

-> there are 2 possible values for Ur-introverta:

Ur-introverta = priapulus_caudatus

(see models 1,2,3,4,6,7, in "ecdysozoans_taxG")

Ur-introverta = UR1 =

[cox1 cox2 atp8 atp6 cox3 nad3 -nad5 -nad4 -nad4L nad6 cob rrnS rrnL nad1 nad2]

(see model 5, in "ecdysozoans_taxG")

-> there are 3 possible values for Ur-ecdysozoa:

Ur-ecdysozoa = limulus_polyphemus

(see models 1,2,3, in "ecdysozoans_taxG")

Ur-ecdysozoa = priapulus_caudatus

(see models 6,7, in "ecdysozoans_taxG")

Ur-ecdysozoa = UR1 =

[cox1 cox2 atp8 atp6 cox3 nad3 -nad5 -nad4 -nad4L nad6 cob rrnS rrnL nad1 nad2]

(see models 4,5, in "ecdysozoans_taxG")

-> in the crustacea decapoda group, there is always:

R(limulus_polyphemus,eriocheir_sinensis)

-> in the mandibulata group, there is always:

R(limulus_polyphemus,narceus_annularis)

-> in the onychophora group, there is always:

R(limulus_polyphemus,x1) and R(x1,x2) and R(x2,epiperipatus_biolleyi)

-> 3 possible forms for the crustacea decapoda group

-> 39 possible forms for the rest of the mandibulata group

(3x39 = 117 possible forms for the mandibulata group)

-> 9 possible forms for the chelicerata group

(with 6 distinct forms for the acari group)

(117x9 = 1053 possible forms for the arthropoda group)

-> 1 possible form for the onychophora group (epiperipatus_biolleyi)

(1x1053 = 1053 possible forms for the panarthropoda group)

-> 4 possible forms for the introverta group

(4x1053= 4212 possible forms for the ecdysozoa group)

LOPHOTROCHOZOANS: (81 possible forms)

-> Ur-lophotrochozoa = katharina_tunicata

-> Ur-mollusca = Ur-eutrochozoa = Ur-lophophorata = katharina_tunicata

-> the mollusca tree is always equal to: R(katharina_tunicata,nautilus_macromphallus) and

R(katharina_tunicata,y) and

R(y,loligo_bleekeri) and

katharina_tunicata linked with the Gastropoda group (3 possible forms)

-> there are 3 possible forms for the Gastropoda group, and cepaea_nemoralis is always at the base of the group, linked with katharina_tunicata by a 6-edges branch.

-> in the eutrochozoa group, there is always:

R(katharina_tunicata,y) and R(y,loxocorone_allax)

-> in the eutrochozoa group, there is always a group "annelida+sipunculida" (3 possible forms), linked with katharina_tunicata.

-> in the lophophorata group, there is always:

R(katharina_tunicata,phoronis_architecta)

-> 3 possible forms for the Gastropoda group (molluscs)

(1x3= 3 possible forms for the mollusca group)

-> 3 possible forms for the rest of the eutrochozoa group

(1x3x3 = 9 possible forms for the eutrochozoa group)

-> 9 possible forms for the lophophorata group

(1x3x3x9 = 81 possible forms for the lophotrochozoa group)

THE BASE OF THE BILATERIANS: (6 possible forms)

-> Ur-bilateria = homo_sapiens

-> 1 possible form for the outgroup tethya_actinia (demospongiae), linked with homo_sapiens by a 5-edges branch (4 + 1 loss of gene atp9)

-> 6 possible forms (BASE1 to BASE6 - see Figure 3) for the subtree "BASE" restricted to homo_sapiens, priapulus_caudatus, limulus_polyphemus and katharina_tunicata

GLOBAL RESULTS:

-> in each tree solution obtained for bilaterians (without chaetognaths), there are:

44 OTUs (43 OTUS bilaterians + 1 outgroup tethya_actinia) and

46 HTUs

= 90 nodes and 89 edges (including 1 "loss mutation" edge)

-> 21 OTUs (bilaterians) have been excluded from the taxonomic datasets, because they have a saturation coefficient close to 100 %:

doliolum_nationalis (Urochordata)

ciona_savignyi (Urochordata)

ciona_intestinalis (Urochordata)

phallusia_mammillata (Urochordata)

phallusia_fumigata (Urochordata)

halocynthia_roretzi (Urochordata)

tigriopus_japonicus (Mandibulata Crustacea Copepoda)

caenorhabditis_elegans (Introverta Nematoda)

siphonodentalium_lobatum (Mollusca Scaphopoda)

graptacme_eborea (Mollusca Scaphopoda)

mytilus_edulis (Mollusca Bivalvia)

lampsilis_ornata (Mollusca Bivalvia)

venerupis_philippinarum (Mollusca Bivalvia)

inversidens_japanensis (Mollusca Bivalvia)

gyrodactylus_derjavinoides (Eutrochozoa Platyhelminthes)

schistosoma_mansoni (Eutrochozoa Platyhelminthes)

leptorhynchoides_thecatus (Eutrochozoa Acanthocephala)

flustrellidra_hispida (Lophophorata Bryozoa)

watersipora_subtorquata (Lophophorata Bryozoa)

laqueus_rubellus (Lophophorata Brachiopoda)

lingula_anatina (Lophophorata Brachiopoda)

their position in the trees must be determined with other methods.

-> the tree solutions for the complete bilateria group (without chaetognaths) have been calculated with the combination of 33 smaller computations (see S6-S9 appendix).

-> the 28 PPHs used in this study:

for bilaterians:

PPH Bilateria

for deuterostomes:

PPH Deuterostomia

PPH Chordata

PPH Ambulacria

PPH Echinodermata

PPH Eleutherozoa

PPH Ophiurida

PPH Crinoidea

for ecdysozoans:

PPH Ecdysozoa

PPH Arthropoda

PPH Mandibulata

PPH Crustacea

PPH Decapodea

PPH Chelicerata

PPH Acari

PPH Panarthropoda

PPH Introverta

for lophotrochozoans:

PPH Lophotrochozoa

PPH Eutrochozoa

PPH Mollusca

PPH Polyplacophora

PPH Cephalopoda

PPH Gastropoda

PPH Annelida

PPH Echiura

PPH Polychaeta

PPH Lophophorata

PPH Brachiopoda

-> among them, 21 PPHs are *necessary* (to obtain the same results):

for bilaterians:

PPH Bilateria

for deuterostomes:

PPH Deuterostomia

PPH Ambulacria

PPH Eleutherozoa

for ecdysozoans:

PPH Ecdysozoa

PPH Arthropoda

PPH Mandibulata

PPH Crustacea

PPH Decapoda

PPH Chelicerata

PPH Introverta

for lophotrochozoans:

PPH Lophotrochozoa

PPH Eutrochozoa

PPH Mollusca

PPH Polyplacophora

PPH Cephalopoda

PPH Gastropoda

PPH Echiura

PPH Polychaeta

PPH Lophophorata

PPH Brachiopoda

Note: among these 21 PPHs, 6 are used implicitly because of outgroup choice to root analyses:

PPH Bilateria

PPH Deuterostomia

PPH Arthropoda

PPH Decapoda

PPH Lophotrochozoa

PPH Mollusca

All the other PPHs (7) are logical consequences of the PHYLO problem:

PPH Chordata

PPH Echinodermata

PPH Ophiurida

PPH Crinoidea

PPH Acari

PPH Panarthropoda

PPH Annelida

-------------------------------

verification of the solutions:

-------------------------------

All the tree solutions can be verified with the program *genome_comparison.c*, by calculating the paths between nodes.

To make easier those verifications, all the possible values for the ancestral states of degree > 2 are given in the solution files (except redundancies).
