# Supplementary material for: A complete logical approach to resolve the evolution and dynamics of mitochondrial genome in bilaterians
Source: PLoS One. 2018 Mar 16;13(3):e0194334. doi: 10.1371/journal.pone.0194334 (PMC5856267; doi:10.1371/journal.pone.0194334)
Supplement: S6 Appendix — (DOC) [file pone.0194334.s006.doc]

S6 Appendix. Axioms and solutions for Deuterostomia.

deuterostomes_taxA_63sol page 2

deuterostomes_taxA_v2_6sol page 17

deuterostomes_taxA_v3_3sol page 22

deuterostomes_taxA_v4_3sol page 26

deuterostomes_taxA_v5_6sol page 30

deuterostomes_taxA_v6_21sol page 35

deuterostomes_taxB_63sol page 42

deuterostomes_taxC_3sol page 57

deuterostomes_taxD_42sol page 63

with_tRNA_crinoids_taxA_1sol page 75

with_tRNA_crinoids_taxB_1sol page 78

with_tRNA_eleutherozoa_taxA_2sol page 81

with_tRNA_ophiurida_taxA_1sol page 84

deuterostomes_taxA_63sol

================================================================================

================================================================================

AXIOMS

================================================================================

================================================================================

{ the solutions of problem PHYLO are the smallest graphs T (defined on the smallest domain possible but containing at least all the OTUs) which verify properties P1 to P6:

P1- T is simple (the relation R(x, y) which defines graph T is not reflexive)

P2- T is non-oriented (the relation R(x, y) which defines graph T is symetrical)

P3- T is connected and acyclic (T is a tree)

P4- T respects the minimal distance matrix, i.e.:

for all couple of OTUs x and y, the length of the path x->y in T is always superior or equals to the minimal distance calculated between x and y (encoded in the minimal distance matrix)

P5- T respects other eventual hypothesis (Primary Phylogenetic Hypothesis = PPH)

used to impose the existence of given monophyletic groups

P6- it is possible to calculate all the values for each HTU in the graph T }

{ OTUs: }

homo_sapiens=0;

asymmetron_inferum=1;

limulus_polyphemus=2; { = outgroup }

xenoturbella=3;

balanoglossus_carnosus=4;

strongylocentrotus_purpuratus=5;

ophiura_lukteni=6;

ophiobolis_aculeata=7;

asterina_pectinifera=8;

florometra_serratissima=9;

gymnocrinus_richeri=10;

antedon_mediterranea=11;

{ PROPERTY P1: R(x, y) is not reflexive}

Q x (-R(x, x));

{ PROPERTY P2: R(x, y) is symetrical}

Q x y (R(x, y) => R(y, x));

{ PROPERTY P3: graph T is connected and acyclic (T is a tree) }

{

This property is verified by a constraint programmed in the model generator, instead of a "heavy" logical formula:

1- it will refuse the partial interpretations in which a connected component of the graph (in construction) is cyclic, i.e. such as: number of edges >= number of vertices

2- it will refuse the complete interpretations in which the constructed graph has more than one connected component

}

{ PROPERTY P4: graph T respects minimal distance matrix }

{

This property is verified by a constraint programmed in the model generator:

it will refuse the partial interpretations in which the graph (in construction) do not respect the minimal distance matrix, i.e. such as:

let x, y a couple of OTUs,

let d= minimal distance calculated between x and y (encoded in the minimal distance matrix), there is a a path of length k between x and y, with: k < d

The minimal distance matrix is encoded directly in the data structure of the model generator:

/* minimal distance matrix DEUTEROSTOMES taxA: */

DIST[0][0]=0;

DIST[1][0]=2; DIST[1][1]=0;

DIST[2][0]=2; DIST[2][1]=4; DIST[2][2]=0;

DIST[3][0]=1; DIST[3][1]=3; DIST[3][2]=3; DIST[3][3]=0;

DIST[4][0]=1; DIST[4][1]=3; DIST[4][2]=2; DIST[4][3]=1; DIST[4][4]=0;

DIST[5][0]=2; DIST[5][1]=4; DIST[5][2]=4; DIST[5][3]=3; DIST[5][4]=3; DIST[5][5]=0;

DIST[6][0]=3; DIST[6][1]=4; DIST[6][2]=4; DIST[6][3]=3; DIST[6][4]=3; DIST[6][5]=2; DIST[6][6]=0;

DIST[7][0]=2; DIST[7][1]=4; DIST[7][2]=4; DIST[7][3]=3; DIST[7][4]=3; DIST[7][5]=2; DIST[7][6]=1; DIST[7][7]=0;

DIST[8][0]=2; DIST[8][1]=4; DIST[8][2]=4; DIST[8][3]=3; DIST[8][4]=3; DIST[8][5]=1; DIST[8][6]=2; DIST[8][7]=1; DIST[8][8]=0;

DIST[9][0]=3; DIST[9][1]=5; DIST[9][2]=4; DIST[9][3]=4; DIST[9][4]=4; DIST[9][5]=1; DIST[9][6]=2; DIST[9][7]=2; DIST[9][8]=1; DIST[9][9]=0;

DIST[10][0]=3; DIST[10][1]=4; DIST[10][2]=4; DIST[10][3]=4; DIST[10][4]=4; DIST[10][5]=2; DIST[10][6]=3; DIST[10][7]=3; DIST[10][8]=2; DIST[10][9]=1; DIST[10][10]=0;

DIST[11][0]=3; DIST[11][1]=5; DIST[11][2]=4; DIST[11][3]=4; DIST[11][4]=4; DIST[11][5]=2; DIST[11][6]=3; DIST[11][7]=3; DIST[11][8]=2; DIST[11][9]=1; DIST[11][10]=1; DIST[11][11]=0;

}

{ PROPERTY P5: graph T respects eventual Primary Phylogenetic Hypotheses }

{

This property is verified by constraints programmed in the model generator:

- monophyly of Deuterostomia = (0,1,3,4,5,6,7,8,9,10,11)

- monophyly of Chordata = (0,1)

- monophyly of Ambulacria = (4,5,6,7,8,9,10,11)

- monophyly of Echinodermata = (5,6,7,8,9,10,11)

- monophyly of Ophiurida = (6,7)

- monophyly of Crinoidea = (9,10,11)

}

{------------------------------------------------------------------------------------------------------------------------}

{ PROPERTY P6: it is possible to calculate all the values for each HTU in the graph T }

{

First we calculate with the model generator the tree solutions which verify properties P1 to P5. Property P6 is verified *a posteriori* for each tree solution, with a *feedback* mechanism:

Studying each tree solution for calculating the values of HTUs, we eventually discover "impossible sub-trees": they appear in tree solutions which verify P1 to P5, but they do not verify P6.

For each impossible subtree A, an additional constraint is programmed into the model generator to forbid the solutions containing A. Tree solutions are recalculated and verified, allowing the discovery of new impossible subtrees and the programming of new constraints to recalculate the solutions (feedback mechanism). Finally, the complete set of optimal solutions is determined after iteration of this process and elimination of all the solutions that do not verify P6.

}

================================================================================

================================================================================

SOLUTIONS

================================================================================

================================================================================

OTUs:

homo_sapiens=0;

asymmetron_inferum=1;

limulus_polyphemus=2;

xenoturbella=3;

balanoglossus_carnosus=4;

strongylocentrotus_purpuratus=5;

ophiura_lukteni=6;

ophiobolis_aculeata=7;

asterina_pectinifera=8;

florometra_serratissima=9;

gymnocrinus_richeri=10;

antedon_mediterranea=11;

HTUs:

n1, n2, n3

D = [0,14]: 63 solutions OK (which verify property P6) (4 impossible sub-trees)

minimal score (best) = 40

maximal score = 88

-------------------------------------------------------------------------------------------------------------

score = 59:

model 1:

-------------

R(0,3) R(0,4) R(0,n1) R(0,n2) R(0,n3) R(1,n1) R(2,n2) R(3,0) R(4,0) R(5,8) R(5,9) R(5,n3) R(6,7) R(7,6) R(7,8) R(8,5) R(8,7) R(9,5) R(9,10) R(9,11) R(10,9) R(11,9) R(n1,0) R(n1,1) R(n2,0) R(n2,2) R(n3,0) R(n3,5)

-------------------------------------------------------------------------------------------------------------

score = 79:

model 2:

-------------

R(0,3) R(0,4) R(0,n1) R(0,n2) R(0,n3) R(1,n1) R(2,n2) R(3,0) R(4,0) R(5,8) R(5,9) R(6,7) R(7,6) R(7,8) R(7,n3) R(8,5) R(8,7) R(9,5) R(9,10) R(9,11) R(10,9) R(11,9) R(n1,0) R(n1,1) R(n2,0) R(n2,2) R(n3,0) R(n3,7)

-------------------------------------------------------------------------------------------------------------

score = 64:

model 3:

-------------

R(0,3) R(0,4) R(0,n1) R(0,n2) R(0,n3) R(1,n1) R(2,n2) R(3,0) R(4,0) R(5,8) R(5,9) R(6,7) R(7,6) R(7,8) R(8,5) R(8,7) R(8,n3) R(9,5) R(9,10) R(9,11) R(10,9) R(11,9) R(n1,0) R(n1,1) R(n2,0) R(n2,2) R(n3,0) R(n3,8)

-------------------------------------------------------------------------------------------------------------

score = 68:

model 4:

-------------

R(0,3) R(0,4) R(0,n1) R(0,n2) R(0,n3) R(1,n1) R(2,n2) R(3,0) R(4,0) R(5,8) R(5,9) R(5,n3) R(6,7) R(7,6) R(7,8) R(8,5) R(8,7) R(9,5) R(9,10) R(10,9) R(10,11) R(11,10) R(n1,0) R(n1,1) R(n2,0) R(n2,2) R(n3,0) R(n3,5)

-------------------------------------------------------------------------------------------------------------

score = 88:

model 5:

-------------

R(0,3) R(0,4) R(0,n1) R(0,n2) R(0,n3) R(1,n1) R(2,n2) R(3,0) R(4,0) R(5,8) R(5,9) R(6,7) R(7,6) R(7,8) R(7,n3) R(8,5) R(8,7) R(9,5) R(9,10) R(10,9) R(10,11) R(11,10) R(n1,0) R(n1,1) R(n2,0) R(n2,2) R(n3,0) R(n3,7)

-------------------------------------------------------------------------------------------------------------

score = 73:

model 6:

-------------

R(0,3) R(0,4) R(0,n1) R(0,n2) R(0,n3) R(1,n1) R(2,n2) R(3,0) R(4,0) R(5,8) R(5,9) R(6,7) R(7,6) R(7,8) R(8,5) R(8,7) R(8,n3) R(9,5) R(9,10) R(10,9) R(10,11) R(11,10) R(n1,0) R(n1,1) R(n2,0) R(n2,2) R(n3,0) R(n3,8)

-------------------------------------------------------------------------------------------------------------

score = 68:

model 7:

-------------

R(0,3) R(0,4) R(0,n1) R(0,n2) R(0,n3) R(1,n1) R(2,n2) R(3,0) R(4,0) R(5,8) R(5,9) R(5,n3) R(6,7) R(7,6) R(7,8) R(8,5) R(8,7) R(9,5) R(9,11) R(10,11) R(11,9) R(11,10) R(n1,0) R(n1,1) R(n2,0) R(n2,2) R(n3,0) R(n3,5)

-------------------------------------------------------------------------------------------------------------

score = 88:

model 8:

-------------

R(0,3) R(0,4) R(0,n1) R(0,n2) R(0,n3) R(1,n1) R(2,n2) R(3,0) R(4,0) R(5,8) R(5,9) R(6,7) R(7,6) R(7,8) R(7,n3) R(8,5) R(8,7) R(9,5) R(9,11) R(10,11) R(11,9) R(11,10) R(n1,0) R(n1,1) R(n2,0) R(n2,2) R(n3,0) R(n3,7)

-------------------------------------------------------------------------------------------------------------

score = 73:

model 9:

-------------

R(0,3) R(0,4) R(0,n1) R(0,n2) R(0,n3) R(1,n1) R(2,n2) R(3,0) R(4,0) R(5,8) R(5,9) R(6,7) R(7,6) R(7,8) R(8,5) R(8,7) R(8,n3) R(9,5) R(9,11) R(10,11) R(11,9) R(11,10) R(n1,0) R(n1,1) R(n2,0) R(n2,2) R(n3,0) R(n3,8)

-------------------------------------------------------------------------------------------------------------

n3(mod10)

[ cox1 nad4L cox2 atp8 atp6 cox3 nad3 nad4 nad5 -nad6 cob rrnS rrnL nad1 nad2 ]

score = 43:

model 10:

-------------

R(0,3) R(0,4) R(0,n1) R(0,n2) R(0,n3) R(1,n1) R(2,n2) R(3,0) R(4,0) R(5,8) R(5,9) R(5,n3) R(6,7) R(7,6) R(7,n3) R(8,5) R(9,5) R(9,10) R(9,11) R(10,9) R(11,9) R(n1,0) R(n1,1) R(n2,0) R(n2,2) R(n3,0) R(n3,5) R(n3,7)

-------------------------------------------------------------------------------------------------------------

n3(mod11)

[ cox1 nad4L cox2 atp8 atp6 cox3 nad3 nad4 nad5 -nad6 cob rrnS rrnL nad1 nad2 ]

score = 64:

model 11:

-------------

R(0,3) R(0,4) R(0,n1) R(0,n2) R(0,n3) R(1,n1) R(2,n2) R(3,0) R(4,0) R(5,8) R(5,9) R(6,7) R(7,6) R(7,n3) R(8,5) R(8,n3) R(9,5) R(9,10) R(9,11) R(10,9) R(11,9) R(n1,0) R(n1,1) R(n2,0) R(n2,2) R(n3,0) R(n3,7) R(n3,8)

-------------------------------------------------------------------------------------------------------------

n3(mod12)

[ cox1 nad4L cox2 atp8 atp6 cox3 nad3 nad4 nad5 -nad6 cob rrnS rrnL nad1 nad2 ]

score = 52:

model 12:

-------------

R(0,3) R(0,4) R(0,n1) R(0,n2) R(0,n3) R(1,n1) R(2,n2) R(3,0) R(4,0) R(5,8) R(5,9) R(5,n3) R(6,7) R(7,6) R(7,n3) R(8,5) R(9,5) R(9,10) R(10,9) R(10,11) R(11,10) R(n1,0) R(n1,1) R(n2,0) R(n2,2) R(n3,0) R(n3,5) R(n3,7)

-------------------------------------------------------------------------------------------------------------

n3(mod13)

[ cox1 nad4L cox2 atp8 atp6 cox3 nad3 nad4 nad5 -nad6 cob rrnS rrnL nad1 nad2 ]

score = 73:

model 13:

-------------

R(0,3) R(0,4) R(0,n1) R(0,n2) R(0,n3) R(1,n1) R(2,n2) R(3,0) R(4,0) R(5,8) R(5,9) R(6,7) R(7,6) R(7,n3) R(8,5) R(8,n3) R(9,5) R(9,10) R(10,9) R(10,11) R(11,10) R(n1,0) R(n1,1) R(n2,0) R(n2,2) R(n3,0) R(n3,7) R(n3,8)

-------------------------------------------------------------------------------------------------------------

n3(mod14)

[ cox1 nad4L cox2 atp8 atp6 cox3 nad3 nad4 nad5 -nad6 cob rrnS rrnL nad1 nad2 ]

score = 52:

model 14:

-------------

R(0,3) R(0,4) R(0,n1) R(0,n2) R(0,n3) R(1,n1) R(2,n2) R(3,0) R(4,0) R(5,8) R(5,9) R(5,n3) R(6,7) R(7,6) R(7,n3) R(8,5) R(9,5) R(9,11) R(10,11) R(11,9) R(11,10) R(n1,0) R(n1,1) R(n2,0) R(n2,2) R(n3,0) R(n3,5) R(n3,7)

-------------------------------------------------------------------------------------------------------------

n3(mod15)

[ cox1 nad4L cox2 atp8 atp6 cox3 nad3 nad4 nad5 -nad6 cob rrnS rrnL nad1 nad2 ]

score = 73:

model 15:

-------------

R(0,3) R(0,4) R(0,n1) R(0,n2) R(0,n3) R(1,n1) R(2,n2) R(3,0) R(4,0) R(5,8) R(5,9) R(6,7) R(7,6) R(7,n3) R(8,5) R(8,n3) R(9,5) R(9,11) R(10,11) R(11,9) R(11,10) R(n1,0) R(n1,1) R(n2,0) R(n2,2) R(n3,0) R(n3,7) R(n3,8)

-------------------------------------------------------------------------------------------------------------

score = 68:

model 16:

-------------

R(0,3) R(0,4) R(0,n1) R(0,n2) R(0,n3) R(1,n1) R(2,n2) R(3,0) R(4,0) R(5,8) R(5,n3) R(6,7) R(7,6) R(7,8) R(8,5) R(8,7) R(8,9) R(9,8) R(9,10) R(9,11) R(10,9) R(11,9) R(n1,0) R(n1,1) R(n2,0) R(n2,2) R(n3,0) R(n3,5)

-------------------------------------------------------------------------------------------------------------

score = 58:

model 17:

-------------

R(0,3) R(0,4) R(0,n1) R(0,n2) R(0,n3) R(1,n1) R(2,n2) R(3,0) R(4,0) R(5,8) R(6,7) R(7,6) R(7,8) R(7,n3) R(8,5) R(8,7) R(8,9) R(9,8) R(9,10) R(9,11) R(10,9) R(11,9) R(n1,0) R(n1,1) R(n2,0) R(n2,2) R(n3,0) R(n3,7)

-------------------------------------------------------------------------------------------------------------

score = 43:

model 18:

-------------

R(0,3) R(0,4) R(0,n1) R(0,n2) R(0,n3) R(1,n1) R(2,n2) R(3,0) R(4,0) R(5,8) R(6,7) R(7,6) R(7,8) R(8,5) R(8,7) R(8,9) R(8,n3) R(9,8) R(9,10) R(9,11) R(10,9) R(11,9) R(n1,0) R(n1,1) R(n2,0) R(n2,2) R(n3,0) R(n3,8)

-------------------------------------------------------------------------------------------------------------

score = 77:

model 19:

-------------

R(0,3) R(0,4) R(0,n1) R(0,n2) R(0,n3) R(1,n1) R(2,n2) R(3,0) R(4,0) R(5,8) R(5,n3) R(6,7) R(7,6) R(7,8) R(8,5) R(8,7) R(8,9) R(9,8) R(9,10) R(10,9) R(10,11) R(11,10) R(n1,0) R(n1,1) R(n2,0) R(n2,2) R(n3,0) R(n3,5)

-------------------------------------------------------------------------------------------------------------

score = 67:

model 20:

-------------

R(0,3) R(0,4) R(0,n1) R(0,n2) R(0,n3) R(1,n1) R(2,n2) R(3,0) R(4,0) R(5,8) R(6,7) R(7,6) R(7,8) R(7,n3) R(8,5) R(8,7) R(8,9) R(9,8) R(9,10) R(10,9) R(10,11) R(11,10) R(n1,0) R(n1,1) R(n2,0) R(n2,2) R(n3,0) R(n3,7)

-------------------------------------------------------------------------------------------------------------

score = 52:

model 21:

-------------

R(0,3) R(0,4) R(0,n1) R(0,n2) R(0,n3) R(1,n1) R(2,n2) R(3,0) R(4,0) R(5,8) R(6,7) R(7,6) R(7,8) R(8,5) R(8,7) R(8,9) R(8,n3) R(9,8) R(9,10) R(10,9) R(10,11) R(11,10) R(n1,0) R(n1,1) R(n2,0) R(n2,2) R(n3,0) R(n3,8)

-------------------------------------------------------------------------------------------------------------

score = 77:

model 22:

-------------

R(0,3) R(0,4) R(0,n1) R(0,n2) R(0,n3) R(1,n1) R(2,n2) R(3,0) R(4,0) R(5,8) R(5,n3) R(6,7) R(7,6) R(7,8) R(8,5) R(8,7) R(8,9) R(9,8) R(9,11) R(10,11) R(11,9) R(11,10) R(n1,0) R(n1,1) R(n2,0) R(n2,2) R(n3,0) R(n3,5)

-------------------------------------------------------------------------------------------------------------

score = 67:

model 23:

-------------

R(0,3) R(0,4) R(0,n1) R(0,n2) R(0,n3) R(1,n1) R(2,n2) R(3,0) R(4,0) R(5,8) R(6,7) R(7,6) R(7,8) R(7,n3) R(8,5) R(8,7) R(8,9) R(9,8) R(9,11) R(10,11) R(11,9) R(11,10) R(n1,0) R(n1,1) R(n2,0) R(n2,2) R(n3,0) R(n3,7)

-------------------------------------------------------------------------------------------------------------

score = 52:

model 24:

-------------

R(0,3) R(0,4) R(0,n1) R(0,n2) R(0,n3) R(1,n1) R(2,n2) R(3,0) R(4,0) R(5,8) R(6,7) R(7,6) R(7,8) R(8,5) R(8,7) R(8,9) R(8,n3) R(9,8) R(9,11) R(10,11) R(11,9) R(11,10) R(n1,0) R(n1,1) R(n2,0) R(n2,2) R(n3,0) R(n3,8)

-------------------------------------------------------------------------------------------------------------

n3(mod25)

[ cox1 nad4L cox2 atp8 atp6 cox3 nad3 nad4 nad5 -nad6 cob rrnS rrnL nad1 nad2 ]

score = 64:

model 25:

-------------

R(0,3) R(0,4) R(0,n1) R(0,n2) R(0,n3) R(1,n1) R(2,n2) R(3,0) R(4,0) R(5,8) R(5,n3) R(6,7) R(7,6) R(7,n3) R(8,5) R(8,9) R(9,8) R(9,10) R(9,11) R(10,9) R(11,9) R(n1,0) R(n1,1) R(n2,0) R(n2,2) R(n3,0) R(n3,5) R(n3,7)

-------------------------------------------------------------------------------------------------------------

n3(mod26)

[ cox1 nad4L cox2 atp8 atp6 cox3 nad3 nad4 nad5 -nad6 cob rrnS rrnL nad1 nad2 ]

score = 43:

model 26:

-------------

R(0,3) R(0,4) R(0,n1) R(0,n2) R(0,n3) R(1,n1) R(2,n2) R(3,0) R(4,0) R(5,8) R(6,7) R(7,6) R(7,n3) R(8,5) R(8,9) R(8,n3) R(9,8) R(9,10) R(9,11) R(10,9) R(11,9) R(n1,0) R(n1,1) R(n2,0) R(n2,2) R(n3,0) R(n3,7) R(n3,8)

-------------------------------------------------------------------------------------------------------------

n3(mod27)

[ cox1 nad4L cox2 atp8 atp6 cox3 nad3 nad4 nad5 -nad6 cob rrnS rrnL nad1 nad2 ]

score = 73:

model 27:

-------------

R(0,3) R(0,4) R(0,n1) R(0,n2) R(0,n3) R(1,n1) R(2,n2) R(3,0) R(4,0) R(5,8) R(5,n3) R(6,7) R(7,6) R(7,n3) R(8,5) R(8,9) R(9,8) R(9,10) R(10,9) R(10,11) R(11,10) R(n1,0) R(n1,1) R(n2,0) R(n2,2) R(n3,0) R(n3,5) R(n3,7)

-------------------------------------------------------------------------------------------------------------

n3(mod28)

[ cox1 nad4L cox2 atp8 atp6 cox3 nad3 nad4 nad5 -nad6 cob rrnS rrnL nad1 nad2 ]

score = 52:

model 28:

-------------

R(0,3) R(0,4) R(0,n1) R(0,n2) R(0,n3) R(1,n1) R(2,n2) R(3,0) R(4,0) R(5,8) R(6,7) R(7,6) R(7,n3) R(8,5) R(8,9) R(8,n3) R(9,8) R(9,10) R(10,9) R(10,11) R(11,10) R(n1,0) R(n1,1) R(n2,0) R(n2,2) R(n3,0) R(n3,7) R(n3,8)

-------------------------------------------------------------------------------------------------------------

n3(mod29)

[ cox1 nad4L cox2 atp8 atp6 cox3 nad3 nad4 nad5 -nad6 cob rrnS rrnL nad1 nad2 ]

score = 73:

model 29:

-------------

R(0,3) R(0,4) R(0,n1) R(0,n2) R(0,n3) R(1,n1) R(2,n2) R(3,0) R(4,0) R(5,8) R(5,n3) R(6,7) R(7,6) R(7,n3) R(8,5) R(8,9) R(9,8) R(9,11) R(10,11) R(11,9) R(11,10) R(n1,0) R(n1,1) R(n2,0) R(n2,2) R(n3,0) R(n3,5) R(n3,7)

-------------------------------------------------------------------------------------------------------------

n3(mod30)

[ cox1 nad4L cox2 atp8 atp6 cox3 nad3 nad4 nad5 -nad6 cob rrnS rrnL nad1 nad2 ]

score = 52:

model 30:

-------------

R(0,3) R(0,4) R(0,n1) R(0,n2) R(0,n3) R(1,n1) R(2,n2) R(3,0) R(4,0) R(5,8) R(6,7) R(7,6) R(7,n3) R(8,5) R(8,9) R(8,n3) R(9,8) R(9,11) R(10,11) R(11,9) R(11,10) R(n1,0) R(n1,1) R(n2,0) R(n2,2) R(n3,0) R(n3,7) R(n3,8)

-------------------------------------------------------------------------------------------------------------

score = 68:

model 31:

-------------

R(0,3) R(0,4) R(0,n1) R(0,n2) R(0,n3) R(1,n1) R(2,n2) R(3,0) R(4,0) R(5,9) R(5,n3) R(6,7) R(7,6) R(7,8) R(8,7) R(8,9) R(9,5) R(9,8) R(9,10) R(9,11) R(10,9) R(11,9) R(n1,0) R(n1,1) R(n2,0) R(n2,2) R(n3,0) R(n3,5)

-------------------------------------------------------------------------------------------------------------

score = 63:

model 32:

-------------

R(0,3) R(0,4) R(0,n1) R(0,n2) R(0,n3) R(1,n1) R(2,n2) R(3,0) R(4,0) R(5,9) R(6,7) R(7,6) R(7,8) R(7,n3) R(8,7) R(8,9) R(9,5) R(9,8) R(9,10) R(9,11) R(10,9) R(11,9) R(n1,0) R(n1,1) R(n2,0) R(n2,2) R(n3,0) R(n3,7)

-------------------------------------------------------------------------------------------------------------

score = 48:

model 33:

-------------

R(0,3) R(0,4) R(0,n1) R(0,n2) R(0,n3) R(1,n1) R(2,n2) R(3,0) R(4,0) R(5,9) R(6,7) R(7,6) R(7,8) R(8,7) R(8,9) R(8,n3) R(9,5) R(9,8) R(9,10) R(9,11) R(10,9) R(11,9) R(n1,0) R(n1,1) R(n2,0) R(n2,2) R(n3,0) R(n3,8)

-------------------------------------------------------------------------------------------------------------

score = 77:

model 34:

-------------

R(0,3) R(0,4) R(0,n1) R(0,n2) R(0,n3) R(1,n1) R(2,n2) R(3,0) R(4,0) R(5,9) R(5,n3) R(6,7) R(7,6) R(7,8) R(8,7) R(8,9) R(9,5) R(9,8) R(9,10) R(10,9) R(10,11) R(11,10) R(n1,0) R(n1,1) R(n2,0) R(n2,2) R(n3,0) R(n3,5)

-------------------------------------------------------------------------------------------------------------

score = 72:

model 35:

-------------

R(0,3) R(0,4) R(0,n1) R(0,n2) R(0,n3) R(1,n1) R(2,n2) R(3,0) R(4,0) R(5,9) R(6,7) R(7,6) R(7,8) R(7,n3) R(8,7) R(8,9) R(9,5) R(9,8) R(9,10) R(10,9) R(10,11) R(11,10) R(n1,0) R(n1,1) R(n2,0) R(n2,2) R(n3,0) R(n3,7)

-------------------------------------------------------------------------------------------------------------

score = 57:

model 36:

-------------

R(0,3) R(0,4) R(0,n1) R(0,n2) R(0,n3) R(1,n1) R(2,n2) R(3,0) R(4,0) R(5,9) R(6,7) R(7,6) R(7,8) R(8,7) R(8,9) R(8,n3) R(9,5) R(9,8) R(9,10) R(10,9) R(10,11) R(11,10) R(n1,0) R(n1,1) R(n2,0) R(n2,2) R(n3,0) R(n3,8)

-------------------------------------------------------------------------------------------------------------

score = 77:

model 37:

-------------

R(0,3) R(0,4) R(0,n1) R(0,n2) R(0,n3) R(1,n1) R(2,n2) R(3,0) R(4,0) R(5,9) R(5,n3) R(6,7) R(7,6) R(7,8) R(8,7) R(8,9) R(9,5) R(9,8) R(9,11) R(10,11) R(11,9) R(11,10) R(n1,0) R(n1,1) R(n2,0) R(n2,2) R(n3,0) R(n3,5)

-------------------------------------------------------------------------------------------------------------

score = 72:

model 38:

-------------

R(0,3) R(0,4) R(0,n1) R(0,n2) R(0,n3) R(1,n1) R(2,n2) R(3,0) R(4,0) R(5,9) R(6,7) R(7,6) R(7,8) R(7,n3) R(8,7) R(8,9) R(9,5) R(9,8) R(9,11) R(10,11) R(11,9) R(11,10) R(n1,0) R(n1,1) R(n2,0) R(n2,2) R(n3,0) R(n3,7)

-------------------------------------------------------------------------------------------------------------

score = 57:

model 39:

-------------

R(0,3) R(0,4) R(0,n1) R(0,n2) R(0,n3) R(1,n1) R(2,n2) R(3,0) R(4,0) R(5,9) R(6,7) R(7,6) R(7,8) R(8,7) R(8,9) R(8,n3) R(9,5) R(9,8) R(9,11) R(10,11) R(11,9) R(11,10) R(n1,0) R(n1,1) R(n2,0) R(n2,2) R(n3,0) R(n3,8)

-------------------------------------------------------------------------------------------------------------

n3(mod40)

[ cox1 nad4L cox2 atp8 atp6 cox3 nad3 nad4 nad5 -nad6 cob rrnS rrnL nad1 nad2 ]

score = 47:

model 40:

-------------

R(0,3) R(0,4) R(0,n1) R(0,n2) R(0,n3) R(1,n1) R(2,n2) R(3,0) R(4,0) R(5,9) R(5,n3) R(6,7) R(7,6) R(7,8) R(7,n3) R(8,7) R(9,5) R(9,10) R(9,11) R(10,9) R(11,9) R(n1,0) R(n1,1) R(n2,0) R(n2,2) R(n3,0) R(n3,5) R(n3,7)

-------------------------------------------------------------------------------------------------------------

n3(mod41)

[ cox1 nad4L cox2 atp8 atp6 cox3 nad3 nad4 nad5 -nad6 cob rrnS rrnL nad1 nad2 ]

score = 56:

model 41:

-------------

R(0,3) R(0,4) R(0,n1) R(0,n2) R(0,n3) R(1,n1) R(2,n2) R(3,0) R(4,0) R(5,9) R(5,n3) R(6,7) R(7,6) R(7,8) R(8,7) R(8,n3) R(9,5) R(9,10) R(9,11) R(10,9) R(11,9) R(n1,0) R(n1,1) R(n2,0) R(n2,2) R(n3,0) R(n3,5) R(n3,8)

-------------------------------------------------------------------------------------------------------------

n3(mod42)

[ cox1 nad4L cox2 atp8 atp6 cox3 nad3 nad4 nad5 -nad6 cob rrnS rrnL nad1 nad2 ]

score = 56:

model 42:

-------------

R(0,3) R(0,4) R(0,n1) R(0,n2) R(0,n3) R(1,n1) R(2,n2) R(3,0) R(4,0) R(5,9) R(5,n3) R(6,7) R(7,6) R(7,8) R(7,n3) R(8,7) R(9,5) R(9,10) R(10,9) R(10,11) R(11,10) R(n1,0) R(n1,1) R(n2,0) R(n2,2) R(n3,0) R(n3,5) R(n3,7)

-------------------------------------------------------------------------------------------------------------

n3(mod43)

[ cox1 nad4L cox2 atp8 atp6 cox3 nad3 nad4 nad5 -nad6 cob rrnS rrnL nad1 nad2 ]

score = 65:

model 43:

-------------

R(0,3) R(0,4) R(0,n1) R(0,n2) R(0,n3) R(1,n1) R(2,n2) R(3,0) R(4,0) R(5,9) R(5,n3) R(6,7) R(7,6) R(7,8) R(8,7) R(8,n3) R(9,5) R(9,10) R(10,9) R(10,11) R(11,10) R(n1,0) R(n1,1) R(n2,0) R(n2,2) R(n3,0) R(n3,5) R(n3,8)

-------------------------------------------------------------------------------------------------------------

n3(mod44)

[ cox1 nad4L cox2 atp8 atp6 cox3 nad3 nad4 nad5 -nad6 cob rrnS rrnL nad1 nad2 ]

score = 56:

model 44:

-------------

R(0,3) R(0,4) R(0,n1) R(0,n2) R(0,n3) R(1,n1) R(2,n2) R(3,0) R(4,0) R(5,9) R(5,n3) R(6,7) R(7,6) R(7,8) R(7,n3) R(8,7) R(9,5) R(9,11) R(10,11) R(11,9) R(11,10) R(n1,0) R(n1,1) R(n2,0) R(n2,2) R(n3,0) R(n3,5) R(n3,7)

-------------------------------------------------------------------------------------------------------------

n3(mod45)

[ cox1 nad4L cox2 atp8 atp6 cox3 nad3 nad4 nad5 -nad6 cob rrnS rrnL nad1 nad2 ]

score = 65:

model 45:

-------------

R(0,3) R(0,4) R(0,n1) R(0,n2) R(0,n3) R(1,n1) R(2,n2) R(3,0) R(4,0) R(5,9) R(5,n3) R(6,7) R(7,6) R(7,8) R(8,7) R(8,n3) R(9,5) R(9,11) R(10,11) R(11,9) R(11,10) R(n1,0) R(n1,1) R(n2,0) R(n2,2) R(n3,0) R(n3,5) R(n3,8)

-------------------------------------------------------------------------------------------------------------

n3(mod46)

[ cox1 nad4L cox2 atp8 atp6 cox3 nad3 nad4 nad5 -nad6 cob rrnS rrnL nad1 nad2 ]

score = 48:

model 46:

-------------

R(0,3) R(0,4) R(0,n1) R(0,n2) R(0,n3) R(1,n1) R(2,n2) R(3,0) R(4,0) R(5,9) R(5,n3) R(6,7) R(7,6) R(7,n3) R(8,9) R(9,5) R(9,8) R(9,10) R(9,11) R(10,9) R(11,9) R(n1,0) R(n1,1) R(n2,0) R(n2,2) R(n3,0) R(n3,5) R(n3,7)

-------------------------------------------------------------------------------------------------------------

n3(mod47)

[ cox1 nad4L cox2 atp8 atp6 cox3 nad3 nad4 nad5 -nad6 cob rrnS rrnL nad1 nad2 ]

score = 48:

model 47:

-------------

R(0,3) R(0,4) R(0,n1) R(0,n2) R(0,n3) R(1,n1) R(2,n2) R(3,0) R(4,0) R(5,9) R(6,7) R(7,6) R(7,n3) R(8,9) R(8,n3) R(9,5) R(9,8) R(9,10) R(9,11) R(10,9) R(11,9) R(n1,0) R(n1,1) R(n2,0) R(n2,2) R(n3,0) R(n3,7) R(n3,8)

-------------------------------------------------------------------------------------------------------------

n3(mod48)

[ cox1 nad4L cox2 atp8 atp6 cox3 nad3 nad4 nad5 -nad6 cob rrnS rrnL nad1 nad2 ]

score = 57:

model 48:

-------------

R(0,3) R(0,4) R(0,n1) R(0,n2) R(0,n3) R(1,n1) R(2,n2) R(3,0) R(4,0) R(5,9) R(5,n3) R(6,7) R(7,6) R(7,n3) R(8,9) R(9,5) R(9,8) R(9,10) R(10,9) R(10,11) R(11,10) R(n1,0) R(n1,1) R(n2,0) R(n2,2) R(n3,0) R(n3,5) R(n3,7)

-------------------------------------------------------------------------------------------------------------

n3(mod49)

[ cox1 nad4L cox2 atp8 atp6 cox3 nad3 nad4 nad5 -nad6 cob rrnS rrnL nad1 nad2 ]

score = 57:

model 49:

-------------

R(0,3) R(0,4) R(0,n1) R(0,n2) R(0,n3) R(1,n1) R(2,n2) R(3,0) R(4,0) R(5,9) R(6,7) R(7,6) R(7,n3) R(8,9) R(8,n3) R(9,5) R(9,8) R(9,10) R(10,9) R(10,11) R(11,10) R(n1,0) R(n1,1) R(n2,0) R(n2,2) R(n3,0) R(n3,7) R(n3,8)

-------------------------------------------------------------------------------------------------------------

n3(mod50)

[ cox1 nad4L cox2 atp8 atp6 cox3 nad3 nad4 nad5 -nad6 cob rrnS rrnL nad1 nad2 ]

score = 57:

model 50:

-------------

R(0,3) R(0,4) R(0,n1) R(0,n2) R(0,n3) R(1,n1) R(2,n2) R(3,0) R(4,0) R(5,9) R(5,n3) R(6,7) R(7,6) R(7,n3) R(8,9) R(9,5) R(9,8) R(9,11) R(10,11) R(11,9) R(11,10) R(n1,0) R(n1,1) R(n2,0) R(n2,2) R(n3,0) R(n3,5) R(n3,7)

-------------------------------------------------------------------------------------------------------------

n3(mod51)

[ cox1 nad4L cox2 atp8 atp6 cox3 nad3 nad4 nad5 -nad6 cob rrnS rrnL nad1 nad2 ]

score = 57:

model 51:

-------------

R(0,3) R(0,4) R(0,n1) R(0,n2) R(0,n3) R(1,n1) R(2,n2) R(3,0) R(4,0) R(5,9) R(6,7) R(7,6) R(7,n3) R(8,9) R(8,n3) R(9,5) R(9,8) R(9,11) R(10,11) R(11,9) R(11,10) R(n1,0) R(n1,1) R(n2,0) R(n2,2) R(n3,0) R(n3,7) R(n3,8)

-------------------------------------------------------------------------------------------------------------

n3(mod52)

[ cox1 nad4L cox2 atp8 atp6 cox3 nad3 nad4 nad5 -nad6 cob rrnS rrnL nad1 nad2 ]

score = 40:

model 52:

-------------

R(0,3) R(0,4) R(0,n1) R(0,n2) R(0,n3) R(1,n1) R(2,n2) R(3,0) R(4,0) R(5,9) R(5,n3) R(6,7) R(7,6) R(7,n3) R(8,n3) R(9,5) R(9,10) R(9,11) R(10,9) R(11,9) R(n1,0) R(n1,1) R(n2,0) R(n2,2) R(n3,0) R(n3,5) R(n3,7) R(n3,8)

-------------------------------------------------------------------------------------------------------------

n3(mod53)

[ cox1 nad4L cox2 atp8 atp6 cox3 nad3 nad4 nad5 -nad6 cob rrnS rrnL nad1 nad2 ]

score = 49:

model 53:

-------------

R(0,3) R(0,4) R(0,n1) R(0,n2) R(0,n3) R(1,n1) R(2,n2) R(3,0) R(4,0) R(5,9) R(5,n3) R(6,7) R(7,6) R(7,n3) R(8,n3) R(9,5) R(9,10) R(10,9) R(10,11) R(11,10) R(n1,0) R(n1,1) R(n2,0) R(n2,2) R(n3,0) R(n3,5) R(n3,7) R(n3,8)

-------------------------------------------------------------------------------------------------------------

n3(mod54)

[ cox1 nad4L cox2 atp8 atp6 cox3 nad3 nad4 nad5 -nad6 cob rrnS rrnL nad1 nad2 ]

score = 49:

model 54:

-------------

R(0,3) R(0,4) R(0,n1) R(0,n2) R(0,n3) R(1,n1) R(2,n2) R(3,0) R(4,0) R(5,9) R(5,n3) R(6,7) R(7,6) R(7,n3) R(8,n3) R(9,5) R(9,11) R(10,11) R(11,9) R(11,10) R(n1,0) R(n1,1) R(n2,0) R(n2,2) R(n3,0) R(n3,5) R(n3,7) R(n3,8)

-------------------------------------------------------------------------------------------------------------

n3(mod55)

[ cox1 nad4L cox2 atp8 atp6 cox3 nad3 nad4 nad5 -nad6 cob rrnS rrnL nad1 nad2 ]

score = 56:

model 55:

-------------

R(0,3) R(0,4) R(0,n1) R(0,n2) R(0,n3) R(1,n1) R(2,n2) R(3,0) R(4,0) R(5,n3) R(6,7) R(7,6) R(7,8) R(7,n3) R(8,7) R(8,9) R(9,8) R(9,10) R(9,11) R(10,9) R(11,9) R(n1,0) R(n1,1) R(n2,0) R(n2,2) R(n3,0) R(n3,5) R(n3,7)

-------------------------------------------------------------------------------------------------------------

n3(mod56)

[ cox1 nad4L cox2 atp8 atp6 cox3 nad3 nad4 nad5 -nad6 cob rrnS rrnL nad1 nad2 ]

score = 44:

model 56:

-------------

R(0,3) R(0,4) R(0,n1) R(0,n2) R(0,n3) R(1,n1) R(2,n2) R(3,0) R(4,0) R(5,n3) R(6,7) R(7,6) R(7,8) R(8,7) R(8,9) R(8,n3) R(9,8) R(9,10) R(9,11) R(10,9) R(11,9) R(n1,0) R(n1,1) R(n2,0) R(n2,2) R(n3,0) R(n3,5) R(n3,8)

-------------------------------------------------------------------------------------------------------------

n3(mod57)

[ cox1 nad4L cox2 atp8 atp6 cox3 nad3 nad4 nad5 -nad6 cob rrnS rrnL nad1 nad2 ]

score = 65:

model 57:

-------------

R(0,3) R(0,4) R(0,n1) R(0,n2) R(0,n3) R(1,n1) R(2,n2) R(3,0) R(4,0) R(5,n3) R(6,7) R(7,6) R(7,8) R(7,n3) R(8,7) R(8,9) R(9,8) R(9,10) R(10,9) R(10,11) R(11,10) R(n1,0) R(n1,1) R(n2,0) R(n2,2) R(n3,0) R(n3,5) R(n3,7)

-------------------------------------------------------------------------------------------------------------

n3(mod58)

[ cox1 nad4L cox2 atp8 atp6 cox3 nad3 nad4 nad5 -nad6 cob rrnS rrnL nad1 nad2 ]

score = 53:

model 58:

-------------

R(0,3) R(0,4) R(0,n1) R(0,n2) R(0,n3) R(1,n1) R(2,n2) R(3,0) R(4,0) R(5,n3) R(6,7) R(7,6) R(7,8) R(8,7) R(8,9) R(8,n3) R(9,8) R(9,10) R(10,9) R(10,11) R(11,10) R(n1,0) R(n1,1) R(n2,0) R(n2,2) R(n3,0) R(n3,5) R(n3,8)

-------------------------------------------------------------------------------------------------------------

n3(mod59)

[ cox1 nad4L cox2 atp8 atp6 cox3 nad3 nad4 nad5 -nad6 cob rrnS rrnL nad1 nad2 ]

score = 65:

model 59:

-------------

R(0,3) R(0,4) R(0,n1) R(0,n2) R(0,n3) R(1,n1) R(2,n2) R(3,0) R(4,0) R(5,n3) R(6,7) R(7,6) R(7,8) R(7,n3) R(8,7) R(8,9) R(9,8) R(9,11) R(10,11) R(11,9) R(11,10) R(n1,0) R(n1,1) R(n2,0) R(n2,2) R(n3,0) R(n3,5) R(n3,7)

-------------------------------------------------------------------------------------------------------------

n3(mod60)

[ cox1 nad4L cox2 atp8 atp6 cox3 nad3 nad4 nad5 -nad6 cob rrnS rrnL nad1 nad2 ]

score = 53:

model 60:

-------------

R(0,3) R(0,4) R(0,n1) R(0,n2) R(0,n3) R(1,n1) R(2,n2) R(3,0) R(4,0) R(5,n3) R(6,7) R(7,6) R(7,8) R(8,7) R(8,9) R(8,n3) R(9,8) R(9,11) R(10,11) R(11,9) R(11,10) R(n1,0) R(n1,1) R(n2,0) R(n2,2) R(n3,0) R(n3,5) R(n3,8)

-------------------------------------------------------------------------------------------------------------

n3(mod61)

[ cox1 nad4L cox2 atp8 atp6 cox3 nad3 nad4 nad5 -nad6 cob rrnS rrnL nad1 nad2 ]

score = 40:

model 61:

-------------

R(0,3) R(0,4) R(0,n1) R(0,n2) R(0,n3) R(1,n1) R(2,n2) R(3,0) R(4,0) R(5,n3) R(6,7) R(7,6) R(7,n3) R(8,9) R(8,n3) R(9,8) R(9,10) R(9,11) R(10,9) R(11,9) R(n1,0) R(n1,1) R(n2,0) R(n2,2) R(n3,0) R(n3,5) R(n3,7) R(n3,8)

-------------------------------------------------------------------------------------------------------------

n3(mod62)

[ cox1 nad4L cox2 atp8 atp6 cox3 nad3 nad4 nad5 -nad6 cob rrnS rrnL nad1 nad2 ]

score = 49:

model 62:

-------------

R(0,3) R(0,4) R(0,n1) R(0,n2) R(0,n3) R(1,n1) R(2,n2) R(3,0) R(4,0) R(5,n3) R(6,7) R(7,6) R(7,n3) R(8,9) R(8,n3) R(9,8) R(9,10) R(10,9) R(10,11) R(11,10) R(n1,0) R(n1,1) R(n2,0) R(n2,2) R(n3,0) R(n3,5) R(n3,7) R(n3,8)

-------------------------------------------------------------------------------------------------------------

n3(mod63)

[ cox1 nad4L cox2 atp8 atp6 cox3 nad3 nad4 nad5 -nad6 cob rrnS rrnL nad1 nad2 ]

score = 49:

model 63:

-------------

R(0,3) R(0,4) R(0,n1) R(0,n2) R(0,n3) R(1,n1) R(2,n2) R(3,0) R(4,0) R(5,n3) R(6,7) R(7,6) R(7,n3) R(8,9) R(8,n3) R(9,8) R(9,11) R(10,11) R(11,9) R(11,10) R(n1,0) R(n1,1) R(n2,0) R(n2,2) R(n3,0) R(n3,5) R(n3,7) R(n3,8)

-------------------------------------------------------------------------------------------------------------

-> no other models

deuterostomes_taxA_v2_6sol

================================================================================

================================================================================

AXIOMS

================================================================================

================================================================================

{ the solutions of problem PHYLO are the smallest graphs T (defined on the smallest domain possible but containing at least all the OTUs) which verify properties P1 to P6:

P1- T is simple (the relation R(x, y) which defines graph T is not reflexive)

P2- T is non-oriented (the relation R(x, y) which defines graph T is symetrical)

P3- T is connected and acyclic (T is a tree)

P4- T respects the minimal distance matrix, i.e.:

for all couple of OTUs x and y, the length of the path x->y in T is always superior or equals to the minimal distance calculated between x and y (encoded in the minimal distance matrix)

P5- T respects other eventual hypothesis (Primary Phylogenetic Hypothesis = PPH)

used to impose the existence of given monophyletic groups

P6- it is possible to calculate all the values for each HTU in the graph T }

{ OTUs: }

homo_sapiens=0;

asymmetron_inferum=1;

limulus_polyphemus=2; { = outgroup }

xenoturbella=3;

balanoglossus_carnosus=4;

strongylocentrotus_purpuratus=5;

ophiura_lukteni=6;

ophiobolis_aculeata=7;

asterina_pectinifera=8;

florometra_serratissima=9;

gymnocrinus_richeri=10;

antedon_mediterranea=11;

{ PROPERTY P1: R(x, y) is not reflexive}

Q x (-R(x, x));

{ PROPERTY P2: R(x, y) is symetrical}

Q x y (R(x, y) => R(y, x));

{ PROPERTY P3: graph T is connected and acyclic (T is a tree) }

{

This property is verified by a constraint programmed in the model generator, instead of a "heavy" logical formula:

1- it will refuse the partial interpretations in which a connected component of the graph (in construction) is cyclic, i.e. such as: number of edges >= number of vertices

2- it will refuse the complete interpretations in which the constructed graph has more than one connected component

}

{ PROPERTY P4: graph T respects minimal distance matrix }

{

This property is verified by a constraint programmed in the model generator:

it will refuse the partial interpretations in which the graph (in construction) do not respect the minimal distance matrix, i.e. such as:

let x, y a couple of OTUs,

let d= minimal distance calculated between x and y (encoded in the minimal distance matrix), there is a path of length k between x and y, with: k < d

The minimal distance matrix is encoded directly in the data structure of the model generator:

/* minimal distance matrix DEUTEROSTOMES taxA: */

DIST[0][0]=0;

DIST[1][0]=2; DIST[1][1]=0;

DIST[2][0]=2; DIST[2][1]=4; DIST[2][2]=0;

DIST[3][0]=1; DIST[3][1]=3; DIST[3][2]=3; DIST[3][3]=0;

DIST[4][0]=1; DIST[4][1]=3; DIST[4][2]=2; DIST[4][3]=1; DIST[4][4]=0;

DIST[5][0]=2; DIST[5][1]=4; DIST[5][2]=4; DIST[5][3]=3; DIST[5][4]=3; DIST[5][5]=0;

DIST[6][0]=3; DIST[6][1]=4; DIST[6][2]=4; DIST[6][3]=3; DIST[6][4]=3; DIST[6][5]=2; DIST[6][6]=0;

DIST[7][0]=2; DIST[7][1]=4; DIST[7][2]=4; DIST[7][3]=3; DIST[7][4]=3; DIST[7][5]=2; DIST[7][6]=1; DIST[7][7]=0;

DIST[8][0]=2; DIST[8][1]=4; DIST[8][2]=4; DIST[8][3]=3; DIST[8][4]=3; DIST[8][5]=1; DIST[8][6]=2; DIST[8][7]=1; DIST[8][8]=0;

DIST[9][0]=3; DIST[9][1]=5; DIST[9][2]=4; DIST[9][3]=4; DIST[9][4]=4; DIST[9][5]=1; DIST[9][6]=2; DIST[9][7]=2; DIST[9][8]=1; DIST[9][9]=0;

DIST[10][0]=3; DIST[10][1]=4; DIST[10][2]=4; DIST[10][3]=4; DIST[10][4]=4; DIST[10][5]=2; DIST[10][6]=3; DIST[10][7]=3; DIST[10][8]=2; DIST[10][9]=1; DIST[10][10]=0;

DIST[11][0]=3; DIST[11][1]=5; DIST[11][2]=4; DIST[11][3]=4; DIST[11][4]=4; DIST[11][5]=2; DIST[11][6]=3; DIST[11][7]=3; DIST[11][8]=2; DIST[11][9]=1; DIST[11][10]=1; DIST[11][11]=0;

}

{ PROPERTY P5: graph T respects eventual Primary Phylogenetic Hypotheses }

{

This property is verified by constraints programmed in the model generator:

- monophyly of Deuterostomia = (0,1,3,4,5,6,7,8,9,10,11)

- monophyly of Chordata = (0,1)

- monophyly of Ambulacria = (4,5,6,7,8,9,10,11)

- monophyly of Echinodermata = (5,6,7,8,9,10,11)

- monophyly of Ophiurida = (6,7)

- monophyly of Crinoidea = (9,10,11)

- monophyly of Eleutherozoa = (5,6,7,8) // additionnal PPH

}

{------------------------------------------------------------------------------------------------------------------------}

{ PROPERTY P6: it is possible to calculate all the values for each HTU in the graph T }

{

First we calculate with the model generator the set of tree solutions which verify properties P1 to P5. Property P6 is verified *a posteriori* for each tree solution, with a *feedback* mechanism:

Studying each tree solution for calculating the values of HTUs, we eventually discover "impossible sub-trees": they appear in tree solutions which verify P1 to P5, but they do not verify P6.

For each impossible subtree A, an additional constraint is programmed into the model generator to forbid the solutions containing A. Tree solutions are recalculated and verified, allowing the discovery of new impossible subtrees and the programming of new constraints to recalculate the solutions (feedback mechanism). Finally, the complete set of optimal solutions is determined after iteration of this process and elimination of all the solutions that do not verify P6.

}

================================================================================

================================================================================

SOLUTIONS

================================================================================

================================================================================

OTUs:

homo_sapiens=0;

asymmetron_inferum=1;

limulus_polyphemus=2;

xenoturbella=3;

balanoglossus_carnosus=4;

strongylocentrotus_purpuratus=5;

ophiura_lukteni=6;

ophiobolis_aculeata=7;

asterina_pectinifera=8;

florometra_serratissima=9;

gymnocrinus_richeri=10;

antedon_mediterranea=11;

HTUs:

n1, n2, n3

D = [0,14]: 6 solutions OK (which verify property P6) (4 impossible sub-trees)

minimal score (best) = 43

maximal score = 52

-------------------------------------------------------------------------------------------------------------

-> solution with Ur-echinodermata = strongylocentrotus_purpuratus

(confirmed by a local analysis of the crinoids with tRNAs genes)

score = 59:

model 1:

-------------

R(0,3) R(0,4) R(0,n1) R(0,n2) R(0,n3) R(1,n1) R(2,n2) R(3,0) R(4,0) R(5,8) R(5,9) R(5,n3) R(6,7) R(7,6) R(7,8) R(8,5) R(8,7) R(9,5) R(9,10) R(9,11) R(10,9) R(11,9) R(n1,0) R(n1,1) R(n2,0) R(n2,2) R(n3,0) R(n3,5)

-------------------------------------------------------------------------------------------------------------

score = 68:

model 2:

-------------

R(0,3) R(0,4) R(0,n1) R(0,n2) R(0,n3) R(1,n1) R(2,n2) R(3,0) R(4,0) R(5,8) R(5,9) R(5,n3) R(6,7) R(7,6) R(7,8) R(8,5) R(8,7) R(9,5) R(9,10) R(10,9) R(10,11) R(11,10) R(n1,0) R(n1,1) R(n2,0) R(n2,2) R(n3,0) R(n3,5)

-------------------------------------------------------------------------------------------------------------

score = 68:

model 3:

-------------

R(0,3) R(0,4) R(0,n1) R(0,n2) R(0,n3) R(1,n1) R(2,n2) R(3,0) R(4,0) R(5,8) R(5,9) R(5,n3) R(6,7) R(7,6) R(7,8) R(8,5) R(8,7) R(9,5) R(9,11) R(10,11) R(11,9) R(11,10) R(n1,0) R(n1,1) R(n2,0) R(n2,2) R(n3,0) R(n3,5)

-------------------------------------------------------------------------------------------------------------

-> solution with Ur-echinodermata = asterina_pectinifera

(confirmed by a local analysis of the crinoids with tRNAs genes)

score = 43:

model 4:

-------------

R(0,3) R(0,4) R(0,n1) R(0,n2) R(0,n3) R(1,n1) R(2,n2) R(3,0) R(4,0) R(5,8) R(6,7) R(7,6) R(7,8) R(8,5) R(8,7) R(8,9) R(8,n3) R(9,8) R(9,10) R(9,11) R(10,9) R(11,9) R(n1,0) R(n1,1) R(n2,0) R(n2,2) R(n3,0) R(n3,8)

-------------------------------------------------------------------------------------------------------------

score = 52:

model 5:

-------------

R(0,3) R(0,4) R(0,n1) R(0,n2) R(0,n3) R(1,n1) R(2,n2) R(3,0) R(4,0) R(5,8) R(6,7) R(7,6) R(7,8) R(8,5) R(8,7) R(8,9) R(8,n3) R(9,8) R(9,10) R(10,9) R(10,11) R(11,10) R(n1,0) R(n1,1) R(n2,0) R(n2,2) R(n3,0) R(n3,8)

-------------------------------------------------------------------------------------------------------------

score = 52:

model 6:

-------------

R(0,3) R(0,4) R(0,n1) R(0,n2) R(0,n3) R(1,n1) R(2,n2) R(3,0) R(4,0) R(5,8) R(6,7) R(7,6) R(7,8) R(8,5) R(8,7) R(8,9) R(8,n3) R(9,8) R(9,11) R(10,11) R(11,9) R(11,10) R(n1,0) R(n1,1) R(n2,0) R(n2,2) R(n3,0) R(n3,8)

-------------------------------------------------------------------------------------------------------------

-> no other models

deuterostomes_taxA_v3_3sol

================================================================================

================================================================================

AXIOMS

================================================================================

================================================================================

{ the solutions of problem PHYLO are the smallest graphs T (defined on the smallest domain possible but containing at least all the OTUs) which verify properties P1 to P6:

P1- T is simple (the relation R(x, y) which defines graph T is not reflexive)

P2- T is non-oriented (the relation R(x, y) which defines graph T is symetrical)

P3- T is connected and acyclic (T is a tree)

P4- T respects the minimal distance matrix, i.e.:

for all couple of OTUs x and y, the length of the path x->y in T is always superior or equals to the minimal distance calculated between x and y (encoded in the minimal distance matrix)

P5- T respects other eventual hypothesis (Primary Phylogenetic Hypothesis = PPH)

used to impose the existence of given monophyletic groups

P6- it is possible to calculate all the values for each HTU in the graph T }

{ OTUs: }

homo_sapiens=0;

asymmetron_inferum=1;

limulus_polyphemus=2; { = outgroup }

xenoturbella=3;

balanoglossus_carnosus=4;

strongylocentrotus_purpuratus=5;

ophiura_lukteni=6;

ophiobolis_aculeata=7;

asterina_pectinifera=8;

florometra_serratissima=9;

gymnocrinus_richeri=10;

antedon_mediterranea=11;

{ PROPERTY P1: R(x, y) is not reflexive}

Q x (-R(x, x));

{ PROPERTY P2: R(x, y) is symetrical}

Q x y (R(x, y) => R(y, x));

{ PROPERTY P3: graph T is connected and acyclic (T is a tree) }

{

This property is verified by a constraint programmed in the model generator, instead of a "heavy" logical formula:

1- it will refuse the partial interpretations in which a connected component of the graph (in construction) is cyclic, i.e. such as: number of edges >= number of vertices

2- it will refuse the complete interpretations in which the constructed graph has more than one connected component

}

{ PROPERTY P4: graph T respects minimal distance matrix }

{

This property is verified by a constraint programmed in the model generator:

it will refuse the partial interpretations in which the graph (in construction) do not respect the minimal distance matrix, i.e. such as:

let x, y a couple of OTUs,

let d= minimal distance calculated between x and y (encoded in the minimal distance matrix), there is a a path of length k between x and y, with: k < d

The minimal distance matrix is encoded directly in the data structure of the model generator:

/* minimal distance matrix DEUTEROSTOMES taxA: */

DIST[0][0]=0;

DIST[1][0]=2; DIST[1][1]=0;

DIST[2][0]=2; DIST[2][1]=4; DIST[2][2]=0;

DIST[3][0]=1; DIST[3][1]=3; DIST[3][2]=3; DIST[3][3]=0;

DIST[4][0]=1; DIST[4][1]=3; DIST[4][2]=2; DIST[4][3]=1; DIST[4][4]=0;

DIST[5][0]=2; DIST[5][1]=4; DIST[5][2]=4; DIST[5][3]=3; DIST[5][4]=3; DIST[5][5]=0;

DIST[6][0]=3; DIST[6][1]=4; DIST[6][2]=4; DIST[6][3]=3; DIST[6][4]=3; DIST[6][5]=2; DIST[6][6]=0;

DIST[7][0]=2; DIST[7][1]=4; DIST[7][2]=4; DIST[7][3]=3; DIST[7][4]=3; DIST[7][5]=2; DIST[7][6]=1; DIST[7][7]=0;

DIST[8][0]=2; DIST[8][1]=4; DIST[8][2]=4; DIST[8][3]=3; DIST[8][4]=3; DIST[8][5]=1; DIST[8][6]=2; DIST[8][7]=1; DIST[8][8]=0;

DIST[9][0]=3; DIST[9][1]=5; DIST[9][2]=4; DIST[9][3]=4; DIST[9][4]=4; DIST[9][5]=1; DIST[9][6]=2; DIST[9][7]=2; DIST[9][8]=1; DIST[9][9]=0;

DIST[10][0]=3; DIST[10][1]=4; DIST[10][2]=4; DIST[10][3]=4; DIST[10][4]=4; DIST[10][5]=2; DIST[10][6]=3; DIST[10][7]=3; DIST[10][8]=2; DIST[10][9]=1; DIST[10][10]=0;

DIST[11][0]=3; DIST[11][1]=5; DIST[11][2]=4; DIST[11][3]=4; DIST[11][4]=4; DIST[11][5]=2; DIST[11][6]=3; DIST[11][7]=3; DIST[11][8]=2; DIST[11][9]=1; DIST[11][10]=1; DIST[11][11]=0;

}

{ PROPERTY P5: graph T respects eventual Primary Phylogenetic Hypotheses }

{

This property is verified by constraints programmed in the model generator:

- monophyly of Deuterostomia = (0,1,3,4,5,6,7,8,9,10,11)

- monophyly of Chordata = (0,1)

- monophyly of Ambulacria = (4,5,6,7,8,9,10,11)

- monophyly of Echinodermata = (5,6,7,8,9,10,11)

- monophyly of Ophiurida = (6,7)

- monophyly of Crinoidea = (9,10,11)

- monophyly of Eleutherozoa = (5,6,7,8) // additionnal PPH

- monophyly of (Asteroidea+Echinoidea) = (5,8) // additionnal PPH

}

{------------------------------------------------------------------------------------------------------------------------}

{ PROPERTY P6: it is possible to calculate all the values for each HTU in the graph T }

{

First we calculate with the model generator the set of tree solutions which verify properties P1 to P5. Property P6 is verified *a posteriori* for each tree solution, with a *feedback* mechanism:

Studying each tree solution for calculating the values of HTUs, we eventually discover "impossible sub-trees": they appear in tree solutions which verify P1 to P5, but they do not verify P6.

For each impossible subtree A, an additional constraint is programmed into the model generator to forbid the solutions containing A. Tree solutions are recalculated and verified, allowing the discovery of new impossible subtrees and the programming of new constraints to recalculate the solutions (feedback mechanism). Finally, the complete set of optimal solutions is determined after iteration of this process and elimination of all the solutions that do not verify P6.

}

================================================================================

================================================================================

SOLUTIONS

================================================================================

================================================================================

OTUs:

homo_sapiens=0;

asymmetron_inferum=1;

limulus_polyphemus=2;

xenoturbella=3;

balanoglossus_carnosus=4;

strongylocentrotus_purpuratus=5;

ophiura_lukteni=6;

ophiobolis_aculeata=7;

asterina_pectinifera=8;

florometra_serratissima=9;

gymnocrinus_richeri=10;

antedon_mediterranea=11;

HTUs:

n1, n2, n3

D = [0,14]: 3 solutions OK (which verify property P6) (4 impossible sub-trees)

minimal score (best) = 43

maximal score = 52

-------------------------------------------------------------------------------------------------------------

-> solution with Ur-echinodermta = asterina_pectinifera

(confirmed by a local analysis of the crinoids with tRNAs genes)

score = 43:

model 1:

-------------

R(0,3) R(0,4) R(0,n1) R(0,n2) R(0,n3) R(1,n1) R(2,n2) R(3,0) R(4,0) R(5,8) R(6,7) R(7,6) R(7,8) R(8,5) R(8,7) R(8,9) R(8,n3) R(9,8) R(9,10) R(9,11) R(10,9) R(11,9) R(n1,0) R(n1,1) R(n2,0) R(n2,2) R(n3,0) R(n3,8)

-------------------------------------------------------------------------------------------------------------

score = 52:

model 2:

-------------

R(0,3) R(0,4) R(0,n1) R(0,n2) R(0,n3) R(1,n1) R(2,n2) R(3,0) R(4,0) R(5,8) R(6,7) R(7,6) R(7,8) R(8,5) R(8,7) R(8,9) R(8,n3) R(9,8) R(9,10) R(10,9) R(10,11) R(11,10) R(n1,0) R(n1,1) R(n2,0) R(n2,2) R(n3,0) R(n3,8)

-------------------------------------------------------------------------------------------------------------

score = 52:

model 3:

-------------

R(0,3) R(0,4) R(0,n1) R(0,n2) R(0,n3) R(1,n1) R(2,n2) R(3,0) R(4,0) R(5,8) R(6,7) R(7,6) R(7,8) R(8,5) R(8,7) R(8,9) R(8,n3) R(9,8) R(9,11) R(10,11) R(11,9) R(11,10) R(n1,0) R(n1,1) R(n2,0) R(n2,2) R(n3,0) R(n3,8)

-------------------------------------------------------------------------------------------------------------

-> no other models

deuterostomes_taxA_v4_3sol

================================================================================

================================================================================

AXIOMS

================================================================================

================================================================================

{ the solutions of problem PHYLO are the smallest graphs T (defined on the smallest domain possible but containing at least all the OTUs) which verify properties P1 to P6:

P1- T is simple (the relation R(x, y) which defines graph T is not reflexive)

P2- T is non-oriented (the relation R(x, y) which defines graph T is symetrical)

P3- T is connected and acyclic (T is a tree)

P4- T respects the minimal distance matrix, i.e.:

for all couple of OTUs x and y, the length of the path x->y in T is always superior or equals to the minimal distance calculated between x and y (encoded in the minimal distance matrix)

P5- T respects other eventual hypothesis (Primary Phylogenetic Hypothesis = PPH)

used to impose the existence of given monophyletic groups

P6- it is possible to calculate all the values for each HTU in the graph T }

{ OTUs: }

homo_sapiens=0;

asymmetron_inferum=1;

limulus_polyphemus=2; { = outgroup }

xenoturbella=3;

balanoglossus_carnosus=4;

strongylocentrotus_purpuratus=5;

ophiura_lukteni=6;

ophiobolis_aculeata=7;

asterina_pectinifera=8;

florometra_serratissima=9;

gymnocrinus_richeri=10;

antedon_mediterranea=11;

{ PROPERTY P1: R(x, y) is not reflexive}

Q x (-R(x, x));

{ PROPERTY P2: R(x, y) is symetrical}

Q x y (R(x, y) => R(y, x));

{ PROPERTY P3: graph T is connected and acyclic (T is a tree) }

{

This property is verified by a constraint programmed in the model generator, instead of a "heavy" logical formula:

1- it will refuse the partial interpretations in which a connected component of the graph (in construction) is cyclic, i.e. such as: number of edges >= number of vertices

2- it will refuse the complete interpretations in which the constructed graph has more than one connected component

}

{ PROPERTY P4: graph T respects minimal distance matrix }

{

This property is verified by a constraint programmed in the model generator:

it will refuse the partial interpretations in which the graph (in construction) do not respect the minimal distance matrix, i.e. such as:

let x, y a couple of OTUs,

let d= minimal distance calculated between x and y (encoded in the minimal distance matrix), there is a a path of length k between x and y, with: k < d

The minimal distance matrix is encoded directly in the data structure of the model generator:

/* minimal distance matrix DEUTEROSTOMES taxA: */

DIST[0][0]=0;

DIST[1][0]=2; DIST[1][1]=0;

DIST[2][0]=2; DIST[2][1]=4; DIST[2][2]=0;

DIST[3][0]=1; DIST[3][1]=3; DIST[3][2]=3; DIST[3][3]=0;

DIST[4][0]=1; DIST[4][1]=3; DIST[4][2]=2; DIST[4][3]=1; DIST[4][4]=0;

DIST[5][0]=2; DIST[5][1]=4; DIST[5][2]=4; DIST[5][3]=3; DIST[5][4]=3; DIST[5][5]=0;

DIST[6][0]=3; DIST[6][1]=4; DIST[6][2]=4; DIST[6][3]=3; DIST[6][4]=3; DIST[6][5]=2; DIST[6][6]=0;

DIST[7][0]=2; DIST[7][1]=4; DIST[7][2]=4; DIST[7][3]=3; DIST[7][4]=3; DIST[7][5]=2; DIST[7][6]=1; DIST[7][7]=0;

DIST[8][0]=2; DIST[8][1]=4; DIST[8][2]=4; DIST[8][3]=3; DIST[8][4]=3; DIST[8][5]=1; DIST[8][6]=2; DIST[8][7]=1; DIST[8][8]=0;

DIST[9][0]=3; DIST[9][1]=5; DIST[9][2]=4; DIST[9][3]=4; DIST[9][4]=4; DIST[9][5]=1; DIST[9][6]=2; DIST[9][7]=2; DIST[9][8]=1; DIST[9][9]=0;

DIST[10][0]=3; DIST[10][1]=4; DIST[10][2]=4; DIST[10][3]=4; DIST[10][4]=4; DIST[10][5]=2; DIST[10][6]=3; DIST[10][7]=3; DIST[10][8]=2; DIST[10][9]=1; DIST[10][10]=0;

DIST[11][0]=3; DIST[11][1]=5; DIST[11][2]=4; DIST[11][3]=4; DIST[11][4]=4; DIST[11][5]=2; DIST[11][6]=3; DIST[11][7]=3; DIST[11][8]=2; DIST[11][9]=1; DIST[11][10]=1; DIST[11][11]=0;

}

{ PROPERTY P5: graph T respects eventual Primary Phylogenetic Hypotheses }

{

This property is verified by constraints programmed in the model generator:

- monophyly of Deuterostomia = (0,1,3,4,5,6,7,8,9,10,11)

- monophyly of Chordata = (0,1)

- monophyly of Ambulacria = (4,5,6,7,8,9,10,11)

- monophyly of Echinodermata = (5,6,7,8,9,10,11)

- monophyly of Ophiurida = (6,7)

- monophyly of Crinoidea = (9,10,11)

- monophyly of Eleutherozoa = (5,6,7,8) // additionnal PPH

- monophyly of Cryptosyringid = (5,8) // additionnal PPH

}

{------------------------------------------------------------------------------------------------------------------------}

{ PROPERTY P6: it is possible to calculate all the values for each HTU in the graph T }

{

First we calculate with the model generator the set of tree solutions which verify properties P1 to P5. Property P6 is verified *a posteriori* for each tree solution, with a *feedback* mechanism:

Studying each tree solution for calculating the values of HTUs, we eventually discover "impossible sub-trees": they appear in tree solutions which verify P1 to P5, but they do not verify P6.

For each impossible subtree A, an additional constraint is programmed into the model generator to forbid the solutions containing A. Tree solutions are recalculated and verified, allowing the discovery of new impossible subtrees and the programming of new constraints to recalculate the solutions (feedback mechanism). Finally, the complete set of optimal solutions is determined after iteration of this process and elimination of all the solutions that do not verify P6.

}

================================================================================

================================================================================

SOLUTIONS

================================================================================

================================================================================

OTUs:

homo_sapiens=0;

asymmetron_inferum=1;

limulus_polyphemus=2;

xenoturbella=3;

balanoglossus_carnosus=4;

strongylocentrotus_purpuratus=5;

ophiura_lukteni=6;

ophiobolis_aculeata=7;

asterina_pectinifera=8;

florometra_serratissima=9;

gymnocrinus_richeri=10;

antedon_mediterranea=11;

HTUs:

n1, n2, n3

D = [0,14]: 3 solutions OK (which verify property P6) (4 impossible sub-trees)

minimal score (best) = 43

maximal score = 52

-------------------------------------------------------------------------------------------------------------

-> solution with Ur-echinodermata = asterina_pectinifera

(confirmed by a local analysis of the crinoids with tRNAs genes)

score = 43:

model 1:

-------------

R(0,3) R(0,4) R(0,n1) R(0,n2) R(0,n3) R(1,n1) R(2,n2) R(3,0) R(4,0) R(5,8) R(6,7) R(7,6) R(7,8) R(8,5) R(8,7) R(8,9) R(8,n3) R(9,8) R(9,10) R(9,11) R(10,9) R(11,9) R(n1,0) R(n1,1) R(n2,0) R(n2,2) R(n3,0) R(n3,8)

-------------------------------------------------------------------------------------------------------------

score = 52:

model 2:

-------------

R(0,3) R(0,4) R(0,n1) R(0,n2) R(0,n3) R(1,n1) R(2,n2) R(3,0) R(4,0) R(5,8) R(6,7) R(7,6) R(7,8) R(8,5) R(8,7) R(8,9) R(8,n3) R(9,8) R(9,10) R(10,9) R(10,11) R(11,10) R(n1,0) R(n1,1) R(n2,0) R(n2,2) R(n3,0) R(n3,8)

-------------------------------------------------------------------------------------------------------------

score = 52:

model 3:

-------------

R(0,3) R(0,4) R(0,n1) R(0,n2) R(0,n3) R(1,n1) R(2,n2) R(3,0) R(4,0) R(5,8) R(6,7) R(7,6) R(7,8) R(8,5) R(8,7) R(8,9) R(8,n3) R(9,8) R(9,11) R(10,11) R(11,9) R(11,10) R(n1,0) R(n1,1) R(n2,0) R(n2,2) R(n3,0) R(n3,8)

-------------------------------------------------------------------------------------------------------------

-> no other models

deuterostomes_taxA_v5_6sol

================================================================================

================================================================================

AXIOMS

================================================================================

================================================================================

{ the solutions of problem PHYLO are the smallest graphs T (defined on the smallest domain possible but containing at least all the OTUs) which verify properties P1 to P6:

P1- T is simple (the relation R(x, y) which defines graph T is not reflexive)

P2- T is non-oriented (the relation R(x, y) which defines graph T is symetrical)

P3- T is connected and acyclic (T is a tree)

P4- T respects the minimal distance matrix, i.e.:

for all couple of OTUs x and y, the length of the path x->y in T is always superior or equals to the minimal distance calculated between x and y (encoded in the minimal distance matrix)

P5- T respects other eventual hypothesis (Primary Phylogenetic Hypothesis = PPH)

used to impose the existence of given monophyletic groups

P6- it is possible to calculate all the values for each HTU in the graph T }

{ OTUs: }

homo_sapiens=0;

asymmetron_inferum=1;

limulus_polyphemus=2; { = outgroup }

xenoturbella=3;

balanoglossus_carnosus=4;

strongylocentrotus_purpuratus=5;

ophiura_lukteni=6;

ophiobolis_aculeata=7;

asterina_pectinifera=8;

florometra_serratissima=9;

gymnocrinus_richeri=10;

antedon_mediterranea=11;

{ PROPERTY P1: R(x, y) is not reflexive}

Q x (-R(x, x));

{ PROPERTY P2: R(x, y) is symetrical}

Q x y (R(x, y) => R(y, x));

{ PROPERTY P3: graph T is connected and acyclic (T is a tree) }

{

This property is verified by a constraint programmed in the model generator, instead of a "heavy" logical formula:

1- it will refuse the partial interpretations in which a connected component of the graph (in construction) is cyclic, i.e. such as: number of edges >= number of vertices

2- it will refuse the complete interpretations in which the constructed graph has more than one connected component

}

{ PROPERTY P4: graph T respects minimal distance matrix }

{

This property is verified by a constraint programmed in the model generator:

it will refuse the partial interpretations in which the graph (in construction) do not respect the minimal distance matrix, i.e. such as:

let x, y a couple of OTUs,

let d= minimal distance calculated between x and y (encoded in the minimal distance matrix), there is a a path of length k between x and y, with: k < d

The minimal distance matrix is encoded directly in the data structure of the model generator:

/* minimal distance matrix DEUTEROSTOMES taxA: */

DIST[0][0]=0;

DIST[1][0]=2; DIST[1][1]=0;

DIST[2][0]=2; DIST[2][1]=4; DIST[2][2]=0;

DIST[3][0]=1; DIST[3][1]=3; DIST[3][2]=3; DIST[3][3]=0;

DIST[4][0]=1; DIST[4][1]=3; DIST[4][2]=2; DIST[4][3]=1; DIST[4][4]=0;

DIST[5][0]=2; DIST[5][1]=4; DIST[5][2]=4; DIST[5][3]=3; DIST[5][4]=3; DIST[5][5]=0;

DIST[6][0]=3; DIST[6][1]=4; DIST[6][2]=4; DIST[6][3]=3; DIST[6][4]=3; DIST[6][5]=2; DIST[6][6]=0;

DIST[7][0]=2; DIST[7][1]=4; DIST[7][2]=4; DIST[7][3]=3; DIST[7][4]=3; DIST[7][5]=2; DIST[7][6]=1; DIST[7][7]=0;

DIST[8][0]=2; DIST[8][1]=4; DIST[8][2]=4; DIST[8][3]=3; DIST[8][4]=3; DIST[8][5]=1; DIST[8][6]=2; DIST[8][7]=1; DIST[8][8]=0;

DIST[9][0]=3; DIST[9][1]=5; DIST[9][2]=4; DIST[9][3]=4; DIST[9][4]=4; DIST[9][5]=1; DIST[9][6]=2; DIST[9][7]=2; DIST[9][8]=1; DIST[9][9]=0;

DIST[10][0]=3; DIST[10][1]=4; DIST[10][2]=4; DIST[10][3]=4; DIST[10][4]=4; DIST[10][5]=2; DIST[10][6]=3; DIST[10][7]=3; DIST[10][8]=2; DIST[10][9]=1; DIST[10][10]=0;

DIST[11][0]=3; DIST[11][1]=5; DIST[11][2]=4; DIST[11][3]=4; DIST[11][4]=4; DIST[11][5]=2; DIST[11][6]=3; DIST[11][7]=3; DIST[11][8]=2; DIST[11][9]=1; DIST[11][10]=1; DIST[11][11]=0;

}

{ PROPERTY P5: graph T respects eventual Primary Phylogenetic Hypotheses }

{

This property is verified by constraints programmed in the model generator:

- monophyly of Deuterostomia = (0,1,3,4,5,6,7,8,9,10,11)

- monophyly of Chordata = (0,1)

- monophyly of Ambulacria = (4,5,6,7,8,9,10,11)

- monophyly of Echinodermata = (5,6,7,8,9,10,11)

- monophyly of Ophiurida = (6,7)

- monophyly of Crinoidea = (9,10,11)

- monophyly of Eleutherozoa = (5,6,7,8) // additionnal PPH

- monophyly of Asterozoa = (6,7,8) // additionnal PPH

}

{------------------------------------------------------------------------------------------------------------------------}

{ PROPERTY P6: it is possible to calculate all the values for each HTU in the graph T }

{

First we calculate with the model generator the set of tree solutions which verify properties P1 to P5. Property P6 is verified *a posteriori* for each tree solution, with a *feedback* mechanism:

Studying each tree solution for calculating the values of HTUs, we eventually discover "impossible sub-trees": they appear in tree solutions which verify P1 to P5, but they do not verify P6.

For each impossible subtree A, an additional constraint is programmed into the model generator to forbid the solutions containing A. Tree solutions are recalculated and verified, allowing the discovery of new impossible subtrees and the programming of new constraints to recalculate the solutions (feedback mechanism). Finally, the complete set of optimal solutions is determined after iteration of this process and elimination of all the solutions that do not verify P6.

}

================================================================================

================================================================================

SOLUTIONS

================================================================================

================================================================================

OTUs:

homo_sapiens=0;

asymmetron_inferum=1;

limulus_polyphemus=2;

xenoturbella=3;

balanoglossus_carnosus=4;

strongylocentrotus_purpuratus=5;

ophiura_lukteni=6;

ophiobolis_aculeata=7;

asterina_pectinifera=8;

florometra_serratissima=9;

gymnocrinus_richeri=10;

antedon_mediterranea=11;

HTUs:

n1, n2, n3

D = [0,14]: 6 solutions OK (which verify property P6) (4 impossible sub-trees)

minimal score (best) = 43

maximal score = 52

-------------------------------------------------------------------------------------------------------------

-> solution with Ur-echinodermata = strongylocentrotus_purpuratus

(confirmed by a local analysis of the crinoids with tRNAs genes)

score = 59:

model 1:

-------------

R(0,3) R(0,4) R(0,n1) R(0,n2) R(0,n3) R(1,n1) R(2,n2) R(3,0) R(4,0) R(5,8) R(5,9) R(5,n3) R(6,7) R(7,6) R(7,8) R(8,5) R(8,7) R(9,5) R(9,10) R(9,11) R(10,9) R(11,9) R(n1,0) R(n1,1) R(n2,0) R(n2,2) R(n3,0) R(n3,5)

-------------------------------------------------------------------------------------------------------------

score = 68:

model 2:

-------------

R(0,3) R(0,4) R(0,n1) R(0,n2) R(0,n3) R(1,n1) R(2,n2) R(3,0) R(4,0) R(5,8) R(5,9) R(5,n3) R(6,7) R(7,6) R(7,8) R(8,5) R(8,7) R(9,5) R(9,10) R(10,9) R(10,11) R(11,10) R(n1,0) R(n1,1) R(n2,0) R(n2,2) R(n3,0) R(n3,5)

-------------------------------------------------------------------------------------------------------------

score = 68:

model 3:

-------------

R(0,3) R(0,4) R(0,n1) R(0,n2) R(0,n3) R(1,n1) R(2,n2) R(3,0) R(4,0) R(5,8) R(5,9) R(5,n3) R(6,7) R(7,6) R(7,8) R(8,5) R(8,7) R(9,5) R(9,11) R(10,11) R(11,9) R(11,10) R(n1,0) R(n1,1) R(n2,0) R(n2,2) R(n3,0) R(n3,5)

-------------------------------------------------------------------------------------------------------------

-> solution with Ur-echinodermata = asterina_pectinifera

(confirmed by a local analysis of the crinoids with tRNAs genes)

score = 43:

model 4:

-------------

R(0,3) R(0,4) R(0,n1) R(0,n2) R(0,n3) R(1,n1) R(2,n2) R(3,0) R(4,0) R(5,8) R(6,7) R(7,6) R(7,8) R(8,5) R(8,7) R(8,9) R(8,n3) R(9,8) R(9,10) R(9,11) R(10,9) R(11,9) R(n1,0) R(n1,1) R(n2,0) R(n2,2) R(n3,0) R(n3,8)

-------------------------------------------------------------------------------------------------------------

score = 52:

model 5:

-------------

R(0,3) R(0,4) R(0,n1) R(0,n2) R(0,n3) R(1,n1) R(2,n2) R(3,0) R(4,0) R(5,8) R(6,7) R(7,6) R(7,8) R(8,5) R(8,7) R(8,9) R(8,n3) R(9,8) R(9,10) R(10,9) R(10,11) R(11,10) R(n1,0) R(n1,1) R(n2,0) R(n2,2) R(n3,0) R(n3,8)

-------------------------------------------------------------------------------------------------------------

score = 52:

model 6:

-------------

R(0,3) R(0,4) R(0,n1) R(0,n2) R(0,n3) R(1,n1) R(2,n2) R(3,0) R(4,0) R(5,8) R(6,7) R(7,6) R(7,8) R(8,5) R(8,7) R(8,9) R(8,n3) R(9,8) R(9,11) R(10,11) R(11,9) R(11,10) R(n1,0) R(n1,1) R(n2,0) R(n2,2) R(n3,0) R(n3,8)

-------------------------------------------------------------------------------------------------------------

-> no other models

deuterostomes_taxA_v6_21sol

================================================================================

================================================================================

AXIOMS

================================================================================

================================================================================

{ the solutions of problem PHYLO are the smallest graphs T (defined on the smallest domain possible but containing at least all the OTUs) which verify properties P1 to P6:

P1- T is simple (the relation R(x, y) which defines graph T is not reflexive)

P2- T is non-oriented (the relation R(x, y) which defines graph T is symetrical)

P3- T is connected and acyclic (T is a tree)

P4- T respects the minimal distance matrix, i.e.:

for all couple of OTUs x and y, the length of the path x->y in T is always superior or equals to the minimal distance calculated between x and y (encoded in the minimal distance matrix)

P5- T respects other eventual hypothesis (Primary Phylogenetic Hypothesis = PPH)

used to impose the existence of given monophyletic groups

P6- it is possible to calculate all the values for each HTU in the graph T }

{ OTUs: }

homo_sapiens=0;

asymmetron_inferum=1;

limulus_polyphemus=2; { = outgroup }

xenoturbella=3;

balanoglossus_carnosus=4;

strongylocentrotus_purpuratus=5;

ophiura_lukteni=6;

ophiobolis_aculeata=7;

asterina_pectinifera=8;

florometra_serratissima=9;

gymnocrinus_richeri=10;

antedon_mediterranea=11;

{ THE CRINOIDEA GROUP IS FIXED to one of the 3 possible forms: }

R(gymnocrinus_richeri,florometra_serratissima);

Q x ( x<>florometra_serratissima => -R(gymnocrinus_richeri,x)

);

R(antedon_mediterranea,florometra_serratissima);

Q x ( x<>florometra_serratissima => -R(antedon_mediterranea,x)

);

{ PROPERTY P1: R(x, y) is not reflexive}

Q x (-R(x, x));

{ PROPERTY P2: R(x, y) is symetrical}

Q x y (R(x, y) => R(y, x));

{ PROPERTY P3: graph T is connected and acyclic (T is a tree) }

{

This property is verified by a constraint programmed in the model generator, instead of a "heavy" logical formula:

1- it will refuse the partial interpretations in which a connected component of the graph (in construction) is cyclic, i.e. such as: number of edges >= number of vertices

2- it will refuse the complete interpretations in which the constructed graph has more than one connected component

}

{ PROPERTY P4: graph T respects minimal distance matrix }

{

This property is verified by a constraint programmed in the model generator:

it will refuse the partial interpretations in which the graph (in construction) do not respect the minimal distance matrix, i.e. such as:

let x, y a couple of OTUs,

let d= minimal distance calculated between x and y (encoded in the minimal distance matrix), there is a a path of length k between x and y, with: k < d

The minimal distance matrix is encoded directly in the data structure of the model generator:

/* minimal distance matrix DEUTEROSTOMES taxA: */

DIST[0][0]=0;

DIST[1][0]=2; DIST[1][1]=0;

DIST[2][0]=2; DIST[2][1]=4; DIST[2][2]=0;

DIST[3][0]=1; DIST[3][1]=3; DIST[3][2]=3; DIST[3][3]=0;

DIST[4][0]=1; DIST[4][1]=3; DIST[4][2]=2; DIST[4][3]=1; DIST[4][4]=0;

DIST[5][0]=2; DIST[5][1]=4; DIST[5][2]=4; DIST[5][3]=3; DIST[5][4]=3; DIST[5][5]=0;

DIST[6][0]=3; DIST[6][1]=4; DIST[6][2]=4; DIST[6][3]=3; DIST[6][4]=3; DIST[6][5]=2; DIST[6][6]=0;

DIST[7][0]=2; DIST[7][1]=4; DIST[7][2]=4; DIST[7][3]=3; DIST[7][4]=3; DIST[7][5]=2; DIST[7][6]=1; DIST[7][7]=0;

DIST[8][0]=2; DIST[8][1]=4; DIST[8][2]=4; DIST[8][3]=3; DIST[8][4]=3; DIST[8][5]=1; DIST[8][6]=2; DIST[8][7]=1; DIST[8][8]=0;

DIST[9][0]=3; DIST[9][1]=5; DIST[9][2]=4; DIST[9][3]=4; DIST[9][4]=4; DIST[9][5]=1; DIST[9][6]=2; DIST[9][7]=2; DIST[9][8]=1; DIST[9][9]=0;

DIST[10][0]=3; DIST[10][1]=4; DIST[10][2]=4; DIST[10][3]=4; DIST[10][4]=4; DIST[10][5]=2; DIST[10][6]=3; DIST[10][7]=3; DIST[10][8]=2; DIST[10][9]=1; DIST[10][10]=0;

DIST[11][0]=3; DIST[11][1]=5; DIST[11][2]=4; DIST[11][3]=4; DIST[11][4]=4; DIST[11][5]=2; DIST[11][6]=3; DIST[11][7]=3; DIST[11][8]=2; DIST[11][9]=1; DIST[11][10]=1; DIST[11][11]=0;

}

{ PROPERTY P5: graph T respects eventual Primary Phylogenetic Hypotheses }

{

This property is verified by constraints programmed in the model generator:

- monophyly of Deuterostomia = (0,1,3,4,5,6,7,8,9,10,11)

- monophyly of Chordata = (0,1)

- monophyly of Ambulacria = (4,5,6,7,8,9,10,11)

- monophyly of Echinodermata = (5,6,7,8,9,10,11)

- monophyly of Ophiurida = (6,7)

- monophyly of Crinoidea = (9,10,11)

}

{------------------------------------------------------------------------------------------------------------------------}

{ PROPERTY P6: it is possible to calculate all the values for each HTU in the graph T }

{

First we calculate with the model generator the set of tree solutions which verify properties P1 to P5. Property P6 is verified *a posteriori* for each tree solution, with a *feedback* mechanism:

Studying each tree solution for calculating the values of HTUs, we eventually discover "impossible sub-trees": they appear in tree solutions which verify P1 to P5, but they do not verify P6.

For each impossible subtree A, an additional constraint is programmed into the model generator to forbid the solutions containing A. Tree solutions are recalculated and verified, allowing the discovery of new impossible subtrees and the programming of new constraints to recalculate the solutions (feedback mechanism). Finally, the complete set of optimal solutions is determined after iteration of this process and elimination of all the solutions that do not verify P6.

}

================================================================================

================================================================================

SOLUTIONS

================================================================================

================================================================================

OTUs:

homo_sapiens=0;

asymmetron_inferum=1;

limulus_polyphemus=2;

xenoturbella=3;

balanoglossus_carnosus=4;

strongylocentrotus_purpuratus=5;

ophiura_lukteni=6;

ophiobolis_aculeata=7;

asterina_pectinifera=8;

florometra_serratissima=9;

gymnocrinus_richeri=10;

antedon_mediterranea=11;

HTUs:

n1, n2, n3

-> the crinoidea group is fixed (to one of the 3 possible forms)

D = [0,14]: 21 solutions OK (which verify property P6) (4 impossible sub-trees)

minimal score (best) = 40

maximal score = 79

-------------------------------------------------------------------------------------------------------------

score = 64:

model 1:

-------------

R(0,3) R(0,4) R(0,n1) R(0,n2) R(0,n3) R(1,n2) R(2,n3) R(3,0) R(4,0) R(5,8) R(5,9) R(6,7) R(7,6) R(7,8) R(8,5) R(8,7) R(8,n1) R(9,5) R(9,10) R(9,11) R(10,9) R(11,9) R(n1,0) R(n1,8) R(n2,0) R(n2,1) R(n3,0) R(n3,2)

-------------------------------------------------------------------------------------------------------------

score = 59:

model 2:

-------------

R(0,3) R(0,4) R(0,n1) R(0,n2) R(0,n3) R(1,n1) R(2,n2) R(3,0) R(4,0) R(5,8) R(5,9) R(5,n3) R(6,7) R(7,6) R(7,8) R(8,5) R(8,7) R(9,5) R(9,10) R(9,11) R(10,9) R(11,9) R(n1,0) R(n1,1) R(n2,0) R(n2,2) R(n3,0) R(n3,5)

-------------------------------------------------------------------------------------------------------------

score = 79:

model 3:

-------------

R(0,3) R(0,4) R(0,n1) R(0,n2) R(0,n3) R(1,n1) R(2,n2) R(3,0) R(4,0) R(5,8) R(5,9) R(6,7) R(7,6) R(7,8) R(7,n3) R(8,5) R(8,7) R(9,5) R(9,10) R(9,11) R(10,9) R(11,9) R(n1,0) R(n1,1) R(n2,0) R(n2,2) R(n3,0) R(n3,7)

-------------------------------------------------------------------------------------------------------------

score = 64:

model 4:

-------------

R(0,3) R(0,4) R(0,n1) R(0,n2) R(0,n3) R(1,n2) R(2,n3) R(3,0) R(4,0) R(5,8) R(5,9) R(6,7) R(7,6) R(7,n1) R(8,5) R(8,n1) R(9,5) R(9,10) R(9,11) R(10,9) R(11,9) R(n1,0) R(n1,7) R(n1,8) R(n2,0) R(n2,1) R(n3,0) R(n3,2)

-------------------------------------------------------------------------------------------------------------

score = 43:

model 5:

-------------

R(0,3) R(0,4) R(0,n1) R(0,n2) R(0,n3) R(1,n1) R(2,n2) R(3,0) R(4,0) R(5,8) R(5,9) R(5,n3) R(6,7) R(7,6) R(7,n3) R(8,5) R(9,5) R(9,10) R(9,11) R(10,9) R(11,9) R(n1,0) R(n1,1) R(n2,0) R(n2,2) R(n3,0) R(n3,5) R(n3,7)

-------------------------------------------------------------------------------------------------------------

score = 43:

model 6:

-------------

R(0,3) R(0,4) R(0,n1) R(0,n2) R(0,n3) R(1,n2) R(2,n3) R(3,0) R(4,0) R(5,8) R(6,7) R(7,6) R(7,8) R(8,5) R(8,7) R(8,9) R(8,n1) R(9,8) R(9,10) R(9,11) R(10,9) R(11,9) R(n1,0) R(n1,8) R(n2,0) R(n2,1) R(n3,0) R(n3,2)

-------------------------------------------------------------------------------------------------------------

score = 68:

model 7:

-------------

R(0,3) R(0,4) R(0,n1) R(0,n2) R(0,n3) R(1,n1) R(2,n2) R(3,0) R(4,0) R(5,8) R(5,n3) R(6,7) R(7,6) R(7,8) R(8,5) R(8,7) R(8,9) R(9,8) R(9,10) R(9,11) R(10,9) R(11,9) R(n1,0) R(n1,1) R(n2,0) R(n2,2) R(n3,0) R(n3,5)

-------------------------------------------------------------------------------------------------------------

score = 58:

model 8:

-------------

R(0,3) R(0,4) R(0,n1) R(0,n2) R(0,n3) R(1,n1) R(2,n2) R(3,0) R(4,0) R(5,8) R(6,7) R(7,6) R(7,8) R(7,n3) R(8,5) R(8,7) R(8,9) R(9,8) R(9,10) R(9,11) R(10,9) R(11,9) R(n1,0) R(n1,1) R(n2,0) R(n2,2) R(n3,0) R(n3,7)

-------------------------------------------------------------------------------------------------------------

score = 43:

model 9:

-------------

R(0,3) R(0,4) R(0,n1) R(0,n2) R(0,n3) R(1,n2) R(2,n3) R(3,0) R(4,0) R(5,8) R(6,7) R(7,6) R(7,n1) R(8,5) R(8,9) R(8,n1) R(9,8) R(9,10) R(9,11) R(10,9) R(11,9) R(n1,0) R(n1,7) R(n1,8) R(n2,0) R(n2,1) R(n3,0) R(n3,2)

-------------------------------------------------------------------------------------------------------------

score = 64:

model 10:

-------------

R(0,3) R(0,4) R(0,n1) R(0,n2) R(0,n3) R(1,n1) R(2,n2) R(3,0) R(4,0) R(5,8) R(5,n3) R(6,7) R(7,6) R(7,n3) R(8,5) R(8,9) R(9,8) R(9,10) R(9,11) R(10,9) R(11,9) R(n1,0) R(n1,1) R(n2,0) R(n2,2) R(n3,0) R(n3,5) R(n3,7)

-------------------------------------------------------------------------------------------------------------

score = 48:

model 11:

-------------

R(0,3) R(0,4) R(0,n1) R(0,n2) R(0,n3) R(1,n2) R(2,n3) R(3,0) R(4,0) R(5,9) R(6,7) R(7,6) R(7,8) R(8,7) R(8,9) R(8,n1) R(9,5) R(9,8) R(9,10) R(9,11) R(10,9) R(11,9) R(n1,0) R(n1,8) R(n2,0) R(n2,1) R(n3,0) R(n3,2)

-------------------------------------------------------------------------------------------------------------

score = 68:

model 12:

-------------

R(0,3) R(0,4) R(0,n1) R(0,n2) R(0,n3) R(1,n1) R(2,n2) R(3,0) R(4,0) R(5,9) R(5,n3) R(6,7) R(7,6) R(7,8) R(8,7) R(8,9) R(9,5) R(9,8) R(9,10) R(9,11) R(10,9) R(11,9) R(n1,0) R(n1,1) R(n2,0) R(n2,2) R(n3,0) R(n3,5)

-------------------------------------------------------------------------------------------------------------

score = 63:

model 13:

-------------

R(0,3) R(0,4) R(0,n1) R(0,n2) R(0,n3) R(1,n1) R(2,n2) R(3,0) R(4,0) R(5,9) R(6,7) R(7,6) R(7,8) R(7,n3) R(8,7) R(8,9) R(9,5) R(9,8) R(9,10) R(9,11) R(10,9) R(11,9) R(n1,0) R(n1,1) R(n2,0) R(n2,2) R(n3,0) R(n3,7)

-------------------------------------------------------------------------------------------------------------

score = 47:

model 14:

-------------

R(0,3) R(0,4) R(0,n1) R(0,n2) R(0,n3) R(1,n1) R(2,n2) R(3,0) R(4,0) R(5,9) R(5,n3) R(6,7) R(7,6) R(7,8) R(7,n3) R(8,7) R(9,5) R(9,10) R(9,11) R(10,9) R(11,9) R(n1,0) R(n1,1) R(n2,0) R(n2,2) R(n3,0) R(n3,5) R(n3,7)

-------------------------------------------------------------------------------------------------------------

score = 56:

model 15:

-------------

R(0,3) R(0,4) R(0,n1) R(0,n2) R(0,n3) R(1,n1) R(2,n2) R(3,0) R(4,0) R(5,9) R(5,n3) R(6,7) R(7,6) R(7,8) R(8,7) R(8,n3) R(9,5) R(9,10) R(9,11) R(10,9) R(11,9) R(n1,0) R(n1,1) R(n2,0) R(n2,2) R(n3,0) R(n3,5) R(n3,8)

-------------------------------------------------------------------------------------------------------------

score = 48:

model 16:

-------------

R(0,3) R(0,4) R(0,n1) R(0,n2) R(0,n3) R(1,n2) R(2,n3) R(3,0) R(4,0) R(5,9) R(6,7) R(7,6) R(7,n1) R(8,9) R(8,n1) R(9,5) R(9,8) R(9,10) R(9,11) R(10,9) R(11,9) R(n1,0) R(n1,7) R(n1,8) R(n2,0) R(n2,1) R(n3,0) R(n3,2)

-------------------------------------------------------------------------------------------------------------

score = 48:

model 17:

-------------

R(0,3) R(0,4) R(0,n1) R(0,n2) R(0,n3) R(1,n1) R(2,n2) R(3,0) R(4,0) R(5,9) R(5,n3) R(6,7) R(7,6) R(7,n3) R(8,9) R(9,5) R(9,8) R(9,10) R(9,11) R(10,9) R(11,9) R(n1,0) R(n1,1) R(n2,0) R(n2,2) R(n3,0) R(n3,5) R(n3,7)

-------------------------------------------------------------------------------------------------------------

score = 40:

model 18:

-------------

R(0,3) R(0,4) R(0,n1) R(0,n2) R(0,n3) R(1,n1) R(2,n2) R(3,0) R(4,0) R(5,9) R(5,n3) R(6,7) R(7,6) R(7,n3) R(8,n3) R(9,5) R(9,10) R(9,11) R(10,9) R(11,9) R(n1,0) R(n1,1) R(n2,0) R(n2,2) R(n3,0) R(n3,5) R(n3,7) R(n3,8)

-------------------------------------------------------------------------------------------------------------

score = 44:

model 19:

-------------

R(0,3) R(0,4) R(0,n1) R(0,n2) R(0,n3) R(1,n2) R(2,n3) R(3,0) R(4,0) R(5,n1) R(6,7) R(7,6) R(7,8) R(8,7) R(8,9) R(8,n1) R(9,8) R(9,10) R(9,11) R(10,9) R(11,9) R(n1,0) R(n1,5) R(n1,8) R(n2,0) R(n2,1) R(n3,0) R(n3,2)

-------------------------------------------------------------------------------------------------------------

score = 56:

model 20:

-------------

R(0,3) R(0,4) R(0,n1) R(0,n2) R(0,n3) R(1,n1) R(2,n2) R(3,0) R(4,0) R(5,n3) R(6,7) R(7,6) R(7,8) R(7,n3) R(8,7) R(8,9) R(9,8) R(9,10) R(9,11) R(10,9) R(11,9) R(n1,0) R(n1,1) R(n2,0) R(n2,2) R(n3,0) R(n3,5) R(n3,7)

-------------------------------------------------------------------------------------------------------------

score = 40:

model 21:

-------------

R(0,3) R(0,4) R(0,n1) R(0,n2) R(0,n3) R(1,n2) R(2,n3) R(3,0) R(4,0) R(5,n1) R(6,7) R(7,6) R(7,n1) R(8,9) R(8,n1) R(9,8) R(9,10) R(9,11) R(10,9) R(11,9) R(n1,0) R(n1,5) R(n1,7) R(n1,8) R(n2,0) R(n2,1) R(n3,0) R(n3,2)

-------------------------------------------------------------------------------------------------------------

-> no other models

deuterostomes_taxB_63sol

================================================================================

================================================================================

AXIOMS

================================================================================

================================================================================

{ the solutions of problem PHYLO are the smallest graphs T (defined on the smallest domain possible but containing at least all the OTUs) which verify properties P1 to P6:

P1- T is simple (the relation R(x, y) which defines graph T is not reflexive)

P2- T is non-oriented (the relation R(x, y) which defines graph T is symetrical)

P3- T is connected and acyclic (T is a tree)

P4- T respects the minimal distance matrix, i.e.:

for all couple of OTUs x and y, the length of the path X->Y in T is always superior or equals to the minimal distance calculated between x and y (encoded in the minimal distance matrix)

P5- T respects other eventual hypothesis (Primary Phylogenetic Hypothesis = PPH)

used to impose the existence of given monophyletic groups

P6- it is possible to calculate all the values for each HTU in the graph T }

{ OTUs: }

homo_sapiens=0;

asymmetron_inferum=1;

limulus_polyphemus=2; { = outgroup1 }

xenoturbella=3;

balanoglossus_carnosus=4;

strongylocentrotus_purpuratus=5;

ophiura_lukteni=6;

ophiobolis_aculeata=7;

asterina_pectinifera=8;

florometra_serratissima=9;

gymnocrinus_richeri=10;

antedon_mediterranea=11;

katharina_tunicata = 12 { = outgroup2 }

{ THE CRINOIDEA GROUP IS FIXED to one of the 3 possible forms: }

R(gymnocrinus_richeri,florometra_serratissima);

Q x ( x<>florometra_serratissima => -R(gymnocrinus_richeri,x)

);

R(antedon_mediterranea,florometra_serratissima);

Q x ( x<>florometra_serratissima => -R(antedon_mediterranea,x)

);

{ PROPERTY P1: R(x, y) is not reflexive}

Q x (-R(x, x));

{ PROPERTY P2: R(x, y) is symetrical}

Q x y (R(x, y) => R(y, x));

{ PROPERTY P3: graph T is connected and acyclic (T is a tree) }

{

This property is verified by a constraint programmed in the model generator, instead of a "heavy" logical formula:

1- it will refuse the partial interpretations in which a connected component of the graph (in construction) is cyclic, i.e. such as: number of edges >= number of vertices

2- it will refuse the complete interpretations in which the constructed graph has more than one connected component

}

{ PROPERTY P4: graph T respects minimal distance matrix }

{

This property is verified by a constraint programmed in the model generator:

it will refuse the partial interpretations in which the graph (in construction) do not respect the minimal distance matrix, i.e. such as:

let x, y a couple of OTUs,

let d= minimal distance calculated between x and y (encoded in the minimal distance matrix), there is a a path of length k between x and y, with: k < d

The minimal distance matrix is encoded directly in the data structure of the model generator:

/* minimal distance matrix DEUTEROSTOMES taxB: */

DIST[0][0]=0;

DIST[1][0]=2; DIST[1][1]=0;

DIST[2][0]=2; DIST[2][1]=4; DIST[2][2]=0;

DIST[3][0]=1; DIST[3][1]=3; DIST[3][2]=3; DIST[3][3]=0;

DIST[4][0]=1; DIST[4][1]=3; DIST[4][2]=2; DIST[4][3]=1; DIST[4][4]=0;

DIST[5][0]=2; DIST[5][1]=4; DIST[5][2]=4; DIST[5][3]=3; DIST[5][4]=3; DIST[5][5]=0;

DIST[6][0]=3; DIST[6][1]=4; DIST[6][2]=4; DIST[6][3]=3; DIST[6][4]=3; DIST[6][5]=2; DIST[6][6]=0;

DIST[7][0]=2; DIST[7][1]=4; DIST[7][2]=4; DIST[7][3]=3; DIST[7][4]=3; DIST[7][5]=2; DIST[7][6]=1; DIST[7][7]=0;

DIST[8][0]=2; DIST[8][1]=4; DIST[8][2]=4; DIST[8][3]=3; DIST[8][4]=3; DIST[8][5]=1; DIST[8][6]=2; DIST[8][7]=1; DIST[8][8]=0;

DIST[9][0]=3; DIST[9][1]=5; DIST[9][2]=4; DIST[9][3]=4; DIST[9][4]=4; DIST[9][5]=1; DIST[9][6]=2; DIST[9][7]=2; DIST[9][8]=1; DIST[9][9]=0;

DIST[10][0]=3; DIST[10][1]=4; DIST[10][2]=4; DIST[10][3]=4; DIST[10][4]=4; DIST[10][5]=2; DIST[10][6]=3; DIST[10][7]=3; DIST[10][8]=2; DIST[10][9]=1; DIST[10][10]=0;

DIST[11][0]=3; DIST[11][1]=5; DIST[11][2]=4; DIST[11][3]=4; DIST[11][4]=4; DIST[11][5]=2; DIST[11][6]=3; DIST[11][7]=3; DIST[11][8]=2; DIST[11][9]=1; DIST[11][10]=1; DIST[11][11]=0;

DIST[12][0]=3; DIST[12][1]=4; DIST[12][2]=2; DIST[12][3]=3; DIST[12][4]=3; DIST[12][5]=5; DIST[12][6]=5; DIST[12][7]=5; DIST[12][8]=5; DIST[12][9]=5; DIST[12][10]=5; DIST[12][11]=5; DIST[12][12]=0;

}

{ PROPERTY P5: graph T respects eventual Primary Phylogenetic Hypotheses }

{

This property is verified by constraints programmed in the model generator:

- monophyly of Deuterostomia = (0,1,3,4,5,6,7,8,9,10,11)

- monophyly of Chordata = (0,1)

- monophyly of Ambulacria = (4,5,6,7,8,9,10,11)

- monophyly of Echinodermata = (5,6,7,8,9,10,11)

- monophyly of Ophiurida = (6,7)

- monophyly of Crinoidea = (9,10,11)

}

{------------------------------------------------------------------------------------------------------------------------}

{ PROPERTY P6: it is possible to calculate all the values for each HTU in the graph T }

{

First we calculate with the model generator the set of tree solutions which verify properties P1 to P5. Property P6 is verified *a posteriori* for each tree solution, with a *feedback* mechanism:

Studying each tree solution for calculating the values of HTUs, we eventually discover "impossible sub-trees": they appear in tree solutions which verify P1 to P5, but they do not verify P6.

For each impossible subtree A, an additional constraint is programmed into the model generator to forbid the solutions containing A. Tree solutions are recalculated and verified, allowing the discovery of new impossible subtrees and the programming of new constraints to recalculate the solutions (feedback mechanism). Finally, the complete set of optimal solutions is determined after iteration of this process and elimination of all the solutions that do not verify P6.

}

================================================================================

================================================================================

SOLUTIONS

================================================================================

================================================================================

OTUs:

homo_sapiens=0;

asymmetron_inferum=1;

limulus_polyphemus=2;

xenoturbella=3;

balanoglossus_carnosus=4;

strongylocentrotus_purpuratus=5;

ophiura_lukteni=6;

ophiobolis_aculeata=7;

asterina_pectinifera=8;

florometra_serratissima=9;

gymnocrinus_richeri=10;

antedon_mediterranea=11;

katharina_tunicata=12;

HTUs:

n1, n2, n3, n4

-> the crinoidea group is fixed (to one of the 3 possible forms)

D = [0,16]: 63 solutions OK (which verify property P6) (5 impossible sub-trees)

minimal score (best) = 50

maximal score = 108

-------------------------------------------------------------------------------------------------------------

score =84:

model 1:

-------------

R(0,3) R(0,4) R(0,n1) R(0,n2) R(0,n3) R(1,n2) R(2,n1) R(2,n4) R(3,0) R(4,0) R(5,8) R(5,9) R(5,n3) R(6,7) R(7,6) R(7,8) R(8,5) R(8,7) R(9,5) R(9,10) R(9,11) R(10,9) R(11,9) R(12,n4) R(n1,0) R(n1,2) R(n2,0) R(n2,1) R(n3,0) R(n3,5) R(n4,2) R(n4,12)

-------------------------------------------------------------------------------------------------------------

score =108:

model 2:

-------------

R(0,3) R(0,4) R(0,n1) R(0,n2) R(0,n3) R(1,n2) R(2,n1) R(2,n4) R(3,0) R(4,0) R(5,8) R(5,9) R(6,7) R(7,6) R(7,8) R(7,n3) R(8,5) R(8,7) R(9,5) R(9,10) R(9,11) R(10,9) R(11,9) R(12,n4) R(n1,0) R(n1,2) R(n2,0) R(n2,1) R(n3,0) R(n3,7) R(n4,2) R(n4,12)

-------------------------------------------------------------------------------------------------------------

score =90:

model 3:

-------------

R(0,3) R(0,4) R(0,n1) R(0,n2) R(0,n3) R(1,n2) R(2,n1) R(2,n4) R(3,0) R(4,0) R(5,8) R(5,9) R(6,7) R(7,6) R(7,8) R(8,5) R(8,7) R(8,n3) R(9,5) R(9,10) R(9,11) R(10,9) R(11,9) R(12,n4) R(n1,0) R(n1,2) R(n2,0) R(n2,1) R(n3,0) R(n3,8) R(n4,2) R(n4,12)

-------------------------------------------------------------------------------------------------------------

score =84:

model 4:

-------------

R(0,3) R(0,4) R(0,n1) R(0,n2) R(0,n3) R(1,n2) R(2,n4) R(3,0) R(4,0) R(5,8) R(5,9) R(5,n3) R(6,7) R(7,6) R(7,8) R(8,5) R(8,7) R(9,5) R(9,10) R(9,11) R(10,9) R(11,9) R(12,n4) R(n1,0) R(n1,n4) R(n2,0) R(n2,1) R(n3,0) R(n3,5) R(n4,2) R(n4,12) R(n4,n1)

-------------------------------------------------------------------------------------------------------------

score =108:

model 5:

-------------

R(0,3) R(0,4) R(0,n1) R(0,n2) R(0,n3) R(1,n2) R(2,n4) R(3,0) R(4,0) R(5,8) R(5,9) R(6,7) R(7,6) R(7,8) R(7,n3) R(8,5) R(8,7) R(9,5) R(9,10) R(9,11) R(10,9) R(11,9) R(12,n4) R(n1,0) R(n1,n4) R(n2,0) R(n2,1) R(n3,0) R(n3,7) R(n4,2) R(n4,12) R(n4,n1)

-------------------------------------------------------------------------------------------------------------

score =90:

model 6:

-------------

R(0,3) R(0,4) R(0,n1) R(0,n2) R(0,n3) R(1,n2) R(2,n4) R(3,0) R(4,0) R(5,8) R(5,9) R(6,7) R(7,6) R(7,8) R(8,5) R(8,7) R(8,n3) R(9,5) R(9,10) R(9,11) R(10,9) R(11,9) R(12,n4) R(n1,0) R(n1,n4) R(n2,0) R(n2,1) R(n3,0) R(n3,8) R(n4,2) R(n4,12) R(n4,n1)

-------------------------------------------------------------------------------------------------------------

score =64:

model 7:

-------------

R(0,3) R(0,4) R(0,n1) R(0,n2) R(0,n3) R(1,n2) R(2,n1) R(2,n4) R(3,0) R(4,0) R(5,8) R(5,9) R(5,n3) R(6,7) R(7,6) R(7,n3) R(8,5) R(9,5) R(9,10) R(9,11) R(10,9) R(11,9) R(12,n4) R(n1,0) R(n1,2) R(n2,0) R(n2,1) R(n3,0) R(n3,5) R(n3,7) R(n4,2) R(n4,12)

-------------------------------------------------------------------------------------------------------------

score =88:

model 8:

-------------

R(0,3) R(0,4) R(0,n1) R(0,n2) R(0,n3) R(1,n2) R(2,n1) R(2,n4) R(3,0) R(4,0) R(5,8) R(5,9) R(6,7) R(7,6) R(7,n3) R(8,5) R(8,n3) R(9,5) R(9,10) R(9,11) R(10,9) R(11,9) R(12,n4) R(n1,0) R(n1,2) R(n2,0) R(n2,1) R(n3,0) R(n3,7) R(n3,8) R(n4,2) R(n4,12)

score =64:

model 9:

-------------

R(0,3) R(0,4) R(0,n1) R(0,n2) R(0,n3) R(1,n2) R(2,n4) R(3,0) R(4,0) R(5,8) R(5,9) R(5,n3) R(6,7) R(7,6) R(7,n3) R(8,5) R(9,5) R(9,10) R(9,11) R(10,9) R(11,9) R(12,n4) R(n1,0) R(n1,n4) R(n2,0) R(n2,1) R(n3,0) R(n3,5) R(n3,7) R(n4,2) R(n4,12) R(n4,n1)

-------------------------------------------------------------------------------------------------------------

score =88:

model 10:

-------------

R(0,3) R(0,4) R(0,n1) R(0,n2) R(0,n3) R(1,n2) R(2,n4) R(3,0) R(4,0) R(5,8) R(5,9) R(6,7) R(7,6) R(7,n3) R(8,5) R(8,n3) R(9,5) R(9,10) R(9,11) R(10,9) R(11,9) R(12,n4) R(n1,0) R(n1,n4) R(n2,0) R(n2,1) R(n3,0) R(n3,7) R(n3,8) R(n4,2) R(n4,12) R(n4,n1)

-------------------------------------------------------------------------------------------------------------

score =96:

model 11:

-------------

R(0,3) R(0,4) R(0,n1) R(0,n2) R(0,n3) R(1,n2) R(2,n1) R(2,n4) R(3,0) R(4,0) R(5,8) R(5,n3) R(6,7) R(7,6) R(7,8) R(8,5) R(8,7) R(8,9) R(9,8) R(9,10) R(9,11) R(10,9) R(11,9) R(12,n4) R(n1,0) R(n1,2) R(n2,0) R(n2,1) R(n3,0) R(n3,5) R(n4,2) R(n4,12)

-------------------------------------------------------------------------------------------------------------

score =84:

model 12:

-------------

R(0,3) R(0,4) R(0,n1) R(0,n2) R(0,n3) R(1,n2) R(2,n1) R(2,n4) R(3,0) R(4,0) R(5,8) R(6,7) R(7,6) R(7,8) R(7,n3) R(8,5) R(8,7) R(8,9) R(9,8) R(9,10) R(9,11) R(10,9) R(11,9) R(12,n4) R(n1,0) R(n1,2) R(n2,0) R(n2,1) R(n3,0) R(n3,7) R(n4,2) R(n4,12)

-------------------------------------------------------------------------------------------------------------

score =66:

model 13:

-------------

R(0,3) R(0,4) R(0,n1) R(0,n2) R(0,n3) R(1,n2) R(2,n1) R(2,n4) R(3,0) R(4,0) R(5,8) R(6,7) R(7,6) R(7,8) R(8,5) R(8,7) R(8,9) R(8,n3) R(9,8) R(9,10) R(9,11) R(10,9) R(11,9) R(12,n4) R(n1,0) R(n1,2) R(n2,0) R(n2,1) R(n3,0) R(n3,8) R(n4,2) R(n4,12)

-------------------------------------------------------------------------------------------------------------

score =96:

model 14:

-------------

R(0,3) R(0,4) R(0,n1) R(0,n2) R(0,n3) R(1,n2) R(2,n4) R(3,0) R(4,0) R(5,8) R(5,n3) R(6,7) R(7,6) R(7,8) R(8,5) R(8,7) R(8,9) R(9,8) R(9,10) R(9,11) R(10,9) R(11,9) R(12,n4) R(n1,0) R(n1,n4) R(n2,0) R(n2,1) R(n3,0) R(n3,5) R(n4,2) R(n4,12) R(n4,n1)

-------------------------------------------------------------------------------------------------------------

score =84:

model 15:

-------------

R(0,3) R(0,4) R(0,n1) R(0,n2) R(0,n3) R(1,n2) R(2,n4) R(3,0) R(4,0) R(5,8) R(6,7) R(7,6) R(7,8) R(7,n3) R(8,5) R(8,7) R(8,9) R(9,8) R(9,10) R(9,11) R(10,9) R(11,9) R(12,n4) R(n1,0) R(n1,n4) R(n2,0) R(n2,1) R(n3,0) R(n3,7) R(n4,2) R(n4,12) R(n4,n1)

-------------------------------------------------------------------------------------------------------------

score =66:

model 16:

-------------

R(0,3) R(0,4) R(0,n1) R(0,n2) R(0,n3) R(1,n2) R(2,n4) R(3,0) R(4,0) R(5,8) R(6,7) R(7,6) R(7,8) R(8,5) R(8,7) R(8,9) R(8,n3) R(9,8) R(9,10) R(9,11) R(10,9) R(11,9) R(12,n4) R(n1,0) R(n1,n4) R(n2,0) R(n2,1) R(n3,0) R(n3,8) R(n4,2) R(n4,12) R(n4,n1)

-------------------------------------------------------------------------------------------------------------

score =88:

model 17:

-------------

R(0,3) R(0,4) R(0,n1) R(0,n2) R(0,n3) R(1,n2) R(2,n1) R(2,n4) R(3,0) R(4,0) R(5,8) R(5,n3) R(6,7) R(7,6) R(7,n3) R(8,5) R(8,9) R(9,8) R(9,10) R(9,11) R(10,9) R(11,9) R(12,n4) R(n1,0) R(n1,2) R(n2,0) R(n2,1) R(n3,0) R(n3,5) R(n3,7) R(n4,2) R(n4,12)

-------------------------------------------------------------------------------------------------------------

score =64:

model 18:

-------------

R(0,3) R(0,4) R(0,n1) R(0,n2) R(0,n3) R(1,n2) R(2,n1) R(2,n4) R(3,0) R(4,0) R(5,8) R(6,7) R(7,6) R(7,n3) R(8,5) R(8,9) R(8,n3) R(9,8) R(9,10) R(9,11) R(10,9) R(11,9) R(12,n4) R(n1,0) R(n1,2) R(n2,0) R(n2,1) R(n3,0) R(n3,7) R(n3,8) R(n4,2) R(n4,12)

-------------------------------------------------------------------------------------------------------------

score =88:

model 19:

-------------

R(0,3) R(0,4) R(0,n1) R(0,n2) R(0,n3) R(1,n2) R(2,n4) R(3,0) R(4,0) R(5,8) R(5,n3) R(6,7) R(7,6) R(7,n3) R(8,5) R(8,9) R(9,8) R(9,10) R(9,11) R(10,9) R(11,9) R(12,n4) R(n1,0) R(n1,n4) R(n2,0) R(n2,1) R(n3,0) R(n3,5) R(n3,7) R(n4,2) R(n4,12) R(n4,n1)

-------------------------------------------------------------------------------------------------------------

score =64:

model 20:

-------------

R(0,3) R(0,4) R(0,n1) R(0,n2) R(0,n3) R(1,n2) R(2,n4) R(3,0) R(4,0) R(5,8) R(6,7) R(7,6) R(7,n3) R(8,5) R(8,9) R(8,n3) R(9,8) R(9,10) R(9,11) R(10,9) R(11,9) R(12,n4) R(n1,0) R(n1,n4) R(n2,0) R(n2,1) R(n3,0) R(n3,7) R(n3,8) R(n4,2) R(n4,12) R(n4,n1)

-------------------------------------------------------------------------------------------------------------

score =96:

model 21:

-------------

R(0,3) R(0,4) R(0,n1) R(0,n2) R(0,n3) R(1,n2) R(2,n1) R(2,n4) R(3,0) R(4,0) R(5,9) R(5,n3) R(6,7) R(7,6) R(7,8) R(8,7) R(8,9) R(9,5) R(9,8) R(9,10) R(9,11) R(10,9) R(11,9) R(12,n4) R(n1,0) R(n1,2) R(n2,0) R(n2,1) R(n3,0) R(n3,5) R(n4,2) R(n4,12)

-------------------------------------------------------------------------------------------------------------

score =90:

model 22:

-------------

R(0,3) R(0,4) R(0,n1) R(0,n2) R(0,n3) R(1,n2) R(2,n1) R(2,n4) R(3,0) R(4,0) R(5,9) R(6,7) R(7,6) R(7,8) R(7,n3) R(8,7) R(8,9) R(9,5) R(9,8) R(9,10) R(9,11) R(10,9) R(11,9) R(12,n4) R(n1,0) R(n1,2) R(n2,0) R(n2,1) R(n3,0) R(n3,7) R(n4,2) R(n4,12)

-------------------------------------------------------------------------------------------------------------

score =72:

model 23:

-------------

R(0,3) R(0,4) R(0,n1) R(0,n2) R(0,n3) R(1,n2) R(2,n1) R(2,n4) R(3,0) R(4,0) R(5,9) R(6,7) R(7,6) R(7,8) R(8,7) R(8,9) R(8,n3) R(9,5) R(9,8) R(9,10) R(9,11) R(10,9) R(11,9) R(12,n4) R(n1,0) R(n1,2) R(n2,0) R(n2,1) R(n3,0) R(n3,8) R(n4,2) R(n4,12)

-------------------------------------------------------------------------------------------------------------

score =96:

model 24:

-------------

R(0,3) R(0,4) R(0,n1) R(0,n2) R(0,n3) R(1,n2) R(2,n4) R(3,0) R(4,0) R(5,9) R(5,n3) R(6,7) R(7,6) R(7,8) R(8,7) R(8,9) R(9,5) R(9,8) R(9,10) R(9,11) R(10,9) R(11,9) R(12,n4) R(n1,0) R(n1,n4) R(n2,0) R(n2,1) R(n3,0) R(n3,5) R(n4,2) R(n4,12) R(n4,n1)

-------------------------------------------------------------------------------------------------------------

score =90:

model 25:

-------------

R(0,3) R(0,4) R(0,n1) R(0,n2) R(0,n3) R(1,n2) R(2,n4) R(3,0) R(4,0) R(5,9) R(6,7) R(7,6) R(7,8) R(7,n3) R(8,7) R(8,9) R(9,5) R(9,8) R(9,10) R(9,11) R(10,9) R(11,9) R(12,n4) R(n1,0) R(n1,n4) R(n2,0) R(n2,1) R(n3,0) R(n3,7) R(n4,2) R(n4,12) R(n4,n1)

-------------------------------------------------------------------------------------------------------------

score =72:

model 26:

-------------

R(0,3) R(0,4) R(0,n1) R(0,n2) R(0,n3) R(1,n2) R(2,n4) R(3,0) R(4,0) R(5,9) R(6,7) R(7,6) R(7,8) R(8,7) R(8,9) R(8,n3) R(9,5) R(9,8) R(9,10) R(9,11) R(10,9) R(11,9) R(12,n4) R(n1,0) R(n1,n4) R(n2,0) R(n2,1) R(n3,0) R(n3,8) R(n4,2) R(n4,12) R(n4,n1)

-------------------------------------------------------------------------------------------------------------

score =68:

model 27:

-------------

R(0,3) R(0,4) R(0,n1) R(0,n2) R(0,n3) R(1,n2) R(2,n1) R(2,n4) R(3,0) R(4,0) R(5,9) R(5,n3) R(6,7) R(7,6) R(7,8) R(7,n3) R(8,7) R(9,5) R(9,10) R(9,11) R(10,9) R(11,9) R(12,n4) R(n1,0) R(n1,2) R(n2,0) R(n2,1) R(n3,0) R(n3,5) R(n3,7) R(n4,2) R(n4,12)

-------------------------------------------------------------------------------------------------------------

score =78:

model 28:

-------------

R(0,3) R(0,4) R(0,n1) R(0,n2) R(0,n3) R(1,n2) R(2,n1) R(2,n4) R(3,0) R(4,0) R(5,9) R(5,n3) R(6,7) R(7,6) R(7,8) R(8,7) R(8,n3) R(9,5) R(9,10) R(9,11) R(10,9) R(11,9) R(12,n4) R(n1,0) R(n1,2) R(n2,0) R(n2,1) R(n3,0) R(n3,5) R(n3,8) R(n4,2) R(n4,12)

-------------------------------------------------------------------------------------------------------------

score =68:

model 29:

-------------

R(0,3) R(0,4) R(0,n1) R(0,n2) R(0,n3) R(1,n1) R(2,n4) R(3,0) R(4,0) R(5,9) R(5,n2) R(6,7) R(7,6) R(7,8) R(7,n2) R(8,7) R(9,5) R(9,10) R(9,11) R(10,9) R(11,9) R(12,n4) R(n1,0) R(n1,1) R(n2,0) R(n2,5) R(n2,7) R(n3,0) R(n3,n4) R(n4,2) R(n4,12) R(n4,n3)

-------------------------------------------------------------------------------------------------------------

score =78:

model 30:

-------------

R(0,3) R(0,4) R(0,n1) R(0,n2) R(0,n3) R(1,n1) R(2,n4) R(3,0) R(4,0) R(5,9) R(5,n2) R(6,7) R(7,6) R(7,8) R(8,7) R(8,n2) R(9,5) R(9,10) R(9,11) R(10,9) R(11,9) R(12,n4) R(n1,0) R(n1,1) R(n2,0) R(n2,5) R(n2,8) R(n3,0) R(n3,n4) R(n4,2) R(n4,12) R(n4,n3)

-------------------------------------------------------------------------------------------------------------

score =70:

model 31:

-------------

R(0,3) R(0,4) R(0,n1) R(0,n2) R(0,n3) R(1,n2) R(2,n1) R(2,n4) R(3,0) R(4,0) R(5,9) R(5,n3) R(6,7) R(7,6) R(7,n3) R(8,9) R(9,5) R(9,8) R(9,10) R(9,11) R(10,9) R(11,9) R(12,n4) R(n1,0) R(n1,2) R(n2,0) R(n2,1) R(n3,0) R(n3,5) R(n3,7) R(n4,2) R(n4,12)

-------------------------------------------------------------------------------------------------------------

score =70:

model 32:

-------------

R(0,3) R(0,4) R(0,n1) R(0,n2) R(0,n3) R(1,n2) R(2,n1) R(2,n4) R(3,0) R(4,0) R(5,9) R(6,7) R(7,6) R(7,n3) R(8,9) R(8,n3) R(9,5) R(9,8) R(9,10) R(9,11) R(10,9) R(11,9) R(12,n4) R(n1,0) R(n1,2) R(n2,0) R(n2,1) R(n3,0) R(n3,7) R(n3,8) R(n4,2) R(n4,12)

-------------------------------------------------------------------------------------------------------------

score =70:

model 33:

-------------

R(0,3) R(0,4) R(0,n1) R(0,n2) R(0,n3) R(1,n2) R(2,n4) R(3,0) R(4,0) R(5,9) R(5,n3) R(6,7) R(7,6) R(7,n3) R(8,9) R(9,5) R(9,8) R(9,10) R(9,11) R(10,9) R(11,9) R(12,n4) R(n1,0) R(n1,n4) R(n2,0) R(n2,1) R(n3,0) R(n3,5) R(n3,7) R(n4,2) R(n4,12) R(n4,n1)

-------------------------------------------------------------------------------------------------------------

score =70:

model 34:

-------------

R(0,3) R(0,4) R(0,n1) R(0,n2) R(0,n3) R(1,n2) R(2,n4) R(3,0) R(4,0) R(5,9) R(6,7) R(7,6) R(7,n3) R(8,9) R(8,n3) R(9,5) R(9,8) R(9,10) R(9,11) R(10,9) R(11,9) R(12,n4) R(n1,0) R(n1,n4) R(n2,0) R(n2,1) R(n3,0) R(n3,7) R(n3,8) R(n4,2) R(n4,12) R(n4,n1)

-------------------------------------------------------------------------------------------------------------

score =60:

model 35:

-------------

R(0,3) R(0,4) R(0,n1) R(0,n2) R(0,n3) R(1,n2) R(2,n1) R(2,n4) R(3,0) R(4,0) R(5,9) R(5,n3) R(6,7) R(7,6) R(7,n3) R(8,n3) R(9,5) R(9,10) R(9,11) R(10,9) R(11,9) R(12,n4) R(n1,0) R(n1,2) R(n2,0) R(n2,1) R(n3,0) R(n3,5) R(n3,7) R(n3,8) R(n4,2) R(n4,12)

-------------------------------------------------------------------------------------------------------------

score =60:

model 36:

-------------

R(0,3) R(0,4) R(0,n1) R(0,n2) R(0,n3) R(1,n1) R(2,n4) R(3,0) R(4,0) R(5,9) R(5,n2) R(6,7) R(7,6) R(7,n2) R(8,n2) R(9,5) R(9,10) R(9,11) R(10,9) R(11,9) R(12,n4) R(n1,0) R(n1,1) R(n2,0) R(n2,5) R(n2,7) R(n2,8) R(n3,0) R(n3,n4) R(n4,2) R(n4,12) R(n4,n3)

-------------------------------------------------------------------------------------------------------------

score =80:

model 37:

-------------

R(0,3) R(0,4) R(0,n1) R(0,n2) R(0,n3) R(1,n2) R(2,n1) R(2,n4) R(3,0) R(4,0) R(5,n3) R(6,7) R(7,6) R(7,8) R(7,n3) R(8,7) R(8,9) R(9,8) R(9,10) R(9,11) R(10,9) R(11,9) R(12,n4) R(n1,0) R(n1,2) R(n2,0) R(n2,1) R(n3,0) R(n3,5) R(n3,7) R(n4,2) R(n4,12)

-------------------------------------------------------------------------------------------------------------

score =66:

model 38:

-------------

R(0,3) R(0,4) R(0,n1) R(0,n2) R(0,n3) R(1,n2) R(2,n1) R(2,n4) R(3,0) R(4,0) R(5,n3) R(6,7) R(7,6) R(7,8) R(8,7) R(8,9) R(8,n3) R(9,8) R(9,10) R(9,11) R(10,9) R(11,9) R(12,n4) R(n1,0) R(n1,2) R(n2,0) R(n2,1) R(n3,0) R(n3,5) R(n3,8) R(n4,2) R(n4,12)

-------------------------------------------------------------------------------------------------------------

score =80:

model 39:

-------------

R(0,3) R(0,4) R(0,n1) R(0,n2) R(0,n3) R(1,n2) R(2,n4) R(3,0) R(4,0) R(5,n3) R(6,7) R(7,6) R(7,8) R(7,n3) R(8,7) R(8,9) R(9,8) R(9,10) R(9,11) R(10,9) R(11,9) R(12,n4) R(n1,0) R(n1,n4) R(n2,0) R(n2,1) R(n3,0) R(n3,5) R(n3,7) R(n4,2) R(n4,12) R(n4,n1)

-------------------------------------------------------------------------------------------------------------

score =66:

model 40:

-------------

R(0,3) R(0,4) R(0,n1) R(0,n2) R(0,n3) R(1,n2) R(2,n4) R(3,0) R(4,0) R(5,n3) R(6,7) R(7,6) R(7,8) R(8,7) R(8,9) R(8,n3) R(9,8) R(9,10) R(9,11) R(10,9) R(11,9) R(12,n4) R(n1,0) R(n1,n4) R(n2,0) R(n2,1) R(n3,0) R(n3,5) R(n3,8) R(n4,2) R(n4,12) R(n4,n1)

-------------------------------------------------------------------------------------------------------------

score =60:

model 41:

-------------

R(0,3) R(0,4) R(0,n1) R(0,n2) R(0,n3) R(1,n2) R(2,n1) R(2,n4) R(3,0) R(4,0) R(5,n3) R(6,7) R(7,6) R(7,n3) R(8,9) R(8,n3) R(9,8) R(9,10) R(9,11) R(10,9) R(11,9) R(12,n4) R(n1,0) R(n1,2) R(n2,0) R(n2,1) R(n3,0) R(n3,5) R(n3,7) R(n3,8) R(n4,2) R(n4,12)

-------------------------------------------------------------------------------------------------------------

score =60:

model 42:

-------------

R(0,3) R(0,4) R(0,n1) R(0,n2) R(0,n3) R(1,n2) R(2,n4) R(3,0) R(4,0) R(5,n3) R(6,7) R(7,6) R(7,n3) R(8,9) R(8,n3) R(9,8) R(9,10) R(9,11) R(10,9) R(11,9) R(12,n4) R(n1,0) R(n1,n4) R(n2,0) R(n2,1) R(n3,0) R(n3,5) R(n3,7) R(n3,8) R(n4,2) R(n4,12) R(n4,n1)

-------------------------------------------------------------------------------------------------------------

score =74:

model 43:

-------------

R(0,3) R(0,4) R(0,n1) R(0,n2) R(0,n3) R(1,n1) R(2,n2) R(3,0) R(4,0) R(5,8) R(5,9) R(5,n3) R(6,7) R(7,6) R(7,8) R(8,5) R(8,7) R(9,5) R(9,10) R(9,11) R(10,9) R(11,9) R(12,n4) R(n1,0) R(n1,1) R(n2,0) R(n2,2) R(n2,n4) R(n3,0) R(n3,5) R(n4,12) R(n4,n2)

-------------------------------------------------------------------------------------------------------------

score =98:

model 44:

-------------

R(0,3) R(0,4) R(0,n1) R(0,n2) R(0,n3) R(1,n1) R(2,n2) R(3,0) R(4,0) R(5,8) R(5,9) R(6,7) R(7,6) R(7,8) R(7,n3) R(8,5) R(8,7) R(9,5) R(9,10) R(9,11) R(10,9) R(11,9) R(12,n4) R(n1,0) R(n1,1) R(n2,0) R(n2,2) R(n2,n4) R(n3,0) R(n3,7) R(n4,12) R(n4,n2)

-------------------------------------------------------------------------------------------------------------

score =80:

model 45:

-------------

R(0,3) R(0,4) R(0,n1) R(0,n2) R(0,n3) R(1,n1) R(2,n2) R(3,0) R(4,0) R(5,8) R(5,9) R(6,7) R(7,6) R(7,8) R(8,5) R(8,7) R(8,n3) R(9,5) R(9,10) R(9,11) R(10,9) R(11,9) R(12,n4) R(n1,0) R(n1,1) R(n2,0) R(n2,2) R(n2,n4) R(n3,0) R(n3,8) R(n4,12) R(n4,n2)

-------------------------------------------------------------------------------------------------------------

score =54:

model 46:

-------------

R(0,3) R(0,4) R(0,n1) R(0,n2) R(0,n3) R(1,n1) R(2,n2) R(3,0) R(4,0) R(5,8) R(5,9) R(5,n3) R(6,7) R(7,6) R(7,n3) R(8,5) R(9,5) R(9,10) R(9,11) R(10,9) R(11,9) R(12,n4) R(n1,0) R(n1,1) R(n2,0) R(n2,2) R(n2,n4) R(n3,0) R(n3,5) R(n3,7) R(n4,12) R(n4,n2)

-------------------------------------------------------------------------------------------------------------

score =78:

model 47:

-------------

R(0,3) R(0,4) R(0,n1) R(0,n2) R(0,n3) R(1,n1) R(2,n2) R(3,0) R(4,0) R(5,8) R(5,9) R(6,7) R(7,6) R(7,n3) R(8,5) R(8,n3) R(9,5) R(9,10) R(9,11) R(10,9) R(11,9) R(12,n4) R(n1,0) R(n1,1) R(n2,0) R(n2,2) R(n2,n4) R(n3,0) R(n3,7) R(n3,8) R(n4,12) R(n4,n2)

-------------------------------------------------------------------------------------------------------------

score =86:

model 48:

-------------

R(0,3) R(0,4) R(0,n1) R(0,n2) R(0,n3) R(1,n1) R(2,n2) R(3,0) R(4,0) R(5,8) R(5,n3) R(6,7) R(7,6) R(7,8) R(8,5) R(8,7) R(8,9) R(9,8) R(9,10) R(9,11) R(10,9) R(11,9) R(12,n4) R(n1,0) R(n1,1) R(n2,0) R(n2,2) R(n2,n4) R(n3,0) R(n3,5) R(n4,12) R(n4,n2)

-------------------------------------------------------------------------------------------------------------

score =74:

model 49:

-------------

R(0,3) R(0,4) R(0,n1) R(0,n2) R(0,n3) R(1,n1) R(2,n2) R(3,0) R(4,0) R(5,8) R(6,7) R(7,6) R(7,8) R(7,n3) R(8,5) R(8,7) R(8,9) R(9,8) R(9,10) R(9,11) R(10,9) R(11,9) R(12,n4) R(n1,0) R(n1,1) R(n2,0) R(n2,2) R(n2,n4) R(n3,0) R(n3,7) R(n4,12) R(n4,n2)

-------------------------------------------------------------------------------------------------------------

score =56:

model 50:

-------------

R(0,3) R(0,4) R(0,n1) R(0,n2) R(0,n3) R(1,n1) R(2,n2) R(3,0) R(4,0) R(5,8) R(6,7) R(7,6) R(7,8) R(8,5) R(8,7) R(8,9) R(8,n3) R(9,8) R(9,10) R(9,11) R(10,9) R(11,9) R(12,n4) R(n1,0) R(n1,1) R(n2,0) R(n2,2) R(n2,n4) R(n3,0) R(n3,8) R(n4,12) R(n4,n2)

-------------------------------------------------------------------------------------------------------------

score =78:

model 51:

-------------

R(0,3) R(0,4) R(0,n1) R(0,n2) R(0,n3) R(1,n1) R(2,n2) R(3,0) R(4,0) R(5,8) R(5,n3) R(6,7) R(7,6) R(7,n3) R(8,5) R(8,9) R(9,8) R(9,10) R(9,11) R(10,9) R(11,9) R(12,n4) R(n1,0) R(n1,1) R(n2,0) R(n2,2) R(n2,n4) R(n3,0) R(n3,5) R(n3,7) R(n4,12) R(n4,n2)

-------------------------------------------------------------------------------------------------------------

score =54:

model 52:

-------------

R(0,3) R(0,4) R(0,n1) R(0,n2) R(0,n3) R(1,n1) R(2,n2) R(3,0) R(4,0) R(5,8) R(6,7) R(7,6) R(7,n3) R(8,5) R(8,9) R(8,n3) R(9,8) R(9,10) R(9,11) R(10,9) R(11,9) R(12,n4) R(n1,0) R(n1,1) R(n2,0) R(n2,2) R(n2,n4) R(n3,0) R(n3,7) R(n3,8) R(n4,12) R(n4,n2)

-------------------------------------------------------------------------------------------------------------

score =86:

model 53:

-------------

R(0,3) R(0,4) R(0,n1) R(0,n2) R(0,n3) R(1,n1) R(2,n2) R(3,0) R(4,0) R(5,9) R(5,n3) R(6,7) R(7,6) R(7,8) R(8,7) R(8,9) R(9,5) R(9,8) R(9,10) R(9,11) R(10,9) R(11,9) R(12,n4) R(n1,0) R(n1,1) R(n2,0) R(n2,2) R(n2,n4) R(n3,0) R(n3,5) R(n4,12) R(n4,n2)

-------------------------------------------------------------------------------------------------------------

score =80:

model 54:

-------------

R(0,3) R(0,4) R(0,n1) R(0,n2) R(0,n3) R(1,n1) R(2,n2) R(3,0) R(4,0) R(5,9) R(6,7) R(7,6) R(7,8) R(7,n3) R(8,7) R(8,9) R(9,5) R(9,8) R(9,10) R(9,11) R(10,9) R(11,9) R(12,n4) R(n1,0) R(n1,1) R(n2,0) R(n2,2) R(n2,n4) R(n3,0) R(n3,7) R(n4,12) R(n4,n2)

-------------------------------------------------------------------------------------------------------------

score =62:

model 55:

-------------

R(0,3) R(0,4) R(0,n1) R(0,n2) R(0,n3) R(1,n1) R(2,n2) R(3,0) R(4,0) R(5,9) R(6,7) R(7,6) R(7,8) R(8,7) R(8,9) R(8,n3) R(9,5) R(9,8) R(9,10) R(9,11) R(10,9) R(11,9) R(12,n4) R(n1,0) R(n1,1) R(n2,0) R(n2,2) R(n2,n4) R(n3,0) R(n3,8) R(n4,12) R(n4,n2)

-------------------------------------------------------------------------------------------------------------

score =58:

model 56:

-------------

R(0,3) R(0,4) R(0,n1) R(0,n2) R(0,n3) R(1,n1) R(2,n2) R(3,0) R(4,0) R(5,9) R(5,n3) R(6,7) R(7,6) R(7,8) R(7,n3) R(8,7) R(9,5) R(9,10) R(9,11) R(10,9) R(11,9) R(12,n4) R(n1,0) R(n1,1) R(n2,0) R(n2,2) R(n2,n4) R(n3,0) R(n3,5) R(n3,7) R(n4,12) R(n4,n2)

-------------------------------------------------------------------------------------------------------------

score =68:

model 57:

-------------

R(0,3) R(0,4) R(0,n1) R(0,n2) R(0,n3) R(1,n1) R(2,n2) R(3,0) R(4,0) R(5,9) R(5,n3) R(6,7) R(7,6) R(7,8) R(8,7) R(8,n3) R(9,5) R(9,10) R(9,11) R(10,9) R(11,9) R(12,n4) R(n1,0) R(n1,1) R(n2,0) R(n2,2) R(n2,n4) R(n3,0) R(n3,5) R(n3,8) R(n4,12) R(n4,n2)

-------------------------------------------------------------------------------------------------------------

score =60:

model 58:

-------------

R(0,3) R(0,4) R(0,n1) R(0,n2) R(0,n3) R(1,n1) R(2,n2) R(3,0) R(4,0) R(5,9) R(5,n3) R(6,7) R(7,6) R(7,n3) R(8,9) R(9,5) R(9,8) R(9,10) R(9,11) R(10,9) R(11,9) R(12,n4) R(n1,0) R(n1,1) R(n2,0) R(n2,2) R(n2,n4) R(n3,0) R(n3,5) R(n3,7) R(n4,12) R(n4,n2)

-------------------------------------------------------------------------------------------------------------

Etat Ur-ambulacraires = 0

score =60:

model 59:

-------------

R(0,3) R(0,4) R(0,n1) R(0,n2) R(0,n3) R(1,n1) R(2,n2) R(3,0) R(4,0) R(5,9) R(6,7) R(7,6) R(7,n3) R(8,9) R(8,n3) R(9,5) R(9,8) R(9,10) R(9,11) R(10,9) R(11,9) R(12,n4) R(n1,0) R(n1,1) R(n2,0) R(n2,2) R(n2,n4) R(n3,0) R(n3,7) R(n3,8) R(n4,12) R(n4,n2)

-------------------------------------------------------------------------------------------------------------

-> the BEST model (1/2)

score =50:

model 60:

-------------

R(0,3) R(0,4) R(0,n1) R(0,n2) R(0,n3) R(1,n1) R(2,n2) R(3,0) R(4,0) R(5,9) R(5,n3) R(6,7) R(7,6) R(7,n3) R(8,n3) R(9,5) R(9,10) R(9,11) R(10,9) R(11,9) R(12,n4) R(n1,0) R(n1,1) R(n2,0) R(n2,2) R(n2,n4) R(n3,0) R(n3,5) R(n3,7) R(n3,8) R(n4,12) R(n4,n2)

-------------------------------------------------------------------------------------------------------------

score =70:

model 61:

-------------

R(0,3) R(0,4) R(0,n1) R(0,n2) R(0,n3) R(1,n1) R(2,n2) R(3,0) R(4,0) R(5,n3) R(6,7) R(7,6) R(7,8) R(7,n3) R(8,7) R(8,9) R(9,8) R(9,10) R(9,11) R(10,9) R(11,9) R(12,n4) R(n1,0) R(n1,1) R(n2,0) R(n2,2) R(n2,n4) R(n3,0) R(n3,5) R(n3,7) R(n4,12) R(n4,n2)

-------------------------------------------------------------------------------------------------------------

score =56:

model 62:

-------------

R(0,3) R(0,4) R(0,n1) R(0,n2) R(0,n3) R(1,n1) R(2,n2) R(3,0) R(4,0) R(5,n3) R(6,7) R(7,6) R(7,8) R(8,7) R(8,9) R(8,n3) R(9,8) R(9,10) R(9,11) R(10,9) R(11,9) R(12,n4) R(n1,0) R(n1,1) R(n2,0) R(n2,2) R(n2,n4) R(n3,0) R(n3,5) R(n3,8) R(n4,12) R(n4,n2)

-------------------------------------------------------------------------------------------------------------

-> the BEST model (2/2)

score =50:

model 63:

-------------

R(0,3) R(0,4) R(0,n1) R(0,n2) R(0,n3) R(1,n1) R(2,n2) R(3,0) R(4,0) R(5,n3) R(6,7) R(7,6) R(7,n3) R(8,9) R(8,n3) R(9,8) R(9,10) R(9,11) R(10,9) R(11,9) R(12,n4) R(n1,0) R(n1,1) R(n2,0) R(n2,2) R(n2,n4) R(n3,0) R(n3,5) R(n3,7) R(n3,8) R(n4,12) R(n4,n2)

-------------------------------------------------------------------------------------------------------------

-> no other models

deuterostomes_taxC_3sol

================================================================================

================================================================================

AXIOMS

================================================================================

================================================================================

{ the solutions of problem PHYLO are the smallest graphs T (defined on the smallest domain possible but containing at least all the OTUs) which verify properties P1 to P6:

P1- T is simple (the relation R(x, y) which defines graph T is not reflexive)

P2- T is non-oriented (the relation R(x, y) which defines graph T is symetrical)

P3- T is connected and acyclic (T is a tree)

P4- T respects the minimal distance matrix, i.e.:

for all couple of OTUs x and y, the length of the path X->Y in T is always superior or equals to the minimal distance calculated between x and y (encoded in the minimal distance matrix)

P5- T respects other eventual hypothesis (Primary Phylogenetic Hypothesis = PPH)

used to impose the existence of given monophyletic groups

P6- it is possible to calculate all the values for each HTU in the graph T }

{ OTUs: }

homo_sapiens=0;

asymmetron_inferum=1;

limulus_polyphemus=2; { = outgroup1 }

xenoturbella=3;

balanoglossus_carnosus=4;

strongylocentrotus_purpuratus=5;

ophiura_lukteni=6;

ophiobolis_aculeata=7;

asterina_pectinifera=8;

florometra_serratissima=9;

gymnocrinus_richeri=10;

antedon_mediterranea=11;

katharina_tunicata = 12 { = outgroup2 }

tethya_actinia = 13 { = outgroup3 }

{ THE ECHINODERMATA GROUP IS FIXED to one of the 63 possible forms (consensus solution): }

R(ophiura_lukteni,ophiobolis_aculeata);

Q x ( x<>ophiobolis_aculeata => -R(ophiura_lukteni,x)

);

R(ophiobolis_aculeata,asterina_pectinifera);

R(ophiobolis_aculeata,ophiura_lukteni);

Q x ( (x<>asterina_pectinifera et x<>ophiura_lukteni)

=>

-R(ophiobolis_aculeata,x)

);

R(strongylocentrotus_purpuratus,asterina_pectinifera);

Q x ( x<>asterina_pectinifera => -R(strongylocentrotus_purpuratus,x)

);

R(florometra_serratissima,asterina_pectinifera);

R(florometra_serratissima,gymnocrinus_richeri);

R(florometra_serratissima, antedon_mediterranea);

Q x ( ( x<>asterina_pectinifera et

x<>gymnocrinus_richeri et

x<>antedon_mediterranea

)

=>

-R(florometra_serratissima,x)

);

R(gymnocrinus_richeri,florometra_serratissima);

Q x ( x<>florometra_serratissima => -R(gymnocrinus_richeri,x)

);

R(antedon_mediterranea,florometra_serratissima);

Q x ( x<>florometra_serratissima => -R(antedon_mediterranea,x)

);

{ PROPERTY P1: R(x, y) is not reflexive}

Q x (-R(x, x));

{ PROPERTY P2: R(x, y) is symetrical}

Q x y (R(x, y) => R(y, x));

{ PROPERTY P3: graph T is connected and acyclic (T is a tree) }

{

This property is verified by a constraint programmed in the model generator, instead of a "heavy" logical formula:

1- it will refuse the partial interpretations in which a connected component of the graph (in construction) is cyclic, i.e. such as: number of edges >= number of vertices

2- it will refuse the complete interpretations in which the constructed graph has more than one connected component

}

{ PROPERTY P4: graph T respects minimal distance matrix }

{

This property is verified by a constraint programmed in the model generator:

it will refuse the partial interpretations in which the graph (in construction) do not respect the minimal distance matrix, i.e. such as:

let x, y a couple of OTUs,

let d= minimal distance calculated between x and y (encoded in the minimal distance matrix), there is a a path of length k between x and y, with: k < d

The minimal distance matrix is encoded directly in the data structure of the model generator:

/* minimal distance matrix DEUTEROSTOMES taxC: */

DIST[0][0]=0;

DIST[1][0]=2; DIST[1][1]=0;

DIST[2][0]=2; DIST[2][1]=4; DIST[2][2]=0;

DIST[3][0]=1; DIST[3][1]=3; DIST[3][2]=3; DIST[3][3]=0;

DIST[4][0]=1; DIST[4][1]=3; DIST[4][2]=2; DIST[4][3]=1; DIST[4][4]=0;

DIST[5][0]=2; DIST[5][1]=4; DIST[5][2]=4; DIST[5][3]=3; DIST[5][4]=3; DIST[5][5]=0;

DIST[6][0]=3; DIST[6][1]=4; DIST[6][2]=4; DIST[6][3]=3; DIST[6][4]=3; DIST[6][5]=2; DIST[6][6]=0;

DIST[7][0]=2; DIST[7][1]=4; DIST[7][2]=4; DIST[7][3]=3; DIST[7][4]=3; DIST[7][5]=2; DIST[7][6]=1; DIST[7][7]=0;

DIST[8][0]=2; DIST[8][1]=4; DIST[8][2]=4; DIST[8][3]=3; DIST[8][4]=3; DIST[8][5]=1; DIST[8][6]=2; DIST[8][7]=1; DIST[8][8]=0;

DIST[9][0]=3; DIST[9][1]=5; DIST[9][2]=4; DIST[9][3]=4; DIST[9][4]=4; DIST[9][5]=1; DIST[9][6]=2; DIST[9][7]=2; DIST[9][8]=1; DIST[9][9]=0;

DIST[10][0]=3; DIST[10][1]=4; DIST[10][2]=4; DIST[10][3]=4; DIST[10][4]=4; DIST[10][5]=2; DIST[10][6]=3; DIST[10][7]=3; DIST[10][8]=2; DIST[10][9]=1; DIST[10][10]=0;

DIST[11][0]=3; DIST[11][1]=5; DIST[11][2]=4; DIST[11][3]=4; DIST[11][4]=4; DIST[11][5]=2; DIST[11][6]=3; DIST[11][7]=3; DIST[11][8]=2; DIST[11][9]=1; DIST[11][10]=1; DIST[11][11]=0;

DIST[12][0]=3; DIST[12][1]=4; DIST[12][2]=2; DIST[12][3]=3; DIST[12][4]=3; DIST[12][5]=5; DIST[12][6]=5; DIST[12][7]=5; DIST[12][8]=5; DIST[12][9]=5; DIST[12][10]=5; DIST[12][11]=5; DIST[12][12]=0;

DIST[13][0]=4; DIST[13][1]=5; DIST[13][2]=5; DIST[13][3]=4; DIST[13][4]=4; DIST[13][5]=5; DIST[13][6]=4; DIST[13][7]=4; DIST[13][8]=5; DIST[13][9]=5; DIST[13][10]=6; DIST[13][11]=5; DIST[13][12]=6; DIST[13][13]=0;

}

{ PROPERTY P5: graph T respects eventual Primary Phylogenetic Hypotheses }

{

This property is verified by constraints programmed in the model generator:

- monophyly of Deuterostomia = (0,1,3,4,5,6,7,8,9,10,11)

- monophyly of Chordata = (0,1)

- monophyly of Ambulacria = (4,5,6,7,8,9,10,11)

- monophyly of Echinodermata = (5,6,7,8,9,10,11)

- monophyly of Ophiurida = (6,7)

- monophyly of Crinoidea = (9,10,11)

}

{------------------------------------------------------------------------------------------------------------------------}

{ PROPERTY P6: it is possible to calculate all the values for each HTU in the graph T }

{

First we calculate with the model generator the set of tree solutions which verify properties P1 to P5. Property P6 is verified *a posteriori* for each tree solution, with a *feedback* mechanism:

Studying each tree solution for calculating the values of HTUs, we eventually discover "impossible sub-trees": they appear in tree solutions which verify P1 to P5, but they do not verify P6.

For each impossible subtree A, an additional constraint is programmed into the model generator to forbid the solutions containing A. Tree solutions are recalculated and verified, allowing the discovery of new impossible subtrees and the programming of new constraints to recalculate the solutions (feedback mechanism). Finally, the complete set of optimal solutions is determined after iteration of this process and elimination of all the solutions that do not verify P6.

}

================================================================================

================================================================================

SOLUTIONS

================================================================================

================================================================================

OTUs:

homo_sapiens=0;

asymmetron_inferum=1;

limulus_polyphemus=2;

xenoturbella=3;

balanoglossus_carnosus=4;

strongylocentrotus_purpuratus=5;

ophiura_lukteni=6;

ophiobolis_aculeata=7;

asterina_pectinifera=8;

florometra_serratissima=9;

gymnocrinus_richeri=10;

antedon_mediterranea=11;

katharina_tunicata=12;

tethya_actinia=13;

HTUs:

n1, n2, n3, n4, n5, n6, n7

-> the echinodermata group is fixed (to one of the 63 possible forms)

NOTE:

In every solution, we have to *insert* one mutation "loss of gene atp9" *at* *any position* *between tethya_actinia and homo_sapiens* (OTUs 13, 0): it is the most parsimonious possibility.

We can easily reconstruct the possible values for eventual ancestral states (with 16 genes) between tethya_actinia and the loss mutation.

D = [0,20]: 3 solutions OK (which verify property P6) (15 impossible sub-trees)

minimal score (best) = 78

maximal score = 89

-------------------------------------------------------------------------------------------------------------

-> form outgroup SOL1

n2(mod1)

[ cox1 cox2 atp8 atp6 cox3 nad3 -nad5 -nad4 -nad4L nad6 cob rrnS rrnL nad1 nad2 ]

score =78:

model 1:

-------------

R(0,3) R(0,4) R(0,n1) R(0,n2) R(0,n3) R(0,n4) R(1,n1) R(2,n2) R(3,0) R(4,0) R(5,8) R(6,7) R(7,6) R(7,8) R(8,5) R(8,7) R(8,9) R(8,n4) R(9,8) R(9,10) R(9,11) R(10,9) R(11,9) R(12,n6) R(13,n5) R(n1,0) R(n1,1) R(n2,0) R(n2,2) R(n2,n6) R(n3,0) R(n3,n7) R(n4,0) R(n4,8) R(n5,13) R(n5,n7) R(n6,12) R(n6,n2) R(n7,n3) R(n7,n5)

-------------------------------------------------------------------------------------------------------------

-> form outgroup SOL2

score = 89:

model 2:

-------------

R(0,3) R(0,4) R(0,n1) R(0,n2) R(0,n3) R(0,n4) R(1,n1) R(2,n2) R(2,n7) R(3,0) R(4,0) R(5,8) R(6,7) R(7,6) R(7,8) R(8,5) R(8,7) R(8,9) R(8,n3) R(9,8) R(9,10) R(9,11) R(10,9) R(11,9) R(12,n7) R(13,n5) R(n1,0) R(n1,1) R(n2,0) R(n2,2) R(n3,0) R(n3,8) R(n4,0) R(n4,n6) R(n5,13) R(n5,n6) R(n6,n4) R(n6,n5) R(n7,2) R(n7,12)

-------------------------------------------------------------------------------------------------------------

-> form outgroup SOL3

n7(mod3)

[ cox1 cox2 atp8 atp6 cox3 nad3 -nad5 -nad4 -nad4L -cob -nad6 -nad1 -rrnL -rrnS nad2 ]

score = 89:

model 3:

-------------

R(0,3) R(0,4) R(0,n1) R(0,n2) R(0,n3) R(0,n4) R(1,n1) R(2,n7) R(3,0) R(4,0) R(5,8) R(6,7) R(7,6) R(7,8) R(8,5) R(8,7) R(8,9) R(8,n2) R(9,8) R(9,10) R(9,11) R(10,9) R(11,9) R(12,n7) R(13,n5) R(n1,0) R(n1,1) R(n2,0) R(n2,8) R(n3,0) R(n3,n6) R(n4,0) R(n4,n7) R(n5,13) R(n5,n6) R(n6,n3) R(n6,n5) R(n7,2) R(n7,12) R(n7,n4)

-------------------------------------------------------------------------------------------------------------

-> no other models

deuterostomes_taxD_42sol

================================================================================

================================================================================

AXIOMS

================================================================================

================================================================================

{ the solutions of problem PHYLO are the smallest graphs T (defined on the smallest domain possible but containing at least all the OTUs) which verify properties P1 to P6:

P1- T is simple (the relation R(x, y) which defines graph T is not reflexive)

P2- T is non-oriented (the relation R(x, y) which defines graph T is symetrical)

P3- T is connected and acyclic (T is a tree)

P4- T respects the minimal distance matrix, i.e.:

for all couple of OTUs x and y, the length of the path x->y in T is always superior or equals to the minimal distance calculated between x and y (encoded in the minimal distance matrix)

P5- T respects other eventual hypothesis (Primary Phylogenetic Hypothesis = PPH)

used to impose the existence of given monophyletic groups

P6- it is possible to calculate all the values for each HTU in the graph T }

{ OTUs: }

homo_sapiens=0;

asymmetron_inferum=1;

priapulus_caudatus=2; { = outgroup1 }

xenoturbella=3;

balanoglossus_carnosus=4;

strongylocentrotus_purpuratus=5;

ophiura_lukteni=6;

ophiobolis_aculeata=7;

asterina_pectinifera=8;

florometra_serratissima=9;

gymnocrinus_richeri=10;

antedon_mediterranea=11;

katharina_tunicata = 12 { = outgroup2 }

{ THE CRINOIDEA GROUP IS FIXED to one of the 3 possible forms: }

R(gymnocrinus_richeri,florometra_serratissima);

Q x ( x<>florometra_serratissima => -R(gymnocrinus_richeri,x)

);

R(antedon_mediterranea,florometra_serratissima);

Q x ( x<>florometra_serratissima => -R(antedon_mediterranea,x)

);

{ PROPERTY P1: R(x, y) is not reflexive}

Q x (-R(x, x));

{ PROPERTY P2: R(x, y) is symetrical}

Q x y (R(x, y) => R(y, x));

{ PROPERTY P3: graph T is connected and acyclic (T is a tree) }

{

This property is verified by a constraint programmed in the model generator, instead of a "heavy" logical formula:

1- it will refuse the partial interpretations in which a connected component of the graph (in construction) is cyclic, i.e. such as: number of edges >= number of vertices

2- it will refuse the complete interpretations in which the constructed graph has more than one connected component

}

{ PROPERTY P4: graph T respects minimal distance matrix }

{

This property is verified by a constraint programmed in the model generator:

it will refuse the partial interpretations in which the graph (in construction) do not respect the minimal distance matrix, i.e. such as:

let x, y a couple of OTUs,

let d= minimal distance calculated between x and y (encoded in the minimal distance matrix), there is a a path of length k between x and y, with: k < d

The minimal distance matrix is encoded directly in the data structure of the model generator:

/* minimal distance matrix DEUTEROSTOMES taxD: */

DIST[0][0]=0;

DIST[1][0]=2; DIST[1][1]=0;

DIST[2][0]=2; DIST[2][1]=4; DIST[2][2]=0;

DIST[3][0]=1; DIST[3][1]=3; DIST[3][2]=3; DIST[3][3]=0;

DIST[4][0]=1; DIST[4][1]=3; DIST[4][2]=3; DIST[4][3]=1; DIST[4][4]=0;

DIST[5][0]=2; DIST[5][1]=4; DIST[5][2]=4; DIST[5][3]=3; DIST[5][4]=3; DIST[5][5]=0;

DIST[6][0]=3; DIST[6][1]=4; DIST[6][2]=4; DIST[6][3]=3; DIST[6][4]=3; DIST[6][5]=2; DIST[6][6]=0;

DIST[7][0]=2; DIST[7][1]=4; DIST[7][2]=4; DIST[7][3]=3; DIST[7][4]=3; DIST[7][5]=2; DIST[7][6]=1; DIST[7][7]=0;

DIST[8][0]=2; DIST[8][1]=4; DIST[8][2]=4; DIST[8][3]=3; DIST[8][4]=3; DIST[8][5]=1; DIST[8][6]=2; DIST[8][7]=1; DIST[8][8]=0;

DIST[9][0]=3; DIST[9][1]=5; DIST[9][2]=5; DIST[9][3]=4; DIST[9][4]=4; DIST[9][5]=1; DIST[9][6]=2; DIST[9][7]=2; DIST[9][8]=1; DIST[9][9]=0;

DIST[10][0]=3; DIST[10][1]=4; DIST[10][2]=5; DIST[10][3]=4; DIST[10][4]=4; DIST[10][5]=2; DIST[10][6]=3; DIST[10][7]=3; DIST[10][8]=2; DIST[10][9]=1; DIST[10][10]=0;

DIST[11][0]=3; DIST[11][1]=5; DIST[11][2]=5; DIST[11][3]=4; DIST[11][4]=4; DIST[11][5]=2; DIST[11][6]=3; DIST[11][7]=3; DIST[11][8]=2; DIST[11][9]=1; DIST[11][10]=1; DIST[11][11]=0;

DIST[12][0]=3; DIST[12][1]=4; DIST[12][2]=2; DIST[12][3]=3; DIST[12][4]=3; DIST[12][5]=5; DIST[12][6]=5; DIST[12][7]=5; DIST[12][8]=5; DIST[12][9]=5; DIST[12][10]=5; DIST[12][11]=5; DIST[12][12]=0;

}

{ PROPERTY P5: graph T respects eventual Primary Phylogenetic Hypotheses }

{

This property is verified by constraints programmed in the model generator:

- monophyly of Deuterostomia = (0,1,3,4,5,6,7,8,9,10,11)

- monophyly of Chordata = (0,1)

- monophyly of Ambulacria = (4,5,6,7,8,9,10,11)

- monophyly of Echinodermata = (5,6,7,8,9,10,11)

- monophyly of Ophiurida = (6,7)

- monophyly of Crinoidea = (9,10,11)

}

{------------------------------------------------------------------------------------------------------------------------}

{ PROPERTY P6: it is possible to calculate all the values for each HTU in the graph T }

{

First we calculate with the model generator the set of tree solutions which verify properties P1 to P5. Property P6 is verified *a posteriori* for each tree solution, with a *feedback* mechanism:

Studying each tree solution for calculating the values of HTUs, we eventually discover "impossible sub-trees": they appear in tree solutions which verify P1 to P5, but they do not verify P6.

For each impossible subtree A, an additional constraint is programmed into the model generator to forbid the solutions containing A. Tree solutions are recalculated and verified, allowing the discovery of new impossible subtrees and the programming of new constraints to recalculate the solutions (feedback mechanism). Finally, the complete set of optimal solutions is determined after iteration of this process and elimination of all the solutions that do not verify P6.

}

================================================================================

================================================================================

SOLUTIONS

================================================================================

================================================================================

OTUs:

homo_sapiens=0;

asymmetron_inferum=1;

priapulus_caudatus=2;

xenoturbella=3;

balanoglossus_carnosus=4;

strongylocentrotus_purpuratus=5;

ophiura_lukteni=6;

ophiobolis_aculeata=7;

asterina_pectinifera=8;

florometra_serratissima=9;

gymnocrinus_richeri=10;

antedon_mediterranea=11;

katharina_tunicata=12;

HTUs:

n1, n2, n3, n4

-> the crinoidea group is fixed (to one of the 3 possible forms)

D = [0,16]: 42 solutions OK (which verify property P6) (10 impossible sub-trees)

minimal score (best) = 46

maximal score = 104

-------------------------------------------------------------------------------------------------------------

score =80:

model 1:

-------------

R(0,3) R(0,4) R(0,n1) R(0,n2) R(0,n3) R(1,n2) R(2,n1) R(2,n4) R(3,0) R(4,0) R(5,8) R(5,9) R(5,n3) R(6,7) R(7,6) R(7,8) R(8,5) R(8,7) R(9,5) R(9,10) R(9,11) R(10,9) R(11,9) R(12,n4) R(n1,0) R(n1,2) R(n2,0) R(n2,1) R(n3,0) R(n3,5) R(n4,2) R(n4,12)

-------------------------------------------------------------------------------------------------------------

score = 104:

model 2:

-------------

R(0,3) R(0,4) R(0,n1) R(0,n2) R(0,n3) R(1,n2) R(2,n1) R(2,n4) R(3,0) R(4,0) R(5,8) R(5,9) R(6,7) R(7,6) R(7,8) R(7,n3) R(8,5) R(8,7) R(9,5) R(9,10) R(9,11) R(10,9) R(11,9) R(12,n4) R(n1,0) R(n1,2) R(n2,0) R(n2,1) R(n3,0) R(n3,7) R(n4,2) R(n4,12)

-------------------------------------------------------------------------------------------------------------

score = 86:

model 3:

-------------

R(0,3) R(0,4) R(0,n1) R(0,n2) R(0,n3) R(1,n2) R(2,n1) R(2,n4) R(3,0) R(4,0) R(5,8) R(5,9) R(6,7) R(7,6) R(7,8) R(8,5) R(8,7) R(8,n3) R(9,5) R(9,10) R(9,11) R(10,9) R(11,9) R(12,n4) R(n1,0) R(n1,2) R(n2,0) R(n2,1) R(n3,0) R(n3,8) R(n4,2) R(n4,12)

-------------------------------------------------------------------------------------------------------------

score = 60:

model 4:

-------------

R(0,3) R(0,4) R(0,n1) R(0,n2) R(0,n3) R(1,n2) R(2,n1) R(2,n4) R(3,0) R(4,0) R(5,8) R(5,9) R(5,n3) R(6,7) R(7,6) R(7,n3) R(8,5) R(9,5) R(9,10) R(9,11) R(10,9) R(11,9) R(12,n4) R(n1,0) R(n1,2) R(n2,0) R(n2,1) R(n3,0) R(n3,5) R(n3,7) R(n4,2) R(n4,12)

-------------------------------------------------------------------------------------------------------------

score = 84:

model 5:

-------------

R(0,3) R(0,4) R(0,n1) R(0,n2) R(0,n3) R(1,n2) R(2,n1) R(2,n4) R(3,0) R(4,0) R(5,8) R(5,9) R(6,7) R(7,6) R(7,n3) R(8,5) R(8,n3) R(9,5) R(9,10) R(9,11) R(10,9) R(11,9) R(12,n4) R(n1,0) R(n1,2) R(n2,0) R(n2,1) R(n3,0) R(n3,7) R(n3,8) R(n4,2) R(n4,12)

-------------------------------------------------------------------------------------------------------------

score = 92:

model 6:

-------------

R(0,3) R(0,4) R(0,n1) R(0,n2) R(0,n3) R(1,n2) R(2,n1) R(2,n4) R(3,0) R(4,0) R(5,8) R(5,n3) R(6,7) R(7,6) R(7,8) R(8,5) R(8,7) R(8,9) R(9,8) R(9,10) R(9,11) R(10,9) R(11,9) R(12,n4) R(n1,0) R(n1,2) R(n2,0) R(n2,1) R(n3,0) R(n3,5) R(n4,2) R(n4,12)

-------------------------------------------------------------------------------------------------------------

score = 80:

model 7:

-------------

R(0,3) R(0,4) R(0,n1) R(0,n2) R(0,n3) R(1,n2) R(2,n1) R(2,n4) R(3,0) R(4,0) R(5,8) R(6,7) R(7,6) R(7,8) R(7,n3) R(8,5) R(8,7) R(8,9) R(9,8) R(9,10) R(9,11) R(10,9) R(11,9) R(12,n4) R(n1,0) R(n1,2) R(n2,0) R(n2,1) R(n3,0) R(n3,7) R(n4,2) R(n4,12)

-------------------------------------------------------------------------------------------------------------

score = 62:

model 8:

-------------

R(0,3) R(0,4) R(0,n1) R(0,n2) R(0,n3) R(1,n2) R(2,n1) R(2,n4) R(3,0) R(4,0) R(5,8) R(6,7) R(7,6) R(7,8) R(8,5) R(8,7) R(8,9) R(8,n3) R(9,8) R(9,10) R(9,11) R(10,9) R(11,9) R(12,n4) R(n1,0) R(n1,2) R(n2,0) R(n2,1) R(n3,0) R(n3,8) R(n4,2) R(n4,12)

-------------------------------------------------------------------------------------------------------------

score = 84:

model 9:

-------------

R(0,3) R(0,4) R(0,n1) R(0,n2) R(0,n3) R(1,n2) R(2,n1) R(2,n4) R(3,0) R(4,0) R(5,8) R(5,n3) R(6,7) R(7,6) R(7,n3) R(8,5) R(8,9) R(9,8) R(9,10) R(9,11) R(10,9) R(11,9) R(12,n4) R(n1,0) R(n1,2) R(n2,0) R(n2,1) R(n3,0) R(n3,5) R(n3,7) R(n4,2) R(n4,12)

-------------------------------------------------------------------------------------------------------------

score = 60:

model 10:

-------------

R(0,3) R(0,4) R(0,n1) R(0,n2) R(0,n3) R(1,n2) R(2,n1) R(2,n4) R(3,0) R(4,0) R(5,8) R(6,7) R(7,6) R(7,n3) R(8,5) R(8,9) R(8,n3) R(9,8) R(9,10) R(9,11) R(10,9) R(11,9) R(12,n4) R(n1,0) R(n1,2) R(n2,0) R(n2,1) R(n3,0) R(n3,7) R(n3,8) R(n4,2) R(n4,12)

-------------------------------------------------------------------------------------------------------------

score = 92:

model 11:

-------------

R(0,3) R(0,4) R(0,n1) R(0,n2) R(0,n3) R(1,n2) R(2,n1) R(2,n4) R(3,0) R(4,0) R(5,9) R(5,n3) R(6,7) R(7,6) R(7,8) R(8,7) R(8,9) R(9,5) R(9,8) R(9,10) R(9,11) R(10,9) R(11,9) R(12,n4) R(n1,0) R(n1,2) R(n2,0) R(n2,1) R(n3,0) R(n3,5) R(n4,2) R(n4,12)

-------------------------------------------------------------------------------------------------------------

score = 86:

model 12:

-------------

R(0,3) R(0,4) R(0,n1) R(0,n2) R(0,n3) R(1,n2) R(2,n1) R(2,n4) R(3,0) R(4,0) R(5,9) R(6,7) R(7,6) R(7,8) R(7,n3) R(8,7) R(8,9) R(9,5) R(9,8) R(9,10) R(9,11) R(10,9) R(11,9) R(12,n4) R(n1,0) R(n1,2) R(n2,0) R(n2,1) R(n3,0) R(n3,7) R(n4,2) R(n4,12)

-------------------------------------------------------------------------------------------------------------

score = 68:

model 13:

-------------

R(0,3) R(0,4) R(0,n1) R(0,n2) R(0,n3) R(1,n2) R(2,n1) R(2,n4) R(3,0) R(4,0) R(5,9) R(6,7) R(7,6) R(7,8) R(8,7) R(8,9) R(8,n3) R(9,5) R(9,8) R(9,10) R(9,11) R(10,9) R(11,9) R(12,n4) R(n1,0) R(n1,2) R(n2,0) R(n2,1) R(n3,0) R(n3,8) R(n4,2) R(n4,12)

-------------------------------------------------------------------------------------------------------------

score = 64:

model 14:

-------------

R(0,3) R(0,4) R(0,n1) R(0,n2) R(0,n3) R(1,n2) R(2,n1) R(2,n4) R(3,0) R(4,0) R(5,9) R(5,n3) R(6,7) R(7,6) R(7,8) R(7,n3) R(8,7) R(9,5) R(9,10) R(9,11) R(10,9) R(11,9) R(12,n4) R(n1,0) R(n1,2) R(n2,0) R(n2,1) R(n3,0) R(n3,5) R(n3,7) R(n4,2) R(n4,12)

-------------------------------------------------------------------------------------------------------------

score = 74:

model 15:

-------------

R(0,3) R(0,4) R(0,n1) R(0,n2) R(0,n3) R(1,n2) R(2,n1) R(2,n4) R(3,0) R(4,0) R(5,9) R(5,n3) R(6,7) R(7,6) R(7,8) R(8,7) R(8,n3) R(9,5) R(9,10) R(9,11) R(10,9) R(11,9) R(12,n4) R(n1,0) R(n1,2) R(n2,0) R(n2,1) R(n3,0) R(n3,5) R(n3,8) R(n4,2) R(n4,12)

-------------------------------------------------------------------------------------------------------------

score = 66:

model 16:

-------------

R(0,3) R(0,4) R(0,n1) R(0,n2) R(0,n3) R(1,n2) R(2,n1) R(2,n4) R(3,0) R(4,0) R(5,9) R(5,n3) R(6,7) R(7,6) R(7,n3) R(8,9) R(9,5) R(9,8) R(9,10) R(9,11) R(10,9) R(11,9) R(12,n4) R(n1,0) R(n1,2) R(n2,0) R(n2,1) R(n3,0) R(n3,5) R(n3,7) R(n4,2) R(n4,12)

-------------------------------------------------------------------------------------------------------------

score = 66:

model 17:

-------------

R(0,3) R(0,4) R(0,n1) R(0,n2) R(0,n3) R(1,n2) R(2,n1) R(2,n4) R(3,0) R(4,0) R(5,9) R(6,7) R(7,6) R(7,n3) R(8,9) R(8,n3) R(9,5) R(9,8) R(9,10) R(9,11) R(10,9) R(11,9) R(12,n4) R(n1,0) R(n1,2) R(n2,0) R(n2,1) R(n3,0) R(n3,7) R(n3,8) R(n4,2) R(n4,12)

-------------------------------------------------------------------------------------------------------------

score = 56:

model 18:

-------------

R(0,3) R(0,4) R(0,n1) R(0,n2) R(0,n3) R(1,n2) R(2,n1) R(2,n4) R(3,0) R(4,0) R(5,9) R(5,n3) R(6,7) R(7,6) R(7,n3) R(8,n3) R(9,5) R(9,10) R(9,11) R(10,9) R(11,9) R(12,n4) R(n1,0) R(n1,2) R(n2,0) R(n2,1) R(n3,0) R(n3,5) R(n3,7) R(n3,8) R(n4,2) R(n4,12)

-------------------------------------------------------------------------------------------------------------

score = 76:

model 19:

-------------

R(0,3) R(0,4) R(0,n1) R(0,n2) R(0,n3) R(1,n2) R(2,n1) R(2,n4) R(3,0) R(4,0) R(5,n3) R(6,7) R(7,6) R(7,8) R(7,n3) R(8,7) R(8,9) R(9,8) R(9,10) R(9,11) R(10,9) R(11,9) R(12,n4) R(n1,0) R(n1,2) R(n2,0) R(n2,1) R(n3,0) R(n3,5) R(n3,7) R(n4,2) R(n4,12)

-------------------------------------------------------------------------------------------------------------

score = 62:

model 20:

-------------

R(0,3) R(0,4) R(0,n1) R(0,n2) R(0,n3) R(1,n2) R(2,n1) R(2,n4) R(3,0) R(4,0) R(5,n3) R(6,7) R(7,6) R(7,8) R(8,7) R(8,9) R(8,n3) R(9,8) R(9,10) R(9,11) R(10,9) R(11,9) R(12,n4) R(n1,0) R(n1,2) R(n2,0) R(n2,1) R(n3,0) R(n3,5) R(n3,8) R(n4,2) R(n4,12)

-------------------------------------------------------------------------------------------------------------

score = 56:

model 21:

-------------

R(0,3) R(0,4) R(0,n1) R(0,n2) R(0,n3) R(1,n2) R(2,n1) R(2,n4) R(3,0) R(4,0) R(5,n3) R(6,7) R(7,6) R(7,n3) R(8,9) R(8,n3) R(9,8) R(9,10) R(9,11) R(10,9) R(11,9) R(12,n4) R(n1,0) R(n1,2) R(n2,0) R(n2,1) R(n3,0) R(n3,5) R(n3,7) R(n3,8) R(n4,2) R(n4,12)

-------------------------------------------------------------------------------------------------------------

score = 70:

model 22:

-------------

R(0,3) R(0,4) R(0,n1) R(0,n2) R(0,n3) R(1,n1) R(2,n2) R(3,0) R(4,0) R(5,8) R(5,9) R(5,n3) R(6,7) R(7,6) R(7,8) R(8,5) R(8,7) R(9,5) R(9,10) R(9,11) R(10,9) R(11,9) R(12,n4) R(n1,0) R(n1,1) R(n2,0) R(n2,2) R(n2,n4) R(n3,0) R(n3,5) R(n4,12) R(n4,n2)

-------------------------------------------------------------------------------------------------------------

score = 94:

model 23:

-------------

R(0,3) R(0,4) R(0,n1) R(0,n2) R(0,n3) R(1,n1) R(2,n2) R(3,0) R(4,0) R(5,8) R(5,9) R(6,7) R(7,6) R(7,8) R(7,n3) R(8,5) R(8,7) R(9,5) R(9,10) R(9,11) R(10,9) R(11,9) R(12,n4) R(n1,0) R(n1,1) R(n2,0) R(n2,2) R(n2,n4) R(n3,0) R(n3,7) R(n4,12) R(n4,n2)

-------------------------------------------------------------------------------------------------------------

score = 76:

model 24:

-------------

R(0,3) R(0,4) R(0,n1) R(0,n2) R(0,n3) R(1,n1) R(2,n2) R(3,0) R(4,0) R(5,8) R(5,9) R(6,7) R(7,6) R(7,8) R(8,5) R(8,7) R(8,n3) R(9,5) R(9,10) R(9,11) R(10,9) R(11,9) R(12,n4) R(n1,0) R(n1,1) R(n2,0) R(n2,2) R(n2,n4) R(n3,0) R(n3,8) R(n4,12) R(n4,n2)

-------------------------------------------------------------------------------------------------------------

score = 50:

model 25:

-------------

R(0,3) R(0,4) R(0,n1) R(0,n2) R(0,n3) R(1,n1) R(2,n2) R(3,0) R(4,0) R(5,8) R(5,9) R(5,n3) R(6,7) R(7,6) R(7,n3) R(8,5) R(9,5) R(9,10) R(9,11) R(10,9) R(11,9) R(12,n4) R(n1,0) R(n1,1) R(n2,0) R(n2,2) R(n2,n4) R(n3,0) R(n3,5) R(n3,7) R(n4,12) R(n4,n2)

-------------------------------------------------------------------------------------------------------------

score = 74:

model 26:

-------------

R(0,3) R(0,4) R(0,n1) R(0,n2) R(0,n3) R(1,n1) R(2,n2) R(3,0) R(4,0) R(5,8) R(5,9) R(6,7) R(7,6) R(7,n3) R(8,5) R(8,n3) R(9,5) R(9,10) R(9,11) R(10,9) R(11,9) R(12,n4) R(n1,0) R(n1,1) R(n2,0) R(n2,2) R(n2,n4) R(n3,0) R(n3,7) R(n3,8) R(n4,12) R(n4,n2)

-------------------------------------------------------------------------------------------------------------

score = 82:

model 27:

-------------

R(0,3) R(0,4) R(0,n1) R(0,n2) R(0,n3) R(1,n1) R(2,n2) R(3,0) R(4,0) R(5,8) R(5,n3) R(6,7) R(7,6) R(7,8) R(8,5) R(8,7) R(8,9) R(9,8) R(9,10) R(9,11) R(10,9) R(11,9) R(12,n4) R(n1,0) R(n1,1) R(n2,0) R(n2,2) R(n2,n4) R(n3,0) R(n3,5) R(n4,12) R(n4,n2)

-------------------------------------------------------------------------------------------------------------

score = 70:

model 28:

-------------

R(0,3) R(0,4) R(0,n1) R(0,n2) R(0,n3) R(1,n1) R(2,n2) R(3,0) R(4,0) R(5,8) R(6,7) R(7,6) R(7,8) R(7,n3) R(8,5) R(8,7) R(8,9) R(9,8) R(9,10) R(9,11) R(10,9) R(11,9) R(12,n4) R(n1,0) R(n1,1) R(n2,0) R(n2,2) R(n2,n4) R(n3,0) R(n3,7) R(n4,12) R(n4,n2)

-------------------------------------------------------------------------------------------------------------

score = 52:

model 29:

-------------

R(0,3) R(0,4) R(0,n1) R(0,n2) R(0,n3) R(1,n1) R(2,n2) R(3,0) R(4,0) R(5,8) R(6,7) R(7,6) R(7,8) R(8,5) R(8,7) R(8,9) R(8,n3) R(9,8) R(9,10) R(9,11) R(10,9) R(11,9) R(12,n4) R(n1,0) R(n1,1) R(n2,0) R(n2,2) R(n2,n4) R(n3,0) R(n3,8) R(n4,12) R(n4,n2)

-------------------------------------------------------------------------------------------------------------

score = 74:

model 30:

-------------

R(0,3) R(0,4) R(0,n1) R(0,n2) R(0,n3) R(1,n1) R(2,n2) R(3,0) R(4,0) R(5,8) R(5,n3) R(6,7) R(7,6) R(7,n3) R(8,5) R(8,9) R(9,8) R(9,10) R(9,11) R(10,9) R(11,9) R(12,n4) R(n1,0) R(n1,1) R(n2,0) R(n2,2) R(n2,n4) R(n3,0) R(n3,5) R(n3,7) R(n4,12) R(n4,n2)

-------------------------------------------------------------------------------------------------------------

score = 50:

model 31:

-------------

R(0,3) R(0,4) R(0,n1) R(0,n2) R(0,n3) R(1,n1) R(2,n2) R(3,0) R(4,0) R(5,8) R(6,7) R(7,6) R(7,n3) R(8,5) R(8,9) R(8,n3) R(9,8) R(9,10) R(9,11) R(10,9) R(11,9) R(12,n4) R(n1,0) R(n1,1) R(n2,0) R(n2,2) R(n2,n4) R(n3,0) R(n3,7) R(n3,8) R(n4,12) R(n4,n2)

-------------------------------------------------------------------------------------------------------------

score = 82:

model 32:

-------------

R(0,3) R(0,4) R(0,n1) R(0,n2) R(0,n3) R(1,n1) R(2,n2) R(3,0) R(4,0) R(5,9) R(5,n3) R(6,7) R(7,6) R(7,8) R(8,7) R(8,9) R(9,5) R(9,8) R(9,10) R(9,11) R(10,9) R(11,9) R(12,n4) R(n1,0) R(n1,1) R(n2,0) R(n2,2) R(n2,n4) R(n3,0) R(n3,5) R(n4,12) R(n4,n2)

-------------------------------------------------------------------------------------------------------------

score = 76:

model 33:

-------------

R(0,3) R(0,4) R(0,n1) R(0,n2) R(0,n3) R(1,n1) R(2,n2) R(3,0) R(4,0) R(5,9) R(6,7) R(7,6) R(7,8) R(7,n3) R(8,7) R(8,9) R(9,5) R(9,8) R(9,10) R(9,11) R(10,9) R(11,9) R(12,n4) R(n1,0) R(n1,1) R(n2,0) R(n2,2) R(n2,n4) R(n3,0) R(n3,7) R(n4,12) R(n4,n2)

-------------------------------------------------------------------------------------------------------------

score = 58:

model 34:

-------------

R(0,3) R(0,4) R(0,n1) R(0,n2) R(0,n3) R(1,n1) R(2,n2) R(3,0) R(4,0) R(5,9) R(6,7) R(7,6) R(7,8) R(8,7) R(8,9) R(8,n3) R(9,5) R(9,8) R(9,10) R(9,11) R(10,9) R(11,9) R(12,n4) R(n1,0) R(n1,1) R(n2,0) R(n2,2) R(n2,n4) R(n3,0) R(n3,8) R(n4,12) R(n4,n2)

-------------------------------------------------------------------------------------------------------------

score = 54:

model 35:

-------------

R(0,3) R(0,4) R(0,n1) R(0,n2) R(0,n3) R(1,n1) R(2,n2) R(3,0) R(4,0) R(5,9) R(5,n3) R(6,7) R(7,6) R(7,8) R(7,n3) R(8,7) R(9,5) R(9,10) R(9,11) R(10,9) R(11,9) R(12,n4) R(n1,0) R(n1,1) R(n2,0) R(n2,2) R(n2,n4) R(n3,0) R(n3,5) R(n3,7) R(n4,12) R(n4,n2)

-------------------------------------------------------------------------------------------------------------

score = 64:

model 36:

-------------

R(0,3) R(0,4) R(0,n1) R(0,n2) R(0,n3) R(1,n1) R(2,n2) R(3,0) R(4,0) R(5,9) R(5,n3) R(6,7) R(7,6) R(7,8) R(8,7) R(8,n3) R(9,5) R(9,10) R(9,11) R(10,9) R(11,9) R(12,n4) R(n1,0) R(n1,1) R(n2,0) R(n2,2) R(n2,n4) R(n3,0) R(n3,5) R(n3,8) R(n4,12) R(n4,n2)

-------------------------------------------------------------------------------------------------------------

score = 56:

model 37:

-------------

R(0,3) R(0,4) R(0,n1) R(0,n2) R(0,n3) R(1,n1) R(2,n2) R(3,0) R(4,0) R(5,9) R(5,n3) R(6,7) R(7,6) R(7,n3) R(8,9) R(9,5) R(9,8) R(9,10) R(9,11) R(10,9) R(11,9) R(12,n4) R(n1,0) R(n1,1) R(n2,0) R(n2,2) R(n2,n4) R(n3,0) R(n3,5) R(n3,7) R(n4,12) R(n4,n2)

-------------------------------------------------------------------------------------------------------------

score = 56:

model 38:

-------------

R(0,3) R(0,4) R(0,n1) R(0,n2) R(0,n3) R(1,n1) R(2,n2) R(3,0) R(4,0) R(5,9) R(6,7) R(7,6) R(7,n3) R(8,9) R(8,n3) R(9,5) R(9,8) R(9,10) R(9,11) R(10,9) R(11,9) R(12,n4) R(n1,0) R(n1,1) R(n2,0) R(n2,2) R(n2,n4) R(n3,0) R(n3,7) R(n3,8) R(n4,12) R(n4,n2)

-------------------------------------------------------------------------------------------------------------

score = 46:

model 39:

-------------

R(0,3) R(0,4) R(0,n1) R(0,n2) R(0,n3) R(1,n1) R(2,n2) R(3,0) R(4,0) R(5,9) R(5,n3) R(6,7) R(7,6) R(7,n3) R(8,n3) R(9,5) R(9,10) R(9,11) R(10,9) R(11,9) R(12,n4) R(n1,0) R(n1,1) R(n2,0) R(n2,2) R(n2,n4) R(n3,0) R(n3,5) R(n3,7) R(n3,8) R(n4,12) R(n4,n2)

-------------------------------------------------------------------------------------------------------------

score = 66:

model 40:

-------------

R(0,3) R(0,4) R(0,n1) R(0,n2) R(0,n3) R(1,n1) R(2,n2) R(3,0) R(4,0) R(5,n3) R(6,7) R(7,6) R(7,8) R(7,n3) R(8,7) R(8,9) R(9,8) R(9,10) R(9,11) R(10,9) R(11,9) R(12,n4) R(n1,0) R(n1,1) R(n2,0) R(n2,2) R(n2,n4) R(n3,0) R(n3,5) R(n3,7) R(n4,12) R(n4,n2)

-------------------------------------------------------------------------------------------------------------

score = 52:

model 41:

-------------

R(0,3) R(0,4) R(0,n1) R(0,n2) R(0,n3) R(1,n1) R(2,n2) R(3,0) R(4,0) R(5,n3) R(6,7) R(7,6) R(7,8) R(8,7) R(8,9) R(8,n3) R(9,8) R(9,10) R(9,11) R(10,9) R(11,9) R(12,n4) R(n1,0) R(n1,1) R(n2,0) R(n2,2) R(n2,n4) R(n3,0) R(n3,5) R(n3,8) R(n4,12) R(n4,n2)

-------------------------------------------------------------------------------------------------------------

score = 46:

model 42:

-------------

R(0,3) R(0,4) R(0,n1) R(0,n2) R(0,n3) R(1,n1) R(2,n2) R(3,0) R(4,0) R(5,n3) R(6,7) R(7,6) R(7,n3) R(8,9) R(8,n3) R(9,8) R(9,10) R(9,11) R(10,9) R(11,9) R(12,n4) R(n1,0) R(n1,1) R(n2,0) R(n2,2) R(n2,n4) R(n3,0) R(n3,5) R(n3,7) R(n3,8) R(n4,12) R(n4,n2)

-------------------------------------------------------------------------------------------------------------

-> no other models

with_tRNA_crinoids_taxA_1sol

================================================================================

================================================================================

AXIOMS

================================================================================

================================================================================

{ the solutions of problem PHYLO are the smallest graphs T (defined on the smallest domain possible but containing at least all the OTUs) which verify properties P1 to P6:

P1- T is simple (the relation R(x, y) which defines graph T is not reflexive)

P2- T is non-oriented (the relation R(x, y) which defines graph T is symetrical)

P3- T is connected and acyclic (T is a tree)

P4- T respects the minimal distance matrix, i.e.:

for all couple of OTUs x and y, the length of the path x->y in T is always superior or equals to the minimal distance calculated between x and y (encoded in the minimal distance matrix)

P5- T respects other eventual hypothesis (Primary Phylogenetic Hypothesis = PPH)

used to impose the existence of given monophyletic groups

P6- it is possible to calculate all the values for each HTU in the graph T }

{ OTUs: }

asterina_pectinifera=0; { = outgroup }

florometra_serratissima=1;

gymnocrinus_richeri=2;

antedon_mediterranea=3;

{ PROPERTY P1: R(x, y) is not reflexive}

Q x (-R(x, x));

{ PROPERTY P2: R(x, y) is symetrical}

Q x y (R(x, y) => R(y, x));

{ PROPERTY P3: graph T is connected and acyclic (T is a tree) }

{

This property is verified by a constraint programmed in the model generator, instead of a "heavy" logical formula:

1- it will refuse the partial interpretations in which a connected component of the graph (in construction) is cyclic, i.e. such as: number of edges >= number of vertices

2- it will refuse the complete interpretations in which the constructed graph has more than one connected component

}

{ PROPERTY P4: graph T respects minimal distance matrix }

{

This property is verified by a constraint programmed in the model generator:

it will refuse the partial interpretations in which the graph (in construction) do not respect the minimal distance matrix, i.e. such as:

let x, y a couple of OTUs,

let d= minimal distance calculated between x and y (encoded in the minimal distance matrix), there is a a path of length k between x and y, with: k < d

The minimal distance matrix is encoded directly in the data structure of the model generator:

/* minimal distance matrix CRINOIDS_with_tRNAS taxA: */

DIST[0][0]=0;

DIST[1][0]=3; DIST[1][1]=0;

DIST[2][0]=4; DIST[2][1]=1; DIST[2][2]=0;

DIST[3][0]=6; DIST[3][1]=3; DIST[3][2]=4; DIST[3][3]=0;

}

{ PROPERTY P5: graph T respects eventual Primary Phylogenetic Hypotheses }

{

This property is verified by constraints programmed in the model generator:

- monophyly of Crinoidea = (1,2,3)

}

{------------------------------------------------------------------------------------------------------------------------}

{ PROPERTY P6: it is possible to calculate all the values for each HTU in the graph T }

{

First we calculate with the model generator the set of tree solutions which verify properties P1 to P5. Property P6 is verified *a posteriori* for each tree solution, with a *feedback* mechanism:

Studying each tree solution for calculating the values of HTUs, we eventually discover "impossible sub-trees": they appear in tree solutions which verify P1 to P5, but they do not verify P6.

For each impossible subtree A, an additional constraint is programmed into the model generator to forbid the solutions containing A. Tree solutions are recalculated and verified, allowing the discovery of new impossible subtrees and the programming of new constraints to recalculate the solutions (feedback mechanism). Finally, the complete set of optimal solutions is determined after iteration of this process and elimination of all the solutions that do not verify P6.

}

================================================================================

================================================================================

SOLUTIONS

================================================================================

================================================================================

OTUs:

asterina_pectinifera=0; { = outgroup }

florometra_serratissima=1;

gymnocrinus_richeri=2;

antedon_mediterranea=3;

HTUs:

n1, n2, n3, n4

D = [0,7]: 1 solution OK (which verify property P6) (0 impossible sub-trees)

minimal score (best) = 0

maximal score = 0

-------------------------------------------------------------------------------------------------------------

score = 0:

model 1:

-------------

R(0,n1) R(1,2) R(1,n3) R(1,n4) R(2,1) R(3,n2) R(n1,0) R(n1,n3) R(n2,3) R(n2,n4) R(n3,1) R(n3,n1) R(n4,1) R(n4,n2)

-------------------------------------------------------------------------------------------------------------

-> no other model

with_tRNA_crinoids_taxB_1sol

================================================================================

================================================================================

AXIOMS

================================================================================

================================================================================

{ the solutions of problem PHYLO are the smallest graphs T (defined on the smallest domain possible but containing at least all the OTUs) which verify properties P1 to P6:

P1- T is simple (the relation R(x, y) which defines graph T is not reflexive)

P2- T is non-oriented (the relation R(x, y) which defines graph T is symetrical)

P3- T is connected and acyclic (T is a tree)

P4- T respects the minimal distance matrix, i.e.:

for all couple of OTUs x and y, the length of the path x->y in T is always superior or equals to the minimal distance calculated between x and y (encoded in the minimal distance matrix)

P5- T respects other eventual hypothesis (Primary Phylogenetic Hypothesis = PPH)

used to impose the existence of given monophyletic groups

P6- it is possible to calculate all the values for each HTU in the graph T }

{ OTUs: }

strongylocentrotus_purpuratus=0; { = outgroup }

florometra_serratissima=1;

gymnocrinus_richeri=2;

antedon_mediterranea=3;

{ PROPERTY P1: R(x, y) is not reflexive}

Q x (-R(x, x));

{ PROPERTY P2: R(x, y) is symetrical}

Q x y (R(x, y) => R(y, x));

{ PROPERTY P3: graph T is connected and acyclic (T is a tree) }

{

This property is verified by a constraint programmed in the model generator, instead of a "heavy" logical formula:

1- it will refuse the partial interpretations in which a connected component of the graph (in construction) is cyclic, i.e. such as: number of edges >= number of vertices

2- it will refuse the complete interpretations in which the constructed graph has more than one connected component

}

{ PROPERTY P4: graph T respects minimal distance matrix }

{

This property is verified by a constraint programmed in the model generator:

it will refuse the partial interpretations in which the graph (in construction) do not respect the minimal distance matrix, i.e. such as:

let x, y a couple of OTUs,

let d= minimal distance calculated between x and y (encoded in the minimal distance matrix), there is a a path of length k between x and y, with: k < d

The minimal distance matrix is encoded directly in the data structure of the model generator:

/* minimal distance matrix CRINOIDS_with_tRNAS taxB: */

DIST[0][0]=0;

DIST[1][0]=3; DIST[1][1]=0;

DIST[2][0]=4; DIST[2][1]=1; DIST[2][2]=0;

DIST[3][0]=6; DIST[3][1]=3; DIST[3][2]=4; DIST[3][3]=0;

}

{ PROPERTY P5: graph T respects eventual Primary Phylogenetic Hypotheses }

{

This property is verified by constraints programmed in the model generator:

- monophyly of Crinoidea = (1,2,3)

}

{------------------------------------------------------------------------------------------------------------------------}

{ PROPERTY P6: it is possible to calculate all the values for each HTU in the graph T }

{

First we calculate with the model generator the set of tree solutions which verify properties P1 to P5. Property P6 is verified *a posteriori* for each tree solution, with a *feedback* mechanism:

Studying each tree solution for calculating the values of HTUs, we eventually discover "impossible sub-trees": they appear in tree solutions which verify P1 to P5, but they do not verify P6.

For each impossible subtree A, an additional constraint is programmed into the model generator to forbid the solutions containing A. Tree solutions are recalculated and verified, allowing the discovery of new impossible subtrees and the programming of new constraints to recalculate the solutions (feedback mechanism). Finally, the complete set of optimal solutions is determined after iteration of this process and elimination of all the solutions that do not verify P6.

}

================================================================================

================================================================================

SOLUTIONS

================================================================================

================================================================================

OTUs:

strongylocentrotus_purpuratus=0; { = outgroup }

florometra_serratissima=1;

gymnocrinus_richeri=2;

antedon_mediterranea=3;

HTUs:

n1, n2, n3, n4

D = [0,7]: 1 solution OK (which verify property P6) (0 impossible sub-trees)

minimal score (best) = 0

maximal score = 0

-------------------------------------------------------------------------------------------------------------

score = 0:

model 1:

-------------

R(0,n1) R(1,2) R(1,n3) R(1,n4) R(2,1) R(3,n2) R(n1,0) R(n1,n3) R(n2,3) R(n2,n4) R(n3,1) R(n3,n1) R(n4,1) R(n4,n2)

-------------------------------------------------------------------------------------------------------------

-> no other model

with_tRNA_eleutherozoa_taxA_2sol

================================================================================

================================================================================

AXIOMS

================================================================================

================================================================================

{ the solutions of problem PHYLO are the smallest graphs T (defined on the smallest domain possible but containing at least all the OTUs) which verify properties P1 to P6:

P1- T is simple (the relation R(x, y) which defines graph T is not reflexive)

P2- T is non-oriented (the relation R(x, y) which defines graph T is symetrical)

P3- T is connected and acyclic (T is a tree)

P4- T respects the minimal distance matrix, i.e.:

for all couple of OTUs x and y, the length of the path x->y in T is always superior or equals to the minimal distance calculated between x and y (encoded in the minimal distance matrix)

P5- T respects other eventual hypothesis (Primary Phylogenetic Hypothesis = PPH)

used to impose the existence of given monophyletic groups

P6- it is possible to calculate all the values for each HTU in the graph T }

{ OTUs: }

florometra_serratissima=0; { = OUTGROUP }

strongylocentrotus_purpuratus=1;

asterina_pectinifera=2;

cucumaria_miniata=3;

{ PROPERTY P1: R(x, y) is not reflexive}

Q x (-R(x, x));

{ PROPERTY P2: R(x, y) is symetrical}

Q x y (R(x, y) => R(y, x));

{ PROPERTY P3: graph T is connected and acyclic (T is a tree) }

{

This property is verified by a constraint programmed in the model generator, instead of a "heavy" logical formula:

1- it will refuse the partial interpretations in which a connected component of the graph (in construction) is cyclic, i.e. such as: number of edges >= number of vertices

2- it will refuse the complete interpretations in which the constructed graph has more than one connected component

}

{ PROPERTY P4: graph T respects minimal distance matrix }

{

This property is verified by a constraint programmed in the model generator:

it will refuse the partial interpretations in which the graph (in construction) do not respect the minimal distance matrix, i.e. such as:

let x, y a couple of OTUs,

let d= minimal distance calculated between x and y (encoded in the minimal distance matrix), there is a a path of length k between x and y, with: k < d

The minimal distance matrix is encoded directly in the data structure of the model generator:

/* minimal distance matrix Eleutherozoa_with_tRNAS taxA: */

DIST[0][0]=0;

DIST[1][0]=3; DIST[1][1]=0;

DIST[2][0]=3; DIST[2][1]=1; DIST[2][2]=0;

DIST[3][0]=8; DIST[3][1]=5; DIST[3][2]=6; DIST[3][3]=0;

}

{ PROPERTY P5: graph T respects eventual Primary Phylogenetic Hypotheses }

{

This property is verified by constraints programmed in the model generator:

- monophyly of Eleutherozoa = (1,2,3)

}

{------------------------------------------------------------------------------------------------------------------------}

{ PROPERTY P6: it is possible to calculate all the values for each HTU in the graph T }

{

First we calculate with the model generator the set of tree solutions which verify properties P1 to P5. Property P6 is verified *a posteriori* for each tree solution, with a *feedback* mechanism:

Studying each tree solution for calculating the values of HTUs, we eventually discover "impossible sub-trees": they appear in tree solutions which verify P1 to P5, but they do not verify P6.

For each impossible subtree A, an additional constraint is programmed into the model generator to forbid the solutions containing A. Tree solutions are recalculated and verified, allowing the discovery of new impossible subtrees and the programming of new constraints to recalculate the solutions (feedback mechanism). Finally, the complete set of optimal solutions is determined after iteration of this process and elimination of all the solutions that do not verify P6.

}

================================================================================

================================================================================

SOLUTIONS

================================================================================

================================================================================

OTUs:

florometra_serratissima=0; { = outgroup }

strongylocentrotus_purpuratus=1;

asterina_pectinifera=2;

cucumaria_miniata=3;

HTUs:

n1, n2, n3, n4, n5, n6

D = [0,9]: 2 solution OK (which verify property P6) (1 impossible sub-tree)

minimal score (best) = 1

maximal score = 2

-------------------------------------------------------------------------------------------------------------

score = 1:

model 1:

-------------

R(0,n1) R(1,2) R(1,n2) R(1,n3) R(2,1) R(3,n4) R(n1,0) R(n1,n2) R(n2,1) R(n2,n1) R(n3,1) R(n3,n5) R(n4,3) R(n4,n6) R(n5,n3) R(n5,n6) R(n6,n4) R(n6,n5)

-------------------------------------------------------------------------------------------------------------

score = 2:

model 2:

-------------

R(0,n1) R(1,2) R(1,n3) R(2,1) R(2,n2) R(3,n4) R(n1,0) R(n1,n2) R(n2,2) R(n2,n1) R(n3,1) R(n3,n5) R(n4,3) R(n4,n6) R(n5,n3) R(n5,n6) R(n6,n4) R(n6,n5)

-------------------------------------------------------------------------------------------------------------

-> no other model

with_tRNA_ophiurida_taxA_1sol

================================================================================

================================================================================

AXIOMS

================================================================================

================================================================================

{ the solutions of problem PHYLO are the smallest graphs T (defined on the smallest domain possible but containing at least all the OTUs) which verify properties P1 to P6:

P1- T is simple (the relation R(x, y) which defines graph T is not reflexive)

P2- T is non-oriented (the relation R(x, y) which defines graph T is symetrical)

P3- T is connected and acyclic (T is a tree)

P4- T respects the minimal distance matrix, i.e.:

for all couple of OTUs x and y, the length of the path x->y in T is always superior or equals to the minimal distance calculated between x and y (encoded in the minimal distance matrix)

P5- T respects other eventual hypothesis (Primary Phylogenetic Hypothesis = PPH)

used to impose the existence of given monophyletic groups

P6- it is possible to calculate all the values for each HTU in the graph T }

{ OTUs: }

asterina_pectinifera=0;

ophiobolis_aculeata=1;

ophiura_lukteni=2;

strongylocentrotus_purpuratus=3; { = outgroup }

{ PROPERTY P1: R(x, y) is not reflexive}

Q x (-R(x, x));

{ PROPERTY P2: R(x, y) is symetrical}

Q x y (R(x, y) => R(y, x));

{ PROPERTY P3: graph T is connected and acyclic (T is a tree) }

{

This property is verified by a constraint programmed in the model generator, instead of a "heavy" logical formula:

1- it will refuse the partial interpretations in which a connected component of the graph (in construction) is cyclic, i.e. such as: number of edges >= number of vertices

2- it will refuse the complete interpretations in which the constructed graph has more than one connected component

}

{ PROPERTY P4: graph T respects minimal distance matrix }

{

This property is verified by a constraint programmed in the model generator:

it will refuse the partial interpretations in which the graph (in construction) do not respect the minimal distance matrix, i.e. such as:

let x, y a couple of OTUs,

let d= minimal distance calculated between x and y (encoded in the minimal distance matrix), there is a a path of length k between x and y, with: k < d

The minimal distance matrix is encoded directly in the data structure of the model generator:

/* minimal distance matrix OPHIURIDA_with_tRNAS taxA: */

DIST[0][0]=0;

DIST[1][0]=8; DIST[1][1]=0;

DIST[2][0]=7; DIST[2][1]=5; DIST[2][2]=0;

DIST[3][0]=1; DIST[3][1]=8; DIST[3][2]=8; DIST[3][3]=0;

}

{ PROPERTY P5: graph T respects eventual Primary Phylogenetic Hypotheses }

{

This property is verified by constraints programmed in the model generator:

- monophyly of Ophiurida = (1,2)

}

{------------------------------------------------------------------------------------------------------------------------}

{ PROPERTY P6: it is possible to calculate all the values for each HTU in the graph T }

{

First we calculate with the model generator the set of tree solutions which verify properties P1 to P5. Property P6 is verified *a posteriori* for each tree solution, with a *feedback* mechanism:

Studying each tree solution for calculating the values of HTUs, we eventually discover "impossible sub-trees": they appear in tree solutions which verify P1 to P5, but they do not verify P6.

For each impossible subtree A, an additional constraint is programmed into the model generator to forbid the solutions containing A. Tree solutions are recalculated and verified, allowing the discovery of new impossible subtrees and the programming of new constraints to recalculate the solutions (feedback mechanism). Finally, the complete set of optimal solutions is determined after iteration of this process and elimination of all the solutions that do not verify P6.

}

================================================================================

================================================================================

SOLUTIONS

================================================================================

================================================================================

OTUs:

asterina_pectinifera=0;

ophiobolis_aculeata=1;

ophiura_lukteni=2;

strongylocentrotus_purpuratus=3; { = outgroup }

HTUs:

n1, n2, n3, n4, n5, n6, n7, n8, n9, n10

D = [0,13]: AT LEAST 1 solution OK (which verify property P6) (8 impossible sub-trees)

NOTE:

This computation is NOT COMPLETE: it was impossible to verify property P6 for most of the solutions (untractable). In fact, it has been possible to verify only ONE solution (for property P6), so probably there are others solutions for this computation.

-------------------------------------------------------------------------------------------------------------

n4(mod1) = (6 possible values)

g1: [ cox1 R nad4L cox2 K atp8 atp6 cox3 -S2 nad3 nad4 H S1 nad5 -nad6 -G -rrnL -M -T -E C -V -Y d cob F rrnS P -Q N L2 nad1 I nad2 L1 -A W ]

g2: [ cox1 R nad4L cox2 K atp8 atp6 cox3 -S2 nad3 nad4 H S1 nad5 -nad6 -G -rrnL -M -T -E C -V -Y L2 nad1 I nad2 d cob F rrnS P -Q N L1 -A W ]

g3: [ cox1 R nad4L cox2 K atp8 atp6 cox3 -S2 nad3 nad4 H S1 nad5 -nad6 -nad2 -I -nad1 -L2 -N Q -P -rrnS -F -cob -D y V -C E T M rrnL G -L1 -A W ]

g4: [ cox1 R nad4L cox2 K atp8 atp6 cox3 -S2 nad3 nad4 H S1 nad5 -nad6 -G -rrnL -nad2 -I -nad1 -L2 -N Q -P -rrnS -F -cob -D y V -C E T M -L1 -A W ]

g5: [ cox1 R nad4L cox2 K atp8 atp6 cox3 -S2 nad3 nad4 H S1 nad5 -nad6 -G -rrnL -A -Y -L1 cob F rrnS P -Q N L2 nad1 I nad2 d -M V -C E T W ]

g6: [ cox1 R nad4L cox2 K atp8 atp6 cox3 -S2 nad3 nad4 H S1 nad5 -nad6 -G -rrnL -A -Y -L1 -N Q -P -rrnS -F -cob -D -nad2 -I -nad1 -L2 -M V -C E T W ]

model 1:

-------------

R(0,3) R(0,n1) R(1,n10) R(2,n8) R(3,0) R(n1,0) R(n1,n2) R(n2,n1) R(n2,n3) R(n3,n2) R(n3,n4) R(n4,n3) R(n4,n5) R(n4,n6) R(n5,n4) R(n5,n8) R(n6,n4) R(n6,n9) R(n7,n10) R(n7,n9) R(n8,2) R(n8,n5) R(n9,n6) R(n9,n7) R(n10,1) R(n10,n7)

-------------------------------------------------------------------------------------------------------------

-> perhaps there are other models
