# Supplementary material for: A complete logical approach to resolve the evolution and dynamics of mitochondrial genome in bilaterians
Source: PLoS One. 2018 Mar 16;13(3):e0194334. doi: 10.1371/journal.pone.0194334 (PMC5856267; doi:10.1371/journal.pone.0194334)
Supplement: S7 Appendix — (DOC) [file pone.0194334.s007.doc]

S7 Appendix. Axioms and solutions for Ecdysozoa.

ecdysozoans_taxA_9sol page 2

ecdysozoans_taxB_27sol page 8

ecdysozoans_taxC_39sol page 16

ecdysozoans_taxD_9sol page 33

ecdysozoans_taxE_9sol page 40

ecdysozoans_taxF_27sol page 46

ecdysozoans_taxG_7sol page 54

ecdysozoans_taxA_9sol

================================================================================

================================================================================

AXIOMS

================================================================================

================================================================================

{ the solutions of problem PHYLO are the smallest graphs T (defined on the smallest domain possible but containing at least all the OTUs) which verify properties P1 to P6:

P1- T is simple (the relation R(x, y) which defines graph T is not reflexive)

P2- T is non-oriented (the relation R(x, y) which defines graph T is symetrical)

P3- T is connected and acyclic (T is a tree)

P4- T respects the minimal distance matrix, i.e.:

for all couple of OTUs x and y, the length of the path x->y in T is always superior or equals to the minimal distance calculated between x and y (encoded in the minimal distance matrix)

P5- T respects other eventual hypothesis (Primary Phylogenetic Hypothesis = PPH)

used to impose the existence of given monophyletic groups

P6- it is possible to calculate all the values for each HTU in the graph T }

{ OTUs: }

narceus_annularis=0;

speleonectes_tulumensis=1;

argulus_americanus=2;

megabalanus_volcano=3;

eriocheir_sinensis=4;

chinkia_crosnieri=5;

pagurus_longicarpus=6;

vargula_hilgendorfi=7;

limulus_polyphemus=8;

nymphon_gracile=9; { = outgroup1 }

homo_sapiens=10; { = outgroup2 }

{ PROPERTY P1: R(x, y) is not reflexive}

Q x (-R(x, x));

{ PROPERTY P2: R(x, y) is symetrical}

Q x y (R(x, y) => R(y, x));

{ PROPERTY P3: graph T is connected and acyclic (T is a tree) }

{

This property is verified by a constraint programmed in the model generator, instead of a "heavy" logical formula:

1- it will refuse the partial interpretations in which a connected component of the graph (in construction) is cyclic, i.e. such as: number of edges >= number of vertices

2- it will refuse the complete interpretations in which the constructed graph has more than one connected component

}

{ PROPERTY P4: graph T respects minimal distance matrix }

{

This property is verified by a constraint programmed in the model generator:

it will refuse the partial interpretations in which the graph (in construction) do not respect the minimal distance matrix, i.e. such as:

Let x, y a couple of OTUs,

let d = minimal distance calculated between x and y (encoded in the minimal distance matrix), there is a path of length k between x and y, with: k < d

The minimal distance matrix is encoded directly in the data structure of the model generator:

/* minimal distance matrix ECDYSOZOANS taxA: */

DIST[0][0]=0;

DIST[1][0]=2; DIST[1][1]=0;

DIST[2][0]=2; DIST[2][1]=3; DIST[2][2]=0;

DIST[3][0]=1; DIST[3][1]=2; DIST[3][2]=2; DIST[3][3]=0;

DIST[4][0]=2; DIST[4][1]=2; DIST[4][2]=2; DIST[4][3]=2; DIST[4][4]=0;

DIST[5][0]=3; DIST[5][1]=3; DIST[5][2]=3; DIST[5][3]=3; DIST[5][4]=2; DIST[5][5]=0;

DIST[6][0]=3; DIST[6][1]=3; DIST[6][2]=4; DIST[6][3]=3; DIST[6][4]=3; DIST[6][5]=2; DIST[6][6]=0;

DIST[7][0]=3; DIST[7][1]=2; DIST[7][2]=4; DIST[7][3]=3; DIST[7][4]=3; DIST[7][5]=3; DIST[7][6]=3; DIST[7][7]=0;

DIST[8][0]=1; DIST[8][1]=1; DIST[8][2]=2; DIST[8][3]=1; DIST[8][4]=1; DIST[8][5]=2; DIST[8][6]=2; DIST[8][7]=2; DIST[8][8]=0;

DIST[9][0]=2; DIST[9][1]=2; DIST[9][2]=3; DIST[9][3]=2; DIST[9][4]=2; DIST[9][5]=2; DIST[9][6]=2; DIST[9][7]=3; DIST[9][8]=1; DIST[9][9]=0;

DIST[10][0]=2; DIST[10][1]=3; DIST[10][2]=2; DIST[10][3]=2; DIST[10][4]=2; DIST[10][5]=3; DIST[10][6]=4; DIST[10][7]=4; DIST[10][8]=2; DIST[10][9]=2; DIST[10][10]=0;

}

{ PROPERTY P5: graph T respects eventual Primary Phylogenetic Hypotheses }

{

This property is verified by constraints programmed in the model generator:

- monophyly of Arthropoda = (0,1,2,3,4,5,6,7,8,9)

- monophyly of Mandibulata = (0,1,2,3,4,5,6,7)

- monophyly of Crustacea = (1,2,3,4,5,6,7)

- monophyly of Decapoda = (4,5,6)

- monophyly of Chelicerata = (8,9)

}

{------------------------------------------------------------------------------------------------------------------------}

{ PROPERTY P6: it is possible to calculate all the values for each HTU in the graph T }

{

First we calculate with the model generator the set of tree solutions which verify properties P1 to P5. Property P6 is verified *a posteriori* for each tree solution, with a *feedback* mechanism:

Studying each tree solution for calculating the values of HTUs, we eventually discover "impossible sub-trees": they appear in tree solutions which verify P1 to P5, but they do not verify P6.

For each impossible subtree A, an additional constraint is programmed into the model generator to forbid the solutions containing A. Tree solutions are recalculated and verified, allowing the discovery of new impossible subtrees and the programming of new constraints to recalculate the solutions (feedback mechanism). Finally, the complete set of optimal solutions is determined after iteration of this process and elimination of all the solutions that do not verify P6.

}

================================================================================

================================================================================

SOLUTIONS

================================================================================

================================================================================

OTUs:

narceus_annularis=0;

speleonectes_tulumensis=1;

argulus_americanus=2;

megabalanus_volcano=3;

eriocheir_sinensis=4;

chinkia_crosnieri=5;

pagurus_longicarpus=6;

vargula_hilgendorfi=7;

limulus_polyphemus=8;

nymphon_gracile=9;

homo_sapiens=10;

HTUs:

n1, n2, n3, n4

D = [0,14]: 9 solutions OK (which verify property P6) (4 impossible sub-trees)

minimal score (best) = 18

maximal score = 34

-------------------------------------------------------------------------------------------------------------

n3(mod1)

[ cox1 cox2 nad2 atp8 atp6 cox3 nad3 -nad5 -nad4 -nad4L nad6 cob -nad1 -rrnL -rrnS ]

score = 34:

model 1:

-------------

R(0,8) R(1,8) R(1,n1) R(2,n4) R(3,8) R(3,n4) R(4,8) R(5,n3) R(6,n3) R(7,n1) R(8,0) R(8,1) R(8,3) R(8,4) R(8,9) R(8,n2) R(8,n3) R(9,8) R(10,n2) R(n1,1) R(n1,7) R(n2,8) R(n2,10) R(n3,5) R(n3,6) R(n3,8) R(n4,2) R(n4,3)

-------------------------------------------------------------------------------------------------------------

n3(mod2)

[ cox1 cox2 nad2 atp8 atp6 cox3 nad3 -nad5 -nad4 -nad4L nad6 cob -nad1 -rrnL -rrnS ]

score = 26:

model 2:

-------------

R(0,8) R(1,8) R(1,n1) R(2,n4) R(3,8) R(4,8) R(5,n3) R(6,n3) R(7,n1) R(8,0) R(8,1) R(8,3) R(8,4) R(8,9) R(8,n2) R(8,n3) R(8,n4) R(9,8) R(10,n2) R(n1,1) R(n1,7) R(n2,8) R(n2,10) R(n3,5) R(n3,6) R(n3,8) R(n4,2) R(n4,8)

-------------------------------------------------------------------------------------------------------------

n4(mod3)

[ cox1 cox2 nad2 atp8 atp6 cox3 nad3 -nad5 -nad4 -nad4L nad6 cob -nad1 -rrnL -rrnS ]

n3(mod3)

[ cox1 cox2 atp8 atp6 cox3 nad3 -nad6 nad4L nad4 nad5 cob -nad1 -rrnL -rrnS nad2 ]

score = 34:

model 3:

-------------

R(0,8) R(1,8) R(1,n1) R(2,n3) R(3,n3) R(4,8) R(5,n4) R(6,n4) R(7,n1) R(8,0) R(8,1) R(8,4) R(8,9) R(8,n2) R(8,n3) R(8,n4) R(9,8) R(10,n2) R(n1,1) R(n1,7) R(n2,8) R(n2,10) R(n3,2) R(n3,3) R(n3,8) R(n4,5) R(n4,6) R(n4,8)

-------------------------------------------------------------------------------------------------------------

n1(mod4)

[ cox1 cox2 atp8 atp6 cox3 nad3 -nad5 -nad4 -nad4L nad6 cob -rrnL -rrnS nad2 -nad1 ]

[ cox1 cox2 atp8 atp6 cox3 nad1 nad3 -nad5 -nad4 -nad4L nad6 cob -rrnL -rrnS nad2 ]

n3(mod4)

[ cox1 cox2 nad2 atp8 atp6 cox3 nad3 -nad5 -nad4 -nad4L nad6 cob -nad1 -rrnL -rrnS ]

score = 34:

model 4:

-------------

R(0,8) R(1,n1) R(2,n4) R(3,8) R(3,n4) R(4,8) R(5,n3) R(6,n3) R(7,n1) R(8,0) R(8,3) R(8,4) R(8,9) R(8,n1) R(8,n2) R(8,n3) R(9,8) R(10,n2) R(n1,1) R(n1,7) R(n1,8) R(n2,8) R(n2,10) R(n3,5) R(n3,6) R(n3,8) R(n4,2) R(n4,3)

-------------------------------------------------------------------------------------------------------------

n1(mod5)

[ cox1 cox2 atp8 atp6 cox3 nad3 -nad5 -nad4 -nad4L nad6 cob -rrnL -rrnS nad2 -nad1 ]

[ cox1 cox2 atp8 atp6 cox3 nad1 nad3 -nad5 -nad4 -nad4L nad6 cob -rrnL -rrnS nad2 ]

n3(mod5)

[ cox1 cox2 nad2 atp8 atp6 cox3 nad3 -nad5 -nad4 -nad4L nad6 cob -nad1 -rrnL -rrnS ]

score = 26:

model 5:

-------------

R(0,8) R(1,n1) R(2,n4) R(3,8) R(4,8) R(5,n3) R(6,n3) R(7,n1) R(8,0) R(8,3) R(8,4) R(8,9) R(8,n1) R(8,n2) R(8,n3) R(8,n4) R(9,8) R(10,n2) R(n1,1) R(n1,7) R(n1,8) R(n2,8) R(n2,10) R(n3,5) R(n3,6) R(n3,8) R(n4,2) R(n4,8)

-------------------------------------------------------------------------------------------------------------

n1(mod6)

[ cox1 cox2 atp8 atp6 cox3 nad3 -nad5 -nad4 -nad4L nad6 cob -rrnL -rrnS nad2 -nad1 ]

[ cox1 cox2 atp8 atp6 cox3 nad1 nad3 -nad5 -nad4 -nad4L nad6 cob -rrnL -rrnS nad2 ]

n3(mod6)

[ cox1 cox2 atp8 atp6 cox3 nad3 -nad6 nad4L nad4 nad5 cob -nad1 -rrnL -rrnS nad2 ]

n4(mod6)

[ cox1 cox2 nad2 atp8 atp6 cox3 nad3 -nad5 -nad4 -nad4L nad6 cob -nad1 -rrnL -rrnS ]

score = 34:

model 6:

-------------

R(0,8) R(1,n1) R(2,n3) R(3,n3) R(4,8) R(5,n4) R(6,n4) R(7,n1) R(8,0) R(8,4) R(8,9) R(8,n1) R(8,n2) R(8,n3) R(8,n4) R(9,8) R(10,n2) R(n1,1) R(n1,7) R(n1,8) R(n2,8) R(n2,10) R(n3,2) R(n3,3) R(n3,8) R(n4,5) R(n4,6) R(n4,8)

-------------------------------------------------------------------------------------------------------------

n3(mod7)

[ cox1 cox2 nad2 atp8 atp6 cox3 nad3 -nad5 -nad4 -nad4L nad6 cob -nad1 -rrnL -rrnS ]

score = 26:

model 7:

-------------

R(0,8) R(1,8) R(2,n1) R(3,8) R(3,n1) R(4,8) R(5,n3) R(6,n3) R(7,n4) R(8,0) R(8,1) R(8,3) R(8,4) R(8,9) R(8,n2) R(8,n3) R(8,n4) R(9,8) R(10,n2) R(n1,2) R(n1,3) R(n2,8) R(n2,10) R(n3,5) R(n3,6) R(n3,8) R(n4,7) R(n4,8)

-------------------------------------------------------------------------------------------------------------

-> the best model

n3(mod8)

[ cox1 cox2 nad2 atp8 atp6 cox3 nad3 -nad5 -nad4 -nad4L nad6 cob -nad1 -rrnL -rrnS ]

score = 18:

model 8:

-------------

R(0,8) R(1,8) R(2,n1) R(3,8) R(4,8) R(5,n3) R(6,n3) R(7,n4) R(8,0) R(8,1) R(8,3) R(8,4) R(8,9) R(8,n1) R(8,n2) R(8,n3) R(8,n4) R(9,8) R(10,n2) R(n1,2) R(n1,8) R(n2,8) R(n2,10) R(n3,5) R(n3,6) R(n3,8) R(n4,7) R(n4,8)

-------------------------------------------------------------------------------------------------------------

n3(mod9)

[ cox1 cox2 nad2 atp8 atp6 cox3 nad3 -nad5 -nad4 -nad4L nad6 cob -nad1 -rrnL -rrnS ]

n1(mod9)

[ cox1 cox2 atp8 atp6 cox3 nad3 -nad6 nad4L nad4 nad5 cob -nad1 -rrnL -rrnS nad2 ]

score = 26:

model 9:

-------------

R(0,8) R(1,8) R(2,n1) R(3,n1) R(4,8) R(5,n3) R(6,n3) R(7,n4) R(8,0) R(8,1) R(8,4) R(8,9) R(8,n1) R(8,n2) R(8,n3) R(8,n4) R(9,8) R(10,n2) R(n1,2) R(n1,3) R(n1,8) R(n2,8) R(n2,10) R(n3,5) R(n3,6) R(n3,8) R(n4,7) R(n4,8)

-------------------------------------------------------------------------------------------------------------

-> no other models

ecdysozoans_taxB_27sol

================================================================================

================================================================================

AXIOMS

================================================================================

================================================================================

{ the solutions of problem PHYLO are the smallest graphs T (defined on the smallest domain possible but containing at least all the OTUs) which verify properties P1 to P6:

P1- T is simple (the relation R(x, y) which defines graph T is not reflexive)

P2- T is non-oriented (the relation R(x, y) which defines graph T is symetrical)

P3- T is connected and acyclic (T is a tree)

P4- T respects the minimal distance matrix, i.e.:

for all couple of OTUs x and y, the length of the path x->y in T is always superior or equals to the minimal distance calculated between x and y (encoded in the minimal distance matrix)

P5- T respects other eventual hypothesis (Primary Phylogenetic Hypothesis = PPH)

used to impose the existence of given monophyletic groups

P6- it is possible to calculate all the values for each HTU in the graph T }

{ OTUs: }

narceus_annularis=0;

speleonectes_tulumensis=1;

argulus_americanus=2;

megabalanus_volcano=3;

eriocheir_sinensis=4;

chinkia_crosnieri=5;

pagurus_longicarpus=6;

vargula_hilgendorfi=7;

limulus_polyphemus=8;

nymphon_gracile=9; { = outgroup1 }

homo_sapiens=10; { = outgroup2 }

katharina_tunicata=11; { = outgroup3 }

{ PROPERTY P1: R(x, y) is not reflexive}

Q x (-R(x, x));

{ PROPERTY P2: R(x, y) is symetrical}

Q x y (R(x, y) => R(y, x));

{ PROPERTY P3: graph T is connected and acyclic (T is a tree) }

{

This property is verified by a constraint programmed in the model generator, instead of a "heavy" logical formula:

1- it will refuse the partial interpretations in which a connected component of the graph (in construction) is cyclic, i.e. such as: number of edges >= number of vertices

2- it will refuse the complete interpretations in which the constructed graph has more than one connected component

}

{ PROPERTY P4: graph T respects minimal distance matrix }

{

This property is verified by a constraint programmed in the model generator:

it will refuse the partial interpretations in which the graph (in construction) do not respect the minimal distance matrix, i.e. such as:

Let x, y a couple of OTUs,

let d = minimal distance calculated between x and y (encoded in the minimal distance matrix), there is a path of length k between x and y, with: k < d

The minimal distance matrix is encoded directly in the data structure of the model generator:

/* minimal distance matrix ECDYSOZOANS taxB: */

DIST[0][0]=0;

DIST[1][0]=2; DIST[1][1]=0;

DIST[2][0]=2; DIST[2][1]=3; DIST[2][2]=0;

DIST[3][0]=1; DIST[3][1]=2; DIST[3][2]=2; DIST[3][3]=0;

DIST[4][0]=2; DIST[4][1]=2; DIST[4][2]=2; DIST[4][3]=2; DIST[4][4]=0;

DIST[5][0]=3; DIST[5][1]=3; DIST[5][2]=3; DIST[5][3]=3; DIST[5][4]=2; DIST[5][5]=0;

DIST[6][0]=3; DIST[6][1]=3; DIST[6][2]=4; DIST[6][3]=3; DIST[6][4]=3; DIST[6][5]=2; DIST[6][6]=0;

DIST[7][0]=3; DIST[7][1]=2; DIST[7][2]=4; DIST[7][3]=3; DIST[7][4]=3; DIST[7][5]=3; DIST[7][6]=3; DIST[7][7]=0;

DIST[8][0]=1; DIST[8][1]=1; DIST[8][2]=2; DIST[8][3]=1; DIST[8][4]=1; DIST[8][5]=2; DIST[8][6]=2; DIST[8][7]=2; DIST[8][8]=0;

DIST[9][0]=2; DIST[9][1]=2; DIST[9][2]=3; DIST[9][3]=2; DIST[9][4]=2; DIST[9][5]=2; DIST[9][6]=2; DIST[9][7]=3; DIST[9][8]=1; DIST[9][9]=0;

DIST[10][0]=2; DIST[10][1]=3; DIST[10][2]=2; DIST[10][3]=2; DIST[10][4]=2; DIST[10][5]=3; DIST[10][6]=4; DIST[10][7]=4; DIST[10][8]=2; DIST[10][9]=2; DIST[10][10]=0;

DIST[11][0]=2; DIST[11][1]=3; DIST[11][2]=3; DIST[11][3]=2; DIST[11][4]=3; DIST[11][5]=4; DIST[11][6]=3; DIST[11][7]=4; DIST[11][8]=2; DIST[11][9]=3; DIST[11][10]=3; DIST[11][11]=0;

}

{ PROPERTY P5: graph T respects eventual Primary Phylogenetic Hypotheses }

{

This property is verified by constraints programmed in the model generator:

- monophyly of Arthropoda = (0,1,2,3,4,5,6,7,8,9)

- monophyly of Mandibulata = (0,1,2,3,4,5,6,7)

- monophyly of Crustacea = (1,2,3,4,5,6,7)

- monophyly of Decapoda = (4,5,6)

- monophyly of Chelicerata = (8,9)

}

{------------------------------------------------------------------------------------------------------------------------}

{ PROPERTY P6: it is possible to calculate all the values for each HTU in the graph T }

{

First we calculate with the model generator the set of tree solutions which verify properties P1 to P5. Property P6 is verified *a posteriori* for each tree solution, with a *feedback* mechanism:

Studying each tree solution for calculating the values of HTUs, we eventually discover "impossible sub-trees": they appear in tree solutions which verify P1 to P5, but they do not verify P6.

For each impossible subtree A, an additional constraint is programmed into the model generator to forbid the solutions containing A. Tree solutions are recalculated and verified, allowing the discovery of new impossible subtrees and the programming of new constraints to recalculate the solutions (feedback mechanism). Finally, the complete set of optimal solutions is determined after iteration of this process and elimination of all the solutions that do not verify P6.

}

================================================================================

================================================================================

SOLUTIONS

================================================================================

================================================================================

OTUs:

narceus_annularis=0;

speleonectes_tulumensis=1;

argulus_americanus=2;

megabalanus_volcano=3;

eriocheir_sinensis=4;

chinkia_crosnieri=5;

pagurus_longicarpus=6;

vargula_hilgendorfi=7;

limulus_polyphemus=8;

nymphon_gracile=9;

homo_sapiens=10;

katharina_tunicata=11;

HTUs:

n1, n2, n3, n4, n5

D = [0,16]: 27 solutions OK (which verify property P6) (4 impossible sub-trees)

minimal score (best) = 23

maximal score = 50

-------------------------------------------------------------------------------------------------------------

score = 50:

model 1:

-------------

R(0,8) R(1,8) R(1,n2) R(2,n3) R(3,8) R(3,n3) R(4,8) R(5,n1) R(6,n1) R(7,n2) R(8,0) R(8,1) R(8,3) R(8,4) R(8,9) R(8,n1) R(8,n4) R(9,8) R(10,n5) R(11,n4) R(n1,5) R(n1,6) R(n1,8) R(n2,1) R(n2,7) R(n3,2) R(n3,3) R(n4,8) R(n4,11) R(n4,n5) R(n5,10) R(n5,n4)

-------------------------------------------------------------------------------------------------------------

score = 50:

model 2:

-------------

R(0,8) R(1,8) R(1,n2) R(2,n3) R(3,8) R(3,n3) R(4,8) R(5,n1) R(6,n1) R(7,n2) R(8,0) R(8,1) R(8,3) R(8,4) R(8,9) R(8,n1) R(8,n5) R(9,8) R(10,n5) R(11,n4) R(n1,5) R(n1,6) R(n1,8) R(n2,1) R(n2,7) R(n3,2) R(n3,3) R(n4,11) R(n4,n5) R(n5,8) R(n5,10) R(n5,n4)

-------------------------------------------------------------------------------------------------------------

score = 41:

model 3:

-------------

R(0,8) R(1,8) R(1,n2) R(2,n3) R(3,8) R(3,n3) R(4,8) R(5,n1) R(6,n1) R(7,n2) R(8,0) R(8,1) R(8,3) R(8,4) R(8,9) R(8,n1) R(8,n4) R(8,n5) R(9,8) R(10,n5) R(11,n4) R(n1,5) R(n1,6) R(n1,8) R(n2,1) R(n2,7) R(n3,2) R(n3,3) R(n4,8) R(n4,11) R(n5,8) R(n5,10)

-------------------------------------------------------------------------------------------------------------

score = 41:

model 4:

-------------

R(0,8) R(1,8) R(1,n2) R(2,n3) R(3,8) R(4,8) R(5,n1) R(6,n1) R(7,n2) R(8,0) R(8,1) R(8,3) R(8,4) R(8,9) R(8,n1) R(8,n3) R(8,n4) R(9,8) R(10,n5) R(11,n4) R(n1,5) R(n1,6) R(n1,8) R(n2,1) R(n2,7) R(n3,2) R(n3,8) R(n4,8) R(n4,11) R(n4,n5) R(n5,10) R(n5,n4)

-------------------------------------------------------------------------------------------------------------

score = 41:

model 5:

-------------

R(0,8) R(1,8) R(1,n2) R(2,n3) R(3,8) R(4,8) R(5,n1) R(6,n1) R(7,n2) R(8,0) R(8,1) R(8,3) R(8,4) R(8,9) R(8,n1) R(8,n3) R(8,n5) R(9,8) R(10,n5) R(11,n4) R(n1,5) R(n1,6) R(n1,8) R(n2,1) R(n2,7) R(n3,2) R(n3,8) R(n4,11) R(n4,n5) R(n5,8) R(n5,10) R(n5,n4)

-------------------------------------------------------------------------------------------------------------

score = 32:

model 6:

-------------

R(0,8) R(1,8) R(1,n2) R(2,n3) R(3,8) R(4,8) R(5,n1) R(6,n1) R(7,n2) R(8,0) R(8,1) R(8,3) R(8,4) R(8,9) R(8,n1) R(8,n3) R(8,n4) R(8,n5) R(9,8) R(10,n5) R(11,n4) R(n1,5) R(n1,6) R(n1,8) R(n2,1) R(n2,7) R(n3,2) R(n3,8) R(n4,8) R(n4,11) R(n5,8) R(n5,10)

-------------------------------------------------------------------------------------------------------------

score = 50:

model 7:

-------------

R(0,8) R(1,8) R(1,n2) R(2,n3) R(3,n3) R(4,8) R(5,n1) R(6,n1) R(7,n2) R(8,0) R(8,1) R(8,4) R(8,9) R(8,n1) R(8,n3) R(8,n4) R(9,8) R(10,n4) R(11,n5) R(n1,5) R(n1,6) R(n1,8) R(n2,1) R(n2,7) R(n3,2) R(n3,3) R(n3,8) R(n4,8) R(n4,10) R(n4,n5) R(n5,11) R(n5,n4)

-------------------------------------------------------------------------------------------------------------

score = 50:

model 8:

-------------

R(0,8) R(1,8) R(1,n2) R(2,n3) R(3,n3) R(4,8) R(5,n1) R(6,n1) R(7,n2) R(8,0) R(8,1) R(8,4) R(8,9) R(8,n1) R(8,n3) R(8,n5) R(9,8) R(10,n4) R(11,n5) R(n1,5) R(n1,6) R(n1,8) R(n2,1) R(n2,7) R(n3,2) R(n3,3) R(n3,8) R(n4,10) R(n4,n5) R(n5,8) R(n5,11) R(n5,n4)

-------------------------------------------------------------------------------------------------------------

score = 41:

model 9:

-------------

R(0,8) R(1,8) R(1,n2) R(2,n3) R(3,n3) R(4,8) R(5,n1) R(6,n1) R(7,n2) R(8,0) R(8,1) R(8,4) R(8,9) R(8,n1) R(8,n3) R(8,n4) R(8,n5) R(9,8) R(10,n4) R(11,n5) R(n1,5) R(n1,6) R(n1,8) R(n2,1) R(n2,7) R(n3,2) R(n3,3) R(n3,8) R(n4,8) R(n4,10) R(n5,8) R(n5,11)

-------------------------------------------------------------------------------------------------------------

score = 50:

model 10:

-------------

R(0,8) R(1,n2) R(2,n3) R(3,8) R(3,n3) R(4,8) R(5,n1) R(6,n1) R(7,n2) R(8,0) R(8,3) R(8,4) R(8,9) R(8,n1) R(8,n2) R(8,n4) R(9,8) R(10,n4) R(11,n5) R(n1,5) R(n1,6) R(n1,8) R(n2,1) R(n2,7) R(n2,8) R(n3,2) R(n3,3) R(n4,8) R(n4,10) R(n4,n5) R(n5,11) R(n5,n4)

-------------------------------------------------------------------------------------------------------------

score = 50:

model 11:

-------------

R(0,8) R(1,n2) R(2,n3) R(3,8) R(3,n3) R(4,8) R(5,n1) R(6,n1) R(7,n2) R(8,0) R(8,3) R(8,4) R(8,9) R(8,n1) R(8,n2) R(8,n5) R(9,8) R(10,n4) R(11,n5) R(n1,5) R(n1,6) R(n1,8) R(n2,1) R(n2,7) R(n2,8) R(n3,2) R(n3,3) R(n4,10) R(n4,n5) R(n5,8) R(n5,11) R(n5,n4)

-------------------------------------------------------------------------------------------------------------

score = 41:

model 12:

-------------

R(0,8) R(1,n2) R(2,n3) R(3,8) R(3,n3) R(4,8) R(5,n1) R(6,n1) R(7,n2) R(8,0) R(8,3) R(8,4) R(8,9) R(8,n1) R(8,n2) R(8,n4) R(8,n5) R(9,8) R(10,n4) R(11,n5) R(n1,5) R(n1,6) R(n1,8) R(n2,1) R(n2,7) R(n2,8) R(n3,2) R(n3,3) R(n4,8) R(n4,10) R(n5,8) R(n5,11)

-------------------------------------------------------------------------------------------------------------

score = 41:

model 13:

-------------

R(0,8) R(1,n2) R(2,n3) R(3,8) R(4,8) R(5,n1) R(6,n1) R(7,n2) R(8,0) R(8,3) R(8,4) R(8,9) R(8,n1) R(8,n2) R(8,n3) R(8,n4) R(9,8) R(10,n4) R(11,n5) R(n1,5) R(n1,6) R(n1,8) R(n2,1) R(n2,7) R(n2,8) R(n3,2) R(n3,8) R(n4,8) R(n4,10) R(n4,n5) R(n5,11) R(n5,n4)

-------------------------------------------------------------------------------------------------------------

score = 41:

model 14:

-------------

R(0,8) R(1,n2) R(2,n3) R(3,8) R(4,8) R(5,n1) R(6,n1) R(7,n2) R(8,0) R(8,3) R(8,4) R(8,9) R(8,n1) R(8,n2) R(8,n3) R(8,n5) R(9,8) R(10,n4) R(11,n5) R(n1,5) R(n1,6) R(n1,8) R(n2,1) R(n2,7) R(n2,8) R(n3,2) R(n3,8) R(n4,10) R(n4,n5) R(n5,8) R(n5,11) R(n5,n4)

-------------------------------------------------------------------------------------------------------------

score = 32:

model 15:

-------------

R(0,8) R(1,n2) R(2,n3) R(3,8) R(4,8) R(5,n1) R(6,n1) R(7,n2) R(8,0) R(8,3) R(8,4) R(8,9) R(8,n1) R(8,n2) R(8,n3) R(8,n4) R(8,n5) R(9,8) R(10,n4) R(11,n5) R(n1,5) R(n1,6) R(n1,8) R(n2,1) R(n2,7) R(n2,8) R(n3,2) R(n3,8) R(n4,8) R(n4,10) R(n5,8) R(n5,11)

-------------------------------------------------------------------------------------------------------------

score = 50:

model 16:

-------------

R(0,8) R(1,n2) R(2,n3) R(3,n3) R(4,8) R(5,n1) R(6,n1) R(7,n2) R(8,0) R(8,4) R(8,9) R(8,n1) R(8,n2) R(8,n3) R(8,n4) R(9,8) R(10,n4) R(11,n5) R(n1,5) R(n1,6) R(n1,8) R(n2,1) R(n2,7) R(n2,8) R(n3,2) R(n3,3) R(n3,8) R(n4,8) R(n4,10) R(n4,n5) R(n5,11) R(n5,n4)

-------------------------------------------------------------------------------------------------------------

score = 50:

model 17:

-------------

R(0,8) R(1,n2) R(2,n3) R(3,n3) R(4,8) R(5,n1) R(6,n1) R(7,n2) R(8,0) R(8,4) R(8,9) R(8,n1) R(8,n2) R(8,n3) R(8,n5) R(9,8) R(10,n4) R(11,n5) R(n1,5) R(n1,6) R(n1,8) R(n2,1) R(n2,7) R(n2,8) R(n3,2) R(n3,3) R(n3,8) R(n4,10) R(n4,n5) R(n5,8) R(n5,11) R(n5,n4)

-------------------------------------------------------------------------------------------------------------

score = 41:

model 18:

-------------

R(0,8) R(1,n2) R(2,n3) R(3,n3) R(4,8) R(5,n1) R(6,n1) R(7,n2) R(8,0) R(8,4) R(8,9) R(8,n1) R(8,n2) R(8,n3) R(8,n4) R(8,n5) R(9,8) R(10,n4) R(11,n5) R(n1,5) R(n1,6) R(n1,8) R(n2,1) R(n2,7) R(n2,8) R(n3,2) R(n3,3) R(n3,8) R(n4,8) R(n4,10) R(n5,8) R(n5,11)

-------------------------------------------------------------------------------------------------------------

-> the best model

score = 23:

model 19:

-------------

R(0,8) R(1,8) R(2,n2) R(3,8) R(4,8) R(5,n1) R(6,n1) R(7,n3) R(8,0) R(8,1) R(8,3) R(8,4) R(8,9) R(8,n1) R(8,n2) R(8,n3) R(8,n4) R(8,n5) R(9,8) R(10,n4) R(11,n5) R(n1,5) R(n1,6) R(n1,8) R(n2,2) R(n2,8) R(n3,7) R(n3,8) R(n4,8) R(n4,10) R(n5,8) R(n5,11)

-------------------------------------------------------------------------------------------------------------

score = 32:

model 20:

-------------

R(0,8) R(1,8) R(2,n2) R(3,8) R(4,8) R(5,n1) R(6,n1) R(7,n3) R(8,0) R(8,1) R(8,3) R(8,4) R(8,9) R(8,n1) R(8,n2) R(8,n3) R(8,n4) R(9,8) R(10,n4) R(11,n5) R(n1,5) R(n1,6) R(n1,8) R(n2,2) R(n2,8) R(n3,7) R(n3,8) R(n4,8) R(n4,10) R(n4,n5) R(n5,11) R(n5,n4)

-------------------------------------------------------------------------------------------------------------

score = 32:

model 21:

-------------

R(0,8) R(1,8) R(2,n2) R(3,8) R(4,8) R(5,n1) R(6,n1) R(7,n3) R(8,0) R(8,1) R(8,3) R(8,4) R(8,9) R(8,n1) R(8,n2) R(8,n3) R(8,n4) R(9,8) R(10,n5) R(11,n4) R(n1,5) R(n1,6) R(n1,8) R(n2,2) R(n2,8) R(n3,7) R(n3,8) R(n4,8) R(n4,11) R(n4,n5) R(n5,10) R(n5,n4)

-------------------------------------------------------------------------------------------------------------

score = 41:

model 22:

-------------

R(0,8) R(1,8) R(2,n2) R(3,8) R(3,n2) R(4,8) R(5,n1) R(6,n1) R(7,n3) R(8,0) R(8,1) R(8,3) R(8,4) R(8,9) R(8,n1) R(8,n3) R(8,n4) R(9,8) R(10,n5) R(11,n4) R(n1,5) R(n1,6) R(n1,8) R(n2,2) R(n2,3) R(n3,7) R(n3,8) R(n4,8) R(n4,11) R(n4,n5) R(n5,10) R(n5,n4)

-------------------------------------------------------------------------------------------------------------

score = 41:

model 23:

-------------

R(0,8) R(1,8) R(2,n2) R(3,8) R(3,n2) R(4,8) R(5,n1) R(6,n1) R(7,n3) R(8,0) R(8,1) R(8,3) R(8,4) R(8,9) R(8,n1) R(8,n3) R(8,n5) R(9,8) R(10,n5) R(11,n4) R(n1,5) R(n1,6) R(n1,8) R(n2,2) R(n2,3) R(n3,7) R(n3,8) R(n4,11) R(n4,n5) R(n5,8) R(n5,10) R(n5,n4)

-------------------------------------------------------------------------------------------------------------

score = 32:

model 24:

-------------

R(0,8) R(1,8) R(2,n2) R(3,8) R(3,n2) R(4,8) R(5,n1) R(6,n1) R(7,n3) R(8,0) R(8,1) R(8,3) R(8,4) R(8,9) R(8,n1) R(8,n3) R(8,n4) R(8,n5) R(9,8) R(10,n5) R(11,n4) R(n1,5) R(n1,6) R(n1,8) R(n2,2) R(n2,3) R(n3,7) R(n3,8) R(n4,8) R(n4,11) R(n5,8) R(n5,10)

-------------------------------------------------------------------------------------------------------------

score = 41:

model 25:

-------------

R(0,8) R(1,8) R(2,n2) R(3,n2) R(4,8) R(5,n1) R(6,n1) R(7,n3) R(8,0) R(8,1) R(8,4) R(8,9) R(8,n1) R(8,n2) R(8,n3) R(8,n4) R(9,8) R(10,n5) R(11,n4) R(n1,5) R(n1,6) R(n1,8) R(n2,2) R(n2,3) R(n2,8) R(n3,7) R(n3,8) R(n4,8) R(n4,11) R(n4,n5) R(n5,10) R(n5,n4)

-------------------------------------------------------------------------------------------------------------

score = 41:

model 26:

-------------

R(0,8) R(1,8) R(2,n2) R(3,n2) R(4,8) R(5,n1) R(6,n1) R(7,n3) R(8,0) R(8,1) R(8,4) R(8,9) R(8,n1) R(8,n2) R(8,n3) R(8,n5) R(9,8) R(10,n5) R(11,n4) R(n1,5) R(n1,6) R(n1,8) R(n2,2) R(n2,3) R(n2,8) R(n3,7) R(n3,8) R(n4,11) R(n4,n5) R(n5,8) R(n5,10) R(n5,n4)

-------------------------------------------------------------------------------------------------------------

score = 32:

model 27:

-------------

R(0,8) R(1,8) R(2,n2) R(3,n2) R(4,8) R(5,n1) R(6,n1) R(7,n3) R(8,0) R(8,1) R(8,4) R(8,9) R(8,n1) R(8,n2) R(8,n3) R(8,n4) R(8,n5) R(9,8) R(10,n5) R(11,n4) R(n1,5) R(n1,6) R(n1,8) R(n2,2) R(n2,3) R(n2,8) R(n3,7) R(n3,8) R(n4,8) R(n4,11) R(n5,8) R(n5,10)

-------------------------------------------------------------------------------------------------------------

-> no other models

ecdysozoans_taxC_39sol

================================================================================

================================================================================

AXIOMS

================================================================================

================================================================================

{ the solutions of problem PHYLO are the smallest graphs T (defined on the smallest domain possible but containing at least all the OTUs) which verify properties P1 to P6:

P1- T is simple (the relation R(x, y) which defines graph T is not reflexive)

P2- T is non-oriented (the relation R(x, y) which defines graph T is symetrical)

P3- T is connected and acyclic (T is a tree)

P4- T respects the minimal distance matrix, i.e.:

for all couple of OTUs x and y, the length of the path x->y in T is always superior or equals to the minimal distance calculated between x and y (encoded in the minimal distance matrix)

P5- T respects other eventual hypothesis (Primary Phylogenetic Hypothesis = PPH)

used to impose the existence of given monophyletic groups

P6- it is possible to calculate all the values for each HTU in the graph T }

{ OTUs: }

narceus_annularis=0;

speleonectes_tulumensis=1;

argulus_americanus=2;

megabalanus_volcano=3;

eriocheir_sinensis=4;

chinkia_crosnieri=5;

pagurus_longicarpus=6;

vargula_hilgendorfi=7;

limulus_polyphemus=8;

nymphon_gracile=9; { = outgroup1 }

homo_sapiens=10; { = outgroup2 }

katharina_tunicata=11; { = outgroup3 }

ligia_oceanica=12;

{ AUXILLIARY CONSTANTS used to fix a part of the solution: }

G1=13; { used to fix the decapoda group }

G2=14;

G3=15; { used to fix the outgroup form SOL3 }

{ THE DECAPODA GROUP IS FIXED: }

R(limulus_polyphemus,eriocheir_sinensis);

Q x ( x<>limulus_polyphemus

=>

-R(eriocheir_sinensis,x)

);

R(limulus_polyphemus,G1);

R(chinkia_crosnieri,G1);

R(pagurus_longicarpus,G1);

Q x ( ( x<>limulus_polyphemus et

x<>chinkia_crosnieri et

x<>pagurus_longicarpus

)

=>

-R(G1,x)

);

Q x ( x<>G1

=>

-R(chinkia_crosnieri,x)

);

Q x ( x<>G1

=>

-R(pagurus_longicarpus,x)

);

{ THE OUTGROUP FORM "SOL3" IS FIXED: }

R(homo_sapiens,G2);

R(G2,G3);

R(G3,limulus_polyphemus);

R(G3,katharina_tunicata);

Q x ( x<>G2

=>

-R(homo_sapiens,x)

);

Q x ( ( x<>homo_sapiens et

x<>G3

)

=>

-R(G2,x)

);

Q x ( x<>G3

=>

-R(katharina_tunicata,x)

);

{ PROPERTY P1: R(x, y) is not reflexive}

Q x (-R(x, x));

{ PROPERTY P2: R(x, y) is symetrical}

Q x y (R(x, y) => R(y, x));

{ PROPERTY P3: graph T is connected and acyclic (T is a tree) }

{

This property is verified by a constraint programmed in the model generator, instead of a "heavy" logical formula:

1- it will refuse the partial interpretations in which a connected component of the graph (in construction) is cyclic, i.e. such as: number of edges >= number of vertices

2- it will refuse the complete interpretations in which the constructed graph has more than one connected component

}

{ PROPERTY P4: graph T respects minimal distance matrix }

{

This property is verified by a constraint programmed in the model generator:

it will refuse the partial interpretations in which the graph (in construction) do not respect the minimal distance matrix, i.e. such as:

Let x, y a couple of OTUs,

let d = minimal distance calculated between x and y (encoded in the minimal distance matrix), there is a path of length k between x and y, with: k < d

The minimal distance matrix is encoded directly in the data structure of the model generator:

/* minimal distance matrix ECDYSOZOANS taxC: */

DIST[0][0]=0;

DIST[1][0]=2; DIST[1][1]=0;

DIST[2][0]=2; DIST[2][1]=3; DIST[2][2]=0;

DIST[3][0]=1; DIST[3][1]=2; DIST[3][2]=2; DIST[3][3]=0;

DIST[4][0]=2; DIST[4][1]=2; DIST[4][2]=2; DIST[4][3]=2; DIST[4][4]=0;

DIST[5][0]=3; DIST[5][1]=3; DIST[5][2]=3; DIST[5][3]=3; DIST[5][4]=2; DIST[5][5]=0;

DIST[6][0]=3; DIST[6][1]=3; DIST[6][2]=4; DIST[6][3]=3; DIST[6][4]=3; DIST[6][5]=2; DIST[6][6]=0;

DIST[7][0]=3; DIST[7][1]=2; DIST[7][2]=4; DIST[7][3]=3; DIST[7][4]=3; DIST[7][5]=3; DIST[7][6]=3; DIST[7][7]=0;

DIST[8][0]=1; DIST[8][1]=1; DIST[8][2]=2; DIST[8][3]=1; DIST[8][4]=1; DIST[8][5]=2; DIST[8][6]=2; DIST[8][7]=2; DIST[8][8]=0;

DIST[9][0]=2; DIST[9][1]=2; DIST[9][2]=3; DIST[9][3]=2; DIST[9][4]=2; DIST[9][5]=2; DIST[9][6]=2; DIST[9][7]=3; DIST[9][8]=1; DIST[9][9]=0;

DIST[10][0]=2; DIST[10][1]=3; DIST[10][2]=2; DIST[10][3]=2; DIST[10][4]=2; DIST[10][5]=3; DIST[10][6]=4; DIST[10][7]=4; DIST[10][8]=2; DIST[10][9]=2; DIST[10][10]=0;

DIST[11][0]=2; DIST[11][1]=3; DIST[11][2]=3; DIST[11][3]=2; DIST[11][4]=3; DIST[11][5]=4; DIST[11][6]=3; DIST[11][7]=4; DIST[11][8]=2; DIST[11][9]=3; DIST[11][10]=3; DIST[11][11]=0;

DIST[12][0]=4; DIST[12][1]=3; DIST[12][2]=4; DIST[12][3]=4; DIST[12][4]=3; DIST[12][5]=5; DIST[12][6]=4; DIST[12][7]=4; DIST[12][8]=3; DIST[12][9]=4; DIST[12][10]=4; DIST[12][11]=4; DIST[12][12]=0;

}

{ PROPERTY P5: graph T respects eventual Primary Phylogenetic Hypotheses }

{

This property is verified by constraints programmed in the model generator:

- monophyly of Arthropoda = (0,1,2,3,4,5,6,7,8,9,12)

- monophyly of Mandibulata = (0,1,2,3,4,5,6,7,12)

- monophyly of Crustacea = (1,2,3,4,5,6,7,12)

- monophyly of Decapoda = (4,5,6)

- monophyly of Chelicerata = (8,9)

}

{------------------------------------------------------------------------------------------------------------------------}

{ PROPERTY P6: it is possible to calculate all the values for each HTU in the graph T }

{

First we calculate with the model generator the set of tree solutions which verify properties P1 to P5. Property P6 is verified *a posteriori* for each tree solution, with a *feedback* mechanism:

Studying each tree solution for calculating the values of HTUs, we eventually discover "impossible sub-trees": they appear in tree solutions which verify P1 to P5, but they do not verify P6.

For each impossible subtree A, an additional constraint is programmed into the model generator to forbid the solutions containing A. Tree solutions are recalculated and verified, allowing the discovery of new impossible subtrees and the programming of new constraints to recalculate the solutions (feedback mechanism). Finally, the complete set of optimal solutions is determined after iteration of this process and elimination of all the solutions that do not verify P6.

}

================================================================================

================================================================================

SOLUTIONS

================================================================================

================================================================================

OTUs:

narceus_annularis=0;

speleonectes_tulumensis=1;

argulus_americanus=2;

megabalanus_volcano=3;

eriocheir_sinensis=4;

chinkia_crosnieri=5;

pagurus_longicarpus=6;

vargula_hilgendorfi=7;

limulus_polyphemus=8;

nymphon_gracile=9;

homo_sapiens=10;

katharina_tunicata=11;

ligia_oceanica=12;

AUXILLIARY CONSTANTS used to fix a part of the solution:

G1=13; { used to fix the decapoda group }

G2=14;

G3=15; { used to fix the outgroup form SOL3 }

HTUs:

n1, n2, n3, n4

D = [0,19]: 39 solutions OK (which verify property P6) (15 impossible sub-trees)

minimal score (best) = 147

maximal score = 197

-------------------------------------------------------------------------------------------------------------

G1

[ cox1 cox2 nad2 atp8 atp6 cox3 nad3 -nad5 -nad4 -nad4L nad6 cob -nad1 -rrnL -rrnS ]

G3

[ cox1 cox2 atp8 atp6 cox3 nad3 -nad5 -nad4 -nad4L -cob -nad6 -nad1 -rrnL -rrnS nad2 ]

score = 184:

model 1:

-------------

R(0,8) R(1,8) R(1,n1) R(1,n2) R(2,n4) R(3,8) R(3,n4) R(4,8) R(5,13) R(6,13) R(7,n1) R(8,0) R(8,1) R(8,3) R(8,4) R(8,9) R(8,13) R(8,15) R(9,8) R(10,14) R(11,15) R(12,n3) R(13,5) R(13,6) R(13,8) R(14,10) R(14,15) R(15,8) R(15,11) R(15,14) R(n1,1) R(n1,7) R(n2,1) R(n2,n3) R(n3,12) R(n3,n2) R(n4,2) R(n4,3)

-------------------------------------------------------------------------------------------------------------

G1

[ cox1 cox2 nad2 atp8 atp6 cox3 nad3 -nad5 -nad4 -nad4L nad6 cob -nad1 -rrnL -rrnS ]

G3

[ cox1 cox2 atp8 atp6 cox3 nad3 -nad5 -nad4 -nad4L -cob -nad6 -nad1 -rrnL -rrnS nad2 ]

score = 171:

model 2:

-------------

R(0,8) R(1,8) R(1,n1) R(1,n2) R(2,n4) R(3,8) R(4,8) R(5,13) R(6,13) R(7,n1) R(8,0) R(8,1) R(8,3) R(8,4) R(8,9) R(8,13) R(8,15) R(8,n4) R(9,8) R(10,14) R(11,15) R(12,n3) R(13,5) R(13,6) R(13,8) R(14,10) R(14,15) R(15,8) R(15,11) R(15,14) R(n1,1) R(n1,7) R(n2,1) R(n2,n3) R(n3,12) R(n3,n2) R(n4,2) R(n4,8)

-------------------------------------------------------------------------------------------------------------

G1

[ cox1 cox2 nad2 atp8 atp6 cox3 nad3 -nad5 -nad4 -nad4L nad6 cob -nad1 -rrnL -rrnS ]

G3

[ cox1 cox2 atp8 atp6 cox3 nad3 -nad5 -nad4 -nad4L -cob -nad6 -nad1 -rrnL -rrnS nad2 ]

n4(mod3)

[ cox1 cox2 atp8 atp6 cox3 nad3 -nad6 nad4L nad4 nad5 cob -nad1 -rrnL -rrnS nad2 ]

score = 184:

model 3:

-------------

R(0,8) R(1,8) R(1,n1) R(1,n2) R(2,n4) R(3,n4) R(4,8) R(5,13) R(6,13) R(7,n1) R(8,0) R(8,1) R(8,4) R(8,9) R(8,13) R(8,15) R(8,n4) R(9,8) R(10,14) R(11,15) R(12,n3) R(13,5) R(13,6) R(13,8) R(14,10) R(14,15) R(15,8) R(15,11) R(15,14) R(n1,1) R(n1,7) R(n2,1) R(n2,n3) R(n3,12) R(n3,n2) R(n4,2) R(n4,3) R(n4,8)

-------------------------------------------------------------------------------------------------------------

G1

[ cox1 cox2 nad2 atp8 atp6 cox3 nad3 -nad5 -nad4 -nad4L nad6 cob -nad1 -rrnL -rrnS ]

G3

[ cox1 cox2 atp8 atp6 cox3 nad3 -nad5 -nad4 -nad4L -cob -nad6 -nad1 -rrnL -rrnS nad2 ]

n1(mod4)

[ cox1 cox2 atp8 atp6 cox3 nad3 -nad5 -nad4 -nad4L nad6 cob -rrnL -rrnS nad2 -nad1 ]

[ cox1 cox2 atp8 atp6 cox3 nad1 nad3 -nad5 -nad4 -nad4L nad6 cob -rrnL -rrnS nad2 ]

score = 197:

model 4:

-------------

R(0,8) R(1,n1) R(1,n2) R(2,n4) R(3,8) R(3,n4) R(4,8) R(5,13) R(6,13) R(7,n1) R(8,0) R(8,3) R(8,4) R(8,9) R(8,13) R(8,15) R(8,n1) R(9,8) R(10,14) R(11,15) R(12,n3) R(13,5) R(13,6) R(13,8) R(14,10) R(14,15) R(15,8) R(15,11) R(15,14) R(n1,1) R(n1,7) R(n1,8) R(n2,1) R(n2,n3) R(n3,12) R(n3,n2) R(n4,2) R(n4,3)

-------------------------------------------------------------------------------------------------------------

G1

[ cox1 cox2 nad2 atp8 atp6 cox3 nad3 -nad5 -nad4 -nad4L nad6 cob -nad1 -rrnL -rrnS ]

G3

[ cox1 cox2 atp8 atp6 cox3 nad3 -nad5 -nad4 -nad4L -cob -nad6 -nad1 -rrnL -rrnS nad2 ]

n1(mod5)

[ cox1 cox2 atp8 atp6 cox3 nad3 -nad5 -nad4 -nad4L nad6 cob -rrnL -rrnS nad2 -nad1 ]

[ cox1 cox2 atp8 atp6 cox3 nad1 nad3 -nad5 -nad4 -nad4L nad6 cob -rrnL -rrnS nad2 ]

score = 184:

model 5:

-------------

R(0,8) R(1,n1) R(1,n2) R(2,n4) R(3,8) R(4,8) R(5,13) R(6,13) R(7,n1) R(8,0) R(8,3) R(8,4) R(8,9) R(8,13) R(8,15) R(8,n1) R(8,n4) R(9,8) R(10,14) R(11,15) R(12,n3) R(13,5) R(13,6) R(13,8) R(14,10) R(14,15) R(15,8) R(15,11) R(15,14) R(n1,1) R(n1,7) R(n1,8) R(n2,1) R(n2,n3) R(n3,12) R(n3,n2) R(n4,2) R(n4,8)

-------------------------------------------------------------------------------------------------------------

G1

[ cox1 cox2 nad2 atp8 atp6 cox3 nad3 -nad5 -nad4 -nad4L nad6 cob -nad1 -rrnL -rrnS ]

G3

[ cox1 cox2 atp8 atp6 cox3 nad3 -nad5 -nad4 -nad4L -cob -nad6 -nad1 -rrnL -rrnS nad2 ]

n1(mod6)

[ cox1 cox2 atp8 atp6 cox3 nad3 -nad5 -nad4 -nad4L nad6 cob -rrnL -rrnS nad2 -nad1 ]

[ cox1 cox2 atp8 atp6 cox3 nad1 nad3 -nad5 -nad4 -nad4L nad6 cob -rrnL -rrnS nad2 ]

n4(mod6)

[ cox1 cox2 atp8 atp6 cox3 nad3 -nad6 nad4L nad4 nad5 cob -nad1 -rrnL -rrnS nad2 ]

score = 197:

model 6:

-------------

R(0,8) R(1,n1) R(1,n2) R(2,n4) R(3,n4) R(4,8) R(5,13) R(6,13) R(7,n1) R(8,0) R(8,4) R(8,9) R(8,13) R(8,15) R(8,n1) R(8,n4) R(9,8) R(10,14) R(11,15) R(12,n3) R(13,5) R(13,6) R(13,8) R(14,10) R(14,15) R(15,8) R(15,11) R(15,14) R(n1,1) R(n1,7) R(n1,8) R(n2,1) R(n2,n3) R(n3,12) R(n3,n2) R(n4,2) R(n4,3) R(n4,8)

-------------------------------------------------------------------------------------------------------------

G1

[ cox1 cox2 nad2 atp8 atp6 cox3 nad3 -nad5 -nad4 -nad4L nad6 cob -nad1 -rrnL -rrnS ]

G3

[ cox1 cox2 atp8 atp6 cox3 nad3 -nad5 -nad4 -nad4L -cob -nad6 -nad1 -rrnL -rrnS nad2 ]

score = 173:

model 7:

-------------

R(0,8) R(1,8) R(1,n1) R(2,n2) R(3,8) R(3,n2) R(4,8) R(5,13) R(6,13) R(7,n1) R(8,0) R(8,1) R(8,3) R(8,4) R(8,9) R(8,13) R(8,15) R(8,n3) R(9,8) R(10,14) R(11,15) R(12,n4) R(13,5) R(13,6) R(13,8) R(14,10) R(14,15) R(15,8) R(15,11) R(15,14) R(n1,1) R(n1,7) R(n2,2) R(n2,3) R(n3,8) R(n3,n4) R(n4,12) R(n4,n3)

-------------------------------------------------------------------------------------------------------------

G1

[ cox1 cox2 nad2 atp8 atp6 cox3 nad3 -nad5 -nad4 -nad4L nad6 cob -nad1 -rrnL -rrnS ]

G3

[ cox1 cox2 atp8 atp6 cox3 nad3 -nad5 -nad4 -nad4L -cob -nad6 -nad1 -rrnL -rrnS nad2 ]

n2(mod8)

[ cox1 cox2 atp8 atp6 cox3 nad3 -nad6 nad4L nad4 nad5 cob -nad1 -rrnL -rrnS nad2 ]

score = 197:

model 8:

-------------

R(0,8) R(1,8) R(1,n1) R(2,n2) R(3,8) R(3,n2) R(4,8) R(5,13) R(6,13) R(7,n1) R(8,0) R(8,1) R(8,3) R(8,4) R(8,9) R(8,13) R(8,15) R(9,8) R(10,14) R(11,15) R(12,n4) R(13,5) R(13,6) R(13,8) R(14,10) R(14,15) R(15,8) R(15,11) R(15,14) R(n1,1) R(n1,7) R(n2,2) R(n2,3) R(n2,n3) R(n3,n2) R(n3,n4) R(n4,12) R(n4,n3)

-------------------------------------------------------------------------------------------------------------

G1

[ cox1 cox2 nad2 atp8 atp6 cox3 nad3 -nad5 -nad4 -nad4L nad6 cob -nad1 -rrnL -rrnS ]

G3

[ cox1 cox2 atp8 atp6 cox3 nad3 -nad5 -nad4 -nad4L -cob -nad6 -nad1 -rrnL -rrnS nad2 ]

score = 160:

model 9:

-------------

R(0,8) R(1,8) R(1,n1) R(2,n2) R(3,8) R(4,8) R(5,13) R(6,13) R(7,n1) R(8,0) R(8,1) R(8,3) R(8,4) R(8,9) R(8,13) R(8,15) R(8,n2) R(8,n3) R(9,8) R(10,14) R(11,15) R(12,n4) R(13,5) R(13,6) R(13,8) R(14,10) R(14,15) R(15,8) R(15,11) R(15,14) R(n1,1) R(n1,7) R(n2,2) R(n2,8) R(n3,8) R(n3,n4) R(n4,12) R(n4,n3)

-------------------------------------------------------------------------------------------------------------

G1

[ cox1 cox2 nad2 atp8 atp6 cox3 nad3 -nad5 -nad4 -nad4L nad6 cob -nad1 -rrnL -rrnS ]

G3

[ cox1 cox2 atp8 atp6 cox3 nad3 -nad5 -nad4 -nad4L -cob -nad6 -nad1 -rrnL -rrnS nad2 ]

n2(mod10)

[ cox1 cox2 atp8 atp6 cox3 nad3 -nad6 nad4L nad4 nad5 cob -nad1 -rrnL -rrnS nad2 ]

score = 173:

model 10:

-------------

R(0,8) R(1,8) R(1,n1) R(2,n2) R(3,8) R(4,8) R(5,13) R(6,13) R(7,n1) R(8,0) R(8,1) R(8,3) R(8,4) R(8,9) R(8,13) R(8,15) R(8,n2) R(9,8) R(10,14) R(11,15) R(12,n4) R(13,5) R(13,6) R(13,8) R(14,10) R(14,15) R(15,8) R(15,11) R(15,14) R(n1,1) R(n1,7) R(n2,2) R(n2,8) R(n2,n3) R(n3,n2) R(n3,n4) R(n4,12) R(n4,n3)

-------------------------------------------------------------------------------------------------------------

G1

[ cox1 cox2 nad2 atp8 atp6 cox3 nad3 -nad5 -nad4 -nad4L nad6 cob -nad1 -rrnL -rrnS ]

G3

[ cox1 cox2 atp8 atp6 cox3 nad3 -nad5 -nad4 -nad4L -cob -nad6 -nad1 -rrnL -rrnS nad2 ]

n3(mod11)

[ cox1 cox2 atp8 atp6 cox3 nad3 -nad6 nad4L nad4 -nad5 cob -nad1 -rrnL -rrnS nad2 ]

score = 173:

model 11:

-------------

R(0,8) R(1,8) R(1,n1) R(2,n2) R(3,8) R(4,8) R(5,13) R(6,13) R(7,n1) R(8,0) R(8,1) R(8,3) R(8,4) R(8,9) R(8,13) R(8,15) R(8,n3) R(9,8) R(10,14) R(11,15) R(12,n4) R(13,5) R(13,6) R(13,8) R(14,10) R(14,15) R(15,8) R(15,11) R(15,14) R(n1,1) R(n1,7) R(n2,2) R(n2,n3) R(n3,8) R(n3,n2) R(n3,n4) R(n4,12) R(n4,n3)

-------------------------------------------------------------------------------------------------------------

G1

[ cox1 cox2 nad2 atp8 atp6 cox3 nad3 -nad5 -nad4 -nad4L nad6 cob -nad1 -rrnL -rrnS ]

G3

[ cox1 cox2 atp8 atp6 cox3 nad3 -nad5 -nad4 -nad4L -cob -nad6 -nad1 -rrnL -rrnS nad2 ]

score = 173:

model 12:

-------------

R(0,8) R(1,8) R(1,n1) R(2,n2) R(3,8) R(3,n2) R(4,8) R(5,13) R(6,13) R(7,n4) R(8,0) R(8,1) R(8,3) R(8,4) R(8,9) R(8,13) R(8,15) R(8,n4) R(9,8) R(10,14) R(11,15) R(12,n3) R(13,5) R(13,6) R(13,8) R(14,10) R(14,15) R(15,8) R(15,11) R(15,14) R(n1,1) R(n1,n3) R(n2,2) R(n2,3) R(n3,12) R(n3,n1) R(n4,7) R(n4,8)

-------------------------------------------------------------------------------------------------------------

G1

[ cox1 cox2 nad2 atp8 atp6 cox3 nad3 -nad5 -nad4 -nad4L nad6 cob -nad1 -rrnL -rrnS ]

G3

[ cox1 cox2 atp8 atp6 cox3 nad3 -nad5 -nad4 -nad4L -cob -nad6 -nad1 -rrnL -rrnS nad2 ]

score = 160:

model 13:

-------------

R(0,8) R(1,8) R(1,n1) R(2,n2) R(3,8) R(4,8) R(5,13) R(6,13) R(7,n4) R(8,0) R(8,1) R(8,3) R(8,4) R(8,9) R(8,13) R(8,15) R(8,n2) R(8,n4) R(9,8) R(10,14) R(11,15) R(12,n3) R(13,5) R(13,6) R(13,8) R(14,10) R(14,15) R(15,8) R(15,11) R(15,14) R(n1,1) R(n1,n3) R(n2,2) R(n2,8) R(n3,12) R(n3,n1) R(n4,7) R(n4,8)

-------------------------------------------------------------------------------------------------------------

G1

[ cox1 cox2 nad2 atp8 atp6 cox3 nad3 -nad5 -nad4 -nad4L nad6 cob -nad1 -rrnL -rrnS ]

G3

[ cox1 cox2 atp8 atp6 cox3 nad3 -nad5 -nad4 -nad4L -cob -nad6 -nad1 -rrnL -rrnS nad2 ]

n2(mod14)

[ cox1 cox2 atp8 atp6 cox3 nad3 -nad6 nad4L nad4 nad5 cob -nad1 -rrnL -rrnS nad2 ]

score = 173:

model 14:

-------------

R(0,8) R(1,8) R(1,n1) R(2,n2) R(3,n2) R(4,8) R(5,13) R(6,13) R(7,n1) R(8,0) R(8,1) R(8,4) R(8,9) R(8,13) R(8,15) R(8,n2) R(8,n3) R(9,8) R(10,14) R(11,15) R(12,n4) R(13,5) R(13,6) R(13,8) R(14,10) R(14,15) R(15,8) R(15,11) R(15,14) R(n1,1) R(n1,7) R(n2,2) R(n2,3) R(n2,8) R(n3,8) R(n3,n4) R(n4,12) R(n4,n3)

-------------------------------------------------------------------------------------------------------------

G1

[ cox1 cox2 nad2 atp8 atp6 cox3 nad3 -nad5 -nad4 -nad4L nad6 cob -nad1 -rrnL -rrnS ]

G3

[ cox1 cox2 atp8 atp6 cox3 nad3 -nad5 -nad4 -nad4L -cob -nad6 -nad1 -rrnL -rrnS nad2 ]

n2(mod15)

[ cox1 cox2 atp8 atp6 cox3 nad3 -nad6 nad4L nad4 nad5 cob -nad1 -rrnL -rrnS nad2 ]

score = 184:

model 15:

-------------

R(0,8) R(1,8) R(1,n1) R(2,n2) R(3,n2) R(4,8) R(5,13) R(6,13) R(7,n1) R(8,0) R(8,1) R(8,4) R(8,9) R(8,13) R(8,15) R(8,n2) R(9,8) R(10,14) R(11,15) R(12,n4) R(13,5) R(13,6) R(13,8) R(14,10) R(14,15) R(15,8) R(15,11) R(15,14) R(n1,1) R(n1,7) R(n2,2) R(n2,3) R(n2,8) R(n2,n3) R(n3,n2) R(n3,n4) R(n4,12) R(n4,n3)

-------------------------------------------------------------------------------------------------------------

G1

[ cox1 cox2 nad2 atp8 atp6 cox3 nad3 -nad5 -nad4 -nad4L nad6 cob -nad1 -rrnL -rrnS ]

G3

[ cox1 cox2 atp8 atp6 cox3 nad3 -nad5 -nad4 -nad4L -cob -nad6 -nad1 -rrnL -rrnS nad2 ]

n2(mod16)

[ cox1 cox2 atp8 atp6 cox3 nad3 -nad6 nad4L nad4 nad5 cob -nad1 -rrnL -rrnS nad2 ]

n3(mod16)

[ cox1 cox2 atp8 atp6 cox3 nad3 -nad6 nad4L nad4 -nad5 cob -nad1 -rrnL -rrnS nad2 ]

score = 197:

model 16:

-------------

R(0,8) R(1,8) R(1,n1) R(2,n2) R(3,n2) R(4,8) R(5,13) R(6,13) R(7,n1) R(8,0) R(8,1) R(8,4) R(8,9) R(8,13) R(8,15) R(8,n3) R(9,8) R(10,14) R(11,15) R(12,n4) R(13,5) R(13,6) R(13,8) R(14,10) R(14,15) R(15,8) R(15,11) R(15,14) R(n1,1) R(n1,7) R(n2,2) R(n2,3) R(n2,n3) R(n3,8) R(n3,n2) R(n3,n4) R(n4,12) R(n4,n3)

-------------------------------------------------------------------------------------------------------------

G1

[ cox1 cox2 nad2 atp8 atp6 cox3 nad3 -nad5 -nad4 -nad4L nad6 cob -nad1 -rrnL -rrnS ]

G3

[ cox1 cox2 atp8 atp6 cox3 nad3 -nad5 -nad4 -nad4L -cob -nad6 -nad1 -rrnL -rrnS nad2 ]

n2(mod17)

[ cox1 cox2 atp8 atp6 cox3 nad3 -nad6 nad4L nad4 nad5 cob -nad1 -rrnL -rrnS nad2 ]

score = 173:

model 17:

-------------

R(0,8) R(1,8) R(1,n1) R(2,n2) R(3,n2) R(4,8) R(5,13) R(6,13) R(7,n4) R(8,0) R(8,1) R(8,4) R(8,9) R(8,13) R(8,15) R(8,n2) R(8,n4) R(9,8) R(10,14) R(11,15) R(12,n3) R(13,5) R(13,6) R(13,8) R(14,10) R(14,15) R(15,8) R(15,11) R(15,14) R(n1,1) R(n1,n3) R(n2,2) R(n2,3) R(n2,8) R(n3,12) R(n3,n1) R(n4,7) R(n4,8)

-------------------------------------------------------------------------------------------------------------

G1

[ cox1 cox2 nad2 atp8 atp6 cox3 nad3 -nad5 -nad4 -nad4L nad6 cob -nad1 -rrnL -rrnS ]

G3

[ cox1 cox2 atp8 atp6 cox3 nad3 -nad5 -nad4 -nad4L -cob -nad6 -nad1 -rrnL -rrnS nad2 ]

n1(mod18)

[ cox1 cox2 atp8 atp6 cox3 nad3 -nad5 -nad4 -nad4L nad6 cob -rrnL -rrnS nad2 -nad1 ]

[ cox1 cox2 atp8 atp6 cox3 nad1 nad3 -nad5 -nad4 -nad4L nad6 cob -rrnL -rrnS nad2 ]

score = 173:

model 18:

-------------

R(0,8) R(1,n1) R(2,n2) R(3,8) R(3,n2) R(4,8) R(5,13) R(6,13) R(7,n1) R(8,0) R(8,3) R(8,4) R(8,9) R(8,13) R(8,15) R(8,n1) R(8,n3) R(9,8) R(10,14) R(11,15) R(12,n4) R(13,5) R(13,6) R(13,8) R(14,10) R(14,15) R(15,8) R(15,11) R(15,14) R(n1,1) R(n1,7) R(n1,8) R(n2,2) R(n2,3) R(n3,8) R(n3,n4) R(n4,12) R(n4,n3)

-------------------------------------------------------------------------------------------------------------

G1

[ cox1 cox2 nad2 atp8 atp6 cox3 nad3 -nad5 -nad4 -nad4L nad6 cob -nad1 -rrnL -rrnS ]

G3

[ cox1 cox2 atp8 atp6 cox3 nad3 -nad5 -nad4 -nad4L -cob -nad6 -nad1 -rrnL -rrnS nad2 ]

n1(mod19)

[ cox1 cox2 atp8 atp6 cox3 nad3 -nad5 -nad4 -nad4L nad6 cob -rrnL -rrnS nad2 -nad1 ]

[ cox1 cox2 atp8 atp6 cox3 nad1 nad3 -nad5 -nad4 -nad4L nad6 cob -rrnL -rrnS nad2 ]

n2(mod19)

[ cox1 cox2 atp8 atp6 cox3 nad3 -nad6 nad4L nad4 nad5 cob -nad1 -rrnL -rrnS nad2 ]

score = 197:

model 19:

-------------

R(0,8) R(1,n1) R(2,n2) R(3,8) R(3,n2) R(4,8) R(5,13) R(6,13) R(7,n1) R(8,0) R(8,3) R(8,4) R(8,9) R(8,13) R(8,15) R(8,n1) R(9,8) R(10,14) R(11,15) R(12,n4) R(13,5) R(13,6) R(13,8) R(14,10) R(14,15) R(15,8) R(15,11) R(15,14) R(n1,1) R(n1,7) R(n1,8) R(n2,2) R(n2,3) R(n2,n3) R(n3,n2) R(n3,n4) R(n4,12) R(n4,n3)

-------------------------------------------------------------------------------------------------------------

G1

[ cox1 cox2 nad2 atp8 atp6 cox3 nad3 -nad5 -nad4 -nad4L nad6 cob -nad1 -rrnL -rrnS ]

G3

[ cox1 cox2 atp8 atp6 cox3 nad3 -nad5 -nad4 -nad4L -cob -nad6 -nad1 -rrnL -rrnS nad2 ]

n1(mod20)

[ cox1 cox2 atp8 atp6 cox3 nad3 -nad5 -nad4 -nad4L nad6 cob -rrnL -rrnS nad2 -nad1 ]

[ cox1 cox2 atp8 atp6 cox3 nad1 nad3 -nad5 -nad4 -nad4L nad6 cob -rrnL -rrnS nad2 ]

score = 160:

model 20:

-------------

R(0,8) R(1,n1) R(2,n2) R(3,8) R(4,8) R(5,13) R(6,13) R(7,n1) R(8,0) R(8,3) R(8,4) R(8,9) R(8,13) R(8,15) R(8,n1) R(8,n2) R(8,n3) R(9,8) R(10,14) R(11,15) R(12,n4) R(13,5) R(13,6) R(13,8) R(14,10) R(14,15) R(15,8) R(15,11) R(15,14) R(n1,1) R(n1,7) R(n1,8) R(n2,2) R(n2,8) R(n3,8) R(n3,n4) R(n4,12) R(n4,n3)

-------------------------------------------------------------------------------------------------------------

G1

[ cox1 cox2 nad2 atp8 atp6 cox3 nad3 -nad5 -nad4 -nad4L nad6 cob -nad1 -rrnL -rrnS ]

G3

[ cox1 cox2 atp8 atp6 cox3 nad3 -nad5 -nad4 -nad4L -cob -nad6 -nad1 -rrnL -rrnS nad2 ]

n1(mod21)

[ cox1 cox2 atp8 atp6 cox3 nad3 -nad5 -nad4 -nad4L nad6 cob -rrnL -rrnS nad2 -nad1 ]

[ cox1 cox2 atp8 atp6 cox3 nad1 nad3 -nad5 -nad4 -nad4L nad6 cob -rrnL -rrnS nad2 ]

n2(mod21)

[ cox1 cox2 atp8 atp6 cox3 nad3 -nad6 nad4L nad4 nad5 cob -nad1 -rrnL -rrnS nad2 ]

score = 173:

model 21:

-------------

R(0,8) R(1,n1) R(2,n2) R(3,8) R(4,8) R(5,13) R(6,13) R(7,n1) R(8,0) R(8,3) R(8,4) R(8,9) R(8,13) R(8,15) R(8,n1) R(8,n2) R(9,8) R(10,14) R(11,15) R(12,n4) R(13,5) R(13,6) R(13,8) R(14,10) R(14,15) R(15,8) R(15,11) R(15,14) R(n1,1) R(n1,7) R(n1,8) R(n2,2) R(n2,8) R(n2,n3) R(n3,n2) R(n3,n4) R(n4,12) R(n4,n3)

-------------------------------------------------------------------------------------------------------------

G1

[ cox1 cox2 nad2 atp8 atp6 cox3 nad3 -nad5 -nad4 -nad4L nad6 cob -nad1 -rrnL -rrnS ]

G3

[ cox1 cox2 atp8 atp6 cox3 nad3 -nad5 -nad4 -nad4L -cob -nad6 -nad1 -rrnL -rrnS nad2 ]

n1(mod22)

[ cox1 cox2 atp8 atp6 cox3 nad3 -nad5 -nad4 -nad4L nad6 cob -rrnL -rrnS nad2 -nad1 ]

[ cox1 cox2 atp8 atp6 cox3 nad1 nad3 -nad5 -nad4 -nad4L nad6 cob -rrnL -rrnS nad2 ]

n3(mod22)

[ cox1 cox2 atp8 atp6 cox3 nad3 -nad6 nad4L nad4 -nad5 cob -nad1 -rrnL -rrnS nad2 ]

score = 173:

model 22:

-------------

R(0,8) R(1,n1) R(2,n2) R(3,8) R(4,8) R(5,13) R(6,13) R(7,n1) R(8,0) R(8,3) R(8,4) R(8,9) R(8,13) R(8,15) R(8,n1) R(8,n3) R(9,8) R(10,14) R(11,15) R(12,n4) R(13,5) R(13,6) R(13,8) R(14,10) R(14,15) R(15,8) R(15,11) R(15,14) R(n1,1) R(n1,7) R(n1,8) R(n2,2) R(n2,n3) R(n3,8) R(n3,n2) R(n3,n4) R(n4,12) R(n4,n3)

-------------------------------------------------------------------------------------------------------------

G1

[ cox1 cox2 nad2 atp8 atp6 cox3 nad3 -nad5 -nad4 -nad4L nad6 cob -nad1 -rrnL -rrnS ]

G3

[ cox1 cox2 atp8 atp6 cox3 nad3 -nad5 -nad4 -nad4L -cob -nad6 -nad1 -rrnL -rrnS nad2 ]

n1(mod23)

[ cox1 cox2 atp8 atp6 cox3 nad3 -nad5 -nad4 -nad4L nad6 cob -rrnL -rrnS nad2 -nad1 ]

[ cox1 cox2 atp8 atp6 cox3 nad1 nad3 -nad5 -nad4 -nad4L nad6 cob -rrnL -rrnS nad2 ]

n2(mod23)

[ cox1 cox2 atp8 atp6 cox3 nad3 -nad6 nad4L nad4 nad5 cob -nad1 -rrnL -rrnS nad2 ]

score = 173:

model 23:

-------------

R(0,8) R(1,n1) R(2,n2) R(3,n2) R(4,8) R(5,13) R(6,13) R(7,n1) R(8,0) R(8,4) R(8,9) R(8,13) R(8,15) R(8,n1) R(8,n2) R(8,n3) R(9,8) R(10,14) R(11,15) R(12,n4) R(13,5) R(13,6) R(13,8) R(14,10) R(14,15) R(15,8) R(15,11) R(15,14) R(n1,1) R(n1,7) R(n1,8) R(n2,2) R(n2,3) R(n2,8) R(n3,8) R(n3,n4) R(n4,12) R(n4,n3)

-------------------------------------------------------------------------------------------------------------

G1

[ cox1 cox2 nad2 atp8 atp6 cox3 nad3 -nad5 -nad4 -nad4L nad6 cob -nad1 -rrnL -rrnS ]

G3

[ cox1 cox2 atp8 atp6 cox3 nad3 -nad5 -nad4 -nad4L -cob -nad6 -nad1 -rrnL -rrnS nad2 ]

n1(mod24)

[ cox1 cox2 atp8 atp6 cox3 nad3 -nad5 -nad4 -nad4L nad6 cob -rrnL -rrnS nad2 -nad1 ]

[ cox1 cox2 atp8 atp6 cox3 nad1 nad3 -nad5 -nad4 -nad4L nad6 cob -rrnL -rrnS nad2 ]

n2(mod24)

[ cox1 cox2 atp8 atp6 cox3 nad3 -nad6 nad4L nad4 nad5 cob -nad1 -rrnL -rrnS nad2 ]

score = 184:

model 24:

-------------

R(0,8) R(1,n1) R(2,n2) R(3,n2) R(4,8) R(5,13) R(6,13) R(7,n1) R(8,0) R(8,4) R(8,9) R(8,13) R(8,15) R(8,n1) R(8,n2) R(9,8) R(10,14) R(11,15) R(12,n4) R(13,5) R(13,6) R(13,8) R(14,10) R(14,15) R(15,8) R(15,11) R(15,14) R(n1,1) R(n1,7) R(n1,8) R(n2,2) R(n2,3) R(n2,8) R(n2,n3) R(n3,n2) R(n3,n4) R(n4,12) R(n4,n3)

-------------------------------------------------------------------------------------------------------------

G1

[ cox1 cox2 nad2 atp8 atp6 cox3 nad3 -nad5 -nad4 -nad4L nad6 cob -nad1 -rrnL -rrnS ]

G3

[ cox1 cox2 atp8 atp6 cox3 nad3 -nad5 -nad4 -nad4L -cob -nad6 -nad1 -rrnL -rrnS nad2 ]

n1(mod25)

[ cox1 cox2 atp8 atp6 cox3 nad3 -nad5 -nad4 -nad4L nad6 cob -rrnL -rrnS nad2 -nad1 ]

[ cox1 cox2 atp8 atp6 cox3 nad1 nad3 -nad5 -nad4 -nad4L nad6 cob -rrnL -rrnS nad2 ]

n2(mod25)

[ cox1 cox2 atp8 atp6 cox3 nad3 -nad6 nad4L nad4 nad5 cob -nad1 -rrnL -rrnS nad2 ]

n3(mod25)

[ cox1 cox2 atp8 atp6 cox3 nad3 -nad6 nad4L nad4 -nad5 cob -nad1 -rrnL -rrnS nad2 ]

score = 197:

model 25:

-------------

R(0,8) R(1,n1) R(2,n2) R(3,n2) R(4,8) R(5,13) R(6,13) R(7,n1) R(8,0) R(8,4) R(8,9) R(8,13) R(8,15) R(8,n1) R(8,n3) R(9,8) R(10,14) R(11,15) R(12,n4) R(13,5) R(13,6) R(13,8) R(14,10) R(14,15) R(15,8) R(15,11) R(15,14) R(n1,1) R(n1,7) R(n1,8) R(n2,2) R(n2,3) R(n2,n3) R(n3,8) R(n3,n2) R(n3,n4) R(n4,12) R(n4,n3)

-------------------------------------------------------------------------------------------------------------

G1

[ cox1 cox2 nad2 atp8 atp6 cox3 nad3 -nad5 -nad4 -nad4L nad6 cob -nad1 -rrnL -rrnS ]

G3

[ cox1 cox2 atp8 atp6 cox3 nad3 -nad5 -nad4 -nad4L -cob -nad6 -nad1 -rrnL -rrnS nad2 ]

n1(mod26)

[ cox1 cox2 atp8 atp6 cox3 nad3 -nad6 nad4L nad4 nad5 cob -nad1 -rrnL -rrnS nad2 ]

score = 184:

model 26:

-------------

R(0,8) R(1,8) R(2,n1) R(3,8) R(3,n1) R(4,8) R(5,13) R(6,13) R(7,n2) R(8,0) R(8,1) R(8,3) R(8,4) R(8,9) R(8,13) R(8,15) R(8,n2) R(9,8) R(10,14) R(11,15) R(12,n4) R(13,5) R(13,6) R(13,8) R(14,10) R(14,15) R(15,8) R(15,11) R(15,14) R(n1,2) R(n1,3) R(n1,n3) R(n2,7) R(n2,8) R(n3,n1) R(n3,n4) R(n4,12) R(n4,n3)

-------------------------------------------------------------------------------------------------------------

G1

[ cox1 cox2 nad2 atp8 atp6 cox3 nad3 -nad5 -nad4 -nad4L nad6 cob -nad1 -rrnL -rrnS ]

G3

[ cox1 cox2 atp8 atp6 cox3 nad3 -nad5 -nad4 -nad4L -cob -nad6 -nad1 -rrnL -rrnS nad2 ]

score = 160:

model 27:

-------------

R(0,8) R(1,8) R(2,n1) R(3,8) R(3,n1) R(4,8) R(5,13) R(6,13) R(7,n2) R(8,0) R(8,1) R(8,3) R(8,4) R(8,9) R(8,13) R(8,15) R(8,n2) R(8,n3) R(9,8) R(10,14) R(11,15) R(12,n4) R(13,5) R(13,6) R(13,8) R(14,10) R(14,15) R(15,8) R(15,11) R(15,14) R(n1,2) R(n1,3) R(n2,7) R(n2,8) R(n3,8) R(n3,n4) R(n4,12) R(n4,n3)

-------------------------------------------------------------------------------------------------------------

G1

[ cox1 cox2 nad2 atp8 atp6 cox3 nad3 -nad5 -nad4 -nad4L nad6 cob -nad1 -rrnL -rrnS ]

G3

[ cox1 cox2 atp8 atp6 cox3 nad3 -nad5 -nad4 -nad4L -cob -nad6 -nad1 -rrnL -rrnS nad2 ]

n2(mod28)

[ cox1 cox2 atp8 atp6 cox3 nad3 -nad2 rrnS rrnL -nad5 -nad4 -nad4L nad6 cob -nad1 ]

score = 173:

model 28:

-------------

R(0,8) R(1,8) R(2,n1) R(3,8) R(3,n1) R(4,8) R(5,13) R(6,13) R(7,n2) R(8,0) R(8,1) R(8,3) R(8,4) R(8,9) R(8,13) R(8,15) R(8,n2) R(9,8) R(10,14) R(11,15) R(12,n4) R(13,5) R(13,6) R(13,8) R(14,10) R(14,15) R(15,8) R(15,11) R(15,14) R(n1,2) R(n1,3) R(n2,7) R(n2,8) R(n2,n3) R(n3,n2) R(n3,n4) R(n4,12) R(n4,n3)

-------------------------------------------------------------------------------------------------------------

G1

[ cox1 cox2 nad2 atp8 atp6 cox3 nad3 -nad5 -nad4 -nad4L nad6 cob -nad1 -rrnL -rrnS ]

G3

[ cox1 cox2 atp8 atp6 cox3 nad3 -nad5 -nad4 -nad4L -cob -nad6 -nad1 -rrnL -rrnS nad2 ]

n3(mod29)

[ cox1 cox2 atp8 atp6 cox3 nad3 -nad1 -cob -nad6 nad4L nad4 nad5 -rrnL -rrnS nad2 ]

score = 173:

model 29:

-------------

R(0,8) R(1,8) R(2,n1) R(3,8) R(3,n1) R(4,8) R(5,13) R(6,13) R(7,n2) R(8,0) R(8,1) R(8,3) R(8,4) R(8,9) R(8,13) R(8,15) R(8,n3) R(9,8) R(10,14) R(11,15) R(12,n4) R(13,5) R(13,6) R(13,8) R(14,10) R(14,15) R(15,8) R(15,11) R(15,14) R(n1,2) R(n1,3) R(n2,7) R(n2,n3) R(n3,8) R(n3,n2) R(n3,n4) R(n4,12) R(n4,n3)

-------------------------------------------------------------------------------------------------------------

G1

[ cox1 cox2 nad2 atp8 atp6 cox3 nad3 -nad5 -nad4 -nad4L nad6 cob -nad1 -rrnL -rrnS ]

G3

[ cox1 cox2 atp8 atp6 cox3 nad3 -nad5 -nad4 -nad4L -cob -nad6 -nad1 -rrnL -rrnS nad2 ]

n1(mod30)

[ cox1 cox2 atp8 atp6 cox3 nad3 -nad6 nad4L nad4 nad5 cob -nad1 -rrnL -rrnS nad2 ]

score = 160:

model 30:

-------------

R(0,8) R(1,8) R(2,n1) R(3,8) R(4,8) R(5,13) R(6,13) R(7,n2) R(8,0) R(8,1) R(8,3) R(8,4) R(8,9) R(8,13) R(8,15) R(8,n1) R(8,n2) R(9,8) R(10,14) R(11,15) R(12,n4) R(13,5) R(13,6) R(13,8) R(14,10) R(14,15) R(15,8) R(15,11) R(15,14) R(n1,2) R(n1,8) R(n1,n3) R(n2,7) R(n2,8) R(n3,n1) R(n3,n4) R(n4,12) R(n4,n3)

-------------------------------------------------------------------------------------------------------------

-> the best model

G1

[ cox1 cox2 nad2 atp8 atp6 cox3 nad3 -nad5 -nad4 -nad4L nad6 cob -nad1 -rrnL -rrnS ]

G3

[ cox1 cox2 atp8 atp6 cox3 nad3 -nad5 -nad4 -nad4L -cob -nad6 -nad1 -rrnL -rrnS nad2 ]

score = 147:

model 31:

-------------

R(0,8) R(1,8) R(2,n1) R(3,8) R(4,8) R(5,13) R(6,13) R(7,n2) R(8,0) R(8,1) R(8,3) R(8,4) R(8,9) R(8,13) R(8,15) R(8,n1) R(8,n2) R(8,n3) R(9,8) R(10,14) R(11,15) R(12,n4) R(13,5) R(13,6) R(13,8) R(14,10) R(14,15) R(15,8) R(15,11) R(15,14) R(n1,2) R(n1,8) R(n2,7) R(n2,8) R(n3,8) R(n3,n4) R(n4,12) R(n4,n3)

-------------------------------------------------------------------------------------------------------------

G1

[ cox1 cox2 nad2 atp8 atp6 cox3 nad3 -nad5 -nad4 -nad4L nad6 cob -nad1 -rrnL -rrnS ]

G3

[ cox1 cox2 atp8 atp6 cox3 nad3 -nad5 -nad4 -nad4L -cob -nad6 -nad1 -rrnL -rrnS nad2 ]

n2(mod32)

[ cox1 cox2 atp8 atp6 cox3 nad3 -nad2 rrnS rrnL -nad5 -nad4 -nad4L nad6 cob -nad1 ]

score = 160:

model 32:

-------------

R(0,8) R(1,8) R(2,n1) R(3,8) R(4,8) R(5,13) R(6,13) R(7,n2) R(8,0) R(8,1) R(8,3) R(8,4) R(8,9) R(8,13) R(8,15) R(8,n1) R(8,n2) R(9,8) R(10,14) R(11,15) R(12,n4) R(13,5) R(13,6) R(13,8) R(14,10) R(14,15) R(15,8) R(15,11) R(15,14) R(n1,2) R(n1,8) R(n2,7) R(n2,8) R(n2,n3) R(n3,n2) R(n3,n4) R(n4,12) R(n4,n3)

-------------------------------------------------------------------------------------------------------------

G1

[ cox1 cox2 nad2 atp8 atp6 cox3 nad3 -nad5 -nad4 -nad4L nad6 cob -nad1 -rrnL -rrnS ]

G3

[ cox1 cox2 atp8 atp6 cox3 nad3 -nad5 -nad4 -nad4L -cob -nad6 -nad1 -rrnL -rrnS nad2 ]

n3(mod33)

[ cox1 cox2 atp8 atp6 cox3 nad3 -nad1 -cob -nad6 nad4L nad4 nad5 -rrnL -rrnS nad2 ]

score = 160:

model 33:

-------------

R(0,8) R(1,8) R(2,n1) R(3,8) R(4,8) R(5,13) R(6,13) R(7,n2) R(8,0) R(8,1) R(8,3) R(8,4) R(8,9) R(8,13) R(8,15) R(8,n1) R(8,n3) R(9,8) R(10,14) R(11,15) R(12,n4) R(13,5) R(13,6) R(13,8) R(14,10) R(14,15) R(15,8) R(15,11) R(15,14) R(n1,2) R(n1,8) R(n2,7) R(n2,n3) R(n3,8) R(n3,n2) R(n3,n4) R(n4,12) R(n4,n3)

-------------------------------------------------------------------------------------------------------------

G1

[ cox1 cox2 nad2 atp8 atp6 cox3 nad3 -nad5 -nad4 -nad4L nad6 cob -nad1 -rrnL -rrnS ]

G3

[ cox1 cox2 atp8 atp6 cox3 nad3 -nad5 -nad4 -nad4L -cob -nad6 -nad1 -rrnL -rrnS nad2 ]

n3(mod34)

[ cox1 cox2 atp8 atp6 cox3 nad3 -nad6 nad4L nad4 -nad5 cob -nad1 -rrnL -rrnS nad2 ]

score = 160:

model 34:

-------------

R(0,8) R(1,8) R(2,n1) R(3,8) R(4,8) R(5,13) R(6,13) R(7,n2) R(8,0) R(8,1) R(8,3) R(8,4) R(8,9) R(8,13) R(8,15) R(8,n2) R(8,n3) R(9,8) R(10,14) R(11,15) R(12,n4) R(13,5) R(13,6) R(13,8) R(14,10) R(14,15) R(15,8) R(15,11) R(15,14) R(n1,2) R(n1,n3) R(n2,7) R(n2,8) R(n3,8) R(n3,n1) R(n3,n4) R(n4,12) R(n4,n3)

-------------------------------------------------------------------------------------------------------------

G1

[ cox1 cox2 nad2 atp8 atp6 cox3 nad3 -nad5 -nad4 -nad4L nad6 cob -nad1 -rrnL -rrnS ]

G3

[ cox1 cox2 atp8 atp6 cox3 nad3 -nad5 -nad4 -nad4L -cob -nad6 -nad1 -rrnL -rrnS nad2 ]

n1(mod35)

[ cox1 cox2 atp8 atp6 cox3 nad3 -nad6 nad4L nad4 nad5 cob -nad1 -rrnL -rrnS nad2 ]

score = 171:

model 35:

-------------

R(0,8) R(1,8) R(2,n1) R(3,n1) R(4,8) R(5,13) R(6,13) R(7,n2) R(8,0) R(8,1) R(8,4) R(8,9) R(8,13) R(8,15) R(8,n1) R(8,n2) R(9,8) R(10,14) R(11,15) R(12,n4) R(13,5) R(13,6) R(13,8) R(14,10) R(14,15) R(15,8) R(15,11) R(15,14) R(n1,2) R(n1,3) R(n1,8) R(n1,n3) R(n2,7) R(n2,8) R(n3,n1) R(n3,n4) R(n4,12) R(n4,n3)

-------------------------------------------------------------------------------------------------------------

G1

[ cox1 cox2 nad2 atp8 atp6 cox3 nad3 -nad5 -nad4 -nad4L nad6 cob -nad1 -rrnL -rrnS ]

G3

[ cox1 cox2 atp8 atp6 cox3 nad3 -nad5 -nad4 -nad4L -cob -nad6 -nad1 -rrnL -rrnS nad2 ]

n1(mod36)

[ cox1 cox2 atp8 atp6 cox3 nad3 -nad6 nad4L nad4 nad5 cob -nad1 -rrnL -rrnS nad2 ]

score = 160:

model 36:

-------------

R(0,8) R(1,8) R(2,n1) R(3,n1) R(4,8) R(5,13) R(6,13) R(7,n2) R(8,0) R(8,1) R(8,4) R(8,9) R(8,13) R(8,15) R(8,n1) R(8,n2) R(8,n3) R(9,8) R(10,14) R(11,15) R(12,n4) R(13,5) R(13,6) R(13,8) R(14,10) R(14,15) R(15,8) R(15,11) R(15,14) R(n1,2) R(n1,3) R(n1,8) R(n2,7) R(n2,8) R(n3,8) R(n3,n4) R(n4,12) R(n4,n3)

-------------------------------------------------------------------------------------------------------------

G1

[ cox1 cox2 nad2 atp8 atp6 cox3 nad3 -nad5 -nad4 -nad4L nad6 cob -nad1 -rrnL -rrnS ]

G3

[ cox1 cox2 atp8 atp6 cox3 nad3 -nad5 -nad4 -nad4L -cob -nad6 -nad1 -rrnL -rrnS nad2 ]

n1(mod37)

[ cox1 cox2 atp8 atp6 cox3 nad3 -nad6 nad4L nad4 nad5 cob -nad1 -rrnL -rrnS nad2 ]

n2(mod37)

[ cox1 cox2 atp8 atp6 cox3 nad3 -nad2 rrnS rrnL -nad5 -nad4 -nad4L nad6 cob -nad1 ]

score = 173:

model 37:

-------------

R(0,8) R(1,8) R(2,n1) R(3,n1) R(4,8) R(5,13) R(6,13) R(7,n2) R(8,0) R(8,1) R(8,4) R(8,9) R(8,13) R(8,15) R(8,n1) R(8,n2) R(9,8) R(10,14) R(11,15) R(12,n4) R(13,5) R(13,6) R(13,8) R(14,10) R(14,15) R(15,8) R(15,11) R(15,14) R(n1,2) R(n1,3) R(n1,8) R(n2,7) R(n2,8) R(n2,n3) R(n3,n2) R(n3,n4) R(n4,12) R(n4,n3)

-------------------------------------------------------------------------------------------------------------

G1

[ cox1 cox2 nad2 atp8 atp6 cox3 nad3 -nad5 -nad4 -nad4L nad6 cob -nad1 -rrnL -rrnS ]

G3

[ cox1 cox2 atp8 atp6 cox3 nad3 -nad5 -nad4 -nad4L -cob -nad6 -nad1 -rrnL -rrnS nad2 ]

n1(mod38)

[ cox1 cox2 atp8 atp6 cox3 nad3 -nad6 nad4L nad4 nad5 cob -nad1 -rrnL -rrnS nad2 ]

n3(mod38)

[ cox1 cox2 atp8 atp6 cox3 nad3 -nad1 -cob -nad6 nad4L nad4 nad5 -rrnL -rrnS nad2 ]

score = 173:

model 38:

-------------

R(0,8) R(1,8) R(2,n1) R(3,n1) R(4,8) R(5,13) R(6,13) R(7,n2) R(8,0) R(8,1) R(8,4) R(8,9) R(8,13) R(8,15) R(8,n1) R(8,n3) R(9,8) R(10,14) R(11,15) R(12,n4) R(13,5) R(13,6) R(13,8) R(14,10) R(14,15) R(15,8) R(15,11) R(15,14) R(n1,2) R(n1,3) R(n1,8) R(n2,7) R(n2,n3) R(n3,8) R(n3,n2) R(n3,n4) R(n4,12) R(n4,n3)

-------------------------------------------------------------------------------------------------------------

G1

[ cox1 cox2 nad2 atp8 atp6 cox3 nad3 -nad5 -nad4 -nad4L nad6 cob -nad1 -rrnL -rrnS ]

G3

[ cox1 cox2 atp8 atp6 cox3 nad3 -nad5 -nad4 -nad4L -cob -nad6 -nad1 -rrnL -rrnS nad2 ]

n1(mod39)

[ cox1 cox2 atp8 atp6 cox3 nad3 -nad6 nad4L nad4 nad5 cob -nad1 -rrnL -rrnS nad2 ]

n3(mod39)

[ cox1 cox2 atp8 atp6 cox3 nad3 -nad6 nad4L nad4 -nad5 cob -nad1 -rrnL -rrnS nad2 ]

score = 184:

model 39:

-------------

R(0,8) R(1,8) R(2,n1) R(3,n1) R(4,8) R(5,13) R(6,13) R(7,n2) R(8,0) R(8,1) R(8,4) R(8,9) R(8,13) R(8,15) R(8,n2) R(8,n3) R(9,8) R(10,14) R(11,15) R(12,n4) R(13,5) R(13,6) R(13,8) R(14,10) R(14,15) R(15,8) R(15,11) R(15,14) R(n1,2) R(n1,3) R(n1,n3) R(n2,7) R(n2,8) R(n3,8) R(n3,n1) R(n3,n4) R(n4,12) R(n4,n3)

-------------------------------------------------------------------------------------------------------------

-> no other models

ecdysozoans_taxD_9sol

================================================================================

================================================================================

AXIOMS

================================================================================

================================================================================

{ the solutions of problem PHYLO are the smallest graphs T (defined on the smallest domain possible but containing at least all the OTUs) which verify properties P1 to P6:

P1- T is simple (the relation R(x, y) which defines graph T is not reflexive)

P2- T is non-oriented (the relation R(x, y) which defines graph T is symetrical)

P3- T is connected and acyclic (T is a tree)

P4- T respects the minimal distance matrix, i.e.:

for all couple of OTUs x and y, the length of the path x->y in T is always superior or equals to the minimal distance calculated between x and y (encoded in the minimal distance matrix)

P5- T respects other eventual hypothesis (Primary Phylogenetic Hypothesis = PPH)

used to impose the existence of given monophyletic groups

P6- it is possible to calculate all the values for each HTU in the graph T }

{ OTUs: }

narceus_annularis=0;

speleonectes_tulumensis=1;

argulus_americanus=2;

megabalanus_volcano=3;

eriocheir_sinensis=4;

chinkia_crosnieri=5;

pagurus_longicarpus=6;

vargula_hilgendorfi=7;

limulus_polyphemus=8;

nymphon_gracile=9; { = outgroup1 }

homo_sapiens=10; { = outgroup2 }

katharina_tunicata=11; { = outgroup3 }

ligia_oceanica=12;

cherax_destructor=13;

{ AUXILLIARY CONSTANTS used to fix a part of the solution: }

G1=14;

G2=15;

G3=16;

G4=17; { used to fix the crustacea group (except decapods) }

{ THE CRUSTACEA GROUP IS FIXED (except decapods): }

R(limulus_polyphemus,speleonectes_tulumensis);

R(limulus_polyphemus,megabalanus_volcano);

R(limulus_polyphemus,G1) et R(G1,argulus_americanus);

R(limulus_polyphemus,G2) et R(G2,vargula_hilgendorfi);

R(limulus_polyphemus,G3) et R(G3,G4) et R(G4,ligia_oceanica);

{ PROPERTY P1: R(x, y) is not reflexive}

Q x (-R(x, x));

{ PROPERTY P2: R(x, y) is symetrical}

Q x y (R(x, y) => R(y, x));

{ PROPERTY P3: graph T is connected and acyclic (T is a tree) }

{

This property is verified by a constraint programmed in the model generator, instead of a "heavy" logical formula:

1- it will refuse the partial interpretations in which a connected component of the graph (in construction) is cyclic, i.e. such as: number of edges >= number of vertices

2- it will refuse the complete interpretations in which the constructed graph has more than one connected component

}

{ PROPERTY P4: graph T respects minimal distance matrix }

{

This property is verified by a constraint programmed in the model generator:

it will refuse the partial interpretations in which the graph (in construction) do not respect the minimal distance matrix, i.e. such as:

Let x, y a couple of OTUs,

let d = minimal distance calculated between x and y (encoded in the minimal distance matrix), there is a path of length k between x and y, with: k < d

The minimal distance matrix is encoded directly in the data structure of the model generator:

/* minimal distance matrix ECDYSOZOANS taxD: */

DIST[0][0]=0;

DIST[1][0]=2; DIST[1][1]=0;

DIST[2][0]=2; DIST[2][1]=3; DIST[2][2]=0;

DIST[3][0]=1; DIST[3][1]=2; DIST[3][2]=2; DIST[3][3]=0;

DIST[4][0]=2; DIST[4][1]=2; DIST[4][2]=2; DIST[4][3]=2; DIST[4][4]=0;

DIST[5][0]=3; DIST[5][1]=3; DIST[5][2]=3; DIST[5][3]=3; DIST[5][4]=2; DIST[5][5]=0;

DIST[6][0]=3; DIST[6][1]=3; DIST[6][2]=4; DIST[6][3]=3; DIST[6][4]=3; DIST[6][5]=2; DIST[6][6]=0;

DIST[7][0]=3; DIST[7][1]=2; DIST[7][2]=4; DIST[7][3]=3; DIST[7][4]=3; DIST[7][5]=3; DIST[7][6]=3; DIST[7][7]=0;

DIST[8][0]=1; DIST[8][1]=1; DIST[8][2]=2; DIST[8][3]=1; DIST[8][4]=1; DIST[8][5]=2; DIST[8][6]=2; DIST[8][7]=2; DIST[8][8]=0;

DIST[9][0]=2; DIST[9][1]=2; DIST[9][2]=3; DIST[9][3]=2; DIST[9][4]=2; DIST[9][5]=2; DIST[9][6]=2; DIST[9][7]=3; DIST[9][8]=1; DIST[9][9]=0;

DIST[10][0]=2; DIST[10][1]=3; DIST[10][2]=2; DIST[10][3]=2; DIST[10][4]=2; DIST[10][5]=3; DIST[10][6]=4; DIST[10][7]=4; DIST[10][8]=2; DIST[10][9]=2; DIST[10][10]=0;

DIST[11][0]=2; DIST[11][1]=3; DIST[11][2]=3; DIST[11][3]=2; DIST[11][4]=3; DIST[11][5]=4; DIST[11][6]=3; DIST[11][7]=4; DIST[11][8]=2; DIST[11][9]=3; DIST[11][10]=3; DIST[11][11]=0;

DIST[12][0]=4; DIST[12][1]=3; DIST[12][2]=4; DIST[12][3]=4; DIST[12][4]=3; DIST[12][5]=5; DIST[12][6]=4; DIST[12][7]=4; DIST[12][8]=3; DIST[12][9]=4; DIST[12][10]=4; DIST[12][11]=4; DIST[12][12]=0;

DIST[13][0]=4; DIST[13][1]=3; DIST[13][2]=4; DIST[13][3]=4; DIST[13][4]=4; DIST[13][5]=4; DIST[13][6]=4; DIST[13][7]=4; DIST[13][8]=3; DIST[13][9]=4; DIST[13][10]=4; DIST[13][11]=5; DIST[13][12]=3; DIST[13][13]=0;

}

{ PROPERTY P5: graph T respects eventual Primary Phylogenetic Hypotheses }

{

This property is verified by constraints programmed in the model generator:

- monophyly of Arthropoda = (0,1,2,3,4,5,6,7,8,9,12,13)

- monophyly of Mandibulata = (0,1,2,3,4,5,6,7,12,13)

- monophyly of Crustacea = (1,2,3,4,5,6,7,12,13)

- monophyly of Decapoda = (4,5,6,13)

- monophyly of Chelicerata = (8,9)

}

{------------------------------------------------------------------------------------------------------------------------}

{ PROPERTY P6: it is possible to calculate all the values for each HTU in the graph T }

{

First we calculate with the model generator the set of tree solutions which verify properties P1 to P5. Property P6 is verified *a posteriori* for each tree solution, with a *feedback* mechanism:

Studying each tree solution for calculating the values of HTUs, we eventually discover "impossible sub-trees": they appear in tree solutions which verify P1 to P5, but they do not verify P6.

For each impossible subtree A, an additional constraint is programmed into the model generator to forbid the solutions containing A. Tree solutions are recalculated and verified, allowing the discovery of new impossible subtrees and the programming of new constraints to recalculate the solutions (feedback mechanism). Finally, the complete set of optimal solutions is determined after iteration of this process and elimination of all the solutions that do not verify P6.

}

================================================================================

================================================================================

SOLUTIONS

================================================================================

================================================================================

OTUs:

narceus_annularis=0;

speleonectes_tulumensis=1;

argulus_americanus=2;

megabalanus_volcano=3;

eriocheir_sinensis=4;

chinkia_crosnieri=5;

pagurus_longicarpus=6;

vargula_hilgendorfi=7;

limulus_polyphemus=8;

nymphon_gracile=9;

homo_sapiens=10;

katharina_tunicata=11;

ligia_oceanica=12;

cherax_destructor=13;

AUXILLIARY CONSTANTS used to fix a part of the solution:

G1=14;

G2=15;

G3=16;

G4=17; { used to fix the crustacea group except decapods }

HTUs:

n1, n2, n3, n4, n5

D = [0,22]: 9 solutions OK (which verify property P6) (36 impossible sub-trees)

minimal score (best) = 204

maximal score = 247

-------------------------------------------------------------------------------------------------------------

-> form outgroup SOL1

n1(mod1)

[ cox1 cox2 nad2 atp8 atp6 cox3 nad3 -nad5 -nad4 -nad4L nad6 cob -nad1 -rrnL -rrnS ]

score = 232:

model 1:

-------------

R(0,8) R(1,8) R(2,14) R(3,8) R(4,8) R(5,n1) R(6,n1) R(7,15) R(8,0) R(8,1) R(8,3) R(8,4) R(8,9) R(8,14) R(8,15) R(8,16) R(8,n1) R(8,n4) R(9,8) R(10,n4) R(11,n5) R(12,17) R(13,n3) R(14,2) R(14,8) R(15,7) R(15,8) R(16,8) R(16,17) R(17,12) R(17,16) R(n1,5) R(n1,6) R(n1,8) R(n1,n2) R(n2,n1) R(n2,n3) R(n3,13) R(n3,n2) R(n4,8) R(n4,10) R(n4,n5) R(n5,11) R(n5,n4)

-------------------------------------------------------------------------------------------------------------

-> form outgroup SOL3

n1(mod2)

[ cox1 cox2 nad2 atp8 atp6 cox3 nad3 -nad5 -nad4 -nad4L nad6 cob -nad1 -rrnL -rrnS ]

score = 232:

model 2:

-------------

R(0,8) R(1,8) R(2,14) R(3,8) R(4,8) R(5,n1) R(6,n1) R(7,15) R(8,0) R(8,1) R(8,3) R(8,4) R(8,9) R(8,14) R(8,15) R(8,16) R(8,n1) R(8,n5) R(9,8) R(10,n4) R(11,n5) R(12,17) R(13,n3) R(14,2) R(14,8) R(15,7) R(15,8) R(16,8) R(16,17) R(17,12) R(17,16) R(n1,5) R(n1,6) R(n1,8) R(n1,n2) R(n2,n1) R(n2,n3) R(n3,13) R(n3,n2) R(n4,10) R(n4,n5) R(n5,8) R(n5,11) R(n5,n4)

-------------------------------------------------------------------------------------------------------------

-> form outgroup SOL2

n1(mod3)

[ cox1 cox2 nad2 atp8 atp6 cox3 nad3 -nad5 -nad4 -nad4L nad6 cob -nad1 -rrnL -rrnS ]

score = 217:

model 3:

-------------

R(0,8) R(1,8) R(2,14) R(3,8) R(4,8) R(5,n1) R(6,n1) R(7,15) R(8,0) R(8,1) R(8,3) R(8,4) R(8,9) R(8,14) R(8,15) R(8,16) R(8,n1) R(8,n4) R(8,n5) R(9,8) R(10,n4) R(11,n5) R(12,17) R(13,n3) R(14,2) R(14,8) R(15,7) R(15,8) R(16,8) R(16,17) R(17,12) R(17,16) R(n1,5) R(n1,6) R(n1,8) R(n1,n2) R(n2,n1) R(n2,n3) R(n3,13) R(n3,n2) R(n4,8) R(n4,10) R(n5,8) R(n5,11)

-------------------------------------------------------------------------------------------------------------

-> form outgroup SOL1

n1(mod4)

[ cox1 cox2 nad2 atp8 atp6 cox3 nad3 -nad5 -nad4 -nad4L nad6 cob -nad1 -rrnL -rrnS ]

n2(mod4)

g1: [ cox1 cox2 atp8 atp6 cox3 nad3 -nad5 nad2 -nad4 -nad4L nad6 cob -nad1 -rrnL -rrnS ]

g2: [ cox1 cox2 atp8 atp6 cox3 nad3 -nad5 -nad4 -nad4L nad6 nad2 cob -nad1 -rrnL -rrnS ]

g3: [ cox1 cox2 atp8 atp6 cox3 nad3 -nad5 -nad4 -nad4L nad6 cob -nad1 -rrnL nad2 -rrnS ]

score = 247:

model 4:

-------------

R(0,8) R(1,8) R(2,14) R(3,8) R(4,8) R(5,n1) R(6,n1) R(7,15) R(8,0) R(8,1) R(8,3) R(8,4) R(8,9) R(8,14) R(8,15) R(8,16) R(8,n2) R(8,n4) R(9,8) R(10,n4) R(11,n5) R(12,17) R(13,n3) R(14,2) R(14,8) R(15,7) R(15,8) R(16,8) R(16,17) R(17,12) R(17,16) R(n1,5) R(n1,6) R(n1,n2) R(n2,8) R(n2,n1) R(n2,n3) R(n3,13) R(n3,n2) R(n4,8) R(n4,10) R(n4,n5) R(n5,11) R(n5,n4)

-------------------------------------------------------------------------------------------------------------

-> form outgroup SOL3

n1(mod5)

[ cox1 cox2 nad2 atp8 atp6 cox3 nad3 -nad5 -nad4 -nad4L nad6 cob -nad1 -rrnL -rrnS ]

n2(mod5)

g1: [ cox1 cox2 atp8 atp6 cox3 nad3 -nad5 nad2 -nad4 -nad4L nad6 cob -nad1 -rrnL -rrnS ]

g2: [ cox1 cox2 atp8 atp6 cox3 nad3 -nad5 -nad4 -nad4L nad6 nad2 cob -nad1 -rrnL -rrnS ]

g3: [ cox1 cox2 atp8 atp6 cox3 nad3 -nad5 -nad4 -nad4L nad6 cob -nad1 -rrnL nad2 -rrnS ]

score = 247:

model 5:

-------------

R(0,8) R(1,8) R(2,14) R(3,8) R(4,8) R(5,n1) R(6,n1) R(7,15) R(8,0) R(8,1) R(8,3) R(8,4) R(8,9) R(8,14) R(8,15) R(8,16) R(8,n2) R(8,n5) R(9,8) R(10,n4) R(11,n5) R(12,17) R(13,n3) R(14,2) R(14,8) R(15,7) R(15,8) R(16,8) R(16,17) R(17,12) R(17,16) R(n1,5) R(n1,6) R(n1,n2) R(n2,8) R(n2,n1) R(n2,n3) R(n3,13) R(n3,n2) R(n4,10) R(n4,n5) R(n5,8) R(n5,11) R(n5,n4)

-------------------------------------------------------------------------------------------------------------

-> form outgroup SOL2

n1(mod6)

[ cox1 cox2 nad2 atp8 atp6 cox3 nad3 -nad5 -nad4 -nad4L nad6 cob -nad1 -rrnL -rrnS ]

n2(mod6)

g1: [ cox1 cox2 atp8 atp6 cox3 nad3 -nad5 nad2 -nad4 -nad4L nad6 cob -nad1 -rrnL -rrnS ]

g2: [ cox1 cox2 atp8 atp6 cox3 nad3 -nad5 -nad4 -nad4L nad6 nad2 cob -nad1 -rrnL -rrnS ]

g3: [ cox1 cox2 atp8 atp6 cox3 nad3 -nad5 -nad4 -nad4L nad6 cob -nad1 -rrnL nad2 -rrnS ]

score = 232:

model 6:

-------------

R(0,8) R(1,8) R(2,14) R(3,8) R(4,8) R(5,n1) R(6,n1) R(7,15) R(8,0) R(8,1) R(8,3) R(8,4) R(8,9) R(8,14) R(8,15) R(8,16) R(8,n2) R(8,n4) R(8,n5) R(9,8) R(10,n4) R(11,n5) R(12,17) R(13,n3) R(14,2) R(14,8) R(15,7) R(15,8) R(16,8) R(16,17) R(17,12) R(17,16) R(n1,5) R(n1,6) R(n1,n2) R(n2,8) R(n2,n1) R(n2,n3) R(n3,13) R(n3,n2) R(n4,8) R(n4,10) R(n5,8) R(n5,11)

-------------------------------------------------------------------------------------------------------------

-> the best model

-> form outgroup SOL2

n1(mod7)

[ cox1 cox2 nad2 atp8 atp6 cox3 nad3 -nad5 -nad4 -nad4L nad6 cob -nad1 -rrnL -rrnS ]

score = 204:

model 7:

-------------

R(0,8) R(1,8) R(2,14) R(3,8) R(4,8) R(5,n1) R(6,n1) R(7,15) R(8,0) R(8,1) R(8,3) R(8,4) R(8,9) R(8,14) R(8,15) R(8,16) R(8,n1) R(8,n2) R(8,n3) R(8,n4) R(9,8) R(10,n3) R(11,n2) R(12,17) R(13,n5) R(14,2) R(14,8) R(15,7) R(15,8) R(16,8) R(16,17) R(17,12) R(17,16) R(n1,5) R(n1,6) R(n1,8) R(n2,8) R(n2,11) R(n3,8) R(n3,10) R(n4,8) R(n4,n5) R(n5,13) R(n5,n4)

-------------------------------------------------------------------------------------------------------------

-> form outgroup SOL3

n1(mod8)

[ cox1 cox2 nad2 atp8 atp6 cox3 nad3 -nad5 -nad4 -nad4L nad6 cob -nad1 -rrnL -rrnS ]

score = 219:

model 8:

-------------

R(0,8) R(1,8) R(2,14) R(3,8) R(4,8) R(5,n1) R(6,n1) R(7,15) R(8,0) R(8,1) R(8,3) R(8,4) R(8,9) R(8,14) R(8,15) R(8,16) R(8,n1) R(8,n2) R(8,n3) R(9,8) R(10,n5) R(11,n2) R(12,17) R(13,n4) R(14,2) R(14,8) R(15,7) R(15,8) R(16,8) R(16,17) R(17,12) R(17,16) R(n1,5) R(n1,6) R(n1,8) R(n2,8) R(n2,11) R(n2,n5) R(n3,8) R(n3,n4) R(n4,13) R(n4,n3) R(n5,10) R(n5,n2)

-------------------------------------------------------------------------------------------------------------

-> form outgroup SOL1

n1(mod9)

[ cox1 cox2 nad2 atp8 atp6 cox3 nad3 -nad5 -nad4 -nad4L nad6 cob -nad1 -rrnL -rrnS ]

score = 219:

model 9:

-------------

R(0,8) R(1,8) R(2,14) R(3,8) R(4,8) R(5,n1) R(6,n1) R(7,15) R(8,0) R(8,1) R(8,3) R(8,4) R(8,9) R(8,14) R(8,15) R(8,16) R(8,n1) R(8,n2) R(8,n3) R(9,8) R(10,n2) R(11,n5) R(12,17) R(13,n4) R(14,2) R(14,8) R(15,7) R(15,8) R(16,8) R(16,17) R(17,12) R(17,16) R(n1,5) R(n1,6) R(n1,8) R(n2,8) R(n2,10) R(n2,n5) R(n3,8) R(n3,n4) R(n4,13) R(n4,n3) R(n5,11) R(n5,n2)

-------------------------------------------------------------------------------------------------------------

-> no other models

ecdysozoans_taxE_9sol

================================================================================

================================================================================

AXIOMS

================================================================================

================================================================================

{ the solutions of problem PHYLO are the smallest graphs T (defined on the smallest domain possible but containing at least all the OTUs) which verify properties P1 to P6:

P1- T is simple (the relation R(x, y) which defines graph T is not reflexive)

P2- T is non-oriented (the relation R(x, y) which defines graph T is symetrical)

P3- T is connected and acyclic (T is a tree)

P4- T respects the minimal distance matrix, i.e.:

for all couple of OTUs x and y, the length of the path x->y in T is always superior or equals to the minimal distance calculated between x and y (encoded in the minimal distance matrix)

P5- T respects other eventual hypothesis (Primary Phylogenetic Hypothesis = PPH)

used to impose the existence of given monophyletic groups

P6- it is possible to calculate all the values for each HTU in the graph T }

{ OTUs: }

limulus_polyphemus=0;

nymphon_gracile=1;

dermatophagoides_pteronyssinus=2;

steganacarus_magnus=3;

leptotrombidium_akamushi=4;

narceus_annularis=5; { = outgroup1 }

homo_sapiens=6; { = outgroup2 }

katharina_tunicata=7; { = outgroup3 }

{ THE OUTGROUP FORM "SOL2" IS FIXED: }

E x ( R(homo_sapiens,x) et

R(x,limulus_polyphemus)

);

E x ( R(katharina_tunicata,x) et

R(x,limulus_polyphemus)

);

{ PROPERTY P1: R(x, y) is not reflexive}

Q x (-R(x, x));

{ PROPERTY P2: R(x, y) is symetrical}

Q x y (R(x, y) => R(y, x));

{ PROPERTY P3: graph T is connected and acyclic (T is a tree) }

{

This property is verified by a constraint programmed in the model generator, instead of a "heavy" logical formula:

1- it will refuse the partial interpretations in which a connected component of the graph (in construction) is cyclic, i.e. such as: number of edges >= number of vertices

2- it will refuse the complete interpretations in which the constructed graph has more than one connected component

}

{ PROPERTY P4: graph T respects minimal distance matrix }

{

This property is verified by a constraint programmed in the model generator:

it will refuse the partial interpretations in which the graph (in construction) do not respect the minimal distance matrix, i.e. such as:

Let x, y a couple of OTUs,

let d = minimal distance calculated between x and y (encoded in the minimal distance matrix), there is a path of length k between x and y, with: k < d

The minimal distance matrix is encoded directly in the data structure of the model generator:

/* minimal distance matrix ECDYSOZOANS taxE: */

DIST[0][0]=0;

DIST[1][0]=1; DIST[1][1]=0;

DIST[2][0]=3; DIST[2][1]=3; DIST[2][2]=0;

DIST[3][0]=2; DIST[3][1]=3; DIST[3][2]=3; DIST[3][3]=0;

DIST[4][0]=5; DIST[4][1]=5; DIST[4][2]=6; DIST[4][3]=5; DIST[4][4]=0;

DIST[5][0]=1; DIST[5][1]=2; DIST[5][2]=3; DIST[5][3]=3; DIST[5][4]=5; DIST[5][5]=0;

DIST[6][0]=2; DIST[6][1]=2; DIST[6][2]=3; DIST[6][3]=3; DIST[6][4]=5; DIST[6][5]=2; DIST[6][6]=0;

DIST[7][0]=2; DIST[7][1]=3; DIST[7][2]=4; DIST[7][3]=4; DIST[7][4]=5; DIST[7][5]=2; DIST[7][6]=3; DIST[7][7]=0;

}

{ PROPERTY P5: graph T respects eventual Primary Phylogenetic Hypotheses }

{

This property is verified by constraints programmed in the model generator:

- monophyly of Ecdysozoa = (0,1,2,3,4,5)

- monophyly of Arthropoda = (0,1,2,3,4,5)

- monophyly of Chelicerata = (0,1,2,3,4)

- monophyly of Acari = (2,3,4)

}

{------------------------------------------------------------------------------------------------------------------------}

{ PROPERTY P6: it is possible to calculate all the values for each HTU in the graph T }

{

First we calculate with the model generator the set of tree solutions which verify properties P1 to P5. Property P6 is verified *a posteriori* for each tree solution, with a *feedback* mechanism:

Studying each tree solution for calculating the values of HTUs, we eventually discover "impossible sub-trees": they appear in tree solutions which verify P1 to P5, but they do not verify P6.

For each impossible subtree A, an additional constraint is programmed into the model generator to forbid the solutions containing A. Tree solutions are recalculated and verified, allowing the discovery of new impossible subtrees and the programming of new constraints to recalculate the solutions (feedback mechanism). Finally, the complete set of optimal solutions is determined after iteration of this process and elimination of all the solutions that do not verify P6.

}

================================================================================

================================================================================

SOLUTIONS

================================================================================

================================================================================

OTUs:

limulus_polyphemus=0;

nymphon_gracile=1;

dermatophagoides_pteronyssinus=2;

steganacarus_magnus=3;

leptotrombidium_akamushi=4;

narceus_annularis=5;

homo_sapiens=6;

katharina_tunicata=7;

HTUs:

n1, n2, n3, n4, n5, n6, n7, n8

D = [0,15]: 9 solutions OK (which verify property P6) (17 impossible sub-trees)

minimal score (best) = 28

maximal score = 45

-------------------------------------------------------------------------------------------------------------

n5(mod1)

g1: [ cox1 cox2 atp8 atp6 cox3 nad3 rrnS nad1 rrnL -nad5 -nad4 -nad4L nad6 cob nad2 ]

g2: [ cox1 cox2 atp8 atp6 cox3 nad3 nad1 rrnL -nad2 -cob -nad6 nad4L nad4 nad5 -rrnS ]

g3: [ cox1 cox2 atp8 atp6 cox3 nad3 rrnS -nad5 -nad4 -nad4L nad6 cob nad2 -rrnL -nad1 ]

score = 36:

model 1:

-------------

R(0,1) R(0,5) R(0,n1) R(0,n2) R(0,n3) R(1,0) R(2,n4) R(3,n1) R(3,n5) R(4,n6) R(5,0) R(6,n2) R(7,n3) R(n1,0) R(n1,3) R(n2,0) R(n2,6) R(n3,0) R(n3,7) R(n4,2) R(n4,n5) R(n5,3) R(n5,n4) R(n5,n7) R(n6,4) R(n6,n8) R(n7,n5) R(n7,n8) R(n8,n6) R(n8,n7)

-------------------------------------------------------------------------------------------------------------

n4(mod2)

g1: [ cox1 cox2 atp8 atp6 cox3 nad3 rrnS rrnL -nad1 -nad5 -nad4 -nad4L nad6 cob nad2 ]

g2: [ cox1 cox2 atp8 atp6 cox3 nad3 rrnS rrnL -nad5 -nad4 -nad4L nad6 nad1 cob nad2 ]

n5(mod2)

for n4-g1:

g1: [ cox1 cox2 atp8 atp6 cox3 nad3 rrnS nad1 rrnL -nad5 -nad4 -nad4L nad6 cob nad2 ]

for n4-g2:

g2: [ cox1 cox2 atp8 atp6 cox3 nad3 rrnS nad1 rrnL -nad5 -nad4 -nad4L nad6 cob nad2 ]

score = 36:

model 2:

-------------

R(0,1) R(0,5) R(0,n1) R(0,n2) R(0,n3) R(1,0) R(2,n4) R(3,n5) R(4,n6) R(5,0) R(6,n2) R(7,n3) R(n1,0) R(n1,n4) R(n2,0) R(n2,6) R(n3,0) R(n3,7) R(n4,2) R(n4,n1) R(n4,n5) R(n5,3) R(n5,n4) R(n5,n7) R(n6,4) R(n6,n8) R(n7,n5) R(n7,n8) R(n8,n6) R(n8,n7)

-------------------------------------------------------------------------------------------------------------

n5(mod3)

[ cox1 cox2 atp8 atp6 cox3 nad3 rrnS nad1 rrnL -nad5 -nad4 -nad4L nad6 cob nad2 ]

score = 31:

model 3:

-------------

R(0,1) R(0,5) R(0,n1) R(0,n2) R(0,n3) R(1,0) R(2,n4) R(3,n5) R(4,n6) R(5,0) R(6,n2) R(7,n3) R(n1,0) R(n1,n5) R(n2,0) R(n2,6) R(n3,0) R(n3,7) R(n4,2) R(n4,n5) R(n5,3) R(n5,n1) R(n5,n4) R(n5,n7) R(n6,4) R(n6,n8) R(n7,n5) R(n7,n8) R(n8,n6) R(n8,n7)

-------------------------------------------------------------------------------------------------------------

-> the best model (1/2)

n3(mod4)

[ cox1 cox2 atp8 atp6 cox3 nad3 -nad5 -nad4 -nad4L nad6 cob -rrnL -nad1 -rrnS nad2 ]

n5(mod4)

[ cox1 cox2 atp8 atp6 cox3 nad3 rrnS nad1 rrnL -nad5 -nad4 -nad4L nad6 cob nad2 ]

score = 28:

model 4:

-------------

R(0,1) R(0,5) R(0,n1) R(0,n2) R(0,n3) R(1,0) R(2,n4) R(3,n3) R(4,n6) R(5,0) R(6,n1) R(7,n2) R(n1,0) R(n1,6) R(n2,0) R(n2,7) R(n3,0) R(n3,3) R(n3,n5) R(n4,2) R(n4,n5) R(n5,n3) R(n5,n4) R(n5,n8) R(n6,4) R(n6,n7) R(n7,n6) R(n7,n8) R(n8,n5) R(n8,n7)

-------------------------------------------------------------------------------------------------------------

n3(mod5)

[ cox1 cox2 atp8 atp6 cox3 nad3 rrnS rrnL nad1 -cob -nad6 nad4L nad4 nad5 nad2 ]

n4(mod5)

[ cox1 cox2 atp8 atp6 cox3 nad3 rrnS rrnL -nad1 -nad5 -nad4 -nad4L nad6 cob nad2 ]

n5(mod5)

[ cox1 cox2 atp8 atp6 cox3 nad3 rrnS nad1 rrnL -nad5 -nad4 -nad4L nad6 cob nad2 ]

score = 37:

model 5:

-------------

R(0,5) R(0,n1) R(0,n2) R(0,n3) R(1,n3) R(2,n4) R(3,n5) R(4,n6) R(5,0) R(6,n1) R(7,n2) R(n1,0) R(n1,6) R(n2,0) R(n2,7) R(n3,0) R(n3,1) R(n3,n4) R(n4,2) R(n4,n3) R(n4,n5) R(n5,3) R(n5,n4) R(n5,n8) R(n6,4) R(n6,n7) R(n7,n6) R(n7,n8) R(n8,n5) R(n8,n7)

-------------------------------------------------------------------------------------------------------------

-> the best model (2/2)

n5(mod6)

g1: [ cox1 cox2 atp8 atp6 cox3 nad3 -rrnL -nad1 -rrnS -nad5 -nad4 -nad4L nad6 cob nad2 ]

g2: [ cox1 cox2 atp8 atp6 cox3 nad3 rrnS nad1 rrnL -nad5 -nad4 -nad4L nad6 cob nad2 ]

n3(mod6)

for n5-g1:

g1: [ cox1 cox2 atp8 atp6 cox3 nad3 -nad1 -rrnL -rrnS -nad5 -nad4 -nad4L nad6 cob nad2 ]

for n5-g2:

g2: [ cox1 cox2 atp8 atp6 cox3 nad3 rrnS rrnL nad1 -nad5 -nad4 -nad4L nad6 cob nad2 ]

g3: [ cox1 cox2 atp8 atp6 cox3 nad3 rrnS rrnL -nad5 -nad4 -nad4L nad6 cob -nad1 nad2 ]

score = 28:

model 6:

-------------

R(0,1) R(0,5) R(0,n1) R(0,n2) R(0,n3) R(1,0) R(2,n4) R(3,n5) R(4,n6) R(5,0) R(6,n1) R(7,n2) R(n1,0) R(n1,6) R(n2,0) R(n2,7) R(n3,0) R(n3,n4) R(n3,n5) R(n4,2) R(n4,n3) R(n5,3) R(n5,n3) R(n5,n8) R(n6,4) R(n6,n7) R(n7,n6) R(n7,n8) R(n8,n5) R(n8,n7)

-------------------------------------------------------------------------------------------------------------

n3(mod7)

[ cox1 cox2 rrnS rrnL -nad1 atp8 atp6 cox3 nad3 -nad5 -nad4 -nad4L nad6 cob nad2 ]

n5(mod7)

[ cox1 cox2 rrnS nad1 rrnL atp8 atp6 cox3 nad3 -nad5 -nad4 -nad4L nad6 cob nad2 ]

score = 37:

model 7:

-------------

R(0,1) R(0,5) R(0,n1) R(0,n2) R(1,0) R(1,n3) R(2,n4) R(3,n5) R(4,n6) R(5,0) R(6,n1) R(7,n2) R(n1,0) R(n1,6) R(n2,0) R(n2,7) R(n3,1) R(n3,n4) R(n3,n5) R(n4,2) R(n4,n3) R(n5,3) R(n5,n3) R(n5,n8) R(n6,4) R(n6,n7) R(n7,n6) R(n7,n8) R(n8,n5) R(n8,n7)

-------------------------------------------------------------------------------------------------------------

n6(mod8)

[ cox1 cox2 atp8 atp6 cox3 nad3 rrnS nad1 rrnL -nad5 -nad4 -nad4L nad6 cob nad2 ]

n4(mod8)

g1: [ cox1 cox2 rrnS nad1 rrnL -nad5 -nad4 -nad4L nad6 cob atp8 atp6 cox3 nad3 nad2 ]

g2: [ cox1 cox2 nad1 rrnL -nad5 -nad4 -nad4L nad6 cob atp8 atp6 cox3 nad3 rrnS nad2 ]

score = 45:

model 8:

-------------

R(0,1) R(0,5) R(0,n1) R(0,n2) R(1,0) R(1,n3) R(2,n7) R(3,n6) R(4,n8) R(5,0) R(6,n1) R(7,n2) R(n1,0) R(n1,6) R(n2,0) R(n2,7) R(n3,1) R(n3,n4) R(n4,n3) R(n4,n5) R(n4,n6) R(n5,n4) R(n5,n8) R(n6,3) R(n6,n4) R(n6,n7) R(n7,2) R(n7,n6) R(n8,4) R(n8,n5)

-------------------------------------------------------------------------------------------------------------

n4(mod9)

[ cox1 cox2 atp8 atp6 cox3 nad3 rrnS rrnL -nad1 -nad5 -nad4 -nad4L nad6 cob nad2 ]

n5(mod9)

[ cox1 cox2 atp8 atp6 cox3 nad3 rrnS nad1 rrnL -nad5 -nad4 -nad4L nad6 cob nad2 ]

score = 45:

model 9:

-------------

R(0,1) R(0,5) R(0,n1) R(0,n2) R(1,0) R(1,n3) R(2,n4) R(3,n5) R(4,n6) R(5,0) R(6,n1) R(7,n2) R(n1,0) R(n1,6) R(n2,0) R(n2,7) R(n3,1) R(n3,n4) R(n4,2) R(n4,n3) R(n4,n5) R(n5,3) R(n5,n4) R(n5,n8) R(n6,4) R(n6,n7) R(n7,n6) R(n7,n8) R(n8,n5) R(n8,n7)

-------------------------------------------------------------------------------------------------------------

-> no other models

ecdysozoans_taxF_27sol

================================================================================

================================================================================

AXIOMS

================================================================================

================================================================================

{ the solutions of problem PHYLO are the smallest graphs T (defined on the smallest domain possible but containing at least all the OTUs) which verify properties P1 to P6:

P1- T is simple (the relation R(x, y) which defines graph T is not reflexive)

P2- T is non-oriented (the relation R(x, y) which defines graph T is symetrical)

P3- T is connected and acyclic (T is a tree)

P4- T respects the minimal distance matrix, i.e.:

for all couple of OTUs x and y, the length of the path x->y in T is always superior or equals to the minimal distance calculated between x and y (encoded in the minimal distance matrix)

P5- T respects other eventual hypothesis (Primary Phylogenetic Hypothesis = PPH)

used to impose the existence of given monophyletic groups

P6- it is possible to calculate all the values for each HTU in the graph T }

{ OTUs: }

limulus_polyphemus=0;

nymphon_gracile=1;

dermatophagoides_pteronyssinus=2;

steganacarus_magnus=3;

leptotrombidium_akamushi=4;

narceus_annularis=5; { = outgroup1 }

homo_sapiens=6; { = outgroup2 }

katharina_tunicata=7; { = outgroup3 }

{ PROPERTY P1: R(x, y) is not reflexive}

Q x (-R(x, x));

{ PROPERTY P2: R(x, y) is symetrical}

Q x y (R(x, y) => R(y, x));

{ PROPERTY P3: graph T is connected and acyclic (T is a tree) }

{

This property is verified by a constraint programmed in the model generator, instead of a "heavy" logical formula:

1- it will refuse the partial interpretations in which a connected component of the graph (in construction) is cyclic, i.e. such as: number of edges >= number of vertices

2- it will refuse the complete interpretations in which the constructed graph has more than one connected component

}

{ PROPERTY P4: graph T respects minimal distance matrix }

{

This property is verified by a constraint programmed in the model generator:

it will refuse the partial interpretations in which the graph (in construction) do not respect the minimal distance matrix, i.e. such as:

Let x, y a couple of OTUs,

let d = minimal distance calculated between x and y (encoded in the minimal distance matrix), there is a path of length k between x and y, with: k < d

The minimal distance matrix is encoded directly in the data structure of the model generator:

/* minimal distance matrix ECDYSOZOANS taxF: */

DIST[0][0]=0;

DIST[1][0]=1; DIST[1][1]=0;

DIST[2][0]=3; DIST[2][1]=3; DIST[2][2]=0;

DIST[3][0]=2; DIST[3][1]=3; DIST[3][2]=3; DIST[3][3]=0;

DIST[4][0]=5; DIST[4][1]=5; DIST[4][2]=6; DIST[4][3]=5; DIST[4][4]=0;

DIST[5][0]=1; DIST[5][1]=2; DIST[5][2]=3; DIST[5][3]=3; DIST[5][4]=5; DIST[5][5]=0;

DIST[6][0]=2; DIST[6][1]=2; DIST[6][2]=3; DIST[6][3]=3; DIST[6][4]=5; DIST[6][5]=2; DIST[6][6]=0;

DIST[7][0]=2; DIST[7][1]=3; DIST[7][2]=4; DIST[7][3]=4; DIST[7][4]=5; DIST[7][5]=2; DIST[7][6]=3; DIST[7][7]=0;

}

{ PROPERTY P5: graph T respects eventual Primary Phylogenetic Hypotheses }

{

This property is verified by constraints programmed in the model generator:

- monophyly of Ecdysozoa = (0,1,2,3,4,5)

- monophyly of Arthropoda = (0,1,2,3,4,5)

- monophyly of Chelicerata = (0,1,2,3,4)

- monophyly of Acari = (2,3,4)

}

{------------------------------------------------------------------------------------------------------------------------}

{ PROPERTY P6: it is possible to calculate all the values for each HTU in the graph T }

{

First we calculate with the model generator the set of tree solutions which verify properties P1 to P5. Property P6 is verified *a posteriori* for each tree solution, with a *feedback* mechanism:

Studying each tree solution for calculating the values of HTUs, we eventually discover "impossible sub-trees": they appear in tree solutions which verify P1 to P5, but they do not verify P6.

For each impossible subtree A, an additional constraint is programmed into the model generator to forbid the solutions containing A. Tree solutions are recalculated and verified, allowing the discovery of new impossible subtrees and the programming of new constraints to recalculate the solutions (feedback mechanism). Finally, the complete set of optimal solutions is determined after iteration of this process and elimination of all the solutions that do not verify P6.

}

================================================================================

================================================================================

SOLUTIONS

================================================================================

================================================================================

OTUs:

limulus_polyphemus=0;

nymphon_gracile=1;

dermatophagoides_pteronyssinus=2;

steganacarus_magnus=3;

leptotrombidium_akamushi=4;

narceus_annularis=5;

homo_sapiens=6;

katharina_tunicata=7;

HTUs:

n1, n2, n3, n4, n5, n6, n7, n8

D = [0,15]: 27 solutions OK (which verify property P6 (17 impossible sub-trees)

minimal score (best) = 28

maximal score = 50

-------------------------------------------------------------------------------------------------------------

score = 50:

model 1:

-------------

R(0,1) R(0,5) R(0,n6) R(1,0) R(1,n5) R(2,n3) R(3,n4) R(4,n8) R(5,0) R(6,n7) R(7,n6) R(n1,n2) R(n1,n8) R(n2,n1) R(n2,n4) R(n2,n5) R(n3,2) R(n3,n4) R(n4,3) R(n4,n2) R(n4,n3) R(n5,1) R(n5,n2) R(n6,0) R(n6,7) R(n6,n7) R(n7,6) R(n7,n6) R(n8,4) R(n8,n1)

-------------------------------------------------------------------------------------------------------------

score = 33:

model 2:

-------------

R(0,1) R(0,5) R(0,n5) R(0,n6) R(1,0) R(2,n3) R(3,n5) R(4,n8) R(5,0) R(6,n7) R(7,n6) R(n1,n2) R(n1,n8) R(n2,n1) R(n2,n4) R(n3,2) R(n3,n4) R(n4,n2) R(n4,n3) R(n4,n5) R(n5,0) R(n5,3) R(n5,n4) R(n6,0) R(n6,7) R(n6,n7) R(n7,6) R(n7,n6) R(n8,4) R(n8,n1)

-------------------------------------------------------------------------------------------------------------

score = 36:

model 3:

-------------

R(0,1) R(0,5) R(0,n5) R(0,n6) R(1,0) R(2,n3) R(3,n4) R(4,n8) R(5,0) R(6,n7) R(7,n6) R(n1,n2) R(n1,n8) R(n2,n1) R(n2,n4) R(n3,2) R(n3,n4) R(n4,3) R(n4,n2) R(n4,n3) R(n4,n5) R(n5,0) R(n5,n4) R(n6,0) R(n6,7) R(n6,n7) R(n7,6) R(n7,n6) R(n8,4) R(n8,n1)

-------------------------------------------------------------------------------------------------------------

score = 33:

model 4:

-------------

R(0,1) R(0,5) R(0,n5) R(0,n6) R(1,0) R(2,n3) R(3,n4) R(4,n8) R(5,0) R(6,n7) R(7,n6) R(n1,n2) R(n1,n8) R(n2,n1) R(n2,n4) R(n3,2) R(n3,n5) R(n4,3) R(n4,n2) R(n4,n5) R(n5,0) R(n5,n3) R(n5,n4) R(n6,0) R(n6,7) R(n6,n7) R(n7,6) R(n7,n6) R(n8,4) R(n8,n1)

-------------------------------------------------------------------------------------------------------------

score = 42:

model 5:

-------------

R(0,1) R(0,5) R(0,n6) R(1,0) R(1,n5) R(2,n3) R(3,n4) R(4,n8) R(5,0) R(6,n7) R(7,n6) R(n1,n2) R(n1,n8) R(n2,n1) R(n2,n4) R(n3,2) R(n3,n5) R(n4,3) R(n4,n2) R(n4,n5) R(n5,1) R(n5,n3) R(n5,n4) R(n6,0) R(n6,7) R(n6,n7) R(n7,6) R(n7,n6) R(n8,4) R(n8,n1)

-------------------------------------------------------------------------------------------------------------

score = 41:

model 6:

-------------

R(0,1) R(0,5) R(0,n5) R(0,n6) R(1,0) R(2,n3) R(3,n4) R(3,n5) R(4,n8) R(5,0) R(6,n7) R(7,n6) R(n1,n2) R(n1,n8) R(n2,n1) R(n2,n4) R(n3,2) R(n3,n4) R(n4,3) R(n4,n2) R(n4,n3) R(n5,0) R(n5,3) R(n6,0) R(n6,7) R(n6,n7) R(n7,6) R(n7,n6) R(n8,4) R(n8,n1)

-------------------------------------------------------------------------------------------------------------

score = 41:

model 7:

-------------

R(0,1) R(0,5) R(0,n5) R(0,n6) R(1,0) R(2,n3) R(3,n4) R(4,n8) R(5,0) R(6,n7) R(7,n6) R(n1,n2) R(n1,n8) R(n2,n1) R(n2,n4) R(n3,2) R(n3,n4) R(n3,n5) R(n4,3) R(n4,n2) R(n4,n3) R(n5,0) R(n5,n3) R(n6,0) R(n6,7) R(n6,n7) R(n7,6) R(n7,n6) R(n8,4) R(n8,n1)

-------------------------------------------------------------------------------------------------------------

score = 50:

model 8:

-------------

R(0,1) R(0,5) R(0,n6) R(1,0) R(1,n5) R(2,n3) R(3,n4) R(4,n8) R(5,0) R(6,n7) R(7,n6) R(n1,n2) R(n1,n8) R(n2,n1) R(n2,n4) R(n3,2) R(n3,n4) R(n3,n5) R(n4,3) R(n4,n2) R(n4,n3) R(n5,1) R(n5,n3) R(n6,0) R(n6,7) R(n6,n7) R(n7,6) R(n7,n6) R(n8,4) R(n8,n1)

-------------------------------------------------------------------------------------------------------------

score = 42:

model 9:

-------------

R(0,5) R(0,n5) R(0,n6) R(1,n5) R(2,n3) R(3,n4) R(4,n8) R(5,0) R(6,n7) R(7,n6) R(n1,n2) R(n1,n8) R(n2,n1) R(n2,n4) R(n3,2) R(n3,n4) R(n3,n5) R(n4,3) R(n4,n2) R(n4,n3) R(n5,0) R(n5,1) R(n5,n3) R(n6,0) R(n6,7) R(n6,n7) R(n7,6) R(n7,n6) R(n8,4) R(n8,n1)

-------------------------------------------------------------------------------------------------------------

score = 45:

model 10:

-------------

R(0,1) R(0,5) R(0,n6) R(0,n7) R(1,0) R(1,n5) R(2,n3) R(3,n4) R(4,n8) R(5,0) R(6,n7) R(7,n6) R(n1,n2) R(n1,n8) R(n2,n1) R(n2,n4) R(n2,n5) R(n3,2) R(n3,n4) R(n4,3) R(n4,n2) R(n4,n3) R(n5,1) R(n5,n2) R(n6,0) R(n6,7) R(n7,0) R(n7,6) R(n8,4) R(n8,n1)

-------------------------------------------------------------------------------------------------------------

-> the best model (1/2)

score = 28:

model 11:

-------------

R(0,1) R(0,5) R(0,n5) R(0,n6) R(0,n7) R(1,0) R(2,n3) R(3,n5) R(4,n8) R(5,0) R(6,n7) R(7,n6) R(n1,n2) R(n1,n8) R(n2,n1) R(n2,n4) R(n3,2) R(n3,n4) R(n4,n2) R(n4,n3) R(n4,n5) R(n5,0) R(n5,3) R(n5,n4) R(n6,0) R(n6,7) R(n7,0) R(n7,6) R(n8,4) R(n8,n1)

-------------------------------------------------------------------------------------------------------------

score = 31:

model 12:

-------------

R(0,1) R(0,5) R(0,n5) R(0,n6) R(0,n7) R(1,0) R(2,n3) R(3,n4) R(4,n8) R(5,0) R(6,n7) R(7,n6) R(n1,n2) R(n1,n8) R(n2,n1) R(n2,n4) R(n3,2) R(n3,n4) R(n4,3) R(n4,n2) R(n4,n3) R(n4,n5) R(n5,0) R(n5,n4) R(n6,0) R(n6,7) R(n7,0) R(n7,6) R(n8,4) R(n8,n1)

-------------------------------------------------------------------------------------------------------------

-> the best model (2/2)

score = 28:

model 13:

-------------

R(0,1) R(0,5) R(0,n5) R(0,n6) R(0,n7) R(1,0) R(2,n3) R(3,n4) R(4,n8) R(5,0) R(6,n7) R(7,n6) R(n1,n2) R(n1,n8) R(n2,n1) R(n2,n4) R(n3,2) R(n3,n5) R(n4,3) R(n4,n2) R(n4,n5) R(n5,0) R(n5,n3) R(n5,n4) R(n6,0) R(n6,7) R(n7,0) R(n7,6) R(n8,4) R(n8,n1)

-------------------------------------------------------------------------------------------------------------

score = 37:

model 14:

-------------

R(0,1) R(0,5) R(0,n6) R(0,n7) R(1,0) R(1,n5) R(2,n3) R(3,n4) R(4,n8) R(5,0) R(6,n7) R(7,n6) R(n1,n2) R(n1,n8) R(n2,n1) R(n2,n4) R(n3,2) R(n3,n5) R(n4,3) R(n4,n2) R(n4,n5) R(n5,1) R(n5,n3) R(n5,n4) R(n6,0) R(n6,7) R(n7,0) R(n7,6) R(n8,4) R(n8,n1)

-------------------------------------------------------------------------------------------------------------

score = 36:

model 15:

-------------

R(0,1) R(0,5) R(0,n5) R(0,n6) R(0,n7) R(1,0) R(2,n3) R(3,n4) R(3,n5) R(4,n8) R(5,0) R(6,n7) R(7,n6) R(n1,n2) R(n1,n8) R(n2,n1) R(n2,n4) R(n3,2) R(n3,n4) R(n4,3) R(n4,n2) R(n4,n3) R(n5,0) R(n5,3) R(n6,0) R(n6,7) R(n7,0) R(n7,6) R(n8,4) R(n8,n1)

-------------------------------------------------------------------------------------------------------------

score = 36:

model 16:

-------------

R(0,1) R(0,5) R(0,n5) R(0,n6) R(0,n7) R(1,0) R(2,n3) R(3,n4) R(4,n8) R(5,0) R(6,n7) R(7,n6) R(n1,n2) R(n1,n8) R(n2,n1) R(n2,n4) R(n3,2) R(n3,n4) R(n3,n5) R(n4,3) R(n4,n2) R(n4,n3) R(n5,0) R(n5,n3) R(n6,0) R(n6,7) R(n7,0) R(n7,6) R(n8,4) R(n8,n1)

-------------------------------------------------------------------------------------------------------------

score = 45:

model 17:

-------------

R(0,1) R(0,5) R(0,n6) R(0,n7) R(1,0) R(1,n5) R(2,n3) R(3,n4) R(4,n8) R(5,0) R(6,n7) R(7,n6) R(n1,n2) R(n1,n8) R(n2,n1) R(n2,n4) R(n3,2) R(n3,n4) R(n3,n5) R(n4,3) R(n4,n2) R(n4,n3) R(n5,1) R(n5,n3) R(n6,0) R(n6,7) R(n7,0) R(n7,6) R(n8,4) R(n8,n1)

-------------------------------------------------------------------------------------------------------------

score = 37:

model 18:

-------------

R(0,5) R(0,n5) R(0,n6) R(0,n7) R(1,n5) R(2,n3) R(3,n4) R(4,n8) R(5,0) R(6,n7) R(7,n6) R(n1,n2) R(n1,n8) R(n2,n1) R(n2,n4) R(n3,2) R(n3,n4) R(n3,n5) R(n4,3) R(n4,n2) R(n4,n3) R(n5,0) R(n5,1) R(n5,n3) R(n6,0) R(n6,7) R(n7,0) R(n7,6) R(n8,4) R(n8,n1)

-------------------------------------------------------------------------------------------------------------

score = 50:

model 19:

-------------

R(0,1) R(0,5) R(0,n7) R(1,0) R(1,n5) R(2,n3) R(3,n4) R(4,n8) R(5,0) R(6,n7) R(7,n6) R(n1,n2) R(n1,n8) R(n2,n1) R(n2,n4) R(n2,n5) R(n3,2) R(n3,n4) R(n4,3) R(n4,n2) R(n4,n3) R(n5,1) R(n5,n2) R(n6,7) R(n6,n7) R(n7,0) R(n7,6) R(n7,n6) R(n8,4) R(n8,n1)

-------------------------------------------------------------------------------------------------------------

score = 33:

model 20:

-------------

R(0,1) R(0,5) R(0,n5) R(0,n7) R(1,0) R(2,n3) R(3,n5) R(4,n8) R(5,0) R(6,n7) R(7,n6) R(n1,n2) R(n1,n8) R(n2,n1) R(n2,n4) R(n3,2) R(n3,n4) R(n4,n2) R(n4,n3) R(n4,n5) R(n5,0) R(n5,3) R(n5,n4) R(n6,7) R(n6,n7) R(n7,0) R(n7,6) R(n7,n6) R(n8,4) R(n8,n1)

-------------------------------------------------------------------------------------------------------------

score = 36:

model 21:

-------------

R(0,1) R(0,5) R(0,n5) R(0,n7) R(1,0) R(2,n3) R(3,n4) R(4,n8) R(5,0) R(6,n7) R(7,n6) R(n1,n2) R(n1,n8) R(n2,n1) R(n2,n4) R(n3,2) R(n3,n4) R(n4,3) R(n4,n2) R(n4,n3) R(n4,n5) R(n5,0) R(n5,n4) R(n6,7) R(n6,n7) R(n7,0) R(n7,6) R(n7,n6) R(n8,4) R(n8,n1)

-------------------------------------------------------------------------------------------------------------

score = 33:

model 22:

-------------

R(0,1) R(0,5) R(0,n5) R(0,n7) R(1,0) R(2,n3) R(3,n4) R(4,n8) R(5,0) R(6,n7) R(7,n6) R(n1,n2) R(n1,n8) R(n2,n1) R(n2,n4) R(n3,2) R(n3,n5) R(n4,3) R(n4,n2) R(n4,n5) R(n5,0) R(n5,n3) R(n5,n4) R(n6,7) R(n6,n7) R(n7,0) R(n7,6) R(n7,n6) R(n8,4) R(n8,n1)

-------------------------------------------------------------------------------------------------------------

score = 42:

model 23:

-------------

R(0,1) R(0,5) R(0,n7) R(1,0) R(1,n5) R(2,n3) R(3,n4) R(4,n8) R(5,0) R(6,n7) R(7,n6) R(n1,n2) R(n1,n8) R(n2,n1) R(n2,n4) R(n3,2) R(n3,n5) R(n4,3) R(n4,n2) R(n4,n5) R(n5,1) R(n5,n3) R(n5,n4) R(n6,7) R(n6,n7) R(n7,0) R(n7,6) R(n7,n6) R(n8,4) R(n8,n1)

-------------------------------------------------------------------------------------------------------------

score = 41:

model 24:

-------------

R(0,1) R(0,5) R(0,n5) R(0,n7) R(1,0) R(2,n3) R(3,n4) R(3,n5) R(4,n8) R(5,0) R(6,n7) R(7,n6) R(n1,n2) R(n1,n8) R(n2,n1) R(n2,n4) R(n3,2) R(n3,n4) R(n4,3) R(n4,n2) R(n4,n3) R(n5,0) R(n5,3) R(n6,7) R(n6,n7) R(n7,0) R(n7,6) R(n7,n6) R(n8,4) R(n8,n1)

-------------------------------------------------------------------------------------------------------------

score = 41:

model 25:

-------------

R(0,1) R(0,5) R(0,n5) R(0,n7) R(1,0) R(2,n3) R(3,n4) R(4,n8) R(5,0) R(6,n7) R(7,n6) R(n1,n2) R(n1,n8) R(n2,n1) R(n2,n4) R(n3,2) R(n3,n4) R(n3,n5) R(n4,3) R(n4,n2) R(n4,n3) R(n5,0) R(n5,n3) R(n6,7) R(n6,n7) R(n7,0) R(n7,6) R(n7,n6) R(n8,4) R(n8,n1)

-------------------------------------------------------------------------------------------------------------

score = 50:

model 26:

-------------

R(0,1) R(0,5) R(0,n7) R(1,0) R(1,n5) R(2,n3) R(3,n4) R(4,n8) R(5,0) R(6,n7) R(7,n6) R(n1,n2) R(n1,n8) R(n2,n1) R(n2,n4) R(n3,2) R(n3,n4) R(n3,n5) R(n4,3) R(n4,n2) R(n4,n3) R(n5,1) R(n5,n3) R(n6,7) R(n6,n7) R(n7,0) R(n7,6) R(n7,n6) R(n8,4) R(n8,n1)

-------------------------------------------------------------------------------------------------------------

score = 42:

model 27:

-------------

R(0,5) R(0,n5) R(0,n7) R(1,n5) R(2,n3) R(3,n4) R(4,n8) R(5,0) R(6,n7) R(7,n6) R(n1,n2) R(n1,n8) R(n2,n1) R(n2,n4) R(n3,2) R(n3,n4) R(n3,n5) R(n4,3) R(n4,n2) R(n4,n3) R(n5,0) R(n5,1) R(n5,n3) R(n6,7) R(n6,n7) R(n7,0) R(n7,6) R(n7,n6) R(n8,4) R(n8,n1)

-------------------------------------------------------------------------------------------------------------

-> no other models

ecdysozoans_taxG_7sol

================================================================================

================================================================================

AXIOMS

================================================================================

================================================================================

{ the solutions of problem PHYLO are the smallest graphs T (defined on the smallest domain possible but containing at least all the OTUs) which verify properties P1 to P6:

P1- T is simple (the relation R(x, y) which defines graph T is not reflexive)

P2- T is non-oriented (the relation R(x, y) which defines graph T is symetrical)

P3- T is connected and acyclic (T is a tree)

P4- T respects the minimal distance matrix, i.e.:

for all couple of OTUs x and y, the length of the path x->y in T is always superior or equals to the minimal distance calculated between x and y (encoded in the minimal distance matrix)

P5- T respects other eventual hypothesis (Primary Phylogenetic Hypothesis = PPH)

used to impose the existence of given monophyletic groups

P6- it is possible to calculate all the values for each HTU in the graph T }

{ OTUs: }

limulus_polyphemus=0;

nymphon_gracile=1;

dermatophagoides_pteronyssinus=2;

steganacarus_magnus=3;

leptotrombidium_akamushi=4;

narceus_annularis=5; { = outgroup1 }

homo_sapiens=6; { = outgroup2 }

katharina_tunicata=7; { = outgroup3 }

epiperipatus_biolleyi=8;

priapulus_caudatus=9;

trichinella_spiralis=10;

{ AUXILLIARY CONSTANTS used to fix a part of the solution: }

G1=11; G2=12; G3=13;

G4=14; G5=15; G6=16; { used to fix the arthropoda group }

{ THE ARTHROPODA GROUP IS FIXED: }

R(limulus_polyphemus,narceus_annularis);

Q x ( x<>limulus_polyphemus

=>

-R(narceus_annularis,x)

);

R(limulus_polyphemus,nymphon_gracile);

Q x ( x<>limulus_polyphemus

=>

-R(nymphon_gracile,x)

);

R(limulus_polyphemus,G1);

R(G1,steganacarus_magnus);

R(G1,G2);

R(G2,G3);

R(G3,dermatophagoides_pteronyssinus);

R(G2,G4);

R(G4,G5);

R(G5,G6);

R(G6,leptotrombidium_akamushi);

Q x ( ( x<>limulus_polyphemus et

x<>steganacarus_magnus et

x<>G2

)

=>

-R(G1,x)

);

Q x ( x<>G1

=>

-R(steganacarus_magnus,x)

);

Q x ( ( x<>G1 et

x<>G3 et

x<>G4

)

=>

-R(G2,x)

);

Q x ( ( x<>G2 et

x<>dermatophagoides_pteronyssinus

)

=>

-R(G3,x)

);

Q x ( x<>G3

=>

-R(dermatophagoides_pteronyssinus,x)

);

Q x ( ( x<>G2 et

x<>G5

)

=>

-R(G4,x)

);

Q x ( ( x<>G4 et

x<>G6

)

=>

-R(G5,x)

);

Q x ( ( x<>G5 et

x<>leptotrombidium_akamushi

)

=>

-R(G6,x)

);

Q x ( x<>G6

=>

-R(leptotrombidium_akamushi,x)

);

{ PROPERTY P1: R(x, y) is not reflexive}

Q x (-R(x, x));

{ PROPERTY P2: R(x, y) is symetrical}

Q x y (R(x, y) => R(y, x));

{ PROPERTY P3: graph T is connected and acyclic (T is a tree) }

{

This property is verified by a constraint programmed in the model generator, instead of a "heavy" logical formula:

1- it will refuse the partial interpretations in which a connected component of the graph (in construction) is cyclic, i.e. such as: number of edges >= number of vertices

2- it will refuse the complete interpretations in which the constructed graph has more than one connected component

}

{ PROPERTY P4: graph T respects minimal distance matrix }

{

This property is verified by a constraint programmed in the model generator:

it will refuse the partial interpretations in which the graph (in construction) do not respect the minimal distance matrix, i.e. such as:

Let x, y a couple of OTUs,

let d = minimal distance calculated between x and y (encoded in the minimal distance matrix), there is a path of length k between x and y, with: k < d

The minimal distance matrix is encoded directly in the data structure of the model generator:

/* minimal distance matrix ECDYSOZOANS taxG: */

DIST[0][0]=0;

DIST[1][0]=1; DIST[1][1]=0;

DIST[2][0]=3; DIST[2][1]=3; DIST[2][2]=0;

DIST[3][0]=2; DIST[3][1]=3; DIST[3][2]=3; DIST[3][3]=0;

DIST[4][0]=5; DIST[4][1]=5; DIST[4][2]=6; DIST[4][3]=5; DIST[4][4]=0;

DIST[5][0]=1; DIST[5][1]=2; DIST[5][2]=3; DIST[5][3]=3; DIST[5][4]=5; DIST[5][5]=0;

DIST[6][0]=2; DIST[6][1]=2; DIST[6][2]=3; DIST[6][3]=3; DIST[6][4]=5; DIST[6][5]=2; DIST[6][6]=0;

DIST[7][0]=2; DIST[7][1]=3; DIST[7][2]=4; DIST[7][3]=4; DIST[7][4]=5; DIST[7][5]=2; DIST[7][6]=3; DIST[7][7]=0;

 DIST[8][0]=3; DIST[8][1]=4; DIST[8][2]=4; DIST[8][3]=4; DIST[8][4]=5; DIST[8][5]=4; DIST[8][6]=3; DIST[8][7]=4; DIST[8][8]=0;

DIST[9][0]=1; DIST[9][1]=1; DIST[9][2]=2; DIST[9][3]=3; DIST[9][4]=5; DIST[9][5]=2; DIST[9][6]=2; DIST[9][7]=2; DIST[9][8]=4; DIST[9][9]=0;

DIST[10][0]=4; DIST[10][1]=4; DIST[10][2]=4; DIST[10][3]=4; DIST[10][4]=5; DIST[10][5]=4; DIST[10][6]=4; DIST[10][7]=5; DIST[10][8]=6; DIST[10][9]=3; DIST[10][10]=0;

}

{ PROPERTY P5: graph T respects eventual Primary Phylogenetic Hypotheses }

{

This property is verified by constraints programmed in the model generator:

- monophyly of Ecdysozoa = (0,1,2,3,4,5,8,9,10)

- monophyly of Arthropoda = (0,1,2,3,4,5)

- monophyly of Chelicerata = (0,1,2,3,4)

- monophyly of Acari = (2,3,4)

- monophyly of Panarthropoda = (0,1,2,3,4,5,8)

- monophyly of Introverta = (9,10)

}

{------------------------------------------------------------------------------------------------------------------------}

{ PROPERTY P6: it is possible to calculate all the values for each HTU in the graph T }

{

First we calculate with the model generator the set of tree solutions which verify properties P1 to P5. Property P6 is verified *a posteriori* for each tree solution, with a *feedback* mechanism:

Studying each tree solution for calculating the values of HTUs, we eventually discover "impossible sub-trees": they appear in tree solutions which verify P1 to P5, but they do not verify P6.

For each impossible subtree A, an additional constraint is programmed into the model generator to forbid the solutions containing A. Tree solutions are recalculated and verified, allowing the discovery of new impossible subtrees and the programming of new constraints to recalculate the solutions (feedback mechanism). Finally, the complete set of optimal solutions is determined after iteration of this process and elimination of all the solutions that do not verify P6.

}

================================================================================

================================================================================

SOLUTIONS

================================================================================

================================================================================

OTUs:

limulus_polyphemus=0;

nymphon_gracile=1;

dermatophagoides_pteronyssinus=2;

steganacarus_magnus=3;

leptotrombidium_akamushi=4;

narceus_annularis=5;

homo_sapiens=6;

katharina_tunicata=7;

epiperipatus_biolleyi=8;

priapulus_caudatus=9;

trichinella_spiralis=10;

AUXILLIARY CONSTANTS used to fix a part of the solution:

G1=11; G2=12; G3=13;

G4=14; G5=15; G6=16; { used to fix the arthropoda group }

HTUs:

n1, n2, n3, n4, n5, n6, n7, n8 ???

D = [0,22]: 7 solutions OK (which verify property P6) (36 impossible sub-trees)

minimal score (best) = 380

maximal score = 416

-------------------------------------------------------------------------------------------------------------

-> the best model

-> form outgroup SOL2

score = 380:

model 1:

-------------

R(0,1) R(0,5) R(0,9) R(0,11) R(0,n1) R(0,n2) R(0,n3) R(1,0) R(2,13) R(3,11) R(4,16) R(5,0) R(6,n1) R(7,n2) R(8,n6) R(9,0) R(9,n4) R(10,n5) R(11,0) R(11,3) R(11,12) R(12,11) R(12,13) R(12,14) R(13,2) R(13,12) R(14,12) R(14,15) R(15,14) R(15,16) R(16,4) R(16,15) R(n1,0) R(n1,6) R(n2,0) R(n2,7) R(n3,0) R(n3,n6) R(n4,9) R(n4,n5) R(n5,10) R(n5,n4) R(n6,8) R(n6,n3)

-------------------------------------------------------------------------------------------------------------

-> form outgroup SOL3

n1(mod2)

[ cox1 cox2 atp8 atp6 cox3 nad3 -nad5 -nad4 -nad4L -cob -nad6 -nad1 -rrnL -rrnS nad2 ]

score= 394:

model 2:

-------------

R(0,1) R(0,5) R(0,9) R(0,11) R(0,n1) R(0,n2) R(1,0) R(2,13) R(3,11) R(4,16) R(5,0) R(6,n3) R(7,n1) R(8,n4) R(9,0) R(9,n6) R(10,n5) R(11,0) R(11,3) R(11,12) R(12,11) R(12,13) R(12,14) R(13,2) R(13,12) R(14,12) R(14,15) R(15,14) R(15,16) R(16,4) R(16,15) R(n1,0) R(n1,7) R(n1,n3) R(n2,0) R(n2,n4) R(n3,6) R(n3,n1) R(n4,8) R(n4,n2) R(n5,10) R(n5,n6) R(n6,9) R(n6,n5)

-------------------------------------------------------------------------------------------------------------

-> form outgroup SOL1

n1(mod3)

[ cox1 cox2 atp8 atp6 cox3 nad3 -nad5 -nad4 -nad4L nad6 cob rrnS rrnL nad1 nad2 ]

score= 394:

model 3:

-------------

R(0,1) R(0,5) R(0,9) R(0,11) R(0,n1) R(0,n2) R(1,0) R(2,13) R(3,11) R(4,16) R(5,0) R(6,n1) R(7,n3) R(8,n4) R(9,0) R(9,n6) R(10,n5) R(11,0) R(11,3) R(11,12) R(12,11) R(12,13) R(12,14) R(13,2) R(13,12) R(14,12) R(14,15) R(15,14) R(15,16) R(16,4) R(16,15) R(n1,0) R(n1,6) R(n1,n3) R(n2,0) R(n2,n4) R(n3,7) R(n3,n1) R(n4,8) R(n4,n2) R(n5,10) R(n5,n6) R(n6,9) R(n6,n5)

-------------------------------------------------------------------------------------------------------------

-> form outgroup SOL1 (but with Ur-ecdysozoa=n1)

n1(mod4)

[ cox1 cox2 atp8 atp6 cox3 nad3 -nad5 -nad4 -nad4L nad6 cob rrnS rrnL nad1 nad2 ]

score= 416:

model 4:

-------------

R(0,1) R(0,5) R(0,11) R(0,n1) R(0,n2) R(1,0) R(2,13) R(3,11) R(4,16) R(5,0) R(6,n1) R(7,n3) R(8,n5) R(9,n1) R(9,n4) R(10,n6) R(11,0) R(11,3) R(11,12) R(12,11) R(12,13) R(12,14) R(13,2) R(13,12) R(14,12) R(14,15) R(15,14) R(15,16) R(16,4) R(16,15) R(n1,0) R(n1,6) R(n1,9) R(n1,n3) R(n2,0) R(n2,n5) R(n3,7) R(n3,n1) R(n4,9) R(n4,n6) R(n5,8) R(n5,n2) R(n6,10) R(n6,n4)

-------------------------------------------------------------------------------------------------------------

-> form outgroup SOL1 (but with Ur-ecdysozoa=n1)

n1(mod5)

[ cox1 cox2 atp8 atp6 cox3 nad3 -nad5 -nad4 -nad4L nad6 cob rrnS rrnL nad1 nad2 ]

score= 402:

model 5:

-------------

R(0,1) R(0,5) R(0,11) R(0,n1) R(0,n2) R(1,0) R(2,13) R(3,11) R(4,16) R(5,0) R(6,n1) R(7,n3) R(8,n4) R(9,n1) R(10,n5) R(11,0) R(11,3) R(11,12) R(12,11) R(12,13) R(12,14) R(13,2) R(13,12) R(14,12) R(14,15) R(15,14) R(15,16) R(16,4) R(16,15) R(n1,0) R(n1,6) R(n1,9) R(n1,n3) R(n1,n6) R(n2,0) R(n2,n4) R(n3,7) R(n3,n1) R(n4,8) R(n4,n2) R(n5,10) R(n5,n6) R(n6,n1) R(n6,n5)

-------------------------------------------------------------------------------------------------------------

-> form SOL1_ALTER

-> Ur-ecdysozoa = 9 = priapulus_caudatus

n3(mod6)

[ cox1 cox2 atp8 atp6 cox3 nad3 -nad5 -nad4 -nad4L nad6 cob rrnS rrnL nad1 nad2 ]

score= 416:

model 6:

-------------

R(0,1) R(0,5) R(0,9) R(0,11) R(0,n1) R(1,0) R(2,13) R(3,11) R(4,16) R(5,0) R(6,n3) R(7,n5) R(8,n2) R(9,0) R(9,n3) R(9,n6) R(10,n4) R(11,0) R(11,3) R(11,12) R(12,11) R(12,13) R(12,14) R(13,2) R(13,12) R(14,12) R(14,15) R(15,14) R(15,16) R(16,4) R(16,15) R(n1,0) R(n1,n2) R(n2,8) R(n2,n1) R(n3,6) R(n3,9) R(n3,n5) R(n4,10) R(n4,n6) R(n5,7) R(n5,n3) R(n6,9) R(n6,n4)

-------------------------------------------------------------------------------------------------------------

-> form SOL2_ALTER

-> Ur-ecdysozoa = 9 = priapulus_caudatus

n3(mod7)

[ cox1 cox2 atp8 atp6 cox3 nad3 -nad5 -nad4 -nad4L nad6 cob rrnS rrnL nad1 nad2 ]

score= 402:

model 7:

-------------

R(0,1) R(0,5) R(0,9) R(0,11) R(0,n1) R(1,0) R(2,13) R(3,11) R(4,16) R(5,0) R(6,n3) R(7,n5) R(8,n2) R(9,0) R(9,n3) R(9,n5) R(9,n6) R(10,n4) R(11,0) R(11,3) R(11,12) R(12,11) R(12,13) R(12,14) R(13,2) R(13,12) R(14,12) R(14,15) R(15,14) R(15,16) R(16,4) R(16,15) R(n1,0) R(n1,n2) R(n2,8) R(n2,n1) R(n3,6) R(n3,9) R(n4,10) R(n4,n6) R(n5,7) R(n5,9) R(n6,9) R(n6,n4)

-------------------------------------------------------------------------------------------------------------

-> no other models
