# Supplementary material for: A complete logical approach to resolve the evolution and dynamics of mitochondrial genome in bilaterians
Source: PLoS One. 2018 Mar 16;13(3):e0194334. doi: 10.1371/journal.pone.0194334 (PMC5856267; doi:10.1371/journal.pone.0194334)
Supplement: S8 Appendix — (DOC) [file pone.0194334.s008.doc]

S8 Appendix. Axioms and solutions for Lophotrochozoa.

lophotrochozoans_taxA_9sol page 2

lophotrochozoans_taxB1_3sol page 8

lophotrochozoans_taxB2_3sol page 14

lophotrochozoans_taxB3_3sol page 20

lophotrochozoans_taxC1_9sol page 26

lophotrochozoans_taxC2_9sol page 36

lophotrochozoans_taxC3_9sol page 46

lophotrochozoans_taxD_6sol page 56

lophotrochozoans_taxE1_3sol page 61

lophotrochozoans_taxE2_3sol page 67

lophotrochozoans_taxF1_9sol page 73

lophotrochozoans_taxF2_9sol page 83

lophotrochozoans_taxA_9sol

================================================================================

================================================================================

AXIOMS

================================================================================

================================================================================

{ the solutions of problem PHYLO are the smallest graphs T (defined on the smallest domain possible but containing at least all the OTUs) which verify properties P1 to P6:

P1- T is simple (the relation R(x, y) which defines graph T is not reflexive)

P2- T is non-oriented (the relation R(x, y) which defines graph T is symetrical)

P3- T is connected and acyclic (T is a tree)

P4- T respects the minimal distance matrix, i.e.:

for all couple of OTUs x and y, the length of the path x->y in T is always superior or equals to the minimal distance calculated between x and y (encoded in the minimal distance matrix)

P5- T respects other eventual hypothesis (Primary Phylogenetic Hypothesis = PPH)

used to impose the existence of given monophyletic groups

P6- it is possible to calculate all the values for each HTU in the graph T }

{ OTUs: }

katharina_tunicata = 0;

nautilus_macromphallus = 1;

loligo_bleekeri = 2;

albinaria_soerulea = 3;

cepaea_nemoralis = 4;

biomphalaria_glabrata = 5;

limulus_polyphemus = 6; { = outgroup1 }

homo_sapiens = 7; { = outgroup2 }

{ PROPERTY P1: R(x, y) is not reflexive}

Q x (-R(x, x));

{ PROPERTY P2: R(x, y) is symetrical}

Q x y (R(x, y) => R(y, x));

{ PROPERTY P3: graph T is connected and acyclic (T is a tree) }

{

This property is verified by a constraint programmed in the model generator, instead of a "heavy" logical formula:

1- it will refuse the partial interpretations in which a connected component of the graph (in construction) is cyclic, i.e. such as: number of edges >= number of vertices

2- it will refuse the complete interpretations in which the constructed graph has more than one connected component

}

{ PROPERTY P4: graph T respects minimal distance matrix }

{

This property is verified by a constraint programmed in the model generator:

it will refuse the partial interpretations in which the graph (in construction) do not respect the minimal distance matrix, i.e. such as:

let x, y a couple of OTUs,

let d= minimal distance calculated between x and y (encoded in the minimal distance matrix), there is a path of length k between x and y, with: k < d

The minimal distance matrix is encoded directly in the data structure of the model generator:

/* minimal distance matrix lophotrochozoans taxA: */

DIST[0][0]=0;

DIST[1][0]=1; DIST[1][1]=0;

DIST[2][0]=2; DIST[2][1]=3; DIST[2][2]=0;

DIST[3][0]=7; DIST[3][1]=7; DIST[3][2]=7; DIST[3][3]=0;

DIST[4][0]=6; DIST[4][1]=6; DIST[4][2]=6; DIST[4][3]=1; DIST[4][4]=0;

DIST[5][0]=7; DIST[5][1]=7; DIST[5][2]=7; DIST[5][3]=1; DIST[5][4]=1; DIST[5][5]=0;

DIST[6][0]=2; DIST[6][1]=3; DIST[6][2]=2; DIST[6][3]=7; DIST[6][4]=6; DIST[6][5]=7; DIST[6][6]=0;

DIST[7][0]=3; DIST[7][1]=4; DIST[7][2]=3; DIST[7][3]=7; DIST[7][4]=6; DIST[7][5]=7; DIST[7][6]=2; DIST[7][7]=0;

}

{ PROPERTY P5: graph T respects eventual Primary Phylogenetic Hypotheses }

{

This property is verified by constraints programmed in the model generator:

- monophyly of Lophotrochozoa = (0,1,2,3,4,5)

- monophyly of Mollusca = (0,1,2,3,4,5)

- monophyly of Polyplacophora = (0)

- monophyly of Cephalopoda = (0,1,2)

- monophyly of Gastropoda = (0,3,4,5)

Notes:

1- katharina_tunicata mtDNA is identical to octopus_vulgaris mtDNA (mollusc *Cephalopoda*), considering only the 15 protein-coding genes and rRNA genes. Thus, katharina_tunicata must be also element of Cephalopoda clade in this computation.

2- katharina_tunicata mtDNA is also identical to haliotis_rubra mtDNA (mollusca Gastropoda). Thus, katharina_tunicata must be also element of Gastropoda clade in this computation.

}

{------------------------------------------------------------------------------------------------------------------------}

{ PROPERTY P6: it is possible to calculate all the values for each HTU in the graph T }

{

First we calculate with the model generator the set of tree solutions which verify properties P1 to P5. Property P6 is verified *a posteriori* for each tree solution, with a *feedback* mechanism:

Studying each tree solution for calculating the values of HTUs, we eventually discover "impossible sub-trees": they appear in tree solutions which verify P1 to P5, but they do not verify P6.

For each impossible subtree A, an additional constraint is programmed into the model generator to forbid the solutions containing A. Tree solutions are recalculated and verified, allowing the discovery of new impossible subtrees and the programming of new constraints to recalculate the solutions (feedback mechanism). Finally, the complete set of optimal solutions is determined after iteration of this process and elimination of all the solutions that do not verify P6.

}

================================================================================

================================================================================

SOLUTIONS

================================================================================

================================================================================

OTUs:

katharina_tunicata = 0;

nautilus_macromphallus = 1;

loligo_bleekeri = 2;

albinaria_soerulea = 3;

cepaea_nemoralis = 4;

biomphalaria_glabrata = 5;

limulus_polyphemus = 6; { = outgroup1 }

homo_sapiens = 7; { = outgroup2 }

HTUs:

n1, n2, n3, n4, n5, n6, n7, n8

D = [0,15]: 9 solutions OK (which verify property P6) (0 impossible sub-trees)

minimal score (best) = 30

maximal score = 40

-------------------------------------------------------------------------------------------------------------

-> form outgroup SOL1

n6(mod1)

[ cox1 cox2 atp8 atp6 cox3 nad3 -nad5 -nad4 -nad4L nad6 cob rrnS rrnL nad1 nad2 ]

score = 40:

model 1:

-------------

R(0,1) R(0,n1) R(0,n2) R(0,n3) R(1,0) R(2,n3) R(3,4) R(3,5) R(4,3) R(4,n4) R(5,3) R(6,n6) R(7,n6) R(n1,0) R(n1,n6) R(n2,0) R(n2,n8) R(n3,0) R(n3,2) R(n4,4) R(n4,n5) R(n5,n4) R(n5,n7) R(n6,6) R(n6,7) R(n6,n1) R(n7,n5) R(n7,n8) R(n8,n2) R(n8,n7)

-------------------------------------------------------------------------------------------------------------

-> form outgroup SOL2

score = 40:

model 2:

-------------

R(0,1) R(0,n1) R(0,n2) R(0,n3) R(1,0) R(2,n1) R(3,4) R(3,5) R(4,3) R(4,n4) R(5,3) R(6,n3) R(6,n6) R(7,n6) R(n1,0) R(n1,2) R(n2,0) R(n2,n8) R(n3,0) R(n3,6) R(n4,4) R(n4,n5) R(n5,n4) R(n5,n7) R(n6,6) R(n6,7) R(n7,n5) R(n7,n8) R(n8,n2) R(n8,n7)

-------------------------------------------------------------------------------------------------------------

-> form outgroup SOL3

n1(mod3)

[ cox1 cox2 atp8 atp6 cox3 nad3 -nad5 -nad4 -nad4L -cob -nad6 -nad1 -rrnL -rrnS nad2 ]

score = 35:

model 3:

-------------

R(0,1) R(0,n1) R(0,n2) R(0,n3) R(1,0) R(2,n3) R(3,4) R(3,5) R(4,3) R(4,n4) R(5,3) R(6,n1) R(7,n6) R(n1,0) R(n1,6) R(n1,n6) R(n2,0) R(n2,n8) R(n3,0) R(n3,2) R(n4,4) R(n4,n5) R(n5,n4) R(n5,n7) R(n6,7) R(n6,n1) R(n7,n5) R(n7,n8) R(n8,n2) R(n8,n7)

-------------------------------------------------------------------------------------------------------------

-> form outgroup SOL1

n6(mod4)

[ cox1 cox2 atp8 atp6 cox3 nad3 -nad5 -nad4 -nad4L nad6 cob rrnS rrnL nad1 nad2 ]

score = 35:

model 4:

-------------

R(0,1) R(0,n1) R(0,n2) R(0,n3) R(1,0) R(2,n3) R(3,4) R(4,3) R(4,5) R(4,n4) R(5,4) R(6,n6) R(7,n6) R(n1,0) R(n1,n6) R(n2,0) R(n2,n8) R(n3,0) R(n3,2) R(n4,4) R(n4,n5) R(n5,n4) R(n5,n7) R(n6,6) R(n6,7) R(n6,n1) R(n7,n5) R(n7,n8) R(n8,n2) R(n8,n7)

-------------------------------------------------------------------------------------------------------------

-> form outgroup SOL2

score = 35:

model 5:

-------------

R(0,1) R(0,n1) R(0,n2) R(0,n3) R(1,0) R(2,n1) R(3,4) R(4,3) R(4,5) R(4,n4) R(5,4) R(6,n3) R(6,n6) R(7,n6) R(n1,0) R(n1,2) R(n2,0) R(n2,n8) R(n3,0) R(n3,6) R(n4,4) R(n4,n5) R(n5,n4) R(n5,n7) R(n6,6) R(n6,7) R(n7,n5) R(n7,n8) R(n8,n2) R(n8,n7)

-------------------------------------------------------------------------------------------------------------

-> the best model

-> form outgroup SOL3

n1(mod6)

[ cox1 cox2 atp8 atp6 cox3 nad3 -nad5 -nad4 -nad4L -cob -nad6 -nad1 -rrnL -rrnS nad2 ]

score = 30:

model 6:

-------------

R(0,1) R(0,n1) R(0,n2) R(0,n3) R(1,0) R(2,n3) R(3,4) R(4,3) R(4,5) R(4,n4) R(5,4) R(6,n1) R(7,n6) R(n1,0) R(n1,6) R(n1,n6) R(n2,0) R(n2,n8) R(n3,0) R(n3,2) R(n4,4) R(n4,n5) R(n5,n4) R(n5,n7) R(n6,7) R(n6,n1) R(n7,n5) R(n7,n8) R(n8,n2) R(n8,n7)

-------------------------------------------------------------------------------------------------------------

-> form outgroup SOL1

n6(mod7)

[ cox1 cox2 atp8 atp6 cox3 nad3 -nad5 -nad4 -nad4L nad6 cob rrnS rrnL nad1 nad2 ]

score = 40:

model 7:

-------------

R(0,1) R(0,n1) R(0,n2) R(0,n3) R(1,0) R(2,n3) R(3,5) R(4,5) R(4,n4) R(5,3) R(5,4) R(6,n6) R(7,n6) R(n1,0) R(n1,n6) R(n2,0) R(n2,n8) R(n3,0) R(n3,2) R(n4,4) R(n4,n5) R(n5,n4) R(n5,n7) R(n6,6) R(n6,7) R(n6,n1) R(n7,n5) R(n7,n8) R(n8,n2) R(n8,n7)

-------------------------------------------------------------------------------------------------------------

-> form outgroup SOL2

score = 40:

model 8:

-------------

R(0,1) R(0,n1) R(0,n2) R(0,n3) R(1,0) R(2,n1) R(3,5) R(4,5) R(4,n4) R(5,3) R(5,4) R(6,n3) R(6,n6) R(7,n6) R(n1,0) R(n1,2) R(n2,0) R(n2,n8) R(n3,0) R(n3,6) R(n4,4) R(n4,n5) R(n5,n4) R(n5,n7) R(n6,6) R(n6,7) R(n7,n5) R(n7,n8) R(n8,n2) R(n8,n7)

-------------------------------------------------------------------------------------------------------------

-> form outgroup SOL3

n1(mod3)

[ cox1 cox2 atp8 atp6 cox3 nad3 -nad5 -nad4 -nad4L -cob -nad6 -nad1 -rrnL -rrnS nad2 ]

score = 35:

model 9:

-------------

R(0,1) R(0,n1) R(0,n2) R(0,n3) R(1,0) R(2,n3) R(3,5) R(4,5) R(4,n4) R(5,3) R(5,4) R(6,n1) R(7,n6) R(n1,0) R(n1,6) R(n1,n6) R(n2,0) R(n2,n8) R(n3,0) R(n3,2) R(n4,4) R(n4,n5) R(n5,n4) R(n5,n7) R(n6,7) R(n6,n1) R(n7,n5) R(n7,n8) R(n8,n2) R(n8,n7)

-------------------------------------------------------------------------------------------------------------

-> no other solutions

lophotrochozoans_taxB1_3sol

================================================================================

================================================================================

AXIOMS

================================================================================

================================================================================

{ the solutions of problem PHYLO are the smallest graphs T (defined on the smallest domain possible but containing at least all the OTUs) which verify properties P1 to P6:

P1- T is simple (the relation R(x, y) which defines graph T is not reflexive)

P2- T is non-oriented (the relation R(x, y) which defines graph T is symetrical)

P3- T is connected and acyclic (T is a tree)

P4- T respects the minimal distance matrix, i.e.:

for all couple of OTUs x and y, the length of the path x->y in T is always superior or equals to the minimal distance calculated between x and y (encoded in the minimal distance matrix)

P5- T respects other eventual hypothesis (Primary Phylogenetic Hypothesis = PPH)

used to impose the existence of given monophyletic groups

P6- it is possible to calculate all the values for each HTU in the graph T }

{ OTUs: }

katharina_tunicata = 0;

nautilus_macromphallus = 1;

loligo_bleekeri = 2;

platynereis_dumerilii = 3;

urechis_caupo = 4;

sipunculus_nudus = 5;

limulus_polyphemus = 6; { = outgroup1 }

homo_sapiens = 7; { = outgroup2 }

loxocorone_allax = 8;

terebratulina_retusa = 9;

phoronis_architecta = 10;

{ AUXILLIARY CONSTANTS used to fix a part of the solution: }

G1=11; { used to fix CASE 1 }

G2=12; { used to fix Cephalopoda group }

{ CASE 1 IS FIXED: 2 edges between homo_sapiens and limulus_polyphemus - first form }

R(homo_sapiens,G1);

Q x ( x<> G1

=>

-R(homo_sapiens,x)

);

R(limulus_polyphemus,G1);

Q x ( x<> G1

=>

-R(limulus_polyphemus,x)

);

{ THE CEPHALOPODA GROUP IS FIXED: }

R(nautilus_macromphallus,katharina_tunicata);

Q x ( x<> katharina_tunicata

=>

-R(nautilus_macromphallus,x)

);

R(G2,katharina_tunicata);

R(G2,loligo_bleekeri);

Q x ( ( x<> katharina_tunicata et

x<> loligo_bleekeri

)

=>

-R(G2,x)

);

Q x ( x<> G2

=>

-R(loligo_bleekeri,x)

);

{ PROPERTY P1: R(x, y) is not reflexive}

Q x (-R(x, x));

{ PROPERTY P2: R(x, y) is symetrical}

Q x y (R(x, y) => R(y, x));

{ PROPERTY P3: graph T is connected and acyclic (T is a tree) }

{

This property is verified by a constraint programmed in the model generator, instead of a "heavy" logical formula:

1- it will refuse the partial interpretations in which a connected component of the graph (in construction) is cyclic, i.e. such as: number of edges >= number of vertices

2- it will refuse the complete interpretations in which the constructed graph has more than one connected component

}

{ PROPERTY P4: graph T respects minimal distance matrix }

{

This property is verified by a constraint programmed in the model generator:

it will refuse the partial interpretations in which the graph (in construction) do not respect the minimal distance matrix, i.e. such as:

let x, y a couple of OTUs,

let d= minimal distance calculated between x and y (encoded in the minimal distance matrix), there is a path of length k between x and y, with: k < d

The minimal distance matrix is encoded directly in the data structure of the model generator:

/* minimal distance matrix lophotrochozoans taxB: */

DIST[0][0]=0;

DIST[1][0]=1; DIST[1][1]=0;

DIST[2][0]=2; DIST[2][1]=3; DIST[2][2]=0;

DIST[3][0]=3; DIST[3][1]=4; DIST[3][2]=4; DIST[3][3]=0;

DIST[4][0]=5; DIST[4][1]=5; DIST[4][2]=5; DIST[4][3]=3; DIST[4][4]=0;

DIST[5][0]=4; DIST[5][1]=4; DIST[5][2]=5; DIST[5][3]=3; DIST[5][4]=4; DIST[5][5]=0;

DIST[6][0]=2; DIST[6][1]=3; DIST[6][2]=2; DIST[6][3]=4; DIST[6][4]=5; DIST[6][5]=4; DIST[6][6]=0;

DIST[7][0]=3; DIST[7][1]=4; DIST[7][2]=3; DIST[7][3]=4; DIST[7][4]=5; DIST[7][5]=4; DIST[7][6]=2; DIST[7][7]=0;

DIST[8][0]=2; DIST[8][1]=2; DIST[8][2]=4; DIST[8][3]=5; DIST[8][4]=5; DIST[8][5]=5; DIST[8][6]=4; DIST[8][7]=4; DIST[8][8]=0;

DIST[9][0]=1; DIST[9][1]=2; DIST[9][2]=2; DIST[9][3]=3; DIST[9][4]=4; DIST[9][5]=5; DIST[9][6]=2; DIST[9][7]=3; DIST[9][8]=3; DIST[9][9]=0;

DIST[10][0]=1; DIST[10][1]=2; DIST[10][2]=3; DIST[10][3]=4; DIST[10][4]=5; DIST[10][5]=4; DIST[10][6]=3; DIST[10][7]=4; DIST[10][8]=3; DIST[10][9]=2; DIST[10][10]=0;

}

{ PROPERTY P5: graph T respects eventual Primary Phylogenetic Hypotheses }

{

This property is verified by constraints programmed in the model generator:

- monophyly of Lophotrochozoa = (0,1,2,3,4,5,8,9,10)

- monophyly of Eutrochozoa = (0,1,2,3,4,5,8)

- monophyly of Mollusca = (0,1,2)

- monophyly of Polyplacophora = (0)

- monophyly of Cephalopoda = (0,1,2)

- monophyly of Lophophorata = (9,10)

- monophyly of Annelida = (3,4)

- monophyly of Echiura = (3,4)

- monophyly of Polycheta = (3)

Notes:

1- katharina_tunicata mtDNA is identical to octopus_vulgaris mtDNA (mollusc *Cephalopoda*), considering only the 15 protein-coding genes and rRNA genes. Thus, katharina_tunicata must be also element of Cephalopoda clade in this computation.

2- platynereis_dumerilii mtDNA is identical to clymenella_torquata mtDNA (annelid *Echiura*). Thus, platynereis_dumerilii must be also element of *Echiura* clade in this computation.

}

{------------------------------------------------------------------------------------------------------------------------}

{ PROPERTY P6: it is possible to calculate all the values for each HTU in the graph T }

{

First we calculate with the model generator the set of tree solutions which verify properties P1 to P5. Property P6 is verified *a posteriori* for each tree solution, with a *feedback* mechanism:

Studying each tree solution for calculating the values of HTUs, we eventually discover "impossible sub-trees": they appear in tree solutions which verify P1 to P5, but they do not verify P6.

For each impossible subtree A, an additional constraint is programmed into the model generator to forbid the solutions containing A. Tree solutions are recalculated and verified, allowing the discovery of new impossible subtrees and the programming of new constraints to recalculate the solutions (feedback mechanism). Finally, the complete set of optimal solutions is determined after iteration of this process and elimination of all the solutions that do not verify P6.

}

================================================================================

================================================================================

SOLUTIONS

================================================================================

================================================================================

OTUs:

katharina_tunicata = 0;

nautilus_macromphallus = 1;

loligo_bleekeri = 2;

platynereis_dumerilii = 3;

urechis_caupo = 4;

sipunculus_nudus = 5;

limulus_polyphemus = 6; { = outgroup1 }

homo_sapiens = 7; { = outgroup2 }

loxocorone_allax = 8;

terebratulina_retusa = 9;

phoronis_architecta = 10;

AUXILLIARY CONSTANTS used to fix a part of the solution:

G1=11; { between homo_sapiens and limulus_polyphemus }

G2=12; { between katharina_tunicata and loligo_bleekeri }

HTUs:

n1, n2, n3, n4, n5, n6, n7, n8

D = [0,20]: 3 solutions OK (which verify property P6) (146 impossible sub-trees)

minimal score (best) = 65

maximal score = 79

-------------------------------------------------------------------------------------------------------------

-> form outgroup SOL1

G1(mod1)

[ cox1 cox2 atp8 atp6 cox3 nad3 -nad5 -nad4 -nad4L nad6 cob rrnS rrnL nad1 nad2 ]

score = 71:

model 1:

-------------

R(0,1) R(0,9) R(0,10) R(0,12) R(0,n1) R(0,n2) R(0,n3) R(1,0) R(2,12) R(3,n4) R(3,n5) R(3,n6) R(4,n7) R(5,n8) R(6,11) R(7,11) R(8,n3) R(9,0) R(10,0) R(11,6) R(11,7) R(11,n1) R(12,0) R(12,2) R(n1,0) R(n1,11) R(n2,0) R(n2,n6) R(n3,0) R(n3,8) R(n4,3) R(n4,n7) R(n5,3) R(n5,n8) R(n6,3) R(n6,n2) R(n7,4) R(n7,n4) R(n8,5) R(n8,n5)

-------------------------------------------------------------------------------------------------------------

-> form outgroup SOL1

-> the BEST model

G1(mod2)

[ cox1 cox2 atp8 atp6 cox3 nad3 -nad5 -nad4 -nad4L nad6 cob rrnS rrnL nad1 nad2 ]

n5(mod2)

g1[ cox1 cox2 atp8 atp6 nad5 cox3 nad6 cob nad4L nad4 rrnS rrnL nad1 nad3 nad2 ]

g2[ cox1 cox2 atp8 atp6 nad5 nad4L nad4 -cob -nad6 -cox3 rrnS rrnL nad1 nad3 nad2 ]

g3[ cox1 cox2 atp8 cox3 nad6 cob atp6 -nad5 -nad4 -nad4L rrnS rrnL nad1 nad3 nad2 ]

score = 65:

model 2:

-------------

R(0,1) R(0,9) R(0,10) R(0,12) R(0,n1) R(0,n2) R(0,n3) R(1,0) R(2,12) R(3,n4) R(3,n5) R(4,n6) R(5,n7) R(6,11) R(7,11) R(8,n1) R(9,0) R(10,0) R(11,6) R(11,7) R(11,n3) R(12,0) R(12,2) R(n1,0) R(n1,8) R(n2,0) R(n2,n5) R(n3,0) R(n3,11) R(n4,3) R(n4,n6) R(n5,3) R(n5,n2) R(n5,n8) R(n6,4) R(n6,n4) R(n7,5) R(n7,n8) R(n8,n5) R(n8,n7)

-------------------------------------------------------------------------------------------------------------

-> form outgroup SOL1

G1(mod3)

[ cox1 cox2 atp8 atp6 cox3 nad3 -nad5 -nad4 -nad4L nad6 cob rrnS rrnL nad1 nad2 ]

n5(mod3)

g1: [ cox1 cox2 atp8 cox3 nad6 cob nad4L nad4 atp6 nad5 rrnS rrnL nad1 nad3 nad2 ]

g2: [ cox1 cox2 atp8 cox3 nad6 cob nad4L nad4 rrnS rrnL nad1 atp6 nad5 nad3 nad2 ]

g3: [ cox1 cox2 atp8 cox3 nad6 cob nad4L nad4 rrnS rrnL nad1 nad3 atp6 nad5 nad2 ]

g4: [ cox1 cox2 atp8 cox3 nad6 cob rrnS rrnL nad1 atp6 nad5 nad4L nad4 nad3 nad2 ]

g5: [ cox1 cox2 atp8 cox3 nad6 cob rrnS rrnL nad1 -nad4 -nad4L -nad5 -atp6 nad3 nad2 ]

g6: [ cox1 cox2 atp8 cox3 nad6 cob atp6 nad5 -rrnL -rrnS nad4L nad4 nad1 nad3 nad2 ]

g7: [ cox1 cox2 atp8 cox3 nad6 cob -nad5 -atp6 -nad4 -nad4L rrnS rrnL nad1 nad3 nad2 ]

g8: [ cox1 cox2 atp8 cox3 nad6 cob -nad5 -atp6 -nad1 -rrnL -rrnS -nad4 -nad4L nad3 nad2 ]

g9: [ cox1 cox2 atp8 cox3 nad6 cob -nad4 -nad4L -nad5 -atp6 -nad1 -rrnL -rrnS nad3 nad2 ]

score = 79:

model 3:

-------------

R(0,1) R(0,9) R(0,10) R(0,12) R(0,n1) R(0,n2) R(0,n3) R(1,0) R(2,12) R(3,n4) R(3,n5) R(4,n6) R(5,n7) R(6,11) R(7,11) R(8,n1) R(9,0) R(10,0) R(11,6) R(11,7) R(11,n3) R(12,0) R(12,2) R(n1,0) R(n1,8) R(n2,0) R(n2,n8) R(n3,0) R(n3,11) R(n4,3) R(n4,n6) R(n5,3) R(n5,n7) R(n5,n8) R(n6,4) R(n6,n4) R(n7,5) R(n7,n5) R(n8,n2) R(n8,n5)

-------------------------------------------------------------------------------------------------------------

-> no other solutions

lophotrochozoans_taxB2_3sol

================================================================================

================================================================================

AXIOMS

================================================================================

================================================================================

{ the solutions of problem PHYLO are the smallest graphs T (defined on the smallest domain possible but containing at least all the OTUs) which verify properties P1 to P6:

P1- T is simple (the relation R(x, y) which defines graph T is not reflexive)

P2- T is non-oriented (the relation R(x, y) which defines graph T is symetrical)

P3- T is connected and acyclic (T is a tree)

P4- T respects the minimal distance matrix, i.e.:

for all couple of OTUs x and y, the length of the path x->y in T is always superior or equals to the minimal distance calculated between x and y (encoded in the minimal distance matrix)

P5- T respects other eventual hypothesis (Primary Phylogenetic Hypothesis = PPH)

used to impose the existence of given monophyletic groups

P6- it is possible to calculate all the values for each HTU in the graph T }

{ OTUs: }

katharina_tunicata = 0;

nautilus_macromphallus = 1;

loligo_bleekeri = 2;

platynereis_dumerilii = 3;

urechis_caupo = 4;

sipunculus_nudus = 5;

limulus_polyphemus = 6; { = outgroup1 }

homo_sapiens = 7; { = outgroup2 }

loxocorone_allax = 8;

terebratulina_retusa = 9;

phoronis_architecta = 10;

{ AUXILLIARY CONSTANTS used to fix a part of the solution: }

G1=11; { used to fix CASE 2 }

G2=12; { used to fix Cephalopoda group }

{ CASE 2 IS FIXED: 2 edges between homo_sapiens and limulus_polyphemus - second form }

R(homo_sapiens,G1);

Q x ( x<> G1

=>

-R(homo_sapiens,x)

);

R(G1,limulus_polyphemus);

Q x ( ( x<>limulus_polyphemus et

x<>homo_sapiens

)

=>

-R(G1,x)

);

{ THE CEPHALOPODA GROUP IS FIXED: }

R(nautilus_macromphallus,katharina_tunicata);

Q x ( x<> katharina_tunicata

=>

-R(nautilus_macromphallus,x)

);

R(G2,katharina_tunicata);

R(G2,loligo_bleekeri);

Q x ( ( x<> katharina_tunicata et

x<> loligo_bleekeri

)

=>

-R(G2,x)

);

Q x ( x<> G2

=>

-R(loligo_bleekeri,x)

);

{ PROPERTY P1: R(x, y) is not reflexive}

Q x (-R(x, x));

{ PROPERTY P2: R(x, y) is symetrical}

Q x y (R(x, y) => R(y, x));

{ PROPERTY P3: graph T is connected and acyclic (T is a tree) }

{

This property is verified by a constraint programmed in the model generator, instead of a "heavy" logical formula:

1- it will refuse the partial interpretations in which a connected component of the graph (in construction) is cyclic, i.e. such as: number of edges >= number of vertices

2- it will refuse the complete interpretations in which the constructed graph has more than one connected component

}

{ PROPERTY P4: graph T respects minimal distance matrix }

{

This property is verified by a constraint programmed in the model generator:

it will refuse the partial interpretations in which the graph (in construction) do not respect the minimal distance matrix, i.e. such as:

let x, y a couple of OTUs,

let d= minimal distance calculated between x and y (encoded in the minimal distance matrix), there is a path of length k between x and y, with: k < d

The minimal distance matrix is encoded directly in the data structure of the model generator:

/* minimal distance matrix lophotrochozoans taxB: */

DIST[0][0]=0;

DIST[1][0]=1; DIST[1][1]=0;

DIST[2][0]=2; DIST[2][1]=3; DIST[2][2]=0;

DIST[3][0]=3; DIST[3][1]=4; DIST[3][2]=4; DIST[3][3]=0;

DIST[4][0]=5; DIST[4][1]=5; DIST[4][2]=5; DIST[4][3]=3; DIST[4][4]=0;

DIST[5][0]=4; DIST[5][1]=4; DIST[5][2]=5; DIST[5][3]=3; DIST[5][4]=4; DIST[5][5]=0;

DIST[6][0]=2; DIST[6][1]=3; DIST[6][2]=2; DIST[6][3]=4; DIST[6][4]=5; DIST[6][5]=4; DIST[6][6]=0;

DIST[7][0]=3; DIST[7][1]=4; DIST[7][2]=3; DIST[7][3]=4; DIST[7][4]=5; DIST[7][5]=4; DIST[7][6]=2; DIST[7][7]=0;

DIST[8][0]=2; DIST[8][1]=2; DIST[8][2]=4; DIST[8][3]=5; DIST[8][4]=5; DIST[8][5]=5; DIST[8][6]=4; DIST[8][7]=4; DIST[8][8]=0;

DIST[9][0]=1; DIST[9][1]=2; DIST[9][2]=2; DIST[9][3]=3; DIST[9][4]=4; DIST[9][5]=5; DIST[9][6]=2; DIST[9][7]=3; DIST[9][8]=3; DIST[9][9]=0;

DIST[10][0]=1; DIST[10][1]=2; DIST[10][2]=3; DIST[10][3]=4; DIST[10][4]=5; DIST[10][5]=4; DIST[10][6]=3; DIST[10][7]=4; DIST[10][8]=3; DIST[10][9]=2; DIST[10][10]=0;

}

{ PROPERTY P5: graph T respects eventual Primary Phylogenetic Hypotheses }

{

This property is verified by constraints programmed in the model generator:

- monophyly of Lophotrochozoa = (0,1,2,3,4,5,8,9,10)

- monophyly of Eutrochozoa = (0,1,2,3,4,5,8)

- monophyly of Mollusca = (0,1,2)

- monophyly of Polyplacophora = (0)

- monophyly of Cephalopoda = (0,1,2)

- monophyly of Lophophorata = (9,10)

- monophyly of Annelida = (3,4)

- monophyly of Echiura = (3,4)

- monophyly of Polycheta = (3)

Notes:

1- katharina_tunicata mtDNA is identical to octopus_vulgaris mtDNA (mollusc *Cephalopoda*), considering only the 15 protein-coding genes and rRNA genes. Thus, katharina_tunicata must be also element of Cephalopoda clade in this computation.

2- platynereis_dumerilii mtDNA is identical to clymenella_torquata mtDNA (annelid *Echiura*). Thus, platynereis_dumerilii must be also element of *Echiura* clade in this computation.

}

{------------------------------------------------------------------------------------------------------------------------}

{ PROPERTY P6: it is possible to calculate all the values for each HTU in the graph T }

{

First we calculate with the model generator the set of tree solutions which verify properties P1 to P5. Property P6 is verified *a posteriori* for each tree solution, with a *feedback* mechanism:

Studying each tree solution for calculating the values of HTUs, we eventually discover "impossible sub-trees": they appear in tree solutions which verify P1 to P5, but they do not verify P6.

For each impossible subtree A, an additional constraint is programmed into the model generator to forbid the solutions containing A. Tree solutions are recalculated and verified, allowing the discovery of new impossible subtrees and the programming of new constraints to recalculate the solutions (feedback mechanism). Finally, the complete set of optimal solutions is determined after iteration of this process and elimination of all the solutions that do not verify P6.

}

================================================================================

================================================================================

SOLUTIONS

================================================================================

================================================================================

OTUs:

katharina_tunicata = 0;

nautilus_macromphallus = 1;

loligo_bleekeri = 2;

platynereis_dumerilii = 3;

urechis_caupo = 4;

sipunculus_nudus = 5;

limulus_polyphemus = 6; { = outgroup1 }

homo_sapiens = 7; { = outgroup2 }

loxocorone_allax = 8;

terebratulina_retusa = 9;

phoronis_architecta = 10;

AUXILLIARY CONSTANTS used to fix a part of the solution:

G1=11; { between homo_sapiens and limulus_polyphemus }

G2=12; { between katharina_tunicata and loligo_bleekeri }

HTUs:

n1, n2, n3, n4, n5, n6, n7, n8

D = [0,20]: 3 solutions OK (which verify property P6) (146 impossible sub-trees)

minimal score (best) = 65

maximal score = 79

-------------------------------------------------------------------------------------------------------------

-> form outgroup SOL2

score = 71:

model 1:

-------------

R(0,1) R(0,9) R(0,10) R(0,12) R(0,n1) R(0,n2) R(0,n3) R(1,0) R(2,12) R(3,n4) R(3,n5) R(3,n6) R(4,n7) R(5,n8) R(6,11) R(6,n1) R(7,11) R(8,n2) R(9,0) R(10,0) R(11,6) R(11,7) R(12,0) R(12,2) R(n1,0) R(n1,6) R(n2,0) R(n2,8) R(n3,0) R(n3,n6) R(n4,3) R(n4,n8) R(n5,3) R(n5,n7) R(n6,3) R(n6,n3) R(n7,4) R(n7,n5) R(n8,5) R(n8,n4)

-------------------------------------------------------------------------------------------------------------

-> form outgroup SOL2

-> the best model

n5(mod2)

g1[ cox1 cox2 atp8 atp6 nad5 cox3 nad6 cob nad4L nad4 rrnS rrnL nad1 nad3 nad2 ]

g2[ cox1 cox2 atp8 atp6 nad5 nad4L nad4 -cob -nad6 -cox3 rrnS rrnL nad1 nad3 nad2 ]

g3[ cox1 cox2 atp8 cox3 nad6 cob atp6 -nad5 -nad4 -nad4L rrnS rrnL nad1 nad3 nad2 ]

score = 65:

model 2:

-------------

R(0,1) R(0,9) R(0,10) R(0,12) R(0,n1) R(0,n2) R(0,n3) R(1,0) R(2,12) R(3,n4) R(3,n5) R(4,n6) R(5,n7) R(6,11) R(6,n1) R(7,11) R(8,n3) R(9,0) R(10,0) R(11,6) R(11,7) R(12,0) R(12,2) R(n1,0) R(n1,6) R(n2,0) R(n2,n5) R(n3,0) R(n3,8) R(n4,3) R(n4,n6) R(n5,3) R(n5,n2) R(n5,n8) R(n6,4) R(n6,n4) R(n7,5) R(n7,n8) R(n8,n5) R(n8,n7)

-------------------------------------------------------------------------------------------------------------

-> form outgroup SOL2

n5(mod3)

g1: [ cox1 cox2 atp8 cox3 nad6 cob nad4L nad4 atp6 nad5 rrnS rrnL nad1 nad3 nad2 ]

g2: [ cox1 cox2 atp8 cox3 nad6 cob nad4L nad4 rrnS rrnL nad1 atp6 nad5 nad3 nad2 ]

g3: [ cox1 cox2 atp8 cox3 nad6 cob nad4L nad4 rrnS rrnL nad1 nad3 atp6 nad5 nad2 ]

g4: [ cox1 cox2 atp8 cox3 nad6 cob rrnS rrnL nad1 atp6 nad5 nad4L nad4 nad3 nad2 ]

g5: [ cox1 cox2 atp8 cox3 nad6 cob rrnS rrnL nad1 -nad4 -nad4L -nad5 -atp6 nad3 nad2 ]

g6: [ cox1 cox2 atp8 cox3 nad6 cob atp6 nad5 -rrnL -rrnS nad4L nad4 nad1 nad3 nad2 ]

g7: [ cox1 cox2 atp8 cox3 nad6 cob -nad5 -atp6 -nad4 -nad4L rrnS rrnL nad1 nad3 nad2 ]

g8: [ cox1 cox2 atp8 cox3 nad6 cob -nad5 -atp6 -nad1 -rrnL -rrnS -nad4 -nad4L nad3 nad2 ]

g9: [ cox1 cox2 atp8 cox3 nad6 cob -nad4 -nad4L -nad5 -atp6 -nad1 -rrnL -rrnS nad3 nad2 ]

score = 79:

model 3:

-------------

R(0,1) R(0,9) R(0,10) R(0,12) R(0,n1) R(0,n2) R(0,n3) R(1,0) R(2,12) R(3,n4) R(3,n5) R(4,n6) R(5,n7) R(6,11) R(6,n1) R(7,11) R(8,n3) R(9,0) R(10,0) R(11,6) R(11,7) R(12,0) R(12,2) R(n1,0) R(n1,6) R(n2,0) R(n2,n8) R(n3,0) R(n3,8) R(n4,3) R(n4,n6) R(n5,3) R(n5,n7) R(n5,n8) R(n6,4) R(n6,n4) R(n7,5) R(n7,n5) R(n8,n2) R(n8,n5)

-------------------------------------------------------------------------------------------------------------

-> no other solutions

lophotrochozoans_taxB3_3sol

================================================================================

================================================================================

AXIOMS

================================================================================

================================================================================

{ the solutions of problem PHYLO are the smallest graphs T (defined on the smallest domain possible but containing at least all the OTUs) which verify properties P1 to P6:

P1- T is simple (the relation R(x, y) which defines graph T is not reflexive)

P2- T is non-oriented (the relation R(x, y) which defines graph T is symetrical)

P3- T is connected and acyclic (T is a tree)

P4- T respects the minimal distance matrix, i.e.:

for all couple of OTUs x and y, the length of the path x->y in T is always superior or equals to the minimal distance calculated between x and y (encoded in the minimal distance matrix)

P5- T respects other eventual hypothesis (Primary Phylogenetic Hypothesis = PPH)

used to impose the existence of given monophyletic groups

P6- it is possible to calculate all the values for each HTU in the graph T }

{ OTUs: }

katharina_tunicata = 0;

nautilus_macromphallus = 1;

loligo_bleekeri = 2;

platynereis_dumerilii = 3;

urechis_caupo = 4;

sipunculus_nudus = 5;

limulus_polyphemus = 6; { = outgroup1 }

homo_sapiens = 7; { = outgroup2 }

loxocorone_allax = 8;

terebratulina_retusa = 9;

phoronis_architecta = 10;

{ AUXILLIARY CONSTANTS used to fix a part of the solution: }

G1=11; G2=12; { used to fix CASE 3 }

G3=13; { used to fix Cephalopoda group }

{ CASE 3 IS FIXED: 3 edges between homo_sapiens and limulus_polyphemus - first form }

R(homo_sapiens,G1);

Q x ( x <> G1

=>

-R(homo_sapiens,x)

);

R(G1,G2);

Q x ( ( x <> G2 et

x <> homo_sapiens

)

=>

-R(G1,x)

);

R(limulus_polyphemus,G2);

Q x ( x <> G2

=>

-R(limulus_polyphemus,x)

);

{ THE CEPHALOPODA GROUP IS FIXED: }

R(nautilus_macromphallus,katharina_tunicata);

Q x ( x <> katharina_tunicata

=>

-R(nautilus_macromphallus,x)

);

R(G3,katharina_tunicata);

R(G3,loligo_bleekeri);

Q x ( ( x <> katharina_tunicata et

x <> loligo_bleekeri

)

=>

-R(G3,x)

);

Q x ( x <> G3

=>

-R(loligo_bleekeri,x)

);

{ PROPERTY P1: R(x, y) is not reflexive}

Q x (-R(x, x));

{ PROPERTY P2: R(x, y) is symetrical}

Q x y (R(x, y) => R(y, x));

{ PROPERTY P3: graph T is connected and acyclic (T is a tree) }

{

This property is verified by a constraint programmed in the model generator, instead of a "heavy" logical formula:

1- it will refuse the partial interpretations in which a connected component of the graph (in construction) is cyclic, i.e. such as: number of edges >= number of vertices

2- it will refuse the complete interpretations in which the constructed graph has more than one connected component

}

{ PROPERTY P4: graph T respects minimal distance matrix }

{

This property is verified by a constraint programmed in the model generator:

it will refuse the partial interpretations in which the graph (in construction) do not respect the minimal distance matrix, i.e. such as:

let x, y a couple of OTUs,

let d= minimal distance calculated between x and y (encoded in the minimal distance matrix), there is a path of length k between x and y, with: k < d

The minimal distance matrix is encoded directly in the data structure of the model generator:

/* minimal distance matrix lophotrochozoans taxB: */

DIST[0][0]=0;

DIST[1][0]=1; DIST[1][1]=0;

DIST[2][0]=2; DIST[2][1]=3; DIST[2][2]=0;

DIST[3][0]=3; DIST[3][1]=4; DIST[3][2]=4; DIST[3][3]=0;

DIST[4][0]=5; DIST[4][1]=5; DIST[4][2]=5; DIST[4][3]=3; DIST[4][4]=0;

DIST[5][0]=4; DIST[5][1]=4; DIST[5][2]=5; DIST[5][3]=3; DIST[5][4]=4; DIST[5][5]=0;

DIST[6][0]=2; DIST[6][1]=3; DIST[6][2]=2; DIST[6][3]=4; DIST[6][4]=5; DIST[6][5]=4; DIST[6][6]=0;

DIST[7][0]=3; DIST[7][1]=4; DIST[7][2]=3; DIST[7][3]=4; DIST[7][4]=5; DIST[7][5]=4; DIST[7][6]=2; DIST[7][7]=0;

DIST[8][0]=2; DIST[8][1]=2; DIST[8][2]=4; DIST[8][3]=5; DIST[8][4]=5; DIST[8][5]=5; DIST[8][6]=4; DIST[8][7]=4; DIST[8][8]=0;

DIST[9][0]=1; DIST[9][1]=2; DIST[9][2]=2; DIST[9][3]=3; DIST[9][4]=4; DIST[9][5]=5; DIST[9][6]=2; DIST[9][7]=3; DIST[9][8]=3; DIST[9][9]=0;

DIST[10][0]=1; DIST[10][1]=2; DIST[10][2]=3; DIST[10][3]=4; DIST[10][4]=5; DIST[10][5]=4; DIST[10][6]=3; DIST[10][7]=4; DIST[10][8]=3; DIST[10][9]=2; DIST[10][10]=0;

}

{ PROPERTY P5: graph T respects eventual Primary Phylogenetic Hypotheses }

{

This property is verified by constraints programmed in the model generator:

- monophyly of Lophotrochozoa = (0,1,2,3,4,5,8,9,10)

- monophyly of Eutrochozoa = (0,1,2,3,4,5,8)

- monophyly of Mollusca = (0,1,2)

- monophyly of Polyplacophora = (0)

- monophyly of Cephalopoda = (0,1,2)

- monophyly of Lophophorata = (9,10)

- monophyly of Annelida = (3,4)

- monophyly of Echiura = (3,4)

- monophyly of Polycheta = (3)

Notes:

1- katharina_tunicata mtDNA is identical to octopus_vulgaris mtDNA (mollusc *Cephalopoda*), considering only the 15 protein-coding genes and rRNA genes. Thus, katharina_tunicata must be also element of Cephalopoda clade in this computation.

2- platynereis_dumerilii mtDNA is identical to clymenella_torquata mtDNA (annelid *Echiura*). Thus, platynereis_dumerilii must be also element of *Echiura* clade in this computation.

}

{------------------------------------------------------------------------------------------------------------------------}

{ PROPERTY P6: it is possible to calculate all the values for each HTU in the graph T }

{

First we calculate with the model generator the set of tree solutions which verify properties P1 to P5. Property P6 is verified *a posteriori* for each tree solution, with a *feedback* mechanism:

Studying each tree solution for calculating the values of HTUs, we eventually discover "impossible sub-trees": they appear in tree solutions which verify P1 to P5, but they do not verify P6.

For each impossible subtree A, an additional constraint is programmed into the model generator to forbid the solutions containing A. Tree solutions are recalculated and verified, allowing the discovery of new impossible subtrees and the programming of new constraints to recalculate the solutions (feedback mechanism). Finally, the complete set of optimal solutions is determined after iteration of this process and elimination of all the solutions that do not verify P6.

}

================================================================================

================================================================================

SOLUTIONS

================================================================================

================================================================================

OTUs:

katharina_tunicata = 0;

nautilus_macromphallus = 1;

loligo_bleekeri = 2;

platynereis_dumerilii = 3;

urechis_caupo = 4;

sipunculus_nudus = 5;

limulus_polyphemus = 6; { = outgroup1 }

homo_sapiens = 7; { = outgroup2 }

loxocorone_allax = 8;

terebratulina_retusa = 9;

phoronis_architecta = 10;

AUXILLIARY CONSTANTS used to fix a part of the solution:

G1=11; { between homo_sapiens and limulus_polyphemus }

G2=12; { between homo_sapiens and limulus_polyphemus }

G3=13; { between katharina_tunicata and loligo_bleekeri }

HTUs:

n1, n2, n3, n4, n5, n6, n7

D = [0,20]: 3 solutions OK (which verify property P6) (146 impossible sub-trees)

minimal score (best) = 57

maximal score = 71

-------------------------------------------------------------------------------------------------------------

-> form outgroup SOL3

G2(mod1)

[ cox1 cox2 atp8 atp6 cox3 nad3 -nad5 -nad4 -nad4L -cob -nad6 -nad1 -rrnL -rrnS nad2 ]

score = 63:

model 1:

-------------

R(0,1) R(0,9) R(0,10) R(0,12) R(0,13) R(0,n1) R(0,n2) R(1,0) R(2,13) R(3,n3) R(3,n4) R(3,n5) R(4,n6) R(5,n7) R(6,12) R(7,11) R(8,n2) R(9,0) R(10,0) R(11,7) R(11,12) R(12,0) R(12,6) R(12,11) R(13,0) R(13,2) R(n1,0) R(n1,n5) R(n2,0) R(n2,8) R(n3,3) R(n3,n6) R(n4,3) R(n4,n7) R(n5,3) R(n5,n1) R(n6,4) R(n6,n3) R(n7,5) R(n7,n4)

-------------------------------------------------------------------------------------------------------------

-> form outgroup SOL3

-> the best model

G2(mod2)

[ cox1 cox2 atp8 atp6 cox3 nad3 -nad5 -nad4 -nad4L -cob -nad6 -nad1 -rrnL -rrnS nad2 ]

n4(mod2)

g1[ cox1 cox2 atp8 atp6 nad5 cox3 nad6 cob nad4L nad4 rrnS rrnL nad1 nad3 nad2 ]

g2[ cox1 cox2 atp8 atp6 nad5 nad4L nad4 -cob -nad6 -cox3 rrnS rrnL nad1 nad3 nad2 ]

g3[ cox1 cox2 atp8 cox3 nad6 cob atp6 -nad5 -nad4 -nad4L rrnS rrnL nad1 nad3 nad2 ]

score = 57:

model 2:

-------------

R(0,1) R(0,9) R(0,10) R(0,12) R(0,13) R(0,n1) R(0,n2) R(1,0) R(2,13) R(3,n3) R(3,n4) R(4,n5) R(5,n6) R(6,12) R(7,11) R(8,n2) R(9,0) R(10,0) R(11,7) R(11,12) R(12,0) R(12,6) R(12,11) R(13,0) R(13,2) R(n1,0) R(n1,n4) R(n2,0) R(n2,8) R(n3,3) R(n3,n5) R(n4,3) R(n4,n1) R(n4,n7) R(n5,4) R(n5,n3) R(n6,5) R(n6,n7) R(n7,n4) R(n7,n6)

-------------------------------------------------------------------------------------------------------------

-> form outgroup SOL3

G2(mod3)

[ cox1 cox2 atp8 atp6 cox3 nad3 -nad5 -nad4 -nad4L -cob -nad6 -nad1 -rrnL -rrnS nad2 ]

n4(mod3)

g1: [ cox1 cox2 atp8 cox3 nad6 cob nad4L nad4 atp6 nad5 rrnS rrnL nad1 nad3 nad2 ]

g2: [ cox1 cox2 atp8 cox3 nad6 cob nad4L nad4 rrnS rrnL nad1 atp6 nad5 nad3 nad2 ]

g3: [ cox1 cox2 atp8 cox3 nad6 cob nad4L nad4 rrnS rrnL nad1 nad3 atp6 nad5 nad2 ]

g4: [ cox1 cox2 atp8 cox3 nad6 cob rrnS rrnL nad1 atp6 nad5 nad4L nad4 nad3 nad2 ]

g5: [ cox1 cox2 atp8 cox3 nad6 cob rrnS rrnL nad1 -nad4 -nad4L -nad5 -atp6 nad3 nad2 ]

g6: [ cox1 cox2 atp8 cox3 nad6 cob atp6 nad5 -rrnL -rrnS nad4L nad4 nad1 nad3 nad2 ]

g7: [ cox1 cox2 atp8 cox3 nad6 cob -nad5 -atp6 -nad4 -nad4L rrnS rrnL nad1 nad3 nad2 ]

g8: [ cox1 cox2 atp8 cox3 nad6 cob -nad5 -atp6 -nad1 -rrnL -rrnS -nad4 -nad4L nad3 nad2 ]

g9: [ cox1 cox2 atp8 cox3 nad6 cob -nad4 -nad4L -nad5 -atp6 -nad1 -rrnL -rrnS nad3 nad2 ]

score = 71:

model 3:

-------------

R(0,1) R(0,9) R(0,10) R(0,12) R(0,13) R(0,n1) R(0,n2) R(1,0) R(2,13) R(3,n3) R(3,n4) R(4,n5) R(5,n6) R(6,12) R(7,11) R(8,n2) R(9,0) R(10,0) R(11,7) R(11,12) R(12,0) R(12,6) R(12,11) R(13,0) R(13,2) R(n1,0) R(n1,n7) R(n2,0) R(n2,8) R(n3,3) R(n3,n5) R(n4,3) R(n4,n6) R(n4,n7) R(n5,4) R(n5,n3) R(n6,5) R(n6,n4) R(n7,n1) R(n7,n4)

-------------------------------------------------------------------------------------------------------------

-> no other solutions

lophotrochozoans_taxC1_9sol

================================================================================

================================================================================

AXIOMS

================================================================================

================================================================================

{ the solutions of problem PHYLO are the smallest graphs T (defined on the smallest domain possible but containing at least all the OTUs) which verify properties P1 to P6:

P1- T is simple (the relation R(x, y) which defines graph T is not reflexive)

P2- T is non-oriented (the relation R(x, y) which defines graph T is symetrical)

P3- T is connected and acyclic (T is a tree)

P4- T respects the minimal distance matrix, i.e.:

for all couple of OTUs x and y, the length of the path x->y in T is always superior or equals to the minimal distance calculated between x and y (encoded in the minimal distance matrix)

P5- T respects other eventual hypothesis (Primary Phylogenetic Hypothesis = PPH)

used to impose the existence of given monophyletic groups

P6- it is possible to calculate all the values for each HTU in the graph T }

{ OTUs: }

katharina_tunicata = 0;

nautilus_macromphallus = 1;

loligo_bleekeri = 2;

platynereis_dumerilii = 3;

urechis_caupo = 4;

sipunculus_nudus = 5;

limulus_polyphemus = 6; { = outgroup1 }

homo_sapiens = 7; { = outgroup2 }

loxocorone_allax = 8;

terebratulina_retusa = 9;

phoronis_architecta = 10;

bugula_neritina=11;

terebratalia_transversa=12;

{ AUXILLIARY CONSTANTS used to fix a part of the solution: }

G1=13; G2=14; G3=15;

G4=16; G5=17; G6=18;

G7=19; G8=20; { used to fix the Eutrochozoa group }

G9=21; { used to fix CASE 1 }

{ CASE 1 IS FIXED: 2 edges between homo_sapiens and limulus_polyphemus - first form }

R(homo_sapiens,G9);

Q x ( x<> G9

=>

-R(homo_sapiens,x)

);

R(limulus_polyphemus,G9);

Q x ( x<> G9

=>

-R(limulus_polyphemus,x)

);

{ THE EUTROCHOZOA GROUP IS FIXED: }

{ OTUs (0,1,2): }

R(katharina_tunicata,nautilus_macromphallus);

R(katharina_tunicata,G1);

R(G1,loligo_bleekeri);

Q x ( x<>katharina_tunicata

=>

-R(nautilus_macromphallus,x)

);

Q x ( ( x<>katharina_tunicata et

x<>loligo_bleekeri

)

=>

-R(G1,x)

);

Q x ( x<>G1

=>

-R(loligo_bleekeri,x)

);

{ OTUs (3,4,5,8): }

{ as in the best of the 3 possible forms }

R(katharina_tunicata,G2);

R(G2,G3);

R(G3,platynereis_dumerilii);

R(platynereis_dumerilii,G4);

R(G4,G5);

R(G5,urechis_caupo);

R(G3,G6);

R(G6,G7);

R(G7,sipunculus_nudus);

Q x ( ( x<>katharina_tunicata et

x<>G3

)

=>

-R(G2,x)

);

Q x ( ( x<>G2 et

x<>platynereis_dumerilii et

x<>G6

)

=>

-R(G3,x)

);

Q x ( ( x<>platynereis_dumerilii et

x<>G5

)

=>

-R(G4,x)

);

Q x ( ( x<>G3 et

x<>G4

)

=>

-R(platynereis_dumerilii,x)

);

Q x ( ( x<>G4 et

x<>urechis_caupo

)

=>

-R(G5,x)

);

Q x ( x<>G5

=>

-R(urechis_caupo,x)

);

Q x ( ( x<>G3 et

x<>G7

)

=>

-R(G6,x)

);

Q x ( ( x<>G6 et

x<>sipunculus_nudus

)

=>

-R(G7,x)

);

Q x ( x<>G7

=>

-R(sipunculus_nudus,x)

);

R(katharina_tunicata,G8);

R(G8,loxocorone_allax);

Q x ( ( x<>katharina_tunicata et

x<>loxocorone_allax

)

=>

-R(G8,x)

);

Q x ( x<>G8

=>

-R(loxocorone_allax, x)

);

{ PROPERTY P1: R(x, y) is not reflexive}

Q x (-R(x, x));

{ PROPERTY P2: R(x, y) is symetrical}

Q x y (R(x, y) => R(y, x));

{ PROPERTY P3: graph T is connected and acyclic (T is a tree) }

{

This property is verified by a constraint programmed in the model generator, instead of a "heavy" logical formula:

1- it will refuse the partial interpretations in which a connected component of the graph (in construction) is cyclic, i.e. such as: number of edges >= number of vertices

2- it will refuse the complete interpretations in which the constructed graph has more than one connected component

}

{ PROPERTY P4: graph T respects minimal distance matrix }

{

This property is verified by a constraint programmed in the model generator:

it will refuse the partial interpretations in which the graph (in construction) do not respect the minimal distance matrix, i.e. such as:

let x, y a couple of OTUs,

let d= minimal distance calculated between x and y (encoded in the minimal distance matrix), there is a path of length k between x and y, with: k < d

The minimal distance matrix is encoded directly in the data structure of the model generator:

/* minimal distance matrix lophotrochozoans taxC: */

DIST[0][0]=0;

DIST[1][0]=1; DIST[1][1]=0;

DIST[2][0]=2; DIST[2][1]=3; DIST[2][2]=0;

DIST[3][0]=3; DIST[3][1]=4; DIST[3][2]=4; DIST[3][3]=0;

DIST[4][0]=5; DIST[4][1]=5; DIST[4][2]=5; DIST[4][3]=3; DIST[4][4]=0;

DIST[5][0]=4; DIST[5][1]=4; DIST[5][2]=5; DIST[5][3]=3; DIST[5][4]=4; DIST[5][5]=0;

DIST[6][0]=2; DIST[6][1]=3; DIST[6][2]=2; DIST[6][3]=4; DIST[6][4]=5; DIST[6][5]=4; DIST[6][6]=0;

DIST[7][0]=3; DIST[7][1]=4; DIST[7][2]=3; DIST[7][3]=4; DIST[7][4]=5; DIST[7][5]=4; DIST[7][6]=2; DIST[7][7]=0;

DIST[8][0]=2; DIST[8][1]=2; DIST[8][2]=4; DIST[8][3]=5; DIST[8][4]=5; DIST[8][5]=5; DIST[8][6]=4; DIST[8][7]=4; DIST[8][8]=0;

DIST[9][0]=1; DIST[9][1]=2; DIST[9][2]=2; DIST[9][3]=3; DIST[9][4]=4; DIST[9][5]=5; DIST[9][6]=2; DIST[9][7]=3; DIST[9][8]=3; DIST[9][9]=0;

DIST[10][0]=1; DIST[10][1]=2; DIST[10][2]=3; DIST[10][3]=4; DIST[10][4]=5; DIST[10][5]=4; DIST[10][6]=3; DIST[10][7]=4; DIST[10][8]=3; DIST[10][9]=2; DIST[10][10]=0;

DIST[11][0]=4; DIST[11][1]=5; DIST[11][2]=5; DIST[11][3]=6; DIST[11][4]=6; DIST[11][5]=6; DIST[11][6]=5; DIST[11][7]=5; DIST[11][8]=5; DIST[11][9]=4; DIST[11][10]=4; DIST[11][11]=0;

DIST[12][0]=5; DIST[12][1]=6; DIST[12][2]=6; DIST[12][3]=5; DIST[12][4]=7; DIST[12][5]=6; DIST[12][6]=6; DIST[12][7]=7; DIST[12][8]=7; DIST[12][9]=5; DIST[12][10]=5; DIST[12][11]=7; DIST[12][12]=0;

}

{ PROPERTY P5: graph T respects eventual Primary Phylogenetic Hypotheses }

{

This property is verified by constraints programmed in the model generator:

- monophyly of Lophotrochozoa = (0,1,2,3,4,5,8,9,10,11,12)

- monophyly of Eutrochozoa = (0,1,2,3,4,5,8)

- monophyly of Mollusca = (0,1,2)

- monophyly of Polyplacophora = (0)

- monophyly of Cephalopoda = (0,1,2)

- monophyly of Annelida = (3,4)

- monophyly of Echiura = (3,4)

- monophyly of Polycheta = (3)

- monophyly of Lophophorata = (9,10,11,12)

- monophyly of Brachiopoda = (9,12)

Notes:

1- katharina_tunicata mtDNA is identical to octopus_vulgaris mtDNA (mollusc *Cephalopoda*), considering only the 15 protein-coding genes and rRNA genes. Thus, katharina_tunicata must be also element of Cephalopoda clade in this computation.

2- platynereis_dumerilii mtDNA is identical to clymenella_torquata mtDNA (annelid *Echiura*). Thus, platynereis_dumerilii must be also element of *Echiura* clade in this computation.

}

{------------------------------------------------------------------------------------------------------------------------}

{ PROPERTY P6: it is possible to calculate all the values for each HTU in the graph T }

{

First we calculate with the model generator the set of tree solutions which verify properties P1 to P5. Property P6 is verified *a posteriori* for each tree solution, with a *feedback* mechanism:

Studying each tree solution for calculating the values of HTUs, we eventually discover "impossible sub-trees": they appear in tree solutions which verify P1 to P5, but they do not verify P6.

For each impossible subtree A, an additional constraint is programmed into the model generator to forbid the solutions containing A. Tree solutions are recalculated and verified, allowing the discovery of new impossible subtrees and the programming of new constraints to recalculate the solutions (feedback mechanism). Finally, the complete set of optimal solutions is determined after iteration of this process and elimination of all the solutions that do not verify P6.

}

================================================================================

================================================================================

SOLUTIONS

================================================================================

================================================================================

OTUs:

katharina_tunicata = 0;

nautilus_macromphallus = 1;

loligo_bleekeri = 2;

platynereis_dumerilii = 3;

urechis_caupo = 4;

sipunculus_nudus = 5;

limulus_polyphemus = 6; { = outgroup1 }

homo_sapiens = 7; { = outgroup2 }

loxocorone_allax = 8;

terebratulina_retusa = 9;

phoronis_architecta = 10;

bugula_neritina=11;

terebratalia_transversa=12;

AUXILLIARY CONSTANTS used to fix a part of the solution:

G1=13; { used to fix Cephalopoda group }

G2=14;

G3=15;

G4=16;

G5=17;

G6=18;

G7=19;

G8=20; { used to fix the rest of Eutrochozoa group }

G9=21; { used to fix CASE 1 }

HTUs:

n1, n2, n3, n4, n5, n6, n7, n8

D = [0,29]: 9 solutions OK (which verify property P6) (164 impossible sub-trees)

minimal score (best) = 100

maximal score = 134

-------------------------------------------------------------------------------------------------------------

-> the best model

score = 100:

model 1:

-------------

R(0,1) R(0,9) R(0,10) R(0,13) R(0,14) R(0,20) R(0,n1) R(0,n2) R(0,n3) R(1,0) R(2,13) R(3,15) R(3,16) R(4,17) R(5,19) R(6,21) R(7,21) R(8,20) R(9,0) R(10,0) R(11,n4) R(12,n6) R(13,0) R(13,2) R(14,0) R(14,15) R(15,3) R(15,14) R(15,18) R(16,3) R(16,17) R(17,4) R(17,16) R(18,15) R(18,19) R(19,5) R(19,18) R(20,0) R(20,8) R(21,6) R(21,7) R(21,n1) R(n1,0) R(n1,21) R(n2,0) R(n2,n5) R(n3,0) R(n3,n7) R(n4,11) R(n4,n5) R(n5,n2) R(n5,n4) R(n6,12) R(n6,n8) R(n7,n3) R(n7,n8) R(n8,n6) R(n8,n7)

-------------------------------------------------------------------------------------------------------------

n1(mod2)

[ cox1 cox2 atp8 atp6 nad5 rrnS rrnL nad1 nad6 cob nad4L nad4 cox3 nad3 nad2 ]

score = 110:

model 2:

-------------

R(0,1) R(0,10) R(0,13) R(0,14) R(0,20) R(0,n1) R(0,n2) R(0,n3) R(1,0) R(2,13) R(3,15) R(3,16) R(4,17) R(5,19) R(6,21) R(7,21) R(8,20) R(9,n1) R(10,0) R(11,n4) R(12,n7) R(13,0) R(13,2) R(14,0) R(14,15) R(15,3) R(15,14) R(15,18) R(16,3) R(16,17) R(17,4) R(17,16) R(18,15) R(18,19) R(19,5) R(19,18) R(20,0) R(20,8) R(21,6) R(21,7) R(21,n3) R(n1,0) R(n1,9) R(n1,n6) R(n2,0) R(n2,n5) R(n3,0) R(n3,21) R(n4,11) R(n4,n5) R(n5,n2) R(n5,n4) R(n6,n1) R(n6,n8) R(n7,12) R(n7,n8) R(n8,n6) R(n8,n7)

-------------------------------------------------------------------------------------------------------------

score = 110:

model 3:

-------------

R(0,1) R(0,9) R(0,10) R(0,13) R(0,14) R(0,20) R(0,n1) R(0,n2) R(1,0) R(2,13) R(3,15) R(3,16) R(4,17) R(5,19) R(6,21) R(7,21) R(8,20) R(9,0) R(9,n3) R(10,0) R(11,n7) R(12,n6) R(13,0) R(13,2) R(14,0) R(14,15) R(15,3) R(15,14) R(15,18) R(16,3) R(16,17) R(17,4) R(17,16) R(18,15) R(18,19) R(19,5) R(19,18) R(20,0) R(20,8) R(21,6) R(21,7) R(21,n2) R(n1,0) R(n1,n8) R(n2,0) R(n2,21) R(n3,9) R(n3,n4) R(n4,n3) R(n4,n5) R(n5,n4) R(n5,n6) R(n6,12) R(n6,n5) R(n7,11) R(n7,n8) R(n8,n1) R(n8,n7)

-------------------------------------------------------------------------------------------------------------

n3(mod4)

[ cox1 cox2 atp8 cox3 atp6 rrnS rrnL nad1 nad6 cob nad4L nad4 nad5 nad3 nad2 ]

score = 128:

model 4:

-------------

R(0,1) R(0,10) R(0,13) R(0,14) R(0,20) R(0,n1) R(0,n2) R(1,0) R(2,13) R(3,15) R(3,16) R(4,17) R(5,19) R(6,21) R(7,21) R(8,20) R(9,n3) R(10,0) R(10,n3) R(11,n7) R(12,n6) R(13,0) R(13,2) R(14,0) R(14,15) R(15,3) R(15,14) R(15,18) R(16,3) R(16,17) R(17,4) R(17,16) R(18,15) R(18,19) R(19,5) R(19,18) R(20,0) R(20,8) R(21,6) R(21,7) R(21,n2) R(n1,0) R(n1,n8) R(n2,0) R(n2,21) R(n3,9) R(n3,10) R(n3,n4) R(n4,n3) R(n4,n5) R(n5,n4) R(n5,n6) R(n6,12) R(n6,n5) R(n7,11) R(n7,n8) R(n8,n1) R(n8,n7)

-------------------------------------------------------------------------------------------------------------

score = 110:

model 5:

-------------

R(0,1) R(0,9) R(0,10) R(0,13) R(0,14) R(0,20) R(0,n1) R(0,n2) R(1,0) R(2,13) R(3,15) R(3,16) R(4,17) R(5,19) R(6,21) R(7,21) R(8,20) R(9,0) R(10,0) R(10,n3) R(11,n5) R(12,n6) R(13,0) R(13,2) R(14,0) R(14,15) R(15,3) R(15,14) R(15,18) R(16,3) R(16,17) R(17,4) R(17,16) R(18,15) R(18,19) R(19,5) R(19,18) R(20,0) R(20,8) R(21,6) R(21,7) R(21,n2) R(n1,0) R(n1,n8) R(n2,0) R(n2,21) R(n3,10) R(n3,n4) R(n4,n3) R(n4,n5) R(n5,11) R(n5,n4) R(n6,12) R(n6,n7) R(n7,n6) R(n7,n8) R(n8,n1) R(n8,n7)

-------------------------------------------------------------------------------------------------------------

n1(mod6)

[ cox1 cox2 atp8 atp6 nad5 rrnS rrnL nad1 nad6 cob nad4L nad4 cox3 nad3 nad2 ]

score = 120:

model 6:

-------------

R(0,1) R(0,10) R(0,13) R(0,14) R(0,20) R(0,n1) R(0,n2) R(1,0) R(2,13) R(3,15) R(3,16) R(4,17) R(5,19) R(6,21) R(7,21) R(8,20) R(9,n1) R(10,0) R(10,n3) R(11,n5) R(12,n6) R(13,0) R(13,2) R(14,0) R(14,15) R(15,3) R(15,14) R(15,18) R(16,3) R(16,17) R(17,4) R(17,16) R(18,15) R(18,19) R(19,5) R(19,18) R(20,0) R(20,8) R(21,6) R(21,7) R(21,n2) R(n1,0) R(n1,9) R(n1,n8) R(n2,0) R(n2,21) R(n3,10) R(n3,n4) R(n4,n3) R(n4,n5) R(n5,11) R(n5,n4) R(n6,12) R(n6,n7) R(n7,n6) R(n7,n8) R(n8,n1) R(n8,n7)

-------------------------------------------------------------------------------------------------------------

score = 118:

model 7:

-------------

R(0,1) R(0,9) R(0,10) R(0,13) R(0,14) R(0,20) R(0,n1) R(1,0) R(2,13) R(3,15) R(3,16) R(4,17) R(5,19) R(6,21) R(7,21) R(8,20) R(9,0) R(9,n2) R(9,n3) R(10,0) R(11,n4) R(12,n6) R(13,0) R(13,2) R(14,0) R(14,15) R(15,3) R(15,14) R(15,18) R(16,3) R(16,17) R(17,4) R(17,16) R(18,15) R(18,19) R(19,5) R(19,18) R(20,0) R(20,8) R(21,6) R(21,7) R(21,n1) R(n1,0) R(n1,21) R(n2,9) R(n2,n5) R(n3,9) R(n3,n8) R(n4,11) R(n4,n5) R(n5,n2) R(n5,n4) R(n6,12) R(n6,n7) R(n7,n6) R(n7,n8) R(n8,n3) R(n8,n7)

-------------------------------------------------------------------------------------------------------------

score = 120:

model 8:

-------------

R(0,1) R(0,9) R(0,10) R(0,13) R(0,14) R(0,20) R(0,n1) R(1,0) R(2,13) R(3,15) R(3,16) R(4,17) R(5,19) R(6,21) R(7,21) R(8,20) R(9,0) R(9,n2) R(10,0) R(10,n6) R(11,n7) R(12,n5) R(13,0) R(13,2) R(14,0) R(14,15) R(15,3) R(15,14) R(15,18) R(16,3) R(16,17) R(17,4) R(17,16) R(18,15) R(18,19) R(19,5) R(19,18) R(20,0) R(20,8) R(21,6) R(21,7) R(21,n1) R(n1,0) R(n1,21) R(n2,9) R(n2,n3) R(n3,n2) R(n3,n4) R(n4,n3) R(n4,n5) R(n5,12) R(n5,n4) R(n6,10) R(n6,n8) R(n7,11) R(n7,n8) R(n8,n6) R(n8,n7)

-------------------------------------------------------------------------------------------------------------

n2(mod9)

[ cox1 cox2 atp8 cox3 atp6 rrnS rrnL nad1 nad6 cob nad4L nad4 nad5 nad3 nad2 ]

score = 134:

model 9:

-------------

R(0,1) R(0,10) R(0,13) R(0,14) R(0,20) R(0,n1) R(1,0) R(2,13) R(3,15) R(3,16) R(4,17) R(5,19) R(6,21) R(7,21) R(8,20) R(9,n2) R(10,0) R(10,n2) R(10,n6) R(11,n7) R(12,n5) R(13,0) R(13,2) R(14,0) R(14,15) R(15,3) R(15,14) R(15,18) R(16,3) R(16,17) R(17,4) R(17,16) R(18,15) R(18,19) R(19,5) R(19,18) R(20,0) R(20,8) R(21,6) R(21,7) R(21,n1) R(n1,0) R(n1,21) R(n2,9) R(n2,10) R(n2,n3) R(n3,n2) R(n3,n4) R(n4,n3) R(n4,n5) R(n5,12) R(n5,n4) R(n6,10) R(n6,n8) R(n7,11) R(n7,n8) R(n8,n6) R(n8,n7)

-------------------------------------------------------------------------------------------------------------

-> no other solutions

lophotrochozoans_taxC2_9sol

================================================================================

================================================================================

AXIOMS

================================================================================

================================================================================

{ the solutions of problem PHYLO are the smallest graphs T (defined on the smallest domain possible but containing at least all the OTUs) which verify properties P1 to P6:

P1- T is simple (the relation R(x, y) which defines graph T is not reflexive)

P2- T is non-oriented (the relation R(x, y) which defines graph T is symetrical)

P3- T is connected and acyclic (T is a tree)

P4- T respects the minimal distance matrix, i.e.:

for all couple of OTUs x and y, the length of the path x->y in T is always superior or equals to the minimal distance calculated between x and y (encoded in the minimal distance matrix)

P5- T respects other eventual hypothesis (Primary Phylogenetic Hypothesis = PPH)

used to impose the existence of given monophyletic groups

P6- it is possible to calculate all the values for each HTU in the graph T }

{ OTUs: }

katharina_tunicata = 0;

nautilus_macromphallus = 1;

loligo_bleekeri = 2;

platynereis_dumerilii = 3;

urechis_caupo = 4;

sipunculus_nudus = 5;

limulus_polyphemus = 6; { = outgroup1 }

homo_sapiens = 7; { = outgroup2 }

loxocorone_allax = 8;

terebratulina_retusa = 9;

phoronis_architecta = 10;

bugula_neritina=11;

terebratalia_transversa=12;

{ AUXILLIARY CONSTANTS used to fix a part of the solution: }

G1=13; G2=14; G3=15;

G4=16; G5=17; G6=18;

G7=19; G8=20; { used to fix the Eutrochozoa group }

G9=21; { used to fix CASE 2 }

{ CASE 2 IS FIXED: 2 edges between homo_sapiens and limulus_polyphemus - second form }

R(homo_sapiens,G9);

Q x ( x<> G9

=>

-R(homo_sapiens,x)

);

R(G9,limulus_polyphemus);

Q x ( ( x<>limulus_polyphemus et

x<>homo_sapiens

)

=>

-R(G9,x)

);

{ THE EUTROCHOZOA GROUP IS FIXED: }

{ OTUs (0,1,2): }

R(katharina_tunicata,nautilus_macromphallus);

R(katharina_tunicata,G1);

R(G1,loligo_bleekeri);

Q x ( x<>katharina_tunicata

=>

-R(nautilus_macromphallus,x)

);

Q x ( ( x<>katharina_tunicata et

x<>loligo_bleekeri

)

=>

-R(G1,x)

);

Q x ( x<>G1

=>

-R(loligo_bleekeri,x)

);

{ OTUs (3,4,5,8): }

{ as in the best of the 3 possible forms }

R(katharina_tunicata,G2);

R(G2,G3);

R(G3,platynereis_dumerilii);

R(platynereis_dumerilii,G4);

R(G4,G5);

R(G5,urechis_caupo);

R(G3,G6);

R(G6,G7);

R(G7,sipunculus_nudus);

Q x ( ( x<>katharina_tunicata et

x<>G3

)

=>

-R(G2,x)

);

Q x ( ( x<>G2 et

x<>platynereis_dumerilii et

x<>G6

)

=>

-R(G3,x)

);

Q x ( ( x<>platynereis_dumerilii et

x<>G5

)

=>

-R(G4,x)

);

Q x ( ( x<>G3 et

x<>G4

)

=>

-R(platynereis_dumerilii,x)

);

Q x ( ( x<>G4 et

x<>urechis_caupo

)

=>

-R(G5,x)

);

Q x ( x<>G5

=>

-R(urechis_caupo,x)

);

Q x ( ( x<>G3 et

x<>G7

)

=>

-R(G6,x)

);

Q x ( ( x<>G6 et

x<>sipunculus_nudus

)

=>

-R(G7,x)

);

Q x ( x<>G7

=>

-R(sipunculus_nudus,x)

);

R(katharina_tunicata,G8);

R(G8,loxocorone_allax);

Q x ( ( x<>katharina_tunicata et

x<>loxocorone_allax

)

=>

-R(G8,x)

);

Q x ( x<>G8

=>

-R(loxocorone_allax, x)

);

{ PROPERTY P1: R(x, y) is not reflexive}

Q x (-R(x, x));

{ PROPERTY P2: R(x, y) is symetrical}

Q x y (R(x, y) => R(y, x));

{ PROPERTY P3: graph T is connected and acyclic (T is a tree) }

{

This property is verified by a constraint programmed in the model generator, instead of a "heavy" logical formula:

1- it will refuse the partial interpretations in which a connected component of the graph (in construction) is cyclic, i.e. such as: number of edges >= number of vertices

2- it will refuse the complete interpretations in which the constructed graph has more than one connected component

}

{ PROPERTY P4: graph T respects minimal distance matrix }

{

This property is verified by a constraint programmed in the model generator:

it will refuse the partial interpretations in which the graph (in construction) do not respect the minimal distance matrix, i.e. such as:

let x, y a couple of OTUs,

let d= minimal distance calculated between x and y (encoded in the minimal distance matrix), there is a path of length k between x and y, with: k < d

The minimal distance matrix is encoded directly in the data structure of the model generator:

/* minimal distance matrix lophotrochozoans taxC: */

DIST[0][0]=0;

DIST[1][0]=1; DIST[1][1]=0;

DIST[2][0]=2; DIST[2][1]=3; DIST[2][2]=0;

DIST[3][0]=3; DIST[3][1]=4; DIST[3][2]=4; DIST[3][3]=0;

DIST[4][0]=5; DIST[4][1]=5; DIST[4][2]=5; DIST[4][3]=3; DIST[4][4]=0;

DIST[5][0]=4; DIST[5][1]=4; DIST[5][2]=5; DIST[5][3]=3; DIST[5][4]=4; DIST[5][5]=0;

DIST[6][0]=2; DIST[6][1]=3; DIST[6][2]=2; DIST[6][3]=4; DIST[6][4]=5; DIST[6][5]=4; DIST[6][6]=0;

DIST[7][0]=3; DIST[7][1]=4; DIST[7][2]=3; DIST[7][3]=4; DIST[7][4]=5; DIST[7][5]=4; DIST[7][6]=2; DIST[7][7]=0;

DIST[8][0]=2; DIST[8][1]=2; DIST[8][2]=4; DIST[8][3]=5; DIST[8][4]=5; DIST[8][5]=5; DIST[8][6]=4; DIST[8][7]=4; DIST[8][8]=0;

DIST[9][0]=1; DIST[9][1]=2; DIST[9][2]=2; DIST[9][3]=3; DIST[9][4]=4; DIST[9][5]=5; DIST[9][6]=2; DIST[9][7]=3; DIST[9][8]=3; DIST[9][9]=0;

DIST[10][0]=1; DIST[10][1]=2; DIST[10][2]=3; DIST[10][3]=4; DIST[10][4]=5; DIST[10][5]=4; DIST[10][6]=3; DIST[10][7]=4; DIST[10][8]=3; DIST[10][9]=2; DIST[10][10]=0;

DIST[11][0]=4; DIST[11][1]=5; DIST[11][2]=5; DIST[11][3]=6; DIST[11][4]=6; DIST[11][5]=6; DIST[11][6]=5; DIST[11][7]=5; DIST[11][8]=5; DIST[11][9]=4; DIST[11][10]=4; DIST[11][11]=0;

DIST[12][0]=5; DIST[12][1]=6; DIST[12][2]=6; DIST[12][3]=5; DIST[12][4]=7; DIST[12][5]=6; DIST[12][6]=6; DIST[12][7]=7; DIST[12][8]=7; DIST[12][9]=5; DIST[12][10]=5; DIST[12][11]=7; DIST[12][12]=0;

}

{ PROPERTY P5: graph T respects eventual Primary Phylogenetic Hypotheses }

{

This property is verified by constraints programmed in the model generator:

- monophyly of Lophotrochozoa = (0,1,2,3,4,5,8,9,10,11,12)

- monophyly of Eutrochozoa = (0,1,2,3,4,5,8)

- monophyly of Mollusca = (0,1,2)

- monophyly of Polyplacophora = (0)

- monophyly of Cephalopoda = (0,1,2)

- monophyly of Annelida = (3,4)

- monophyly of Echiura = (3,4)

- monophyly of Polycheta = (3)

- monophyly of Lophophorata = (9,10,11,12)

- monophyly of Brachiopoda = (9,12)

Notes:

1- katharina_tunicata mtDNA is identical to octopus_vulgaris mtDNA (mollusc *Cephalopoda*), considering only the 15 protein-coding genes and rRNA genes. Thus, katharina_tunicata must be also element of Cephalopoda clade in this computation.

2- platynereis_dumerilii mtDNA is identical to clymenella_torquata mtDNA (annelid *Echiura*). Thus, platynereis_dumerilii must be also element of *Echiura* clade in this computation.

}

{------------------------------------------------------------------------------------------------------------------------}

{ PROPERTY P6: it is possible to calculate all the values for each HTU in the graph T }

{

First we calculate with the model generator the set of tree solutions which verify properties P1 to P5. Property P6 is verified *a posteriori* for each tree solution, with a *feedback* mechanism:

Studying each tree solution for calculating the values of HTUs, we eventually discover "impossible sub-trees": they appear in tree solutions which verify P1 to P5, but they do not verify P6.

For each impossible subtree A, an additional constraint is programmed into the model generator to forbid the solutions containing A. Tree solutions are recalculated and verified, allowing the discovery of new impossible subtrees and the programming of new constraints to recalculate the solutions (feedback mechanism). Finally, the complete set of optimal solutions is determined after iteration of this process and elimination of all the solutions that do not verify P6.

}

================================================================================

================================================================================

SOLUTIONS

================================================================================

================================================================================

OTUs:

katharina_tunicata = 0;

nautilus_macromphallus = 1;

loligo_bleekeri = 2;

platynereis_dumerilii = 3;

urechis_caupo = 4;

sipunculus_nudus = 5;

limulus_polyphemus = 6; { = outgroup1 }

homo_sapiens = 7; { = outgroup2 }

loxocorone_allax = 8;

terebratulina_retusa = 9;

phoronis_architecta = 10;

bugula_neritina=11;

terebratalia_transversa=12;

AUXILLIARY CONSTANTS used to fix a part of the solution:

G1=13; { used to fix Cephalopoda group }

G2=14;

G3=15;

G4=16;

G5=17;

G6=18;

G7=19;

G8=20; { used to fix the rest of Eutrochozoa group }

G9=21; { used to fix CASE 2 }

HTUs:

n1, n2, n3, n4, n5, n6, n7, n8

D = [0,29]: 9 solutions OK (which verify property P6) (167 impossible sub-trees)

minimal score (best) = 100

maximal score = 134

-------------------------------------------------------------------------------------------------------------

-> the best model

score = 100:

model 1:

-------------

R(0,1) R(0,9) R(0,10) R(0,13) R(0,14) R(0,20) R(0,n1) R(0,n2) R(0,n3) R(1,0) R(2,13) R(3,15) R(3,16) R(4,17) R(5,19) R(6,21) R(6,n1) R(7,21) R(8,20) R(9,0) R(10,0) R(11,n4) R(12,n6) R(13,0) R(13,2) R(14,0) R(14,15) R(15,3) R(15,14) R(15,18) R(16,3) R(16,17) R(17,4) R(17,16) R(18,15) R(18,19) R(19,5) R(19,18) R(20,0) R(20,8) R(21,6) R(21,7) R(n1,0) R(n1,6) R(n2,0) R(n2,n5) R(n3,0) R(n3,n7) R(n4,11) R(n4,n5) R(n5,n2) R(n5,n4) R(n6,12) R(n6,n8) R(n7,n3) R(n7,n8) R(n8,n6) R(n8,n7)

-------------------------------------------------------------------------------------------------------------

n3(mod2)

[ cox1 cox2 atp8 atp6 nad5 rrnS rrnL nad1 nad6 cob nad4L nad4 cox3 nad3 nad2 ]

score = 110:

model 2:

-------------

R(0,1) R(0,10) R(0,13) R(0,14) R(0,20) R(0,n1) R(0,n2) R(0,n3) R(1,0) R(2,13) R(3,15) R(3,16) R(4,17) R(5,19) R(6,21) R(6,n1) R(7,21) R(8,20) R(9,n3) R(10,0) R(11,n4) R(12,n6) R(13,0) R(13,2) R(14,0) R(14,15) R(15,3) R(15,14) R(15,18) R(16,3) R(16,17) R(17,4) R(17,16) R(18,15) R(18,19) R(19,5) R(19,18) R(20,0) R(20,8) R(21,6) R(21,7) R(n1,0) R(n1,6) R(n2,0) R(n2,n5) R(n3,0) R(n3,9) R(n3,n7) R(n4,11) R(n4,n5) R(n5,n2) R(n5,n4) R(n6,12) R(n6,n8) R(n7,n3) R(n7,n8) R(n8,n6) R(n8,n7)

-------------------------------------------------------------------------------------------------------------

score = 110:

model 3:

-------------

R(0,1) R(0,9) R(0,10) R(0,13) R(0,14) R(0,20) R(0,n1) R(0,n2) R(1,0) R(2,13) R(3,15) R(3,16) R(4,17) R(5,19) R(6,21) R(6,n2) R(7,21) R(8,20) R(9,0) R(9,n3) R(10,0) R(11,n7) R(12,n6) R(13,0) R(13,2) R(14,0) R(14,15) R(15,3) R(15,14) R(15,18) R(16,3) R(16,17) R(17,4) R(17,16) R(18,15) R(18,19) R(19,5) R(19,18) R(20,0) R(20,8) R(21,6) R(21,7) R(n1,0) R(n1,n8) R(n2,0) R(n2,6) R(n3,9) R(n3,n4) R(n4,n3) R(n4,n5) R(n5,n4) R(n5,n6) R(n6,12) R(n6,n5) R(n7,11) R(n7,n8) R(n8,n1) R(n8,n7)

-------------------------------------------------------------------------------------------------------------

n3(mod4)

[ cox1 cox2 atp8 cox3 atp6 rrnS rrnL nad1 nad6 cob nad4L nad4 nad5 nad3 nad2 ]

score = 128:

model 4:

-------------

R(0,1) R(0,10) R(0,13) R(0,14) R(0,20) R(0,n1) R(0,n2) R(1,0) R(2,13) R(3,15) R(3,16) R(4,17) R(5,19) R(6,21) R(6,n1) R(7,21) R(8,20) R(9,n3) R(10,0) R(10,n3) R(11,n6) R(12,n8) R(13,0) R(13,2) R(14,0) R(14,15) R(15,3) R(15,14) R(15,18) R(16,3) R(16,17) R(17,4) R(17,16) R(18,15) R(18,19) R(19,5) R(19,18) R(20,0) R(20,8) R(21,6) R(21,7) R(n1,0) R(n1,6) R(n2,0) R(n2,n7) R(n3,9) R(n3,10) R(n3,n4) R(n4,n3) R(n4,n5) R(n5,n4) R(n5,n8) R(n6,11) R(n6,n7) R(n7,n2) R(n7,n6) R(n8,12) R(n8,n5)

-------------------------------------------------------------------------------------------------------------

score = 110:

model 5:

-------------

R(0,1) R(0,9) R(0,10) R(0,13) R(0,14) R(0,20) R(0,n1) R(0,n2) R(1,0) R(2,13) R(3,15) R(3,16) R(4,17) R(5,19) R(6,21) R(6,n2) R(7,21) R(8,20) R(9,0) R(10,0) R(10,n3) R(11,n5) R(12,n6) R(13,0) R(13,2) R(14,0) R(14,15) R(15,3) R(15,14) R(15,18) R(16,3) R(16,17) R(17,4) R(17,16) R(18,15) R(18,19) R(19,5) R(19,18) R(20,0) R(20,8) R(21,6) R(21,7) R(n1,0) R(n1,n8) R(n2,0) R(n2,6) R(n3,10) R(n3,n4) R(n4,n3) R(n4,n5) R(n5,11) R(n5,n4) R(n6,12) R(n6,n7) R(n7,n6) R(n7,n8) R(n8,n1) R(n8,n7)

-------------------------------------------------------------------------------------------------------------

n1(mod6)

[ cox1 cox2 atp8 atp6 nad5 rrnS rrnL nad1 nad6 cob nad4L nad4 cox3 nad3 nad2 ]

score = 120:

model 6:

-------------

R(0,1) R(0,10) R(0,13) R(0,14) R(0,20) R(0,n1) R(0,n2) R(1,0) R(2,13) R(3,15) R(3,16) R(4,17) R(5,19) R(6,21) R(6,n2) R(7,21) R(8,20) R(9,n1) R(10,0) R(10,n3) R(11,n5) R(12,n6) R(13,0) R(13,2) R(14,0) R(14,15) R(15,3) R(15,14) R(15,18) R(16,3) R(16,17) R(17,4) R(17,16) R(18,15) R(18,19) R(19,5) R(19,18) R(20,0) R(20,8) R(21,6) R(21,7) R(n1,0) R(n1,9) R(n1,n8) R(n2,0) R(n2,6) R(n3,10) R(n3,n4) R(n4,n3) R(n4,n5) R(n5,11) R(n5,n4) R(n6,12) R(n6,n7) R(n7,n6) R(n7,n8) R(n8,n1) R(n8,n7)

-------------------------------------------------------------------------------------------------------------

score = 128:

model 7:

-------------

R(0,1) R(0,9) R(0,10) R(0,13) R(0,14) R(0,20) R(0,n1) R(1,0) R(2,13) R(3,15) R(3,16) R(4,17) R(5,19) R(6,21) R(6,n1) R(7,21) R(8,20) R(9,0) R(9,n2) R(9,n3) R(10,0) R(11,n4) R(12,n6) R(13,0) R(13,2) R(14,0) R(14,15) R(15,3) R(15,14) R(15,18) R(16,3) R(16,17) R(17,4) R(17,16) R(18,15) R(18,19) R(19,5) R(19,18) R(20,0) R(20,8) R(21,6) R(21,7) R(n1,0) R(n1,6) R(n2,9) R(n2,n5) R(n3,9) R(n3,n8) R(n4,11) R(n4,n5) R(n5,n2) R(n5,n4) R(n6,12) R(n6,n7) R(n7,n6) R(n7,n8) R(n8,n3) R(n8,n7)

-------------------------------------------------------------------------------------------------------------

score = 120:

model 8:

-------------

R(0,1) R(0,9) R(0,10) R(0,13) R(0,14) R(0,20) R(0,n1) R(1,0) R(2,13) R(3,15) R(3,16) R(4,17) R(5,19) R(6,21) R(6,n1) R(7,21) R(8,20) R(9,0) R(9,n2) R(10,0) R(10,n6) R(11,n8) R(12,n5) R(13,0) R(13,2) R(14,0) R(14,15) R(15,3) R(15,14) R(15,18) R(16,3) R(16,17) R(17,4) R(17,16) R(18,15) R(18,19) R(19,5) R(19,18) R(20,0) R(20,8) R(21,6) R(21,7) R(n1,0) R(n1,6) R(n2,9) R(n2,n3) R(n3,n2) R(n3,n4) R(n4,n3) R(n4,n5) R(n5,12) R(n5,n4) R(n6,10) R(n6,n7) R(n7,n6) R(n7,n8) R(n8,11) R(n8,n7)

-------------------------------------------------------------------------------------------------------------

n2(mod9)

[ cox1 cox2 atp8 cox3 atp6 rrnS rrnL nad1 nad6 cob nad4L nad4 nad5 nad3 nad2 ]

score = 134:

model 9:

-------------

R(0,1) R(0,10) R(0,13) R(0,14) R(0,20) R(0,n1) R(1,0) R(2,13) R(3,15) R(3,16) R(4,17) R(5,19) R(6,21) R(6,n1) R(7,21) R(8,20) R(9,n2) R(10,0) R(10,n2) R(10,n6) R(11,n8) R(12,n5) R(13,0) R(13,2) R(14,0) R(14,15) R(15,3) R(15,14) R(15,18) R(16,3) R(16,17) R(17,4) R(17,16) R(18,15) R(18,19) R(19,5) R(19,18) R(20,0) R(20,8) R(21,6) R(21,7) R(n1,0) R(n1,6) R(n2,9) R(n2,10) R(n2,n3) R(n3,n2) R(n3,n4) R(n4,n3) R(n4,n5) R(n5,12) R(n5,n4) R(n6,10) R(n6,n7) R(n7,n6) R(n7,n8) R(n8,11) R(n8,n7)

-------------------------------------------------------------------------------------------------------------

-> no other solutions

lophotrochozoans_taxC3_9sol

================================================================================

================================================================================

AXIOMS

================================================================================

================================================================================

{ the solutions of problem PHYLO are the smallest graphs T (defined on the smallest domain possible but containing at least all the OTUs) which verify properties P1 to P6:

P1- T is simple (the relation R(x, y) which defines graph T is not reflexive)

P2- T is non-oriented (the relation R(x, y) which defines graph T is symetrical)

P3- T is connected and acyclic (T is a tree)

P4- T respects the minimal distance matrix, i.e.:

for all couple of OTUs x and y, the length of the path x->y in T is always superior or equals to the minimal distance calculated between x and y (encoded in the minimal distance matrix)

P5- T respects other eventual hypothesis (Primary Phylogenetic Hypothesis = PPH)

used to impose the existence of given monophyletic groups

P6- it is possible to calculate all the values for each HTU in the graph T }

{ OTUs: }

katharina_tunicata = 0;

nautilus_macromphallus = 1;

loligo_bleekeri = 2;

platynereis_dumerilii = 3;

urechis_caupo = 4;

sipunculus_nudus = 5;

limulus_polyphemus = 6; { = outgroup1 }

homo_sapiens = 7; { = outgroup2 }

loxocorone_allax = 8;

terebratulina_retusa = 9;

phoronis_architecta = 10;

bugula_neritina=11;

terebratalia_transversa=12;

{ AUXILLIARY CONSTANTS used to fix a part of the solution: }

G1=13; G2=14; G3=15;

G4=16; G5=17; G6=18;

G7=19; G8=20; { used to fix the Eutrochozoa group }

G9=21; G10=22; { used to fix CASE 3 }

{ CASE 3 IS FIXED: 3 edges between homo_sapiens and limulus_polyphemus - first form }

R(homo_sapiens,G9);

Q x ( x<> G9

=>

-R(homo_sapiens,x)

);

R(G9,G10);

Q x ( ( x<>G10 et

x<>homo_sapiens

)

=>

-R(G9,x)

);

R(limulus_polyphemus,G10);

Q x ( x<> G10

=>

-R(limulus_polyphemus,x)

);

{ THE EUTROCHOZOA GROUP IS FIXED: }

{ OTUs (0,1,2): }

R(katharina_tunicata,nautilus_macromphallus);

R(katharina_tunicata,G1);

R(G1,loligo_bleekeri);

Q x ( x<>katharina_tunicata

=>

-R(nautilus_macromphallus,x)

);

Q x ( ( x<>katharina_tunicata et

x<>loligo_bleekeri

)

=>

-R(G1,x)

);

Q x ( x<>G1

=>

-R(loligo_bleekeri,x)

);

{ OTUs (3,4,5,8): }

{ as in the best of the 3 possible forms }

R(katharina_tunicata,G2);

R(G2,G3);

R(G3,platynereis_dumerilii);

R(platynereis_dumerilii,G4);

R(G4,G5);

R(G5,urechis_caupo);

R(G3,G6);

R(G6,G7);

R(G7,sipunculus_nudus);

Q x ( ( x<>katharina_tunicata et

x<>G3

)

=>

-R(G2,x)

);

Q x ( ( x<>G2 et

x<>platynereis_dumerilii et

x<>G6

)

=>

-R(G3,x)

);

Q x ( ( x<>platynereis_dumerilii et

x<>G5

)

=>

-R(G4,x)

);

Q x ( ( x<>G3 et

x<>G4

)

=>

-R(platynereis_dumerilii,x)

);

Q x ( ( x<>G4 et

x<>urechis_caupo

)

=>

-R(G5,x)

);

Q x ( x<>G5

=>

-R(urechis_caupo,x)

);

Q x ( ( x<>G3 et

x<>G7

)

=>

-R(G6,x)

);

Q x ( ( x<>G6 et

x<>sipunculus_nudus

)

=>

-R(G7,x)

);

Q x ( x<>G7

=>

-R(sipunculus_nudus,x)

);

R(katharina_tunicata,G8);

R(G8,loxocorone_allax);

Q x ( ( x<>katharina_tunicata et

x<>loxocorone_allax

)

=>

-R(G8,x)

);

Q x ( x<>G8

=>

-R(loxocorone_allax, x)

);

{ PROPERTY P1: R(x, y) is not reflexive}

Q x (-R(x, x));

{ PROPERTY P2: R(x, y) is symetrical}

Q x y (R(x, y) => R(y, x));

{ PROPERTY P3: graph T is connected and acyclic (T is a tree) }

{

This property is verified by a constraint programmed in the model generator, instead of a "heavy" logical formula:

1- it will refuse the partial interpretations in which a connected component of the graph (in construction) is cyclic, i.e. such as: number of edges >= number of vertices

2- it will refuse the complete interpretations in which the constructed graph has more than one connected component

}

{ PROPERTY P4: graph T respects minimal distance matrix }

{

This property is verified by a constraint programmed in the model generator:

it will refuse the partial interpretations in which the graph (in construction) do not respect the minimal distance matrix, i.e. such as:

let x, y a couple of OTUs,

let d= minimal distance calculated between x and y (encoded in the minimal distance matrix), there is a path of length k between x and y, with: k < d

The minimal distance matrix is encoded directly in the data structure of the model generator:

/* minimal distance matrix lophotrochozoans taxC: */

DIST[0][0]=0;

DIST[1][0]=1; DIST[1][1]=0;

DIST[2][0]=2; DIST[2][1]=3; DIST[2][2]=0;

DIST[3][0]=3; DIST[3][1]=4; DIST[3][2]=4; DIST[3][3]=0;

DIST[4][0]=5; DIST[4][1]=5; DIST[4][2]=5; DIST[4][3]=3; DIST[4][4]=0;

DIST[5][0]=4; DIST[5][1]=4; DIST[5][2]=5; DIST[5][3]=3; DIST[5][4]=4; DIST[5][5]=0;

DIST[6][0]=2; DIST[6][1]=3; DIST[6][2]=2; DIST[6][3]=4; DIST[6][4]=5; DIST[6][5]=4; DIST[6][6]=0;

DIST[7][0]=3; DIST[7][1]=4; DIST[7][2]=3; DIST[7][3]=4; DIST[7][4]=5; DIST[7][5]=4; DIST[7][6]=2; DIST[7][7]=0;

DIST[8][0]=2; DIST[8][1]=2; DIST[8][2]=4; DIST[8][3]=5; DIST[8][4]=5; DIST[8][5]=5; DIST[8][6]=4; DIST[8][7]=4; DIST[8][8]=0;

DIST[9][0]=1; DIST[9][1]=2; DIST[9][2]=2; DIST[9][3]=3; DIST[9][4]=4; DIST[9][5]=5; DIST[9][6]=2; DIST[9][7]=3; DIST[9][8]=3; DIST[9][9]=0;

DIST[10][0]=1; DIST[10][1]=2; DIST[10][2]=3; DIST[10][3]=4; DIST[10][4]=5; DIST[10][5]=4; DIST[10][6]=3; DIST[10][7]=4; DIST[10][8]=3; DIST[10][9]=2; DIST[10][10]=0;

DIST[11][0]=4; DIST[11][1]=5; DIST[11][2]=5; DIST[11][3]=6; DIST[11][4]=6; DIST[11][5]=6; DIST[11][6]=5; DIST[11][7]=5; DIST[11][8]=5; DIST[11][9]=4; DIST[11][10]=4; DIST[11][11]=0;

DIST[12][0]=5; DIST[12][1]=6; DIST[12][2]=6; DIST[12][3]=5; DIST[12][4]=7; DIST[12][5]=6; DIST[12][6]=6; DIST[12][7]=7; DIST[12][8]=7; DIST[12][9]=5; DIST[12][10]=5; DIST[12][11]=7; DIST[12][12]=0;

}

{ PROPERTY P5: graph T respects eventual Primary Phylogenetic Hypotheses }

{

This property is verified by constraints programmed in the model generator:

- monophyly of Lophotrochozoa = (0,1,2,3,4,5,8,9,10,11,12)

- monophyly of Eutrochozoa = (0,1,2,3,4,5,8)

- monophyly of Mollusca = (0,1,2)

- monophyly of Polyplacophora = (0)

- monophyly of Cephalopoda = (0,1,2)

- monophyly of Annelida = (3,4)

- monophyly of Echiura = (3,4)

- monophyly of Polycheta = (3)

- monophyly of Lophophorata = (9,10,11,12)

- monophyly of Brachiopoda = (9,12)

Notes:

1- katharina_tunicata mtDNA is identical to octopus_vulgaris mtDNA (mollusc *Cephalopoda*), considering only the 15 protein-coding genes and rRNA genes. Thus, katharina_tunicata must be also element of Cephalopoda clade in this computation.

2- platynereis_dumerilii mtDNA is identical to clymenella_torquata mtDNA (annelid *Echiura*). Thus, platynereis_dumerilii must be also element of *Echiura* clade in this computation.

}

{------------------------------------------------------------------------------------------------------------------------}

{ PROPERTY P6: it is possible to calculate all the values for each HTU in the graph T }

{

First we calculate with the model generator the set of tree solutions which verify properties P1 to P5. Property P6 is verified *a posteriori* for each tree solution, with a *feedback* mechanism:

Studying each tree solution for calculating the values of HTUs, we eventually discover "impossible sub-trees": they appear in tree solutions which verify P1 to P5, but they do not verify P6.

For each impossible subtree A, an additional constraint is programmed into the model generator to forbid the solutions containing A. Tree solutions are recalculated and verified, allowing the discovery of new impossible subtrees and the programming of new constraints to recalculate the solutions (feedback mechanism). Finally, the complete set of optimal solutions is determined after iteration of this process and elimination of all the solutions that do not verify P6.

}

================================================================================

================================================================================

SOLUTIONS

================================================================================

================================================================================

OTUs:

katharina_tunicata = 0;

nautilus_macromphallus = 1;

loligo_bleekeri = 2;

platynereis_dumerilii = 3;

urechis_caupo = 4;

sipunculus_nudus = 5;

limulus_polyphemus = 6; { = outgroup1 }

homo_sapiens = 7; { = outgroup2 }

loxocorone_allax = 8;

terebratulina_retusa = 9;

phoronis_architecta = 10;

bugula_neritina=11;

terebratalia_transversa=12;

AUXILLIARY CONSTANTS used to fix a part of the solution:

G1=13; { used to fix Cephalopoda group }

G2=14;

G3=15;

G4=16;

G5=17;

G6=18;

G7=19;

G8=20; { used to fix the rest of Eutrochozoa group }

G9=21;

G10=22; { used to fix CASE 3 }

HTUs:

n1, n2, n3, n4, n5, n6, n7

D = [0,29]: 9 solutions OK (which verify property P6) (167 impossible sub-trees)

minimal score (best) = 90

maximal score = 124

-------------------------------------------------------------------------------------------------------------

-> the best model

score = 90:

model 1:

-------------

R(0,1) R(0,9) R(0,10) R(0,13) R(0,14) R(0,20) R(0,22) R(0,n1) R(0,n2) R(1,0) R(2,13) R(3,15) R(3,16) R(4,17) R(5,19) R(6,22) R(7,21) R(8,20) R(9,0) R(10,0) R(11,n3) R(12,n5) R(13,0) R(13,2) R(14,0) R(14,15) R(15,3) R(15,14) R(15,18) R(16,3) R(16,17) R(17,4) R(17,16) R(18,15) R(18,19) R(19,5) R(19,18) R(20,0) R(20,8) R(21,7) R(21,22) R(22,0) R(22,6) R(22,21) R(n1,0) R(n1,n4) R(n2,0) R(n2,n7) R(n3,11) R(n3,n4) R(n4,n1) R(n4,n3) R(n5,12) R(n5,n6) R(n6,n5) R(n6,n7) R(n7,n2) R(n7,n6)

-------------------------------------------------------------------------------------------------------------

score = 100:

model 2:

-------------

R(0,1) R(0,10) R(0,13) R(0,14) R(0,20) R(0,22) R(0,n1) R(0,n2) R(1,0) R(2,13) R(3,15) R(3,16) R(4,17) R(5,19) R(6,22) R(7,21) R(8,20) R(9,n2) R(10,0) R(11,n3) R(12,n5) R(13,0) R(13,2) R(14,0) R(14,15) R(15,3) R(15,14) R(15,18) R(16,3) R(16,17) R(17,4) R(17,16) R(18,15) R(18,19) R(19,5) R(19,18) R(20,0) R(20,8) R(21,7) R(21,22) R(22,0) R(22,6) R(22,21) R(n1,0) R(n1,n4) R(n2,0) R(n2,9) R(n2,n7) R(n3,11) R(n3,n4) R(n4,n1) R(n4,n3) R(n5,12) R(n5,n6) R(n6,n5) R(n6,n7) R(n7,n2) R(n7,n6)

-------------------------------------------------------------------------------------------------------------

score = 100:

model 3:

-------------

R(0,1) R(0,9) R(0,10) R(0,13) R(0,14) R(0,20) R(0,22) R(0,n1) R(1,0) R(2,13) R(3,15) R(3,16) R(4,17) R(5,19) R(6,22) R(7,21) R(8,20) R(9,0) R(10,0) R(10,n5) R(11,n6) R(12,n4) R(13,0) R(13,2) R(14,0) R(14,15) R(15,3) R(15,14) R(15,18) R(16,3) R(16,17) R(17,4) R(17,16) R(18,15) R(18,19) R(19,5) R(19,18) R(20,0) R(20,8) R(21,7) R(21,22) R(22,0) R(22,6) R(22,21) R(n1,0) R(n1,n2) R(n2,n1) R(n2,n3) R(n3,n2) R(n3,n4) R(n4,12) R(n4,n3) R(n5,10) R(n5,n7) R(n6,11) R(n6,n7) R(n7,n5) R(n7,n6)

-------------------------------------------------------------------------------------------------------------

score = 110:

model 4:

-------------

R(0,1) R(0,10) R(0,13) R(0,14) R(0,20) R(0,22) R(0,n1) R(1,0) R(2,13) R(3,15) R(3,16) R(4,17) R(5,19) R(6,22) R(7,21) R(8,20) R(9,n1) R(10,0) R(10,n5) R(11,n6) R(12,n4) R(13,0) R(13,2) R(14,0) R(14,15) R(15,3) R(15,14) R(15,18) R(16,3) R(16,17) R(17,4) R(17,16) R(18,15) R(18,19) R(19,5) R(19,18) R(20,0) R(20,8) R(21,7) R(21,22) R(22,0) R(22,6) R(22,21) R(n1,0) R(n1,9) R(n1,n2) R(n2,n1) R(n2,n3) R(n3,n2) R(n3,n4) R(n4,12) R(n4,n3) R(n5,10) R(n5,n7) R(n6,11) R(n6,n7) R(n7,n5) R(n7,n6)

-------------------------------------------------------------------------------------------------------------

score = 100:

model 5:

-------------

R(0,1) R(0,9) R(0,10) R(0,13) R(0,14) R(0,20) R(0,22) R(0,n1) R(1,0) R(2,13) R(3,15) R(3,16) R(4,17) R(5,19) R(6,22) R(7,21) R(8,20) R(9,0) R(9,n4) R(10,0) R(11,n3) R(12,n5) R(13,0) R(13,2) R(14,0) R(14,15) R(15,3) R(15,14) R(15,18) R(16,3) R(16,17) R(17,4) R(17,16) R(18,15) R(18,19) R(19,5) R(19,18) R(20,0) R(20,8) R(21,7) R(21,22) R(22,0) R(22,6) R(22,21) R(n1,0) R(n1,n2) R(n2,n1) R(n2,n3) R(n3,11) R(n3,n2) R(n4,9) R(n4,n6) R(n5,12) R(n5,n7) R(n6,n4) R(n6,n7) R(n7,n5) R(n7,n6)

-------------------------------------------------------------------------------------------------------------

score = 118:

model 6:

-------------

R(0,1) R(0,10) R(0,13) R(0,14) R(0,20) R(0,22) R(0,n1) R(1,0) R(2,13) R(3,15) R(3,16) R(4,17) R(5,19) R(6,22) R(7,21) R(8,20) R(9,n4) R(10,0) R(10,n4) R(11,n3) R(12,n5) R(13,0) R(13,2) R(14,0) R(14,15) R(15,3) R(15,14) R(15,18) R(16,3) R(16,17) R(17,4) R(17,16) R(18,15) R(18,19) R(19,5) R(19,18) R(20,0) R(20,8) R(21,7) R(21,22) R(22,0) R(22,6) R(22,21) R(n1,0) R(n1,n2) R(n2,n1) R(n2,n3) R(n3,11) R(n3,n2) R(n4,9) R(n4,10) R(n4,n6) R(n5,12) R(n5,n7) R(n6,n4) R(n6,n7) R(n7,n5) R(n7,n6)

-------------------------------------------------------------------------------------------------------------

score = 108:

model 7:

-------------

R(0,1) R(0,9) R(0,10) R(0,13) R(0,14) R(0,20) R(0,22) R(1,0) R(2,13) R(3,15) R(3,16) R(4,17) R(5,19) R(6,22) R(7,21) R(8,20) R(9,0) R(9,n1) R(9,n2) R(10,0) R(11,n3) R(12,n5) R(13,0) R(13,2) R(14,0) R(14,15) R(15,3) R(15,14) R(15,18) R(16,3) R(16,17) R(17,4) R(17,16) R(18,15) R(18,19) R(19,5) R(19,18) R(20,0) R(20,8) R(21,7) R(21,22) R(22,0) R(22,6) R(22,21) R(n1,9) R(n1,n4) R(n2,9) R(n2,n7) R(n3,11) R(n3,n4) R(n4,n1) R(n4,n3) R(n5,12) R(n5,n6) R(n6,n5) R(n6,n7) R(n7,n2) R(n7,n6)

-------------------------------------------------------------------------------------------------------------

score = 110:

model 8:

-------------

R(0,1) R(0,9) R(0,10) R(0,13) R(0,14) R(0,20) R(0,22) R(1,0) R(2,13) R(3,15) R(3,16) R(4,17) R(5,19) R(6,22) R(7,21) R(8,20) R(9,0) R(9,n1) R(10,0) R(10,n5) R(11,n6) R(12,n4) R(13,0) R(13,2) R(14,0) R(14,15) R(15,3) R(15,14) R(15,18) R(16,3) R(16,17) R(17,4) R(17,16) R(18,15) R(18,19) R(19,5) R(19,18) R(20,0) R(20,8) R(21,7) R(21,22) R(22,0) R(22,6) R(22,21) R(n1,9) R(n1,n2) R(n2,n1) R(n2,n3) R(n3,n2) R(n3,n4) R(n4,12) R(n4,n3) R(n5,10) R(n5,n7) R(n6,11) R(n6,n7) R(n7,n5) R(n7,n6)

-------------------------------------------------------------------------------------------------------------

score = 124:

model 9:

-------------

R(0,1) R(0,10) R(0,13) R(0,14) R(0,20) R(0,22) R(1,0) R(2,13) R(3,15) R(3,16) R(4,17) R(5,19) R(6,22) R(7,21) R(8,20) R(9,n1) R(10,0) R(10,n1) R(10,n5) R(11,n6) R(12,n4) R(13,0) R(13,2) R(14,0) R(14,15) R(15,3) R(15,14) R(15,18) R(16,3) R(16,17) R(17,4) R(17,16) R(18,15) R(18,19) R(19,5) R(19,18) R(20,0) R(20,8) R(21,7) R(21,22) R(22,0) R(22,6) R(22,21) R(n1,9) R(n1,10) R(n1,n2) R(n2,n1) R(n2,n3) R(n3,n2) R(n3,n4) R(n4,12) R(n4,n3) R(n5,10) R(n5,n7) R(n6,11) R(n6,n7) R(n7,n5) R(n7,n6)

-------------------------------------------------------------------------------------------------------------

-> no other solutions

lophotrochozoans_taxD_6sol

================================================================================

================================================================================

AXIOMS

================================================================================

================================================================================

{ the solutions of problem PHYLO are the smallest graphs T (defined on the smallest domain possible but containing at least all the OTUs) which verify properties P1 to P6:

P1- T is simple (the relation R(x, y) which defines graph T is not reflexive)

P2- T is non-oriented (the relation R(x, y) which defines graph T is symetrical)

P3- T is connected and acyclic (T is a tree)

P4- T respects the minimal distance matrix, i.e.:

for all couple of OTUs x and y, the length of the path x->y in T is always superior or equals to the minimal distance calculated between x and y (encoded in the minimal distance matrix)

P5- T respects other eventual hypothesis (Primary Phylogenetic Hypothesis = PPH)

used to impose the existence of given monophyletic groups

P6- it is possible to calculate all the values for each HTU in the graph T }

{ OTUs: }

katharina_tunicata = 0;

nautilus_macromphallus = 1;

loligo_bleekeri = 2;

albinaria_soerulea = 3;

cepaea_nemoralis = 4;

biomphalaria_glabrata = 5;

priapulus_caudatus = 6; { = outgroup1 }

homo_sapiens = 7; { = outgroup2 }

{ PROPERTY P1: R(x, y) is not reflexive}

Q x (-R(x, x));

{ PROPERTY P2: R(x, y) is symetrical}

Q x y (R(x, y) => R(y, x));

{ PROPERTY P3: graph T is connected and acyclic (T is a tree) }

{

This property is verified by a constraint programmed in the model generator, instead of a "heavy" logical formula:

1- it will refuse the partial interpretations in which a connected component of the graph (in construction) is cyclic, i.e. such as: number of edges >= number of vertices

2- it will refuse the complete interpretations in which the constructed graph has more than one connected component

}

{ PROPERTY P4: graph T respects minimal distance matrix }

{

This property is verified by a constraint programmed in the model generator:

it will refuse the partial interpretations in which the graph (in construction) do not respect the minimal distance matrix, i.e. such as:

let x, y a couple of OTUs,

let d= minimal distance calculated between x and y (encoded in the minimal distance matrix), there is a path of length k between x and y, with: k < d

The minimal distance matrix is encoded directly in the data structure of the model generator:

/* minimal distance matrix lophotrochozoans taxD: */

DIST[0][0]=0;

DIST[1][0]=1; DIST[1][1]=0;

DIST[2][0]=2; DIST[2][1]=3; DIST[2][2]=0;

DIST[3][0]=7; DIST[3][1]=7; DIST[3][2]=7; DIST[3][3]=0;

DIST[4][0]=6; DIST[4][1]=6; DIST[4][2]=6; DIST[4][3]=1; DIST[4][4]=0;

DIST[5][0]=7; DIST[5][1]=7; DIST[5][2]=7; DIST[5][3]=1; DIST[5][4]=1; DIST[5][5]=0;

DIST[6][0]=2; DIST[6][1]=3; DIST[6][2]=2; DIST[6][3]=6; DIST[6][4]=6; DIST[6][5]=6; DIST[6][6]=0;

DIST[7][0]=3; DIST[7][1]=4; DIST[7][2]=3; DIST[7][3]=7; DIST[7][4]=6; DIST[7][5]=7; DIST[7][6]=2; DIST[7][7]=0;

}

{ PROPERTY P5: graph T respects eventual Primary Phylogenetic Hypotheses }

{

This property is verified by constraints programmed in the model generator:

- monophyly of Lophotrochozoa = (0,1,2,3,4,5)

- monophyly of Mollusca = (0,1,2,3,4,5)

- monophyly of Polyplacophora = (0)

- monophyly of Cephalopoda = (0,1,2)

- monophyly of Gastropoda = (0,3,4,5)

Notes:

1- katharina_tunicata mtDNA is identical to octopus_vulgaris mtDNA (mollusc *Cephalopoda*), considering only the 15 protein-coding genes and rRNA genes. Thus, katharina_tunicata must be also element of Cephalopoda clade in this computation.

2- katharina_tunicata mtDNA is also identical to haliotis_rubra mtDNA (mollusc Gastropoda). Thus, katharina_tunicata must be also element of Gastropoda clade in this computation.

}

{------------------------------------------------------------------------------------------------------------------------}

{ PROPERTY P6: it is possible to calculate all the values for each HTU in the graph T }

{

First we calculate with the model generator the set of tree solutions which verify properties P1 to P5. Property P6 is verified *a posteriori* for each tree solution, with a *feedback* mechanism:

Studying each tree solution for calculating the values of HTUs, we eventually discover "impossible sub-trees": they appear in tree solutions which verify P1 to P5, but they do not verify P6.

For each impossible subtree A, an additional constraint is programmed into the model generator to forbid the solutions containing A. Tree solutions are recalculated and verified, allowing the discovery of new impossible subtrees and the programming of new constraints to recalculate the solutions (feedback mechanism). Finally, the complete set of optimal solutions is determined after iteration of this process and elimination of all the solutions that do not verify P6.

}

================================================================================

================================================================================

SOLUTIONS

================================================================================

================================================================================

OTUs:

katharina_tunicata = 0;

nautilus_macromphallus = 1;

loligo_bleekeri = 2;

albinaria_soerulea = 3;

cepaea_nemoralis = 4;

biomphalaria_glabrata = 5;

limulus_polyphemus = 6; { = outgroup1 }

homo_sapiens = 7; { = outgroup2 }

HTUs:

n1, n2, n3, n4, n5, n6, n7, n8

D = [0,15]: 6 solutions OK (which verify property P6) (1 impossible sub-tree)

minimal score (best) = 37

maximal score = 42

-------------------------------------------------------------------------------------------------------------

-> form outgroup SOL1_ALTER

n7(mod1)

[ cox1 cox2 atp8 atp6 cox3 nad3 -nad5 -nad4 -nad4L nad6 cob rrnS rrnL nad1 nad2 ]

score = 42:

model 1:

-------------

R(0,1) R(0,n1) R(0,n2) R(0,n8) R(1,0) R(2,n8) R(3,4) R(3,5) R(4,3) R(4,n5) R(5,3) R(6,n7) R(7,n7) R(n1,0) R(n1,n7) R(n2,0) R(n2,n3) R(n3,n2) R(n3,n4) R(n4,n3) R(n4,n6) R(n5,4) R(n5,n6) R(n6,n4) R(n6,n5) R(n7,6) R(n7,7) R(n7,n1) R(n8,0) R(n8,2)

-------------------------------------------------------------------------------------------------------------

-> the best model (1/2)

-> form outgroup SOL1_ALTER

n7(mod2)

[ cox1 cox2 atp8 atp6 cox3 nad3 -nad5 -nad4 -nad4L nad6 cob rrnS rrnL nad1 nad2 ]

score = 37:

model 2:

-------------

R(0,1) R(0,n1) R(0,n2) R(0,n8) R(1,0) R(2,n8) R(3,4) R(4,3) R(4,5) R(4,n4) R(5,4) R(6,n7) R(7,n7) R(n1,0) R(n1,n7) R(n2,0) R(n2,n3) R(n3,n2) R(n3,n5) R(n4,4) R(n4,n6) R(n5,n3) R(n5,n6) R(n6,n4) R(n6,n5) R(n7,6) R(n7,7) R(n7,n1) R(n8,0) R(n8,2)

-------------------------------------------------------------------------------------------------------------

-> form outgroup SOL1_ALTER

n7(mod3)

[ cox1 cox2 atp8 atp6 cox3 nad3 -nad5 -nad4 -nad4L nad6 cob rrnS rrnL nad1 nad2 ]

score = 42:

model 3:

-------------

R(0,1) R(0,n1) R(0,n2) R(0,n8) R(1,0) R(2,n8) R(3,5) R(4,5) R(4,n4) R(5,3) R(5,4) R(6,n7) R(7,n7) R(n1,0) R(n1,n7) R(n2,0) R(n2,n3) R(n3,n2) R(n3,n5) R(n4,4) R(n4,n6) R(n5,n3) R(n5,n6) R(n6,n4) R(n6,n5) R(n7,6) R(n7,7) R(n7,n1) R(n8,0) R(n8,2)

-------------------------------------------------------------------------------------------------------------

-> form outgroup SOL2_ALTER

score = 42:

model 4:

-------------

R(0,1) R(0,n1) R(0,n2) R(0,n8) R(1,0) R(2,n8) R(3,4) R(3,5) R(4,3) R(4,n3) R(5,3) R(6,n1) R(6,n7) R(7,n7) R(n1,0) R(n1,6) R(n2,0) R(n2,n6) R(n3,4) R(n3,n4) R(n4,n3) R(n4,n5) R(n5,n4) R(n5,n6) R(n6,n2) R(n6,n5) R(n7,6) R(n7,7) R(n8,0) R(n8,2)

-------------------------------------------------------------------------------------------------------------

-> the best model (2/2)

-> form outgroup SOL2_ALTER

score = 37:

model 5:

-------------

R(0,1) R(0,n1) R(0,n2) R(0,n8) R(1,0) R(2,n8) R(3,4) R(4,3) R(4,5) R(4,n3) R(5,4) R(6,n2) R(6,n7) R(7,n7) R(n1,0) R(n1,n6) R(n2,0) R(n2,6) R(n3,4) R(n3,n4) R(n4,n3) R(n4,n5) R(n5,n4) R(n5,n6) R(n6,n1) R(n6,n5) R(n7,6) R(n7,7) R(n8,0) R(n8,2)

-------------------------------------------------------------------------------------------------------------

-> form outgroup SOL2_ALTER

score = 42:

model 6:

-------------

R(0,1) R(0,n1) R(0,n2) R(0,n8) R(1,0) R(2,n8) R(3,5) R(4,5) R(4,n3) R(5,3) R(5,4) R(6,n2) R(6,n7) R(7,n7) R(n1,0) R(n1,n6) R(n2,0) R(n2,6) R(n3,4) R(n3,n4) R(n4,n3) R(n4,n5) R(n5,n4) R(n5,n6) R(n6,n1) R(n6,n5) R(n7,6) R(n7,7) R(n8,0) R(n8,2)

-------------------------------------------------------------------------------------------------------------

-> no other solutions

lophotrochozoans_taxE1_3sol

================================================================================

================================================================================

AXIOMS

================================================================================

================================================================================

{ the solutions of problem PHYLO are the smallest graphs T (defined on the smallest domain possible but containing at least all the OTUs) which verify properties P1 to P6:

P1- T is simple (the relation R(x, y) which defines graph T is not reflexive)

P2- T is non-oriented (the relation R(x, y) which defines graph T is symetrical)

P3- T is connected and acyclic (T is a tree)

P4- T respects the minimal distance matrix, i.e.:

for all couple of OTUs x and y, the length of the path x->y in T is always superior or equals to the minimal distance calculated between x and y (encoded in the minimal distance matrix)

P5- T respects other eventual hypothesis (Primary Phylogenetic Hypothesis = PPH)

used to impose the existence of given monophyletic groups

P6- it is possible to calculate all the values for each HTU in the graph T }

{ OTUs: }

katharina_tunicata = 0;

nautilus_macromphallus = 1;

loligo_bleekeri = 2;

platynereis_dumerilii = 3;

urechis_caupo = 4;

sipunculus_nudus = 5;

priapulus_caudatus = 6; { = outgroup1 }

homo_sapiens = 7; { = outgroup2 }

loxocorone_allax = 8;

terebratulina_retusa = 9;

phoronis_architecta = 10;

{ AUXILLIARY CONSTANTS used to fix a part of the solution: }

G1=11; { used to fix CASE 1 }

G2=12; { used to fix Cephalopoda group }

{ CASE 1 IS FIXED: 2 edges between homo_sapiens and priapulus_caudatus - first form }

R(homo_sapiens,G1);

Q x ( x<> G1

=>

-R(homo_sapiens,x)

);

R(priapulus_caudatus,G1);

Q x ( x<> G1

=>

-R(priapulus_caudatus,x)

);

{ THE CEPHALOPODA GROUP IS FIXED: }

R(nautilus_macromphallus,katharina_tunicata);

Q x ( x<> katharina_tunicata

=>

-R(nautilus_macromphallus,x)

);

R(G2,katharina_tunicata);

R(G2,loligo_bleekeri);

Q x ( ( x<> katharina_tunicata et

x<> loligo_bleekeri

)

=>

-R(G2,x)

);

Q x ( x<> G2

=>

-R(loligo_bleekeri,x)

);

{ PROPERTY P1: R(x, y) is not reflexive}

Q x (-R(x, x));

{ PROPERTY P2: R(x, y) is symetrical}

Q x y (R(x, y) => R(y, x));

{ PROPERTY P3: graph T is connected and acyclic (T is a tree) }

{

This property is verified by a constraint programmed in the model generator, instead of a "heavy" logical formula:

1- it will refuse the partial interpretations in which a connected component of the graph (in construction) is cyclic, i.e. such as: number of edges >= number of vertices

2- it will refuse the complete interpretations in which the constructed graph has more than one connected component

}

{ PROPERTY P4: graph T respects minimal distance matrix }

{

This property is verified by a constraint programmed in the model generator:

it will refuse the partial interpretations in which the graph (in construction) do not respect the minimal distance matrix, i.e. such as:

let x, y a couple of OTUs,

let d= minimal distance calculated between x and y (encoded in the minimal distance matrix), there is a path of length k between x and y, with: k < d

The minimal distance matrix is encoded directly in the data structure of the model generator:

/* minimal distance matrix lophotrochozoans taxE: */

DIST[0][0]=0;

DIST[1][0]=1; DIST[1][1]=0;

DIST[2][0]=2; DIST[2][1]=3; DIST[2][2]=0;

DIST[3][0]=3; DIST[3][1]=4; DIST[3][2]=4; DIST[3][3]=0;

DIST[4][0]=5; DIST[4][1]=5; DIST[4][2]=5; DIST[4][3]=3; DIST[4][4]=0;

DIST[5][0]=4; DIST[5][1]=4; DIST[5][2]=5; DIST[5][3]=3; DIST[5][4]=4; DIST[5][5]=0;

DIST[6][0]=2; DIST[6][1]=3; DIST[6][2]=2; DIST[6][3]=4; DIST[6][4]=4; DIST[6][5]=4; DIST[6][6]=0;

DIST[7][0]=3; DIST[7][1]=4; DIST[7][2]=3; DIST[7][3]=4; DIST[7][4]=5; DIST[7][5]=4; DIST[7][6]=2; DIST[7][7]=0;

DIST[8][0]=2; DIST[8][1]=2; DIST[8][2]=4; DIST[8][3]=5; DIST[8][4]=5; DIST[8][5]=5; DIST[8][6]=4; DIST[8][7]=4; DIST[8][8]=0;

DIST[9][0]=1; DIST[9][1]=2; DIST[9][2]=2; DIST[9][3]=3; DIST[9][4]=4; DIST[9][5]=5; DIST[9][6]=2; DIST[9][7]=3; DIST[9][8]=3; DIST[9][9]=0;

DIST[10][0]=1; DIST[10][1]=2; DIST[10][2]=3; DIST[10][3]=4; DIST[10][4]=5; DIST[10][5]=4; DIST[10][6]=3; DIST[10][7]=4; DIST[10][8]=3; DIST[10][9]=2; DIST[10][10]=0;

}

{ PROPERTY P5: graph T respects eventual Primary Phylogenetic Hypotheses }

{

This property is verified by constraints programmed in the model generator:

- monophyly of Lophotrochozoa = (0,1,2,3,4,5,8,9,10)

- monophyly of Eutrochozoa = (0,1,2,3,4,5,8)

- monophyly of Mollusca = (0,1,2)

- monophyly of Polyplacophora = (0)

- monophyly of Cephalopoda = (0,1,2)

- monophyly of Lophophorata = (9,10)

- monophyly of Annelida = (3,4)

- monophyly of Echiura = (3,4)

- monophyly of Polycheta = (3)

Notes:

1- katharina_tunicata mtDNA is identical to octopus_vulgaris mtDNA (mollusc *Cephalopoda*), considering only the 15 protein-coding genes and rRNA genes. Thus, katharina_tunicata must be also element of Cephalopoda clade in this computation.

2- platynereis_dumerilii mtDNA is identical to clymenella_torquata mtDNA (annelid *Echiura*). Thus, platynereis_dumerilii must be also element of *Echiura* clade in this computation.

}

{------------------------------------------------------------------------------------------------------------------------}

{ PROPERTY P6: it is possible to calculate all the values for each HTU in the graph T }

{

First we calculate with the model generator the set of tree solutions which verify properties P1 to P5. Property P6 is verified *a posteriori* for each tree solution, with a *feedback* mechanism:

Studying each tree solution for calculating the values of HTUs, we eventually discover "impossible sub-trees": they appear in tree solutions which verify P1 to P5, but they do not verify P6.

For each impossible subtree A, an additional constraint is programmed into the model generator to forbid the solutions containing A. Tree solutions are recalculated and verified, allowing the discovery of new impossible subtrees and the programming of new constraints to recalculate the solutions (feedback mechanism). Finally, the complete set of optimal solutions is determined after iteration of this process and elimination of all the solutions that do not verify P6.

}

================================================================================

================================================================================

SOLUTIONS

================================================================================

================================================================================

OTUs:

katharina_tunicata = 0;

nautilus_macromphallus = 1;

loligo_bleekeri = 2;

platynereis_dumerilii = 3;

urechis_caupo = 4;

sipunculus_nudus = 5;

priapulus_caudatus = 6; { = outgroup1 }

homo_sapiens = 7; { = outgroup2 }

loxocorone_allax = 8;

terebratulina_retusa = 9;

phoronis_architecta = 10;

AUXILLIARY CONSTANTS used to fix a part of the solution:

G1=11; { between homo_sapiens and priapulus_caudatus }

G2=12; { between katharina_tunicata and loligo_bleekeri }

HTUs:

n1, n2, n3, n4, n5, n6, n7, n8

D = [0,20]: 3 solutions OK (which verify property P6) (99 impossible sub-trees)

minimal score (best) = 66

maximal score = 80

-------------------------------------------------------------------------------------------------------------

score = 72:

model 1:

-------------

R(0,1) R(0,9) R(0,10) R(0,12) R(0,n1) R(0,n2) R(0,n3) R(1,0) R(2,12) R(3,n4) R(3,n5) R(3,n6) R(4,n7) R(5,n8) R(6,11) R(7,11) R(8,n3) R(9,0) R(10,0) R(11,6) R(11,7) R(11,n1) R(12,0) R(12,2) R(n1,0) R(n1,11) R(n2,0) R(n2,n6) R(n3,0) R(n3,8) R(n4,3) R(n4,n7) R(n5,3) R(n5,n8) R(n6,3) R(n6,n2) R(n7,4) R(n7,n4) R(n8,5) R(n8,n5)

-------------------------------------------------------------------------------------------------------------

-> the best model

score = 66:

model 2:

-------------

R(0,1) R(0,9) R(0,10) R(0,12) R(0,n1) R(0,n2) R(0,n3) R(1,0) R(2,12) R(3,n4) R(3,n5) R(4,n6) R(5,n7) R(6,11) R(7,11) R(8,n1) R(9,0) R(10,0) R(11,6) R(11,7) R(11,n3) R(12,0) R(12,2) R(n1,0) R(n1,8) R(n2,0) R(n2,n5) R(n3,0) R(n3,11) R(n4,3) R(n4,n6) R(n5,3) R(n5,n2) R(n5,n8) R(n6,4) R(n6,n4) R(n7,5) R(n7,n8) R(n8,n5) R(n8,n7)

-------------------------------------------------------------------------------------------------------------

score = 80:

model 3:

-------------

R(0,1) R(0,9) R(0,10) R(0,12) R(0,n1) R(0,n2) R(0,n3) R(1,0) R(2,12) R(3,n4) R(3,n5) R(4,n6) R(5,n7) R(6,11) R(7,11) R(8,n1) R(9,0) R(10,0) R(11,6) R(11,7) R(11,n3) R(12,0) R(12,2) R(n1,0) R(n1,8) R(n2,0) R(n2,n8) R(n3,0) R(n3,11) R(n4,3) R(n4,n6) R(n5,3) R(n5,n7) R(n5,n8) R(n6,4) R(n6,n4) R(n7,5) R(n7,n5) R(n8,n2) R(n8,n5)

-------------------------------------------------------------------------------------------------------------

-> no other solutions

lophotrochozoans_taxE2_3sol

================================================================================

================================================================================

AXIOMS

================================================================================

================================================================================

{ the solutions of problem PHYLO are the smallest graphs T (defined on the smallest domain possible but containing at least all the OTUs) which verify properties P1 to P6:

P1- T is simple (the relation R(x, y) which defines graph T is not reflexive)

P2- T is non-oriented (the relation R(x, y) which defines graph T is symetrical)

P3- T is connected and acyclic (T is a tree)

P4- T respects the minimal distance matrix, i.e.:

for all couple of OTUs x and y, the length of the path x->y in T is always superior or equals to the minimal distance calculated between x and y (encoded in the minimal distance matrix)

P5- T respects other eventual hypothesis (Primary Phylogenetic Hypothesis = PPH)

used to impose the existence of given monophyletic groups

P6- it is possible to calculate all the values for each HTU in the graph T }

{ OTUs: }

katharina_tunicata = 0;

nautilus_macromphallus = 1;

loligo_bleekeri = 2;

platynereis_dumerilii = 3;

urechis_caupo = 4;

sipunculus_nudus = 5;

priapulus_caudatus = 6; { = outgroup1 }

homo_sapiens = 7; { = outgroup2 }

loxocorone_allax = 8;

terebratulina_retusa = 9;

phoronis_architecta = 10;

{ AUXILLIARY CONSTANTS used to fix a part of the solution: }

G1=11; { used to fix CASE 2 }

G2=12; { used to fix Cephalopoda }

{ CASE 2 IS FIXED: 2 edges between homo_sapiens and priapulus_caudatus - second form }

R(homo_sapiens,G1);

Q x ( x<> G1

=>

-R(homo_sapiens,x)

);

R(G1,priapulus_caudatus);

Q x ( ( x<>priapulus_caudatus et

x<>homo_sapiens

)

=>

-R(G1,x)

);

{ THE CEPHALOPODA GROUP IS FIXED: }

R(nautilus_macromphallus,katharina_tunicata);

Q x ( x<> katharina_tunicata

=>

-R(nautilus_macromphallus,x)

);

R(G2,katharina_tunicata);

R(G2,loligo_bleekeri);

Q x ( ( x<> katharina_tunicata et

x<> loligo_bleekeri

)

=>

-R(G2,x)

);

Q x ( x<> G2

=>

-R(loligo_bleekeri,x)

);

{ PROPERTY P1: R(x, y) is not reflexive}

Q x (-R(x, x));

{ PROPERTY P2: R(x, y) is symetrical}

Q x y (R(x, y) => R(y, x));

{ PROPERTY P3: graph T is connected and acyclic (T is a tree) }

{

This property is verified by a constraint programmed in the model generator, instead of a "heavy" logical formula:

1- it will refuse the partial interpretations in which a connected component of the graph (in construction) is cyclic, i.e. such as: number of edges >= number of vertices

2- it will refuse the complete interpretations in which the constructed graph has more than one connected component

}

{ PROPERTY P4: graph T respects minimal distance matrix }

{

This property is verified by a constraint programmed in the model generator:

it will refuse the partial interpretations in which the graph (in construction) do not respect the minimal distance matrix, i.e. such as:

let x, y a couple of OTUs,

let d= minimal distance calculated between x and y (encoded in the minimal distance matrix), there is a path of length k between x and y, with: k < d

The minimal distance matrix is encoded directly in the data structure of the model generator:

/* minimal distance matrix lophotrochozoans taxE: */

DIST[0][0]=0;

DIST[1][0]=1; DIST[1][1]=0;

DIST[2][0]=2; DIST[2][1]=3; DIST[2][2]=0;

DIST[3][0]=3; DIST[3][1]=4; DIST[3][2]=4; DIST[3][3]=0;

DIST[4][0]=5; DIST[4][1]=5; DIST[4][2]=5; DIST[4][3]=3; DIST[4][4]=0;

DIST[5][0]=4; DIST[5][1]=4; DIST[5][2]=5; DIST[5][3]=3; DIST[5][4]=4; DIST[5][5]=0;

DIST[6][0]=2; DIST[6][1]=3; DIST[6][2]=2; DIST[6][3]=4; DIST[6][4]=4; DIST[6][5]=4; DIST[6][6]=0;

DIST[7][0]=3; DIST[7][1]=4; DIST[7][2]=3; DIST[7][3]=4; DIST[7][4]=5; DIST[7][5]=4; DIST[7][6]=2; DIST[7][7]=0;

DIST[8][0]=2; DIST[8][1]=2; DIST[8][2]=4; DIST[8][3]=5; DIST[8][4]=5; DIST[8][5]=5; DIST[8][6]=4; DIST[8][7]=4; DIST[8][8]=0;

DIST[9][0]=1; DIST[9][1]=2; DIST[9][2]=2; DIST[9][3]=3; DIST[9][4]=4; DIST[9][5]=5; DIST[9][6]=2; DIST[9][7]=3; DIST[9][8]=3; DIST[9][9]=0;

DIST[10][0]=1; DIST[10][1]=2; DIST[10][2]=3; DIST[10][3]=4; DIST[10][4]=5; DIST[10][5]=4; DIST[10][6]=3; DIST[10][7]=4; DIST[10][8]=3; DIST[10][9]=2; DIST[10][10]=0;

}

{ PROPERTY P5: graph T respects eventual Primary Phylogenetic Hypotheses }

{

This property is verified by constraints programmed in the model generator:

- monophyly of Lophotrochozoa = (0,1,2,3,4,5,8,9,10)

- monophyly of Eutrochozoa = (0,1,2,3,4,5,8)

- monophyly of Mollusca = (0,1,2)

- monophyly of Polyplacophora = (0)

- monophyly of Cephalopoda = (0,1,2)

- monophyly of Lophophorata = (9,10)

- monophyly of Annelida = (3,4)

- monophyly of Echiura = (3,4)

- monophyly of Polychaeta = (3)

Notes:

1- katharina_tunicata mtDNA is identical to octopus_vulgaris mtDNA (mollusc *Cephalopoda*), considering only the 15 protein-coding genes and rRNA genes. Thus, katharina_tunicata must be also element of Cephalopoda clade in this computation.

2- platynereis_dumerilii mtDNA is identical to clymenella_torquata mtDNA (annelid *Echiura*). Thus, platynereis_dumerilii must be also element of *Echiura* clade in this computation.

}

{------------------------------------------------------------------------------------------------------------------------}

{ PROPERTY P6: it is possible to calculate all the values for each HTU in the graph T }

{

First we calculate with the model generator the set of tree solutions which verify properties P1 to P5. Property P6 is verified *a posteriori* for each tree solution, with a *feedback* mechanism:

Studying each tree solution for calculating the values of HTUs, we eventually discover "impossible sub-trees": they appear in tree solutions which verify P1 to P5, but they do not verify P6.

For each impossible subtree A, an additional constraint is programmed into the model generator to forbid the solutions containing A. Tree solutions are recalculated and verified, allowing the discovery of new impossible subtrees and the programming of new constraints to recalculate the solutions (feedback mechanism). Finally, the complete set of optimal solutions is determined after iteration of this process and elimination of all the solutions that do not verify P6.

}

================================================================================

================================================================================

SOLUTIONS

================================================================================

================================================================================

OTUs:

katharina_tunicata = 0;

nautilus_macromphallus = 1;

loligo_bleekeri = 2;

platynereis_dumerilii = 3;

urechis_caupo = 4;

sipunculus_nudus = 5;

priapulus_caudatus = 6; { = outgroup1 }

homo_sapiens = 7; { = outgroup2 }

loxocorone_allax = 8;

terebratulina_retusa = 9;

phoronis_architecta = 10;

AUXILLIARY CONSTANTS used to fix a part of the solution:

G1=11; { between homo_sapiens and priapulus_caudatus }

G2=12; { between katharina_tunicata and loligo_bleekeri }

HTUs:

n1, n2, n3, n4, n5, n6, n7, n8

D = [0,20]: 3 solutions OK (which verify property P6) (108 impossible sub-trees)

minimal score (best) = 66

maximal score = 80

-------------------------------------------------------------------------------------------------------------

score = 72:

model 1:

-------------

R(0,1) R(0,9) R(0,10) R(0,12) R(0,n1) R(0,n2) R(0,n3) R(1,0) R(2,12) R(3,n4) R(3,n5) R(3,n6) R(4,n7) R(5,n8) R(6,11) R(6,n1) R(7,11) R(8,n2) R(9,0) R(10,0) R(11,6) R(11,7) R(12,0) R(12,2) R(n1,0) R(n1,6) R(n2,0) R(n2,8) R(n3,0) R(n3,n6) R(n4,3) R(n4,n8) R(n5,3) R(n5,n7) R(n6,3) R(n6,n3) R(n7,4) R(n7,n5) R(n8,5) R(n8,n4)

-------------------------------------------------------------------------------------------------------------

-> the best model

score = 66:

model 2:

-------------

R(0,1) R(0,9) R(0,10) R(0,12) R(0,n1) R(0,n2) R(0,n3) R(1,0) R(2,12) R(3,n4) R(3,n5) R(4,n6) R(5,n7) R(6,11) R(6,n1) R(7,11) R(8,n3) R(9,0) R(10,0) R(11,6) R(11,7) R(12,0) R(12,2) R(n1,0) R(n1,6) R(n2,0) R(n2,n5) R(n3,0) R(n3,8) R(n4,3) R(n4,n6) R(n5,3) R(n5,n2) R(n5,n8) R(n6,4) R(n6,n4) R(n7,5) R(n7,n8) R(n8,n5) R(n8,n7)

-------------------------------------------------------------------------------------------------------------

score = 80:

model 3:

-------------

R(0,1) R(0,9) R(0,10) R(0,12) R(0,n1) R(0,n2) R(0,n3) R(1,0) R(2,12) R(3,n4) R(3,n5) R(4,n6) R(5,n7) R(6,11) R(6,n1) R(7,11) R(8,n3) R(9,0) R(10,0) R(11,6) R(11,7) R(12,0) R(12,2) R(n1,0) R(n1,6) R(n2,0) R(n2,n8) R(n3,0) R(n3,8) R(n4,3) R(n4,n6) R(n5,3) R(n5,n7) R(n5,n8) R(n6,4) R(n6,n4) R(n7,5) R(n7,n5) R(n8,n2) R(n8,n5)

-------------------------------------------------------------------------------------------------------------

-> no other solutions

lophotrochozoans_taxF1_9sol

================================================================================

================================================================================

AXIOMS

================================================================================

================================================================================

{ the solutions of problem PHYLO are the smallest graphs T (defined on the smallest domain possible but containing at least all the OTUs) which verify properties P1 to P6:

P1- T is simple (the relation R(x, y) which defines graph T is not reflexive)

P2- T is non-oriented (the relation R(x, y) which defines graph T is symetrical)

P3- T is connected and acyclic (T is a tree)

P4- T respects the minimal distance matrix, i.e.:

for all couple of OTUs x and y, the length of the path x->y in T is always superior or equals to the minimal distance calculated between x and y (encoded in the minimal distance matrix)

P5- T respects other eventual hypothesis (Primary Phylogenetic Hypothesis = PPH)

used to impose the existence of given monophyletic groups

P6- it is possible to calculate all the values for each HTU in the graph T }

{ OTUs: }

katharina_tunicata = 0;

nautilus_macromphallus = 1;

loligo_bleekeri = 2;

platynereis_dumerilii = 3;

urechis_caupo = 4;

sipunculus_nudus = 5;

priapulus_caudatus = 6; { = outgroup1 }

homo_sapiens = 7; { = outgroup2 }

loxocorone_allax = 8;

terebratulina_retusa = 9;

phoronis_architecta = 10;

bugula_neritina=11;

terebratalia_transversa=12;

{ AUXILLIARY CONSTANTS used to fix a part of the solution: }

G1=13; G2=14; G3=15;

G4=16; G5=17; G6=18;

G7=19; G8=20; { used to fix the Eutrochozoa group }

G9=21; { used to fix CASE 1 }

{ THE EUTROCHOZOA GROUP IS FIXED: }

{ OTUs (0,1,2): }

R(katharina_tunicata,nautilus_macromphallus);

R(katharina_tunicata,G1);

R(G1,loligo_bleekeri);

Q x ( x<>katharina_tunicata

=>

-R(nautilus_macromphallus,x)

);

Q x ( ( x<>katharina_tunicata et

x<>loligo_bleekeri

)

=>

-R(G1,x)

);

Q x ( x<>G1

=>

-R(loligo_bleekeri,x)

);

{ OTUs (3,4,5,8): }

{ as in the best of the 3 possible forms }

R(katharina_tunicata,G2);

R(G2,G3);

R(G3,platynereis_dumerilii);

R(platynereis_dumerilii,G4);

R(G4,G5);

R(G5,urechis_caupo);

R(G3,G6);

R(G6,G7);

R(G7,sipunculus_nudus);

Q x ( ( x<>katharina_tunicata et

x<>G3

)

=>

-R(G2,x)

);

Q x ( ( x<>G2 et

x<>platynereis_dumerilii et

x<>G6

)

=>

-R(G3,x)

);

Q x ( ( x<>platynereis_dumerilii et

x<>G5

)

=>

-R(G4,x)

);

Q x ( ( x<>G3 et

x<>G4

)

=>

-R(platynereis_dumerilii,x)

);

Q x ( ( x<>G4 et

x<>urechis_caupo

)

=>

-R(G5,x)

);

Q x ( x<>G5

=>

-R(urechis_caupo,x)

);

Q x ( ( x<>G3 et

x<>G7

)

=>

-R(G6,x)

);

Q x ( ( x<>G6 et

x<>sipunculus_nudus

)

=>

-R(G7,x)

);

Q x ( x<>G7

=>

-R(sipunculus_nudus,x)

);

{ OTU 8: }

R(katharina_tunicata,G8);

R(G8,loxocorone_allax);

Q x ( ( x<>katharina_tunicata et

x<>loxocorone_allax

)

=>

-R(G8,x)

);

Q x ( x<>G8

=>

-R(loxocorone_allax, x)

);

{ CASE 1 IS FIXED: 2 edges between homo_sapiens and priapulus_caudatus - first form }

R(homo_sapiens,G9);

Q x ( x<> G9

=>

-R(homo_sapiens,x)

);

R(priapulus_caudatus,G9);

Q x ( x<> G9

=>

-R(priapulus_caudatus,x)

);

{ PROPERTY P1: R(x, y) is not reflexive}

Q x (-R(x, x));

{ PROPERTY P2: R(x, y) is symetrical}

Q x y (R(x, y) => R(y, x));

{ PROPERTY P3: graph T is connected and acyclic (T is a tree) }

{

This property is verified by a constraint programmed in the model generator, instead of a "heavy" logical formula:

1- it will refuse the partial interpretations in which a connected component of the graph (in construction) is cyclic, i.e. such as: number of edges >= number of vertices

2- it will refuse the complete interpretations in which the constructed graph has more than one connected component

}

{ PROPERTY P4: graph T respects minimal distance matrix }

{

This property is verified by a constraint programmed in the model generator:

it will refuse the partial interpretations in which the graph (in construction) do not respect the minimal distance matrix, i.e. such as:

let x, y a couple of OTUs,

let d= minimal distance calculated between x and y (encoded in the minimal distance matrix), there is a path of length k between x and y, with: k < d

The minimal distance matrix is encoded directly in the data structure of the model generator:

/* minimal distance matrix lophotrochozoans taxF: */

DIST[0][0]=0;

DIST[1][0]=1; DIST[1][1]=0;

DIST[2][0]=2; DIST[2][1]=3; DIST[2][2]=0;

DIST[3][0]=3; DIST[3][1]=4; DIST[3][2]=4; DIST[3][3]=0;

DIST[4][0]=5; DIST[4][1]=5; DIST[4][2]=5; DIST[4][3]=3; DIST[4][4]=0;

DIST[5][0]=4; DIST[5][1]=4; DIST[5][2]=5; DIST[5][3]=3; DIST[5][4]=4; DIST[5][5]=0;

DIST[6][0]=2; DIST[6][1]=3; DIST[6][2]=2; DIST[6][3]=4; DIST[6][4]=4; DIST[6][5]=4; DIST[6][6]=0;

DIST[7][0]=3; DIST[7][1]=4; DIST[7][2]=3; DIST[7][3]=4; DIST[7][4]=5; DIST[7][5]=4; DIST[7][6]=2; DIST[7][7]=0;

DIST[8][0]=2; DIST[8][1]=2; DIST[8][2]=4; DIST[8][3]=5; DIST[8][4]=5; DIST[8][5]=5; DIST[8][6]=4; DIST[8][7]=4; DIST[8][8]=0;

DIST[9][0]=1; DIST[9][1]=2; DIST[9][2]=2; DIST[9][3]=3; DIST[9][4]=4; DIST[9][5]=5; DIST[9][6]=2; DIST[9][7]=3; DIST[9][8]=3; DIST[9][9]=0;

DIST[10][0]=1; DIST[10][1]=2; DIST[10][2]=3; DIST[10][3]=4; DIST[10][4]=5; DIST[10][5]=4; DIST[10][6]=3; DIST[10][7]=4; DIST[10][8]=3; DIST[10][9]=2; DIST[10][10]=0;

DIST[11][0]=4; DIST[11][1]=5; DIST[11][2]=5; DIST[11][3]=6; DIST[11][4]=6; DIST[11][5]=6; DIST[11][6]=5; DIST[11][7]=5; DIST[11][8]=5; DIST[11][9]=4; DIST[11][10]=4; DIST[11][11]=0;

DIST[12][0]=5; DIST[12][1]=6; DIST[12][2]=6; DIST[12][3]=5; DIST[12][4]=7; DIST[12][5]=6; DIST[12][6]=6; DIST[12][7]=7; DIST[12][8]=7; DIST[12][9]=5; DIST[12][10]=5; DIST[12][11]=7; DIST[12][12]=0;

}

{ PROPERTY P5: graph T respects eventual Primary Phylogenetic Hypotheses }

{

This property is verified by constraints programmed in the model generator:

- monophyly of Lophotrochozoa = (0,1,2,3,4,5,8,9,10,11,12)

- monophyly of Eutrochozoa = (0,1,2,3,4,5,8)

- monophyly of Mollusca = (0,1,2)

- monophyly of Polyplacophora = (0)

- monophyly of Cephalopoda = (0,1,2)

- monophyly of Annelida = (3,4)

- monophyly of Echiura = (3,4)

- monophyly of Polychaeta = (3)

- monophyly of Lophophorata = (9,10,11,12)

- monophyly of Brachiopoda = (9,12)

Notes:

1- katharina_tunicata mtDNA is identical to octopus_vulgaris mtDNA (mollusc *Cephalopoda*), considering only the 15 protein-coding genes and rRNA genes. Thus, katharina_tunicata must be also element of Cephalopoda clade in this computation.

2- platynereis_dumerilii mtDNA is identical to clymenella_torquata mtDNA (annelid *Echiura*). Thus, platynereis_dumerilii must be also element of *Echiura* clade in this computation.

}

{------------------------------------------------------------------------------------------------------------------------}

{ PROPERTY P6: it is possible to calculate all the values for each HTU in the graph T }

{

First we calculate with the model generator the set of tree solutions which verify properties P1 to P5. Property P6 is verified *a posteriori* for each tree solution, with a *feedback* mechanism:

Studying each tree solution for calculating the values of HTUs, we eventually discover "impossible sub-trees": they appear in tree solutions which verify P1 to P5, but they do not verify P6.

For each impossible subtree A, an additional constraint is programmed into the model generator to forbid the solutions containing A. Tree solutions are recalculated and verified, allowing the discovery of new impossible subtrees and the programming of new constraints to recalculate the solutions (feedback mechanism). Finally, the complete set of optimal solutions is determined after iteration of this process and elimination of all the solutions that do not verify P6.

}

================================================================================

================================================================================

SOLUTIONS

================================================================================

================================================================================

OTUs:

katharina_tunicata = 0;

nautilus_macromphallus = 1;

loligo_bleekeri = 2;

platynereis_dumerilii = 3;

urechis_caupo = 4;

sipunculus_nudus = 5;

priapulus_caudatus = 6; { = outgroup1 }

homo_sapiens = 7; { = outgroup2 }

loxocorone_allax = 8;

terebratulina_retusa = 9;

phoronis_architecta = 10;

bugula_neritina=11;

terebratalia_transversa=12;

AUXILLIARY CONSTANTS used to fix a part of the solution:

G1=13; { used to fix Cephalopoda group }

G2=14; G3=15; G4=16; G5=17;

G6=18; G7=19; G8=20; { used to fix the rest of Eutrochozoa group }

G9=21; { used to fix CASE 1 }

HTUs:

n1, n2, n3, n4, n5, n6, n7, n8

D = [0,29]: 9 solutions OK (which verify property P6) (128 impossible sub-trees)

minimal score (best) = 101

maximal score = 135

-------------------------------------------------------------------------------------------------------------

-> the best model

score = 101:

model 1:

-------------

R(0,1) R(0,9) R(0,10) R(0,13) R(0,14) R(0,20) R(0,n1) R(0,n2) R(0,n3) R(1,0) R(2,13) R(3,15) R(3,16) R(4,17) R(5,19) R(6,21) R(7,21) R(8,20) R(9,0) R(10,0) R(11,n4) R(12,n6) R(13,0) R(13,2) R(14,0) R(14,15) R(15,3) R(15,14) R(15,18) R(16,3) R(16,17) R(17,4) R(17,16) R(18,15) R(18,19) R(19,5) R(19,18) R(20,0) R(20,8) R(21,6) R(21,7) R(21,n1) R(n1,0) R(n1,21) R(n2,0) R(n2,n5) R(n3,0) R(n3,n7) R(n4,11) R(n4,n5) R(n5,n2) R(n5,n4) R(n6,12) R(n6,n8) R(n7,n3) R(n7,n8) R(n8,n6) R(n8,n7)

-------------------------------------------------------------------------------------------------------------

score = 111:

model 2:

-------------

R(0,1) R(0,10) R(0,13) R(0,14) R(0,20) R(0,n1) R(0,n2) R(0,n3) R(1,0) R(2,13) R(3,15) R(3,16) R(4,17) R(5,19) R(6,21) R(7,21) R(8,20) R(9,n1) R(10,0) R(11,n4) R(12,n7) R(13,0) R(13,2) R(14,0) R(14,15) R(15,3) R(15,14) R(15,18) R(16,3) R(16,17) R(17,4) R(17,16) R(18,15) R(18,19) R(19,5) R(19,18) R(20,0) R(20,8) R(21,6) R(21,7) R(21,n3) R(n1,0) R(n1,9) R(n1,n6) R(n2,0) R(n2,n5) R(n3,0) R(n3,21) R(n4,11) R(n4,n5) R(n5,n2) R(n5,n4) R(n6,n1) R(n6,n8) R(n7,12) R(n7,n8) R(n8,n6) R(n8,n7)

-------------------------------------------------------------------------------------------------------------

score = 111:

model 3:

-------------

R(0,1) R(0,9) R(0,10) R(0,13) R(0,14) R(0,20) R(0,n1) R(0,n2) R(1,0) R(2,13) R(3,15) R(3,16) R(4,17) R(5,19) R(6,21) R(7,21) R(8,20) R(9,0) R(9,n3) R(10,0) R(11,n7) R(12,n6) R(13,0) R(13,2) R(14,0) R(14,15) R(15,3) R(15,14) R(15,18) R(16,3) R(16,17) R(17,4) R(17,16) R(18,15) R(18,19) R(19,5) R(19,18) R(20,0) R(20,8) R(21,6) R(21,7) R(21,n2) R(n1,0) R(n1,n8) R(n2,0) R(n2,21) R(n3,9) R(n3,n4) R(n4,n3) R(n4,n5) R(n5,n4) R(n5,n6) R(n6,12) R(n6,n5) R(n7,11) R(n7,n8) R(n8,n1) R(n8,n7)

-------------------------------------------------------------------------------------------------------------

score = 129:

model 4:

-------------

R(0,1) R(0,10) R(0,13) R(0,14) R(0,20) R(0,n1) R(0,n2) R(1,0) R(2,13) R(3,15) R(3,16) R(4,17) R(5,19) R(6,21) R(7,21) R(8,20) R(9,n3) R(10,0) R(10,n3) R(11,n7) R(12,n6) R(13,0) R(13,2) R(14,0) R(14,15) R(15,3) R(15,14) R(15,18) R(16,3) R(16,17) R(17,4) R(17,16) R(18,15) R(18,19) R(19,5) R(19,18) R(20,0) R(20,8) R(21,6) R(21,7) R(21,n2) R(n1,0) R(n1,n8) R(n2,0) R(n2,21) R(n3,9) R(n3,10) R(n3,n4) R(n4,n3) R(n4,n5) R(n5,n4) R(n5,n6) R(n6,12) R(n6,n5) R(n7,11) R(n7,n8) R(n8,n1) R(n8,n7)

-------------------------------------------------------------------------------------------------------------

score = 111:

model 5:

-------------

R(0,1) R(0,9) R(0,10) R(0,13) R(0,14) R(0,20) R(0,n1) R(0,n2) R(1,0) R(2,13) R(3,15) R(3,16) R(4,17) R(5,19) R(6,21) R(7,21) R(8,20) R(9,0) R(10,0) R(10,n3) R(11,n5) R(12,n6) R(13,0) R(13,2) R(14,0) R(14,15) R(15,3) R(15,14) R(15,18) R(16,3) R(16,17) R(17,4) R(17,16) R(18,15) R(18,19) R(19,5) R(19,18) R(20,0) R(20,8) R(21,6) R(21,7) R(21,n2) R(n1,0) R(n1,n8) R(n2,0) R(n2,21) R(n3,10) R(n3,n4) R(n4,n3) R(n4,n5) R(n5,11) R(n5,n4) R(n6,12) R(n6,n7) R(n7,n6) R(n7,n8) R(n8,n1) R(n8,n7)

-------------------------------------------------------------------------------------------------------------

score = 121:

model 6:

-------------

R(0,1) R(0,10) R(0,13) R(0,14) R(0,20) R(0,n1) R(0,n2) R(1,0) R(2,13) R(3,15) R(3,16) R(4,17) R(5,19) R(6,21) R(7,21) R(8,20) R(9,n1) R(10,0) R(10,n3) R(11,n5) R(12,n6) R(13,0) R(13,2) R(14,0) R(14,15) R(15,3) R(15,14) R(15,18) R(16,3) R(16,17) R(17,4) R(17,16) R(18,15) R(18,19) R(19,5) R(19,18) R(20,0) R(20,8) R(21,6) R(21,7) R(21,n2) R(n1,0) R(n1,9) R(n1,n8) R(n2,0) R(n2,21) R(n3,10) R(n3,n4) R(n4,n3) R(n4,n5) R(n5,11) R(n5,n4) R(n6,12) R(n6,n7) R(n7,n6) R(n7,n8) R(n8,n1) R(n8,n7)

-------------------------------------------------------------------------------------------------------------

score = 119:

model 7:

-------------

R(0,1) R(0,9) R(0,10) R(0,13) R(0,14) R(0,20) R(0,n1) R(1,0) R(2,13) R(3,15) R(3,16) R(4,17) R(5,19) R(6,21) R(7,21) R(8,20) R(9,0) R(9,n2) R(9,n3) R(10,0) R(11,n4) R(12,n6) R(13,0) R(13,2) R(14,0) R(14,15) R(15,3) R(15,14) R(15,18) R(16,3) R(16,17) R(17,4) R(17,16) R(18,15) R(18,19) R(19,5) R(19,18) R(20,0) R(20,8) R(21,6) R(21,7) R(21,n1) R(n1,0) R(n1,21) R(n2,9) R(n2,n5) R(n3,9) R(n3,n8) R(n4,11) R(n4,n5) R(n5,n2) R(n5,n4) R(n6,12) R(n6,n7) R(n7,n6) R(n7,n8) R(n8,n3) R(n8,n7)

-------------------------------------------------------------------------------------------------------------

score = 121:

model 8:

-------------

R(0,1) R(0,9) R(0,10) R(0,13) R(0,14) R(0,20) R(0,n1) R(1,0) R(2,13) R(3,15) R(3,16) R(4,17) R(5,19) R(6,21) R(7,21) R(8,20) R(9,0) R(9,n2) R(10,0) R(10,n6) R(11,n7) R(12,n5) R(13,0) R(13,2) R(14,0) R(14,15) R(15,3) R(15,14) R(15,18) R(16,3) R(16,17) R(17,4) R(17,16) R(18,15) R(18,19) R(19,5) R(19,18) R(20,0) R(20,8) R(21,6) R(21,7) R(21,n1) R(n1,0) R(n1,21) R(n2,9) R(n2,n3) R(n3,n2) R(n3,n4) R(n4,n3) R(n4,n5) R(n5,12) R(n5,n4) R(n6,10) R(n6,n8) R(n7,11) R(n7,n8) R(n8,n6) R(n8,n7)

-------------------------------------------------------------------------------------------------------------

score = 135:

model 9:

-------------

R(0,1) R(0,10) R(0,13) R(0,14) R(0,20) R(0,n1) R(1,0) R(2,13) R(3,15) R(3,16) R(4,17) R(5,19) R(6,21) R(7,21) R(8,20) R(9,n2) R(10,0) R(10,n2) R(10,n6) R(11,n7) R(12,n5) R(13,0) R(13,2) R(14,0) R(14,15) R(15,3) R(15,14) R(15,18) R(16,3) R(16,17) R(17,4) R(17,16) R(18,15) R(18,19) R(19,5) R(19,18) R(20,0) R(20,8) R(21,6) R(21,7) R(21,n1) R(n1,0) R(n1,21) R(n2,9) R(n2,10) R(n2,n3) R(n3,n2) R(n3,n4) R(n4,n3) R(n4,n5) R(n5,12) R(n5,n4) R(n6,10) R(n6,n8) R(n7,11) R(n7,n8) R(n8,n6) R(n8,n7)

-------------------------------------------------------------------------------------------------------------

-> no other solutions

lophotrochozoans_taxF2_9sol

================================================================================

================================================================================

AXIOMS

================================================================================

================================================================================

{ the solutions of problem PHYLO are the smallest graphs T (defined on the smallest domain possible but containing at least all the OTUs) which verify properties P1 to P6:

P1- T is simple (the relation R(x, y) which defines graph T is not reflexive)

P2- T is non-oriented (the relation R(x, y) which defines graph T is symetrical)

P3- T is connected and acyclic (T is a tree)

P4- T respects the minimal distance matrix, i.e.:

for all couple of OTUs x and y, the length of the path x->y in T is always superior or equals to the minimal distance calculated between x and y (encoded in the minimal distance matrix)

P5- T respects other eventual hypothesis (Primary Phylogenetic Hypothesis = PPH)

used to impose the existence of given monophyletic groups

P6- it is possible to calculate all the values for each HTU in the graph T }

{ OTUs: }

katharina_tunicata = 0;

nautilus_macromphallus = 1;

loligo_bleekeri = 2;

platynereis_dumerilii = 3;

urechis_caupo = 4;

sipunculus_nudus = 5;

priapulus_caudatus = 6; { = outgroup1 }

homo_sapiens = 7; { = outgroup2 }

loxocorone_allax = 8;

terebratulina_retusa = 9;

phoronis_architecta = 10;

bugula_neritina=11;

terebratalia_transversa=12;

{ AUXILLIARY CONSTANTS used to fix a part of the solution: }

G1=13; G2=14; G3=15;

G4=16; G5=17; G6=18;

G7=19; G8=20; { used to fix the Eutrochozoa group }

G9=21; { used to fix CASE 2 }

{ THE EUTROCHOZOA GROUP IS FIXED: }

{ OTUs (0,1,2): }

R(katharina_tunicata,nautilus_macromphallus);

R(katharina_tunicata,G1);

R(G1,loligo_bleekeri);

Q x ( x<>katharina_tunicata

=>

-R(nautilus_macromphallus,x)

);

Q x ( ( x<>katharina_tunicata et

x<>loligo_bleekeri

)

=>

-R(G1,x)

);

Q x ( x<>G1

=>

-R(loligo_bleekeri,x)

);

{ OTUs (3,4,5): }

R(katharina_tunicata,G2);

R(G2,G3);

R(G3,platynereis_dumerilii);

R(platynereis_dumerilii,G4);

R(G4,G5);

R(G5,urechis_caupo);

R(G3,G6);

R(G6,G7);

R(G7,sipunculus_nudus);

Q x ( ( x<>katharina_tunicata et

x<>G3

)

=>

-R(G2,x)

);

Q x ( ( x<>G2 et

x<>platynereis_dumerilii et

x<>G6

)

=>

-R(G3,x)

);

Q x ( ( x<>platynereis_dumerilii et

x<>G5

)

=>

-R(G4,x)

);

Q x ( ( x<>G3 et

x<>G4

)

=>

-R(platynereis_dumerilii,x)

);

Q x ( ( x<>G4 et

x<>urechis_caupo

)

=>

-R(G5,x)

);

Q x ( x<>G5

=>

-R(urechis_caupo,x)

);

Q x ( ( x<>G3 et

x<>G7

)

=>

-R(G6,x)

);

Q x ( ( x<>G6 et

x<>sipunculus_nudus

)

=>

-R(G7,x)

);

Q x ( x<>G7

=>

-R(sipunculus_nudus,x)

);

{ OTU 8: }

R(katharina_tunicata,G8);

R(G8,loxocorone_allax);

Q x ( ( x<>katharina_tunicata et

x<>loxocorone_allax

)

=>

-R(G8,x)

);

Q x ( x<>G8

=>

-R(loxocorone_allax, x)

);

{ CASE 2 IS FIXED: 2 edges between homo_sapiens and priapulus_caudatus - second form }

R(homo_sapiens,G9);

Q x ( x<> G9

=>

-R(homo_sapiens,x)

);

R(G9,priapulus_caudatus);

Q x ( ( x<>priapulus_caudatus et

x<>homo_sapiens

)

=>

-R(G9,x)

);

{ PROPERTY P1: R(x, y) is not reflexive}

Q x (-R(x, x));

{ PROPERTY P2: R(x, y) is symetrical}

Q x y (R(x, y) => R(y, x));

{ PROPERTY P3: graph T is connected and acyclic (T is a tree) }

{

This property is verified by a constraint programmed in the model generator, instead of a "heavy" logical formula:

1- it will refuse the partial interpretations in which a connected component of the graph (in construction) is cyclic, i.e. such as: number of edges >= number of vertices

2- it will refuse the complete interpretations in which the constructed graph has more than one connected component

}

{ PROPERTY P4: graph T respects minimal distance matrix }

{

This property is verified by a constraint programmed in the model generator:

it will refuse the partial interpretations in which the graph (in construction) do not respect the minimal distance matrix, i.e. such as:

let x, y a couple of OTUs,

let d= minimal distance calculated between x and y (encoded in the minimal distance matrix), there is a path of length k between x and y, with: k < d

The minimal distance matrix is encoded directly in the data structure of the model generator:

/* minimal distance matrix lophotrochozoans taxF: */

DIST[0][0]=0;

DIST[1][0]=1; DIST[1][1]=0;

DIST[2][0]=2; DIST[2][1]=3; DIST[2][2]=0;

DIST[3][0]=3; DIST[3][1]=4; DIST[3][2]=4; DIST[3][3]=0;

DIST[4][0]=5; DIST[4][1]=5; DIST[4][2]=5; DIST[4][3]=3; DIST[4][4]=0;

DIST[5][0]=4; DIST[5][1]=4; DIST[5][2]=5; DIST[5][3]=3; DIST[5][4]=4; DIST[5][5]=0;

DIST[6][0]=2; DIST[6][1]=3; DIST[6][2]=2; DIST[6][3]=4; DIST[6][4]=4; DIST[6][5]=4; DIST[6][6]=0;

DIST[7][0]=3; DIST[7][1]=4; DIST[7][2]=3; DIST[7][3]=4; DIST[7][4]=5; DIST[7][5]=4; DIST[7][6]=2; DIST[7][7]=0;

DIST[8][0]=2; DIST[8][1]=2; DIST[8][2]=4; DIST[8][3]=5; DIST[8][4]=5; DIST[8][5]=5; DIST[8][6]=4; DIST[8][7]=4; DIST[8][8]=0;

DIST[9][0]=1; DIST[9][1]=2; DIST[9][2]=2; DIST[9][3]=3; DIST[9][4]=4; DIST[9][5]=5; DIST[9][6]=2; DIST[9][7]=3; DIST[9][8]=3; DIST[9][9]=0;

DIST[10][0]=1; DIST[10][1]=2; DIST[10][2]=3; DIST[10][3]=4; DIST[10][4]=5; DIST[10][5]=4; DIST[10][6]=3; DIST[10][7]=4; DIST[10][8]=3; DIST[10][9]=2; DIST[10][10]=0;

DIST[11][0]=4; DIST[11][1]=5; DIST[11][2]=5; DIST[11][3]=6; DIST[11][4]=6; DIST[11][5]=6; DIST[11][6]=5; DIST[11][7]=5; DIST[11][8]=5; DIST[11][9]=4; DIST[11][10]=4; DIST[11][11]=0;

DIST[12][0]=5; DIST[12][1]=6; DIST[12][2]=6; DIST[12][3]=5; DIST[12][4]=7; DIST[12][5]=6; DIST[12][6]=6; DIST[12][7]=7; DIST[12][8]=7; DIST[12][9]=5; DIST[12][10]=5; DIST[12][11]=7; DIST[12][12]=0;

}

{ PROPERTY P5: graph T respects eventual Primary Phylogenetic Hypotheses }

{

This property is verified by constraints programmed in the model generator:

- monophyly of Lophotrochozoa = (0,1,2,3,4,5,8,9,10,11,12)

- monophyly of Eutrochozoa = (0,1,2,3,4,5,8)

- monophyly of Mollusca = (0,1,2)

- monophyly of Polyplacophora = (0)

- monophyly of Cephalopoda = (0,1,2)

- monophyly of Annelida = (3,4)

- monophyly of Echiura = (3,4)

- monophyly of Polychaeta = (3)

- monophyly of Lophophorata = (9,10,11,12)

- monophyly of Brachiopoda = (9,12)

Notes:

1- katharina_tunicata mtDNA is identical to octopus_vulgaris mtDNA (mollusc *Cephalopoda*), considering only the 15 protein-coding genes and rRNA genes. Thus, katharina_tunicata must be also element of Cephalopoda clade in this computation.

2- platynereis_dumerilii mtDNA is identical to clymenella_torquata mtDNA (annelid *Echiura*). Thus, platynereis_dumerilii must be also element of *Echiura* clade in this computation.

}

{------------------------------------------------------------------------------------------------------------------------}

{ PROPERTY P6: it is possible to calculate all the values for each HTU in the graph T }

{

First we calculate with the model generator the set of tree solutions which verify properties P1 to P5. Property P6 is verified *a posteriori* for each tree solution, with a *feedback* mechanism:

Studying each tree solution for calculating the values of HTUs, we eventually discover "impossible sub-trees": they appear in tree solutions which verify P1 to P5, but they do not verify P6.

For each impossible subtree A, an additional constraint is programmed into the model generator to forbid the solutions containing A. Tree solutions are recalculated and verified, allowing the discovery of new impossible subtrees and the programming of new constraints to recalculate the solutions (feedback mechanism). Finally, the complete set of optimal solutions is determined after iteration of this process and elimination of all the solutions that do not verify P6.

}

================================================================================

================================================================================

SOLUTIONS

================================================================================

================================================================================

OTUs:

katharina_tunicata = 0;

nautilus_macromphallus = 1;

loligo_bleekeri = 2;

platynereis_dumerilii = 3;

urechis_caupo = 4;

sipunculus_nudus = 5;

priapulus_caudatus = 6; { = outgroup1 }

homo_sapiens = 7; { = outgroup2 }

loxocorone_allax = 8;

terebratulina_retusa = 9;

phoronis_architecta = 10;

bugula_neritina=11;

terebratalia_transversa=12;

AUXILLIARY CONSTANTS used to fix a part of the solution:

G1=13; { used to fix Cephalopoda group }

G2=14; G3=15; G4=16; G5=17;

G6=18; G7=19; G8=20; { used to fix the rest of Eutrochozoa group }

G9=21; { used to fix CASE 2 }

HTUs:

n1, n2, n3, n4, n5, n6, n7, n8

D = [0,29]: 9 solutions OK (which verify property P6) (143 impossible sub-trees)

minimal score (best) = 101

maximal score = 135

-------------------------------------------------------------------------------------------------------------

score = 111:

model 1:

-------------

R(0,1) R(0,9) R(0,10) R(0,13) R(0,14) R(0,20) R(0,n3) R(0,n6) R(1,0) R(2,13) R(3,15) R(3,16) R(4,17) R(5,19) R(6,21) R(6,n6) R(7,21) R(8,20) R(9,0) R(10,0) R(10,n4) R(11,n8) R(12,n7) R(13,0) R(13,2) R(14,0) R(14,15) R(15,3) R(15,14) R(15,18) R(16,3) R(16,17) R(17,4) R(17,16) R(18,15) R(18,19) R(19,5) R(19,18) R(20,0) R(20,8) R(21,6) R(21,7) R(n1,n2) R(n1,n7) R(n2,n1) R(n2,n3) R(n3,0) R(n3,n2) R(n4,10) R(n4,n5) R(n5,n4) R(n5,n8) R(n6,0) R(n6,6) R(n7,12) R(n7,n1) R(n8,11) R(n8,n5)

-------------------------------------------------------------------------------------------------------------

-> the best model

score = 101:

model 2:

-------------

R(0,1) R(0,9) R(0,10) R(0,13) R(0,14) R(0,20) R(0,n3) R(0,n5) R(0,n6) R(1,0) R(2,13) R(3,15) R(3,16) R(4,17) R(5,19) R(6,21) R(6,n6) R(7,21) R(8,20) R(9,0) R(10,0) R(11,n8) R(12,n7) R(13,0) R(13,2) R(14,0) R(14,15) R(15,3) R(15,14) R(15,18) R(16,3) R(16,17) R(17,4) R(17,16) R(18,15) R(18,19) R(19,5) R(19,18) R(20,0) R(20,8) R(21,6) R(21,7) R(n1,n2) R(n1,n7) R(n2,n1) R(n2,n3) R(n3,0) R(n3,n2) R(n4,n5) R(n4,n8) R(n5,0) R(n5,n4) R(n6,0) R(n6,6) R(n7,12) R(n7,n1) R(n8,11) R(n8,n4)

-------------------------------------------------------------------------------------------------------------

score = 121:

model 3:

-------------

R(0,1) R(0,9) R(0,10) R(0,13) R(0,14) R(0,20) R(0,n6) R(1,0) R(2,13) R(3,15) R(3,16) R(4,17) R(5,19) R(6,21) R(6,n6) R(7,21) R(8,20) R(9,0) R(9,n3) R(10,0) R(10,n4) R(11,n8) R(12,n7) R(13,0) R(13,2) R(14,0) R(14,15) R(15,3) R(15,14) R(15,18) R(16,3) R(16,17) R(17,4) R(17,16) R(18,15) R(18,19) R(19,5) R(19,18) R(20,0) R(20,8) R(21,6) R(21,7) R(n1,n2) R(n1,n7) R(n2,n1) R(n2,n3) R(n3,9) R(n3,n2) R(n4,10) R(n4,n5) R(n5,n4) R(n5,n8) R(n6,0) R(n6,6) R(n7,12) R(n7,n1) R(n8,11) R(n8,n5)

-------------------------------------------------------------------------------------------------------------

score = 111:

model 4:

-------------

R(0,1) R(0,9) R(0,10) R(0,13) R(0,14) R(0,20) R(0,n5) R(0,n6) R(1,0) R(2,13) R(3,15) R(3,16) R(4,17) R(5,19) R(6,21) R(6,n6) R(7,21) R(8,20) R(9,0) R(9,n3) R(10,0) R(11,n8) R(12,n7) R(13,0) R(13,2) R(14,0) R(14,15) R(15,3) R(15,14) R(15,18) R(16,3) R(16,17) R(17,4) R(17,16) R(18,15) R(18,19) R(19,5) R(19,18) R(20,0) R(20,8) R(21,6) R(21,7) R(n1,n2) R(n1,n7) R(n2,n1) R(n2,n3) R(n3,9) R(n3,n2) R(n4,n5) R(n4,n8) R(n5,0) R(n5,n4) R(n6,0) R(n6,6) R(n7,12) R(n7,n1) R(n8,11) R(n8,n4)

-------------------------------------------------------------------------------------------------------------

score = 119:

model 5:

-------------

R(0,1) R(0,9) R(0,10) R(0,13) R(0,14) R(0,20) R(0,n6) R(1,0) R(2,13) R(3,15) R(3,16) R(4,17) R(5,19) R(6,21) R(6,n6) R(7,21) R(8,20) R(9,0) R(9,n3) R(9,n5) R(10,0) R(11,n8) R(12,n7) R(13,0) R(13,2) R(14,0) R(14,15) R(15,3) R(15,14) R(15,18) R(16,3) R(16,17) R(17,4) R(17,16) R(18,15) R(18,19) R(19,5) R(19,18) R(20,0) R(20,8) R(21,6) R(21,7) R(n1,n2) R(n1,n7) R(n2,n1) R(n2,n3) R(n3,9) R(n3,n2) R(n4,n5) R(n4,n8) R(n5,9) R(n5,n4) R(n6,0) R(n6,6) R(n7,12) R(n7,n1) R(n8,11) R(n8,n4)

-------------------------------------------------------------------------------------------------------------

score = 121:

model 6:

-------------

R(0,1) R(0,10) R(0,13) R(0,14) R(0,20) R(0,n3) R(0,n6) R(1,0) R(2,13) R(3,15) R(3,16) R(4,17) R(5,19) R(6,21) R(6,n6) R(7,21) R(8,20) R(9,n3) R(10,0) R(10,n4) R(11,n8) R(12,n7) R(13,0) R(13,2) R(14,0) R(14,15) R(15,3) R(15,14) R(15,18) R(16,3) R(16,17) R(17,4) R(17,16) R(18,15) R(18,19) R(19,5) R(19,18) R(20,0) R(20,8) R(21,6) R(21,7) R(n1,n2) R(n1,n7) R(n2,n1) R(n2,n3) R(n3,0) R(n3,9) R(n3,n2) R(n4,10) R(n4,n5) R(n5,n4) R(n5,n8) R(n6,0) R(n6,6) R(n7,12) R(n7,n1) R(n8,11) R(n8,n5)

-------------------------------------------------------------------------------------------------------------

score = 111:

model 7:

-------------

R(0,1) R(0,10) R(0,13) R(0,14) R(0,20) R(0,n3) R(0,n5) R(0,n6) R(1,0) R(2,13) R(3,15) R(3,16) R(4,17) R(5,19) R(6,21) R(6,n6) R(7,21) R(8,20) R(9,n3) R(10,0) R(11,n8) R(12,n7) R(13,0) R(13,2) R(14,0) R(14,15) R(15,3) R(15,14) R(15,18) R(16,3) R(16,17) R(17,4) R(17,16) R(18,15) R(18,19) R(19,5) R(19,18) R(20,0) R(20,8) R(21,6) R(21,7) R(n1,n2) R(n1,n7) R(n2,n1) R(n2,n3) R(n3,0) R(n3,9) R(n3,n2) R(n4,n5) R(n4,n8) R(n5,0) R(n5,n4) R(n6,0) R(n6,6) R(n7,12) R(n7,n1) R(n8,11) R(n8,n4)

-------------------------------------------------------------------------------------------------------------

score = 135:

model 8:

-------------

R(0,1) R(0,10) R(0,13) R(0,14) R(0,20) R(0,n5) R(1,0) R(2,13) R(3,15) R(3,16) R(4,17) R(5,19) R(6,21) R(6,n5) R(7,21) R(8,20) R(9,n3) R(10,0) R(10,n3) R(10,n4) R(11,n8) R(12,n7) R(13,0) R(13,2) R(14,0) R(14,15) R(15,3) R(15,14) R(15,18) R(16,3) R(16,17) R(17,4) R(17,16) R(18,15) R(18,19) R(19,5) R(19,18) R(20,0) R(20,8) R(21,6) R(21,7) R(n1,n2) R(n1,n7) R(n2,n1) R(n2,n3) R(n3,9) R(n3,10) R(n3,n2) R(n4,10) R(n4,n6) R(n5,0) R(n5,6) R(n6,n4) R(n6,n8) R(n7,12) R(n7,n1) R(n8,11) R(n8,n6)

-------------------------------------------------------------------------------------------------------------

score = 129:

model 9:

-------------

R(0,1) R(0,10) R(0,13) R(0,14) R(0,20) R(0,n5) R(0,n6) R(1,0) R(2,13) R(3,15) R(3,16) R(4,17) R(5,19) R(6,21) R(6,n6) R(7,21) R(8,20) R(9,n3) R(10,0) R(10,n3) R(11,n8) R(12,n7) R(13,0) R(13,2) R(14,0) R(14,15) R(15,3) R(15,14) R(15,18) R(16,3) R(16,17) R(17,4) R(17,16) R(18,15) R(18,19) R(19,5) R(19,18) R(20,0) R(20,8) R(21,6) R(21,7) R(n1,n2) R(n1,n7) R(n2,n1) R(n2,n3) R(n3,9) R(n3,10) R(n3,n2) R(n4,n5) R(n4,n8) R(n5,0) R(n5,n4) R(n6,0) R(n6,6) R(n7,12) R(n7,n1) R(n8,11) R(n8,n4)

-------------------------------------------------------------------------------------------------------------

-> no other solutions
