# Supplementary material for: A complete logical approach to resolve the evolution and dynamics of mitochondrial genome in bilaterians
Source: PLoS One. 2018 Mar 16;13(3):e0194334. doi: 10.1371/journal.pone.0194334 (PMC5856267; doi:10.1371/journal.pone.0194334)
Supplement: S9 Appendix — (DOC) [file pone.0194334.s009.doc]

S9 Appendix. Axioms and solutions for Bilateria.

bilaterians_taxA_6sol page 2

bilaterians_taxA_6sol

================================================================================

================================================================================

AXIOMS

================================================================================

================================================================================

{ the solutions of problem PHYLO are the smallest graphs T (defined on the smallest domain possible but containing at least all the OTUs) which verify properties P1 to P6:

P1- T is simple (the relation R(x, y) which defines graph T is not reflexive)

P2- T is non-oriented (the relation R(x, y) which defines graph T is symetrical)

P3- T is connected and acyclic (T is a tree)

P4- T respects the minimal distance matrix, *i.e.*:

for all couple of OTUs x and y, the length of the path x->y in T is always superior or equals to the minimal distance calculated between x and y (encoded in the minimal distance matrix)

P5- T respects other eventual hypothesis (Primary Phylogenetic Hypothesis = PPH)

used to impose the existence of given monophyletic groups

P6- it is possible to calculate all the values for each HTU in the graph T }

{ OTUs: }

homo_sapiens=0;

priapulus_caudautus=1;

limulus_polyphemus=2;

katharina_tunicata=3;

tethya_actinia_15g=4; { = outgroup }

{ PROPERTY P1: R(x, y) is not reflexive}

Q x (-R(x, x));

{ PROPERTY P2: R(x, y) is symetrical}

Q x y (R(x, y) => R(y, x));

{ PROPERTY P3: graph T is connected and acyclic (T is a tree) }

{

This property is verified by a constraint programmed in the model generator, instead of a "heavy" logical formula:

1- it will refuse the partial interpretations in which a connected component of the graph (in construction) is cyclic, i.e. such as: number of edges >= number of vertices

2- it will refuse the complete interpretations in which the constructed graph has more than one connected component

}

{ PROPERTY P4: graph T respects minimal distance matrix }

{

This property is verified by a constraint programmed in the model generator:

it will refuse the partial interpretations in which the graph (in construction) do not respect the minimal distance matrix, i.e. such as:

let x, y a couple of OTUs,

let d= minimal distance calculated between x and y (encoded in the minimal distance matrix), there is a a path of length k between x and y, with: k < d

The minimal distance matrix is encoded directly in the data structure of the model generator:

/* minimal distance matrix the base of bilaterians - taxA: */

DIST[0][0]=0;

DIST[1][0]=2; DIST[1][1]=0;

DIST[2][0]=2; DIST[2][1]=1; DIST[2][2]=0;

DIST[3][0]=3; DIST[3][1]=2; DIST[3][2]=2; DIST[3][3]=0;

DIST[4][0]=4; DIST[4][1]=5; DIST[4][2]=5; DIST[4][3]=6; DIST[4][4]=0;

}

{ PROPERTY P5: graph T respects eventual Primary Phylogenetic Hypotheses }

{

This property is verified by constraints programmed in the model generator:

- monophyly of Ecdysozoa = (1,2)

- monophyly of Bilateria = (0,1,2,3)

}

{------------------------------------------------------------------------------------------------------------------------}

{ PROPERTY P6: it is possible to calculate all the values for each HTU in the graph T }

{

First we calculate with the model generator the set of tree solutions which verify properties P1 to P5. Property P6 is verified *a posteriori* for each tree solution, with a *feedback* mechanism:

Studying each tree solution for calculating the values of HTUs, we eventually discover "impossible sub-trees": they appear in tree solutions which verify P1 to P5, but they do not verify P6.

For each impossible subtree A, an additional constraint is programmed into the model generator to forbid the solutions containing A. Tree solutions are recalculated and verified, allowing the discovery of new impossible subtrees and the programming of new constraints to recalculate the solutions (feedback mechanism). Finally, the complete set of optimal solutions is determined after iteration of this process and elimination of all the solutions that do not verify P6.

}

================================================================================

================================================================================

SOLUTIONS

================================================================================

================================================================================

OTUs:

homo_sapiens=0;

priapulus_caudautus=1;

limulus_polyphemus=2;

katharina_tunicata=3;

tethya_actinia_15g=4; { = outgroup }

HTUs:

n1, n2, n3, n4, n5

D = [0,9]: 6 solutions OK (which verify property P6) (8 impossible sub-trees)

minimal score (best) = 6

maximal score = 10

-------------------------------------------------------------------------------------------------------------

-> BASE5

(correspond to the form SOL2_Alter, and to the form BILA 11 with chaetognaths)

score = 8:

model 1:

-------------

R(0,n1) R(0,n2) R(1,2) R(1,n2) R(1,n3) R(2,1) R(3,n3) R(4,n4) R(n1,0) R(n1,n5) R(n2,0) R(n2,1) R(n3,1) R(n3,3) R(n4,4) R(n4,n5) R(n5,n1) R(n5,n4)

-------------------------------------------------------------------------------------------------------------

-> BASE2

(correspond to the form SOL2, and to the form BILA 2 with chaetognaths)

score = 8:

model 2:

-------------

R(0,n1) R(0,n2) R(1,2) R(2,1) R(2,n2) R(2,n3) R(3,n3) R(4,n4) R(n1,0) R(n1,n5) R(n2,0) R(n2,2) R(n3,2) R(n3,3) R(n4,4) R(n4,n5) R(n5,n1) R(n5,n4)

-------------------------------------------------------------------------------------------------------------

-> BASE3

(correspond to the form SOL3, and to the form BILA 3 with chaetognaths)

n3 = [ cox1 cox2 atp8 atp6 cox3 nad3 -nad5 -nad4 -nad4L -cob -nad6 -nad1 -rrnL -rrnS nad2 ]

score = 10:

model 3:

-------------

R(0,n1) R(0,n2) R(1,2) R(2,1) R(2,n3) R(3,n3) R(4,n4) R(n1,0) R(n1,n5) R(n2,0) R(n2,n3) R(n3,2) R(n3,3) R(n3,n2) R(n4,4) R(n4,n5) R(n5,n1) R(n5,n4)

-------------------------------------------------------------------------------------------------------------

-> BASE4

(correspond to the form SOL1_Alter, and to the form BILA 10 with chaetognaths)

n2 = [ cox1 cox2 atp8 atp6 cox3 nad3 -nad5 -nad4 -nad4L nad6 cob rrnS rrnL nad1 nad2 ]

score = 8:

model 4:

-------------

R(0,n1) R(0,n2) R(1,2) R(1,n2) R(2,1) R(3,n3) R(4,n4) R(n1,0) R(n1,n5) R(n2,0) R(n2,1) R(n2,n3) R(n3,3) R(n3,n2) R(n4,4) R(n4,n5) R(n5,n1) R(n5,n4)

-------------------------------------------------------------------------------------------------------------

-> BASE1

(correspond to the form SOL1, and to the form BILA 1 with chaetognaths)

n2 = [ cox1 cox2 atp8 atp6 cox3 nad3 -nad5 -nad4 -nad4L nad6 cob rrnS rrnL nad1 nad2 ]

score = 8:

model 5:

-------------

R(0,n1) R(0,n2) R(1,2) R(2,1) R(2,n2) R(3,n3) R(4,n4) R(n1,0) R(n1,n5) R(n2,0) R(n2,2) R(n2,n3) R(n3,3) R(n3,n2) R(n4,4) R(n4,n5) R(n5,n1) R(n5,n4)

-------------------------------------------------------------------------------------------------------------

-> BASE6

(correspond to the form SOL1 + SOL1_Alter, and to the form BILA 15 with chaetognaths)

n2 = UR1 = [ cox1 cox2 atp8 atp6 cox3 nad3 -nad5 -nad4 -nad4L nad6 cob rrnS rrnL nad1 nad2 ]

score = 6:

model 6:

-------------

R(0,n1) R(0,n2) R(1,n2) R(2,n2) R(3,n3) R(4,n4) R(n1,0) R(n1,n5) R(n2,0) R(n2,1) R(n2,2) R(n2,n3) R(n3,3) R(n3,n2) R(n4,4) R(n4,n5) R(n5,n1) R(n5,n4)

-------------------------------------------------------------------------------------------------------------

-> no other solutions
