# Supplementary material for: A complete logical approach to resolve the evolution and dynamics of mitochondrial genome in bilaterians
Source: PLoS One. 2018 Mar 16;13(3):e0194334. doi: 10.1371/journal.pone.0194334 (PMC5856267; doi:10.1371/journal.pone.0194334)
Supplement: S10 Appendix — (DOC) [file pone.0194334.s010.doc]

S10 Appendix. Log book 2 - Chronological description of computations for Bilateria – Annexe for Chaetognatha.

-------------------------------------------------------------------------

(1) computation of the CHAETOGNATHA tree with outgroup limulus_polyphemus

-------------------------------------------------------------------------

CHOICE OF THE TAXONOMIC DATASET:

All the chaetognaths (4), all the lophotrochozoans (11), and the outgroups homo_sapiens (Ur-deuterostomia) and limulus_polyphemus (ecdysozoa).

LIST OF OTUs for the computation "chaetognaths_taxA":

katharina_tunicata=0; (Eutrochozoa Mollusca Polyplacophora)

nautilus_macromphallus=1; (Eutrochozoa Mollusca Cephalopoda)

loligo_bleekeri=2; (Eutrochozoa Mollusca Cephalopoda)

platynereis_dumerilii=3; (Eutrochozoa Annelida)

urechis_caupo=4; (Eutrochozoa Annelida)

sipunculus_nudus=5; (Eutrochozoa Sipunculida)

limulus_polyphemus=6; (Ecdysozoa) = OUTGROUP1

homo_sapiens=7; (Ur-deuterostomia) = OUTGROUP2

loxocorone_allax=8; (Eutrochozoa Entoprocta)

terebratulina_retusa=9; (Lophophorata Brachiopoda)

phoronis_architecta=10; (Lophophorata Phoronida)

bugula_neritina=11; (Lophophorata Bryozoa)

terebratalia_transversa=12; (Lophophorata Brachiopoda)

sagitta_enflata=13; (Chaetognatha) (13 genes)

sagitta_nagae=14; (Chaetognatha) (13 genes)

paraspadella_gotoi=15; (Chaetognatha) (13 genes)

spadella_cephaloptera=16; (Chaetognatha) (13 genes)

PRIMARY PHYLOGENETIC HYPOTHESYS (PPH) used:

- monophyly of Lophotrochozoa = (0,1,2,3,4,5,8,9,10,11,12)

- monophyly of Eutrochozoa = (0,1,2,3,4,5,8)

- monophyly of Mollusca = (0,1,2)

- monophyly of Polyplacophora = (0)

- monophyly of Cephalopoda = (0,1,2)

- monophyly of Annelida = (3,4)

- monophyly of Echiura = (3,4)

- monophyly of Polychaeta = (3)

- monophyly of Lophophorata = (9,10,11,12)

- monophyly of Chaetognatha = (13,14,15,16)

Notes:

1- katharina_tunicata mtDNA is identical to octopus_vulgaris mtDNA (mollusc *Cephalopoda*), considering only the 15 protein-coding genes and rRNA genes. Thus, katharina_tunicata must be also element of Cephalopoda clade in this computation.

2- platynereis_dumerilii mtDNA is identical to clymenella_torquata mtDNA (annelid *Echiura*). Thus, platynereis_dumerilii must be also element of *Echiura* clade in this computation.

ADDITIONNAL HYPOTHESIS:

We fix the eutrochozoa group (OTUs 0,1,2,3,4,5,8) and the lophophorata group (OTUs 9,10,11,12) with their best possible form (and Ur-lophotrochozoa = katharina_tunicata).

Therefore this computation "chaetognaths_taxA" is similar to a computation of the Chaetognatha tree with the 3 outgroups: homo_sapiens, limulus_polyphemus, and katharina_tunicata.

A PARTICULAR CASE: THE LOSS OF GENES atp8 and atp6 FOR THE CHAETOGNATHS

To calculate the minimal distance between a chaetognath (13 genes) and another mtDNA (15 genes), we only consider the 13 genes common to the 2 mtDNAs, in order to compute the minimal paths between genomes of same size.

Then we add 1 or 2 steps for the loss of genes atp8 and atp6.

But to compute the tree solutions, it is simpler to code this minimal distance in the distance matrix of the model generator *without adding anything* (for the loss of genes atp8 and atp6), as if we "forgot" the loss steps. This is done in order to simplify the computation. Then we will insert *a posteriori* in the tree solutions the necessary loss steps (as we did in "deuterostomes_taxC").

Here too, for the chaetognaths, *only one loss step is necessary* (loss of the 2 successive genes atp8-atp6, *proper to the chaetognaths lineage*: it is the most parsimonious possibility). In effect, when we calculate the values of the ancestral states in each solution (OK for P6), we see that the mtDNA associated to the node at the start of the chaetognaths lineage *always contains the 2 successive genes atp8 and atp6*.

In our solution files, we chose arbitrarily to insert this loss step at the start of the chaetognaths lineage, so the possible values for the ancestral state at the start of the chaetognaths lineage are given: first, with 15 genes (before the step "loss of genes atp8-atp6"), then with 13 genes (after the step "loss of genes atp8-atp6").

But in fact it is possible to insert this loss step at any position *proper to the chaetognaths lineage*, and easily reconstruct the values of the ancestral states (with 15 genes) at the start of the lineage, before the loss step.

SOLUTIONS:

On domain D=[0,38], we obtain:

141 possible solutions (OK for P6) (221 impossible subtrees).

(see S11 appendix - section 'chaetognaths_taxA_141sol')

The analysis of all solutions shows the following results:

-> for the subtree HOLIKAT restricted to homo_sapiens, limulus_polyphemus and katharina_tunicata, there are 9 possible forms:

form SOL1: (4 edges) (previously obtained)

R(homo_sapiens,x1) and

R(x1,limulus_polyphemus) and

R(x1,x2) and

R(x2,katharina_tunicata)

form SOL2: (4 edges) (previously obtained)

R(homo_sapiens,x1) and

R(x1,limulus_polyphemus) and

R(limulus_polyphemus,x2) and

R(x2,katharina_tunicata)

form SOL3: (4 edges) (previously obtained)

R(homo_sapiens,x1) and

R(x1,x2) and

R(x2,limulus_polyphemus) and

R(x2,katharina_tunicata)

form SOL4: (5 edges)

R(homo_sapiens,x1) and

R(x1,limulus_polyphemus) and

R(x1,x2) and

R(x2,x3) and

R(x3,katharina_tunicata)

form SOL5: (5 edges)

R(homo_sapiens,x1) and

R(x1,limulus_polyphemus) and

R(limulus_polyphemus,x2) and

R(x2,x3) and

R(x3,katharina_tunicata)

form SOL6: (5 edges)

R(homo_sapiens,x1) and

R(x1,x2) and

R(x2,limulus_polyphemus) and

R(x2,x3) and

R(x3,katharina_tunicata)

form SOL7: (5 edges)

R(homo_sapiens,x1) and

R(x1,x2) and

R(x2,limulus_polyphemus) and

R(x1,x3) and

R(x3,katharina_tunicata)

form SOL8: (5 edges)

R(homo_sapiens,x1) and

R(x1,x2) and

R(x2,limulus_polyphemus) and

R(limulus_polyphemus,x3) and

R(x3,katharina_tunicata)

form SOL9: (5 edges)

R(homo_sapiens,x1) and

R(x1,x2) and

R(x2,x3) and

R(x3,limulus_polyphemus) and

R(x3,katharina_tunicata)

-> for the subtree HOLIKAT *with* the start of the chaetognaths lineage, there are 19 possible configurations:

SOL1 - A, SOL1 - B, SOL1 - C, SOL1 - D:

4 possible positions for the start of the chaetognaths lineage: katharina_tunicata, x2, x1, and limulus_polyphemus

(with 9 edges + 1 loss atp8-atp6 = 10 edges for the chaetognaths lineage)

SOL2 - A, SOL2 - B, SOL2 - C, SOL2 - D:

4 possible positions for the start of the chaetognaths lineage: katharina_tunicata, x2, limulus_polyphemus, and x1

(with 9 edges + 1 loss atp8-atp6 = 10 edges for the chaetognaths lineage)

SOL3 - A, SOL3 - B, SOL3 - C, SOL3 - D:

4 possible positions for the start of the chaetognaths lineage: katharina_tunicata, x2, limulus_polyphemus, and x1

(with 9 edges + 1 loss atp8-atp6 = 10 edges for the chaetognaths lineage)

SOL4 - A, SOL4 - B:

2 possible positions for the start of the chaetognaths lineage:

x3 and x2

(with 8 edges + 1 loss atp8-atp6 = 9 edges for the chaetognaths lineage)

SOL5 - A:

1 possible position for the start of the chaetognaths lineage: x3

(with 8 edges + 1 loss atp8-atp6 = 9 edges for the chaetognaths lineage)

SOL6 - A:

1 possible position for the start of the chaetognaths lineage: x3

(with 8 edges + 1 loss atp8-atp6 = 9 edges for the chaetognaths lineage)

SOL7 - A:

1 possible position for the start of the chaetognaths lineage: x2

(with 8 edges + 1 loss atp8-atp6 = 9 edges for the chaetognaths lineage)

SOL8 - A:

1 possible position for the start of the chaetognaths lineage: x2

(with 8 edges + 1 loss atp8-atp6 = 9 edges for the chaetognaths lineage)

SOL9 - A:

1 possible position for the start of the chaetognaths lineage: x2

(with 8 edges + 1 loss atp8-atp6 = 9 edges for the chaetognaths lineage)

-> for the subtree HOLIKAT with the complete Chaetognatha group, there are 141 possible forms:

WITH 10 EDGES for the chaetognaths lineage, and 4 EDGES for HOLIKAT:

* SOL1 - A: 13 possible forms for the Chaetognatha group

* SOL1 - B: the same 13 possible forms

* SOL1 - C: 1 possible form (as model 8 / SOL1 - A)

* SOL1 - D: 1 possible form (as model 8 / SOL1 - A)

* SOL2 - A: the same 13 possible forms

* SOL2 - B: the same 13 possible forms *PLUS* 2 new forms (models 126 and 127, possible because of the specific value of the mtDNA at the start of the chaetognaths lineage) = 15 forms all together

* SOL2 - C: 1 possible form (as model 8 / SOL1 - A)

* SOL2 - D: 1 possible form (as model 8 / SOL1 - A)

* SOL3 - A: the same 13 possible forms

* SOL3 - B: the same 13 possible forms

* SOL3 - C: 1 possible form (as model 8 / SOL1 - A)

* SOL3 - D: 1 possible form (as model 8 / SOL1 - A)

WITH 9 EDGES for the chaetognaths lineage, and 5 EDGES for HOLIKAT:

* SOL4 - A: the same 13 possible forms

* SOL4 - B: 1 possible form (as model 8 / SOL1 - A)

* SOL5 - A: the same 13 possible forms

* SOL6 - A: the same 13 possible forms

* SOL7 - A: 1 possible form (as model 8 / SOL1 - A)

* SOL8 - A: 1 possible form (as model 8 / SOL1 - A)

* SOL9 - A: the same 13 possible forms

-> in the Chaetognatha group, there is always 1 possible form for sagitta_enflata and sagitta_nagae (OTUs 13 and 14), with the existence of a sagitta group, and sagitta_enflata always at the base of this group:

R(sagitta_enflata, sagitta_nagae) and

sagitta_enflata linked with the rest of the tree

(Ur-sagitta = sagitta_enflata)

ADDITIONNAL COMPUTATIONS:

-> if no PPH are imposed, we obtain (with the lophotrochozoa group fixed) exactly the same solutions. Therefore, the PPH Chaetognatha is a logical consequence of the problem.

-------------------------------------------------------------------------

(2) Computation of the CHAETOGNATHA tree with outgroup priapulus_caudatus

-------------------------------------------------------------------------

CHOICE OF THE TAXONOMIC DATASET:

All the chaetognaths (4), all the lophotrochozoans (11), and the outgroups homo_sapiens (Ur-deuterostomia) and priapulus_caudatus (ecdysozoa).

LIST OF OTUs for the computation "chaetognaths_taxB":

katharina_tunicata=0; (Eutrochozoa Mollusca Polyplacophora)

nautilus_macromphallus=1; (Eutrochozoa Mollusca Cephalopoda)

loligo_bleekeri=2; (Eutrochozoa Mollusca Cephalopoda)

platynereis_dumerilii=3; (Eutrochozoa Annelida)

urechis_caupo=4; (Eutrochozoa Annelida)

sipunculus_nudus=5; (Eutrochozoa Sipunculida)

priapulus_caudatus=6; (Ecdysozoa) = OUTGROUP1

homo_sapiens=7; (Ur-deuterostomia) = OUTGROUP2

loxocorone_allax=8; (Eutrochozoa Entoprocta)

terebratulina_retusa=9; (Lophophorata Brachiopoda)

phoronis_architecta=10; (Lophophorata Phoronida)

bugula_neritina=11; (Lophophorata Bryozoa)

terebratalia_transversa=12; (Lophophorata Brachiopoda)

sagitta_enflata=13; (Chaetognatha)

sagitta_nagae=14; (Chaetognatha)

paraspadella_gotoi=15; (Chaetognatha)

spadella_cephaloptera=16; (Chaetognatha)

(the same OTUs than in "chaetognaths_taxA" with priapulus_caudatus instead of limulus_polyphemus)

PRIMARY PHYLOGENETIC HYPOTHESYS (PPH) used:

- monophyly of Lophotrochozoa = (0,1,2,3,4,5,8,9,10,11,12)

- monophyly of Eutrochozoa = (0,1,2,3,4,5,8)

- monophyly of Mollusca = (0,1,2)

- monophyly of Polyplacophora = (0)

- monophyly of Cephalopoda = (0,1,2)

- monophyly of Annelida = (3,4)

- monophyly of Echiura = (3,4)

- monophyly of Polychaeta = (3)

- monophyly of Lophophorata = (9,10,11,12)

- monophyly of Chaetognatha = (13,14,15,16)

Notes:

1- katharina_tunicata mtDNA is identical to octopus_vulgaris mtDNA (mollusc *Cephalopoda*), considering only the 15 protein-coding genes and rRNA genes. Thus, katharina_tunicata must be also element of Cephalopoda clade in this computation.

2- platynereis_dumerilii mtDNA is identical to clymenella_torquata mtDNA (annelid *Echiura*). Thus, platynereis_dumerilii must be also element of *Echiura* clade in this computation.

ADDITIONNAL HYPOTHESIS:

We fix the eutrochozoa group (OTUs 0,1,2,3,4,5,8) and the lophophorata group (OTUs 9,10,11,12) with their best possible form (and Ur-lophotrochozoa = katharina_tunicata).

A PARTICULAR CASE: THE LOSS OF GENES atp8 and atp6 FOR THE CHAETOGNATHS

As for the previous computation, we code the minimal distances in the distance matrix of the model generator *without adding anything* for the loss of genes atp8 and atp6, as if we "forgot" the loss steps (in order to simplify the computation).

Then we can insert *a posteriori* in each tree solution obtained one step "loss of the 2 successive genes atp8-atp6" proper to the chaetognaths lineage: it is the most parsimonious possibility.

In our solution files, we chose arbitrarily to insert this loss step *at the start* of the chaetognaths lineage, so the possible values for the ancestral state at the start of the chaetognaths lineage are given: first, with 15 genes (before the step "loss of genes atp8-atp6"), then with 13 genes (after the step "loss of genes atp8-atp6").

SOLUTIONS:

On domain D=[0,38], we obtain:

96 possible solutions (OK for P6) (191 impossible subtrees).

(see S11 appendix - section 'chaetognaths_taxB_96sol')

The analysis of all solutions shows the following results:

-> for the subtree HOPRIAKAT restricted to homo_sapiens, priapulus_caudatus and katharina_tunicata, there are 5 possible forms:

form SOL1_ALTER: (4 edges) (previously obtained)

R(homo_sapiens,x1) and

R(x1,priapulus_caudatus) and

R(x1,x2) and

R(x2,katharina_tunicata)

form SOL2_ALTER: (4 edges) (previously obtained)

R(homo_sapiens,x1) and

R(x1,priapulus_caudatus) and

R(priapulus_caudatus,x2) and

R(x2,katharina_tunicata)

form SOL4_ALTER: (5 edges)

R(homo_sapiens,x1) and

R(x1,priapulus_caudatus) and

R(x1,x2) and

R(x2,x3) and

R(x3,katharina_tunicata)

form SOL5_ALTER: (5 edges)

R(homo_sapiens,x1) and

R(x1,priapulus_caudatus) and

R(priapulus_caudatus,x2) and

R(x2,x3) and

R(x3,katharina_tunicata)

form SOL9_ALTER: (5 edges)

R(homo_sapiens,x1) and

R(x1,x2) and

R(x2,x3) and

R(x3,priapulus_caudatus) and

R(x3,katharina_tunicata)

(SOL"x"_ALTER is equivalent to SOL"x" in which we substitute limulus_polyphemus with priapulus_caudatus)

-> for the subtree HOPRIAKAT *with* the start of the chaetognaths lineage, there are 10 possible configurations:

SOL1_ALTER - A, SOL1_ALTER - B, SOL1_ALTER - C:

3 possible positions for the start of the chaetognaths lineage: katharina_tunicata, x2, and x1

(with 9 edges + 1 loss atp8-atp6 = 10 edges for the chaetognaths lineage)

SOL2_ALTER - A, SOL2_ALTER - B, SOL2_ALTER - C:

3 possible positions for the start of the chaetognaths lineage: katharina_tunicata, x2, and x1

(with 9 edges + 1 loss atp8-atp6 = 10 edges for the chaetognaths lineage)

SOL4_ALTER - A, SOL4_ALTER - B:

2 possible positions for the start of the chaetognaths lineage:

x3 and x2

(with 8 edges + 1 loss atp8-atp6 = 9 edges for the chaetognaths lineage)

SOL5_ALTER - A:

1 possible position for the start of the chaetognaths lineage: x3

(with 8 edges + 1 loss atp8-atp6 = 9 edges for the chaetognaths lineage)

SOL9_ALTER - A:

1 possible position for the start of the chaetognaths lineage: x2

(with 8 edges + 1 loss atp8-atp6 = 9 edges for the chaetognaths lineage)

-> for the subtree HOPRIAKAT with the complete Chaetognatha group, there are 96 possible forms:

WITH 10 EDGES for the chaetognaths lineage, and 4 EDGES for HOPRIAKAT:

* SOL1_ALTER - A: 13 possible forms for the Chaetognatha group

* SOL1_ALTER - B: the same 13 possible forms

* SOL1_ALTER - C: 1 possible form (as model 8 / SOL1_ALTER - A)

* SOL2_ALTER - A: the same 13 possible forms

* SOL2_ALTER - B: the same 13 possible forms *PLUS* 2 new forms (models 86 and 87, possible because of the specific value of the mtDNA at the start of the chaetognaths lineage) = 15 forms all together

* SOL2_ALTER - C: 1 possible form (as model 8 / SOL1_ALTER - A)

WITH 9 EDGES for the chaetognaths lineage, and 5 EDGES for HOPRIAKAT:

* SOL4_ALTER - A: the same 13 possible forms

* SOL4_ALTER - B: 1 possible form (as model 8 / SOL1_ALTER - A)

* SOL5_ALTER - A: the same 13 possible forms

* SOL9_ALTER - A: the same 13 possible forms

-> in the Chaetognatha group, there is always 1 possible form for sagitta_enflata and sagitta_nagae (OTUs 13 and 14), with the existence of a sagitta group, and sagitta_enflata always at the base of this group:

R(sagitta_enflata, sagitta_nagae) and

sagitta_enflata linked with the rest of the tree

(Ur-sagitta = sagitta_enflata)

ADDITIONNAL COMPUTATIONS:

-> if no PPH are imposed, we obtain (with the lophotrochozoa group fixed) exactly the same solutions. The PPH Chaetognatha is a logical consequence of the problem.

-------------------------------------------------------------------------

(3) computation of the CHAETOGNATHA tree with limulus AND priapulus

-------------------------------------------------------------------------

The computation "chaetognaths_taxA" give all the solutions (= 141) for the Chaetognatha group and the base of bilaterians, with Ur-ecdysozoa = limulus_polyphemus (as in models 1,2,3 in computation "ecdysozoans_taxG").

The computation "chaetognaths_taxB" give all the solutions (= 96) for the Chaetognatha group and the base of bilaterians, with Ur-ecdysozoa = priapulus_caudatus (as in models 6,7 in computation "ecdysozoans_taxG").

But, for the ecdysozoa tree, there are also solutions with Ur-ecdysozoa different from limulus_polyphemus and priapulus_caudatus (as in models 4,5 in computation "ecdysozoans_taxG").

Therefore, one last computation is necessary:

The chaetognaths tree with the base of bilaterians with the 2 outgroups limulus_polyphemus AND priapulus_caudatus.

CHOICE OF THE TAXONOMIC DATASET:

All the chaetognaths (4), all the lophotrochozoans (11), and the outgroups homo_sapiens (Ur-deuterostomia), limulus_polyphemus and priapulus_caudatus (ecdysozoans).

LIST OF OTUs for the computation "chaetognaths_taxC":

katharina_tunicata=0; (Eutrochozoa Mollusca Polyplacophora)

nautilus_macromphallus=1; (Eutrochozoa Mollusca Cephalopoda)

loligo_bleekeri=2; (Eutrochozoa Mollusca Cephalopoda)

platynereis_dumerilii=3; (Eutrochozoa Annelida)

urechis_caupo=4; (Eutrochozoa Annelida)

sipunculus_nudus=5; (Eutrochozoa Sipunculida)

limulus_polyphemus=6; (Ecdysozoa) = OUTGROUP1

homo_sapiens=7; (Ur-deuterostomia) = OUTGROUP2

loxocorone_allax=8; (Eutrochozoa Entoprocta)

terebratulina_retusa=9; (Lophophorata Brachiopoda)

phoronis_architecta=10; (Lophophorata Phoronida)

bugula_neritina=11; (Lophophorata Bryozoa)

terebratalia_transversa=12; (Lophophorata Brachiopoda)

sagitta_enflata=13; (Chaetognatha) (13 genes)

sagitta_nagae=14; (Chaetognatha) (13 genes)

paraspadella_gotoi=15; (Chaetognatha) (13 genes)

spadella_cephaloptera=16; (Chaetognatha) (13 genes)

priapulus_caudatus=17; (Ecdysozoa) = OUTGROUP3

(the same OTUs than in "chaetognaths_taxA", plus priapulus_caudatus)

PRIMARY PHYLOGENETIC HYPOTHESYS (PPH) used:

- monophyly of Lophotrochozoa = (0,1,2,3,4,5,8,9,10,11,12)

- monophyly of Eutrochozoa = (0,1,2,3,4,5,8)

- monophyly of Mollusca = (0,1,2)

- monophyly of Polyplacophora = (0)

- monophyly of Cephalopoda = (1,2)

- monophyly of Annelida = (3,4)

- monophyly of Echiura = (3,4)

- monophyly of Polychaeta = (3)

- monophyly of Lophophorata = (9,10,11,12)

- monophyly of Chaetognatha = (13,14,15,16)

- monophyly of Ecdysozoa = (6,17)

Notes:

1- katharina_tunicata mtDNA is identical to octopus_vulgaris mtDNA (mollusc *Cephalopoda*), considering only the 15 protein-coding genes and rRNA genes. Thus, katharina_tunicata must be also element of Cephalopoda clade in this computation.

2- platynereis_dumerilii mtDNA is identical to clymenella_torquata mtDNA (annelid *Echiura*). Thus, platynereis_dumerilii must be also element of *Echiura* clade in this computation.

ADDITIONNAL HYPOTHESIS:

We fix the eutrochozoa group (OTUs 0,1,2,3,4,5,8) and the lophophorata group (OTUs 9,10,11,12) with their best possible form (and Ur-lophotrochozoa = katharina_tunicata).

A PARTICULAR CASE: THE LOSS OF GENES atp8 and atp6 FOR THE CHAETOGNATHS

As for the 2 previous computations, we code the minimal distances in the distance matrix of the model generator *without adding anything* for the loss of genes atp8 and atp6, as if we "forgot" the loss steps (in order to simplify the computation). Then we can insert *a posteriori* in each tree solution obtained one step "loss of the 2 successive genes atp8-atp6" proper to the chaetognaths lineage: it is the most parsimonious possibility.

In our solution files, we chose arbitrarily to insert this loss step *at the start* of the chaetognaths lineage, so the possible values for the ancestral state at the start of the chaetognaths lineage are given: first, with 15 genes (before the step "loss of genes atp8-atp6"), then with 13 genes (after the step "loss of genes atp8-atp6").

SOLUTIONS:

On domain D=[0,39], we obtain:

278 possible solutions (OK for P6) (266 impossible subtrees).

(see S11 appendix - section 'chaetognaths_taxC_278sol')

The analysis of all solutions shows the following results:

-> there are 141 solutions with Ur-ecdysozoa = limulus_polyphemus, already obtained in S11 appendix - section 'chaetognaths_taxA_141sol'

(see further the forms "BILA1" to "BILA9")

-> there are 96 solutions with Ur-ecdysozoa = priapulus_caudatus, already obtained in S11 appendix - section 'chaetognaths_taxB_96sol'

(see further the forms "BILA10" à "BILA14")

-> there are 41 solutions with Ur-ecdysozoa = UR1 =

[ cox1 cox2 atp8 atp6 cox3 nad3 -nad5 -nad4 -nad4L nad6 cob rrnS rrnL nad1 nad2 ]

in which: R(UR1,limulus_polyphemus) and R(UR1,priapulus_caudatus).

They are the new solutions in S11 appendix - section 'chaetognaths_taxC_278sol', for which we give all the possible values for the ancestral states of degree > 2

(see further the forms "BILA15" and "BILA16")

We can describe and organize the 278 solutions (141+96+41 = 278) by considering the possible forms for the subtree "BILA" restricted to the *four* OTUs homo_sapiens, katharina_tunicata, limulus_polyphemus and priapulus_caudatus. 16 forms are possibles:

form BILA1: ( = SOL1 + R(limulus_polyphemus,priapulus_caudatus) )

R(homo_sapiens,x1) and

R(x1,limulus_polyphemus) and

R(x1,x2) and

R(x2,katharina_tunicata) and

R(limulus_polyphemus,priapulus_caudatus)

form BILA2: ( = SOL2 + R(limulus_polyphemus,priapulus_caudatus) )

R(homo_sapiens,x1) and

R(x1,limulus_polyphemus) and

R(limulus_polyphemus,x2) and

R(x2,katharina_tunicata) and

R(limulus_polyphemus,priapulus_caudatus)

form BILA3: ( = SOL3 + R(limulus_polyphemus,priapulus_caudatus) )

R(homo_sapiens,x1) and

R(x1,x2) and

R(x2,limulus_polyphemus) and

R(x2,katharina_tunicata) and

R(limulus_polyphemus,priapulus_caudatus)

form BILA4: ( = SOL4 + R(limulus_polyphemus,priapulus_caudatus) )

R(homo_sapiens,x1) and

R(x1,limulus_polyphemus) and

R(x1,x2) and

R(x2,x3) and

R(x3,katharina_tunicata) and

R(limulus_polyphemus,priapulus_caudatus)

form BILA5: ( = SOL5 + R(limulus_polyphemus,priapulus_caudatus) )

R(homo_sapiens,x1) and

R(x1,limulus_polyphemus) and

R(limulus_polyphemus,x2) and

R(x2,x3) and

R(x3,katharina_tunicata) and

R(limulus_polyphemus,priapulus_caudatus)

form BILA6: ( = SOL6 + R(limulus_polyphemus,priapulus_caudatus) )

R(homo_sapiens,x1) and

R(x1,x2) and

R(x2,limulus_polyphemus) and

R(x2,x3) and

R(x3,katharina_tunicata) and

R(limulus_polyphemus,priapulus_caudatus)

form BILA7: ( = SOL7 + R(limulus_polyphemus,priapulus_caudatus) )

R(homo_sapiens,x1) and

R(x1,x2) and

R(x2,limulus_polyphemus) and

R(x1,x3) and

R(x3,katharina_tunicata) and

R(limulus_polyphemus,priapulus_caudatus)

form BILA8: ( = SOL8 + R(limulus_polyphemus,priapulus_caudatus) )

R(homo_sapiens,x1) and

R(x1,x2) and

R(x2,limulus_polyphemus) and

R(limulus_polyphemus,x3) and

R(x3,katharina_tunicata) and

R(limulus_polyphemus,priapulus_caudatus)

form BILA9: ( = SOL9 + R(limulus_polyphemus,priapulus_caudatus) )

R(homo_sapiens,x1) and

R(x1,x2) and

R(x2,x3) and

R(x3,limulus_polyphemus) and

R(x3,katharina_tunicata) and

R(limulus_polyphemus,priapulus_caudatus)

form BILA10: ( = SOL1_ALTER + R(priapulus_caudatus,limulus_polyphemus) )

R(homo_sapiens,x1) and

R(x1,priapulus_caudatus) and

R(x1,x2) and

R(x2,katharina_tunicata) and

R(priapulus_caudatus,limulus_polyphemus)

form BILA11: ( = SOL2_ALTER + R(priapulus_caudatus,limulus_polyphemus) )

R(homo_sapiens,x1) and

R(x1,priapulus_caudatus) and

R(priapulus_caudatus,x2) and

R(x2,katharina_tunicata) and

R(priapulus_caudatus,limulus_polyphemus)

form BILA12: ( = SOL4_ALTER + R(priapulus_caudatus,limulus_polyphemus) )

R(homo_sapiens,x1) and

R(x1,priapulus_caudatus) and

R(x1,x2) and

R(x2,x3) and

R(x3,katharina_tunicata) and

R(priapulus_caudatus,limulus_polyphemus)

form BILA13: ( = SOL5_ALTER + R(priapulus_caudatus,limulus_polyphemus) )

R(homo_sapiens,x1) and

R(x1,priapulus_caudatus) and

R(priapulus_caudatus,x2) and

R(x2,x3) and

R(x3,katharina_tunicata) and

R(priapulus_caudatus,limulus_polyphemus)

form BILA14: ( = SOL9_ALTER + R(priapulus_caudatus,limulus_polyphemus) )

R(homo_sapiens,x1) and

R(x1,x2) and

R(x2,x3) and

R(x3,priapulus_caudatus) and

R(x3,katharina_tunicata) and

R(priapulus_caudatus,limulus_polyphemus

form BILA15: ( = SOL1 + SOL1_ALTER)

R(homo_sapiens,x1) and

R(x1,limulus_polyphemus) and

R(x1,priapulus_caudatus) and

R(x1,x2) and

R(x2,katharina_tunicata)

form BILA16: ( = SOL4 + SOL4_ALTER)

R(homo_sapiens,x1) and

R(x1,limulus_polyphemus) and

R(x1,priapulus_caudatus) and

R(x1,x2) and

R(x2,x3) and

R(x3,katharina_tunicata)

-> for the subtree BILA *with* the start of the chaetognaths lineage, there are 34 possible configurations:

BILA1-A, BILA1-B, BILA1-C, BILA1-D:

4 possible positions for the start of the chaetognaths lineage: katharina_tunicata, x2, x1, and limulus_polyphemus

(with 9 edges + 1 loss atp8-atp6 = 10 edges for the chaetognaths lineage)

BILA2-A, BILA2-B, BILA2-C, BILA2-D:

4 possible positions for the start of the chaetognaths lineage: katharina_tunicata, x2, limulus_polyphemus, and x1

(with 9 edges + 1 loss atp8-atp6 = 10 edges for the chaetognaths lineage)

BILA3-A, BILA3-B, BILA3-C, BILA3-D:

4 possible positions for the start of the chaetognaths lineage: katharina_tunicata, x2, limulus_polyphemus, and x1

(with 9 edges + 1 loss atp8-atp6 = 10 edges for the chaetognaths lineage)

BILA4-A, BILA4-B:

2 possible positions for the start of the chaetognaths lineage:

x3 and x2

(with 8 edges + 1 loss atp8-atp6 = 9 edges for the chaetognaths lineage)

BILA5-A:

1 possible position for the start of the chaetognaths lineage: x3

(with 8 edges + 1 loss atp8-atp6 = 9 edges for the chaetognaths lineage)

BILA6-A:

1 possible position for the start of the chaetognaths lineage: x3

(with 8 edges + 1 loss atp8-atp6 = 9 edges for the chaetognaths lineage)

BILA7-A:

1 possible position for the start of the chaetognaths lineage: x2

(with 8 edges + 1 loss atp8-atp6 = 9 edges for the chaetognaths lineage)

BILA8-A:

1 possible position for the start of the chaetognaths lineage: x2

(with 8 edges + 1 loss atp8-atp6 = 9 edges for the chaetognaths lineage)

BILA9-A:

1 possible position for the start of the chaetognaths lineage: x2

(with 8 edges + 1 loss atp8-atp6 = 9 edges for the chaetognaths lineage)

BILA10-A, BILA10-B, BILA10-C:

3 possible positions for the start of the chaetognaths lineage: katharina_tunicata, x2, and x1

(with 9 edges + 1 loss atp8-atp6 = 10 edges for the chaetognaths lineage)

BILA11-A, BILA11-B, BILA11-C:

3 possible positions for the start of the chaetognaths lineage: katharina_tunicata, x2, and x1

(with 9 edges + 1 loss atp8-atp6 = 10 edges for the chaetognaths lineage)

BILA12-A, BILA12-B:

2 possible positions for the start of the chaetognaths lineage:

x3 and x2

(with 8 edges + 1 loss atp8-atp6 = 9 edges for the chaetognaths lineage)

BILA13-A:

1 possible position for the start of the chaetognaths lineage: x3

(with 8 edges + 1 loss atp8-atp6 = 9 edges for the chaetognaths lineage)

BILA14-A:

1 possible position for the start of the chaetognaths lineage: x2

(with 8 edges + 1 loss atp8-atp6 = 9 edges for the chaetognaths lineage)

BILA15-A, BILA15-B, BILA15-C:

3 possible positions for the start of the chaetognaths lineage: katharina_tunicata, x2, and x1

(with 9 edges + 1 loss atp8-atp6 = 10 edges for the chaetognaths lineage)

BILA16-A, BILA16-B:

2 possible positions for the start of the chaetognaths lineage:

x3 and x2

(with 8 edges + 1 loss atp8-atp6 = 9 edges for the chaetognaths lineage)

-> for the subtree BILA with the complete Chaetognatha group, there are 278 possible forms:

WITH 10 EDGES for the chaetognaths lineage, and 5 EDGES for BILA:

* BILA1-A: 13 possible forms for the Chaetognatha group

* BILA1-B: the same 13 possible forms

* BILA1-C: 1 possible form

* BILA1-D: 1 possible form

* BILA2-A: the same 13 possible forms

* BILA2-B: the same 13 possible forms *PLUS* 2 new forms, possible because of the specific value of the mtDNA at the start of the chaetognaths lineage) = 15 forms all together

* BILA2-C: 1 possible form

* BILA2-D: 1 possible form

* BILA3-A: the same 13 possible forms

* BILA3-B: the same 13 possible forms

* BILA3-C: 1 possible form

* BILA3-D: 1 possible form

* BILA10-A: the same 13 possible forms

* BILA10-B: the same 13 possible forms

* BILA10-C: 1 possible form

* BILA11-A: the same 13 possible forms

* BILA11-B: the same 13 possible forms *PLUS* 2 new forms, possible because of the specific value of the mtDNA at the start of the chaetognaths lineage) = 15 forms all together

* BILA11-C: 1 possible form

* BILA15-A: the same 13 possible forms

* BILA15-B: the same 13 possible forms

* BILA15-C: 1 possible form

WITH 9 EDGES for the chaetognaths lineage, and 6 EDGES for BILA:

* BILA4-A: the same 13 possible forms

* BILA4-B: 1 possible form

* BILA5-A: the same 13 possible forms

* BILA6-A: the same 13 possible forms

* BILA7-A: 1 possible form

* BILA8-A: 1 possible form

* BILA9-A: the same 13 possible forms

* BILA12-A: the same 13 possible forms

* BILA12-B: 1 possible form

* BILA13-A: the same 13 possible forms

* BILA14-A: the same 13 possible forms

* BILA16-A: the same 13 possible forms

* BILA16-B: 1 possible form

-> in the Chaetognatha group, there is always 1 possible form for sagitta_enflata and sagitta_nagae (OTUs 13 and 14), with the existence of a sagitta group, and sagitta_enflata always at the base of this group:

R(sagitta_enflata, sagitta_nagae) and

sagitta_enflata linked with the rest of the tree

(Ur-sagitta = sagitta_enflata)

ADDITIONNAL COMPUTATIONS:

-> if no PPH are imposed, we obtain (with the lophotrochozoa group fixed) exactly the same solutions. The PPH Chaetognatha is a logical consequence of the problem.

============================================

SYNTHESIS OF THE SOLUTIONS

============================================

THE BASE OF THE BILATERIANS with chaetognaths: (16 possible forms)

-> Ur-bilateria = homo_sapiens

-> 1 possible form for the outgroup tethya_actinia (demospongiae), linked with homo_sapiens by a 5-edges branch (4 + 1 loss of gene atp9)

-> 16 possible forms (BILA1 to BILA16) for the subtree BILA restricted to homo_sapiens, katharina_tunicata, limulus_polyphemus and priapulus_caudatus:

- 6 possible forms with 5 edges

(BILA1, BILA2, BILA3, BILA10, BILA11, BILA15)

- 10 possible forms with 6 edges

(BILA4, BILA5, BILA6, BILA7, BILA8, BILA9, BILA12, BILA13,

BILA14, BILA16)

CHAETOGNATHS: (up to 15 possible forms)

-> 34 possible configurations (BILA1-A, ..., BILA16-B) for the position start of the chaetognaths lineage in the subtree BILA:

21 configurations with 10 edges for the chaetognaths lineage

(9 edges + 1 loss atp8/atp6) and 5 edges for BILA

13 configurations with 9 edges for the chaetognaths lineage

(8 edges + 1 loss atp8/atp6) and 6 edges for BILA

-> up to 15 possible forms for the Chaetognatha group, in each of the 34 previous configurations:

* the same 13 forms for: BILA1-A, BILA1-B, BILA2-A, BILA2-B, BILA3-A, BILA3-B, BILA4-A, BILA5-A, BILA6-A, BILA9-A, BILA10-A, BILA10-B, BILA11-A, BILA11-B, BILA12-A, BILA13-A, BILA14-A, BILA15-A, BILA15-B, BILA16-A

(plus 2 supplementary forms for BILA2-B and BILA11-B = 15 forms all together)

* the same 1 form for BILA1-C, BILA1-D, BILA2-C, BILA2-D, BILA3-C, BILA3-D, BILA4-B, BILA7-A, BILA8-A, BILA10-C, BILA11-C, BILA12-B, BILA15-C, BILA16-B

-> 278 possible forms all together for the Chaetognatha group with the subtree BILA

-> in the Chaetognatha group, there is always:

R(sagitta_enflata, sagitta_nagae) and

sagitta_enflata linked with the rest of the tree

(monophyly of Sagitta, and Ur-sagitta = sagitta_enflata)

-------------------------------

Verification of the solutions:

-------------------------------

All the tree solutions can be verified with the program *genome_comparison.c*, by calculating the paths between nodes.

To facilitate these verifications, all the possible values for the ancestral states of degree > 2 are given in the solution files (except redundancies).
