# Supplementary material for: A complete logical approach to resolve the evolution and dynamics of mitochondrial genome in bilaterians
Source: PLoS One. 2018 Mar 16;13(3):e0194334. doi: 10.1371/journal.pone.0194334 (PMC5856267; doi:10.1371/journal.pone.0194334)
Supplement: S11 Appendix — (DOC) [file pone.0194334.s011.doc]

S11 Appendix. Axioms and solutions for Chaetognatha.

chaetognaths_taxA_141sol page 2

chaetognaths_taxB_96sol page 64

chaetognaths_taxC_278sol page 110

chaetognaths_taxA_141sol

================================================================================

================================================================================

AXIOMS

================================================================================

================================================================================

{ the solutions of problem PHYLO are the smallest graphs T (defined on the smallest domain possible but containing at least all the OTUs) which verify properties P1 to P6:

P1- T is simple (the relation R(x, y) which defines graph T is not reflexive)

P2- T is non-oriented (the relation R(x, y) which defines graph T is symmetrical)

P3- T is connected and acyclic (T is a tree)

P4- T respects the minimal distance matrix, i.e.:

for all couple of OTUs x and y, the length of the path x->y in T is always superior or equals to the minimal distance calculated between x and y (encoded in the minimal distance matrix)

P5- T respects other eventual hypothesis (Primary Phylogenetic Hypothesis = PPH)

used to impose the existence of given monophyletic groups

P6- it is possible to calculate all the values for each HTU in the graph T }

{ OTUs: }

katharina_tunicata = 0;

nautilus_macromphallus = 1;

loligo_bleekeri = 2;

platynereis_dumerilii = 3;

urechis_caupo = 4;

sipunculus_nudus = 5;

limulus_polyphemus = 6; { = outgroup1 }

homo_sapiens = 7; { = outgroup2 }

loxocorone_allax = 8;

terebratulina_retusa = 9;

phoronis_architecta = 10;

bugula_neritina=11;

terebratalia_transversa=12;

sagitta_enflata=13;

sagitta_nagae=14;

paraspadella_gotoi=15;

spadella_cephaloptera=16;

{ AUXILLIARY CONSTANTS used to fix a part of the solution: }

G1=17; G2=18; G3=19; G4=20;

G5=21; G6=22; G7=23; G8=24; { used to fix the Eutrochozoa group }

G9=25; G10=26; G11=27; G12=28;

G13=29; G14=30; G15=31; { used to fix the Lophophorata group }

{ THE EUTROCHOZOA GROUP IS FIXED: }

{ OTUs (0,1,2): }

R(katharina_tunicata,nautilus_macromphallus);

R(katharina_tunicata,G1);

R(G1,loligo_bleekeri);

Q x ( x<>katharina_tunicata

=>

-R(nautilus_macromphallus,x)

);

Q x ( ( x<>katharina_tunicata et

x<>loligo_bleekeri

)

=>

-R(G1,x)

);

Q x ( x<>G1

=>

-R(loligo_bleekeri,x)

);

{ OTUs (3,4,5,8): }

{ as in the best of the 3 possible forms }

R(katharina_tunicata,G2);

R(G2,G3);

R(G3,platynereis_dumerilii);

R(platynereis_dumerilii,G4);

R(G4,G5);

R(G5,urechis_caupo);

R(G3,G6);

R(G6,G7);

R(G7,sipunculus_nudus);

Q x ( ( x<>katharina_tunicata et

x<>G3

)

=>

-R(G2,x)

);

Q x ( ( x<>G2 et

x<>platynereis_dumerilii et

x<>G6

)

=>

-R(G3,x)

);

Q x ( ( x<>platynereis_dumerilii et

x<>G5

)

=>

-R(G4,x)

);

Q x ( ( x<>G3 et

x<>G4

)

=>

-R(platynereis_dumerilii,x)

);

Q x ( ( x<>G4 et

x<>urechis_caupo

)

=>

-R(G5,x)

);

Q x ( x<>G5

=>

-R(urechis_caupo,x)

);

Q x ( ( x<>G3 et

x<>G7

)

=>

-R(G6,x)

);

Q x ( ( x<>G6 et

x<>sipunculus_nudus

)

=>

-R(G7,x)

);

Q x ( x<>G7

=>

-R(sipunculus_nudus,x)

);

R(katharina_tunicata,G8);

R(G8,loxocorone_allax);

Q x ( ( x<>katharina_tunicata et

x<>loxocorone_allax

)

=>

-R(G8,x)

);

Q x ( x<>G8

=>

-R(loxocorone_allax, x)

);

{ THE LOPHOPHORATA GROUP IS FIXED: }

{ as in the best of the 9 possible forms }

R(katharina_tunicata,terebratulina_retusa);

R(katharina_tunicata,phoronis_architecta);

Q x ( x<>katharina_tunicata

=>

-R(terebratulina_retusa,x)

);

Q x ( x<>katharina_tunicata

=>

-R(phoronis_architecta,x)

);

R(katharina_tunicata,G9);

R(G9,G10);

R(G10,G11);

R(G11,bugula_neritina);

Q x ( ( x<>katharina_tunicata et

x<>G10

)

=>

-R(G9,x)

);

Q x ( ( x<>G9 et

x<>G11

)

=>

-R(G10,x)

);

Q x ( ( x<>G10 et

x<>bugula_neritina

)

=>

-R(G11,x)

);

Q x ( x<>G11

=>

-R(bugula_neritina,x)

);

R(katharina_tunicata,G12);

R(G12,G13);

R(G13,G14);

R(G14,G15);

R(G15,terebratalia_transversa);

Q x ( ( x<>katharina_tunicata et

x<>G13

)

=>

-R(G12,x)

);

Q x ( ( x<>G12 et

x<>G14

)

=>

-R(G13,x)

);

Q x ( ( x<>G13 et

x<>G15

)

=>

-R(G14,x)

);

Q x ( ( x<>G14 et

x<>terebratalia_transversa

)

=>

-R(G15,x)

);

Q x ( x<>G15

=>

-R(terebratalia_transversa,x)

);

{ PROPERTY P1: R(x, y) is not reflexive}

Q x (-R(x, x));

{ PROPERTY P2: R(x, y) is symmetrical}

Q x y (R(x, y) => R(y, x));

{ PROPERTY P3: graph T is connected and acyclic (T is a tree) }

{

This property is verified by a constraint programmed in the model generator, instead of a "heavy" logical formula:

1- it will refuse the partial interpretations in which a connected component of the graph (in construction) is cyclic, i.e. such as: number of edges >= number of vertices

2- it will refuse the complete interpretations in which the constructed graph has more than one connected component

}

{ PROPERTY P4: graph T respects minimal distance matrix }

{

This property is verified by a constraint programmed in the model generator:

it will refuse the partial interpretations in which the graph (in construction) do not respect the minimal distance matrix, i.e. such as:

let x, y a couple of OTUs,

let d= minimal distance calculated between x and y (encoded in the minimal distance matrix), there is a a path of length k between x and y, with: k < d

The minimal distance matrix is encoded directly in the data structure of the model generator:

/* minimal distance matrix chaetognaths taxA: */

DIST[0][0]=0;

DIST[1][0]=1; DIST[1][1]=0;

DIST[2][0]=2; DIST[2][1]=3; DIST[2][2]=0;

DIST[3][0]=3; DIST[3][1]=4; DIST[3][2]=4; DIST[3][3]=0;

DIST[4][0]=5; DIST[4][1]=5; DIST[4][2]=5; DIST[4][3]=3; DIST[4][4]=0;

DIST[5][0]=4; DIST[5][1]=4; DIST[5][2]=5; DIST[5][3]=3; DIST[5][4]=4; DIST[5][5]=0;

DIST[6][0]=2; DIST[6][1]=3; DIST[6][2]=2; DIST[6][3]=4; DIST[6][4]=5; DIST[6][5]=4; DIST[6][6]=0;

DIST[7][0]=3; DIST[7][1]=4; DIST[7][2]=3; DIST[7][3]=4; DIST[7][4]=5; DIST[7][5]=4; DIST[7][6]=2; DIST[7][7]=0;

DIST[8][0]=2; DIST[8][1]=2; DIST[8][2]=4; DIST[8][3]=5; DIST[8][4]=5; DIST[8][5]=5; DIST[8][6]=4; DIST[8][7]=4; DIST[8][8]=0;

DIST[9][0]=1; DIST[9][1]=2; DIST[9][2]=2; DIST[9][3]=3; DIST[9][4]=4; DIST[9][5]=5; DIST[9][6]=2; DIST[9][7]=3; DIST[9][8]=3; DIST[9][9]=0;

DIST[10][0]=1; DIST[10][1]=2; DIST[10][2]=3; DIST[10][3]=4; DIST[10][4]=5; DIST[10][5]=4; DIST[10][6]=3; DIST[10][7]=4; DIST[10][8]=3; DIST[10][9]=2; DIST[10][10]=0;

DIST[11][0]=4; DIST[11][1]=5; DIST[11][2]=5; DIST[11][3]=6; DIST[11][4]=6; DIST[11][5]=6; DIST[11][6]=5; DIST[11][7]=5; DIST[11][8]=5; DIST[11][9]=4; DIST[11][10]=4; DIST[11][11]=0;

DIST[12][0]=5; DIST[12][1]=6; DIST[12][2]=6; DIST[12][3]=5; DIST[12][4]=7; DIST[12][5]=6; DIST[12][6]=6; DIST[12][7]=7; DIST[12][8]=7; DIST[12][9]=5; DIST[12][10]=5; DIST[12][11]=7; DIST[12][12]=0;

DIST[13][0]=4; DIST[13][1]=4; DIST[13][2]=5; DIST[13][3]=3; DIST[13][4]=5; DIST[13][5]=5; DIST[13][6]=5; DIST[13][7]=5; DIST[13][8]=5; DIST[13][9]=4; DIST[13][10]=4; DIST[13][11]=4; DIST[13][12]=6; DIST[13][13]=0;

DIST[14][0]=5; DIST[14][1]=4; DIST[14][2]=6; DIST[14][3]=4; DIST[14][4]=6; DIST[14][5]=6; DIST[14][6]=5; DIST[14][7]=5; DIST[14][8]=5; DIST[14][9]=5; DIST[14][10]=5; DIST[14][11]=4; DIST[14][12]=6; DIST[14][13]=1; DIST[14][14]=0;

DIST[15][0]=4; DIST[15][1]=4; DIST[15][2]=5; DIST[15][3]=4; DIST[15][4]=5; DIST[15][5]=5; DIST[15][6]=5; DIST[15][7]=5; DIST[15][8]=5; DIST[15][9]=4; DIST[15][10]=4; DIST[15][11]=4; DIST[15][12]=5; DIST[15][13]=1; DIST[15][14]=2; DIST[15][15]=0;

DIST[16][0]=6; DIST[16][1]=5; DIST[16][2]=6; DIST[16][3]=4; DIST[16][4]=5; DIST[16][5]=6; DIST[16][6]=6; DIST[16][7]=6; DIST[16][8]=5; DIST[16][9]=5; DIST[16][10]=5; DIST[16][11]=5; DIST[16][12]=6; DIST[16][13]=3; DIST[16][14]=4; DIST[16][15]=3; DIST[16][16]=0;

}

{ PROPERTY P5: graph T respects eventual Primary Phylogenetic Hypotheses }

{

This property is verified by constraints programmed in the model generator:

- monophyly of Lophotrochozoa = (0,1,2,3,4,5,8,9,10,11,12)

- monophyly of Eutrochozoa = (0,1,2,3,4,5,8)

- monophyly of Mollusca = (0,1,2)

- monophyly of Cephalopoda = (1,2)

- monophyly of Annelida = (3,4)

- monophyly of Lophophorata = (9,10,11,12)

- monophyly of Chaetognatha = (13,14,15,16)

}

{------------------------------------------------------------------------------------------------------------------------}

{ PROPERTY P6: it is possible to calculate all the values for each HTU in the graph T }

{

First we calculate with the model generator the set of tree solutions which verify properties P1 to P5. Property P6 is verified *a posteriori* for each tree solution, with a *feedback* mechanism:

Studying each tree solution for calculating the values of HTUs, we eventually discover "impossible sub-trees": they appear in tree solutions which verify P1 to P5, but they do not verify P6.

For each impossible subtree A, an additional constraint is programmed into the model generator to forbid the solutions containing A. Tree solutions are recalculated and verified, allowing the discovery of new impossible subtrees and the programming of new constraints to recalculate the solutions (feedback mechanism). Finally, the complete set of optimal solutions is determined after iteration of this process and elimination of all the solutions that do not verify P6.

}

================================================================================

================================================================================

SOLUTIONS

================================================================================

================================================================================

OTUs:

katharina_tunicata = 0;

nautilus_macromphallus = 1;

loligo_bleekeri = 2;

platynereis_dumerilii = 3;

urechis_caupo = 4;

sipunculus_nudus = 5;

limulus_polyphemus = 6; { = outgroup1 }

homo_sapiens = 7; { = outgroup2 }

loxocorone_allax = 8;

terebratulina_retusa = 9;

phoronis_architecta = 10;

bugula_neritina=11;

terebratalia_transversa=12;

sagitta_enflata=13;

sagitta_nagae=14;

paraspadella_gotoi=15;

spadella_cephaloptera=16;

AUXILLIARY CONSTANTS used to fix a part of the solution:

G1=17; G2=18; G3=19; G4=20;

G5=21; G6=22; G7=23; G8=24; { used to fix the Eutrochozoa group }

G9=25; G10=26; G11=27; G12=28;

G13=29; G14=30; G15=31; { used to fix the Lophophorata group }

HTUs:

n1, n2, n3, n4, n5, n6, n7

NOTE:

In every solution, we have to *insert* in the chaetognaths lineage (OTUs 13,14,15,16) one mutation "loss of the 2 successive genes atp8-atp6" proper to the chaetognaths lineage (common for all chaetognaths): it is the most parsimonious possibility.

In every solution of this file, this "loss" mutation can be inserted at the *beginning* of the chaetognaths lineage, and the possible values for the corresponding ancestral state are given first with 15 genes (before the loss) and then with 13 genes (after the loss of genes atp8-atp6).

But it is possible to insert this loss mutation *at* *any position* proper to the chaetognaths lineage, and easily reconstruct the possible values for ancestral states (with 15 genes) at the beginning of the chaetognaths lineage, before the loss mutation.

D = [0,38]: 141 solutions OK (which verify property P6) (221 impossible sub-trees)

minimal score (best) = 214

maximal score = 392

-------------------------------------------------------------------------------------------------------------

-> form outgroup SOL2 - A

score = 250:

model 1:

-------------

R(0,1) R(0,9) R(0,10) R(0,17) R(0,18) R(0,24) R(0,25) R(0,28) R(0,n1) R(0,n2) R(1,0) R(2,17) R(3,19) R(3,20) R(4,21) R(5,23) R(6,n2) R(6,n3) R(7,n3) R(8,24) R(9,0) R(10,0) R(11,27) R(12,31) R(13,14) R(13,15) R(13,n4) R(13,n5) R(14,13) R(15,13) R(16,n6) R(17,0) R(17,2) R(18,0) R(18,19) R(19,3) R(19,18) R(19,22) R(20,3) R(20,21) R(21,4) R(21,20) R(22,19) R(22,23) R(23,5) R(23,22) R(24,0) R(24,8) R(25,0) R(25,26) R(26,25) R(26,27) R(27,11) R(27,26) R(28,0) R(28,29) R(29,28) R(29,30) R(30,29) R(30,31) R(31,12) R(31,30) R(n1,0) R(n1,n7) R(n2,0) R(n2,6) R(n3,6) R(n3,7) R(n4,13) R(n4,n6) R(n5,13) R(n5,n7) R(n6,16) R(n6,n4) R(n7,n1) R(n7,n5)

-------------------------------------------------------------------------------------------------------------

-> form outgroup SOL1 - A

n3(mod2)

[ cox1 cox2 atp8 atp6 cox3 nad3 -nad5 -nad4 -nad4L nad6 cob rrnS rrnL nad1 nad2 ]

score = 250:

model 2:

-------------

R(0,1) R(0,9) R(0,10) R(0,17) R(0,18) R(0,24) R(0,25) R(0,28) R(0,n1) R(0,n2) R(1,0) R(2,17) R(3,19) R(3,20) R(4,21) R(5,23) R(6,n3) R(7,n3) R(8,24) R(9,0) R(10,0) R(11,27) R(12,31) R(13,14) R(13,15) R(13,n4) R(13,n5) R(14,13) R(15,13) R(16,n6) R(17,0) R(17,2) R(18,0) R(18,19) R(19,3) R(19,18) R(19,22) R(20,3) R(20,21) R(21,4) R(21,20) R(22,19) R(22,23) R(23,5) R(23,22) R(24,0) R(24,8) R(25,0) R(25,26) R(26,25) R(26,27) R(27,11) R(27,26) R(28,0) R(28,29) R(29,28) R(29,30) R(30,29) R(30,31) R(31,12) R(31,30) R(n1,0) R(n1,n7) R(n2,0) R(n2,n3) R(n3,6) R(n3,7) R(n3,n2) R(n4,13) R(n4,n6) R(n5,13) R(n5,n7) R(n6,16) R(n6,n4) R(n7,n1) R(n7,n5)

-------------------------------------------------------------------------------------------------------------

-> form outgroup SOL2 - A

n4(mod3)

[ cox1 cox2 nad1 nad3 nad2 cox3 rrnS -nad4L -nad4 -nad5 -cob -nad6 rrnL ]

score = 264:

model 3:

-------------

R(0,1) R(0,9) R(0,10) R(0,17) R(0,18) R(0,24) R(0,25) R(0,28) R(0,n1) R(0,n2) R(1,0) R(2,17) R(3,19) R(3,20) R(4,21) R(5,23) R(6,n2) R(6,n3) R(7,n3) R(8,24) R(9,0) R(10,0) R(11,27) R(12,31) R(13,14) R(13,n4) R(13,n5) R(14,13) R(15,n4) R(16,n6) R(17,0) R(17,2) R(18,0) R(18,19) R(19,3) R(19,18) R(19,22) R(20,3) R(20,21) R(21,4) R(21,20) R(22,19) R(22,23) R(23,5) R(23,22) R(24,0) R(24,8) R(25,0) R(25,26) R(26,25) R(26,27) R(27,11) R(27,26) R(28,0) R(28,29) R(29,28) R(29,30) R(30,29) R(30,31) R(31,12) R(31,30) R(n1,0) R(n1,n7) R(n2,0) R(n2,6) R(n3,6) R(n3,7) R(n4,13) R(n4,15) R(n4,n6) R(n5,13) R(n5,n7) R(n6,16) R(n6,n4) R(n7,n1) R(n7,n5)

-------------------------------------------------------------------------------------------------------------

-> form outgroup SOL1 - A

n3(mod4)

[ cox1 cox2 atp8 atp6 cox3 nad3 -nad5 -nad4 -nad4L nad6 cob rrnS rrnL nad1 nad2 ]

n4(mod4)

[ cox1 cox2 nad1 nad3 nad2 cox3 rrnS -nad4L -nad4 -nad5 -cob -nad6 rrnL ]

score = 264:

model 4:

-------------

R(0,1) R(0,9) R(0,10) R(0,17) R(0,18) R(0,24) R(0,25) R(0,28) R(0,n1) R(0,n2) R(1,0) R(2,17) R(3,19) R(3,20) R(4,21) R(5,23) R(6,n3) R(7,n3) R(8,24) R(9,0) R(10,0) R(11,27) R(12,31) R(13,14) R(13,n4) R(13,n5) R(14,13) R(15,n4) R(16,n6) R(17,0) R(17,2) R(18,0) R(18,19) R(19,3) R(19,18) R(19,22) R(20,3) R(20,21) R(21,4) R(21,20) R(22,19) R(22,23) R(23,5) R(23,22) R(24,0) R(24,8) R(25,0) R(25,26) R(26,25) R(26,27) R(27,11) R(27,26) R(28,0) R(28,29) R(29,28) R(29,30) R(30,29) R(30,31) R(31,12) R(31,30) R(n1,0) R(n1,n7) R(n2,0) R(n2,n3) R(n3,6) R(n3,7) R(n3,n2) R(n4,13) R(n4,15) R(n4,n6) R(n5,13) R(n5,n7) R(n6,16) R(n6,n4) R(n7,n1) R(n7,n5)

-------------------------------------------------------------------------------------------------------------

-> form outgroup SOL2 - A

n4(mod5)

[ cox1 cox2 nad1 nad3 nad2 cox3 rrnS -nad5 -nad4 -nad4L -cob -nad6 rrnL ]

score = 240:

model 5:

-------------

R(0,1) R(0,9) R(0,10) R(0,17) R(0,18) R(0,24) R(0,25) R(0,28) R(0,n1) R(0,n2) R(1,0) R(2,17) R(3,19) R(3,20) R(4,21) R(5,23) R(6,n1) R(6,n3) R(7,n3) R(8,24) R(9,0) R(10,0) R(11,27) R(12,31) R(13,14) R(13,n4) R(13,n5) R(14,13) R(15,n4) R(16,n7) R(17,0) R(17,2) R(18,0) R(18,19) R(19,3) R(19,18) R(19,22) R(20,3) R(20,21) R(21,4) R(21,20) R(22,19) R(22,23) R(23,5) R(23,22) R(24,0) R(24,8) R(25,0) R(25,26) R(26,25) R(26,27) R(27,11) R(27,26) R(28,0) R(28,29) R(29,28) R(29,30) R(30,29) R(30,31) R(31,12) R(31,30) R(n1,0) R(n1,6) R(n2,0) R(n2,n6) R(n3,6) R(n3,7) R(n4,13) R(n4,15) R(n4,n6) R(n5,13) R(n5,n7) R(n6,n2) R(n6,n4) R(n7,16) R(n7,n5)

-------------------------------------------------------------------------------------------------------------

-> form outgroup SOL1 - A

n3(mod6)

[ cox1 cox2 atp8 atp6 cox3 nad3 -nad5 -nad4 -nad4L nad6 cob rrnS rrnL nad1 nad2 ]

n4(mod6)

[ cox1 cox2 nad1 nad3 nad2 cox3 rrnS -nad5 -nad4 -nad4L -cob -nad6 rrnL ]

score = 240:

model 6:

-------------

R(0,1) R(0,9) R(0,10) R(0,17) R(0,18) R(0,24) R(0,25) R(0,28) R(0,n1) R(0,n2) R(1,0) R(2,17) R(3,19) R(3,20) R(4,21) R(5,23) R(6,n3) R(7,n3) R(8,24) R(9,0) R(10,0) R(11,27) R(12,31) R(13,14) R(13,n4) R(13,n5) R(14,13) R(15,n4) R(16,n7) R(17,0) R(17,2) R(18,0) R(18,19) R(19,3) R(19,18) R(19,22) R(20,3) R(20,21) R(21,4) R(21,20) R(22,19) R(22,23) R(23,5) R(23,22) R(24,0) R(24,8) R(25,0) R(25,26) R(26,25) R(26,27) R(27,11) R(27,26) R(28,0) R(28,29) R(29,28) R(29,30) R(30,29) R(30,31) R(31,12) R(31,30) R(n1,0) R(n1,n3) R(n2,0) R(n2,n6) R(n3,6) R(n3,7) R(n3,n1) R(n4,13) R(n4,15) R(n4,n6) R(n5,13) R(n5,n7) R(n6,n2) R(n6,n4) R(n7,16) R(n7,n5)

-------------------------------------------------------------------------------------------------------------

-> form outgroup SOL2 - A

n4(mod7)

g1: [ cox1 cox2 nad1 nad3 nad2 cox3 rrnS -nad4 -nad5 -cob -nad6 -nad4L rrnL ]

g2: [ cox1 cox2 nad1 nad3 nad2 cox3 rrnS -nad4 -nad5 -cob -nad6 rrnL -nad4L ]

g3: [ cox1 cox2 nad1 nad3 nad2 cox3 rrnS nad5 nad4 -nad4L -cob -nad6 rrnL ]

g4: [ cox1 cox2 nad1 nad3 nad2 cox3 rrnS rrnL nad6 cob nad4L nad5 nad4 ]

g5: [ cox1 cox2 nad1 nad3 nad2 cox3 rrnS -rrnL nad6 cob nad4L nad5 nad4 ]

g6: [ cox1 cox2 nad1 nad3 nad2 cox3 rrnS -nad4 -nad5 rrnL nad6 cob nad4L ]

g7: [ cox1 cox2 nad1 nad3 nad2 cox3 rrnS nad6 cob nad4L -nad4 -nad5 rrnL ]

g8: [ cox1 cox2 nad1 nad3 nad2 cox3 rrnS -nad4 -nad5 nad6 cob nad4L rrnL ]

g9: [ cox1 cox2 nad1 nad3 nad2 cox3 rrnS -rrnL nad6 cob nad4L -nad4 -nad5 ]

g10: [ cox1 cox2 nad1 nad3 nad2 cox3 rrnS -nad4 -nad5 -rrnL -nad4L -cob -nad6 ]

g11: [ cox1 cox2 nad1 nad3 nad2 cox3 rrnS -nad4 -nad5 -nad4L -cob -nad6 -rrnL ]

score = 276:

model 7:

-------------

R(0,1) R(0,9) R(0,10) R(0,17) R(0,18) R(0,24) R(0,25) R(0,28) R(0,n1) R(0,n2) R(1,0) R(2,17) R(3,19) R(3,20) R(4,21) R(5,23) R(6,n2) R(6,n3) R(7,n3) R(8,24) R(9,0) R(10,0) R(11,27) R(12,31) R(13,14) R(13,15) R(13,n4) R(14,13) R(15,13) R(16,n5) R(17,0) R(17,2) R(18,0) R(18,19) R(19,3) R(19,18) R(19,22) R(20,3) R(20,21) R(21,4) R(21,20) R(22,19) R(22,23) R(23,5) R(23,22) R(24,0) R(24,8) R(25,0) R(25,26) R(26,25) R(26,27) R(27,11) R(27,26) R(28,0) R(28,29) R(29,28) R(29,30) R(30,29) R(30,31) R(31,12) R(31,30) R(n1,0) R(n1,n7) R(n2,0) R(n2,6) R(n3,6) R(n3,7) R(n4,13) R(n4,n5) R(n4,n6) R(n5,16) R(n5,n4) R(n6,n4) R(n6,n7) R(n7,n1) R(n7,n6)

-------------------------------------------------------------------------------------------------------------

-> form outgroup SOL1 - A

n3(mod8)

[ cox1 cox2 atp8 atp6 cox3 nad3 -nad5 -nad4 -nad4L nad6 cob rrnS rrnL nad1 nad2 ]

n4(mod8)

g1: [ cox1 cox2 nad1 nad3 nad2 cox3 rrnS -nad4 -nad5 -cob -nad6 -nad4L rrnL ]

g2: [ cox1 cox2 nad1 nad3 nad2 cox3 rrnS -nad4 -nad5 -cob -nad6 rrnL -nad4L ]

g3: [ cox1 cox2 nad1 nad3 nad2 cox3 rrnS nad5 nad4 -nad4L -cob -nad6 rrnL ]

g4: [ cox1 cox2 nad1 nad3 nad2 cox3 rrnS rrnL nad6 cob nad4L nad5 nad4 ]

g5: [ cox1 cox2 nad1 nad3 nad2 cox3 rrnS -rrnL nad6 cob nad4L nad5 nad4 ]

g6: [ cox1 cox2 nad1 nad3 nad2 cox3 rrnS -nad4 -nad5 rrnL nad6 cob nad4L ]

g7: [ cox1 cox2 nad1 nad3 nad2 cox3 rrnS nad6 cob nad4L -nad4 -nad5 rrnL ]

g8: [ cox1 cox2 nad1 nad3 nad2 cox3 rrnS -nad4 -nad5 nad6 cob nad4L rrnL ]

g9: [ cox1 cox2 nad1 nad3 nad2 cox3 rrnS -rrnL nad6 cob nad4L -nad4 -nad5 ]

g10: [ cox1 cox2 nad1 nad3 nad2 cox3 rrnS -nad4 -nad5 -rrnL -nad4L -cob -nad6 ]

g11: [ cox1 cox2 nad1 nad3 nad2 cox3 rrnS -nad4 -nad5 -nad4L -cob -nad6 -rrnL ]

score = 276:

model 8:

-------------

R(0,1) R(0,9) R(0,10) R(0,17) R(0,18) R(0,24) R(0,25) R(0,28) R(0,n1) R(0,n2) R(1,0) R(2,17) R(3,19) R(3,20) R(4,21) R(5,23) R(6,n3) R(7,n3) R(8,24) R(9,0) R(10,0) R(11,27) R(12,31) R(13,14) R(13,15) R(13,n4) R(14,13) R(15,13) R(16,n5) R(17,0) R(17,2) R(18,0) R(18,19) R(19,3) R(19,18) R(19,22) R(20,3) R(20,21) R(21,4) R(21,20) R(22,19) R(22,23) R(23,5) R(23,22) R(24,0) R(24,8) R(25,0) R(25,26) R(26,25) R(26,27) R(27,11) R(27,26) R(28,0) R(28,29) R(29,28) R(29,30) R(30,29) R(30,31) R(31,12) R(31,30) R(n1,0) R(n1,n7) R(n2,0) R(n2,n3) R(n3,6) R(n3,7) R(n3,n2) R(n4,13) R(n4,n5) R(n4,n6) R(n5,16) R(n5,n4) R(n6,n4) R(n6,n7) R(n7,n1) R(n7,n6)

-------------------------------------------------------------------------------------------------------------

-> form outgroup SOL1 - A

n3(mod9)

[ cox1 cox2 atp8 atp6 cox3 nad3 -nad5 -nad4 -nad4L nad6 cob rrnS rrnL nad1 nad2 ]

score = 276:

model 9:

-------------

R(0,1) R(0,9) R(0,10) R(0,17) R(0,18) R(0,24) R(0,25) R(0,28) R(0,n1) R(0,n2) R(1,0) R(2,17) R(3,19) R(3,20) R(4,21) R(5,23) R(6,n3) R(7,n3) R(8,24) R(9,0) R(10,0) R(11,27) R(12,31) R(13,14) R(13,15) R(13,n4) R(14,13) R(15,13) R(15,n6) R(16,n5) R(17,0) R(17,2) R(18,0) R(18,19) R(19,3) R(19,18) R(19,22) R(20,3) R(20,21) R(21,4) R(21,20) R(22,19) R(22,23) R(23,5) R(23,22) R(24,0) R(24,8) R(25,0) R(25,26) R(26,25) R(26,27) R(27,11) R(27,26) R(28,0) R(28,29) R(29,28) R(29,30) R(30,29) R(30,31) R(31,12) R(31,30) R(n1,0) R(n1,n3) R(n2,0) R(n2,n7) R(n3,6) R(n3,7) R(n3,n1) R(n4,13) R(n4,n5) R(n5,16) R(n5,n4) R(n6,15) R(n6,n7) R(n7,n2) R(n7,n6)

-------------------------------------------------------------------------------------------------------------

-> form outgroup SOL2 - A

score = 276:

model 10:

-------------

R(0,1) R(0,9) R(0,10) R(0,17) R(0,18) R(0,24) R(0,25) R(0,28) R(0,n1) R(0,n2) R(1,0) R(2,17) R(3,19) R(3,20) R(4,21) R(5,23) R(6,n1) R(6,n3) R(7,n3) R(8,24) R(9,0) R(10,0) R(11,27) R(12,31) R(13,14) R(13,15) R(13,n4) R(14,13) R(15,13) R(15,n7) R(16,n5) R(17,0) R(17,2) R(18,0) R(18,19) R(19,3) R(19,18) R(19,22) R(20,3) R(20,21) R(21,4) R(21,20) R(22,19) R(22,23) R(23,5) R(23,22) R(24,0) R(24,8) R(25,0) R(25,26) R(26,25) R(26,27) R(27,11) R(27,26) R(28,0) R(28,29) R(29,28) R(29,30) R(30,29) R(30,31) R(31,12) R(31,30) R(n1,0) R(n1,6) R(n2,0) R(n2,n6) R(n3,6) R(n3,7) R(n4,13) R(n4,n5) R(n5,16) R(n5,n4) R(n6,n2) R(n6,n7) R(n7,15) R(n7,n6)

-------------------------------------------------------------------------------------------------------------

-> form outgroup SOL2 - A

score = 264:

model 11:

-------------

R(0,1) R(0,9) R(0,10) R(0,17) R(0,18) R(0,24) R(0,25) R(0,28) R(0,n1) R(0,n2) R(1,0) R(2,17) R(3,19) R(3,20) R(4,21) R(5,23) R(6,n1) R(6,n3) R(7,n3) R(8,24) R(9,0) R(10,0) R(11,27) R(12,31) R(13,14) R(13,15) R(13,n4) R(14,13) R(15,13) R(15,n7) R(16,n5) R(17,0) R(17,2) R(18,0) R(18,19) R(19,3) R(19,18) R(19,22) R(20,3) R(20,21) R(21,4) R(21,20) R(22,19) R(22,23) R(23,5) R(23,22) R(24,0) R(24,8) R(25,0) R(25,26) R(26,25) R(26,27) R(27,11) R(27,26) R(28,0) R(28,29) R(29,28) R(29,30) R(30,29) R(30,31) R(31,12) R(31,30) R(n1,0) R(n1,6) R(n2,0) R(n2,n6) R(n3,6) R(n3,7) R(n4,13) R(n4,n6) R(n5,16) R(n5,n7) R(n6,n2) R(n6,n4) R(n7,15) R(n7,n5)

-------------------------------------------------------------------------------------------------------------

-> form outgroup SOL2 - A

n4(mod12)

g1: [ cox1 cox2 -nad4 -nad5 -nad4L -cob -nad6 nad1 nad3 nad2 cox3 rrnS rrnL ]

g2: [ cox1 cox2 -nad4 -nad5 -nad4L -cob -nad6 rrnL nad1 nad3 nad2 cox3 rrnS ]

g3: [ cox1 cox2 nad1 nad3 nad2 -nad4 -nad5 -nad4L -cob -nad6 cox3 rrnS rrnL ]

g4: [ cox1 cox2 nad1 nad3 nad2 cox3 rrnS -nad5 -nad4 -nad4L -cob -nad6 rrnL ]

g5: [ cox1 cox2 nad1 nad6 cob nad4L nad5 nad4 nad3 nad2 cox3 rrnS rrnL ]

score = 240:

model 12:

-------------

R(0,1) R(0,9) R(0,10) R(0,17) R(0,18) R(0,24) R(0,25) R(0,28) R(0,n1) R(0,n2) R(1,0) R(2,17) R(3,19) R(3,20) R(4,21) R(5,23) R(6,n1) R(6,n3) R(7,n3) R(8,24) R(9,0) R(10,0) R(11,27) R(12,31) R(13,14) R(13,15) R(13,n4) R(14,13) R(15,13) R(16,n5) R(17,0) R(17,2) R(18,0) R(18,19) R(19,3) R(19,18) R(19,22) R(20,3) R(20,21) R(21,4) R(21,20) R(22,19) R(22,23) R(23,5) R(23,22) R(24,0) R(24,8) R(25,0) R(25,26) R(26,25) R(26,27) R(27,11) R(27,26) R(28,0) R(28,29) R(29,28) R(29,30) R(30,29) R(30,31) R(31,12) R(31,30) R(n1,0) R(n1,6) R(n2,0) R(n2,n6) R(n3,6) R(n3,7) R(n4,13) R(n4,n6) R(n4,n7) R(n5,16) R(n5,n7) R(n6,n2) R(n6,n4) R(n7,n4) R(n7,n5)

-------------------------------------------------------------------------------------------------------------

-> form outgroup SOL1 - A

n3(mod13)

[ cox1 cox2 atp8 atp6 cox3 nad3 -nad5 -nad4 -nad4L nad6 cob rrnS rrnL nad1 nad2 ]

score = 264:

model 13:

-------------

R(0,1) R(0,9) R(0,10) R(0,17) R(0,18) R(0,24) R(0,25) R(0,28) R(0,n1) R(0,n2) R(1,0) R(2,17) R(3,19) R(3,20) R(4,21) R(5,23) R(6,n3) R(7,n3) R(8,24) R(9,0) R(10,0) R(11,27) R(12,31) R(13,14) R(13,15) R(13,n4) R(14,13) R(15,13) R(15,n6) R(16,n5) R(17,0) R(17,2) R(18,0) R(18,19) R(19,3) R(19,18) R(19,22) R(20,3) R(20,21) R(21,4) R(21,20) R(22,19) R(22,23) R(23,5) R(23,22) R(24,0) R(24,8) R(25,0) R(25,26) R(26,25) R(26,27) R(27,11) R(27,26) R(28,0) R(28,29) R(29,28) R(29,30) R(30,29) R(30,31) R(31,12) R(31,30) R(n1,0) R(n1,n7) R(n2,0) R(n2,n3) R(n3,6) R(n3,7) R(n3,n2) R(n4,13) R(n4,n7) R(n5,16) R(n5,n6) R(n6,15) R(n6,n5) R(n7,n1) R(n7,n4)

-------------------------------------------------------------------------------------------------------------

-> form outgroup SOL1 - A

n3(mod14)

[ cox1 cox2 atp8 atp6 cox3 nad3 -nad5 -nad4 -nad4L nad6 cob rrnS rrnL nad1 nad2 ]

n4(mod14)

g1: [ cox1 cox2 -nad4 -nad5 -nad4L -cob -nad6 nad1 nad3 nad2 cox3 rrnS rrnL ]

g2: [ cox1 cox2 -nad4 -nad5 -nad4L -cob -nad6 rrnL nad1 nad3 nad2 cox3 rrnS ]

g3: [ cox1 cox2 nad1 nad3 nad2 -nad4 -nad5 -nad4L -cob -nad6 cox3 rrnS rrnL ]

g4: [ cox1 cox2 nad1 nad3 nad2 cox3 rrnS -nad5 -nad4 -nad4L -cob -nad6 rrnL ]

g5: [ cox1 cox2 nad1 nad6 cob nad4L nad5 nad4 nad3 nad2 cox3 rrnS rrnL ]

score = 240:

model 14:

-------------

R(0,1) R(0,9) R(0,10) R(0,17) R(0,18) R(0,24) R(0,25) R(0,28) R(0,n1) R(0,n2) R(1,0) R(2,17) R(3,19) R(3,20) R(4,21) R(5,23) R(6,n3) R(7,n3) R(8,24) R(9,0) R(10,0) R(11,27) R(12,31) R(13,14) R(13,15) R(13,n4) R(14,13) R(15,13) R(16,n5) R(17,0) R(17,2) R(18,0) R(18,19) R(19,3) R(19,18) R(19,22) R(20,3) R(20,21) R(21,4) R(21,20) R(22,19) R(22,23) R(23,5) R(23,22) R(24,0) R(24,8) R(25,0) R(25,26) R(26,25) R(26,27) R(27,11) R(27,26) R(28,0) R(28,29) R(29,28) R(29,30) R(30,29) R(30,31) R(31,12) R(31,30) R(n1,0) R(n1,n3) R(n2,0) R(n2,n7) R(n3,6) R(n3,7) R(n3,n1) R(n4,13) R(n4,n6) R(n4,n7) R(n5,16) R(n5,n6) R(n6,n4) R(n6,n5) R(n7,n2) R(n7,n4)

-------------------------------------------------------------------------------------------------------------

-> form outgroup SOL2 - A

n4(mod15)

[ cox1 cox2 nad1 nad3 nad2 cox3 rrnS -nad4L -nad4 -nad5 -cob -nad6 rrnL ]

score = 290:

model 15:

-------------

R(0,1) R(0,9) R(0,10) R(0,17) R(0,18) R(0,24) R(0,25) R(0,28) R(0,n1) R(0,n2) R(1,0) R(2,17) R(3,19) R(3,20) R(4,21) R(5,23) R(6,n2) R(6,n3) R(7,n3) R(8,24) R(9,0) R(10,0) R(11,27) R(12,31) R(13,14) R(13,n4) R(14,13) R(15,n4) R(15,n5) R(16,n6) R(17,0) R(17,2) R(18,0) R(18,19) R(19,3) R(19,18) R(19,22) R(20,3) R(20,21) R(21,4) R(21,20) R(22,19) R(22,23) R(23,5) R(23,22) R(24,0) R(24,8) R(25,0) R(25,26) R(26,25) R(26,27) R(27,11) R(27,26) R(28,0) R(28,29) R(29,28) R(29,30) R(30,29) R(30,31) R(31,12) R(31,30) R(n1,0) R(n1,n7) R(n2,0) R(n2,6) R(n3,6) R(n3,7) R(n4,13) R(n4,15) R(n4,n6) R(n5,15) R(n5,n7) R(n6,16) R(n6,n4) R(n7,n1) R(n7,n5)

-------------------------------------------------------------------------------------------------------------

-> form outgroup SOL1 - A

n3(mod16)

[ cox1 cox2 atp8 atp6 cox3 nad3 -nad5 -nad4 -nad4L nad6 cob rrnS rrnL nad1 nad2 ]

n4(mod16)

[ cox1 cox2 nad1 nad3 nad2 cox3 rrnS -nad4L -nad4 -nad5 -cob -nad6 rrnL ]

score = 290:

model 16:

-------------

R(0,1) R(0,9) R(0,10) R(0,17) R(0,18) R(0,24) R(0,25) R(0,28) R(0,n1) R(0,n2) R(1,0) R(2,17) R(3,19) R(3,20) R(4,21) R(5,23) R(6,n3) R(7,n3) R(8,24) R(9,0) R(10,0) R(11,27) R(12,31) R(13,14) R(13,n4) R(14,13) R(15,n4) R(15,n5) R(16,n6) R(17,0) R(17,2) R(18,0) R(18,19) R(19,3) R(19,18) R(19,22) R(20,3) R(20,21) R(21,4) R(21,20) R(22,19) R(22,23) R(23,5) R(23,22) R(24,0) R(24,8) R(25,0) R(25,26) R(26,25) R(26,27) R(27,11) R(27,26) R(28,0) R(28,29) R(29,28) R(29,30) R(30,29) R(30,31) R(31,12) R(31,30) R(n1,0) R(n1,n7) R(n2,0) R(n2,n3) R(n3,6) R(n3,7) R(n3,n2) R(n4,13) R(n4,15) R(n4,n6) R(n5,15) R(n5,n7) R(n6,16) R(n6,n4) R(n7,n1) R(n7,n5)

-------------------------------------------------------------------------------------------------------------

-> form outgroup SOL2 - A

n4(mod17)

[ cox1 cox2 nad1 nad3 nad2 cox3 rrnS -nad5 -nad4 -nad4L -cob -nad6 rrnL ]

score = 242:

model 17:

-------------

R(0,1) R(0,9) R(0,10) R(0,17) R(0,18) R(0,24) R(0,25) R(0,28) R(0,n1) R(0,n2) R(1,0) R(2,17) R(3,19) R(3,20) R(4,21) R(5,23) R(6,n1) R(6,n3) R(7,n3) R(8,24) R(9,0) R(10,0) R(11,27) R(12,31) R(13,14) R(13,n4) R(14,13) R(15,n4) R(15,n5) R(16,n7) R(17,0) R(17,2) R(18,0) R(18,19) R(19,3) R(19,18) R(19,22) R(20,3) R(20,21) R(21,4) R(21,20) R(22,19) R(22,23) R(23,5) R(23,22) R(24,0) R(24,8) R(25,0) R(25,26) R(26,25) R(26,27) R(27,11) R(27,26) R(28,0) R(28,29) R(29,28) R(29,30) R(30,29) R(30,31) R(31,12) R(31,30) R(n1,0) R(n1,6) R(n2,0) R(n2,n6) R(n3,6) R(n3,7) R(n4,13) R(n4,15) R(n4,n6) R(n5,15) R(n5,n7) R(n6,n2) R(n6,n4) R(n7,16) R(n7,n5)

-------------------------------------------------------------------------------------------------------------

-> form outgroup SOL1 - A

n3(mod18)

[ cox1 cox2 atp8 atp6 cox3 nad3 -nad5 -nad4 -nad4L nad6 cob rrnS rrnL nad1 nad2 ]

n4(mod18)

[ cox1 cox2 nad1 nad3 nad2 cox3 rrnS -nad5 -nad4 -nad4L -cob -nad6 rrnL ]

score = 242:

model 18:

-------------

R(0,1) R(0,9) R(0,10) R(0,17) R(0,18) R(0,24) R(0,25) R(0,28) R(0,n1) R(0,n2) R(1,0) R(2,17) R(3,19) R(3,20) R(4,21) R(5,23) R(6,n3) R(7,n3) R(8,24) R(9,0) R(10,0) R(11,27) R(12,31) R(13,14) R(13,n4) R(14,13) R(15,n4) R(15,n5) R(16,n7) R(17,0) R(17,2) R(18,0) R(18,19) R(19,3) R(19,18) R(19,22) R(20,3) R(20,21) R(21,4) R(21,20) R(22,19) R(22,23) R(23,5) R(23,22) R(24,0) R(24,8) R(25,0) R(25,26) R(26,25) R(26,27) R(27,11) R(27,26) R(28,0) R(28,29) R(29,28) R(29,30) R(30,29) R(30,31) R(31,12) R(31,30) R(n1,0) R(n1,n3) R(n2,0) R(n2,n6) R(n3,6) R(n3,7) R(n3,n1) R(n4,13) R(n4,15) R(n4,n6) R(n5,15) R(n5,n7) R(n6,n2) R(n6,n4) R(n7,16) R(n7,n5)

-------------------------------------------------------------------------------------------------------------

-> form outgroup SOL2 - A

n4(mod19)

[ cox1 cox2 nad1 nad3 nad2 cox3 rrnS -nad5 -nad4 -nad4L -cob -nad6 rrnL ]

score = 228:

model 19:

-------------

R(0,1) R(0,9) R(0,10) R(0,17) R(0,18) R(0,24) R(0,25) R(0,28) R(0,n1) R(0,n2) R(1,0) R(2,17) R(3,19) R(3,20) R(4,21) R(5,23) R(6,n1) R(6,n3) R(7,n3) R(8,24) R(9,0) R(10,0) R(11,27) R(12,31) R(13,14) R(13,n4) R(14,13) R(15,n4) R(16,n5) R(17,0) R(17,2) R(18,0) R(18,19) R(19,3) R(19,18) R(19,22) R(20,3) R(20,21) R(21,4) R(21,20) R(22,19) R(22,23) R(23,5) R(23,22) R(24,0) R(24,8) R(25,0) R(25,26) R(26,25) R(26,27) R(27,11) R(27,26) R(28,0) R(28,29) R(29,28) R(29,30) R(30,29) R(30,31) R(31,12) R(31,30) R(n1,0) R(n1,6) R(n2,0) R(n2,n6) R(n3,6) R(n3,7) R(n4,13) R(n4,15) R(n4,n6) R(n4,n7) R(n5,16) R(n5,n7) R(n6,n2) R(n6,n4) R(n7,n4) R(n7,n5)

-------------------------------------------------------------------------------------------------------------

-> form outgroup SOL1 - A

n3(mod20)

[ cox1 cox2 atp8 atp6 cox3 nad3 -nad5 -nad4 -nad4L nad6 cob rrnS rrnL nad1 nad2 ]

n4(mod20)

[ cox1 cox2 nad1 nad3 nad2 cox3 rrnS -nad5 -nad4 -nad4L -cob -nad6 rrnL ]

score = 228:

model 20:

-------------

R(0,1) R(0,9) R(0,10) R(0,17) R(0,18) R(0,24) R(0,25) R(0,28) R(0,n1) R(0,n2) R(1,0) R(2,17) R(3,19) R(3,20) R(4,21) R(5,23) R(6,n3) R(7,n3) R(8,24) R(9,0) R(10,0) R(11,27) R(12,31) R(13,14) R(13,n4) R(14,13) R(15,n4) R(16,n5) R(17,0) R(17,2) R(18,0) R(18,19) R(19,3) R(19,18) R(19,22) R(20,3) R(20,21) R(21,4) R(21,20) R(22,19) R(22,23) R(23,5) R(23,22) R(24,0) R(24,8) R(25,0) R(25,26) R(26,25) R(26,27) R(27,11) R(27,26) R(28,0) R(28,29) R(29,28) R(29,30) R(30,29) R(30,31) R(31,12) R(31,30) R(n1,0) R(n1,n3) R(n2,0) R(n2,n6) R(n3,6) R(n3,7) R(n3,n1) R(n4,13) R(n4,15) R(n4,n6) R(n4,n7) R(n5,16) R(n5,n7) R(n6,n2) R(n6,n4) R(n7,n4) R(n7,n5)

-------------------------------------------------------------------------------------------------------------

-> form outgroup SOL2 - A

n5(mod21)

[ cox1 cox2 nad1 nad3 nad2 cox3 rrnS -nad5 -nad4 -nad4L -cob -nad6 rrnL ]

n4(mod21)

[ cox1 cox2 nad1 nad3 nad2 cox3 rrnS nad5 nad4 -nad4L -cob -nad6 rrnL ]

score = 254:

model 21:

-------------

R(0,1) R(0,9) R(0,10) R(0,17) R(0,18) R(0,24) R(0,25) R(0,28) R(0,n1) R(0,n2) R(1,0) R(2,17) R(3,19) R(3,20) R(4,21) R(5,23) R(6,n2) R(6,n3) R(7,n3) R(8,24) R(9,0) R(10,0) R(11,27) R(12,31) R(13,14) R(13,n4) R(14,13) R(15,n5) R(16,n6) R(17,0) R(17,2) R(18,0) R(18,19) R(19,3) R(19,18) R(19,22) R(20,3) R(20,21) R(21,4) R(21,20) R(22,19) R(22,23) R(23,5) R(23,22) R(24,0) R(24,8) R(25,0) R(25,26) R(26,25) R(26,27) R(27,11) R(27,26) R(28,0) R(28,29) R(29,28) R(29,30) R(30,29) R(30,31) R(31,12) R(31,30) R(n1,0) R(n1,n7) R(n2,0) R(n2,6) R(n3,6) R(n3,7) R(n4,13) R(n4,n5) R(n4,n6) R(n5,15) R(n5,n4) R(n5,n7) R(n6,16) R(n6,n4) R(n7,n1) R(n7,n5)

-------------------------------------------------------------------------------------------------------------

-> form outgroup SOL1 - A

n3(mod22)

[ cox1 cox2 atp8 atp6 cox3 nad3 -nad5 -nad4 -nad4L nad6 cob rrnS rrnL nad1 nad2 ]

n5(mod22)

[ cox1 cox2 nad1 nad3 nad2 cox3 rrnS -nad5 -nad4 -nad4L -cob -nad6 rrnL ]

n4(mod22)

[ cox1 cox2 nad1 nad3 nad2 cox3 rrnS nad5 nad4 -nad4L -cob -nad6 rrnL ]

score = 254:

model 22:

-------------

R(0,1) R(0,9) R(0,10) R(0,17) R(0,18) R(0,24) R(0,25) R(0,28) R(0,n1) R(0,n2) R(1,0) R(2,17) R(3,19) R(3,20) R(4,21) R(5,23) R(6,n3) R(7,n3) R(8,24) R(9,0) R(10,0) R(11,27) R(12,31) R(13,14) R(13,n4) R(14,13) R(15,n5) R(16,n6) R(17,0) R(17,2) R(18,0) R(18,19) R(19,3) R(19,18) R(19,22) R(20,3) R(20,21) R(21,4) R(21,20) R(22,19) R(22,23) R(23,5) R(23,22) R(24,0) R(24,8) R(25,0) R(25,26) R(26,25) R(26,27) R(27,11) R(27,26) R(28,0) R(28,29) R(29,28) R(29,30) R(30,29) R(30,31) R(31,12) R(31,30) R(n1,0) R(n1,n7) R(n2,0) R(n2,n3) R(n3,6) R(n3,7) R(n3,n2) R(n4,13) R(n4,n5) R(n4,n6) R(n5,15) R(n5,n4) R(n5,n7) R(n6,16) R(n6,n4) R(n7,n1) R(n7,n5)

-------------------------------------------------------------------------------------------------------------

-> form outgroup SOL2 - A

score = 264:

model 23:

-------------

R(0,1) R(0,9) R(0,10) R(0,17) R(0,18) R(0,24) R(0,25) R(0,28) R(0,n1) R(0,n2) R(1,0) R(2,17) R(3,19) R(3,20) R(4,21) R(5,23) R(6,n2) R(6,n3) R(7,n3) R(8,24) R(9,0) R(10,0) R(11,27) R(12,31) R(13,14) R(13,15) R(14,13) R(15,13) R(15,n4) R(15,n5) R(16,n6) R(17,0) R(17,2) R(18,0) R(18,19) R(19,3) R(19,18) R(19,22) R(20,3) R(20,21) R(21,4) R(21,20) R(22,19) R(22,23) R(23,5) R(23,22) R(24,0) R(24,8) R(25,0) R(25,26) R(26,25) R(26,27) R(27,11) R(27,26) R(28,0) R(28,29) R(29,28) R(29,30) R(30,29) R(30,31) R(31,12) R(31,30) R(n1,0) R(n1,n7) R(n2,0) R(n2,6) R(n3,6) R(n3,7) R(n4,15) R(n4,n6) R(n5,15) R(n5,n7) R(n6,16) R(n6,n4) R(n7,n1) R(n7,n5)

-------------------------------------------------------------------------------------------------------------

-> form outgroup SOL1 - A

n3(mod24)

[ cox1 cox2 atp8 atp6 cox3 nad3 -nad5 -nad4 -nad4L nad6 cob rrnS rrnL nad1 nad2 ]

score = 264:

model 24:

-------------

R(0,1) R(0,9) R(0,10) R(0,17) R(0,18) R(0,24) R(0,25) R(0,28) R(0,n1) R(0,n2) R(1,0) R(2,17) R(3,19) R(3,20) R(4,21) R(5,23) R(6,n3) R(7,n3) R(8,24) R(9,0) R(10,0) R(11,27) R(12,31) R(13,14) R(13,15) R(14,13) R(15,13) R(15,n4) R(15,n5) R(16,n6) R(17,0) R(17,2) R(18,0) R(18,19) R(19,3) R(19,18) R(19,22) R(20,3) R(20,21) R(21,4) R(21,20) R(22,19) R(22,23) R(23,5) R(23,22) R(24,0) R(24,8) R(25,0) R(25,26) R(26,25) R(26,27) R(27,11) R(27,26) R(28,0) R(28,29) R(29,28) R(29,30) R(30,29) R(30,31) R(31,12) R(31,30) R(n1,0) R(n1,n7) R(n2,0) R(n2,n3) R(n3,6) R(n3,7) R(n3,n2) R(n4,15) R(n4,n6) R(n5,15) R(n5,n7) R(n6,16) R(n6,n4) R(n7,n1) R(n7,n5)

-------------------------------------------------------------------------------------------------------------

-> form outgroup SOL2 - A

n4(mod25)

[ cox1 cox2 nad1 nad3 nad2 cox3 rrnS -nad5 -nad4 -nad4L -cob -nad6 rrnL ]

score = 254:

model 25:

-------------

R(0,1) R(0,9) R(0,10) R(0,17) R(0,18) R(0,24) R(0,25) R(0,28) R(0,n1) R(0,n2) R(1,0) R(2,17) R(3,19) R(3,20) R(4,21) R(5,23) R(6,n1) R(6,n3) R(7,n3) R(8,24) R(9,0) R(10,0) R(11,27) R(12,31) R(13,14) R(13,15) R(14,13) R(15,13) R(15,n4) R(16,n5) R(17,0) R(17,2) R(18,0) R(18,19) R(19,3) R(19,18) R(19,22) R(20,3) R(20,21) R(21,4) R(21,20) R(22,19) R(22,23) R(23,5) R(23,22) R(24,0) R(24,8) R(25,0) R(25,26) R(26,25) R(26,27) R(27,11) R(27,26) R(28,0) R(28,29) R(29,28) R(29,30) R(30,29) R(30,31) R(31,12) R(31,30) R(n1,0) R(n1,6) R(n2,0) R(n2,n6) R(n3,6) R(n3,7) R(n4,15) R(n4,n6) R(n4,n7) R(n5,16) R(n5,n7) R(n6,n2) R(n6,n4) R(n7,n4) R(n7,n5)

-------------------------------------------------------------------------------------------------------------

-> form outgroup SOL1 - A

n3(mod26)

[ cox1 cox2 atp8 atp6 cox3 nad3 -nad5 -nad4 -nad4L nad6 cob rrnS rrnL nad1 nad2 ]

n4(mod26)

[ cox1 cox2 nad1 nad3 nad2 cox3 rrnS -nad5 -nad4 -nad4L -cob -nad6 rrnL ]

score = 254:

model 26:

-------------

R(0,1) R(0,9) R(0,10) R(0,17) R(0,18) R(0,24) R(0,25) R(0,28) R(0,n1) R(0,n2) R(1,0) R(2,17) R(3,19) R(3,20) R(4,21) R(5,23) R(6,n3) R(7,n3) R(8,24) R(9,0) R(10,0) R(11,27) R(12,31) R(13,14) R(13,15) R(14,13) R(15,13) R(15,n4) R(16,n5) R(17,0) R(17,2) R(18,0) R(18,19) R(19,3) R(19,18) R(19,22) R(20,3) R(20,21) R(21,4) R(21,20) R(22,19) R(22,23) R(23,5) R(23,22) R(24,0) R(24,8) R(25,0) R(25,26) R(26,25) R(26,27) R(27,11) R(27,26) R(28,0) R(28,29) R(29,28) R(29,30) R(30,29) R(30,31) R(31,12) R(31,30) R(n1,0) R(n1,n3) R(n2,0) R(n2,n6) R(n3,6) R(n3,7) R(n3,n1) R(n4,15) R(n4,n6) R(n4,n7) R(n5,16) R(n5,n7) R(n6,n2) R(n6,n4) R(n7,n4) R(n7,n5)

-------------------------------------------------------------------------------------------------------------

-> form outgroup SOL3 - A

n2(mod27)

[ cox1 cox2 atp8 atp6 cox3 nad3 -nad5 -nad4 -nad4L -cob -nad6 -nad1 -rrnL -rrnS nad2 ]

score = 236:

model 27:

-------------

R(0,1) R(0,9) R(0,10) R(0,17) R(0,18) R(0,24) R(0,25) R(0,28) R(0,n1) R(0,n2) R(1,0) R(2,17) R(3,19) R(3,20) R(4,21) R(5,23) R(6,n2) R(7,n3) R(8,24) R(9,0) R(10,0) R(11,27) R(12,31) R(13,14) R(13,15) R(13,n4) R(13,n5) R(14,13) R(15,13) R(16,n6) R(17,0) R(17,2) R(18,0) R(18,19) R(19,3) R(19,18) R(19,22) R(20,3) R(20,21) R(21,4) R(21,20) R(22,19) R(22,23) R(23,5) R(23,22) R(24,0) R(24,8) R(25,0) R(25,26) R(26,25) R(26,27) R(27,11) R(27,26) R(28,0) R(28,29) R(29,28) R(29,30) R(30,29) R(30,31) R(31,12) R(31,30) R(n1,0) R(n1,n7) R(n2,0) R(n2,6) R(n2,n3) R(n3,7) R(n3,n2) R(n4,13) R(n4,n6) R(n5,13) R(n5,n7) R(n6,16) R(n6,n4) R(n7,n1) R(n7,n5)

-------------------------------------------------------------------------------------------------------------

-> form outgroup SOL3 - A

n2(mod28)

[ cox1 cox2 atp8 atp6 cox3 nad3 -nad5 -nad4 -nad4L -cob -nad6 -nad1 -rrnL -rrnS nad2 ]

n4(mod28)

[ cox1 cox2 nad1 nad3 nad2 cox3 rrnS -nad4L -nad4 -nad5 -cob -nad6 rrnL ]

score = 250:

model 28:

-------------

R(0,1) R(0,9) R(0,10) R(0,17) R(0,18) R(0,24) R(0,25) R(0,28) R(0,n1) R(0,n2) R(1,0) R(2,17) R(3,19) R(3,20) R(4,21) R(5,23) R(6,n2) R(7,n3) R(8,24) R(9,0) R(10,0) R(11,27) R(12,31) R(13,14) R(13,n4) R(13,n5) R(14,13) R(15,n4) R(16,n6) R(17,0) R(17,2) R(18,0) R(18,19) R(19,3) R(19,18) R(19,22) R(20,3) R(20,21) R(21,4) R(21,20) R(22,19) R(22,23) R(23,5) R(23,22) R(24,0) R(24,8) R(25,0) R(25,26) R(26,25) R(26,27) R(27,11) R(27,26) R(28,0) R(28,29) R(29,28) R(29,30) R(30,29) R(30,31) R(31,12) R(31,30) R(n1,0) R(n1,n7) R(n2,0) R(n2,6) R(n2,n3) R(n3,7) R(n3,n2) R(n4,13) R(n4,15) R(n4,n6) R(n5,13) R(n5,n7) R(n6,16) R(n6,n4) R(n7,n1) R(n7,n5)

-------------------------------------------------------------------------------------------------------------

-> form outgroup SOL3 - A

n1(mod29)

[ cox1 cox2 atp8 atp6 cox3 nad3 -nad5 -nad4 -nad4L -cob -nad6 -nad1 -rrnL -rrnS nad2 ]

n4(mod29)

[ cox1 cox2 nad1 nad3 nad2 cox3 rrnS -nad5 -nad4 -nad4L -cob -nad6 rrnL ]

score = 226:

model 29:

-------------

R(0,1) R(0,9) R(0,10) R(0,17) R(0,18) R(0,24) R(0,25) R(0,28) R(0,n1) R(0,n2) R(1,0) R(2,17) R(3,19) R(3,20) R(4,21) R(5,23) R(6,n1) R(7,n3) R(8,24) R(9,0) R(10,0) R(11,27) R(12,31) R(13,14) R(13,n4) R(13,n5) R(14,13) R(15,n4) R(16,n7) R(17,0) R(17,2) R(18,0) R(18,19) R(19,3) R(19,18) R(19,22) R(20,3) R(20,21) R(21,4) R(21,20) R(22,19) R(22,23) R(23,5) R(23,22) R(24,0) R(24,8) R(25,0) R(25,26) R(26,25) R(26,27) R(27,11) R(27,26) R(28,0) R(28,29) R(29,28) R(29,30) R(30,29) R(30,31) R(31,12) R(31,30) R(n1,0) R(n1,6) R(n1,n3) R(n2,0) R(n2,n6) R(n3,7) R(n3,n1) R(n4,13) R(n4,15) R(n4,n6) R(n5,13) R(n5,n7) R(n6,n2) R(n6,n4) R(n7,16) R(n7,n5)

-------------------------------------------------------------------------------------------------------------

-> form outgroup SOL3 - A

n2(mod30)

[ cox1 cox2 atp8 atp6 cox3 nad3 -nad5 -nad4 -nad4L -cob -nad6 -nad1 -rrnL -rrnS nad2 ]

n4(mod30)

g1: [ cox1 cox2 nad1 nad3 nad2 cox3 rrnS -nad4 -nad5 -cob -nad6 -nad4L rrnL ]

g2: [ cox1 cox2 nad1 nad3 nad2 cox3 rrnS -nad4 -nad5 -cob -nad6 rrnL -nad4L ]

g3: [ cox1 cox2 nad1 nad3 nad2 cox3 rrnS nad5 nad4 -nad4L -cob -nad6 rrnL ]

g4: [ cox1 cox2 nad1 nad3 nad2 cox3 rrnS rrnL nad6 cob nad4L nad5 nad4 ]

g5: [ cox1 cox2 nad1 nad3 nad2 cox3 rrnS -rrnL nad6 cob nad4L nad5 nad4 ]

g6: [ cox1 cox2 nad1 nad3 nad2 cox3 rrnS -nad4 -nad5 rrnL nad6 cob nad4L ]

g7: [ cox1 cox2 nad1 nad3 nad2 cox3 rrnS nad6 cob nad4L -nad4 -nad5 rrnL ]

g8: [ cox1 cox2 nad1 nad3 nad2 cox3 rrnS -nad4 -nad5 nad6 cob nad4L rrnL ]

g9: [ cox1 cox2 nad1 nad3 nad2 cox3 rrnS -rrnL nad6 cob nad4L -nad4 -nad5 ]

g10: [ cox1 cox2 nad1 nad3 nad2 cox3 rrnS -nad4 -nad5 -rrnL -nad4L -cob -nad6 ]

g11: [ cox1 cox2 nad1 nad3 nad2 cox3 rrnS -nad4 -nad5 -nad4L -cob -nad6 -rrnL ]

score = 262:

model 30:

-------------

R(0,1) R(0,9) R(0,10) R(0,17) R(0,18) R(0,24) R(0,25) R(0,28) R(0,n1) R(0,n2) R(1,0) R(2,17) R(3,19) R(3,20) R(4,21) R(5,23) R(6,n2) R(7,n3) R(8,24) R(9,0) R(10,0) R(11,27) R(12,31) R(13,14) R(13,15) R(13,n4) R(14,13) R(15,13) R(16,n5) R(17,0) R(17,2) R(18,0) R(18,19) R(19,3) R(19,18) R(19,22) R(20,3) R(20,21) R(21,4) R(21,20) R(22,19) R(22,23) R(23,5) R(23,22) R(24,0) R(24,8) R(25,0) R(25,26) R(26,25) R(26,27) R(27,11) R(27,26) R(28,0) R(28,29) R(29,28) R(29,30) R(30,29) R(30,31) R(31,12) R(31,30) R(n1,0) R(n1,n7) R(n2,0) R(n2,6) R(n2,n3) R(n3,7) R(n3,n2) R(n4,13) R(n4,n5) R(n4,n6) R(n5,16) R(n5,n4) R(n6,n4) R(n6,n7) R(n7,n1) R(n7,n6)

-------------------------------------------------------------------------------------------------------------

-> form outgroup SOL3 - A

n1(mod31)

[ cox1 cox2 atp8 atp6 cox3 nad3 -nad5 -nad4 -nad4L -cob -nad6 -nad1 -rrnL -rrnS nad2 ]

score = 262:

model 31:

-------------

R(0,1) R(0,9) R(0,10) R(0,17) R(0,18) R(0,24) R(0,25) R(0,28) R(0,n1) R(0,n2) R(1,0) R(2,17) R(3,19) R(3,20) R(4,21) R(5,23) R(6,n1) R(7,n3) R(8,24) R(9,0) R(10,0) R(11,27) R(12,31) R(13,14) R(13,15) R(13,n4) R(14,13) R(15,13) R(15,n7) R(16,n5) R(17,0) R(17,2) R(18,0) R(18,19) R(19,3) R(19,18) R(19,22) R(20,3) R(20,21) R(21,4) R(21,20) R(22,19) R(22,23) R(23,5) R(23,22) R(24,0) R(24,8) R(25,0) R(25,26) R(26,25) R(26,27) R(27,11) R(27,26) R(28,0) R(28,29) R(29,28) R(29,30) R(30,29) R(30,31) R(31,12) R(31,30) R(n1,0) R(n1,6) R(n1,n3) R(n2,0) R(n2,n6) R(n3,7) R(n3,n1) R(n4,13) R(n4,n5) R(n5,16) R(n5,n4) R(n6,n2) R(n6,n7) R(n7,15) R(n7,n6)

-------------------------------------------------------------------------------------------------------------

-> form outgroup SOL3 - A

n1(mod32)

[ cox1 cox2 atp8 atp6 cox3 nad3 -nad5 -nad4 -nad4L -cob -nad6 -nad1 -rrnL -rrnS nad2 ]

score = 250:

model 32:

-------------

R(0,1) R(0,9) R(0,10) R(0,17) R(0,18) R(0,24) R(0,25) R(0,28) R(0,n1) R(0,n2) R(1,0) R(2,17) R(3,19) R(3,20) R(4,21) R(5,23) R(6,n1) R(7,n3) R(8,24) R(9,0) R(10,0) R(11,27) R(12,31) R(13,14) R(13,15) R(13,n4) R(14,13) R(15,13) R(15,n7) R(16,n5) R(17,0) R(17,2) R(18,0) R(18,19) R(19,3) R(19,18) R(19,22) R(20,3) R(20,21) R(21,4) R(21,20) R(22,19) R(22,23) R(23,5) R(23,22) R(24,0) R(24,8) R(25,0) R(25,26) R(26,25) R(26,27) R(27,11) R(27,26) R(28,0) R(28,29) R(29,28) R(29,30) R(30,29) R(30,31) R(31,12) R(31,30) R(n1,0) R(n1,6) R(n1,n3) R(n2,0) R(n2,n6) R(n3,7) R(n3,n1) R(n4,13) R(n4,n6) R(n5,16) R(n5,n7) R(n6,n2) R(n6,n4) R(n7,15) R(n7,n5)

-------------------------------------------------------------------------------------------------------------

-> form outgroup SOL3 - A

n1(mod33)

[ cox1 cox2 atp8 atp6 cox3 nad3 -nad5 -nad4 -nad4L -cob -nad6 -nad1 -rrnL -rrnS nad2 ]

n4(mod33)

g1: [ cox1 cox2 -nad4 -nad5 -nad4L -cob -nad6 nad1 nad3 nad2 cox3 rrnS rrnL ]

g2: [ cox1 cox2 -nad4 -nad5 -nad4L -cob -nad6 rrnL nad1 nad3 nad2 cox3 rrnS ]

g3: [ cox1 cox2 nad1 nad3 nad2 -nad4 -nad5 -nad4L -cob -nad6 cox3 rrnS rrnL ]

g4: [ cox1 cox2 nad1 nad3 nad2 cox3 rrnS -nad5 -nad4 -nad4L -cob -nad6 rrnL ]

g5: [ cox1 cox2 nad1 nad6 cob nad4L nad5 nad4 nad3 nad2 cox3 rrnS rrnL ]

score = 226:

model 33:

-------------

R(0,1) R(0,9) R(0,10) R(0,17) R(0,18) R(0,24) R(0,25) R(0,28) R(0,n1) R(0,n2) R(1,0) R(2,17) R(3,19) R(3,20) R(4,21) R(5,23) R(6,n1) R(7,n3) R(8,24) R(9,0) R(10,0) R(11,27) R(12,31) R(13,14) R(13,15) R(13,n4) R(14,13) R(15,13) R(16,n5) R(17,0) R(17,2) R(18,0) R(18,19) R(19,3) R(19,18) R(19,22) R(20,3) R(20,21) R(21,4) R(21,20) R(22,19) R(22,23) R(23,5) R(23,22) R(24,0) R(24,8) R(25,0) R(25,26) R(26,25) R(26,27) R(27,11) R(27,26) R(28,0) R(28,29) R(29,28) R(29,30) R(30,29) R(30,31) R(31,12) R(31,30) R(n1,0) R(n1,6) R(n1,n3) R(n2,0) R(n2,n6) R(n3,7) R(n3,n1) R(n4,13) R(n4,n6) R(n4,n7) R(n5,16) R(n5,n7) R(n6,n2) R(n6,n4) R(n7,n4) R(n7,n5)

-------------------------------------------------------------------------------------------------------------

-> form outgroup SOL3 - A

n2(mod34)

[ cox1 cox2 atp8 atp6 cox3 nad3 -nad5 -nad4 -nad4L -cob -nad6 -nad1 -rrnL -rrnS nad2 ]

n4(mod34)

[ cox1 cox2 nad1 nad3 nad2 cox3 rrnS -nad4L -nad4 -nad5 -cob -nad6 rrnL ]

score = 276:

model 34:

-------------

R(0,1) R(0,9) R(0,10) R(0,17) R(0,18) R(0,24) R(0,25) R(0,28) R(0,n1) R(0,n2) R(1,0) R(2,17) R(3,19) R(3,20) R(4,21) R(5,23) R(6,n2) R(7,n3) R(8,24) R(9,0) R(10,0) R(11,27) R(12,31) R(13,14) R(13,n4) R(14,13) R(15,n4) R(15,n5) R(16,n6) R(17,0) R(17,2) R(18,0) R(18,19) R(19,3) R(19,18) R(19,22) R(20,3) R(20,21) R(21,4) R(21,20) R(22,19) R(22,23) R(23,5) R(23,22) R(24,0) R(24,8) R(25,0) R(25,26) R(26,25) R(26,27) R(27,11) R(27,26) R(28,0) R(28,29) R(29,28) R(29,30) R(30,29) R(30,31) R(31,12) R(31,30) R(n1,0) R(n1,n7) R(n2,0) R(n2,6) R(n2,n3) R(n3,7) R(n3,n2) R(n4,13) R(n4,15) R(n4,n6) R(n5,15) R(n5,n7) R(n6,16) R(n6,n4) R(n7,n1) R(n7,n5)

-------------------------------------------------------------------------------------------------------------

-> form outgroup SOL3 - A

n1(mod35)

[ cox1 cox2 atp8 atp6 cox3 nad3 -nad5 -nad4 -nad4L -cob -nad6 -nad1 -rrnL -rrnS nad2 ]

n4(mod35)

[ cox1 cox2 nad1 nad3 nad2 cox3 rrnS -nad5 -nad4 -nad4L -cob -nad6 rrnL ]

score = 228:

model 35:

-------------

R(0,1) R(0,9) R(0,10) R(0,17) R(0,18) R(0,24) R(0,25) R(0,28) R(0,n1) R(0,n2) R(1,0) R(2,17) R(3,19) R(3,20) R(4,21) R(5,23) R(6,n1) R(7,n3) R(8,24) R(9,0) R(10,0) R(11,27) R(12,31) R(13,14) R(13,n4) R(14,13) R(15,n4) R(15,n5) R(16,n7) R(17,0) R(17,2) R(18,0) R(18,19) R(19,3) R(19,18) R(19,22) R(20,3) R(20,21) R(21,4) R(21,20) R(22,19) R(22,23) R(23,5) R(23,22) R(24,0) R(24,8) R(25,0) R(25,26) R(26,25) R(26,27) R(27,11) R(27,26) R(28,0) R(28,29) R(29,28) R(29,30) R(30,29) R(30,31) R(31,12) R(31,30) R(n1,0) R(n1,6) R(n1,n3) R(n2,0) R(n2,n6) R(n3,7) R(n3,n1) R(n4,13) R(n4,15) R(n4,n6) R(n5,15) R(n5,n7) R(n6,n2) R(n6,n4) R(n7,16) R(n7,n5)

-------------------------------------------------------------------------------------------------------------

-> form outgroup SOL3 - A

-> the BEST model

n1(mod36)

[ cox1 cox2 atp8 atp6 cox3 nad3 -nad5 -nad4 -nad4L -cob -nad6 -nad1 -rrnL -rrnS nad2 ]

n4(mod36)

[ cox1 cox2 nad1 nad3 nad2 cox3 rrnS -nad5 -nad4 -nad4L -cob -nad6 rrnL ]

score = 214:

model 36:

-------------

R(0,1) R(0,9) R(0,10) R(0,17) R(0,18) R(0,24) R(0,25) R(0,28) R(0,n1) R(0,n2) R(1,0) R(2,17) R(3,19) R(3,20) R(4,21) R(5,23) R(6,n1) R(7,n3) R(8,24) R(9,0) R(10,0) R(11,27) R(12,31) R(13,14) R(13,n4) R(14,13) R(15,n4) R(16,n5) R(17,0) R(17,2) R(18,0) R(18,19) R(19,3) R(19,18) R(19,22) R(20,3) R(20,21) R(21,4) R(21,20) R(22,19) R(22,23) R(23,5) R(23,22) R(24,0) R(24,8) R(25,0) R(25,26) R(26,25) R(26,27) R(27,11) R(27,26) R(28,0) R(28,29) R(29,28) R(29,30) R(30,29) R(30,31) R(31,12) R(31,30) R(n1,0) R(n1,6) R(n1,n3) R(n2,0) R(n2,n6) R(n3,7) R(n3,n1) R(n4,13) R(n4,15) R(n4,n6) R(n4,n7) R(n5,16) R(n5,n7) R(n6,n2) R(n6,n4) R(n7,n4) R(n7,n5)

-------------------------------------------------------------------------------------------------------------

-> form outgroup SOL3 - A

n2(mod37)

[ cox1 cox2 atp8 atp6 cox3 nad3 -nad5 -nad4 -nad4L -cob -nad6 -nad1 -rrnL -rrnS nad2 ]

n5(mod37)

[ cox1 cox2 nad1 nad3 nad2 cox3 rrnS -nad5 -nad4 -nad4L -cob -nad6 rrnL ]

n4(mod37)

[ cox1 cox2 nad1 nad3 nad2 cox3 rrnS nad5 nad4 -nad4L -cob -nad6 rrnL ]

score = 240:

model 37:

-------------

R(0,1) R(0,9) R(0,10) R(0,17) R(0,18) R(0,24) R(0,25) R(0,28) R(0,n1) R(0,n2) R(1,0) R(2,17) R(3,19) R(3,20) R(4,21) R(5,23) R(6,n2) R(7,n3) R(8,24) R(9,0) R(10,0) R(11,27) R(12,31) R(13,14) R(13,n4) R(14,13) R(15,n5) R(16,n6) R(17,0) R(17,2) R(18,0) R(18,19) R(19,3) R(19,18) R(19,22) R(20,3) R(20,21) R(21,4) R(21,20) R(22,19) R(22,23) R(23,5) R(23,22) R(24,0) R(24,8) R(25,0) R(25,26) R(26,25) R(26,27) R(27,11) R(27,26) R(28,0) R(28,29) R(29,28) R(29,30) R(30,29) R(30,31) R(31,12) R(31,30) R(n1,0) R(n1,n7) R(n2,0) R(n2,6) R(n2,n3) R(n3,7) R(n3,n2) R(n4,13) R(n4,n5) R(n4,n6) R(n5,15) R(n5,n4) R(n5,n7) R(n6,16) R(n6,n4) R(n7,n1) R(n7,n5)

-------------------------------------------------------------------------------------------------------------

-> form outgroup SOL3 - A

n2(mod38)

[ cox1 cox2 atp8 atp6 cox3 nad3 -nad5 -nad4 -nad4L -cob -nad6 -nad1 -rrnL -rrnS nad2 ]

score = 250:

model 38:

-------------

R(0,1) R(0,9) R(0,10) R(0,17) R(0,18) R(0,24) R(0,25) R(0,28) R(0,n1) R(0,n2) R(1,0) R(2,17) R(3,19) R(3,20) R(4,21) R(5,23) R(6,n2) R(7,n3) R(8,24) R(9,0) R(10,0) R(11,27) R(12,31) R(13,14) R(13,15) R(14,13) R(15,13) R(15,n4) R(15,n5) R(16,n6) R(17,0) R(17,2) R(18,0) R(18,19) R(19,3) R(19,18) R(19,22) R(20,3) R(20,21) R(21,4) R(21,20) R(22,19) R(22,23) R(23,5) R(23,22) R(24,0) R(24,8) R(25,0) R(25,26) R(26,25) R(26,27) R(27,11) R(27,26) R(28,0) R(28,29) R(29,28) R(29,30) R(30,29) R(30,31) R(31,12) R(31,30) R(n1,0) R(n1,n7) R(n2,0) R(n2,6) R(n2,n3) R(n3,7) R(n3,n2) R(n4,15) R(n4,n6) R(n5,15) R(n5,n7) R(n6,16) R(n6,n4) R(n7,n1) R(n7,n5)

-------------------------------------------------------------------------------------------------------------

-> form outgroup SOL3 - A

n1(mod39)

[ cox1 cox2 atp8 atp6 cox3 nad3 -nad5 -nad4 -nad4L -cob -nad6 -nad1 -rrnL -rrnS nad2 ]

n4(mod39)

[ cox1 cox2 nad1 nad3 nad2 cox3 rrnS -nad5 -nad4 -nad4L -cob -nad6 rrnL ]

score = 240:

model 39:

-------------

R(0,1) R(0,9) R(0,10) R(0,17) R(0,18) R(0,24) R(0,25) R(0,28) R(0,n1) R(0,n2) R(1,0) R(2,17) R(3,19) R(3,20) R(4,21) R(5,23) R(6,n1) R(7,n3) R(8,24) R(9,0) R(10,0) R(11,27) R(12,31) R(13,14) R(13,15) R(14,13) R(15,13) R(15,n4) R(16,n5) R(17,0) R(17,2) R(18,0) R(18,19) R(19,3) R(19,18) R(19,22) R(20,3) R(20,21) R(21,4) R(21,20) R(22,19) R(22,23) R(23,5) R(23,22) R(24,0) R(24,8) R(25,0) R(25,26) R(26,25) R(26,27) R(27,11) R(27,26) R(28,0) R(28,29) R(29,28) R(29,30) R(30,29) R(30,31) R(31,12) R(31,30) R(n1,0) R(n1,6) R(n1,n3) R(n2,0) R(n2,n6) R(n3,7) R(n3,n1) R(n4,15) R(n4,n6) R(n4,n7) R(n5,16) R(n5,n7) R(n6,n2) R(n6,n4) R(n7,n4) R(n7,n5)

-------------------------------------------------------------------------------------------------------------

-> form outgroup SOL4 - A

n4(mod40)

[ cox1 cox2 atp8 atp6 cox3 nad3 -nad5 -nad4 -nad4L nad6 cob rrnS rrnL nad1 nad2 ]

n1(mod40)15g

[ cox1 cox2 atp8 atp6 cox3 rrnS rrnL nad1 nad6 cob nad4L nad4 nad5 nad3 nad2 ]

n1(mod40)13g

[ cox1 cox2 cox3 rrnS rrnL nad1 nad6 cob nad4L nad4 nad5 nad3 nad2 ]

score = 278:

model 40:

-------------

R(0,1) R(0,9) R(0,10) R(0,17) R(0,18) R(0,24) R(0,25) R(0,28) R(0,n1) R(1,0) R(2,17) R(3,19) R(3,20) R(4,21) R(5,23) R(6,n4) R(7,n4) R(8,24) R(9,0) R(10,0) R(11,27) R(12,31) R(13,14) R(13,15) R(14,13) R(15,13) R(15,n6) R(15,n7) R(16,n5) R(17,0) R(17,2) R(18,0) R(18,19) R(19,3) R(19,18) R(19,22) R(20,3) R(20,21) R(21,4) R(21,20) R(22,19) R(22,23) R(23,5) R(23,22) R(24,0) R(24,8) R(25,0) R(25,26) R(26,25) R(26,27) R(27,11) R(27,26) R(28,0) R(28,29) R(29,28) R(29,30) R(30,29) R(30,31) R(31,12) R(31,30) R(n1,0) R(n1,n2) R(n1,n3) R(n2,n1) R(n2,n4) R(n3,n1) R(n3,n6) R(n4,6) R(n4,7) R(n4,n2) R(n5,16) R(n5,n7) R(n6,15) R(n6,n3) R(n7,15) R(n7,n5)

-------------------------------------------------------------------------------------------------------------

-> form outgroup SOL4 - A

n4(mod41)

[ cox1 cox2 atp8 atp6 cox3 nad3 -nad5 -nad4 -nad4L nad6 cob rrnS rrnL nad1 nad2 ]

n1(mod41)15g

[ cox1 cox2 atp8 atp6 cox3 rrnS rrnL nad1 nad6 cob nad4L nad4 nad5 nad3 nad2 ]

n1(mod41)13g

[ cox1 cox2 cox3 rrnS rrnL nad1 nad6 cob nad4L nad4 nad5 nad3 nad2 ]

n6(mod41)

[ cox1 cox2 nad1 nad3 nad2 cox3 rrnS -nad5 -nad4 -nad4L -cob -nad6 rrnL ]

score = 268:

model 41:

-------------

R(0,1) R(0,9) R(0,10) R(0,17) R(0,18) R(0,24) R(0,25) R(0,28) R(0,n1) R(1,0) R(2,17) R(3,19) R(3,20) R(4,21) R(5,23) R(6,n4) R(7,n4) R(8,24) R(9,0) R(10,0) R(11,27) R(12,31) R(13,14) R(13,15) R(14,13) R(15,13) R(15,n6) R(16,n5) R(17,0) R(17,2) R(18,0) R(18,19) R(19,3) R(19,18) R(19,22) R(20,3) R(20,21) R(21,4) R(21,20) R(22,19) R(22,23) R(23,5) R(23,22) R(24,0) R(24,8) R(25,0) R(25,26) R(26,25) R(26,27) R(27,11) R(27,26) R(28,0) R(28,29) R(29,28) R(29,30) R(30,29) R(30,31) R(31,12) R(31,30) R(n1,0) R(n1,n2) R(n1,n3) R(n2,n1) R(n2,n4) R(n3,n1) R(n3,n6) R(n4,6) R(n4,7) R(n4,n2) R(n5,16) R(n5,n7) R(n6,15) R(n6,n3) R(n6,n7) R(n7,n5) R(n7,n6)

-------------------------------------------------------------------------------------------------------------

-> form outgroup SOL4 - A

n4(mod42)

[ cox1 cox2 atp8 atp6 cox3 nad3 -nad5 -nad4 -nad4L nad6 cob rrnS rrnL nad1 nad2 ]

n1(mod42)15g

[ cox1 cox2 atp8 atp6 cox3 rrnS rrnL nad1 nad6 cob nad4L nad4 nad5 nad3 nad2 ]

n1(mod42)13g

[ cox1 cox2 cox3 rrnS rrnL nad1 nad6 cob nad4L nad4 nad5 nad3 nad2 ]

score = 278:

model 42:

-------------

R(0,1) R(0,9) R(0,10) R(0,17) R(0,18) R(0,24) R(0,25) R(0,28) R(0,n1) R(1,0) R(2,17) R(3,19) R(3,20) R(4,21) R(5,23) R(6,n4) R(7,n4) R(8,24) R(9,0) R(10,0) R(11,27) R(12,31) R(13,14) R(13,15) R(13,n7) R(14,13) R(15,13) R(15,n6) R(16,n5) R(17,0) R(17,2) R(18,0) R(18,19) R(19,3) R(19,18) R(19,22) R(20,3) R(20,21) R(21,4) R(21,20) R(22,19) R(22,23) R(23,5) R(23,22) R(24,0) R(24,8) R(25,0) R(25,26) R(26,25) R(26,27) R(27,11) R(27,26) R(28,0) R(28,29) R(29,28) R(29,30) R(30,29) R(30,31) R(31,12) R(31,30) R(n1,0) R(n1,n2) R(n1,n3) R(n2,n1) R(n2,n4) R(n3,n1) R(n3,n7) R(n4,6) R(n4,7) R(n4,n2) R(n5,16) R(n5,n6) R(n6,15) R(n6,n5) R(n7,13) R(n7,n3)

-------------------------------------------------------------------------------------------------------------

-> form outgroup SOL4 - A

n4(mod43)

[ cox1 cox2 atp8 atp6 cox3 nad3 -nad5 -nad4 -nad4L nad6 cob rrnS rrnL nad1 nad2 ]

n1(mod43)15g

[ cox1 cox2 atp8 atp6 cox3 rrnS rrnL nad1 nad6 cob nad4L nad4 nad5 nad3 nad2 ]

n1(mod43)13g

[ cox1 cox2 cox3 rrnS rrnL nad1 nad6 cob nad4L nad4 nad5 nad3 nad2 ]

score = 290:

model 43:

-------------

R(0,1) R(0,9) R(0,10) R(0,17) R(0,18) R(0,24) R(0,25) R(0,28) R(0,n1) R(1,0) R(2,17) R(3,19) R(3,20) R(4,21) R(5,23) R(6,n4) R(7,n4) R(8,24) R(9,0) R(10,0) R(11,27) R(12,31) R(13,14) R(13,15) R(13,n7) R(14,13) R(15,13) R(15,n6) R(16,n5) R(17,0) R(17,2) R(18,0) R(18,19) R(19,3) R(19,18) R(19,22) R(20,3) R(20,21) R(21,4) R(21,20) R(22,19) R(22,23) R(23,5) R(23,22) R(24,0) R(24,8) R(25,0) R(25,26) R(26,25) R(26,27) R(27,11) R(27,26) R(28,0) R(28,29) R(29,28) R(29,30) R(30,29) R(30,31) R(31,12) R(31,30) R(n1,0) R(n1,n2) R(n1,n3) R(n2,n1) R(n2,n4) R(n3,n1) R(n3,n6) R(n4,6) R(n4,7) R(n4,n2) R(n5,16) R(n5,n7) R(n6,15) R(n6,n3) R(n7,13) R(n7,n5)

-------------------------------------------------------------------------------------------------------------

-> form outgroup SOL4 - A

n4(mod44)

[ cox1 cox2 atp8 atp6 cox3 nad3 -nad5 -nad4 -nad4L nad6 cob rrnS rrnL nad1 nad2 ]

n1(mod44)15g

[ cox1 cox2 atp8 atp6 cox3 rrnS rrnL nad1 nad6 cob nad4L nad4 nad5 nad3 nad2 ]

n1(mod44)13g

[ cox1 cox2 cox3 rrnS rrnL nad1 nad6 cob nad4L nad4 nad5 nad3 nad2 ]

score = 264:

model 44:

-------------

R(0,1) R(0,9) R(0,10) R(0,17) R(0,18) R(0,24) R(0,25) R(0,28) R(0,n1) R(1,0) R(2,17) R(3,19) R(3,20) R(4,21) R(5,23) R(6,n4) R(7,n4) R(8,24) R(9,0) R(10,0) R(11,27) R(12,31) R(13,14) R(13,15) R(13,n6) R(13,n7) R(14,13) R(15,13) R(16,n5) R(17,0) R(17,2) R(18,0) R(18,19) R(19,3) R(19,18) R(19,22) R(20,3) R(20,21) R(21,4) R(21,20) R(22,19) R(22,23) R(23,5) R(23,22) R(24,0) R(24,8) R(25,0) R(25,26) R(26,25) R(26,27) R(27,11) R(27,26) R(28,0) R(28,29) R(29,28) R(29,30) R(30,29) R(30,31) R(31,12) R(31,30) R(n1,0) R(n1,n2) R(n1,n3) R(n2,n1) R(n2,n4) R(n3,n1) R(n3,n6) R(n4,6) R(n4,7) R(n4,n2) R(n5,16) R(n5,n7) R(n6,13) R(n6,n3) R(n7,13) R(n7,n5)

-------------------------------------------------------------------------------------------------------------

-> form outgroup SOL4 - A

n4(mod45)

[ cox1 cox2 atp8 atp6 cox3 nad3 -nad5 -nad4 -nad4L nad6 cob rrnS rrnL nad1 nad2 ]

n1(mod45)15g

[ cox1 cox2 atp8 atp6 cox3 rrnS rrnL nad1 nad6 cob nad4L nad4 nad5 nad3 nad2 ]

n1(mod45)13g

[ cox1 cox2 cox3 rrnS rrnL nad1 nad6 cob nad4L nad4 nad5 nad3 nad2 ]

n6(mod45)

g1: [ cox1 cox2 nad1 nad3 nad2 cox3 rrnS -nad5 -nad4 -nad4L -cob -nad6 rrnL ]

g2: [ cox1 cox2 nad1 nad6 cob nad4L nad5 nad4 nad3 nad2 cox3 rrnS rrnL ]

score = 254:

model 45:

-------------

R(0,1) R(0,9) R(0,10) R(0,17) R(0,18) R(0,24) R(0,25) R(0,28) R(0,n1) R(1,0) R(2,17) R(3,19) R(3,20) R(4,21) R(5,23) R(6,n4) R(7,n4) R(8,24) R(9,0) R(10,0) R(11,27) R(12,31) R(13,14) R(13,15) R(13,n6) R(14,13) R(15,13) R(16,n5) R(17,0) R(17,2) R(18,0) R(18,19) R(19,3) R(19,18) R(19,22) R(20,3) R(20,21) R(21,4) R(21,20) R(22,19) R(22,23) R(23,5) R(23,22) R(24,0) R(24,8) R(25,0) R(25,26) R(26,25) R(26,27) R(27,11) R(27,26) R(28,0) R(28,29) R(29,28) R(29,30) R(30,29) R(30,31) R(31,12) R(31,30) R(n1,0) R(n1,n2) R(n1,n3) R(n2,n1) R(n2,n4) R(n3,n1) R(n3,n6) R(n4,6) R(n4,7) R(n4,n2) R(n5,16) R(n5,n7) R(n6,13) R(n6,n3) R(n6,n7) R(n7,n5) R(n7,n6)

-------------------------------------------------------------------------------------------------------------

-> form outgroup SOL4 - A

n4(mod46)

[ cox1 cox2 atp8 atp6 cox3 nad3 -nad5 -nad4 -nad4L nad6 cob rrnS rrnL nad1 nad2 ]

n6(mod46)

g1: [ cox1 cox2 nad1 nad3 nad2 cox3 rrnS -nad4 -nad5 -cob -nad6 -nad4L rrnL ]

g2: [ cox1 cox2 nad1 nad3 nad2 cox3 rrnS nad5 nad4 -nad4L -cob -nad6 rrnL ]

g3: [ cox1 cox2 nad1 nad3 nad2 cox3 rrnS rrnL nad6 cob nad4L nad5 nad4 ]

g4: [ cox1 cox2 nad1 nad3 nad2 cox3 rrnS nad6 cob nad4L -nad4 -nad5 rrnL ]

g5: [ cox1 cox2 nad1 nad3 nad2 cox3 rrnS -rrnL nad6 cob nad4L -nad4 -nad5 ]

n1(mod46)15g

for n6-g1:

g1[ cox1 cox2 atp8 atp6 cox3 rrnS rrnL nad1 nad6 cob nad4L nad4 nad5 nad3 nad2 ]

for n6-g2:

g2[ cox1 cox2 atp8 atp6 cox3 rrnS rrnL nad1 nad6 cob nad4L nad4 nad5 nad3 nad2 ]

for n6-g3:

g3[ cox1 cox2 atp8 atp6 cox3 rrnS rrnL nad1 nad6 cob nad4L nad4 nad5 nad3 nad2 ]

g4[ cox1 cox2 atp8 atp6 cox3 nad3 nad2 rrnS rrnL nad1 nad6 cob nad4L nad4 nad5 ]

g5[ cox1 cox2 atp8 atp6 -nad5 -nad4 -nad4L -cob -nad6 -rrnL -rrnS nad1 cox3 nad3 nad2 ]

for n6-g4:

g6[ cox1 cox2 atp8 atp6 cox3 rrnS rrnL nad1 nad6 cob nad4L nad4 nad5 nad3 nad2 ]

for n6-g5:

g7[ cox1 cox2 atp8 atp6 nad6 cob nad4L nad4 nad5 -cox3 rrnS rrnL nad1 nad3 nad2 ]

n1(mod46)13g

for n6-g1:

g1[ cox1 cox2 cox3 rrnS rrnL nad1 nad6 cob nad4L nad4 nad5 nad3 nad2 ]

for n6-g2:

g2[ cox1 cox2 cox3 rrnS rrnL nad1 nad6 cob nad4L nad4 nad5 nad3 nad2 ]

for n6-g3:

g3[ cox1 cox2 cox3 rrnS rrnL nad1 nad6 cob nad4L nad4 nad5 nad3 nad2 ]

g4[ cox1 cox2 cox3 nad3 nad2 rrnS rrnL nad1 nad6 cob nad4L nad4 nad5 ]

g5[ cox1 cox2 -nad5 -nad4 -nad4L -cob -nad6 -rrnL -rrnS nad1 cox3 nad3 nad2 ]

for n6-g4:

g6[ cox1 cox2 cox3 rrnS rrnL nad1 nad6 cob nad4L nad4 nad5 nad3 nad2 ]

for n6-g5:

g7[ cox1 cox2 nad6 cob nad4L nad4 nad5 -cox3 rrnS rrnL nad1 nad3 nad2 ]

score = 290:

model 46:

-------------

R(0,1) R(0,9) R(0,10) R(0,17) R(0,18) R(0,24) R(0,25) R(0,28) R(0,n1) R(1,0) R(2,17) R(3,19) R(3,20) R(4,21) R(5,23) R(6,n4) R(7,n4) R(8,24) R(9,0) R(10,0) R(11,27) R(12,31) R(13,14) R(13,15) R(13,n6) R(14,13) R(15,13) R(16,n5) R(17,0) R(17,2) R(18,0) R(18,19) R(19,3) R(19,18) R(19,22) R(20,3) R(20,21) R(21,4) R(21,20) R(22,19) R(22,23) R(23,5) R(23,22) R(24,0) R(24,8) R(25,0) R(25,26) R(26,25) R(26,27) R(27,11) R(27,26) R(28,0) R(28,29) R(29,28) R(29,30) R(30,29) R(30,31) R(31,12) R(31,30) R(n1,0) R(n1,n2) R(n1,n3) R(n2,n1) R(n2,n4) R(n3,n1) R(n3,n7) R(n4,6) R(n4,7) R(n4,n2) R(n5,16) R(n5,n6) R(n6,13) R(n6,n5) R(n6,n7) R(n7,n3) R(n7,n6)

-------------------------------------------------------------------------------------------------------------

-> form outgroup SOL5 - A

n1(mod47)15g

[ cox1 cox2 atp8 atp6 cox3 rrnS rrnL nad1 nad6 cob nad4L nad4 nad5 nad3 nad2 ]

n1(mod47)13g

[ cox1 cox2 cox3 rrnS rrnL nad1 nad6 cob nad4L nad4 nad5 nad3 nad2 ]

n6(mod47)

g1: [ cox1 cox2 nad1 nad3 nad2 cox3 rrnS -nad5 -nad4 -nad4L -cob -nad6 rrnL ]

g2: [ cox1 cox2 nad1 nad6 cob nad4L nad5 nad4 nad3 nad2 cox3 rrnS rrnL ]

score = 254:

model 47:

-------------

R(0,1) R(0,9) R(0,10) R(0,17) R(0,18) R(0,24) R(0,25) R(0,28) R(0,n1) R(1,0) R(2,17) R(3,19) R(3,20) R(4,21) R(5,23) R(6,n2) R(6,n4) R(7,n4) R(8,24) R(9,0) R(10,0) R(11,27) R(12,31) R(13,14) R(13,15) R(13,n6) R(14,13) R(15,13) R(16,n5) R(17,0) R(17,2) R(18,0) R(18,19) R(19,3) R(19,18) R(19,22) R(20,3) R(20,21) R(21,4) R(21,20) R(22,19) R(22,23) R(23,5) R(23,22) R(24,0) R(24,8) R(25,0) R(25,26) R(26,25) R(26,27) R(27,11) R(27,26) R(28,0) R(28,29) R(29,28) R(29,30) R(30,29) R(30,31) R(31,12) R(31,30) R(n1,0) R(n1,n2) R(n1,n3) R(n2,6) R(n2,n1) R(n3,n1) R(n3,n6) R(n4,6) R(n4,7) R(n5,16) R(n5,n7) R(n6,13) R(n6,n3) R(n6,n7) R(n7,n5) R(n7,n6)

-------------------------------------------------------------------------------------------------------------

-> form outgroup SOL5 - A

n1(mod48)15g

[ cox1 cox2 atp8 atp6 cox3 rrnS rrnL nad1 nad6 cob nad4L nad4 nad5 nad3 nad2 ]

n1(mod48)13g

[ cox1 cox2 cox3 rrnS rrnL nad1 nad6 cob nad4L nad4 nad5 nad3 nad2 ]

n6(mod48)

[ cox1 cox2 nad1 nad3 nad2 cox3 rrnS -nad5 -nad4 -nad4L -cob -nad6 rrnL ]

score = 268:

model 48:

-------------

R(0,1) R(0,9) R(0,10) R(0,17) R(0,18) R(0,24) R(0,25) R(0,28) R(0,n1) R(1,0) R(2,17) R(3,19) R(3,20) R(4,21) R(5,23) R(6,n2) R(6,n4) R(7,n4) R(8,24) R(9,0) R(10,0) R(11,27) R(12,31) R(13,14) R(13,15) R(14,13) R(15,13) R(15,n6) R(16,n5) R(17,0) R(17,2) R(18,0) R(18,19) R(19,3) R(19,18) R(19,22) R(20,3) R(20,21) R(21,4) R(21,20) R(22,19) R(22,23) R(23,5) R(23,22) R(24,0) R(24,8) R(25,0) R(25,26) R(26,25) R(26,27) R(27,11) R(27,26) R(28,0) R(28,29) R(29,28) R(29,30) R(30,29) R(30,31) R(31,12) R(31,30) R(n1,0) R(n1,n2) R(n1,n3) R(n2,6) R(n2,n1) R(n3,n1) R(n3,n6) R(n4,6) R(n4,7) R(n5,16) R(n5,n7) R(n6,15) R(n6,n3) R(n6,n7) R(n7,n5) R(n7,n6)

-------------------------------------------------------------------------------------------------------------

-> form outgroup SOL5 - A

n7(mod49)

g1: [ cox1 cox2 nad1 nad3 nad2 cox3 rrnS -nad4 -nad5 -cob -nad6 -nad4L rrnL ]

g2: [ cox1 cox2 nad1 nad3 nad2 cox3 rrnS nad5 nad4 -nad4L -cob -nad6 rrnL ]

g3: [ cox1 cox2 nad1 nad3 nad2 cox3 rrnS rrnL nad6 cob nad4L nad5 nad4 ]

g4: [ cox1 cox2 nad1 nad3 nad2 cox3 rrnS nad6 cob nad4L -nad4 -nad5 rrnL ]

g5: [ cox1 cox2 nad1 nad3 nad2 cox3 rrnS -nad4 -nad5 -rrnL -nad4L -cob -nad6 ]

n1(mod49)15g

for n7-g1:

g1: [ cox1 cox2 atp8 atp6 cox3 rrnS rrnL nad1 nad6 cob nad4L nad4 nad5 nad3 nad2 ]

for n7-g2:

g2: [ cox1 cox2 atp8 atp6 cox3 rrnS rrnL nad1 nad6 cob nad4L nad4 nad5 nad3 nad2 ]

for n7-g3:

g3: [ cox1 cox2 atp8 atp6 cox3 rrnS rrnL nad1 nad6 cob nad4L nad4 nad5 nad3 nad2 ]

g4: [ cox1 cox2 atp8 atp6 cox3 nad3 nad2 rrnS rrnL nad1 nad6 cob nad4L nad4 nad5 ]

g5: [ cox1 cox2 atp8 atp6 -nad5 -nad4 -nad4L -cob -nad6 -cox3 rrnS rrnL nad1 nad3 nad2 ]

for n7-g4:

g6: [ cox1 cox2 atp8 atp6 cox3 rrnS rrnL nad1 nad6 cob nad4L nad4 nad5 nad3 nad2 ]

for n7-g5:

g7: [ cox1 cox2 atp8 atp6 -nad5 -nad4 -nad4L -cob -nad6 -cox3 rrnS rrnL nad1 nad3 nad2 ]

n1(mod49)(13g)

for n7-g1:

g1: [ cox1 cox2 cox3 rrnS rrnL nad1 nad6 cob nad4L nad4 nad5 nad3 nad2 ]

for n7-g2:

g2: [ cox1 cox2 cox3 rrnS rrnL nad1 nad6 cob nad4L nad4 nad5 nad3 nad2 ]

for n7-g3:

g3: [ cox1 cox2 cox3 rrnS rrnL nad1 nad6 cob nad4L nad4 nad5 nad3 nad2 ]

g4: [ cox1 cox2 cox3 nad3 nad2 rrnS rrnL nad1 nad6 cob nad4L nad4 nad5 ]

g5: [ cox1 cox2 -nad5 -nad4 -nad4L -cob -nad6 -cox3 rrnS rrnL nad1 nad3 nad2 ]

for n7-g4:

g6: [ cox1 cox2 cox3 rrnS rrnL nad1 nad6 cob nad4L nad4 nad5 nad3 nad2 ]

for n7-g5:

g7: [ cox1 cox2 -nad5 -nad4 -nad4L -cob -nad6 -cox3 rrnS rrnL nad1 nad3 nad2 ]

score = 290:

model 49:

-------------

R(0,1) R(0,9) R(0,10) R(0,17) R(0,18) R(0,24) R(0,25) R(0,28) R(0,n1) R(1,0) R(2,17) R(3,19) R(3,20) R(4,21) R(5,23) R(6,n2) R(6,n4) R(7,n4) R(8,24) R(9,0) R(10,0) R(11,27) R(12,31) R(13,14) R(13,15) R(13,n7) R(14,13) R(15,13) R(16,n5) R(17,0) R(17,2) R(18,0) R(18,19) R(19,3) R(19,18) R(19,22) R(20,3) R(20,21) R(21,4) R(21,20) R(22,19) R(22,23) R(23,5) R(23,22) R(24,0) R(24,8) R(25,0) R(25,26) R(26,25) R(26,27) R(27,11) R(27,26) R(28,0) R(28,29) R(29,28) R(29,30) R(30,29) R(30,31) R(31,12) R(31,30) R(n1,0) R(n1,n2) R(n1,n3) R(n2,6) R(n2,n1) R(n3,n1) R(n3,n6) R(n4,6) R(n4,7) R(n5,16) R(n5,n7) R(n6,n3) R(n6,n7) R(n7,13) R(n7,n5) R(n7,n6)

-------------------------------------------------------------------------------------------------------------

-> form outgroup SOL5 - A

n1(mod50)15g

[ cox1 cox2 atp8 atp6 cox3 rrnS rrnL nad1 nad6 cob nad4L nad4 nad5 nad3 nad2 ]

n1(mod50)13g

[ cox1 cox2 cox3 rrnS rrnL nad1 nad6 cob nad4L nad4 nad5 nad3 nad2 ]

score = 278:

model 50:

-------------

R(0,1) R(0,9) R(0,10) R(0,17) R(0,18) R(0,24) R(0,25) R(0,28) R(0,n1) R(1,0) R(2,17) R(3,19) R(3,20) R(4,21) R(5,23) R(6,n2) R(6,n4) R(7,n4) R(8,24) R(9,0) R(10,0) R(11,27) R(12,31) R(13,14) R(13,15) R(14,13) R(15,13) R(15,n6) R(15,n7) R(16,n5) R(17,0) R(17,2) R(18,0) R(18,19) R(19,3) R(19,18) R(19,22) R(20,3) R(20,21) R(21,4) R(21,20) R(22,19) R(22,23) R(23,5) R(23,22) R(24,0) R(24,8) R(25,0) R(25,26) R(26,25) R(26,27) R(27,11) R(27,26) R(28,0) R(28,29) R(29,28) R(29,30) R(30,29) R(30,31) R(31,12) R(31,30) R(n1,0) R(n1,n2) R(n1,n3) R(n2,6) R(n2,n1) R(n3,n1) R(n3,n6) R(n4,6) R(n4,7) R(n5,16) R(n5,n7) R(n6,15) R(n6,n3) R(n7,15) R(n7,n5)

-------------------------------------------------------------------------------------------------------------

-> form outgroup SOL5 - A

n1(mod51)15g

[ cox1 cox2 atp8 atp6 cox3 rrnS rrnL nad1 nad6 cob nad4L nad4 nad5 nad3 nad2 ]

n1(mod51)13g

[ cox1 cox2 cox3 rrnS rrnL nad1 nad6 cob nad4L nad4 nad5 nad3 nad2 ]

score = 290:

model 51:

-------------

R(0,1) R(0,9) R(0,10) R(0,17) R(0,18) R(0,24) R(0,25) R(0,28) R(0,n1) R(1,0) R(2,17) R(3,19) R(3,20) R(4,21) R(5,23) R(6,n2) R(6,n4) R(7,n4) R(8,24) R(9,0) R(10,0) R(11,27) R(12,31) R(13,14) R(13,15) R(13,n7) R(14,13) R(15,13) R(15,n6) R(16,n5) R(17,0) R(17,2) R(18,0) R(18,19) R(19,3) R(19,18) R(19,22) R(20,3) R(20,21) R(21,4) R(21,20) R(22,19) R(22,23) R(23,5) R(23,22) R(24,0) R(24,8) R(25,0) R(25,26) R(26,25) R(26,27) R(27,11) R(27,26) R(28,0) R(28,29) R(29,28) R(29,30) R(30,29) R(30,31) R(31,12) R(31,30) R(n1,0) R(n1,n2) R(n1,n3) R(n2,6) R(n2,n1) R(n3,n1) R(n3,n6) R(n4,6) R(n4,7) R(n5,16) R(n5,n7) R(n6,15) R(n6,n3) R(n7,13) R(n7,n5)

-------------------------------------------------------------------------------------------------------------

-> form outgroup SOL5 - A

n1(mod52)15g

[ cox1 cox2 atp8 atp6 cox3 rrnS rrnL nad1 nad6 cob nad4L nad4 nad5 nad3 nad2 ]

n1(mod52)13g

[ cox1 cox2 cox3 rrnS rrnL nad1 nad6 cob nad4L nad4 nad5 nad3 nad2 ]

score = 264:

model 52:

-------------

R(0,1) R(0,9) R(0,10) R(0,17) R(0,18) R(0,24) R(0,25) R(0,28) R(0,n1) R(1,0) R(2,17) R(3,19) R(3,20) R(4,21) R(5,23) R(6,n2) R(6,n4) R(7,n4) R(8,24) R(9,0) R(10,0) R(11,27) R(12,31) R(13,14) R(13,15) R(13,n6) R(13,n7) R(14,13) R(15,13) R(16,n5) R(17,0) R(17,2) R(18,0) R(18,19) R(19,3) R(19,18) R(19,22) R(20,3) R(20,21) R(21,4) R(21,20) R(22,19) R(22,23) R(23,5) R(23,22) R(24,0) R(24,8) R(25,0) R(25,26) R(26,25) R(26,27) R(27,11) R(27,26) R(28,0) R(28,29) R(29,28) R(29,30) R(30,29) R(30,31) R(31,12) R(31,30) R(n1,0) R(n1,n2) R(n1,n3) R(n2,6) R(n2,n1) R(n3,n1) R(n3,n6) R(n4,6) R(n4,7) R(n5,16) R(n5,n7) R(n6,13) R(n6,n3) R(n7,13) R(n7,n5)

-------------------------------------------------------------------------------------------------------------

-> form outgroup SOL5 - A

n1(mod53)15g

[ cox1 cox2 atp8 atp6 cox3 rrnS rrnL nad1 nad6 cob nad4L nad4 nad5 nad3 nad2 ]

n1(mod53)13g

[ cox1 cox2 cox3 rrnS rrnL nad1 nad6 cob nad4L nad4 nad5 nad3 nad2 ]

score = 278:

model 53:

-------------

R(0,1) R(0,9) R(0,10) R(0,17) R(0,18) R(0,24) R(0,25) R(0,28) R(0,n1) R(1,0) R(2,17) R(3,19) R(3,20) R(4,21) R(5,23) R(6,n2) R(6,n4) R(7,n4) R(8,24) R(9,0) R(10,0) R(11,27) R(12,31) R(13,14) R(13,15) R(13,n6) R(14,13) R(15,13) R(15,n7) R(16,n5) R(17,0) R(17,2) R(18,0) R(18,19) R(19,3) R(19,18) R(19,22) R(20,3) R(20,21) R(21,4) R(21,20) R(22,19) R(22,23) R(23,5) R(23,22) R(24,0) R(24,8) R(25,0) R(25,26) R(26,25) R(26,27) R(27,11) R(27,26) R(28,0) R(28,29) R(29,28) R(29,30) R(30,29) R(30,31) R(31,12) R(31,30) R(n1,0) R(n1,n2) R(n1,n3) R(n2,6) R(n2,n1) R(n3,n1) R(n3,n6) R(n4,6) R(n4,7) R(n5,16) R(n5,n7) R(n6,13) R(n6,n3) R(n7,15) R(n7,n5)

-------------------------------------------------------------------------------------------------------------

-> form outgroup SOL4 - A

n4(mod54)

[ cox1 cox2 atp8 atp6 cox3 nad3 -nad5 -nad4 -nad4L nad6 cob rrnS rrnL nad1 nad2 ]

n1(mod54)15g

[ cox1 cox2 atp8 atp6 cox3 rrnS rrnL nad1 nad6 cob nad4L nad4 nad5 nad3 nad2 ]

n1(mod54)13g

[ cox1 cox2 cox3 rrnS rrnL nad1 nad6 cob nad4L nad4 nad5 nad3 nad2 ]

n7(mod54)

[ cox1 cox2 nad1 nad3 nad2 cox3 rrnS -nad4L -nad4 -nad5 -cob -nad6 rrnL ]

score = 304:

model 54:

-------------

R(0,1) R(0,9) R(0,10) R(0,17) R(0,18) R(0,24) R(0,25) R(0,28) R(0,n1) R(1,0) R(2,17) R(3,19) R(3,20) R(4,21) R(5,23) R(6,n4) R(7,n4) R(8,24) R(9,0) R(10,0) R(11,27) R(12,31) R(13,14) R(13,n7) R(14,13) R(15,n6) R(15,n7) R(16,n5) R(17,0) R(17,2) R(18,0) R(18,19) R(19,3) R(19,18) R(19,22) R(20,3) R(20,21) R(21,4) R(21,20) R(22,19) R(22,23) R(23,5) R(23,22) R(24,0) R(24,8) R(25,0) R(25,26) R(26,25) R(26,27) R(27,11) R(27,26) R(28,0) R(28,29) R(29,28) R(29,30) R(30,29) R(30,31) R(31,12) R(31,30) R(n1,0) R(n1,n2) R(n1,n3) R(n2,n1) R(n2,n4) R(n3,n1) R(n3,n6) R(n4,6) R(n4,7) R(n4,n2) R(n5,16) R(n5,n7) R(n6,15) R(n6,n3) R(n7,13) R(n7,15) R(n7,n5)

-------------------------------------------------------------------------------------------------------------

-> form outgroup SOL4 - A

n4(mod55)

[ cox1 cox2 atp8 atp6 cox3 nad3 -nad5 -nad4 -nad4L nad6 cob rrnS rrnL nad1 nad2 ]

n1(mod55)15g

[ cox1 cox2 atp8 atp6 cox3 rrnS rrnL nad1 nad6 cob nad4L nad4 nad5 nad3 nad2 ]

n1(mod55)13g

[ cox1 cox2 cox3 rrnS rrnL nad1 nad6 cob nad4L nad4 nad5 nad3 nad2 ]

n6(mod55)

[ cox1 cox2 nad1 nad3 nad2 cox3 rrnS -nad5 -nad4 -nad4L -cob -nad6 rrnL ]

score = 256:

model 55:

-------------

R(0,1) R(0,9) R(0,10) R(0,17) R(0,18) R(0,24) R(0,25) R(0,28) R(0,n1) R(1,0) R(2,17) R(3,19) R(3,20) R(4,21) R(5,23) R(6,n4) R(7,n4) R(8,24) R(9,0) R(10,0) R(11,27) R(12,31) R(13,14) R(13,n6) R(14,13) R(15,n6) R(15,n7) R(16,n5) R(17,0) R(17,2) R(18,0) R(18,19) R(19,3) R(19,18) R(19,22) R(20,3) R(20,21) R(21,4) R(21,20) R(22,19) R(22,23) R(23,5) R(23,22) R(24,0) R(24,8) R(25,0) R(25,26) R(26,25) R(26,27) R(27,11) R(27,26) R(28,0) R(28,29) R(29,28) R(29,30) R(30,29) R(30,31) R(31,12) R(31,30) R(n1,0) R(n1,n2) R(n1,n3) R(n2,n1) R(n2,n4) R(n3,n1) R(n3,n6) R(n4,6) R(n4,7) R(n4,n2) R(n5,16) R(n5,n7) R(n6,13) R(n6,15) R(n6,n3) R(n7,15) R(n7,n5)

-------------------------------------------------------------------------------------------------------------

-> form outgroup SOL4 - A

n4(mod56)

[ cox1 cox2 atp8 atp6 cox3 nad3 -nad5 -nad4 -nad4L nad6 cob rrnS rrnL nad1 nad2 ]

n1(mod56)15g

[ cox1 cox2 atp8 atp6 cox3 rrnS rrnL nad1 nad6 cob nad4L nad4 nad5 nad3 nad2 ]

n1(mod56)13g

[ cox1 cox2 cox3 rrnS rrnL nad1 nad6 cob nad4L nad4 nad5 nad3 nad2 ]

n6(mod56)

[ cox1 cox2 nad1 nad3 nad2 cox3 rrnS -nad5 -nad4 -nad4L -cob -nad6 rrnL ]

score = 242:

model 56:

-------------

R(0,1) R(0,9) R(0,10) R(0,17) R(0,18) R(0,24) R(0,25) R(0,28) R(0,n1) R(1,0) R(2,17) R(3,19) R(3,20) R(4,21) R(5,23) R(6,n4) R(7,n4) R(8,24) R(9,0) R(10,0) R(11,27) R(12,31) R(13,14) R(13,n6) R(14,13) R(15,n6) R(16,n5) R(17,0) R(17,2) R(18,0) R(18,19) R(19,3) R(19,18) R(19,22) R(20,3) R(20,21) R(21,4) R(21,20) R(22,19) R(22,23) R(23,5) R(23,22) R(24,0) R(24,8) R(25,0) R(25,26) R(26,25) R(26,27) R(27,11) R(27,26) R(28,0) R(28,29) R(29,28) R(29,30) R(30,29) R(30,31) R(31,12) R(31,30) R(n1,0) R(n1,n2) R(n1,n3) R(n2,n1) R(n2,n4) R(n3,n1) R(n3,n6) R(n4,6) R(n4,7) R(n4,n2) R(n5,16) R(n5,n7) R(n6,13) R(n6,15) R(n6,n3) R(n6,n7) R(n7,n5) R(n7,n6)

-------------------------------------------------------------------------------------------------------------

-> form outgroup SOL4 - A

n4(mod57)

[ cox1 cox2 atp8 atp6 cox3 nad3 -nad5 -nad4 -nad4L nad6 cob rrnS rrnL nad1 nad2 ]

n1(mod57)15g

[ cox1 cox2 atp8 atp6 cox3 rrnS rrnL nad1 nad6 cob nad4L nad4 nad5 nad3 nad2 ]

n1(mod57)13g

[ cox1 cox2 cox3 rrnS rrnL nad1 nad6 cob nad4L nad4 nad5 nad3 nad2 ]

n6(mod57)

[ cox1 cox2 nad1 nad3 nad2 cox3 rrnS -nad5 -nad4 -nad4L -cob -nad6 rrnL ]

n7(mod57)

[ cox1 cox2 nad1 nad3 nad2 cox3 rrnS nad5 nad4 -nad4L -cob -nad6 rrnL ]

score = 268:

model 57:

-------------

R(0,1) R(0,9) R(0,10) R(0,17) R(0,18) R(0,24) R(0,25) R(0,28) R(0,n1) R(1,0) R(2,17) R(3,19) R(3,20) R(4,21) R(5,23) R(6,n4) R(7,n4) R(8,24) R(9,0) R(10,0) R(11,27) R(12,31) R(13,14) R(13,n7) R(14,13) R(15,n6) R(16,n5) R(17,0) R(17,2) R(18,0) R(18,19) R(19,3) R(19,18) R(19,22) R(20,3) R(20,21) R(21,4) R(21,20) R(22,19) R(22,23) R(23,5) R(23,22) R(24,0) R(24,8) R(25,0) R(25,26) R(26,25) R(26,27) R(27,11) R(27,26) R(28,0) R(28,29) R(29,28) R(29,30) R(30,29) R(30,31) R(31,12) R(31,30) R(n1,0) R(n1,n2) R(n1,n3) R(n2,n1) R(n2,n4) R(n3,n1) R(n3,n6) R(n4,6) R(n4,7) R(n4,n2) R(n5,16) R(n5,n7) R(n6,15) R(n6,n3) R(n6,n7) R(n7,13) R(n7,n5) R(n7,n6)

-------------------------------------------------------------------------------------------------------------

-> form outgroup SOL4 - A

n4(mod58)

[ cox1 cox2 atp8 atp6 cox3 nad3 -nad5 -nad4 -nad4L nad6 cob rrnS rrnL nad1 nad2 ]

n1(mod58)15g

[ cox1 cox2 atp8 atp6 cox3 rrnS rrnL nad1 nad6 cob nad4L nad4 nad5 nad3 nad2 ]

n1(mod58)13g

[ cox1 cox2 cox3 rrnS rrnL nad1 nad6 cob nad4L nad4 nad5 nad3 nad2 ]

n6(mod58)

[ cox1 cox2 nad1 nad3 nad2 cox3 rrnS -nad5 -nad4 -nad4L -cob -nad6 rrnL ]

score = 254:

model 58:

-------------

R(0,1) R(0,9) R(0,10) R(0,17) R(0,18) R(0,24) R(0,25) R(0,28) R(0,n1) R(1,0) R(2,17) R(3,19) R(3,20) R(4,21) R(5,23) R(6,n4) R(7,n4) R(8,24) R(9,0) R(10,0) R(11,27) R(12,31) R(13,14) R(13,n6) R(13,n7) R(14,13) R(15,n6) R(16,n5) R(17,0) R(17,2) R(18,0) R(18,19) R(19,3) R(19,18) R(19,22) R(20,3) R(20,21) R(21,4) R(21,20) R(22,19) R(22,23) R(23,5) R(23,22) R(24,0) R(24,8) R(25,0) R(25,26) R(26,25) R(26,27) R(27,11) R(27,26) R(28,0) R(28,29) R(29,28) R(29,30) R(30,29) R(30,31) R(31,12) R(31,30) R(n1,0) R(n1,n2) R(n1,n3) R(n2,n1) R(n2,n4) R(n3,n1) R(n3,n6) R(n4,6) R(n4,7) R(n4,n2) R(n5,16) R(n5,n7) R(n6,13) R(n6,15) R(n6,n3) R(n7,13) R(n7,n5)

-------------------------------------------------------------------------------------------------------------

-> form outgroup SOL4 - A

n4(mod59)

[ cox1 cox2 atp8 atp6 cox3 nad3 -nad5 -nad4 -nad4L nad6 cob rrnS rrnL nad1 nad2 ]

n1(mod59)15g

[ cox1 cox2 atp8 atp6 cox3 rrnS rrnL nad1 nad6 cob nad4L nad4 nad5 nad3 nad2 ]

n1(mod59)13g

[ cox1 cox2 cox3 rrnS rrnL nad1 nad6 cob nad4L nad4 nad5 nad3 nad2 ]

n6(mod59)

[ cox1 cox2 nad1 nad3 nad2 cox3 rrnS -nad4L -nad4 -nad5 -cob -nad6 rrnL ]

score = 278:

model 59:

-------------

R(0,1) R(0,9) R(0,10) R(0,17) R(0,18) R(0,24) R(0,25) R(0,28) R(0,n1) R(1,0) R(2,17) R(3,19) R(3,20) R(4,21) R(5,23) R(6,n4) R(7,n4) R(8,24) R(9,0) R(10,0) R(11,27) R(12,31) R(13,14) R(13,n6) R(13,n7) R(14,13) R(15,n6) R(16,n5) R(17,0) R(17,2) R(18,0) R(18,19) R(19,3) R(19,18) R(19,22) R(20,3) R(20,21) R(21,4) R(21,20) R(22,19) R(22,23) R(23,5) R(23,22) R(24,0) R(24,8) R(25,0) R(25,26) R(26,25) R(26,27) R(27,11) R(27,26) R(28,0) R(28,29) R(29,28) R(29,30) R(30,29) R(30,31) R(31,12) R(31,30) R(n1,0) R(n1,n2) R(n1,n3) R(n2,n1) R(n2,n4) R(n3,n1) R(n3,n7) R(n4,6) R(n4,7) R(n4,n2) R(n5,16) R(n5,n6) R(n6,13) R(n6,15) R(n6,n5) R(n7,13) R(n7,n3)

-------------------------------------------------------------------------------------------------------------

-> form outgroup SOL5 - A

n1(mod60)15g

[ cox1 cox2 atp8 atp6 cox3 rrnS rrnL nad1 nad6 cob nad4L nad4 nad5 nad3 nad2 ]

n1(mod60)13g

[ cox1 cox2 cox3 rrnS rrnL nad1 nad6 cob nad4L nad4 nad5 nad3 nad2 ]

n6(mod60)

[ cox1 cox2 nad1 nad3 nad2 cox3 rrnS -nad5 -nad4 -nad4L -cob -nad6 rrnL ]

score = 242:

model 60:

-------------

R(0,1) R(0,9) R(0,10) R(0,17) R(0,18) R(0,24) R(0,25) R(0,28) R(0,n1) R(1,0) R(2,17) R(3,19) R(3,20) R(4,21) R(5,23) R(6,n2) R(6,n4) R(7,n4) R(8,24) R(9,0) R(10,0) R(11,27) R(12,31) R(13,14) R(13,n6) R(14,13) R(15,n6) R(16,n5) R(17,0) R(17,2) R(18,0) R(18,19) R(19,3) R(19,18) R(19,22) R(20,3) R(20,21) R(21,4) R(21,20) R(22,19) R(22,23) R(23,5) R(23,22) R(24,0) R(24,8) R(25,0) R(25,26) R(26,25) R(26,27) R(27,11) R(27,26) R(28,0) R(28,29) R(29,28) R(29,30) R(30,29) R(30,31) R(31,12) R(31,30) R(n1,0) R(n1,n2) R(n1,n3) R(n2,6) R(n2,n1) R(n3,n1) R(n3,n6) R(n4,6) R(n4,7) R(n5,16) R(n5,n7) R(n6,13) R(n6,15) R(n6,n3) R(n6,n7) R(n7,n5) R(n7,n6)

-------------------------------------------------------------------------------------------------------------

-> form outgroup SOL5 - A

n1(mod61)15g

[ cox1 cox2 atp8 atp6 cox3 rrnS rrnL nad1 nad6 cob nad4L nad4 nad5 nad3 nad2 ]

n1(mod61)13g

[ cox1 cox2 cox3 rrnS rrnL nad1 nad6 cob nad4L nad4 nad5 nad3 nad2 ]

n6(mod61)

[ cox1 cox2 nad1 nad3 nad2 cox3 rrnS -nad5 -nad4 -nad4L -cob -nad6 rrnL ]

n7(mod61)

[ cox1 cox2 nad1 nad3 nad2 cox3 rrnS nad5 nad4 -nad4L -cob -nad6 rrnL ]

score = 268:

model 61:

-------------

R(0,1) R(0,9) R(0,10) R(0,17) R(0,18) R(0,24) R(0,25) R(0,28) R(0,n1) R(1,0) R(2,17) R(3,19) R(3,20) R(4,21) R(5,23) R(6,n2) R(6,n4) R(7,n4) R(8,24) R(9,0) R(10,0) R(11,27) R(12,31) R(13,14) R(13,n7) R(14,13) R(15,n6) R(16,n5) R(17,0) R(17,2) R(18,0) R(18,19) R(19,3) R(19,18) R(19,22) R(20,3) R(20,21) R(21,4) R(21,20) R(22,19) R(22,23) R(23,5) R(23,22) R(24,0) R(24,8) R(25,0) R(25,26) R(26,25) R(26,27) R(27,11) R(27,26) R(28,0) R(28,29) R(29,28) R(29,30) R(30,29) R(30,31) R(31,12) R(31,30) R(n1,0) R(n1,n2) R(n1,n3) R(n2,6) R(n2,n1) R(n3,n1) R(n3,n6) R(n4,6) R(n4,7) R(n5,16) R(n5,n7) R(n6,15) R(n6,n3) R(n6,n7) R(n7,13) R(n7,n5) R(n7,n6)

-------------------------------------------------------------------------------------------------------------

-> form outgroup SOL5 - A

n1(mod62)15g

[ cox1 cox2 atp8 atp6 cox3 rrnS rrnL nad1 nad6 cob nad4L nad4 nad5 nad3 nad2 ]

n1(mod62)13g

[ cox1 cox2 cox3 rrnS rrnL nad1 nad6 cob nad4L nad4 nad5 nad3 nad2 ]

n6(mod62)

[ cox1 cox2 nad1 nad3 nad2 cox3 rrnS -nad5 -nad4 -nad4L -cob -nad6 rrnL ]

score = 254:

model 62:

-------------

R(0,1) R(0,9) R(0,10) R(0,17) R(0,18) R(0,24) R(0,25) R(0,28) R(0,n1) R(1,0) R(2,17) R(3,19) R(3,20) R(4,21) R(5,23) R(6,n2) R(6,n4) R(7,n4) R(8,24) R(9,0) R(10,0) R(11,27) R(12,31) R(13,14) R(13,n6) R(13,n7) R(14,13) R(15,n6) R(16,n5) R(17,0) R(17,2) R(18,0) R(18,19) R(19,3) R(19,18) R(19,22) R(20,3) R(20,21) R(21,4) R(21,20) R(22,19) R(22,23) R(23,5) R(23,22) R(24,0) R(24,8) R(25,0) R(25,26) R(26,25) R(26,27) R(27,11) R(27,26) R(28,0) R(28,29) R(29,28) R(29,30) R(30,29) R(30,31) R(31,12) R(31,30) R(n1,0) R(n1,n2) R(n1,n3) R(n2,6) R(n2,n1) R(n3,n1) R(n3,n6) R(n4,6) R(n4,7) R(n5,16) R(n5,n7) R(n6,13) R(n6,15) R(n6,n3) R(n7,13) R(n7,n5)

-------------------------------------------------------------------------------------------------------------

-> form outgroup SOL5 - A

n1(mod63)15g

[ cox1 cox2 atp8 atp6 cox3 rrnS rrnL nad1 nad6 cob nad4L nad4 nad5 nad3 nad2 ]

n1(mod63)13g

[ cox1 cox2 cox3 rrnS rrnL nad1 nad6 cob nad4L nad4 nad5 nad3 nad2 ]

n6(mod63)

[ cox1 cox2 nad1 nad3 nad2 cox3 rrnS -nad5 -nad4 -nad4L -cob -nad6 rrnL ]

score = 256:

model 63:

-------------

R(0,1) R(0,9) R(0,10) R(0,17) R(0,18) R(0,24) R(0,25) R(0,28) R(0,n1) R(1,0) R(2,17) R(3,19) R(3,20) R(4,21) R(5,23) R(6,n2) R(6,n4) R(7,n4) R(8,24) R(9,0) R(10,0) R(11,27) R(12,31) R(13,14) R(13,n6) R(14,13) R(15,n6) R(15,n7) R(16,n5) R(17,0) R(17,2) R(18,0) R(18,19) R(19,3) R(19,18) R(19,22) R(20,3) R(20,21) R(21,4) R(21,20) R(22,19) R(22,23) R(23,5) R(23,22) R(24,0) R(24,8) R(25,0) R(25,26) R(26,25) R(26,27) R(27,11) R(27,26) R(28,0) R(28,29) R(29,28) R(29,30) R(30,29) R(30,31) R(31,12) R(31,30) R(n1,0) R(n1,n2) R(n1,n3) R(n2,6) R(n2,n1) R(n3,n1) R(n3,n6) R(n4,6) R(n4,7) R(n5,16) R(n5,n7) R(n6,13) R(n6,15) R(n6,n3) R(n7,15) R(n7,n5)

-------------------------------------------------------------------------------------------------------------

-> form outgroup SOL5 - A

n1(mod64)15g

[ cox1 cox2 atp8 atp6 cox3 rrnS rrnL nad1 nad6 cob nad4L nad4 nad5 nad3 nad2 ]

n1(mod64)13g

[ cox1 cox2 cox3 rrnS rrnL nad1 nad6 cob nad4L nad4 nad5 nad3 nad2 ]

n7(mod64)

[ cox1 cox2 nad1 nad3 nad2 cox3 rrnS -nad4L -nad4 -nad5 -cob -nad6 rrnL ]

score = 278:

model 64:

-------------

R(0,1) R(0,9) R(0,10) R(0,17) R(0,18) R(0,24) R(0,25) R(0,28) R(0,n1) R(1,0) R(2,17) R(3,19) R(3,20) R(4,21) R(5,23) R(6,n2) R(6,n4) R(7,n4) R(8,24) R(9,0) R(10,0) R(11,27) R(12,31) R(13,14) R(13,n6) R(13,n7) R(14,13) R(15,n7) R(16,n5) R(17,0) R(17,2) R(18,0) R(18,19) R(19,3) R(19,18) R(19,22) R(20,3) R(20,21) R(21,4) R(21,20) R(22,19) R(22,23) R(23,5) R(23,22) R(24,0) R(24,8) R(25,0) R(25,26) R(26,25) R(26,27) R(27,11) R(27,26) R(28,0) R(28,29) R(29,28) R(29,30) R(30,29) R(30,31) R(31,12) R(31,30) R(n1,0) R(n1,n2) R(n1,n3) R(n2,6) R(n2,n1) R(n3,n1) R(n3,n6) R(n4,6) R(n4,7) R(n5,16) R(n5,n7) R(n6,13) R(n6,n3) R(n7,13) R(n7,15) R(n7,n5)

-------------------------------------------------------------------------------------------------------------

-> form outgroup SOL5 - A

n1(mod65)15g

[ cox1 cox2 atp8 atp6 cox3 rrnS rrnL nad1 nad6 cob nad4L nad4 nad5 nad3 nad2 ]

n1(mod65)13g

[ cox1 cox2 cox3 rrnS rrnL nad1 nad6 cob nad4L nad4 nad5 nad3 nad2 ]

n7(mod65)

[ cox1 cox2 nad1 nad3 nad2 cox3 rrnS -nad4L -nad4 -nad5 -cob -nad6 rrnL ]

score = 304:

model 65:

-------------

R(0,1) R(0,9) R(0,10) R(0,17) R(0,18) R(0,24) R(0,25) R(0,28) R(0,n1) R(1,0) R(2,17) R(3,19) R(3,20) R(4,21) R(5,23) R(6,n2) R(6,n4) R(7,n4) R(8,24) R(9,0) R(10,0) R(11,27) R(12,31) R(13,14) R(13,n7) R(14,13) R(15,n6) R(15,n7) R(16,n5) R(17,0) R(17,2) R(18,0) R(18,19) R(19,3) R(19,18) R(19,22) R(20,3) R(20,21) R(21,4) R(21,20) R(22,19) R(22,23) R(23,5) R(23,22) R(24,0) R(24,8) R(25,0) R(25,26) R(26,25) R(26,27) R(27,11) R(27,26) R(28,0) R(28,29) R(29,28) R(29,30) R(30,29) R(30,31) R(31,12) R(31,30) R(n1,0) R(n1,n2) R(n1,n3) R(n2,6) R(n2,n1) R(n3,n1) R(n3,n6) R(n4,6) R(n4,7) R(n5,16) R(n5,n7) R(n6,15) R(n6,n3) R(n7,13) R(n7,15) R(n7,n5)

-------------------------------------------------------------------------------------------------------------

-> form outgroup SOL6 - A

n2(mod66)

[ cox1 cox2 atp8 atp6 cox3 nad3 -nad5 -nad4 -nad4L -cob -nad6 -nad1 -rrnL -rrnS nad2 ]

n1(mod66)15g

[ cox1 cox2 atp8 atp6 cox3 rrnS rrnL nad1 nad6 cob nad4L nad4 nad5 nad3 nad2 ]

n1(mod66)13g

[ cox1 cox2 cox3 rrnS rrnL nad1 nad6 cob nad4L nad4 nad5 nad3 nad2 ]

n6(mod66)

g1: [ cox1 cox2 nad1 nad3 nad2 cox3 rrnS -nad5 -nad4 -nad4L -cob -nad6 rrnL ]

g2: [ cox1 cox2 nad1 nad6 cob nad4L nad5 nad4 nad3 nad2 cox3 rrnS rrnL ]

score = 240:

model 66:

-------------

R(0,1) R(0,9) R(0,10) R(0,17) R(0,18) R(0,24) R(0,25) R(0,28) R(0,n1) R(1,0) R(2,17) R(3,19) R(3,20) R(4,21) R(5,23) R(6,n2) R(7,n4) R(8,24) R(9,0) R(10,0) R(11,27) R(12,31) R(13,14) R(13,15) R(13,n6) R(14,13) R(15,13) R(16,n5) R(17,0) R(17,2) R(18,0) R(18,19) R(19,3) R(19,18) R(19,22) R(20,3) R(20,21) R(21,4) R(21,20) R(22,19) R(22,23) R(23,5) R(23,22) R(24,0) R(24,8) R(25,0) R(25,26) R(26,25) R(26,27) R(27,11) R(27,26) R(28,0) R(28,29) R(29,28) R(29,30) R(30,29) R(30,31) R(31,12) R(31,30) R(n1,0) R(n1,n2) R(n1,n3) R(n2,6) R(n2,n1) R(n2,n4) R(n3,n1) R(n3,n6) R(n4,7) R(n4,n2) R(n5,16) R(n5,n7) R(n6,13) R(n6,n3) R(n6,n7) R(n7,n5) R(n7,n6)

-------------------------------------------------------------------------------------------------------------

-> form outgroup SOL6 - A

n2(mod67)

[ cox1 cox2 atp8 atp6 cox3 nad3 -nad5 -nad4 -nad4L -cob -nad6 -nad1 -rrnL -rrnS nad2 ]

n1(mod67)15g

[ cox1 cox2 atp8 atp6 cox3 rrnS rrnL nad1 nad6 cob nad4L nad4 nad5 nad3 nad2 ]

n1(mod67)13g

[ cox1 cox2 cox3 rrnS rrnL nad1 nad6 cob nad4L nad4 nad5 nad3 nad2 ]

n6(mod67)

[ cox1 cox2 nad1 nad3 nad2 cox3 rrnS -nad5 -nad4 -nad4L -cob -nad6 rrnL ]

score = 254:

model 67:

-------------

R(0,1) R(0,9) R(0,10) R(0,17) R(0,18) R(0,24) R(0,25) R(0,28) R(0,n1) R(1,0) R(2,17) R(3,19) R(3,20) R(4,21) R(5,23) R(6,n2) R(7,n4) R(8,24) R(9,0) R(10,0) R(11,27) R(12,31) R(13,14) R(13,15) R(14,13) R(15,13) R(15,n6) R(16,n5) R(17,0) R(17,2) R(18,0) R(18,19) R(19,3) R(19,18) R(19,22) R(20,3) R(20,21) R(21,4) R(21,20) R(22,19) R(22,23) R(23,5) R(23,22) R(24,0) R(24,8) R(25,0) R(25,26) R(26,25) R(26,27) R(27,11) R(27,26) R(28,0) R(28,29) R(29,28) R(29,30) R(30,29) R(30,31) R(31,12) R(31,30) R(n1,0) R(n1,n2) R(n1,n3) R(n2,6) R(n2,n1) R(n2,n4) R(n3,n1) R(n3,n6) R(n4,7) R(n4,n2) R(n5,16) R(n5,n7) R(n6,15) R(n6,n3) R(n6,n7) R(n7,n5) R(n7,n6)

-------------------------------------------------------------------------------------------------------------

-> form outgroup SOL6 - A

n2(mod68)

[ cox1 cox2 atp8 atp6 cox3 nad3 -nad5 -nad4 -nad4L -cob -nad6 -nad1 -rrnL -rrnS nad2 ]

n1(mod68)15g

g1: [ cox1 cox2 atp8 atp6 cox3 rrnS rrnL nad1 nad6 cob nad4L nad4 nad5 nad3 nad2 ]

and also for n7-g3:

g2: [ cox1 cox2 atp8 atp6 cox3 nad3 nad2 rrnS rrnL nad1 nad6 cob nad4L nad4 nad5 ]

n1(mod68)13g

g1: [ cox1 cox2 cox3 rrnS rrnL nad1 nad6 cob nad4L nad4 nad5 nad3 nad2 ]

and also for n7-g3:

g2: [ cox1 cox2 cox3 nad3 nad2 rrnS rrnL nad1 nad6 cob nad4L nad4 nad5 ]

n7(mod68)

g1: [ cox1 cox2 nad1 nad3 nad2 cox3 rrnS -nad4 -nad5 -cob -nad6 -nad4L rrnL ]

g2: [ cox1 cox2 nad1 nad3 nad2 cox3 rrnS nad5 nad4 -nad4L -cob -nad6 rrnL ]

g3: [ cox1 cox2 nad1 nad3 nad2 cox3 rrnS rrnL nad6 cob nad4L nad5 nad4 ]

g4: [ cox1 cox2 nad1 nad3 nad2 cox3 rrnS nad6 cob nad4L -nad4 -nad5 rrnL ]

score = 276:

model 68:

-------------

R(0,1) R(0,9) R(0,10) R(0,17) R(0,18) R(0,24) R(0,25) R(0,28) R(0,n1) R(1,0) R(2,17) R(3,19) R(3,20) R(4,21) R(5,23) R(6,n2) R(7,n4) R(8,24) R(9,0) R(10,0) R(11,27) R(12,31) R(13,14) R(13,15) R(13,n7) R(14,13) R(15,13) R(16,n5) R(17,0) R(17,2) R(18,0) R(18,19) R(19,3) R(19,18) R(19,22) R(20,3) R(20,21) R(21,4) R(21,20) R(22,19) R(22,23) R(23,5) R(23,22) R(24,0) R(24,8) R(25,0) R(25,26) R(26,25) R(26,27) R(27,11) R(27,26) R(28,0) R(28,29) R(29,28) R(29,30) R(30,29) R(30,31) R(31,12) R(31,30) R(n1,0) R(n1,n2) R(n1,n3) R(n2,6) R(n2,n1) R(n2,n4) R(n3,n1) R(n3,n6) R(n4,7) R(n4,n2) R(n5,16) R(n5,n7) R(n6,n3) R(n6,n7) R(n7,13) R(n7,n5) R(n7,n6)

-------------------------------------------------------------------------------------------------------------

-> form outgroup SOL6 - A

n2(mod69)

[ cox1 cox2 atp8 atp6 cox3 nad3 -nad5 -nad4 -nad4L -cob -nad6 -nad1 -rrnL -rrnS nad2 ]

n1(mod69)15g

[ cox1 cox2 atp8 atp6 cox3 rrnS rrnL nad1 nad6 cob nad4L nad4 nad5 nad3 nad2 ]

n1(mod69)13g

[ cox1 cox2 cox3 rrnS rrnL nad1 nad6 cob nad4L nad4 nad5 nad3 nad2 ]

score = 264:

model 69:

-------------

R(0,1) R(0,9) R(0,10) R(0,17) R(0,18) R(0,24) R(0,25) R(0,28) R(0,n1) R(1,0) R(2,17) R(3,19) R(3,20) R(4,21) R(5,23) R(6,n2) R(7,n4) R(8,24) R(9,0) R(10,0) R(11,27) R(12,31) R(13,14) R(13,15) R(14,13) R(15,13) R(15,n6) R(15,n7) R(16,n5) R(17,0) R(17,2) R(18,0) R(18,19) R(19,3) R(19,18) R(19,22) R(20,3) R(20,21) R(21,4) R(21,20) R(22,19) R(22,23) R(23,5) R(23,22) R(24,0) R(24,8) R(25,0) R(25,26) R(26,25) R(26,27) R(27,11) R(27,26) R(28,0) R(28,29) R(29,28) R(29,30) R(30,29) R(30,31) R(31,12) R(31,30) R(n1,0) R(n1,n2) R(n1,n3) R(n2,6) R(n2,n1) R(n2,n4) R(n3,n1) R(n3,n6) R(n4,7) R(n4,n2) R(n5,16) R(n5,n7) R(n6,15) R(n6,n3) R(n7,15) R(n7,n5)

-------------------------------------------------------------------------------------------------------------

-> form outgroup SOL6 - A

n2(mod70)

[ cox1 cox2 atp8 atp6 cox3 nad3 -nad5 -nad4 -nad4L -cob -nad6 -nad1 -rrnL -rrnS nad2 ]

n1(mod70)15g

[ cox1 cox2 atp8 atp6 cox3 rrnS rrnL nad1 nad6 cob nad4L nad4 nad5 nad3 nad2 ]

n1(mod70)13g

[ cox1 cox2 cox3 rrnS rrnL nad1 nad6 cob nad4L nad4 nad5 nad3 nad2 ]

score = 276:

model 70:

-------------

R(0,1) R(0,9) R(0,10) R(0,17) R(0,18) R(0,24) R(0,25) R(0,28) R(0,n1) R(1,0) R(2,17) R(3,19) R(3,20) R(4,21) R(5,23) R(6,n2) R(7,n4) R(8,24) R(9,0) R(10,0) R(11,27) R(12,31) R(13,14) R(13,15) R(13,n7) R(14,13) R(15,13) R(15,n6) R(16,n5) R(17,0) R(17,2) R(18,0) R(18,19) R(19,3) R(19,18) R(19,22) R(20,3) R(20,21) R(21,4) R(21,20) R(22,19) R(22,23) R(23,5) R(23,22) R(24,0) R(24,8) R(25,0) R(25,26) R(26,25) R(26,27) R(27,11) R(27,26) R(28,0) R(28,29) R(29,28) R(29,30) R(30,29) R(30,31) R(31,12) R(31,30) R(n1,0) R(n1,n2) R(n1,n3) R(n2,6) R(n2,n1) R(n2,n4) R(n3,n1) R(n3,n6) R(n4,7) R(n4,n2) R(n5,16) R(n5,n7) R(n6,15) R(n6,n3) R(n7,13) R(n7,n5)

-------------------------------------------------------------------------------------------------------------

-> form outgroup SOL6 - A

n2(mod71)

[ cox1 cox2 atp8 atp6 cox3 nad3 -nad5 -nad4 -nad4L -cob -nad6 -nad1 -rrnL -rrnS nad2 ]

n1(mod71)15g

[ cox1 cox2 atp8 atp6 cox3 rrnS rrnL nad1 nad6 cob nad4L nad4 nad5 nad3 nad2 ]

n1(mod71)13g

[ cox1 cox2 cox3 rrnS rrnL nad1 nad6 cob nad4L nad4 nad5 nad3 nad2 ]

score = 250:

model 71:

-------------

R(0,1) R(0,9) R(0,10) R(0,17) R(0,18) R(0,24) R(0,25) R(0,28) R(0,n1) R(1,0) R(2,17) R(3,19) R(3,20) R(4,21) R(5,23) R(6,n2) R(7,n4) R(8,24) R(9,0) R(10,0) R(11,27) R(12,31) R(13,14) R(13,15) R(13,n6) R(13,n7) R(14,13) R(15,13) R(16,n5) R(17,0) R(17,2) R(18,0) R(18,19) R(19,3) R(19,18) R(19,22) R(20,3) R(20,21) R(21,4) R(21,20) R(22,19) R(22,23) R(23,5) R(23,22) R(24,0) R(24,8) R(25,0) R(25,26) R(26,25) R(26,27) R(27,11) R(27,26) R(28,0) R(28,29) R(29,28) R(29,30) R(30,29) R(30,31) R(31,12) R(31,30) R(n1,0) R(n1,n2) R(n1,n3) R(n2,6) R(n2,n1) R(n2,n4) R(n3,n1) R(n3,n6) R(n4,7) R(n4,n2) R(n5,16) R(n5,n7) R(n6,13) R(n6,n3) R(n7,13) R(n7,n5)

-------------------------------------------------------------------------------------------------------------

-> form outgroup SOL6 - A

n2(mod72)

[ cox1 cox2 atp8 atp6 cox3 nad3 -nad5 -nad4 -nad4L -cob -nad6 -nad1 -rrnL -rrnS nad2 ]

n1(mod72)15g

[ cox1 cox2 atp8 atp6 cox3 rrnS rrnL nad1 nad6 cob nad4L nad4 nad5 nad3 nad2 ]

n1(mod72)13g

[ cox1 cox2 cox3 rrnS rrnL nad1 nad6 cob nad4L nad4 nad5 nad3 nad2 ]

score = 264:

model 72:

-------------

R(0,1) R(0,9) R(0,10) R(0,17) R(0,18) R(0,24) R(0,25) R(0,28) R(0,n1) R(1,0) R(2,17) R(3,19) R(3,20) R(4,21) R(5,23) R(6,n2) R(7,n4) R(8,24) R(9,0) R(10,0) R(11,27) R(12,31) R(13,14) R(13,15) R(13,n6) R(14,13) R(15,13) R(15,n7) R(16,n5) R(17,0) R(17,2) R(18,0) R(18,19) R(19,3) R(19,18) R(19,22) R(20,3) R(20,21) R(21,4) R(21,20) R(22,19) R(22,23) R(23,5) R(23,22) R(24,0) R(24,8) R(25,0) R(25,26) R(26,25) R(26,27) R(27,11) R(27,26) R(28,0) R(28,29) R(29,28) R(29,30) R(30,29) R(30,31) R(31,12) R(31,30) R(n1,0) R(n1,n2) R(n1,n3) R(n2,6) R(n2,n1) R(n2,n4) R(n3,n1) R(n3,n6) R(n4,7) R(n4,n2) R(n5,16) R(n5,n7) R(n6,13) R(n6,n3) R(n7,15) R(n7,n5)

-------------------------------------------------------------------------------------------------------------

-> form outgroup SOL6 - A

n2(mod73)

[ cox1 cox2 atp8 atp6 cox3 nad3 -nad5 -nad4 -nad4L -cob -nad6 -nad1 -rrnL -rrnS nad2 ]

n1(mod73)15g

[ cox1 cox2 atp8 atp6 cox3 rrnS rrnL nad1 nad6 cob nad4L nad4 nad5 nad3 nad2 ]

n1(mod73)13g

[ cox1 cox2 cox3 rrnS rrnL nad1 nad6 cob nad4L nad4 nad5 nad3 nad2 ]

n6(mod73)

[ cox1 cox2 nad1 nad3 nad2 cox3 rrnS -nad5 -nad4 -nad4L -cob -nad6 rrnL ]

score = 228:

model 73:

-------------

R(0,1) R(0,9) R(0,10) R(0,17) R(0,18) R(0,24) R(0,25) R(0,28) R(0,n1) R(1,0) R(2,17) R(3,19) R(3,20) R(4,21) R(5,23) R(6,n2) R(7,n4) R(8,24) R(9,0) R(10,0) R(11,27) R(12,31) R(13,14) R(13,n6) R(14,13) R(15,n6) R(16,n5) R(17,0) R(17,2) R(18,0) R(18,19) R(19,3) R(19,18) R(19,22) R(20,3) R(20,21) R(21,4) R(21,20) R(22,19) R(22,23) R(23,5) R(23,22) R(24,0) R(24,8) R(25,0) R(25,26) R(26,25) R(26,27) R(27,11) R(27,26) R(28,0) R(28,29) R(29,28) R(29,30) R(30,29) R(30,31) R(31,12) R(31,30) R(n1,0) R(n1,n2) R(n1,n3) R(n2,6) R(n2,n1) R(n2,n4) R(n3,n1) R(n3,n6) R(n4,7) R(n4,n2) R(n5,16) R(n5,n7) R(n6,13) R(n6,15) R(n6,n3) R(n6,n7) R(n7,n5) R(n7,n6)

-------------------------------------------------------------------------------------------------------------

-> form outgroup SOL6 - A

n2(mod74)

[ cox1 cox2 atp8 atp6 cox3 nad3 -nad5 -nad4 -nad4L -cob -nad6 -nad1 -rrnL -rrnS nad2 ]

n1(mod74)15g

[ cox1 cox2 atp8 atp6 cox3 rrnS rrnL nad1 nad6 cob nad4L nad4 nad5 nad3 nad2 ]

n1(mod74)13g

[ cox1 cox2 cox3 rrnS rrnL nad1 nad6 cob nad4L nad4 nad5 nad3 nad2 ]

n6(mod74)

[ cox1 cox2 nad1 nad3 nad2 cox3 rrnS -nad5 -nad4 -nad4L -cob -nad6 rrnL ]

n7(mod74)

[ cox1 cox2 nad1 nad3 nad2 cox3 rrnS nad5 nad4 -nad4L -cob -nad6 rrnL ]

score = 254:

model 74:

-------------

R(0,1) R(0,9) R(0,10) R(0,17) R(0,18) R(0,24) R(0,25) R(0,28) R(0,n1) R(1,0) R(2,17) R(3,19) R(3,20) R(4,21) R(5,23) R(6,n2) R(7,n4) R(8,24) R(9,0) R(10,0) R(11,27) R(12,31) R(13,14) R(13,n7) R(14,13) R(15,n6) R(16,n5) R(17,0) R(17,2) R(18,0) R(18,19) R(19,3) R(19,18) R(19,22) R(20,3) R(20,21) R(21,4) R(21,20) R(22,19) R(22,23) R(23,5) R(23,22) R(24,0) R(24,8) R(25,0) R(25,26) R(26,25) R(26,27) R(27,11) R(27,26) R(28,0) R(28,29) R(29,28) R(29,30) R(30,29) R(30,31) R(31,12) R(31,30) R(n1,0) R(n1,n2) R(n1,n3) R(n2,6) R(n2,n1) R(n2,n4) R(n3,n1) R(n3,n6) R(n4,7) R(n4,n2) R(n5,16) R(n5,n7) R(n6,15) R(n6,n3) R(n6,n7) R(n7,13) R(n7,n5) R(n7,n6)

-------------------------------------------------------------------------------------------------------------

-> form outgroup SOL6 - A

n2(mod75)

[ cox1 cox2 atp8 atp6 cox3 nad3 -nad5 -nad4 -nad4L -cob -nad6 -nad1 -rrnL -rrnS nad2 ]

n1(mod75)15g

[ cox1 cox2 atp8 atp6 cox3 rrnS rrnL nad1 nad6 cob nad4L nad4 nad5 nad3 nad2 ]

n1(mod75)13g

[ cox1 cox2 cox3 rrnS rrnL nad1 nad6 cob nad4L nad4 nad5 nad3 nad2 ]

n7(mod75)

[ cox1 cox2 nad1 nad3 nad2 cox3 rrnS -nad4L -nad4 -nad5 -cob -nad6 rrnL ]

score = 290:

model 75:

-------------

R(0,1) R(0,9) R(0,10) R(0,17) R(0,18) R(0,24) R(0,25) R(0,28) R(0,n1) R(1,0) R(2,17) R(3,19) R(3,20) R(4,21) R(5,23) R(6,n2) R(7,n4) R(8,24) R(9,0) R(10,0) R(11,27) R(12,31) R(13,14) R(13,n7) R(14,13) R(15,n6) R(15,n7) R(16,n5) R(17,0) R(17,2) R(18,0) R(18,19) R(19,3) R(19,18) R(19,22) R(20,3) R(20,21) R(21,4) R(21,20) R(22,19) R(22,23) R(23,5) R(23,22) R(24,0) R(24,8) R(25,0) R(25,26) R(26,25) R(26,27) R(27,11) R(27,26) R(28,0) R(28,29) R(29,28) R(29,30) R(30,29) R(30,31) R(31,12) R(31,30) R(n1,0) R(n1,n2) R(n1,n3) R(n2,6) R(n2,n1) R(n2,n4) R(n3,n1) R(n3,n6) R(n4,7) R(n4,n2) R(n5,16) R(n5,n7) R(n6,15) R(n6,n3) R(n7,13) R(n7,15) R(n7,n5)

-------------------------------------------------------------------------------------------------------------

-> form outgroup SOL6 - A

n2(mod76)

[ cox1 cox2 atp8 atp6 cox3 nad3 -nad5 -nad4 -nad4L -cob -nad6 -nad1 -rrnL -rrnS nad2 ]

n1(mod76)15g

[ cox1 cox2 atp8 atp6 cox3 rrnS rrnL nad1 nad6 cob nad4L nad4 nad5 nad3 nad2 ]

n1(mod76)13g

[ cox1 cox2 cox3 rrnS rrnL nad1 nad6 cob nad4L nad4 nad5 nad3 nad2 ]

n6(mod76)

[ cox1 cox2 nad1 nad3 nad2 cox3 rrnS -nad5 -nad4 -nad4L -cob -nad6 rrnL ]

score = 242:

model 76:

-------------

R(0,1) R(0,9) R(0,10) R(0,17) R(0,18) R(0,24) R(0,25) R(0,28) R(0,n1) R(1,0) R(2,17) R(3,19) R(3,20) R(4,21) R(5,23) R(6,n2) R(7,n4) R(8,24) R(9,0) R(10,0) R(11,27) R(12,31) R(13,14) R(13,n6) R(14,13) R(15,n6) R(15,n7) R(16,n5) R(17,0) R(17,2) R(18,0) R(18,19) R(19,3) R(19,18) R(19,22) R(20,3) R(20,21) R(21,4) R(21,20) R(22,19) R(22,23) R(23,5) R(23,22) R(24,0) R(24,8) R(25,0) R(25,26) R(26,25) R(26,27) R(27,11) R(27,26) R(28,0) R(28,29) R(29,28) R(29,30) R(30,29) R(30,31) R(31,12) R(31,30) R(n1,0) R(n1,n2) R(n1,n3) R(n2,6) R(n2,n1) R(n2,n4) R(n3,n1) R(n3,n6) R(n4,7) R(n4,n2) R(n5,16) R(n5,n7) R(n6,13) R(n6,15) R(n6,n3) R(n7,15) R(n7,n5)

-------------------------------------------------------------------------------------------------------------

-> form outgroup SOL6 - A

n2(mod77)

[ cox1 cox2 atp8 atp6 cox3 nad3 -nad5 -nad4 -nad4L -cob -nad6 -nad1 -rrnL -rrnS nad2 ]

n1(mod77)15g

[ cox1 cox2 atp8 atp6 cox3 rrnS rrnL nad1 nad6 cob nad4L nad4 nad5 nad3 nad2 ]

n1(mod77)13g

[ cox1 cox2 cox3 rrnS rrnL nad1 nad6 cob nad4L nad4 nad5 nad3 nad2 ]

n7(mod77)

[ cox1 cox2 nad1 nad3 nad2 cox3 rrnS -nad4L -nad4 -nad5 -cob -nad6 rrnL ]

score = 264:

model 77:

-------------

R(0,1) R(0,9) R(0,10) R(0,17) R(0,18) R(0,24) R(0,25) R(0,28) R(0,n1) R(1,0) R(2,17) R(3,19) R(3,20) R(4,21) R(5,23) R(6,n2) R(7,n4) R(8,24) R(9,0) R(10,0) R(11,27) R(12,31) R(13,14) R(13,n6) R(13,n7) R(14,13) R(15,n7) R(16,n5) R(17,0) R(17,2) R(18,0) R(18,19) R(19,3) R(19,18) R(19,22) R(20,3) R(20,21) R(21,4) R(21,20) R(22,19) R(22,23) R(23,5) R(23,22) R(24,0) R(24,8) R(25,0) R(25,26) R(26,25) R(26,27) R(27,11) R(27,26) R(28,0) R(28,29) R(29,28) R(29,30) R(30,29) R(30,31) R(31,12) R(31,30) R(n1,0) R(n1,n2) R(n1,n3) R(n2,6) R(n2,n1) R(n2,n4) R(n3,n1) R(n3,n6) R(n4,7) R(n4,n2) R(n5,16) R(n5,n7) R(n6,13) R(n6,n3) R(n7,13) R(n7,15) R(n7,n5)

-------------------------------------------------------------------------------------------------------------

-> form outgroup SOL6 - A

n2(mod78)

[ cox1 cox2 atp8 atp6 cox3 nad3 -nad5 -nad4 -nad4L -cob -nad6 -nad1 -rrnL -rrnS nad2 ]

n1(mod78)15g

[ cox1 cox2 atp8 atp6 cox3 rrnS rrnL nad1 nad6 cob nad4L nad4 nad5 nad3 nad2 ]

n1(mod78)13g

[ cox1 cox2 cox3 rrnS rrnL nad1 nad6 cob nad4L nad4 nad5 nad3 nad2 ]

n6(mod78)

[ cox1 cox2 nad1 nad3 nad2 cox3 rrnS -nad5 -nad4 -nad4L -cob -nad6 rrnL ]

score = 240:

model 78:

-------------

R(0,1) R(0,9) R(0,10) R(0,17) R(0,18) R(0,24) R(0,25) R(0,28) R(0,n1) R(1,0) R(2,17) R(3,19) R(3,20) R(4,21) R(5,23) R(6,n2) R(7,n4) R(8,24) R(9,0) R(10,0) R(11,27) R(12,31) R(13,14) R(13,n6) R(13,n7) R(14,13) R(15,n6) R(16,n5) R(17,0) R(17,2) R(18,0) R(18,19) R(19,3) R(19,18) R(19,22) R(20,3) R(20,21) R(21,4) R(21,20) R(22,19) R(22,23) R(23,5) R(23,22) R(24,0) R(24,8) R(25,0) R(25,26) R(26,25) R(26,27) R(27,11) R(27,26) R(28,0) R(28,29) R(29,28) R(29,30) R(30,29) R(30,31) R(31,12) R(31,30) R(n1,0) R(n1,n2) R(n1,n3) R(n2,6) R(n2,n1) R(n2,n4) R(n3,n1) R(n3,n6) R(n4,7) R(n4,n2) R(n5,16) R(n5,n7) R(n6,13) R(n6,15) R(n6,n3) R(n7,13) R(n7,n5)

-------------------------------------------------------------------------------------------------------------

-> form outgroup SOL3 - B

n1(mod79)15g

[ cox1 cox2 atp8 atp6 cox3 nad3 -nad5 -nad4 -nad4L -cob -nad6 -nad1 -rrnL -rrnS nad2 ]

n1(mod79)13g

[ cox1 cox2 cox3 nad3 -nad5 -nad4 -nad4L -cob -nad6 -nad1 -rrnL -rrnS nad2 ]

score = 272:

model 79:

-------------

R(0,1) R(0,9) R(0,10) R(0,17) R(0,18) R(0,24) R(0,25) R(0,28) R(0,n1) R(1,0) R(2,17) R(3,19) R(3,20) R(4,21) R(5,23) R(6,n1) R(7,n3) R(8,24) R(9,0) R(10,0) R(11,27) R(12,31) R(13,14) R(13,15) R(13,n4) R(13,n5) R(14,13) R(15,13) R(16,n6) R(17,0) R(17,2) R(18,0) R(18,19) R(19,3) R(19,18) R(19,22) R(20,3) R(20,21) R(21,4) R(21,20) R(22,19) R(22,23) R(23,5) R(23,22) R(24,0) R(24,8) R(25,0) R(25,26) R(26,25) R(26,27) R(27,11) R(27,26) R(28,0) R(28,29) R(29,28) R(29,30) R(30,29) R(30,31) R(31,12) R(31,30) R(n1,0) R(n1,6) R(n1,n2) R(n1,n3) R(n2,n1) R(n2,n7) R(n3,7) R(n3,n1) R(n4,13) R(n4,n6) R(n5,13) R(n5,n7) R(n6,16) R(n6,n4) R(n7,n2) R(n7,n5)

-------------------------------------------------------------------------------------------------------------

-> form outgroup SOL3 - B

n1(mod80)15g

[ cox1 cox2 atp8 atp6 cox3 nad3 -nad5 -nad4 -nad4L -cob -nad6 -nad1 -rrnL -rrnS nad2 ]

n1(mod80)13g

[ cox1 cox2 cox3 nad3 -nad5 -nad4 -nad4L -cob -nad6 -nad1 -rrnL -rrnS nad2 ]

n4(mod80)

[ cox1 cox2 nad1 nad3 nad2 cox3 rrnS -nad4L -nad4 -nad5 -cob -nad6 rrnL ]

score = 286:

model 80:

-------------

R(0,1) R(0,9) R(0,10) R(0,17) R(0,18) R(0,24) R(0,25) R(0,28) R(0,n1) R(1,0) R(2,17) R(3,19) R(3,20) R(4,21) R(5,23) R(6,n1) R(7,n3) R(8,24) R(9,0) R(10,0) R(11,27) R(12,31) R(13,14) R(13,n4) R(13,n5) R(14,13) R(15,n4) R(16,n6) R(17,0) R(17,2) R(18,0) R(18,19) R(19,3) R(19,18) R(19,22) R(20,3) R(20,21) R(21,4) R(21,20) R(22,19) R(22,23) R(23,5) R(23,22) R(24,0) R(24,8) R(25,0) R(25,26) R(26,25) R(26,27) R(27,11) R(27,26) R(28,0) R(28,29) R(29,28) R(29,30) R(30,29) R(30,31) R(31,12) R(31,30) R(n1,0) R(n1,6) R(n1,n2) R(n1,n3) R(n2,n1) R(n2,n7) R(n3,7) R(n3,n1) R(n4,13) R(n4,15) R(n4,n6) R(n5,13) R(n5,n7) R(n6,16) R(n6,n4) R(n7,n2) R(n7,n5)

-------------------------------------------------------------------------------------------------------------

-> form outgroup SOL3 - B

n1(mod81)15g

[ cox1 cox2 atp8 atp6 cox3 nad3 -nad5 -nad4 -nad4L -cob -nad6 -nad1 -rrnL -rrnS nad2 ]

n1(mod81)13g

[ cox1 cox2 cox3 nad3 -nad5 -nad4 -nad4L -cob -nad6 -nad1 -rrnL -rrnS nad2 ]

n5(mod81)

[ cox1 cox2 nad1 nad3 nad2 cox3 rrnS -nad5 -nad4 -nad4L -cob -nad6 rrnL ]

score = 262:

model 81:

-------------

R(0,1) R(0,9) R(0,10) R(0,17) R(0,18) R(0,24) R(0,25) R(0,28) R(0,n1) R(1,0) R(2,17) R(3,19) R(3,20) R(4,21) R(5,23) R(6,n1) R(7,n3) R(8,24) R(9,0) R(10,0) R(11,27) R(12,31) R(13,14) R(13,n4) R(13,n5) R(14,13) R(15,n5) R(16,n6) R(17,0) R(17,2) R(18,0) R(18,19) R(19,3) R(19,18) R(19,22) R(20,3) R(20,21) R(21,4) R(21,20) R(22,19) R(22,23) R(23,5) R(23,22) R(24,0) R(24,8) R(25,0) R(25,26) R(26,25) R(26,27) R(27,11) R(27,26) R(28,0) R(28,29) R(29,28) R(29,30) R(30,29) R(30,31) R(31,12) R(31,30) R(n1,0) R(n1,6) R(n1,n2) R(n1,n3) R(n2,n1) R(n2,n7) R(n3,7) R(n3,n1) R(n4,13) R(n4,n6) R(n5,13) R(n5,15) R(n5,n7) R(n6,16) R(n6,n4) R(n7,n2) R(n7,n5)

-------------------------------------------------------------------------------------------------------------

-> form outgroup SOL1 - B

n3(mod82)

[ cox1 cox2 atp8 atp6 cox3 nad3 -nad5 -nad4 -nad4L nad6 cob rrnS rrnL nad1 nad2 ]

n1(mod82)15g

[ cox1 cox2 atp8 atp6 cox3 nad3 -nad5 -nad4 -nad4L -cob -nad6 -nad1 -rrnL -rrnS nad2 ]

n1(mod82)13g

[ cox1 cox2 cox3 nad3 -nad5 -nad4 -nad4L -cob -nad6 -nad1 -rrnL -rrnS nad2 ]

score = 286:

model 82:

-------------

R(0,1) R(0,9) R(0,10) R(0,17) R(0,18) R(0,24) R(0,25) R(0,28) R(0,n1) R(1,0) R(2,17) R(3,19) R(3,20) R(4,21) R(5,23) R(6,n3) R(7,n3) R(8,24) R(9,0) R(10,0) R(11,27) R(12,31) R(13,14) R(13,15) R(13,n4) R(13,n5) R(14,13) R(15,13) R(16,n6) R(17,0) R(17,2) R(18,0) R(18,19) R(19,3) R(19,18) R(19,22) R(20,3) R(20,21) R(21,4) R(21,20) R(22,19) R(22,23) R(23,5) R(23,22) R(24,0) R(24,8) R(25,0) R(25,26) R(26,25) R(26,27) R(27,11) R(27,26) R(28,0) R(28,29) R(29,28) R(29,30) R(30,29) R(30,31) R(31,12) R(31,30) R(n1,0) R(n1,n2) R(n1,n3) R(n2,n1) R(n2,n7) R(n3,6) R(n3,7) R(n3,n1) R(n4,13) R(n4,n6) R(n5,13) R(n5,n7) R(n6,16) R(n6,n4) R(n7,n2) R(n7,n5)

-------------------------------------------------------------------------------------------------------------

-> form outgroup SOL1 - B

n3(mod83)

[ cox1 cox2 atp8 atp6 cox3 nad3 -nad5 -nad4 -nad4L nad6 cob rrnS rrnL nad1 nad2 ]

n1(mod83)15g

[ cox1 cox2 atp8 atp6 cox3 nad3 -nad5 -nad4 -nad4L -cob -nad6 -nad1 -rrnL -rrnS nad2 ]

n1(mod83)13g

[ cox1 cox2 cox3 nad3 -nad5 -nad4 -nad4L -cob -nad6 -nad1 -rrnL -rrnS nad2 ]

n4(mod83)

[ cox1 cox2 nad1 nad3 nad2 cox3 rrnS -nad4L -nad4 -nad5 -cob -nad6 rrnL ]

score = 300:

model 83:

-------------

R(0,1) R(0,9) R(0,10) R(0,17) R(0,18) R(0,24) R(0,25) R(0,28) R(0,n1) R(1,0) R(2,17) R(3,19) R(3,20) R(4,21) R(5,23) R(6,n3) R(7,n3) R(8,24) R(9,0) R(10,0) R(11,27) R(12,31) R(13,14) R(13,n4) R(13,n5) R(14,13) R(15,n4) R(16,n6) R(17,0) R(17,2) R(18,0) R(18,19) R(19,3) R(19,18) R(19,22) R(20,3) R(20,21) R(21,4) R(21,20) R(22,19) R(22,23) R(23,5) R(23,22) R(24,0) R(24,8) R(25,0) R(25,26) R(26,25) R(26,27) R(27,11) R(27,26) R(28,0) R(28,29) R(29,28) R(29,30) R(30,29) R(30,31) R(31,12) R(31,30) R(n1,0) R(n1,n2) R(n1,n3) R(n2,n1) R(n2,n7) R(n3,6) R(n3,7) R(n3,n1) R(n4,13) R(n4,15) R(n4,n6) R(n5,13) R(n5,n7) R(n6,16) R(n6,n4) R(n7,n2) R(n7,n5)

-------------------------------------------------------------------------------------------------------------

-> form outgroup SOL1 - B

n3(mod84)

[ cox1 cox2 atp8 atp6 cox3 nad3 -nad5 -nad4 -nad4L nad6 cob rrnS rrnL nad1 nad2 ]

n1(mod84)15g

[ cox1 cox2 atp8 atp6 cox3 nad3 -nad5 -nad4 -nad4L -cob -nad6 -nad1 -rrnL -rrnS nad2 ]

n1(mod84)13g

[ cox1 cox2 cox3 nad3 -nad5 -nad4 -nad4L -cob -nad6 -nad1 -rrnL -rrnS nad2 ]

n5(mod84)

[ cox1 cox2 nad1 nad3 nad2 cox3 rrnS -nad5 -nad4 -nad4L -cob -nad6 rrnL ]

score = 276:

model 84:

-------------

R(0,1) R(0,9) R(0,10) R(0,17) R(0,18) R(0,24) R(0,25) R(0,28) R(0,n1) R(1,0) R(2,17) R(3,19) R(3,20) R(4,21) R(5,23) R(6,n3) R(7,n3) R(8,24) R(9,0) R(10,0) R(11,27) R(12,31) R(13,14) R(13,n4) R(13,n5) R(14,13) R(15,n5) R(16,n6) R(17,0) R(17,2) R(18,0) R(18,19) R(19,3) R(19,18) R(19,22) R(20,3) R(20,21) R(21,4) R(21,20) R(22,19) R(22,23) R(23,5) R(23,22) R(24,0) R(24,8) R(25,0) R(25,26) R(26,25) R(26,27) R(27,11) R(27,26) R(28,0) R(28,29) R(29,28) R(29,30) R(30,29) R(30,31) R(31,12) R(31,30) R(n1,0) R(n1,n2) R(n1,n3) R(n2,n1) R(n2,n7) R(n3,6) R(n3,7) R(n3,n1) R(n4,13) R(n4,n6) R(n5,13) R(n5,15) R(n5,n7) R(n6,16) R(n6,n4) R(n7,n2) R(n7,n5)

-------------------------------------------------------------------------------------------------------------

-> form outgroup SOL3 - B

n1(mod85)15g

[ cox1 cox2 atp8 atp6 cox3 nad3 -nad5 -nad4 -nad4L -cob -nad6 -nad1 -rrnL -rrnS nad2 ]

n1(mod85)13g

[ cox1 cox2 cox3 nad3 -nad5 -nad4 -nad4L -cob -nad6 -nad1 -rrnL -rrnS nad2 ]

n4(mod85)

g1: [ cox1 cox2 nad1 nad3 nad2 cox3 rrnS -nad4 -nad5 -cob -nad6 -nad4L rrnL ]

g2: [ cox1 cox2 nad1 nad3 nad2 cox3 rrnS nad5 nad4 -nad4L -cob -nad6 rrnL ]

g3: [ cox1 cox2 nad1 nad3 nad2 cox3 rrnS rrnL nad6 cob nad4L nad5 nad4 ]

g4: [ cox1 cox2 nad1 nad3 nad2 cox3 rrnS nad6 cob nad4L -nad4 -nad5 rrnL ]

g5: [ cox1 cox2 nad1 nad3 nad2 cox3 rrnS -nad4 -nad5 nad6 cob nad4L rrnL ]

score = 298:

model 85:

-------------

R(0,1) R(0,9) R(0,10) R(0,17) R(0,18) R(0,24) R(0,25) R(0,28) R(0,n1) R(1,0) R(2,17) R(3,19) R(3,20) R(4,21) R(5,23) R(6,n1) R(7,n3) R(8,24) R(9,0) R(10,0) R(11,27) R(12,31) R(13,14) R(13,15) R(13,n4) R(14,13) R(15,13) R(16,n5) R(17,0) R(17,2) R(18,0) R(18,19) R(19,3) R(19,18) R(19,22) R(20,3) R(20,21) R(21,4) R(21,20) R(22,19) R(22,23) R(23,5) R(23,22) R(24,0) R(24,8) R(25,0) R(25,26) R(26,25) R(26,27) R(27,11) R(27,26) R(28,0) R(28,29) R(29,28) R(29,30) R(30,29) R(30,31) R(31,12) R(31,30) R(n1,0) R(n1,6) R(n1,n2) R(n1,n3) R(n2,n1) R(n2,n7) R(n3,7) R(n3,n1) R(n4,13) R(n4,n5) R(n4,n6) R(n5,16) R(n5,n4) R(n6,n4) R(n6,n7) R(n7,n2) R(n7,n6)

-------------------------------------------------------------------------------------------------------------

-> form outgroup SOL3 - B

n1(mod86)15g

[ cox1 cox2 atp8 atp6 cox3 nad3 -nad5 -nad4 -nad4L -cob -nad6 -nad1 -rrnL -rrnS nad2 ]

n1(mod86)13g

[ cox1 cox2 cox3 nad3 -nad5 -nad4 -nad4L -cob -nad6 -nad1 -rrnL -rrnS nad2 ]

score = 298:

model 86:

-------------

R(0,1) R(0,9) R(0,10) R(0,17) R(0,18) R(0,24) R(0,25) R(0,28) R(0,n1) R(1,0) R(2,17) R(3,19) R(3,20) R(4,21) R(5,23) R(6,n1) R(7,n3) R(8,24) R(9,0) R(10,0) R(11,27) R(12,31) R(13,14) R(13,15) R(13,n4) R(14,13) R(15,13) R(15,n6) R(16,n5) R(17,0) R(17,2) R(18,0) R(18,19) R(19,3) R(19,18) R(19,22) R(20,3) R(20,21) R(21,4) R(21,20) R(22,19) R(22,23) R(23,5) R(23,22) R(24,0) R(24,8) R(25,0) R(25,26) R(26,25) R(26,27) R(27,11) R(27,26) R(28,0) R(28,29) R(29,28) R(29,30) R(30,29) R(30,31) R(31,12) R(31,30) R(n1,0) R(n1,6) R(n1,n2) R(n1,n3) R(n2,n1) R(n2,n7) R(n3,7) R(n3,n1) R(n4,13) R(n4,n5) R(n5,16) R(n5,n4) R(n6,15) R(n6,n7) R(n7,n2) R(n7,n6)

-------------------------------------------------------------------------------------------------------------

-> form outgroup SOL3 - B

n1(mod87)15g

[ cox1 cox2 atp8 atp6 cox3 nad3 -nad5 -nad4 -nad4L -cob -nad6 -nad1 -rrnL -rrnS nad2 ]

n1(mod87)13g

[ cox1 cox2 cox3 nad3 -nad5 -nad4 -nad4L -cob -nad6 -nad1 -rrnL -rrnS nad2 ]

score = 286:

model 87:

-------------

R(0,1) R(0,9) R(0,10) R(0,17) R(0,18) R(0,24) R(0,25) R(0,28) R(0,n1) R(1,0) R(2,17) R(3,19) R(3,20) R(4,21) R(5,23) R(6,n1) R(7,n2) R(8,24) R(9,0) R(10,0) R(11,27) R(12,31) R(13,14) R(13,15) R(13,n4) R(14,13) R(15,13) R(15,n7) R(16,n5) R(17,0) R(17,2) R(18,0) R(18,19) R(19,3) R(19,18) R(19,22) R(20,3) R(20,21) R(21,4) R(21,20) R(22,19) R(22,23) R(23,5) R(23,22) R(24,0) R(24,8) R(25,0) R(25,26) R(26,25) R(26,27) R(27,11) R(27,26) R(28,0) R(28,29) R(29,28) R(29,30) R(30,29) R(30,31) R(31,12) R(31,30) R(n1,0) R(n1,6) R(n1,n2) R(n1,n3) R(n2,7) R(n2,n1) R(n3,n1) R(n3,n6) R(n4,13) R(n4,n6) R(n5,16) R(n5,n7) R(n6,n3) R(n6,n4) R(n7,15) R(n7,n5)

-------------------------------------------------------------------------------------------------------------

-> form outgroup SOL3 - B

n1(mod88)15g

[ cox1 cox2 atp8 atp6 cox3 nad3 -nad5 -nad4 -nad4L -cob -nad6 -nad1 -rrnL -rrnS nad2 ]

n1(mod88)13g

[ cox1 cox2 cox3 nad3 -nad5 -nad4 -nad4L -cob -nad6 -nad1 -rrnL -rrnS nad2 ]

n4(mod88)

g1: [ cox1 cox2 nad1 nad3 -nad4 -nad5 -nad4L -cob -nad6 nad2 cox3 rrnS rrnL ]

g2: [ cox1 cox2 nad1 nad3 nad2 cox3 rrnS -nad5 -nad4 -nad4L -cob -nad6 rrnL ]

g3: [ cox1 cox2 nad1 nad6 cob nad4L nad5 nad4 nad3 nad2 cox3 rrnS rrnL ]

score = 262:

model 88:

-------------

R(0,1) R(0,9) R(0,10) R(0,17) R(0,18) R(0,24) R(0,25) R(0,28) R(0,n1) R(1,0) R(2,17) R(3,19) R(3,20) R(4,21) R(5,23) R(6,n1) R(7,n2) R(8,24) R(9,0) R(10,0) R(11,27) R(12,31) R(13,14) R(13,15) R(13,n4) R(14,13) R(15,13) R(16,n5) R(17,0) R(17,2) R(18,0) R(18,19) R(19,3) R(19,18) R(19,22) R(20,3) R(20,21) R(21,4) R(21,20) R(22,19) R(22,23) R(23,5) R(23,22) R(24,0) R(24,8) R(25,0) R(25,26) R(26,25) R(26,27) R(27,11) R(27,26) R(28,0) R(28,29) R(29,28) R(29,30) R(30,29) R(30,31) R(31,12) R(31,30) R(n1,0) R(n1,6) R(n1,n2) R(n1,n3) R(n2,7) R(n2,n1) R(n3,n1) R(n3,n6) R(n4,13) R(n4,n6) R(n4,n7) R(n5,16) R(n5,n7) R(n6,n3) R(n6,n4) R(n7,n4) R(n7,n5)

-------------------------------------------------------------------------------------------------------------

-> form outgroup SOL3 - B

n1(mod89)15g

[ cox1 cox2 atp8 atp6 cox3 nad3 -nad5 -nad4 -nad4L -cob -nad6 -nad1 -rrnL -rrnS nad2 ]

n1(mod89)13g

[ cox1 cox2 cox3 nad3 -nad5 -nad4 -nad4L -cob -nad6 -nad1 -rrnL -rrnS nad2 ]

n4(mod89)

[ cox1 cox2 nad1 nad3 nad2 cox3 rrnS -nad4L -nad4 -nad5 -cob -nad6 rrnL ]

score = 312:

model 89:

-------------

R(0,1) R(0,9) R(0,10) R(0,17) R(0,18) R(0,24) R(0,25) R(0,28) R(0,n1) R(1,0) R(2,17) R(3,19) R(3,20) R(4,21) R(5,23) R(6,n1) R(7,n3) R(8,24) R(9,0) R(10,0) R(11,27) R(12,31) R(13,14) R(13,n4) R(14,13) R(15,n4) R(15,n6) R(16,n5) R(17,0) R(17,2) R(18,0) R(18,19) R(19,3) R(19,18) R(19,22) R(20,3) R(20,21) R(21,4) R(21,20) R(22,19) R(22,23) R(23,5) R(23,22) R(24,0) R(24,8) R(25,0) R(25,26) R(26,25) R(26,27) R(27,11) R(27,26) R(28,0) R(28,29) R(29,28) R(29,30) R(30,29) R(30,31) R(31,12) R(31,30) R(n1,0) R(n1,6) R(n1,n2) R(n1,n3) R(n2,n1) R(n2,n7) R(n3,7) R(n3,n1) R(n4,13) R(n4,15) R(n4,n5) R(n5,16) R(n5,n4) R(n6,15) R(n6,n7) R(n7,n2) R(n7,n6)

-------------------------------------------------------------------------------------------------------------

-> form outgroup SOL3 - B

n1(mod90)15g

[ cox1 cox2 atp8 atp6 cox3 nad3 -nad5 -nad4 -nad4L -cob -nad6 -nad1 -rrnL -rrnS nad2 ]

n1(mod90)13g

[ cox1 cox2 cox3 nad3 -nad5 -nad4 -nad4L -cob -nad6 -nad1 -rrnL -rrnS nad2 ]

n4(mod90)

[ cox1 cox2 nad1 nad3 nad2 cox3 rrnS -nad5 -nad4 -nad4L -cob -nad6 rrnL ]

score = 264:

model 90:

-------------

R(0,1) R(0,9) R(0,10) R(0,17) R(0,18) R(0,24) R(0,25) R(0,28) R(0,n1) R(1,0) R(2,17) R(3,19) R(3,20) R(4,21) R(5,23) R(6,n1) R(7,n2) R(8,24) R(9,0) R(10,0) R(11,27) R(12,31) R(13,14) R(13,n4) R(14,13) R(15,n4) R(15,n7) R(16,n5) R(17,0) R(17,2) R(18,0) R(18,19) R(19,3) R(19,18) R(19,22) R(20,3) R(20,21) R(21,4) R(21,20) R(22,19) R(22,23) R(23,5) R(23,22) R(24,0) R(24,8) R(25,0) R(25,26) R(26,25) R(26,27) R(27,11) R(27,26) R(28,0) R(28,29) R(29,28) R(29,30) R(30,29) R(30,31) R(31,12) R(31,30) R(n1,0) R(n1,6) R(n1,n2) R(n1,n3) R(n2,7) R(n2,n1) R(n3,n1) R(n3,n6) R(n4,13) R(n4,15) R(n4,n6) R(n5,16) R(n5,n7) R(n6,n3) R(n6,n4) R(n7,15) R(n7,n5)

-------------------------------------------------------------------------------------------------------------

-> form outgroup SOL3 - B

n1(mod91)15g

[ cox1 cox2 atp8 atp6 cox3 nad3 -nad5 -nad4 -nad4L -cob -nad6 -nad1 -rrnL -rrnS nad2 ]

n1(mod91)13g

[ cox1 cox2 cox3 nad3 -nad5 -nad4 -nad4L -cob -nad6 -nad1 -rrnL -rrnS nad2 ]

n4(mod91)

[ cox1 cox2 nad1 nad3 nad2 cox3 rrnS -nad5 -nad4 -nad4L -cob -nad6 rrnL ]

score = 250:

model 91:

-------------

R(0,1) R(0,9) R(0,10) R(0,17) R(0,18) R(0,24) R(0,25) R(0,28) R(0,n1) R(1,0) R(2,17) R(3,19) R(3,20) R(4,21) R(5,23) R(6,n1) R(7,n2) R(8,24) R(9,0) R(10,0) R(11,27) R(12,31) R(13,14) R(13,n4) R(14,13) R(15,n4) R(16,n5) R(17,0) R(17,2) R(18,0) R(18,19) R(19,3) R(19,18) R(19,22) R(20,3) R(20,21) R(21,4) R(21,20) R(22,19) R(22,23) R(23,5) R(23,22) R(24,0) R(24,8) R(25,0) R(25,26) R(26,25) R(26,27) R(27,11) R(27,26) R(28,0) R(28,29) R(29,28) R(29,30) R(30,29) R(30,31) R(31,12) R(31,30) R(n1,0) R(n1,6) R(n1,n2) R(n1,n3) R(n2,7) R(n2,n1) R(n3,n1) R(n3,n6) R(n4,13) R(n4,15) R(n4,n6) R(n4,n7) R(n5,16) R(n5,n7) R(n6,n3) R(n6,n4) R(n7,n4) R(n7,n5)

-------------------------------------------------------------------------------------------------------------

-> form outgroup SOL3 - B

n1(mod92)15g

[ cox1 cox2 atp8 atp6 cox3 nad3 -nad5 -nad4 -nad4L -cob -nad6 -nad1 -rrnL -rrnS nad2 ]

n1(mod92)13g

[ cox1 cox2 cox3 nad3 -nad5 -nad4 -nad4L -cob -nad6 -nad1 -rrnL -rrnS nad2 ]

n6(mod92)

[ cox1 cox2 nad1 nad3 nad2 cox3 rrnS -nad5 -nad4 -nad4L -cob -nad6 rrnL ]

n4(mod92)

[ cox1 cox2 nad1 nad3 nad2 cox3 rrnS nad5 nad4 -nad4L -cob -nad6 rrnL ]

score = 276:

model 92:

-------------

R(0,1) R(0,9) R(0,10) R(0,17) R(0,18) R(0,24) R(0,25) R(0,28) R(0,n1) R(1,0) R(2,17) R(3,19) R(3,20) R(4,21) R(5,23) R(6,n1) R(7,n3) R(8,24) R(9,0) R(10,0) R(11,27) R(12,31) R(13,14) R(13,n4) R(14,13) R(15,n6) R(16,n5) R(17,0) R(17,2) R(18,0) R(18,19) R(19,3) R(19,18) R(19,22) R(20,3) R(20,21) R(21,4) R(21,20) R(22,19) R(22,23) R(23,5) R(23,22) R(24,0) R(24,8) R(25,0) R(25,26) R(26,25) R(26,27) R(27,11) R(27,26) R(28,0) R(28,29) R(29,28) R(29,30) R(30,29) R(30,31) R(31,12) R(31,30) R(n1,0) R(n1,6) R(n1,n2) R(n1,n3) R(n2,n1) R(n2,n7) R(n3,7) R(n3,n1) R(n4,13) R(n4,n5) R(n4,n6) R(n5,16) R(n5,n4) R(n6,15) R(n6,n4) R(n6,n7) R(n7,n2) R(n7,n6)

-------------------------------------------------------------------------------------------------------------

-> form outgroup SOL1 - B

n3(mod93)

[ cox1 cox2 atp8 atp6 cox3 nad3 -nad5 -nad4 -nad4L nad6 cob rrnS rrnL nad1 nad2 ]

n1(mod93)15g

[ cox1 cox2 atp8 atp6 cox3 nad3 -nad5 -nad4 -nad4L -cob -nad6 -nad1 -rrnL -rrnS nad2 ]

n1(mod93)13g

[ cox1 cox2 cox3 nad3 -nad5 -nad4 -nad4L -cob -nad6 -nad1 -rrnL -rrnS nad2 ]

n4(mod93)

[ cox1 cox2 nad1 nad3 nad2 cox3 rrnS -nad4L -nad4 -nad5 -cob -nad6 rrnL ]

score = 326:

model 93:

-------------

R(0,1) R(0,9) R(0,10) R(0,17) R(0,18) R(0,24) R(0,25) R(0,28) R(0,n1) R(1,0) R(2,17) R(3,19) R(3,20) R(4,21) R(5,23) R(6,n3) R(7,n3) R(8,24) R(9,0) R(10,0) R(11,27) R(12,31) R(13,14) R(13,n4) R(14,13) R(15,n4) R(15,n6) R(16,n5) R(17,0) R(17,2) R(18,0) R(18,19) R(19,3) R(19,18) R(19,22) R(20,3) R(20,21) R(21,4) R(21,20) R(22,19) R(22,23) R(23,5) R(23,22) R(24,0) R(24,8) R(25,0) R(25,26) R(26,25) R(26,27) R(27,11) R(27,26) R(28,0) R(28,29) R(29,28) R(29,30) R(30,29) R(30,31) R(31,12) R(31,30) R(n1,0) R(n1,n2) R(n1,n3) R(n2,n1) R(n2,n7) R(n3,6) R(n3,7) R(n3,n1) R(n4,13) R(n4,15) R(n4,n5) R(n5,16) R(n5,n4) R(n6,15) R(n6,n7) R(n7,n2) R(n7,n6)

-------------------------------------------------------------------------------------------------------------

-> form outgroup SOL1 - B

n2(mod94)

[ cox1 cox2 atp8 atp6 cox3 nad3 -nad5 -nad4 -nad4L nad6 cob rrnS rrnL nad1 nad2 ]

n1(mod94)15g

[ cox1 cox2 atp8 atp6 cox3 nad3 -nad5 -nad4 -nad4L -cob -nad6 -nad1 -rrnL -rrnS nad2 ]

n1(mod94)13g

[ cox1 cox2 cox3 nad3 -nad5 -nad4 -nad4L -cob -nad6 -nad1 -rrnL -rrnS nad2 ]

n4(mod94)

[ cox1 cox2 nad1 nad3 nad2 cox3 rrnS -nad5 -nad4 -nad4L -cob -nad6 rrnL ]

score = 278:

model 94:

-------------

R(0,1) R(0,9) R(0,10) R(0,17) R(0,18) R(0,24) R(0,25) R(0,28) R(0,n1) R(1,0) R(2,17) R(3,19) R(3,20) R(4,21) R(5,23) R(6,n2) R(7,n2) R(8,24) R(9,0) R(10,0) R(11,27) R(12,31) R(13,14) R(13,n4) R(14,13) R(15,n4) R(15,n7) R(16,n5) R(17,0) R(17,2) R(18,0) R(18,19) R(19,3) R(19,18) R(19,22) R(20,3) R(20,21) R(21,4) R(21,20) R(22,19) R(22,23) R(23,5) R(23,22) R(24,0) R(24,8) R(25,0) R(25,26) R(26,25) R(26,27) R(27,11) R(27,26) R(28,0) R(28,29) R(29,28) R(29,30) R(30,29) R(30,31) R(31,12) R(31,30) R(n1,0) R(n1,n2) R(n1,n3) R(n2,6) R(n2,7) R(n2,n1) R(n3,n1) R(n3,n6) R(n4,13) R(n4,15) R(n4,n6) R(n5,16) R(n5,n7) R(n6,n3) R(n6,n4) R(n7,15) R(n7,n5)

-------------------------------------------------------------------------------------------------------------

-> form outgroup SOL1 - B

n2(mod95)

[ cox1 cox2 atp8 atp6 cox3 nad3 -nad5 -nad4 -nad4L nad6 cob rrnS rrnL nad1 nad2 ]

n1(mod95)15g

[ cox1 cox2 atp8 atp6 cox3 nad3 -nad5 -nad4 -nad4L -cob -nad6 -nad1 -rrnL -rrnS nad2 ]

n1(mod95)13g

[ cox1 cox2 cox3 nad3 -nad5 -nad4 -nad4L -cob -nad6 -nad1 -rrnL -rrnS nad2 ]

n4(mod95)

[ cox1 cox2 nad1 nad3 nad2 cox3 rrnS -nad5 -nad4 -nad4L -cob -nad6 rrnL ]

score = 264:

model 95:

-------------

R(0,1) R(0,9) R(0,10) R(0,17) R(0,18) R(0,24) R(0,25) R(0,28) R(0,n1) R(1,0) R(2,17) R(3,19) R(3,20) R(4,21) R(5,23) R(6,n2) R(7,n2) R(8,24) R(9,0) R(10,0) R(11,27) R(12,31) R(13,14) R(13,n4) R(14,13) R(15,n4) R(16,n5) R(17,0) R(17,2) R(18,0) R(18,19) R(19,3) R(19,18) R(19,22) R(20,3) R(20,21) R(21,4) R(21,20) R(22,19) R(22,23) R(23,5) R(23,22) R(24,0) R(24,8) R(25,0) R(25,26) R(26,25) R(26,27) R(27,11) R(27,26) R(28,0) R(28,29) R(29,28) R(29,30) R(30,29) R(30,31) R(31,12) R(31,30) R(n1,0) R(n1,n2) R(n1,n3) R(n2,6) R(n2,7) R(n2,n1) R(n3,n1) R(n3,n6) R(n4,13) R(n4,15) R(n4,n6) R(n4,n7) R(n5,16) R(n5,n7) R(n6,n3) R(n6,n4) R(n7,n4) R(n7,n5)

-------------------------------------------------------------------------------------------------------------

-> form outgroup SOL1 - B

n3(mod96)

[ cox1 cox2 atp8 atp6 cox3 nad3 -nad5 -nad4 -nad4L nad6 cob rrnS rrnL nad1 nad2 ]

n1(mod96)15g

g1[ cox1 cox2 atp8 atp6 -nad5 -nad4 -nad4L nad6 cob rrnS rrnL nad1 cox3 nad3 nad2 ]

g2[ cox1 cox2 atp8 atp6 nad6 cob nad4L nad4 nad5 -nad3 -cox3 rrnS rrnL nad1 nad2 ]

g3[ cox1 cox2 atp8 atp6 cox3 nad3 -nad5 -nad4 -nad4L -cob -nad6 -nad1 -rrnL -rrnS nad2 ]

n1(mod96)13g

g1[ cox1 cox2 -nad5 -nad4 -nad4L nad6 cob rrnS rrnL nad1 cox3 nad3 nad2 ]

g2[ cox1 cox2 nad6 cob nad4L nad4 nad5 -nad3 -cox3 rrnS rrnL nad1 nad2 ]

g3[ cox1 cox2 cox3 nad3 -nad5 -nad4 -nad4L -cob -nad6 -nad1 -rrnL -rrnS nad2 ]

n4(mod96)

for n1-g1:

g1: [ cox1 cox2 nad1 nad3 nad2 cox3 rrnS -nad4 -nad5 -cob -nad6 rrnL -nad4L ]

g2: [ cox1 cox2 nad1 nad3 nad2 cox3 rrnS rrnL nad6 cob nad4L nad5 nad4 ]

g3: [ cox1 cox2 nad1 nad3 nad2 cox3 rrnS -nad4 -nad5 -cob -nad6 nad4L rrnL ]

g4: [ cox1 cox2 nad1 nad3 nad2 cox3 rrnS -nad4 -nad5 -rrnL -nad4L -cob -nad6 ]

for n1-g2:

g5: [ cox1 cox2 nad1 nad3 nad2 cox3 rrnS rrnL nad6 cob nad4L nad5 nad4 ]

g6: [ cox1 cox2 nad1 nad3 nad2 cox3 rrnS -rrnL nad6 cob nad4L nad5 nad4 ]

g7: [ cox1 cox2 nad1 nad3 nad2 cox3 rrnS -rrnL nad6 cob nad4L -nad4 -nad5 ]

for n1-g3:

g8: [ cox1 cox2 nad1 nad3 nad2 cox3 rrnS -nad4 -nad5 -cob -nad6 -nad4L rrnL ]

g9: [ cox1 cox2 nad1 nad3 nad2 cox3 rrnS nad5 nad4 -nad4L -cob -nad6 rrnL ]

g10: [ cox1 cox2 nad1 nad3 nad2 cox3 rrnS rrnL nad6 cob nad4L nad5 nad4 ]

g11: [ cox1 cox2 nad1 nad3 nad2 cox3 rrnS nad6 cob nad4L -nad4 -nad5 rrnL ]

g12: [ cox1 cox2 nad1 nad3 nad2 cox3 rrnS -nad4 -nad5 nad6 cob nad4L rrnL ]

score = 312:

model 96:

-------------

R(0,1) R(0,9) R(0,10) R(0,17) R(0,18) R(0,24) R(0,25) R(0,28) R(0,n1) R(1,0) R(2,17) R(3,19) R(3,20) R(4,21) R(5,23) R(6,n3) R(7,n3) R(8,24) R(9,0) R(10,0) R(11,27) R(12,31) R(13,14) R(13,15) R(13,n4) R(14,13) R(15,13) R(16,n5) R(17,0) R(17,2) R(18,0) R(18,19) R(19,3) R(19,18) R(19,22) R(20,3) R(20,21) R(21,4) R(21,20) R(22,19) R(22,23) R(23,5) R(23,22) R(24,0) R(24,8) R(25,0) R(25,26) R(26,25) R(26,27) R(27,11) R(27,26) R(28,0) R(28,29) R(29,28) R(29,30) R(30,29) R(30,31) R(31,12) R(31,30) R(n1,0) R(n1,n2) R(n1,n3) R(n2,n1) R(n2,n7) R(n3,6) R(n3,7) R(n3,n1) R(n4,13) R(n4,n5) R(n4,n6) R(n5,16) R(n5,n4) R(n6,n4) R(n6,n7) R(n7,n2) R(n7,n6)

-------------------------------------------------------------------------------------------------------------

-> form outgroup SOL1 - B

n3(mod97)

[ cox1 cox2 atp8 atp6 cox3 nad3 -nad5 -nad4 -nad4L nad6 cob rrnS rrnL nad1 nad2 ]

n1(mod97)15g

[ cox1 cox2 atp8 atp6 cox3 nad3 -nad5 -nad4 -nad4L -cob -nad6 -nad1 -rrnL -rrnS nad2 ]

n1(mod97)13g

[ cox1 cox2 cox3 nad3 -nad5 -nad4 -nad4L -cob -nad6 -nad1 -rrnL -rrnS nad2 ]

score = 312:

model 97:

-------------

R(0,1) R(0,9) R(0,10) R(0,17) R(0,18) R(0,24) R(0,25) R(0,28) R(0,n1) R(1,0) R(2,17) R(3,19) R(3,20) R(4,21) R(5,23) R(6,n3) R(7,n3) R(8,24) R(9,0) R(10,0) R(11,27) R(12,31) R(13,14) R(13,15) R(13,n4) R(14,13) R(15,13) R(15,n6) R(16,n5) R(17,0) R(17,2) R(18,0) R(18,19) R(19,3) R(19,18) R(19,22) R(20,3) R(20,21) R(21,4) R(21,20) R(22,19) R(22,23) R(23,5) R(23,22) R(24,0) R(24,8) R(25,0) R(25,26) R(26,25) R(26,27) R(27,11) R(27,26) R(28,0) R(28,29) R(29,28) R(29,30) R(30,29) R(30,31) R(31,12) R(31,30) R(n1,0) R(n1,n2) R(n1,n3) R(n2,n1) R(n2,n7) R(n3,6) R(n3,7) R(n3,n1) R(n4,13) R(n4,n5) R(n5,16) R(n5,n4) R(n6,15) R(n6,n7) R(n7,n2) R(n7,n6)

-------------------------------------------------------------------------------------------------------------

-> form outgroup SOL1 - B

n2(mod98)

[ cox1 cox2 atp8 atp6 cox3 nad3 -nad5 -nad4 -nad4L nad6 cob rrnS rrnL nad1 nad2 ]

n1(mod98)15g

[ cox1 cox2 atp8 atp6 cox3 nad3 -nad5 -nad4 -nad4L -cob -nad6 -nad1 -rrnL -rrnS nad2 ]

n1(mod98)13g

[ cox1 cox2 cox3 nad3 -nad5 -nad4 -nad4L -cob -nad6 -nad1 -rrnL -rrnS nad2 ]

score = 300:

model 98:

-------------

R(0,1) R(0,9) R(0,10) R(0,17) R(0,18) R(0,24) R(0,25) R(0,28) R(0,n1) R(1,0) R(2,17) R(3,19) R(3,20) R(4,21) R(5,23) R(6,n2) R(7,n2) R(8,24) R(9,0) R(10,0) R(11,27) R(12,31) R(13,14) R(13,15) R(13,n4) R(14,13) R(15,13) R(15,n7) R(16,n5) R(17,0) R(17,2) R(18,0) R(18,19) R(19,3) R(19,18) R(19,22) R(20,3) R(20,21) R(21,4) R(21,20) R(22,19) R(22,23) R(23,5) R(23,22) R(24,0) R(24,8) R(25,0) R(25,26) R(26,25) R(26,27) R(27,11) R(27,26) R(28,0) R(28,29) R(29,28) R(29,30) R(30,29) R(30,31) R(31,12) R(31,30) R(n1,0) R(n1,n2) R(n1,n3) R(n2,6) R(n2,7) R(n2,n1) R(n3,n1) R(n3,n6) R(n4,13) R(n4,n6) R(n5,16) R(n5,n7) R(n6,n3) R(n6,n4) R(n7,15) R(n7,n5)

-------------------------------------------------------------------------------------------------------------

-> form outgroup SOL1 - B

n2(mod99)

[ cox1 cox2 atp8 atp6 cox3 nad3 -nad5 -nad4 -nad4L nad6 cob rrnS rrnL nad1 nad2 ]

n1(mod99)15g

[ cox1 cox2 atp8 atp6 cox3 nad3 -nad5 -nad4 -nad4L -cob -nad6 -nad1 -rrnL -rrnS nad2 ]

n1(mod99)13g

[ cox1 cox2 cox3 nad3 -nad5 -nad4 -nad4L -cob -nad6 -nad1 -rrnL -rrnS nad2 ]

n4(mod99)

g1: [ cox1 cox2 nad1 nad3 -nad4 -nad5 -nad4L -cob -nad6 nad2 cox3 rrnS rrnL ]

g2: [ cox1 cox2 nad1 nad3 nad2 cox3 rrnS -nad5 -nad4 -nad4L -cob -nad6 rrnL ]

g3: [ cox1 cox2 nad1 nad6 cob nad4L nad5 nad4 nad3 nad2 cox3 rrnS rrnL ]

score = 276:

model 99:

-------------

R(0,1) R(0,9) R(0,10) R(0,17) R(0,18) R(0,24) R(0,25) R(0,28) R(0,n1) R(1,0) R(2,17) R(3,19) R(3,20) R(4,21) R(5,23) R(6,n2) R(7,n2) R(8,24) R(9,0) R(10,0) R(11,27) R(12,31) R(13,14) R(13,15) R(13,n4) R(14,13) R(15,13) R(16,n5) R(17,0) R(17,2) R(18,0) R(18,19) R(19,3) R(19,18) R(19,22) R(20,3) R(20,21) R(21,4) R(21,20) R(22,19) R(22,23) R(23,5) R(23,22) R(24,0) R(24,8) R(25,0) R(25,26) R(26,25) R(26,27) R(27,11) R(27,26) R(28,0) R(28,29) R(29,28) R(29,30) R(30,29) R(30,31) R(31,12) R(31,30) R(n1,0) R(n1,n2) R(n1,n3) R(n2,6) R(n2,7) R(n2,n1) R(n3,n1) R(n3,n6) R(n4,13) R(n4,n6) R(n4,n7) R(n5,16) R(n5,n7) R(n6,n3) R(n6,n4) R(n7,n4) R(n7,n5)

-------------------------------------------------------------------------------------------------------------

-> form outgroup SOL1 - B

n3(mod100)

[ cox1 cox2 atp8 atp6 cox3 nad3 -nad5 -nad4 -nad4L nad6 cob rrnS rrnL nad1 nad2 ]

n1(mod100)15g

[ cox1 cox2 atp8 atp6 cox3 nad3 -nad5 -nad4 -nad4L -cob -nad6 -nad1 -rrnL -rrnS nad2 ]

n1(mod100)13g

[ cox1 cox2 cox3 nad3 -nad5 -nad4 -nad4L -cob -nad6 -nad1 -rrnL -rrnS nad2 ]

n6(mod100)

[ cox1 cox2 nad1 nad3 nad2 cox3 rrnS -nad5 -nad4 -nad4L -cob -nad6 rrnL ]

n4(mod100)

[ cox1 cox2 nad1 nad3 nad2 cox3 rrnS nad5 nad4 -nad4L -cob -nad6 rrnL ]

score = 290:

model 100:

-------------

R(0,1) R(0,9) R(0,10) R(0,17) R(0,18) R(0,24) R(0,25) R(0,28) R(0,n1) R(1,0) R(2,17) R(3,19) R(3,20) R(4,21) R(5,23) R(6,n3) R(7,n3) R(8,24) R(9,0) R(10,0) R(11,27) R(12,31) R(13,14) R(13,n4) R(14,13) R(15,n6) R(16,n5) R(17,0) R(17,2) R(18,0) R(18,19) R(19,3) R(19,18) R(19,22) R(20,3) R(20,21) R(21,4) R(21,20) R(22,19) R(22,23) R(23,5) R(23,22) R(24,0) R(24,8) R(25,0) R(25,26) R(26,25) R(26,27) R(27,11) R(27,26) R(28,0) R(28,29) R(29,28) R(29,30) R(30,29) R(30,31) R(31,12) R(31,30) R(n1,0) R(n1,n2) R(n1,n3) R(n2,n1) R(n2,n7) R(n3,6) R(n3,7) R(n3,n1) R(n4,13) R(n4,n5) R(n4,n6) R(n5,16) R(n5,n4) R(n6,15) R(n6,n4) R(n6,n7) R(n7,n2) R(n7,n6)

-------------------------------------------------------------------------------------------------------------

-> form outgroup SOL3 - B

n1(mod101)15g

[ cox1 cox2 atp8 atp6 cox3 nad3 -nad5 -nad4 -nad4L -cob -nad6 -nad1 -rrnL -rrnS nad2 ]

n1(mod101)13g

[ cox1 cox2 cox3 nad3 -nad5 -nad4 -nad4L -cob -nad6 -nad1 -rrnL -rrnS nad2 ]

score = 286:

model 101:

-------------

R(0,1) R(0,9) R(0,10) R(0,17) R(0,18) R(0,24) R(0,25) R(0,28) R(0,n1) R(1,0) R(2,17) R(3,19) R(3,20) R(4,21) R(5,23) R(6,n1) R(7,n3) R(8,24) R(9,0) R(10,0) R(11,27) R(12,31) R(13,14) R(13,15) R(14,13) R(15,13) R(15,n4) R(15,n5) R(16,n6) R(17,0) R(17,2) R(18,0) R(18,19) R(19,3) R(19,18) R(19,22) R(20,3) R(20,21) R(21,4) R(21,20) R(22,19) R(22,23) R(23,5) R(23,22) R(24,0) R(24,8) R(25,0) R(25,26) R(26,25) R(26,27) R(27,11) R(27,26) R(28,0) R(28,29) R(29,28) R(29,30) R(30,29) R(30,31) R(31,12) R(31,30) R(n1,0) R(n1,6) R(n1,n2) R(n1,n3) R(n2,n1) R(n2,n7) R(n3,7) R(n3,n1) R(n4,15) R(n4,n6) R(n5,15) R(n5,n7) R(n6,16) R(n6,n4) R(n7,n2) R(n7,n5)

-------------------------------------------------------------------------------------------------------------

-> form outgroup SOL1 - B

n3(mod102)

[ cox1 cox2 atp8 atp6 cox3 nad3 -nad5 -nad4 -nad4L nad6 cob rrnS rrnL nad1 nad2 ]

n1(mod102)15g

[ cox1 cox2 atp8 atp6 cox3 nad3 -nad5 -nad4 -nad4L -cob -nad6 -nad1 -rrnL -rrnS nad2 ]

n1(mod102)13g

[ cox1 cox2 cox3 nad3 -nad5 -nad4 -nad4L -cob -nad6 -nad1 -rrnL -rrnS nad2 ]

score = 300:

model 102:

-------------

R(0,1) R(0,9) R(0,10) R(0,17) R(0,18) R(0,24) R(0,25) R(0,28) R(0,n1) R(1,0) R(2,17) R(3,19) R(3,20) R(4,21) R(5,23) R(6,n3) R(7,n3) R(8,24) R(9,0) R(10,0) R(11,27) R(12,31) R(13,14) R(13,15) R(14,13) R(15,13) R(15,n4) R(15,n5) R(16,n6) R(17,0) R(17,2) R(18,0) R(18,19) R(19,3) R(19,18) R(19,22) R(20,3) R(20,21) R(21,4) R(21,20) R(22,19) R(22,23) R(23,5) R(23,22) R(24,0) R(24,8) R(25,0) R(25,26) R(26,25) R(26,27) R(27,11) R(27,26) R(28,0) R(28,29) R(29,28) R(29,30) R(30,29) R(30,31) R(31,12) R(31,30) R(n1,0) R(n1,n2) R(n1,n3) R(n2,n1) R(n2,n7) R(n3,6) R(n3,7) R(n3,n1) R(n4,15) R(n4,n6) R(n5,15) R(n5,n7) R(n6,16) R(n6,n4) R(n7,n2) R(n7,n5)

-------------------------------------------------------------------------------------------------------------

-> form outgroup SOL3 - B

n1(mod103)15g

[ cox1 cox2 atp8 atp6 cox3 nad3 -nad5 -nad4 -nad4L -cob -nad6 -nad1 -rrnL -rrnS nad2 ]

n1(mod103)13g

[ cox1 cox2 cox3 nad3 -nad5 -nad4 -nad4L -cob -nad6 -nad1 -rrnL -rrnS nad2 ]

n4(mod103)

[ cox1 cox2 nad1 nad3 nad2 cox3 rrnS -nad5 -nad4 -nad4L -cob -nad6 rrnL ]

score = 276:

model 103:

-------------

R(0,1) R(0,9) R(0,10) R(0,17) R(0,18) R(0,24) R(0,25) R(0,28) R(0,n1) R(1,0) R(2,17) R(3,19) R(3,20) R(4,21) R(5,23) R(6,n1) R(7,n2) R(8,24) R(9,0) R(10,0) R(11,27) R(12,31) R(13,14) R(13,15) R(14,13) R(15,13) R(15,n4) R(16,n5) R(17,0) R(17,2) R(18,0) R(18,19) R(19,3) R(19,18) R(19,22) R(20,3) R(20,21) R(21,4) R(21,20) R(22,19) R(22,23) R(23,5) R(23,22) R(24,0) R(24,8) R(25,0) R(25,26) R(26,25) R(26,27) R(27,11) R(27,26) R(28,0) R(28,29) R(29,28) R(29,30) R(30,29) R(30,31) R(31,12) R(31,30) R(n1,0) R(n1,6) R(n1,n2) R(n1,n3) R(n2,7) R(n2,n1) R(n3,n1) R(n3,n6) R(n4,15) R(n4,n6) R(n4,n7) R(n5,16) R(n5,n7) R(n6,n3) R(n6,n4) R(n7,n4) R(n7,n5)

-------------------------------------------------------------------------------------------------------------

-> form outgroup SOL1 - B

n2(mod104)

[ cox1 cox2 atp8 atp6 cox3 nad3 -nad5 -nad4 -nad4L nad6 cob rrnS rrnL nad1 nad2 ]

n1(mod104)15g

[ cox1 cox2 atp8 atp6 cox3 nad3 -nad5 -nad4 -nad4L -cob -nad6 -nad1 -rrnL -rrnS nad2 ]

n1(mod104)13g

[ cox1 cox2 cox3 nad3 -nad5 -nad4 -nad4L -cob -nad6 -nad1 -rrnL -rrnS nad2 ]

n4(mod104)

[ cox1 cox2 nad1 nad3 nad2 cox3 rrnS -nad5 -nad4 -nad4L -cob -nad6 rrnL ]

score = 290:

model 104:

-------------

R(0,1) R(0,9) R(0,10) R(0,17) R(0,18) R(0,24) R(0,25) R(0,28) R(0,n1) R(1,0) R(2,17) R(3,19) R(3,20) R(4,21) R(5,23) R(6,n2) R(7,n2) R(8,24) R(9,0) R(10,0) R(11,27) R(12,31) R(13,14) R(13,15) R(14,13) R(15,13) R(15,n4) R(16,n5) R(17,0) R(17,2) R(18,0) R(18,19) R(19,3) R(19,18) R(19,22) R(20,3) R(20,21) R(21,4) R(21,20) R(22,19) R(22,23) R(23,5) R(23,22) R(24,0) R(24,8) R(25,0) R(25,26) R(26,25) R(26,27) R(27,11) R(27,26) R(28,0) R(28,29) R(29,28) R(29,30) R(30,29) R(30,31) R(31,12) R(31,30) R(n1,0) R(n1,n2) R(n1,n3) R(n2,6) R(n2,7) R(n2,n1) R(n3,n1) R(n3,n6) R(n4,15) R(n4,n6) R(n4,n7) R(n5,16) R(n5,n7) R(n6,n3) R(n6,n4) R(n7,n4) R(n7,n5)

-------------------------------------------------------------------------------------------------------------

-> form outgroup SOL9 - A

n1(mod105)15g

[ cox1 cox2 atp8 atp6 cox3 nad3 -nad5 -nad4 -nad4L -cob -nad6 -nad1 -rrnL -rrnS nad2 ]

n2(mod105)15g

[ cox1 cox2 atp8 atp6 cox3 nad3 -nad1 -rrnL nad6 cob nad4L nad4 nad5 -rrnS nad2 ]

n2(mod105)13g

[ cox1 cox2 cox3 nad3 -nad1 -rrnL nad6 cob nad4L nad4 nad5 -rrnS nad2 ]

score = 294:

model 105:

-------------

R(0,1) R(0,9) R(0,10) R(0,17) R(0,18) R(0,24) R(0,25) R(0,28) R(0,n1) R(1,0) R(2,17) R(3,19) R(3,20) R(4,21) R(5,23) R(6,n1) R(7,n4) R(8,24) R(9,0) R(10,0) R(11,27) R(12,31) R(13,14) R(13,15) R(14,13) R(15,13) R(15,n6) R(15,n7) R(16,n5) R(17,0) R(17,2) R(18,0) R(18,19) R(19,3) R(19,18) R(19,22) R(20,3) R(20,21) R(21,4) R(21,20) R(22,19) R(22,23) R(23,5) R(23,22) R(24,0) R(24,8) R(25,0) R(25,26) R(26,25) R(26,27) R(27,11) R(27,26) R(28,0) R(28,29) R(29,28) R(29,30) R(30,29) R(30,31) R(31,12) R(31,30) R(n1,0) R(n1,6) R(n1,n2) R(n2,n1) R(n2,n3) R(n2,n4) R(n3,n2) R(n3,n7) R(n4,7) R(n4,n2) R(n5,16) R(n5,n6) R(n6,15) R(n6,n5) R(n7,15) R(n7,n3)

-------------------------------------------------------------------------------------------------------------

-> form outgroup SOL9 - A

n1(mod106)15g

[ cox1 cox2 atp8 atp6 cox3 nad3 -nad5 -nad4 -nad4L -cob -nad6 -nad1 -rrnL -rrnS nad2 ]

n2(mod106)15g

[ cox1 cox2 atp8 atp6 cox3 nad3 -nad1 -rrnL nad6 cob nad4L nad4 nad5 -rrnS nad2 ]

n2(mod106)13g

[ cox1 cox2 cox3 nad3 -nad1 -rrnL nad6 cob nad4L nad4 nad5 -rrnS nad2 ]

n6(mod106)

[ cox1 cox2 nad1 nad3 nad2 cox3 rrnS -nad5 -nad4 -nad4L -cob -nad6 rrnL ]

score = 284:

model 106:

-------------

R(0,1) R(0,9) R(0,10) R(0,17) R(0,18) R(0,24) R(0,25) R(0,28) R(0,n1) R(1,0) R(2,17) R(3,19) R(3,20) R(4,21) R(5,23) R(6,n1) R(7,n4) R(8,24) R(9,0) R(10,0) R(11,27) R(12,31) R(13,14) R(13,15) R(14,13) R(15,13) R(15,n6) R(16,n5) R(17,0) R(17,2) R(18,0) R(18,19) R(19,3) R(19,18) R(19,22) R(20,3) R(20,21) R(21,4) R(21,20) R(22,19) R(22,23) R(23,5) R(23,22) R(24,0) R(24,8) R(25,0) R(25,26) R(26,25) R(26,27) R(27,11) R(27,26) R(28,0) R(28,29) R(29,28) R(29,30) R(30,29) R(30,31) R(31,12) R(31,30) R(n1,0) R(n1,6) R(n1,n2) R(n2,n1) R(n2,n3) R(n2,n4) R(n3,n2) R(n3,n6) R(n4,7) R(n4,n2) R(n5,16) R(n5,n7) R(n6,15) R(n6,n3) R(n6,n7) R(n7,n5) R(n7,n6)

-------------------------------------------------------------------------------------------------------------

-> form outgroup SOL9 - A

n1(mod107)

[ cox1 cox2 atp8 atp6 cox3 nad3 -nad5 -nad4 -nad4L -cob -nad6 -nad1 -rrnL -rrnS nad2 ]

n2(mod107)15g

[ cox1 cox2 atp8 atp6 cox3 nad3 -nad1 -rrnL nad6 cob nad4L nad4 nad5 -rrnS nad2 ]

n2(mod107)13g

[ cox1 cox2 cox3 nad3 -nad1 -rrnL nad6 cob nad4L nad4 nad5 -rrnS nad2 ]

score = 294:

model 107:

-------------

R(0,1) R(0,9) R(0,10) R(0,17) R(0,18) R(0,24) R(0,25) R(0,28) R(0,n1) R(1,0) R(2,17) R(3,19) R(3,20) R(4,21) R(5,23) R(6,n1) R(7,n3) R(8,24) R(9,0) R(10,0) R(11,27) R(12,31) R(13,14) R(13,15) R(13,n7) R(14,13) R(15,13) R(15,n6) R(16,n5) R(17,0) R(17,2) R(18,0) R(18,19) R(19,3) R(19,18) R(19,22) R(20,3) R(20,21) R(21,4) R(21,20) R(22,19) R(22,23) R(23,5) R(23,22) R(24,0) R(24,8) R(25,0) R(25,26) R(26,25) R(26,27) R(27,11) R(27,26) R(28,0) R(28,29) R(29,28) R(29,30) R(30,29) R(30,31) R(31,12) R(31,30) R(n1,0) R(n1,6) R(n1,n2) R(n2,n1) R(n2,n3) R(n2,n4) R(n3,7) R(n3,n2) R(n4,n2) R(n4,n7) R(n5,16) R(n5,n6) R(n6,15) R(n6,n5) R(n7,13) R(n7,n4)

-------------------------------------------------------------------------------------------------------------

-> form outgroup SOL9 - A

n1(mod108)15g

[ cox1 cox2 atp8 atp6 cox3 nad3 -nad5 -nad4 -nad4L -cob -nad6 -nad1 -rrnL -rrnS nad2 ]

n2(mod108)15g

[ cox1 cox2 atp8 atp6 cox3 nad3 -nad1 -rrnL nad6 cob nad4L nad4 nad5 -rrnS nad2 ]

n2(mod108)13g

[ cox1 cox2 cox3 nad3 -nad1 -rrnL nad6 cob nad4L nad4 nad5 -rrnS nad2 ]

score = 306:

model 108:

-------------

R(0,1) R(0,9) R(0,10) R(0,17) R(0,18) R(0,24) R(0,25) R(0,28) R(0,n1) R(1,0) R(2,17) R(3,19) R(3,20) R(4,21) R(5,23) R(6,n1) R(7,n4) R(8,24) R(9,0) R(10,0) R(11,27) R(12,31) R(13,14) R(13,15) R(13,n7) R(14,13) R(15,13) R(15,n6) R(16,n5) R(17,0) R(17,2) R(18,0) R(18,19) R(19,3) R(19,18) R(19,22) R(20,3) R(20,21) R(21,4) R(21,20) R(22,19) R(22,23) R(23,5) R(23,22) R(24,0) R(24,8) R(25,0) R(25,26) R(26,25) R(26,27) R(27,11) R(27,26) R(28,0) R(28,29) R(29,28) R(29,30) R(30,29) R(30,31) R(31,12) R(31,30) R(n1,0) R(n1,6) R(n1,n2) R(n2,n1) R(n2,n3) R(n2,n4) R(n3,n2) R(n3,n6) R(n4,7) R(n4,n2) R(n5,16) R(n5,n7) R(n6,15) R(n6,n3) R(n7,13) R(n7,n5)

-------------------------------------------------------------------------------------------------------------

-> form outgroup SOL9 - A

n1(mod109)

[ cox1 cox2 atp8 atp6 cox3 nad3 -nad5 -nad4 -nad4L -cob -nad6 -nad1 -rrnL -rrnS nad2 ]

n2(mod109)15g

[ cox1 cox2 atp8 atp6 cox3 nad3 -nad1 -rrnL nad6 cob nad4L nad4 nad5 -rrnS nad2 ]

n2(mod109)13g

[ cox1 cox2 cox3 nad3 -nad1 -rrnL nad6 cob nad4L nad4 nad5 -rrnS nad2 ]

n6(mod109)

[ cox1 cox2 nad1 nad3 nad2 cox3 rrnS nad5 nad4 -nad4L -cob -nad6 rrnL ]

score = 306:

model 109:

-------------

R(0,1) R(0,9) R(0,10) R(0,17) R(0,18) R(0,24) R(0,25) R(0,28) R(0,n1) R(1,0) R(2,17) R(3,19) R(3,20) R(4,21) R(5,23) R(6,n1) R(7,n4) R(8,24) R(9,0) R(10,0) R(11,27) R(12,31) R(13,14) R(13,15) R(13,n6) R(14,13) R(15,13) R(16,n5) R(17,0) R(17,2) R(18,0) R(18,19) R(19,3) R(19,18) R(19,22) R(20,3) R(20,21) R(21,4) R(21,20) R(22,19) R(22,23) R(23,5) R(23,22) R(24,0) R(24,8) R(25,0) R(25,26) R(26,25) R(26,27) R(27,11) R(27,26) R(28,0) R(28,29) R(29,28) R(29,30) R(30,29) R(30,31) R(31,12) R(31,30) R(n1,0) R(n1,6) R(n1,n2) R(n2,n1) R(n2,n3) R(n2,n4) R(n3,n2) R(n3,n7) R(n4,7) R(n4,n2) R(n5,16) R(n5,n6) R(n6,13) R(n6,n5) R(n6,n7) R(n7,n3) R(n7,n6)

-------------------------------------------------------------------------------------------------------------

-> form outgroup SOL9 - A

n1(mod110)

[ cox1 cox2 atp8 atp6 cox3 nad3 -nad5 -nad4 -nad4L -cob -nad6 -nad1 -rrnL -rrnS nad2 ]

n2(mod110)15g

[ cox1 cox2 atp8 atp6 cox3 nad3 -nad1 -rrnL nad6 cob nad4L nad4 nad5 -rrnS nad2 ]

n2(mod110)13g

[ cox1 cox2 cox3 nad3 -nad1 -rrnL nad6 cob nad4L nad4 nad5 -rrnS nad2 ]

n7(mod110)

[ cox1 cox2 nad1 nad3 nad2 cox3 rrnS -nad5 -nad4 -nad4L -cob -nad6 rrnL ]

score = 270:

model 110:

-------------

R(0,1) R(0,9) R(0,10) R(0,17) R(0,18) R(0,24) R(0,25) R(0,28) R(0,n1) R(1,0) R(2,17) R(3,19) R(3,20) R(4,21) R(5,23) R(6,n1) R(7,n4) R(8,24) R(9,0) R(10,0) R(11,27) R(12,31) R(13,14) R(13,15) R(13,n7) R(14,13) R(15,13) R(16,n5) R(17,0) R(17,2) R(18,0) R(18,19) R(19,3) R(19,18) R(19,22) R(20,3) R(20,21) R(21,4) R(21,20) R(22,19) R(22,23) R(23,5) R(23,22) R(24,0) R(24,8) R(25,0) R(25,26) R(26,25) R(26,27) R(27,11) R(27,26) R(28,0) R(28,29) R(29,28) R(29,30) R(30,29) R(30,31) R(31,12) R(31,30) R(n1,0) R(n1,6) R(n1,n2) R(n2,n1) R(n2,n3) R(n2,n4) R(n3,n2) R(n3,n7) R(n4,7) R(n4,n2) R(n5,16) R(n5,n6) R(n6,n5) R(n6,n7) R(n7,13) R(n7,n3) R(n7,n6)

-------------------------------------------------------------------------------------------------------------

-> form outgroup SOL9 - A

n1(mod111)

[ cox1 cox2 atp8 atp6 cox3 nad3 -nad5 -nad4 -nad4L -cob -nad6 -nad1 -rrnL -rrnS nad2 ]

n2(mod111)15g

[ cox1 cox2 atp8 atp6 cox3 nad3 -nad1 -rrnL nad6 cob nad4L nad4 nad5 -rrnS nad2 ]

n2(mod111)13g

[ cox1 cox2 cox3 nad3 -nad1 -rrnL nad6 cob nad4L nad4 nad5 -rrnS nad2 ]

score = 280:

model 111:

-------------

R(0,1) R(0,9) R(0,10) R(0,17) R(0,18) R(0,24) R(0,25) R(0,28) R(0,n1) R(1,0) R(2,17) R(3,19) R(3,20) R(4,21) R(5,23) R(6,n1) R(7,n3) R(8,24) R(9,0) R(10,0) R(11,27) R(12,31) R(13,14) R(13,15) R(13,n6) R(13,n7) R(14,13) R(15,13) R(16,n5) R(17,0) R(17,2) R(18,0) R(18,19) R(19,3) R(19,18) R(19,22) R(20,3) R(20,21) R(21,4) R(21,20) R(22,19) R(22,23) R(23,5) R(23,22) R(24,0) R(24,8) R(25,0) R(25,26) R(26,25) R(26,27) R(27,11) R(27,26) R(28,0) R(28,29) R(29,28) R(29,30) R(30,29) R(30,31) R(31,12) R(31,30) R(n1,0) R(n1,6) R(n1,n2) R(n2,n1) R(n2,n3) R(n2,n4) R(n3,7) R(n3,n2) R(n4,n2) R(n4,n7) R(n5,16) R(n5,n6) R(n6,13) R(n6,n5) R(n7,13) R(n7,n4)

-------------------------------------------------------------------------------------------------------------

-> form outgroup SOL9 - A

n1(mod112)15g

[ cox1 cox2 atp8 atp6 cox3 nad3 -nad5 -nad4 -nad4L -cob -nad6 -nad1 -rrnL -rrnS nad2 ]

n2(mod112)15g

[ cox1 cox2 atp8 atp6 cox3 nad3 -nad1 -rrnL nad6 cob nad4L nad4 nad5 -rrnS nad2 ]

n2(mod112)13g

[ cox1 cox2 cox3 nad3 -nad1 -rrnL nad6 cob nad4L nad4 nad5 -rrnS nad2 ]

n6(mod112)

[ cox1 cox2 nad1 nad3 nad2 cox3 rrnS -nad4L -nad4 -nad5 -cob -nad6 rrnL ]

score = 320:

model 112:

-------------

R(0,1) R(0,9) R(0,10) R(0,17) R(0,18) R(0,24) R(0,25) R(0,28) R(0,n1) R(1,0) R(2,17) R(3,19) R(3,20) R(4,21) R(5,23) R(6,n1) R(7,n4) R(8,24) R(9,0) R(10,0) R(11,27) R(12,31) R(13,14) R(13,n6) R(14,13) R(15,n6) R(15,n7) R(16,n5) R(17,0) R(17,2) R(18,0) R(18,19) R(19,3) R(19,18) R(19,22) R(20,3) R(20,21) R(21,4) R(21,20) R(22,19) R(22,23) R(23,5) R(23,22) R(24,0) R(24,8) R(25,0) R(25,26) R(26,25) R(26,27) R(27,11) R(27,26) R(28,0) R(28,29) R(29,28) R(29,30) R(30,29) R(30,31) R(31,12) R(31,30) R(n1,0) R(n1,6) R(n1,n2) R(n2,n1) R(n2,n3) R(n2,n4) R(n3,n2) R(n3,n7) R(n4,7) R(n4,n2) R(n5,16) R(n5,n6) R(n6,13) R(n6,15) R(n6,n5) R(n7,15) R(n7,n3)

-------------------------------------------------------------------------------------------------------------

-> form outgroup SOL9 - A

n1(mod113)15g

[ cox1 cox2 atp8 atp6 cox3 nad3 -nad5 -nad4 -nad4L -cob -nad6 -nad1 -rrnL -rrnS nad2 ]

n2(mod113)15g

[ cox1 cox2 atp8 atp6 cox3 nad3 -nad1 -rrnL nad6 cob nad4L nad4 nad5 -rrnS nad2 ]

n2(mod113)13g

[ cox1 cox2 cox3 nad3 -nad1 -rrnL nad6 cob nad4L nad4 nad5 -rrnS nad2 ]

n7(mod113)

[ cox1 cox2 nad1 nad3 nad2 cox3 rrnS -nad5 -nad4 -nad4L -cob -nad6 rrnL ]

score = 272:

model 113:

-------------

R(0,1) R(0,9) R(0,10) R(0,17) R(0,18) R(0,24) R(0,25) R(0,28) R(0,n1) R(1,0) R(2,17) R(3,19) R(3,20) R(4,21) R(5,23) R(6,n1) R(7,n3) R(8,24) R(9,0) R(10,0) R(11,27) R(12,31) R(13,14) R(13,n7) R(14,13) R(15,n6) R(15,n7) R(16,n5) R(17,0) R(17,2) R(18,0) R(18,19) R(19,3) R(19,18) R(19,22) R(20,3) R(20,21) R(21,4) R(21,20) R(22,19) R(22,23) R(23,5) R(23,22) R(24,0) R(24,8) R(25,0) R(25,26) R(26,25) R(26,27) R(27,11) R(27,26) R(28,0) R(28,29) R(29,28) R(29,30) R(30,29) R(30,31) R(31,12) R(31,30) R(n1,0) R(n1,6) R(n1,n2) R(n2,n1) R(n2,n3) R(n2,n4) R(n3,7) R(n3,n2) R(n4,n2) R(n4,n7) R(n5,16) R(n5,n6) R(n6,15) R(n6,n5) R(n7,13) R(n7,15) R(n7,n4)

-------------------------------------------------------------------------------------------------------------

-> form outgroup SOL9 - A

n1(mod114)15g

[ cox1 cox2 atp8 atp6 cox3 nad3 -nad5 -nad4 -nad4L -cob -nad6 -nad1 -rrnL -rrnS nad2 ]

n2(mod114)15g

[ cox1 cox2 atp8 atp6 cox3 nad3 -nad1 -rrnL nad6 cob nad4L nad4 nad5 -rrnS nad2 ]

n2(mod114)13g

[ cox1 cox2 cox3 nad3 -nad1 -rrnL nad6 cob nad4L nad4 nad5 -rrnS nad2 ]

n6(mod114)

[ cox1 cox2 nad1 nad3 nad2 cox3 rrnS -nad5 -nad4 -nad4L -cob -nad6 rrnL ]

score = 258:

model 114:

-------------

R(0,1) R(0,9) R(0,10) R(0,17) R(0,18) R(0,24) R(0,25) R(0,28) R(0,n1) R(1,0) R(2,17) R(3,19) R(3,20) R(4,21) R(5,23) R(6,n1) R(7,n4) R(8,24) R(9,0) R(10,0) R(11,27) R(12,31) R(13,14) R(13,n6) R(14,13) R(15,n6) R(16,n5) R(17,0) R(17,2) R(18,0) R(18,19) R(19,3) R(19,18) R(19,22) R(20,3) R(20,21) R(21,4) R(21,20) R(22,19) R(22,23) R(23,5) R(23,22) R(24,0) R(24,8) R(25,0) R(25,26) R(26,25) R(26,27) R(27,11) R(27,26) R(28,0) R(28,29) R(29,28) R(29,30) R(30,29) R(30,31) R(31,12) R(31,30) R(n1,0) R(n1,6) R(n1,n2) R(n2,n1) R(n2,n3) R(n2,n4) R(n3,n2) R(n3,n6) R(n4,7) R(n4,n2) R(n5,16) R(n5,n7) R(n6,13) R(n6,15) R(n6,n3) R(n6,n7) R(n7,n5) R(n7,n6)

-------------------------------------------------------------------------------------------------------------

-> form outgroup SOL9 - A

n1(mod115)15g

[ cox1 cox2 atp8 atp6 cox3 nad3 -nad5 -nad4 -nad4L -cob -nad6 -nad1 -rrnL -rrnS nad2 ]

n2(mod115)15g

[ cox1 cox2 atp8 atp6 cox3 nad3 -nad1 -rrnL nad6 cob nad4L nad4 nad5 -rrnS nad2 ]

n2(mod115)13g

[ cox1 cox2 cox3 nad3 -nad1 -rrnL nad6 cob nad4L nad4 nad5 -rrnS nad2 ]

n6(mod115)

[ cox1 cox2 nad1 nad3 nad2 cox3 rrnS -nad5 -nad4 -nad4L -cob -nad6 rrnL ]

n7(mod115)

[ cox1 cox2 nad1 nad3 nad2 cox3 rrnS nad5 nad4 -nad4L -cob -nad6 rrnL ]

score = 284:

model 115:

-------------

R(0,1) R(0,9) R(0,10) R(0,17) R(0,18) R(0,24) R(0,25) R(0,28) R(0,n1) R(1,0) R(2,17) R(3,19) R(3,20) R(4,21) R(5,23) R(6,n1) R(7,n4) R(8,24) R(9,0) R(10,0) R(11,27) R(12,31) R(13,14) R(13,n7) R(14,13) R(15,n6) R(16,n5) R(17,0) R(17,2) R(18,0) R(18,19) R(19,3) R(19,18) R(19,22) R(20,3) R(20,21) R(21,4) R(21,20) R(22,19) R(22,23) R(23,5) R(23,22) R(24,0) R(24,8) R(25,0) R(25,26) R(26,25) R(26,27) R(27,11) R(27,26) R(28,0) R(28,29) R(29,28) R(29,30) R(30,29) R(30,31) R(31,12) R(31,30) R(n1,0) R(n1,6) R(n1,n2) R(n2,n1) R(n2,n3) R(n2,n4) R(n3,n2) R(n3,n6) R(n4,7) R(n4,n2) R(n5,16) R(n5,n7) R(n6,15) R(n6,n3) R(n6,n7) R(n7,13) R(n7,n5) R(n7,n6)

-------------------------------------------------------------------------------------------------------------

-> form outgroup SOL9 - A

n1(mod116)

[ cox1 cox2 atp8 atp6 cox3 nad3 -nad5 -nad4 -nad4L -cob -nad6 -nad1 -rrnL -rrnS nad2 ]

n2(mod116)15g

[ cox1 cox2 atp8 atp6 cox3 nad3 -nad1 -rrnL nad6 cob nad4L nad4 nad5 -rrnS nad2 ]

n2(mod116)13g

[ cox1 cox2 cox3 nad3 -nad1 -rrnL nad6 cob nad4L nad4 nad5 -rrnS nad2 ]

n6(mod116)

[ cox1 cox2 nad1 nad3 nad2 cox3 rrnS -nad4L -nad4 -nad5 -cob -nad6 rrnL ]

score = 294:

model 116:

-------------

R(0,1) R(0,9) R(0,10) R(0,17) R(0,18) R(0,24) R(0,25) R(0,28) R(0,n1) R(1,0) R(2,17) R(3,19) R(3,20) R(4,21) R(5,23) R(6,n1) R(7,n3) R(8,24) R(9,0) R(10,0) R(11,27) R(12,31) R(13,14) R(13,n6) R(13,n7) R(14,13) R(15,n6) R(16,n5) R(17,0) R(17,2) R(18,0) R(18,19) R(19,3) R(19,18) R(19,22) R(20,3) R(20,21) R(21,4) R(21,20) R(22,19) R(22,23) R(23,5) R(23,22) R(24,0) R(24,8) R(25,0) R(25,26) R(26,25) R(26,27) R(27,11) R(27,26) R(28,0) R(28,29) R(29,28) R(29,30) R(30,29) R(30,31) R(31,12) R(31,30) R(n1,0) R(n1,6) R(n1,n2) R(n2,n1) R(n2,n3) R(n2,n4) R(n3,7) R(n3,n2) R(n4,n2) R(n4,n7) R(n5,16) R(n5,n6) R(n6,13) R(n6,15) R(n6,n5) R(n7,13) R(n7,n4)

-------------------------------------------------------------------------------------------------------------

-> form outgroup SOL9 - A

n1(mod117)15g

[ cox1 cox2 atp8 atp6 cox3 nad3 -nad5 -nad4 -nad4L -cob -nad6 -nad1 -rrnL -rrnS nad2 ]

n2(mod117)15g

[ cox1 cox2 atp8 atp6 cox3 nad3 -nad1 -rrnL nad6 cob nad4L nad4 nad5 -rrnS nad2 ]

n2(mod117)13g

[ cox1 cox2 cox3 nad3 -nad1 -rrnL nad6 cob nad4L nad4 nad5 -rrnS nad2 ]

n6(mod117)

[ cox1 cox2 nad1 nad3 nad2 cox3 rrnS -nad5 -nad4 -nad4L -cob -nad6 rrnL ]

score = 270:

model 117:

-------------

R(0,1) R(0,9) R(0,10) R(0,17) R(0,18) R(0,24) R(0,25) R(0,28) R(0,n1) R(1,0) R(2,17) R(3,19) R(3,20) R(4,21) R(5,23) R(6,n1) R(7,n4) R(8,24) R(9,0) R(10,0) R(11,27) R(12,31) R(13,14) R(13,n6) R(13,n7) R(14,13) R(15,n6) R(16,n5) R(17,0) R(17,2) R(18,0) R(18,19) R(19,3) R(19,18) R(19,22) R(20,3) R(20,21) R(21,4) R(21,20) R(22,19) R(22,23) R(23,5) R(23,22) R(24,0) R(24,8) R(25,0) R(25,26) R(26,25) R(26,27) R(27,11) R(27,26) R(28,0) R(28,29) R(29,28) R(29,30) R(30,29) R(30,31) R(31,12) R(31,30) R(n1,0) R(n1,6) R(n1,n2) R(n2,n1) R(n2,n3) R(n2,n4) R(n3,n2) R(n3,n6) R(n4,7) R(n4,n2) R(n5,16) R(n5,n7) R(n6,13) R(n6,15) R(n6,n3) R(n7,13) R(n7,n5)

-------------------------------------------------------------------------------------------------------------

-> form outgroup SOL4 - B

n4(mod118)

[ cox1 cox2 atp8 atp6 cox3 nad3 -nad5 -nad4 -nad4L nad6 cob rrnS rrnL nad1 nad2 ]

n2(mod118)15g

[ cox1 cox2 atp8 atp6 cox3 -nad5 -nad4 -nad4L nad6 cob rrnS rrnL nad1 nad3 nad2 ]

n2(mod118)13g

[ cox1 cox2 cox3 -nad5 -nad4 -nad4L nad6 cob rrnS rrnL nad1 nad3 nad2 ]

n6(mod118)

[ cox1 cox2 nad1 nad3 nad2 cox3 rrnS -nad4 -nad5 -cob -nad6 nad4L rrnL ]

score = 326:

model 118:

-------------

R(0,1) R(0,9) R(0,10) R(0,17) R(0,18) R(0,24) R(0,25) R(0,28) R(0,n1) R(1,0) R(2,17) R(3,19) R(3,20) R(4,21) R(5,23) R(6,n4) R(7,n4) R(8,24) R(9,0) R(10,0) R(11,27) R(12,31) R(13,14) R(13,15) R(13,n6) R(14,13) R(15,13) R(16,n5) R(17,0) R(17,2) R(18,0) R(18,19) R(19,3) R(19,18) R(19,22) R(20,3) R(20,21) R(21,4) R(21,20) R(22,19) R(22,23) R(23,5) R(23,22) R(24,0) R(24,8) R(25,0) R(25,26) R(26,25) R(26,27) R(27,11) R(27,26) R(28,0) R(28,29) R(29,28) R(29,30) R(30,29) R(30,31) R(31,12) R(31,30) R(n1,0) R(n1,n2) R(n2,n1) R(n2,n3) R(n2,n4) R(n3,n2) R(n3,n7) R(n4,6) R(n4,7) R(n4,n2) R(n5,16) R(n5,n6) R(n6,13) R(n6,n5) R(n6,n7) R(n7,n3) R(n7,n6)

-------------------------------------------------------------------------------------------------------------

-> form outgroup SOL3 - D

n1(mod119)

[ cox1 cox2 atp8 atp6 cox3 nad3 -nad5 -nad4 -nad4L -cob -nad6 -nad1 -rrnL -rrnS nad2 ]

n2(mod119)15g

[ cox1 cox2 atp8 atp6 cox3 nad3 -nad5 -nad4 -nad4L nad6 cob rrnS rrnL nad1 nad2 ]

n2(mod119)13g

[ cox1 cox2 cox3 nad3 -nad5 -nad4 -nad4L nad6 cob rrnS rrnL nad1 nad2 ]

n7(mod119)

[ cox1 cox2 nad1 nad3 nad2 cox3 rrnS -nad4 -nad5 -cob -nad6 nad4L rrnL ]

score = 342:

model 119:

-------------

R(0,1) R(0,9) R(0,10) R(0,17) R(0,18) R(0,24) R(0,25) R(0,28) R(0,n1) R(1,0) R(2,17) R(3,19) R(3,20) R(4,21) R(5,23) R(6,n1) R(7,n2) R(8,24) R(9,0) R(10,0) R(11,27) R(12,31) R(13,14) R(13,15) R(13,n7) R(14,13) R(15,13) R(16,n5) R(17,0) R(17,2) R(18,0) R(18,19) R(19,3) R(19,18) R(19,22) R(20,3) R(20,21) R(21,4) R(21,20) R(22,19) R(22,23) R(23,5) R(23,22) R(24,0) R(24,8) R(25,0) R(25,26) R(26,25) R(26,27) R(27,11) R(27,26) R(28,0) R(28,29) R(29,28) R(29,30) R(30,29) R(30,31) R(31,12) R(31,30) R(n1,0) R(n1,6) R(n1,n2) R(n2,7) R(n2,n1) R(n2,n3) R(n3,n2) R(n3,n4) R(n4,n3) R(n4,n6) R(n5,16) R(n5,n7) R(n6,n4) R(n6,n7) R(n7,13) R(n7,n5) R(n7,n6)

-------------------------------------------------------------------------------------------------------------

-> form outgroup SOL2 - B

n1(mod120)15g

[ cox1 cox2 atp8 atp6 cox3 nad3 -nad5 -nad4 -nad4L -cob -nad6 -nad1 -rrnL -rrnS nad2 ]

n1(mod120)13g

[ cox1 cox2 cox3 nad3 -nad5 -nad4 -nad4L -cob -nad6 -nad1 -rrnL -rrnS nad2 ]

n4(mod120)

g1: [ cox1 cox2 nad1 nad3 -nad4 -nad5 -nad4L -cob -nad6 nad2 cox3 rrnS rrnL ]

g2: [ cox1 cox2 nad1 nad3 nad2 cox3 rrnS -nad5 -nad4 -nad4L -cob -nad6 rrnL ]

g3: [ cox1 cox2 nad1 nad6 cob nad4L nad5 nad4 nad3 nad2 cox3 rrnS rrnL ]

score = 276:

model 120:

-------------

R(0,1) R(0,9) R(0,10) R(0,17) R(0,18) R(0,24) R(0,25) R(0,28) R(0,n1) R(1,0) R(2,17) R(3,19) R(3,20) R(4,21) R(5,23) R(6,n1) R(6,n7) R(7,n7) R(8,24) R(9,0) R(10,0) R(11,27) R(12,31) R(13,14) R(13,15) R(13,n4) R(14,13) R(15,13) R(16,n5) R(17,0) R(17,2) R(18,0) R(18,19) R(19,3) R(19,18) R(19,22) R(20,3) R(20,21) R(21,4) R(21,20) R(22,19) R(22,23) R(23,5) R(23,22) R(24,0) R(24,8) R(25,0) R(25,26) R(26,25) R(26,27) R(27,11) R(27,26) R(28,0) R(28,29) R(29,28) R(29,30) R(30,29) R(30,31) R(31,12) R(31,30) R(n1,0) R(n1,6) R(n1,n2) R(n2,n1) R(n2,n3) R(n3,n2) R(n3,n4) R(n4,13) R(n4,n3) R(n4,n6) R(n5,16) R(n5,n6) R(n6,n4) R(n6,n5) R(n7,6) R(n7,7)

-------------------------------------------------------------------------------------------------------------

-> form outgroup SOL2 - B

n1(mod121)15g

[ cox1 cox2 atp8 atp6 cox3 nad3 -nad5 -nad4 -nad4L -cob -nad6 -nad1 -rrnL -rrnS nad2 ]

n1(mod121)13g

[ cox1 cox2 cox3 nad3 -nad5 -nad4 -nad4L -cob -nad6 -nad1 -rrnL -rrnS nad2 ]

score = 286:

model 121:

-------------

R(0,1) R(0,9) R(0,10) R(0,17) R(0,18) R(0,24) R(0,25) R(0,28) R(0,n1) R(1,0) R(2,17) R(3,19) R(3,20) R(4,21) R(5,23) R(6,n1) R(6,n6) R(7,n6) R(8,24) R(9,0) R(10,0) R(11,27) R(12,31) R(13,14) R(13,15) R(13,n4) R(13,n7) R(14,13) R(15,13) R(16,n5) R(17,0) R(17,2) R(18,0) R(18,19) R(19,3) R(19,18) R(19,22) R(20,3) R(20,21) R(21,4) R(21,20) R(22,19) R(22,23) R(23,5) R(23,22) R(24,0) R(24,8) R(25,0) R(25,26) R(26,25) R(26,27) R(27,11) R(27,26) R(28,0) R(28,29) R(29,28) R(29,30) R(30,29) R(30,31) R(31,12) R(31,30) R(n1,0) R(n1,6) R(n1,n2) R(n2,n1) R(n2,n3) R(n3,n2) R(n3,n4) R(n4,13) R(n4,n3) R(n5,16) R(n5,n7) R(n6,6) R(n6,7) R(n7,13) R(n7,n5)

-------------------------------------------------------------------------------------------------------------

-> form outgroup SOL2 - B

n1(mod122)15g

[ cox1 cox2 atp8 atp6 cox3 nad3 -nad5 -nad4 -nad4L -cob -nad6 -nad1 -rrnL -rrnS nad2 ]

n1(mod122)13g

[ cox1 cox2 cox3 nad3 -nad5 -nad4 -nad4L -cob -nad6 -nad1 -rrnL -rrnS nad2 ]

score = 300:

model 122:

-------------

R(0,1) R(0,9) R(0,10) R(0,17) R(0,18) R(0,24) R(0,25) R(0,28) R(0,n1) R(1,0) R(2,17) R(3,19) R(3,20) R(4,21) R(5,23) R(6,n1) R(6,n6) R(7,n6) R(8,24) R(9,0) R(10,0) R(11,27) R(12,31) R(13,14) R(13,15) R(13,n4) R(14,13) R(15,13) R(15,n7) R(16,n5) R(17,0) R(17,2) R(18,0) R(18,19) R(19,3) R(19,18) R(19,22) R(20,3) R(20,21) R(21,4) R(21,20) R(22,19) R(22,23) R(23,5) R(23,22) R(24,0) R(24,8) R(25,0) R(25,26) R(26,25) R(26,27) R(27,11) R(27,26) R(28,0) R(28,29) R(29,28) R(29,30) R(30,29) R(30,31) R(31,12) R(31,30) R(n1,0) R(n1,6) R(n1,n2) R(n2,n1) R(n2,n3) R(n3,n2) R(n3,n4) R(n4,13) R(n4,n3) R(n5,16) R(n5,n7) R(n6,6) R(n6,7) R(n7,15) R(n7,n5)

-------------------------------------------------------------------------------------------------------------

-> form outgroup SOL2 - B

n1(mod123)15g

[ cox1 cox2 atp8 atp6 cox3 nad3 -nad5 -nad4 -nad4L -cob -nad6 -nad1 -rrnL -rrnS nad2 ]

n1(mod123)13g

[ cox1 cox2 cox3 nad3 -nad5 -nad4 -nad4L -cob -nad6 -nad1 -rrnL -rrnS nad2 ]

n4(mod123)

[ cox1 cox2 nad1 nad3 nad2 cox3 rrnS -nad5 -nad4 -nad4L -cob -nad6 rrnL ]

score = 290:

model 123:

-------------

R(0,1) R(0,9) R(0,10) R(0,17) R(0,18) R(0,24) R(0,25) R(0,28) R(0,n1) R(1,0) R(2,17) R(3,19) R(3,20) R(4,21) R(5,23) R(6,n1) R(6,n7) R(7,n7) R(8,24) R(9,0) R(10,0) R(11,27) R(12,31) R(13,14) R(13,15) R(14,13) R(15,13) R(15,n4) R(16,n5) R(17,0) R(17,2) R(18,0) R(18,19) R(19,3) R(19,18) R(19,22) R(20,3) R(20,21) R(21,4) R(21,20) R(22,19) R(22,23) R(23,5) R(23,22) R(24,0) R(24,8) R(25,0) R(25,26) R(26,25) R(26,27) R(27,11) R(27,26) R(28,0) R(28,29) R(29,28) R(29,30) R(30,29) R(30,31) R(31,12) R(31,30) R(n1,0) R(n1,6) R(n1,n2) R(n2,n1) R(n2,n3) R(n3,n2) R(n3,n4) R(n4,15) R(n4,n3) R(n4,n6) R(n5,16) R(n5,n6) R(n6,n4) R(n6,n5) R(n7,6) R(n7,7)

-------------------------------------------------------------------------------------------------------------

-> form outgroup SOL2 - B

n1(mod124)15g

[ cox1 cox2 atp8 atp6 cox3 nad3 -nad5 -nad4 -nad4L -cob -nad6 -nad1 -rrnL -rrnS nad2 ]

n1(mod124)13g

[ cox1 cox2 cox3 nad3 -nad5 -nad4 -nad4L -cob -nad6 -nad1 -rrnL -rrnS nad2 ]

score = 312:

model 124:

-------------

R(0,1) R(0,9) R(0,10) R(0,17) R(0,18) R(0,24) R(0,25) R(0,28) R(0,n1) R(1,0) R(2,17) R(3,19) R(3,20) R(4,21) R(5,23) R(6,n1) R(6,n6) R(7,n6) R(8,24) R(9,0) R(10,0) R(11,27) R(12,31) R(13,14) R(13,15) R(13,n7) R(14,13) R(15,13) R(15,n4) R(16,n5) R(17,0) R(17,2) R(18,0) R(18,19) R(19,3) R(19,18) R(19,22) R(20,3) R(20,21) R(21,4) R(21,20) R(22,19) R(22,23) R(23,5) R(23,22) R(24,0) R(24,8) R(25,0) R(25,26) R(26,25) R(26,27) R(27,11) R(27,26) R(28,0) R(28,29) R(29,28) R(29,30) R(30,29) R(30,31) R(31,12) R(31,30) R(n1,0) R(n1,6) R(n1,n2) R(n2,n1) R(n2,n3) R(n3,n2) R(n3,n4) R(n4,15) R(n4,n3) R(n5,16) R(n5,n7) R(n6,6) R(n6,7) R(n7,13) R(n7,n5)

-------------------------------------------------------------------------------------------------------------

-> form outgroup SOL2 - B

n1(mod125)15g

[ cox1 cox2 atp8 atp6 cox3 nad3 -nad5 -nad4 -nad4L -cob -nad6 -nad1 -rrnL -rrnS nad2 ]

n1(mod125)13g

[ cox1 cox2 cox3 nad3 -nad5 -nad4 -nad4L -cob -nad6 -nad1 -rrnL -rrnS nad2 ]

score = 300:

model 125:

-------------

R(0,1) R(0,9) R(0,10) R(0,17) R(0,18) R(0,24) R(0,25) R(0,28) R(0,n1) R(1,0) R(2,17) R(3,19) R(3,20) R(4,21) R(5,23) R(6,n1) R(6,n6) R(7,n6) R(8,24) R(9,0) R(10,0) R(11,27) R(12,31) R(13,14) R(13,15) R(14,13) R(15,13) R(15,n4) R(15,n7) R(16,n5) R(17,0) R(17,2) R(18,0) R(18,19) R(19,3) R(19,18) R(19,22) R(20,3) R(20,21) R(21,4) R(21,20) R(22,19) R(22,23) R(23,5) R(23,22) R(24,0) R(24,8) R(25,0) R(25,26) R(26,25) R(26,27) R(27,11) R(27,26) R(28,0) R(28,29) R(29,28) R(29,30) R(30,29) R(30,31) R(31,12) R(31,30) R(n1,0) R(n1,6) R(n1,n2) R(n2,n1) R(n2,n3) R(n3,n2) R(n3,n4) R(n4,15) R(n4,n3) R(n5,16) R(n5,n7) R(n6,6) R(n6,7) R(n7,15) R(n7,n5)

-------------------------------------------------------------------------------------------------------------

-> form outgroup SOL2 - B

-> a new form for chaetognates group (different from the 13 usual forms), possible because of the specific value for n1

n1(mod126)15g

[ cox1 cox2 atp8 atp6 -nad5 -nad4 -nad4L nad6 cob -nad1 -rrnL -rrnS cox3 nad3 nad2 ]

n1(mod126)13g

[ cox1 cox2 -nad5 -nad4 -nad4L nad6 cob -nad1 -rrnL -rrnS cox3 nad3 nad2 ]

n4(mod126)

[ cox1 cox2 nad1 nad3 nad2 cox3 -nad4 -nad5 -cob -nad6 rrnS rrnL -nad4L ]

score = 302:

model 126:

-------------

R(0,1) R(0,9) R(0,10) R(0,17) R(0,18) R(0,24) R(0,25) R(0,28) R(0,n1) R(1,0) R(2,17) R(3,19) R(3,20) R(4,21) R(5,23) R(6,n1) R(6,n7) R(7,n7) R(8,24) R(9,0) R(10,0) R(11,27) R(12,31) R(13,14) R(13,15) R(13,n6) R(14,13) R(15,13) R(16,n5) R(17,0) R(17,2) R(18,0) R(18,19) R(19,3) R(19,18) R(19,22) R(20,3) R(20,21) R(21,4) R(21,20) R(22,19) R(22,23) R(23,5) R(23,22) R(24,0) R(24,8) R(25,0) R(25,26) R(26,25) R(26,27) R(27,11) R(27,26) R(28,0) R(28,29) R(29,28) R(29,30) R(30,29) R(30,31) R(31,12) R(31,30) R(n1,0) R(n1,6) R(n1,n2) R(n2,n1) R(n2,n3) R(n3,n2) R(n3,n4) R(n4,n3) R(n4,n5) R(n4,n6) R(n5,16) R(n5,n4) R(n6,13) R(n6,n4) R(n7,6) R(n7,7)

-------------------------------------------------------------------------------------------------------------

-> form outgroup SOL2 - B

-> a new form for chaetognates group (different from the 13 usual forms), possible because of the specific value for n1

n1(mod127)15g

[ cox1 cox2 atp8 atp6 -nad5 -nad4 -nad4L nad6 cob -nad1 -rrnL -rrnS cox3 nad3 nad2 ]

n1(mod127)13g

[ cox1 cox2 -nad5 -nad4 -nad4L nad6 cob -nad1 -rrnL -rrnS cox3 nad3 nad2 ]

n5(mod127)

[ cox1 cox2 nad1 nad3 nad2 cox3 rrnS rrnL nad6 cob nad5 nad4 -nad4L ]

score = 338:

model 127:

-------------

R(0,1) R(0,9) R(0,10) R(0,17) R(0,18) R(0,24) R(0,25) R(0,28) R(0,n1) R(1,0) R(2,17) R(3,19) R(3,20) R(4,21) R(5,23) R(6,n1) R(6,n6) R(7,n6) R(8,24) R(9,0) R(10,0) R(11,27) R(12,31) R(13,14) R(13,15) R(13,n7) R(14,13) R(15,13) R(16,n5) R(17,0) R(17,2) R(18,0) R(18,19) R(19,3) R(19,18) R(19,22) R(20,3) R(20,21) R(21,4) R(21,20) R(22,19) R(22,23) R(23,5) R(23,22) R(24,0) R(24,8) R(25,0) R(25,26) R(26,25) R(26,27) R(27,11) R(27,26) R(28,0) R(28,29) R(29,28) R(29,30) R(30,29) R(30,31) R(31,12) R(31,30) R(n1,0) R(n1,6) R(n1,n2) R(n2,n1) R(n2,n3) R(n3,n2) R(n3,n4) R(n4,n3) R(n4,n5) R(n5,16) R(n5,n4) R(n5,n7) R(n6,6) R(n6,7) R(n7,13) R(n7,n5)

-------------------------------------------------------------------------------------------------------------

-> form outgroup SOL2 - B

n1(mod128)15g

g1[ cox1 cox2 atp8 atp6 cox3 nad3 -nad5 -nad4 -nad4L -cob -nad6 -nad1 -rrnL -rrnS nad2 ]

g2[ cox1 cox2 atp8 atp6 -nad5 -nad4 -nad4L nad6 cob -nad1 -rrnL -rrnS cox3 nad3 nad2 ]

n1(mod128)13g

g1[ cox1 cox2 cox3 nad3 -nad5 -nad4 -nad4L -cob -nad6 -nad1 -rrnL -rrnS nad2 ]

g2[ cox1 cox2 -nad5 -nad4 -nad4L nad6 cob -nad1 -rrnL -rrnS cox3 nad3 nad2 ]

n7(mod128)

for n1-g1:

g1: [ cox1 cox2 nad1 nad3 nad2 cox3 rrnS -nad4 -nad5 -cob -nad6 -nad4L rrnL ]

g2: [ cox1 cox2 nad1 nad3 nad2 cox3 rrnS nad5 nad4 -nad4L -cob -nad6 rrnL ]

g3: [ cox1 cox2 nad1 nad3 nad2 cox3 rrnS rrnL nad6 cob nad4L nad5 nad4 ]

g4: [ cox1 cox2 nad1 nad3 nad2 cox3 rrnS nad6 cob nad4L -nad4 -nad5 rrnL ]

g5: [ cox1 cox2 nad1 nad3 nad2 cox3 rrnS -nad4 -nad5 nad6 cob nad4L rrnL ]

for n1-g2:

g6: [ cox1 cox2 nad1 nad3 nad2 cox3 rrnS -nad4 -nad5 -cob -nad6 rrnL -nad4L ]

g7: [ cox1 cox2 nad1 nad3 nad2 cox3 rrnS rrnL nad6 cob nad4L nad5 nad4 ]

g8: [ cox1 cox2 nad1 nad3 nad2 cox3 rrnS -nad4 -nad5 -cob -nad6 nad4L rrnL ]

g9: [ cox1 cox2 nad1 nad3 nad2 cox3 rrnS -nad4 -nad5 -rrnL -nad4L -cob -nad6 ]

score = 312:

model 128:

-------------

R(0,1) R(0,9) R(0,10) R(0,17) R(0,18) R(0,24) R(0,25) R(0,28) R(0,n1) R(1,0) R(2,17) R(3,19) R(3,20) R(4,21) R(5,23) R(6,n1) R(6,n6) R(7,n6) R(8,24) R(9,0) R(10,0) R(11,27) R(12,31) R(13,14) R(13,15) R(13,n7) R(14,13) R(15,13) R(16,n5) R(17,0) R(17,2) R(18,0) R(18,19) R(19,3) R(19,18) R(19,22) R(20,3) R(20,21) R(21,4) R(21,20) R(22,19) R(22,23) R(23,5) R(23,22) R(24,0) R(24,8) R(25,0) R(25,26) R(26,25) R(26,27) R(27,11) R(27,26) R(28,0) R(28,29) R(29,28) R(29,30) R(30,29) R(30,31) R(31,12) R(31,30) R(n1,0) R(n1,6) R(n1,n2) R(n2,n1) R(n2,n3) R(n3,n2) R(n3,n4) R(n4,n3) R(n4,n7) R(n5,16) R(n5,n7) R(n6,6) R(n6,7) R(n7,13) R(n7,n4) R(n7,n5)

-------------------------------------------------------------------------------------------------------------

-> form outgroup SOL2 - B

n1(mod129)15g

[ cox1 cox2 atp8 atp6 cox3 nad3 -nad5 -nad4 -nad4L -cob -nad6 -nad1 -rrnL -rrnS nad2 ]

n1(mod129)13g

[ cox1 cox2 cox3 nad3 -nad5 -nad4 -nad4L -cob -nad6 -nad1 -rrnL -rrnS nad2 ]

n4(mod129)

[ cox1 cox2 nad1 nad3 nad2 cox3 rrnS -nad5 -nad4 -nad4L -cob -nad6 rrnL ]

score = 264:

model 129:

-------------

R(0,1) R(0,9) R(0,10) R(0,17) R(0,18) R(0,24) R(0,25) R(0,28) R(0,n1) R(1,0) R(2,17) R(3,19) R(3,20) R(4,21) R(5,23) R(6,n1) R(6,n7) R(7,n7) R(8,24) R(9,0) R(10,0) R(11,27) R(12,31) R(13,14) R(13,n4) R(14,13) R(15,n4) R(16,n5) R(17,0) R(17,2) R(18,0) R(18,19) R(19,3) R(19,18) R(19,22) R(20,3) R(20,21) R(21,4) R(21,20) R(22,19) R(22,23) R(23,5) R(23,22) R(24,0) R(24,8) R(25,0) R(25,26) R(26,25) R(26,27) R(27,11) R(27,26) R(28,0) R(28,29) R(29,28) R(29,30) R(30,29) R(30,31) R(31,12) R(31,30) R(n1,0) R(n1,6) R(n1,n2) R(n2,n1) R(n2,n3) R(n3,n2) R(n3,n4) R(n4,13) R(n4,15) R(n4,n3) R(n4,n6) R(n5,16) R(n5,n6) R(n6,n4) R(n6,n5) R(n7,6) R(n7,7)

-------------------------------------------------------------------------------------------------------------

-> form outgroup SOL2 - B

n1(mod130)15g

[ cox1 cox2 atp8 atp6 cox3 nad3 -nad5 -nad4 -nad4L -cob -nad6 -nad1 -rrnL -rrnS nad2 ]

n1(mod130)13g

[ cox1 cox2 cox3 nad3 -nad5 -nad4 -nad4L -cob -nad6 -nad1 -rrnL -rrnS nad2 ]

n4(mod130)

[ cox1 cox2 nad1 nad3 nad2 cox3 rrnS -nad5 -nad4 -nad4L -cob -nad6 rrnL ]

score = 276:

model 130:

-------------

R(0,1) R(0,9) R(0,10) R(0,17) R(0,18) R(0,24) R(0,25) R(0,28) R(0,n1) R(1,0) R(2,17) R(3,19) R(3,20) R(4,21) R(5,23) R(6,n1) R(6,n6) R(7,n6) R(8,24) R(9,0) R(10,0) R(11,27) R(12,31) R(13,14) R(13,n4) R(13,n7) R(14,13) R(15,n4) R(16,n5) R(17,0) R(17,2) R(18,0) R(18,19) R(19,3) R(19,18) R(19,22) R(20,3) R(20,21) R(21,4) R(21,20) R(22,19) R(22,23) R(23,5) R(23,22) R(24,0) R(24,8) R(25,0) R(25,26) R(26,25) R(26,27) R(27,11) R(27,26) R(28,0) R(28,29) R(29,28) R(29,30) R(30,29) R(30,31) R(31,12) R(31,30) R(n1,0) R(n1,6) R(n1,n2) R(n2,n1) R(n2,n3) R(n3,n2) R(n3,n4) R(n4,13) R(n4,15) R(n4,n3) R(n5,16) R(n5,n7) R(n6,6) R(n6,7) R(n7,13) R(n7,n5)

-------------------------------------------------------------------------------------------------------------

-> form outgroup SOL2 - B

n1(mod131)15g

[ cox1 cox2 atp8 atp6 cox3 nad3 -nad5 -nad4 -nad4L -cob -nad6 -nad1 -rrnL -rrnS nad2 ]

n1(mod131)13g

[ cox1 cox2 cox3 nad3 -nad5 -nad4 -nad4L -cob -nad6 -nad1 -rrnL -rrnS nad2 ]

n4(mod131)

[ cox1 cox2 nad1 nad3 nad2 cox3 rrnS -nad5 -nad4 -nad4L -cob -nad6 rrnL ]

score = 278:

model 131:

-------------

R(0,1) R(0,9) R(0,10) R(0,17) R(0,18) R(0,24) R(0,25) R(0,28) R(0,n1) R(1,0) R(2,17) R(3,19) R(3,20) R(4,21) R(5,23) R(6,n1) R(6,n6) R(7,n6) R(8,24) R(9,0) R(10,0) R(11,27) R(12,31) R(13,14) R(13,n4) R(14,13) R(15,n4) R(15,n7) R(16,n5) R(17,0) R(17,2) R(18,0) R(18,19) R(19,3) R(19,18) R(19,22) R(20,3) R(20,21) R(21,4) R(21,20) R(22,19) R(22,23) R(23,5) R(23,22) R(24,0) R(24,8) R(25,0) R(25,26) R(26,25) R(26,27) R(27,11) R(27,26) R(28,0) R(28,29) R(29,28) R(29,30) R(30,29) R(30,31) R(31,12) R(31,30) R(n1,0) R(n1,6) R(n1,n2) R(n2,n1) R(n2,n3) R(n3,n2) R(n3,n4) R(n4,13) R(n4,15) R(n4,n3) R(n5,16) R(n5,n7) R(n6,6) R(n6,7) R(n7,15) R(n7,n5)

-------------------------------------------------------------------------------------------------------------

-> form outgroup SOL2 - B

n1(mod132)15g

[ cox1 cox2 atp8 atp6 cox3 nad3 -nad5 -nad4 -nad4L -cob -nad6 -nad1 -rrnL -rrnS nad2 ]

n1(mod132)13g

[ cox1 cox2 cox3 nad3 -nad5 -nad4 -nad4L -cob -nad6 -nad1 -rrnL -rrnS nad2 ]

n7(mod132)

[ cox1 cox2 nad1 nad3 nad2 cox3 rrnS -nad4L -nad4 -nad5 -cob -nad6 rrnL ]

score = 300:

model 132:

-------------

R(0,1) R(0,9) R(0,10) R(0,17) R(0,18) R(0,24) R(0,25) R(0,28) R(0,n1) R(1,0) R(2,17) R(3,19) R(3,20) R(4,21) R(5,23) R(6,n1) R(6,n6) R(7,n6) R(8,24) R(9,0) R(10,0) R(11,27) R(12,31) R(13,14) R(13,n4) R(13,n7) R(14,13) R(15,n7) R(16,n5) R(17,0) R(17,2) R(18,0) R(18,19) R(19,3) R(19,18) R(19,22) R(20,3) R(20,21) R(21,4) R(21,20) R(22,19) R(22,23) R(23,5) R(23,22) R(24,0) R(24,8) R(25,0) R(25,26) R(26,25) R(26,27) R(27,11) R(27,26) R(28,0) R(28,29) R(29,28) R(29,30) R(30,29) R(30,31) R(31,12) R(31,30) R(n1,0) R(n1,6) R(n1,n2) R(n2,n1) R(n2,n3) R(n3,n2) R(n3,n4) R(n4,13) R(n4,n3) R(n5,16) R(n5,n7) R(n6,6) R(n6,7) R(n7,13) R(n7,15) R(n7,n5)

-------------------------------------------------------------------------------------------------------------

-> form outgroup SOL2 - B

n1(mod133)15g

[ cox1 cox2 atp8 atp6 cox3 nad3 -nad5 -nad4 -nad4L -cob -nad6 -nad1 -rrnL -rrnS nad2 ]

n1(mod133)13g

[ cox1 cox2 cox3 nad3 -nad5 -nad4 -nad4L -cob -nad6 -nad1 -rrnL -rrnS nad2 ]

n4(mod133)

[ cox1 cox2 nad1 nad3 nad2 cox3 rrnS -nad5 -nad4 -nad4L -cob -nad6 rrnL ]

n6(mod133)

[ cox1 cox2 nad1 nad3 nad2 cox3 rrnS nad5 nad4 -nad4L -cob -nad6 rrnL ]

score = 290:

model 133:

-------------

R(0,1) R(0,9) R(0,10) R(0,17) R(0,18) R(0,24) R(0,25) R(0,28) R(0,n1) R(1,0) R(2,17) R(3,19) R(3,20) R(4,21) R(5,23) R(6,n1) R(6,n7) R(7,n7) R(8,24) R(9,0) R(10,0) R(11,27) R(12,31) R(13,14) R(13,n6) R(14,13) R(15,n4) R(16,n5) R(17,0) R(17,2) R(18,0) R(18,19) R(19,3) R(19,18) R(19,22) R(20,3) R(20,21) R(21,4) R(21,20) R(22,19) R(22,23) R(23,5) R(23,22) R(24,0) R(24,8) R(25,0) R(25,26) R(26,25) R(26,27) R(27,11) R(27,26) R(28,0) R(28,29) R(29,28) R(29,30) R(30,29) R(30,31) R(31,12) R(31,30) R(n1,0) R(n1,6) R(n1,n2) R(n2,n1) R(n2,n3) R(n3,n2) R(n3,n4) R(n4,15) R(n4,n3) R(n4,n6) R(n5,16) R(n5,n6) R(n6,13) R(n6,n4) R(n6,n5) R(n7,6) R(n7,7)

-------------------------------------------------------------------------------------------------------------

-> form outgroup SOL2 - B

n1(mod134)15g

[ cox1 cox2 atp8 atp6 cox3 nad3 -nad5 -nad4 -nad4L -cob -nad6 -nad1 -rrnL -rrnS nad2 ]

n1(mod134)13g

[ cox1 cox2 cox3 nad3 -nad5 -nad4 -nad4L -cob -nad6 -nad1 -rrnL -rrnS nad2 ]

n6(mod134)

[ cox1 cox2 nad1 nad3 nad2 cox3 rrnS -nad4L -nad4 -nad5 -cob -nad6 rrnL ]

score = 326:

model 134:

-------------

R(0,1) R(0,9) R(0,10) R(0,17) R(0,18) R(0,24) R(0,25) R(0,28) R(0,n1) R(1,0) R(2,17) R(3,19) R(3,20) R(4,21) R(5,23) R(6,n1) R(6,n7) R(7,n7) R(8,24) R(9,0) R(10,0) R(11,27) R(12,31) R(13,14) R(13,n6) R(14,13) R(15,n4) R(15,n6) R(16,n5) R(17,0) R(17,2) R(18,0) R(18,19) R(19,3) R(19,18) R(19,22) R(20,3) R(20,21) R(21,4) R(21,20) R(22,19) R(22,23) R(23,5) R(23,22) R(24,0) R(24,8) R(25,0) R(25,26) R(26,25) R(26,27) R(27,11) R(27,26) R(28,0) R(28,29) R(29,28) R(29,30) R(30,29) R(30,31) R(31,12) R(31,30) R(n1,0) R(n1,6) R(n1,n2) R(n2,n1) R(n2,n3) R(n3,n2) R(n3,n4) R(n4,15) R(n4,n3) R(n5,16) R(n5,n6) R(n6,13) R(n6,15) R(n6,n5) R(n7,6) R(n7,7)

-------------------------------------------------------------------------------------------------------------

-> form outgroup SOL1 - C

n2(mod135)15g

[ cox1 cox2 atp8 atp6 cox3 nad3 -nad5 -nad4 -nad4L nad6 cob rrnS rrnL nad1 nad2 ]

n2(mod135)13g

[ cox1 cox2 cox3 nad3 -nad5 -nad4 -nad4L nad6 cob rrnS rrnL nad1 nad2 ]

n7(mod135)

[ cox1 cox2 nad1 nad3 nad2 cox3 rrnS -nad4 -nad5 -cob -nad6 nad4L rrnL ]

score = 348:

model 135:

-------------

R(0,1) R(0,9) R(0,10) R(0,17) R(0,18) R(0,24) R(0,25) R(0,28) R(0,n1) R(1,0) R(2,17) R(3,19) R(3,20) R(4,21) R(5,23) R(6,n2) R(7,n2) R(8,24) R(9,0) R(10,0) R(11,27) R(12,31) R(13,14) R(13,15) R(13,n7) R(14,13) R(15,13) R(16,n5) R(17,0) R(17,2) R(18,0) R(18,19) R(19,3) R(19,18) R(19,22) R(20,3) R(20,21) R(21,4) R(21,20) R(22,19) R(22,23) R(23,5) R(23,22) R(24,0) R(24,8) R(25,0) R(25,26) R(26,25) R(26,27) R(27,11) R(27,26) R(28,0) R(28,29) R(29,28) R(29,30) R(30,29) R(30,31) R(31,12) R(31,30) R(n1,0) R(n1,n2) R(n2,6) R(n2,7) R(n2,n1) R(n2,n3) R(n3,n2) R(n3,n4) R(n4,n3) R(n4,n6) R(n5,16) R(n5,n7) R(n6,n4) R(n6,n7) R(n7,13) R(n7,n5) R(n7,n6)

-------------------------------------------------------------------------------------------------------------

-> form outgroup SOL7 - A

n2(mod136)

[ cox1 cox2 atp8 atp6 cox3 nad3 -nad5 -nad4 -nad4L nad6 cob rrnS rrnL nad1 nad2 ]

n3(mod136)15g

[ cox1 cox2 atp8 atp6 cox3 rrnS rrnL nad1 nad3 -nad5 -nad4 -nad4L nad6 cob nad2 ]

n3(mod136)13g

[ cox1 cox2 cox3 rrnS rrnL nad1 nad3 -nad5 -nad4 -nad4L nad6 cob nad2 ]

n7(mod136)

[ cox1 cox2 nad1 nad3 nad2 cox3 rrnS -nad4 -nad5 -cob -nad6 nad4L rrnL ]

score = 356:

model 136:

-------------

R(0,1) R(0,9) R(0,10) R(0,17) R(0,18) R(0,24) R(0,25) R(0,28) R(0,n1) R(1,0) R(2,17) R(3,19) R(3,20) R(4,21) R(5,23) R(6,n3) R(7,n2) R(8,24) R(9,0) R(10,0) R(11,27) R(12,31) R(13,14) R(13,15) R(13,n7) R(14,13) R(15,13) R(16,n5) R(17,0) R(17,2) R(18,0) R(18,19) R(19,3) R(19,18) R(19,22) R(20,3) R(20,21) R(21,4) R(21,20) R(22,19) R(22,23) R(23,5) R(23,22) R(24,0) R(24,8) R(25,0) R(25,26) R(26,25) R(26,27) R(27,11) R(27,26) R(28,0) R(28,29) R(29,28) R(29,30) R(30,29) R(30,31) R(31,12) R(31,30) R(n1,0) R(n1,n2) R(n2,7) R(n2,n1) R(n2,n3) R(n3,6) R(n3,n2) R(n3,n4) R(n4,n3) R(n4,n6) R(n5,16) R(n5,n7) R(n6,n4) R(n6,n7) R(n7,13) R(n7,n5) R(n7,n6)

-------------------------------------------------------------------------------------------------------------

-> form outgroup SOL3 - C

n1(mod137)

[ cox1 cox2 atp8 atp6 cox3 nad3 -nad5 -nad4 -nad4L -cob -nad6 -nad1 -rrnL -rrnS nad2 ]

n7(mod137)

[ cox1 cox2 nad1 nad3 nad2 cox3 rrnS -nad4 -nad5 -cob -nad6 nad4L rrnL ]

score = 342:

model 137:

-------------

R(0,1) R(0,9) R(0,10) R(0,17) R(0,18) R(0,24) R(0,25) R(0,28) R(0,n1) R(1,0) R(2,17) R(3,19) R(3,20) R(4,21) R(5,23) R(6,n1) R(6,n3) R(7,n2) R(8,24) R(9,0) R(10,0) R(11,27) R(12,31) R(13,14) R(13,15) R(13,n7) R(14,13) R(15,13) R(16,n5) R(17,0) R(17,2) R(18,0) R(18,19) R(19,3) R(19,18) R(19,22) R(20,3) R(20,21) R(21,4) R(21,20) R(22,19) R(22,23) R(23,5) R(23,22) R(24,0) R(24,8) R(25,0) R(25,26) R(26,25) R(26,27) R(27,11) R(27,26) R(28,0) R(28,29) R(29,28) R(29,30) R(30,29) R(30,31) R(31,12) R(31,30) R(n1,0) R(n1,6) R(n1,n2) R(n2,7) R(n2,n1) R(n3,6) R(n3,n4) R(n4,n3) R(n4,n6) R(n5,16) R(n5,n7) R(n6,n4) R(n6,n7) R(n7,13) R(n7,n5) R(n7,n6)

-------------------------------------------------------------------------------------------------------------

-> form outgroup SOL1 - D

n2(mod138)

[ cox1 cox2 atp8 atp6 cox3 nad3 -nad5 -nad4 -nad4L nad6 cob rrnS rrnL nad1 nad2 ]

n7(mod138)

[ cox1 cox2 nad1 nad3 nad2 cox3 rrnS -nad4 -nad5 -cob -nad6 nad4L rrnL ]

score = 392:

model 138:

-------------

R(0,1) R(0,9) R(0,10) R(0,17) R(0,18) R(0,24) R(0,25) R(0,28) R(0,n1) R(1,0) R(2,17) R(3,19) R(3,20) R(4,21) R(5,23) R(6,n2) R(6,n3) R(7,n2) R(8,24) R(9,0) R(10,0) R(11,27) R(12,31) R(13,14) R(13,15) R(13,n7) R(14,13) R(15,13) R(16,n5) R(17,0) R(17,2) R(18,0) R(18,19) R(19,3) R(19,18) R(19,22) R(20,3) R(20,21) R(21,4) R(21,20) R(22,19) R(22,23) R(23,5) R(23,22) R(24,0) R(24,8) R(25,0) R(25,26) R(26,25) R(26,27) R(27,11) R(27,26) R(28,0) R(28,29) R(29,28) R(29,30) R(30,29) R(30,31) R(31,12) R(31,30) R(n1,0) R(n1,n2) R(n2,6) R(n2,7) R(n2,n1) R(n3,6) R(n3,n4) R(n4,n3) R(n4,n6) R(n5,16) R(n5,n7) R(n6,n4) R(n6,n7) R(n7,13) R(n7,n5) R(n7,n6)

-------------------------------------------------------------------------------------------------------------

-> form outgroup SOL2 - C

n4(mod139)

[ cox1 cox2 nad1 nad3 nad2 cox3 rrnS -nad4 -nad5 -cob -nad6 nad4L rrnL ]

score = 348:

model 139:

-------------

R(0,1) R(0,9) R(0,10) R(0,17) R(0,18) R(0,24) R(0,25) R(0,28) R(0,n1) R(1,0) R(2,17) R(3,19) R(3,20) R(4,21) R(5,23) R(6,n1) R(6,n2) R(6,n3) R(7,n3) R(8,24) R(9,0) R(10,0) R(11,27) R(12,31) R(13,14) R(13,15) R(13,n4) R(14,13) R(15,13) R(16,n5) R(17,0) R(17,2) R(18,0) R(18,19) R(19,3) R(19,18) R(19,22) R(20,3) R(20,21) R(21,4) R(21,20) R(22,19) R(22,23) R(23,5) R(23,22) R(24,0) R(24,8) R(25,0) R(25,26) R(26,25) R(26,27) R(27,11) R(27,26) R(28,0) R(28,29) R(29,28) R(29,30) R(30,29) R(30,31) R(31,12) R(31,30) R(n1,0) R(n1,6) R(n2,6) R(n2,n7) R(n3,6) R(n3,7) R(n4,13) R(n4,n5) R(n4,n6) R(n5,16) R(n5,n4) R(n6,n4) R(n6,n7) R(n7,n2) R(n7,n6)

-------------------------------------------------------------------------------------------------------------

-> form outgroup SOL8 - A

n2(mod140)15g

[ cox1 cox2 atp8 atp6 cox3 rrnS rrnL nad1 nad3 -nad5 -nad4 -nad4L nad6 cob nad2 ]

n2(mod140)13g

[ cox1 cox2 cox3 rrnS rrnL nad1 nad3 -nad5 -nad4 -nad4L nad6 cob nad2 ]

n6(mod140)

[ cox1 cox2 nad1 nad3 nad2 cox3 rrnS -nad4 -nad5 -cob -nad6 nad4L rrnL ]

score = 356:

model 140:

-------------

R(0,1) R(0,9) R(0,10) R(0,17) R(0,18) R(0,24) R(0,25) R(0,28) R(0,n1) R(1,0) R(2,17) R(3,19) R(3,20) R(4,21) R(5,23) R(6,n1) R(6,n2) R(7,n4) R(8,24) R(9,0) R(10,0) R(11,27) R(12,31) R(13,14) R(13,15) R(13,n6) R(14,13) R(15,13) R(16,n5) R(17,0) R(17,2) R(18,0) R(18,19) R(19,3) R(19,18) R(19,22) R(20,3) R(20,21) R(21,4) R(21,20) R(22,19) R(22,23) R(23,5) R(23,22) R(24,0) R(24,8) R(25,0) R(25,26) R(26,25) R(26,27) R(27,11) R(27,26) R(28,0) R(28,29) R(29,28) R(29,30) R(30,29) R(30,31) R(31,12) R(31,30) R(n1,0) R(n1,6) R(n2,6) R(n2,n3) R(n2,n4) R(n3,n2) R(n3,n7) R(n4,7) R(n4,n2) R(n5,16) R(n5,n6) R(n6,13) R(n6,n5) R(n6,n7) R(n7,n3) R(n7,n6)

-------------------------------------------------------------------------------------------------------------

-> form outgroup SOL2 - D

n2(mod141)15g

[ cox1 cox2 atp8 atp6 cox3 nad3 -nad5 -nad4 -nad4L nad6 cob rrnS rrnL nad1 nad2 ]

n2(mod141)13g

[ cox1 cox2 cox3 nad3 -nad5 -nad4 -nad4L nad6 cob rrnS rrnL nad1 nad2 ]

n7(mod141)

[ cox1 cox2 nad1 nad3 nad2 cox3 rrnS -nad4 -nad5 -cob -nad6 nad4L rrnL ]

score = 392:

model 141:

-------------

R(0,1) R(0,9) R(0,10) R(0,17) R(0,18) R(0,24) R(0,25) R(0,28) R(0,n1) R(1,0) R(2,17) R(3,19) R(3,20) R(4,21) R(5,23) R(6,n1) R(6,n2) R(7,n2) R(8,24) R(9,0) R(10,0) R(11,27) R(12,31) R(13,14) R(13,15) R(13,n7) R(14,13) R(15,13) R(16,n5) R(17,0) R(17,2) R(18,0) R(18,19) R(19,3) R(19,18) R(19,22) R(20,3) R(20,21) R(21,4) R(21,20) R(22,19) R(22,23) R(23,5) R(23,22) R(24,0) R(24,8) R(25,0) R(25,26) R(26,25) R(26,27) R(27,11) R(27,26) R(28,0) R(28,29) R(29,28) R(29,30) R(30,29) R(30,31) R(31,12) R(31,30) R(n1,0) R(n1,6) R(n2,6) R(n2,7) R(n2,n3) R(n3,n2) R(n3,n4) R(n4,n3) R(n4,n6) R(n5,16) R(n5,n7) R(n6,n4) R(n6,n7) R(n7,13) R(n7,n5) R(n7,n6)

-------------------------------------------------------------------------------------------------------------

-> no other solutions

chaetognaths_taxB_96sol

================================================================================

================================================================================

AXIOMS

================================================================================

================================================================================

{ the solutions of problem PHYLO are the smallest graphs T (defined on the smallest domain possible but containing at least all the OTUs) which verify properties P1 to P6:

P1- T is simple (the relation R(x, y) which defines graph T is not reflexive)

P2- T is non-oriented (the relation R(x, y) which defines graph T is symmetrical)

P3- T is connected and acyclic (T is a tree)

P4- T respects the minimal distance matrix, i.e.:

for all couple of OTUs x and y, the length of the path x->y in T is always superior or equals to the minimal distance calculated between x and y (encoded in the minimal distance matrix)

P5- T respects other eventual hypothesis (Primary Phylogenetic Hypothesis = PPH)

used to impose the existence of given monophyletic groups

P6- it is possible to calculate all the values for each HTU in the graph T }

{ OTUs: }

katharina_tunicata = 0;

nautilus_macromphallus = 1;

loligo_bleekeri = 2;

platynereis_dumerilii = 3;

urechis_caupo = 4;

sipunculus_nudus = 5;

priapulus_caudatus = 6; { = outgroup1 }

homo_sapiens = 7; { = outgroup2 }

loxocorone_allax = 8;

terebratulina_retusa = 9;

phoronis_architecta = 10;

bugula_neritina=11;

terebratalia_transversa=12;

sagitta_enflata=13;

sagitta_nagae=14;

paraspadella_gotoi=15;

spadella_cephaloptera=16;

{ AUXILLIARY CONSTANTS used to fix a part of the solution: }

G1=17; G2=18; G3=19; G4=20;

G5=21; G6=22; G7=23; G8=24; { used to fix the Eutrochozoa group }

G9=25; G10=26; G11=27; G12=28;

G13=29; G14=30; G15=31; { used to fix the Lophophorata group }

{ THE EUTROCHOZOA GROUP IS FIXED: }

{ OTUs (0,1,2): }

R(katharina_tunicata,nautilus_macromphallus);

R(katharina_tunicata,G1);

R(G1,loligo_bleekeri);

Q x ( x<>katharina_tunicata

=>

-R(nautilus_macromphallus,x)

);

Q x ( ( x<>katharina_tunicata et

x<>loligo_bleekeri

)

=>

-R(G1,x)

);

Q x ( x<>G1

=>

-R(loligo_bleekeri,x)

);

{ OTUs (3,4,5,8): }

{ as in the best of the 3 possible forms }

R(katharina_tunicata,G2);

R(G2,G3);

R(G3,platynereis_dumerilii);

R(platynereis_dumerilii,G4);

R(G4,G5);

R(G5,urechis_caupo);

R(G3,G6);

R(G6,G7);

R(G7,sipunculus_nudus);

Q x ( ( x<>katharina_tunicata et

x<>G3

)

=>

-R(G2,x)

);

Q x ( ( x<>G2 et

x<>platynereis_dumerilii et

x<>G6

)

=>

-R(G3,x)

);

Q x ( ( x<>platynereis_dumerilii et

x<>G5

)

=>

-R(G4,x)

);

Q x ( ( x<>G3 et

x<>G4

)

=>

-R(platynereis_dumerilii,x)

);

Q x ( ( x<>G4 et

x<>urechis_caupo

)

=>

-R(G5,x)

);

Q x ( x<>G5

=>

-R(urechis_caupo,x)

);

Q x ( ( x<>G3 et

x<>G7

)

=>

-R(G6,x)

);

Q x ( ( x<>G6 et

x<>sipunculus_nudus

)

=>

-R(G7,x)

);

Q x ( x<>G7

=>

-R(sipunculus_nudus,x)

);

R(katharina_tunicata,G8);

R(G8,loxocorone_allax);

Q x ( ( x<>katharina_tunicata et

x<>loxocorone_allax

)

=>

-R(G8,x)

);

Q x ( x<>G8

=>

-R(loxocorone_allax, x)

);

{ THE LOPHOPHORATA GROUP IS FIXED: }

{ as in the best of the 9 possible forms }

R(katharina_tunicata,terebratulina_retusa);

R(katharina_tunicata,phoronis_architecta);

Q x ( x<>katharina_tunicata

=>

-R(terebratulina_retusa,x)

);

Q x ( x<>katharina_tunicata

=>

-R(phoronis_architecta,x)

);

R(katharina_tunicata,G9);

R(G9,G10);

R(G10,G11);

R(G11,bugula_neritina);

Q x ( ( x<>katharina_tunicata et

x<>G10

)

=>

-R(G9,x)

);

Q x ( ( x<>G9 et

x<>G11

)

=>

-R(G10,x)

);

Q x ( ( x<>G10 et

x<>bugula_neritina

)

=>

-R(G11,x)

);

Q x ( x<>G11

=>

-R(bugula_neritina,x)

);

R(katharina_tunicata,G12);

R(G12,G13);

R(G13,G14);

R(G14,G15);

R(G15,terebratalia_transversa);

Q x ( ( x<>katharina_tunicata et

x<>G13

)

=>

-R(G12,x)

);

Q x ( ( x<>G12 et

x<>G14

)

=>

-R(G13,x)

);

Q x ( ( x<>G13 et

x<>G15

)

=>

-R(G14,x)

);

Q x ( ( x<>G14 et

x<>terebratalia_transversa

)

=>

-R(G15,x)

);

Q x ( x<>G15

=>

-R(terebratalia_transversa,x)

);

{ PROPERTY P1: R(x, y) is not reflexive}

Q x (-R(x, x));

{ PROPERTY P2: R(x, y) is symmetrical}

Q x y (R(x, y) => R(y, x));

{ PROPERTY P3: graph T is connected and acyclic (T is a tree) }

{

This property is verified by a constraint programmed in the model generator, instead of a "heavy" logical formula:

1- it will refuse the partial interpretations in which a connected component of the graph (in construction) is cyclic, i.e. such as: number of edges >= number of vertices

2- it will refuse the complete interpretations in which the constructed graph has more than one connected component

}

{ PROPERTY P4: graph T respects minimal distance matrix }

{

This property is verified by a constraint programmed in the model generator:

it will refuse the partial interpretations in which the graph (in construction) do not respect the minimal distance matrix, i.e. such as:

let x, y a couple of OTUs,

let d= minimal distance calculated between x and y (encoded in the minimal distance matrix), there is a a path of length k between x and y, with: k < d

The minimal distance matrix is encoded directly in the data structure of the model generator:

/* minimal distance matrix chaetognaths taxB: */

DIST[0][0]=0;

DIST[1][0]=1; DIST[1][1]=0;

DIST[2][0]=2; DIST[2][1]=3; DIST[2][2]=0;

DIST[3][0]=3; DIST[3][1]=4; DIST[3][2]=4; DIST[3][3]=0;

DIST[4][0]=5; DIST[4][1]=5; DIST[4][2]=5; DIST[4][3]=3; DIST[4][4]=0;

DIST[5][0]=4; DIST[5][1]=4; DIST[5][2]=5; DIST[5][3]=3; DIST[5][4]=4; DIST[5][5]=0;

DIST[6][0]=2; DIST[6][1]=3; DIST[6][2]=2; DIST[6][3]=4; DIST[6][4]=4; DIST[6][5]=4; DIST[6][6]=0;

DIST[7][0]=3; DIST[7][1]=4; DIST[7][2]=3; DIST[7][3]=4; DIST[7][4]=5; DIST[7][5]=4; DIST[7][6]=2; DIST[7][7]=0;

DIST[8][0]=2; DIST[8][1]=2; DIST[8][2]=4; DIST[8][3]=5; DIST[8][4]=5; DIST[8][5]=5; DIST[8][6]=4; DIST[8][7]=4; DIST[8][8]=0;

DIST[9][0]=1; DIST[9][1]=2; DIST[9][2]=2; DIST[9][3]=3; DIST[9][4]=4; DIST[9][5]=5; DIST[9][6]=2; DIST[9][7]=3; DIST[9][8]=3; DIST[9][9]=0;

DIST[10][0]=1; DIST[10][1]=2; DIST[10][2]=3; DIST[10][3]=4; DIST[10][4]=5; DIST[10][5]=4; DIST[10][6]=3; DIST[10][7]=4; DIST[10][8]=3; DIST[10][9]=2; DIST[10][10]=0;

DIST[11][0]=4; DIST[11][1]=5; DIST[11][2]=5; DIST[11][3]=6; DIST[11][4]=6; DIST[11][5]=6; DIST[11][6]=5; DIST[11][7]=5; DIST[11][8]=5; DIST[11][9]=4; DIST[11][10]=4; DIST[11][11]=0;

DIST[12][0]=5; DIST[12][1]=6; DIST[12][2]=6; DIST[12][3]=5; DIST[12][4]=7; DIST[12][5]=6; DIST[12][6]=6; DIST[12][7]=7; DIST[12][8]=7; DIST[12][9]=5; DIST[12][10]=5; DIST[12][11]=7; DIST[12][12]=0;

DIST[13][0]=4; DIST[13][1]=4; DIST[13][2]=5; DIST[13][3]=3; DIST[13][4]=5; DIST[13][5]=5; DIST[13][6]=5; DIST[13][7]=5; DIST[13][8]=5; DIST[13][9]=4; DIST[13][10]=4; DIST[13][11]=4; DIST[13][12]=6; DIST[13][13]=0;

DIST[14][0]=5; DIST[14][1]=4; DIST[14][2]=6; DIST[14][3]=4; DIST[14][4]=6; DIST[14][5]=6; DIST[14][6]=5; DIST[14][7]=5; DIST[14][8]=5; DIST[14][9]=5; DIST[14][10]=5; DIST[14][11]=4; DIST[14][12]=6; DIST[14][13]=1; DIST[14][14]=0;

DIST[15][0]=4; DIST[15][1]=4; DIST[15][2]=5; DIST[15][3]=4; DIST[15][4]=5; DIST[15][5]=5; DIST[15][6]=5; DIST[15][7]=5; DIST[15][8]=5; DIST[15][9]=4; DIST[15][10]=4; DIST[15][11]=4; DIST[15][12]=5; DIST[15][13]=1; DIST[15][14]=2; DIST[15][15]=0;

DIST[16][0]=6; DIST[16][1]=5; DIST[16][2]=6; DIST[16][3]=4; DIST[16][4]=5; DIST[16][5]=6; DIST[16][6]=6; DIST[16][7]=6; DIST[16][8]=5; DIST[16][9]=5; DIST[16][10]=5; DIST[16][11]=5; DIST[16][12]=6; DIST[16][13]=3; DIST[16][14]=4; DIST[16][15]=3; DIST[16][16]=0;

}

{ PROPERTY P5: graph T respects eventual Primary Phylogenetic Hypotheses }

{

This property is verified by constraints programmed in the model generator:

- monophyly of Lophotrochozoa = (0,1,2,3,4,5,8,9,10,11,12)

- monophyly of Eutrochozoa = (0,1,2,3,4,5,8)

- monophyly of Mollusca = (0,1,2)

- monophyly of Cephalopoda = (1,2)

- monophyly of Annelida = (3,4)

- monophyly of Lophophorata = (9,10,11,12)

- monophyly of Chaetognatha = (13,14,15,16)

}

{------------------------------------------------------------------------------------------------------------------------}

{ PROPERTY P6: it is possible to calculate all the values for each HTU in the graph T }

{

First we calculate with the model generator the set of tree solutions which verify properties P1 to P5. Property P6 is verified *a posteriori* for each tree solution, with a *feedback* mechanism:

Studying each tree solution for calculating the values of HTUs, we eventually discover "impossible sub-trees": they appear in tree solutions which verify P1 to P5, but they do not verify P6.

For each impossible subtree A, an additional constraint is programmed into the model generator to forbid the solutions containing A. Tree solutions are recalculated and verified, allowing the discovery of new impossible subtrees and the programming of new constraints to recalculate the solutions (feedback mechanism). Finally, the complete set of optimal solutions is determined after iteration of this process and elimination of all the solutions that do not verify P6.

}

================================================================================

================================================================================

SOLUTIONS

================================================================================

================================================================================

OTUs:

katharina_tunicata = 0;

nautilus_macromphallus = 1;

loligo_bleekeri = 2;

platynereis_dumerilii = 3;

urechis_caupo = 4;

sipunculus_nudus = 5;

priapulus_caudatus = 6; { = outgroup1 }

homo_sapiens = 7; { = outgroup2 }

loxocorone_allax = 8;

terebratulina_retusa = 9;

phoronis_architecta = 10;

bugula_neritina=11;

terebratalia_transversa=12;

sagitta_enflata=13;

sagitta_nagae=14;

paraspadella_gotoi=15;

spadella_cephaloptera=16;

AUXILLIARY CONSTANTS used to fix a part of the solution:

G1=17; G2=18; G3=19; G4=20;

G5=21; G6=22; G7=23; G8=24; { used to fix the Eutrochozoa group }

G9=25; G10=26; G11=27; G12=28;

G13=29; G14=30; G15=31; { used to fix the Lophophorata group }

HTUs:

n1, n2, n3, n4, n5, n6, n7

NOTE:

In every solution, we have to *insert* in the chaetognaths lineage (OTUs 13,14,15,16) one mutation "loss of the 2 successive genes atp8-atp6" proper to the chaetognaths lineage (common for all chaetognaths): it is the most parsimonious possibility.

In every solution of this file, this "loss" mutation can be inserted at the *beginning* of the chaetognaths lineage, and the possible values for the corresponding ancestral state are given first with 15 genes (before the loss) and then with 13 genes (after the loss of genes atp8-atp6).

But it is possible to insert this loss mutation *at* *any position* proper to the chaetognaths lineage, and easily reconstruct the possible values for ancestral states (with 15 genes) at the beginning of the chaetognaths lineage, before the loss mutation.

D = [0,38]: 96 solutions OK (which verify property P6) (191 impossible sub-trees)

minimal score (best) = 229

maximal score = 393

-------------------------------------------------------------------------------------------------------------

-> form outgroup SOL2_ALTER - A

score = 251:

model 1:

-------------

R(0,1) R(0,9) R(0,10) R(0,17) R(0,18) R(0,24) R(0,25) R(0,28) R(0,n1) R(0,n2) R(1,0) R(2,17) R(3,19) R(3,20) R(4,21) R(5,23) R(6,n2) R(6,n3) R(7,n3) R(8,24) R(9,0) R(10,0) R(11,27) R(12,31) R(13,14) R(13,15) R(13,n4) R(13,n5) R(14,13) R(15,13) R(16,n6) R(17,0) R(17,2) R(18,0) R(18,19) R(19,3) R(19,18) R(19,22) R(20,3) R(20,21) R(21,4) R(21,20) R(22,19) R(22,23) R(23,5) R(23,22) R(24,0) R(24,8) R(25,0) R(25,26) R(26,25) R(26,27) R(27,11) R(27,26) R(28,0) R(28,29) R(29,28) R(29,30) R(30,29) R(30,31) R(31,12) R(31,30) R(n1,0) R(n1,n7) R(n2,0) R(n2,6) R(n3,6) R(n3,7) R(n4,13) R(n4,n6) R(n5,13) R(n5,n7) R(n6,16) R(n6,n4) R(n7,n1) R(n7,n5)

-------------------------------------------------------------------------------------------------------------

-> form outgroup SOL1_ALTER - A

n3(mod2)

[ cox1 cox2 atp8 atp6 cox3 nad3 -nad5 -nad4 -nad4L nad6 cob rrnS rrnL nad1 nad2 ]

score = 251:

model 2:

-------------

R(0,1) R(0,9) R(0,10) R(0,17) R(0,18) R(0,24) R(0,25) R(0,28) R(0,n1) R(0,n2) R(1,0) R(2,17) R(3,19) R(3,20) R(4,21) R(5,23) R(6,n3) R(7,n3) R(8,24) R(9,0) R(10,0) R(11,27) R(12,31) R(13,14) R(13,15) R(13,n4) R(13,n5) R(14,13) R(15,13) R(16,n6) R(17,0) R(17,2) R(18,0) R(18,19) R(19,3) R(19,18) R(19,22) R(20,3) R(20,21) R(21,4) R(21,20) R(22,19) R(22,23) R(23,5) R(23,22) R(24,0) R(24,8) R(25,0) R(25,26) R(26,25) R(26,27) R(27,11) R(27,26) R(28,0) R(28,29) R(29,28) R(29,30) R(30,29) R(30,31) R(31,12) R(31,30) R(n1,0) R(n1,n7) R(n2,0) R(n2,n3) R(n3,6) R(n3,7) R(n3,n2) R(n4,13) R(n4,n6) R(n5,13) R(n5,n7) R(n6,16) R(n6,n4) R(n7,n1) R(n7,n5)

-------------------------------------------------------------------------------------------------------------

-> form outgroup SOL2_ALTER - A

n4(mod3)

[ cox1 cox2 nad1 nad3 nad2 cox3 rrnS -nad4L -nad4 -nad5 -cob -nad6 rrnL ]

score = 265:

model 3:

-------------

R(0,1) R(0,9) R(0,10) R(0,17) R(0,18) R(0,24) R(0,25) R(0,28) R(0,n1) R(0,n2) R(1,0) R(2,17) R(3,19) R(3,20) R(4,21) R(5,23) R(6,n2) R(6,n3) R(7,n3) R(8,24) R(9,0) R(10,0) R(11,27) R(12,31) R(13,14) R(13,n4) R(13,n5) R(14,13) R(15,n4) R(16,n6) R(17,0) R(17,2) R(18,0) R(18,19) R(19,3) R(19,18) R(19,22) R(20,3) R(20,21) R(21,4) R(21,20) R(22,19) R(22,23) R(23,5) R(23,22) R(24,0) R(24,8) R(25,0) R(25,26) R(26,25) R(26,27) R(27,11) R(27,26) R(28,0) R(28,29) R(29,28) R(29,30) R(30,29) R(30,31) R(31,12) R(31,30) R(n1,0) R(n1,n7) R(n2,0) R(n2,6) R(n3,6) R(n3,7) R(n4,13) R(n4,15) R(n4,n6) R(n5,13) R(n5,n7) R(n6,16) R(n6,n4) R(n7,n1) R(n7,n5)

-------------------------------------------------------------------------------------------------------------

-> form outgroup SOL1_ALTER - A

n3(mod4)

[ cox1 cox2 atp8 atp6 cox3 nad3 -nad5 -nad4 -nad4L nad6 cob rrnS rrnL nad1 nad2 ]

n4(mod4)

[ cox1 cox2 nad1 nad3 nad2 cox3 rrnS -nad4L -nad4 -nad5 -cob -nad6 rrnL ]

score = 265:

model 4:

-------------

R(0,1) R(0,9) R(0,10) R(0,17) R(0,18) R(0,24) R(0,25) R(0,28) R(0,n1) R(0,n2) R(1,0) R(2,17) R(3,19) R(3,20) R(4,21) R(5,23) R(6,n3) R(7,n3) R(8,24) R(9,0) R(10,0) R(11,27) R(12,31) R(13,14) R(13,n4) R(13,n5) R(14,13) R(15,n4) R(16,n6) R(17,0) R(17,2) R(18,0) R(18,19) R(19,3) R(19,18) R(19,22) R(20,3) R(20,21) R(21,4) R(21,20) R(22,19) R(22,23) R(23,5) R(23,22) R(24,0) R(24,8) R(25,0) R(25,26) R(26,25) R(26,27) R(27,11) R(27,26) R(28,0) R(28,29) R(29,28) R(29,30) R(30,29) R(30,31) R(31,12) R(31,30) R(n1,0) R(n1,n7) R(n2,0) R(n2,n3) R(n3,6) R(n3,7) R(n3,n2) R(n4,13) R(n4,15) R(n4,n6) R(n5,13) R(n5,n7) R(n6,16) R(n6,n4) R(n7,n1) R(n7,n5)

-------------------------------------------------------------------------------------------------------------

-> form outgroup SOL2_ALTER - A

n4(mod5)

[ cox1 cox2 nad1 nad3 nad2 cox3 rrnS -nad5 -nad4 -nad4L -cob -nad6 rrnL ]

score = 241:

model 5:

-------------

R(0,1) R(0,9) R(0,10) R(0,17) R(0,18) R(0,24) R(0,25) R(0,28) R(0,n1) R(0,n2) R(1,0) R(2,17) R(3,19) R(3,20) R(4,21) R(5,23) R(6,n1) R(6,n3) R(7,n3) R(8,24) R(9,0) R(10,0) R(11,27) R(12,31) R(13,14) R(13,n4) R(13,n5) R(14,13) R(15,n4) R(16,n7) R(17,0) R(17,2) R(18,0) R(18,19) R(19,3) R(19,18) R(19,22) R(20,3) R(20,21) R(21,4) R(21,20) R(22,19) R(22,23) R(23,5) R(23,22) R(24,0) R(24,8) R(25,0) R(25,26) R(26,25) R(26,27) R(27,11) R(27,26) R(28,0) R(28,29) R(29,28) R(29,30) R(30,29) R(30,31) R(31,12) R(31,30) R(n1,0) R(n1,6) R(n2,0) R(n2,n6) R(n3,6) R(n3,7) R(n4,13) R(n4,15) R(n4,n6) R(n5,13) R(n5,n7) R(n6,n2) R(n6,n4) R(n7,16) R(n7,n5)

-------------------------------------------------------------------------------------------------------------

-> form outgroup SOL1_ALTER - A

n3(mod6)

[ cox1 cox2 atp8 atp6 cox3 nad3 -nad5 -nad4 -nad4L nad6 cob rrnS rrnL nad1 nad2 ]

n4(mod6)

[ cox1 cox2 nad1 nad3 nad2 cox3 rrnS -nad5 -nad4 -nad4L -cob -nad6 rrnL ]

score = 241:

model 6:

-------------

R(0,1) R(0,9) R(0,10) R(0,17) R(0,18) R(0,24) R(0,25) R(0,28) R(0,n1) R(0,n2) R(1,0) R(2,17) R(3,19) R(3,20) R(4,21) R(5,23) R(6,n3) R(7,n3) R(8,24) R(9,0) R(10,0) R(11,27) R(12,31) R(13,14) R(13,n4) R(13,n5) R(14,13) R(15,n4) R(16,n7) R(17,0) R(17,2) R(18,0) R(18,19) R(19,3) R(19,18) R(19,22) R(20,3) R(20,21) R(21,4) R(21,20) R(22,19) R(22,23) R(23,5) R(23,22) R(24,0) R(24,8) R(25,0) R(25,26) R(26,25) R(26,27) R(27,11) R(27,26) R(28,0) R(28,29) R(29,28) R(29,30) R(30,29) R(30,31) R(31,12) R(31,30) R(n1,0) R(n1,n3) R(n2,0) R(n2,n6) R(n3,6) R(n3,7) R(n3,n1) R(n4,13) R(n4,15) R(n4,n6) R(n5,13) R(n5,n7) R(n6,n2) R(n6,n4) R(n7,16) R(n7,n5)

-------------------------------------------------------------------------------------------------------------

-> form outgroup SOL2_ALTER - A

n4(mod7)

g1: [ cox1 cox2 nad1 nad3 nad2 cox3 rrnS -nad4 -nad5 -cob -nad6 -nad4L rrnL ]

g2: [ cox1 cox2 nad1 nad3 nad2 cox3 rrnS -nad4 -nad5 -cob -nad6 rrnL -nad4L ]

g3: [ cox1 cox2 nad1 nad3 nad2 cox3 rrnS nad5 nad4 -nad4L -cob -nad6 rrnL ]

g4: [ cox1 cox2 nad1 nad3 nad2 cox3 rrnS rrnL nad6 cob nad4L nad5 nad4 ]

g5: [ cox1 cox2 nad1 nad3 nad2 cox3 rrnS -rrnL nad6 cob nad4L nad5 nad4 ]

g6: [ cox1 cox2 nad1 nad3 nad2 cox3 rrnS -nad4 -nad5 rrnL nad6 cob nad4L ]

g7: [ cox1 cox2 nad1 nad3 nad2 cox3 rrnS nad6 cob nad4L -nad4 -nad5 rrnL ]

g8: [ cox1 cox2 nad1 nad3 nad2 cox3 rrnS -nad4 -nad5 nad6 cob nad4L rrnL ]

g9: [ cox1 cox2 nad1 nad3 nad2 cox3 rrnS -rrnL nad6 cob nad4L -nad4 -nad5 ]

g10: [ cox1 cox2 nad1 nad3 nad2 cox3 rrnS -nad4 -nad5 -rrnL -nad4L -cob -nad6 ]

g11: [ cox1 cox2 nad1 nad3 nad2 cox3 rrnS -nad4 -nad5 -nad4L -cob -nad6 -rrnL ]

score = 277:

model 7:

-------------

R(0,1) R(0,9) R(0,10) R(0,17) R(0,18) R(0,24) R(0,25) R(0,28) R(0,n1) R(0,n2) R(1,0) R(2,17) R(3,19) R(3,20) R(4,21) R(5,23) R(6,n2) R(6,n3) R(7,n3) R(8,24) R(9,0) R(10,0) R(11,27) R(12,31) R(13,14) R(13,15) R(13,n4) R(14,13) R(15,13) R(16,n5) R(17,0) R(17,2) R(18,0) R(18,19) R(19,3) R(19,18) R(19,22) R(20,3) R(20,21) R(21,4) R(21,20) R(22,19) R(22,23) R(23,5) R(23,22) R(24,0) R(24,8) R(25,0) R(25,26) R(26,25) R(26,27) R(27,11) R(27,26) R(28,0) R(28,29) R(29,28) R(29,30) R(30,29) R(30,31) R(31,12) R(31,30) R(n1,0) R(n1,n7) R(n2,0) R(n2,6) R(n3,6) R(n3,7) R(n4,13) R(n4,n5) R(n4,n6) R(n5,16) R(n5,n4) R(n6,n4) R(n6,n7) R(n7,n1) R(n7,n6)

-------------------------------------------------------------------------------------------------------------

-> form outgroup SOL1_ALTER - A

n3(mod8)

[ cox1 cox2 atp8 atp6 cox3 nad3 -nad5 -nad4 -nad4L nad6 cob rrnS rrnL nad1 nad2 ]

n4(mod8)

g1: [ cox1 cox2 nad1 nad3 nad2 cox3 rrnS -nad4 -nad5 -cob -nad6 -nad4L rrnL ]

g2: [ cox1 cox2 nad1 nad3 nad2 cox3 rrnS -nad4 -nad5 -cob -nad6 rrnL -nad4L ]

g3: [ cox1 cox2 nad1 nad3 nad2 cox3 rrnS nad5 nad4 -nad4L -cob -nad6 rrnL ]

g4: [ cox1 cox2 nad1 nad3 nad2 cox3 rrnS rrnL nad6 cob nad4L nad5 nad4 ]

g5: [ cox1 cox2 nad1 nad3 nad2 cox3 rrnS -rrnL nad6 cob nad4L nad5 nad4 ]

g6: [ cox1 cox2 nad1 nad3 nad2 cox3 rrnS -nad4 -nad5 rrnL nad6 cob nad4L ]

g7: [ cox1 cox2 nad1 nad3 nad2 cox3 rrnS nad6 cob nad4L -nad4 -nad5 rrnL ]

g8: [ cox1 cox2 nad1 nad3 nad2 cox3 rrnS -nad4 -nad5 nad6 cob nad4L rrnL ]

g9: [ cox1 cox2 nad1 nad3 nad2 cox3 rrnS -rrnL nad6 cob nad4L -nad4 -nad5 ]

g10: [ cox1 cox2 nad1 nad3 nad2 cox3 rrnS -nad4 -nad5 -rrnL -nad4L -cob -nad6 ]

g11: [ cox1 cox2 nad1 nad3 nad2 cox3 rrnS -nad4 -nad5 -nad4L -cob -nad6 -rrnL ]

score = 277:

model 8:

-------------

R(0,1) R(0,9) R(0,10) R(0,17) R(0,18) R(0,24) R(0,25) R(0,28) R(0,n1) R(0,n2) R(1,0) R(2,17) R(3,19) R(3,20) R(4,21) R(5,23) R(6,n3) R(7,n3) R(8,24) R(9,0) R(10,0) R(11,27) R(12,31) R(13,14) R(13,15) R(13,n4) R(14,13) R(15,13) R(16,n5) R(17,0) R(17,2) R(18,0) R(18,19) R(19,3) R(19,18) R(19,22) R(20,3) R(20,21) R(21,4) R(21,20) R(22,19) R(22,23) R(23,5) R(23,22) R(24,0) R(24,8) R(25,0) R(25,26) R(26,25) R(26,27) R(27,11) R(27,26) R(28,0) R(28,29) R(29,28) R(29,30) R(30,29) R(30,31) R(31,12) R(31,30) R(n1,0) R(n1,n7) R(n2,0) R(n2,n3) R(n3,6) R(n3,7) R(n3,n2) R(n4,13) R(n4,n5) R(n4,n6) R(n5,16) R(n5,n4) R(n6,n4) R(n6,n7) R(n7,n1) R(n7,n6)

-------------------------------------------------------------------------------------------------------------

-> form outgroup SOL1_ALTER - A

n3(mod9)

[ cox1 cox2 atp8 atp6 cox3 nad3 -nad5 -nad4 -nad4L nad6 cob rrnS rrnL nad1 nad2 ]

score = 277:

model 9:

-------------

R(0,1) R(0,9) R(0,10) R(0,17) R(0,18) R(0,24) R(0,25) R(0,28) R(0,n1) R(0,n2) R(1,0) R(2,17) R(3,19) R(3,20) R(4,21) R(5,23) R(6,n3) R(7,n3) R(8,24) R(9,0) R(10,0) R(11,27) R(12,31) R(13,14) R(13,15) R(13,n4) R(14,13) R(15,13) R(15,n6) R(16,n5) R(17,0) R(17,2) R(18,0) R(18,19) R(19,3) R(19,18) R(19,22) R(20,3) R(20,21) R(21,4) R(21,20) R(22,19) R(22,23) R(23,5) R(23,22) R(24,0) R(24,8) R(25,0) R(25,26) R(26,25) R(26,27) R(27,11) R(27,26) R(28,0) R(28,29) R(29,28) R(29,30) R(30,29) R(30,31) R(31,12) R(31,30) R(n1,0) R(n1,n3) R(n2,0) R(n2,n7) R(n3,6) R(n3,7) R(n3,n1) R(n4,13) R(n4,n5) R(n5,16) R(n5,n4) R(n6,15) R(n6,n7) R(n7,n2) R(n7,n6)

-------------------------------------------------------------------------------------------------------------

-> form outgroup SOL2_ALTER - A

score = 277:

model 10:

-------------

R(0,1) R(0,9) R(0,10) R(0,17) R(0,18) R(0,24) R(0,25) R(0,28) R(0,n1) R(0,n2) R(1,0) R(2,17) R(3,19) R(3,20) R(4,21) R(5,23) R(6,n1) R(6,n3) R(7,n3) R(8,24) R(9,0) R(10,0) R(11,27) R(12,31) R(13,14) R(13,15) R(13,n4) R(14,13) R(15,13) R(15,n7) R(16,n5) R(17,0) R(17,2) R(18,0) R(18,19) R(19,3) R(19,18) R(19,22) R(20,3) R(20,21) R(21,4) R(21,20) R(22,19) R(22,23) R(23,5) R(23,22) R(24,0) R(24,8) R(25,0) R(25,26) R(26,25) R(26,27) R(27,11) R(27,26) R(28,0) R(28,29) R(29,28) R(29,30) R(30,29) R(30,31) R(31,12) R(31,30) R(n1,0) R(n1,6) R(n2,0) R(n2,n6) R(n3,6) R(n3,7) R(n4,13) R(n4,n5) R(n5,16) R(n5,n4) R(n6,n2) R(n6,n7) R(n7,15) R(n7,n6)

-------------------------------------------------------------------------------------------------------------

-> form outgroup SOL2_ALTER - A

score = 265:

model 11:

-------------

R(0,1) R(0,9) R(0,10) R(0,17) R(0,18) R(0,24) R(0,25) R(0,28) R(0,n1) R(0,n2) R(1,0) R(2,17) R(3,19) R(3,20) R(4,21) R(5,23) R(6,n1) R(6,n3) R(7,n3) R(8,24) R(9,0) R(10,0) R(11,27) R(12,31) R(13,14) R(13,15) R(13,n4) R(14,13) R(15,13) R(15,n7) R(16,n5) R(17,0) R(17,2) R(18,0) R(18,19) R(19,3) R(19,18) R(19,22) R(20,3) R(20,21) R(21,4) R(21,20) R(22,19) R(22,23) R(23,5) R(23,22) R(24,0) R(24,8) R(25,0) R(25,26) R(26,25) R(26,27) R(27,11) R(27,26) R(28,0) R(28,29) R(29,28) R(29,30) R(30,29) R(30,31) R(31,12) R(31,30) R(n1,0) R(n1,6) R(n2,0) R(n2,n6) R(n3,6) R(n3,7) R(n4,13) R(n4,n6) R(n5,16) R(n5,n7) R(n6,n2) R(n6,n4) R(n7,15) R(n7,n5)

-------------------------------------------------------------------------------------------------------------

-> form outgroup SOL2_ALTER - A

n4(mod12)

g1: [ cox1 cox2 -nad4 -nad5 -nad4L -cob -nad6 nad1 nad3 nad2 cox3 rrnS rrnL ]

g2: [ cox1 cox2 -nad4 -nad5 -nad4L -cob -nad6 rrnL nad1 nad3 nad2 cox3 rrnS ]

g3: [ cox1 cox2 nad1 nad3 nad2 -nad4 -nad5 -nad4L -cob -nad6 cox3 rrnS rrnL ]

g4: [ cox1 cox2 nad1 nad3 nad2 cox3 rrnS -nad5 -nad4 -nad4L -cob -nad6 rrnL ]

g5: [ cox1 cox2 nad1 nad6 cob nad4L nad5 nad4 nad3 nad2 cox3 rrnS rrnL ]

score = 241:

model 12:

-------------

R(0,1) R(0,9) R(0,10) R(0,17) R(0,18) R(0,24) R(0,25) R(0,28) R(0,n1) R(0,n2) R(1,0) R(2,17) R(3,19) R(3,20) R(4,21) R(5,23) R(6,n1) R(6,n3) R(7,n3) R(8,24) R(9,0) R(10,0) R(11,27) R(12,31) R(13,14) R(13,15) R(13,n4) R(14,13) R(15,13) R(16,n5) R(17,0) R(17,2) R(18,0) R(18,19) R(19,3) R(19,18) R(19,22) R(20,3) R(20,21) R(21,4) R(21,20) R(22,19) R(22,23) R(23,5) R(23,22) R(24,0) R(24,8) R(25,0) R(25,26) R(26,25) R(26,27) R(27,11) R(27,26) R(28,0) R(28,29) R(29,28) R(29,30) R(30,29) R(30,31) R(31,12) R(31,30) R(n1,0) R(n1,6) R(n2,0) R(n2,n6) R(n3,6) R(n3,7) R(n4,13) R(n4,n6) R(n4,n7) R(n5,16) R(n5,n7) R(n6,n2) R(n6,n4) R(n7,n4) R(n7,n5)

-------------------------------------------------------------------------------------------------------------

-> form outgroup SOL1_ALTER - A

n3(mod13)

[ cox1 cox2 atp8 atp6 cox3 nad3 -nad5 -nad4 -nad4L nad6 cob rrnS rrnL nad1 nad2 ]

score = 265:

model 13:

-------------

R(0,1) R(0,9) R(0,10) R(0,17) R(0,18) R(0,24) R(0,25) R(0,28) R(0,n1) R(0,n2) R(1,0) R(2,17) R(3,19) R(3,20) R(4,21) R(5,23) R(6,n3) R(7,n3) R(8,24) R(9,0) R(10,0) R(11,27) R(12,31) R(13,14) R(13,15) R(13,n4) R(14,13) R(15,13) R(15,n6) R(16,n5) R(17,0) R(17,2) R(18,0) R(18,19) R(19,3) R(19,18) R(19,22) R(20,3) R(20,21) R(21,4) R(21,20) R(22,19) R(22,23) R(23,5) R(23,22) R(24,0) R(24,8) R(25,0) R(25,26) R(26,25) R(26,27) R(27,11) R(27,26) R(28,0) R(28,29) R(29,28) R(29,30) R(30,29) R(30,31) R(31,12) R(31,30) R(n1,0) R(n1,n7) R(n2,0) R(n2,n3) R(n3,6) R(n3,7) R(n3,n2) R(n4,13) R(n4,n7) R(n5,16) R(n5,n6) R(n6,15) R(n6,n5) R(n7,n1) R(n7,n4)

-------------------------------------------------------------------------------------------------------------

-> form outgroup SOL1_ALTER - A

n3(mod14)

[ cox1 cox2 atp8 atp6 cox3 nad3 -nad5 -nad4 -nad4L nad6 cob rrnS rrnL nad1 nad2 ]

n4(mod14)

g1: [ cox1 cox2 -nad4 -nad5 -nad4L -cob -nad6 nad1 nad3 nad2 cox3 rrnS rrnL ]

g2: [ cox1 cox2 -nad4 -nad5 -nad4L -cob -nad6 rrnL nad1 nad3 nad2 cox3 rrnS ]

g3: [ cox1 cox2 nad1 nad3 nad2 -nad4 -nad5 -nad4L -cob -nad6 cox3 rrnS rrnL ]

g4: [ cox1 cox2 nad1 nad3 nad2 cox3 rrnS -nad5 -nad4 -nad4L -cob -nad6 rrnL ]

g5: [ cox1 cox2 nad1 nad6 cob nad4L nad5 nad4 nad3 nad2 cox3 rrnS rrnL ]

score = 241:

model 14:

-------------

R(0,1) R(0,9) R(0,10) R(0,17) R(0,18) R(0,24) R(0,25) R(0,28) R(0,n1) R(0,n2) R(1,0) R(2,17) R(3,19) R(3,20) R(4,21) R(5,23) R(6,n3) R(7,n3) R(8,24) R(9,0) R(10,0) R(11,27) R(12,31) R(13,14) R(13,15) R(13,n4) R(14,13) R(15,13) R(16,n5) R(17,0) R(17,2) R(18,0) R(18,19) R(19,3) R(19,18) R(19,22) R(20,3) R(20,21) R(21,4) R(21,20) R(22,19) R(22,23) R(23,5) R(23,22) R(24,0) R(24,8) R(25,0) R(25,26) R(26,25) R(26,27) R(27,11) R(27,26) R(28,0) R(28,29) R(29,28) R(29,30) R(30,29) R(30,31) R(31,12) R(31,30) R(n1,0) R(n1,n3) R(n2,0) R(n2,n7) R(n3,6) R(n3,7) R(n3,n1) R(n4,13) R(n4,n6) R(n4,n7) R(n5,16) R(n5,n6) R(n6,n4) R(n6,n5) R(n7,n2) R(n7,n4)

-------------------------------------------------------------------------------------------------------------

-> form outgroup SOL2_ALTER - A

n4(mod15)

[ cox1 cox2 nad1 nad3 nad2 cox3 rrnS -nad4L -nad4 -nad5 -cob -nad6 rrnL ]

score = 291:

model 15:

-------------

R(0,1) R(0,9) R(0,10) R(0,17) R(0,18) R(0,24) R(0,25) R(0,28) R(0,n1) R(0,n2) R(1,0) R(2,17) R(3,19) R(3,20) R(4,21) R(5,23) R(6,n2) R(6,n3) R(7,n3) R(8,24) R(9,0) R(10,0) R(11,27) R(12,31) R(13,14) R(13,n4) R(14,13) R(15,n4) R(15,n5) R(16,n6) R(17,0) R(17,2) R(18,0) R(18,19) R(19,3) R(19,18) R(19,22) R(20,3) R(20,21) R(21,4) R(21,20) R(22,19) R(22,23) R(23,5) R(23,22) R(24,0) R(24,8) R(25,0) R(25,26) R(26,25) R(26,27) R(27,11) R(27,26) R(28,0) R(28,29) R(29,28) R(29,30) R(30,29) R(30,31) R(31,12) R(31,30) R(n1,0) R(n1,n7) R(n2,0) R(n2,6) R(n3,6) R(n3,7) R(n4,13) R(n4,15) R(n4,n6) R(n5,15) R(n5,n7) R(n6,16) R(n6,n4) R(n7,n1) R(n7,n5)

-------------------------------------------------------------------------------------------------------------

-> form outgroup SOL1_ALTER - A

n3(mod16)

[ cox1 cox2 atp8 atp6 cox3 nad3 -nad5 -nad4 -nad4L nad6 cob rrnS rrnL nad1 nad2 ]

n4(mod16)

[ cox1 cox2 nad1 nad3 nad2 cox3 rrnS -nad4L -nad4 -nad5 -cob -nad6 rrnL ]

score = 291:

model 16:

-------------

R(0,1) R(0,9) R(0,10) R(0,17) R(0,18) R(0,24) R(0,25) R(0,28) R(0,n1) R(0,n2) R(1,0) R(2,17) R(3,19) R(3,20) R(4,21) R(5,23) R(6,n3) R(7,n3) R(8,24) R(9,0) R(10,0) R(11,27) R(12,31) R(13,14) R(13,n4) R(14,13) R(15,n4) R(15,n5) R(16,n6) R(17,0) R(17,2) R(18,0) R(18,19) R(19,3) R(19,18) R(19,22) R(20,3) R(20,21) R(21,4) R(21,20) R(22,19) R(22,23) R(23,5) R(23,22) R(24,0) R(24,8) R(25,0) R(25,26) R(26,25) R(26,27) R(27,11) R(27,26) R(28,0) R(28,29) R(29,28) R(29,30) R(30,29) R(30,31) R(31,12) R(31,30) R(n1,0) R(n1,n7) R(n2,0) R(n2,n3) R(n3,6) R(n3,7) R(n3,n2) R(n4,13) R(n4,15) R(n4,n6) R(n5,15) R(n5,n7) R(n6,16) R(n6,n4) R(n7,n1) R(n7,n5)

-------------------------------------------------------------------------------------------------------------

-> form outgroup SOL2_ALTER - A

n4(mod17)

[ cox1 cox2 nad1 nad3 nad2 cox3 rrnS -nad5 -nad4 -nad4L -cob -nad6 rrnL ]

score = 243:

model 17:

-------------

R(0,1) R(0,9) R(0,10) R(0,17) R(0,18) R(0,24) R(0,25) R(0,28) R(0,n1) R(0,n2) R(1,0) R(2,17) R(3,19) R(3,20) R(4,21) R(5,23) R(6,n1) R(6,n3) R(7,n3) R(8,24) R(9,0) R(10,0) R(11,27) R(12,31) R(13,14) R(13,n4) R(14,13) R(15,n4) R(15,n5) R(16,n7) R(17,0) R(17,2) R(18,0) R(18,19) R(19,3) R(19,18) R(19,22) R(20,3) R(20,21) R(21,4) R(21,20) R(22,19) R(22,23) R(23,5) R(23,22) R(24,0) R(24,8) R(25,0) R(25,26) R(26,25) R(26,27) R(27,11) R(27,26) R(28,0) R(28,29) R(29,28) R(29,30) R(30,29) R(30,31) R(31,12) R(31,30) R(n1,0) R(n1,6) R(n2,0) R(n2,n6) R(n3,6) R(n3,7) R(n4,13) R(n4,15) R(n4,n6) R(n5,15) R(n5,n7) R(n6,n2) R(n6,n4) R(n7,16) R(n7,n5)

-------------------------------------------------------------------------------------------------------------

-> form outgroup SOL1_ALTER - A

n3(mod18)

[ cox1 cox2 atp8 atp6 cox3 nad3 -nad5 -nad4 -nad4L nad6 cob rrnS rrnL nad1 nad2 ]

n4(mod18)

[ cox1 cox2 nad1 nad3 nad2 cox3 rrnS -nad5 -nad4 -nad4L -cob -nad6 rrnL ]

score = 243:

model 18:

-------------

R(0,1) R(0,9) R(0,10) R(0,17) R(0,18) R(0,24) R(0,25) R(0,28) R(0,n1) R(0,n2) R(1,0) R(2,17) R(3,19) R(3,20) R(4,21) R(5,23) R(6,n3) R(7,n3) R(8,24) R(9,0) R(10,0) R(11,27) R(12,31) R(13,14) R(13,n4) R(14,13) R(15,n4) R(15,n5) R(16,n7) R(17,0) R(17,2) R(18,0) R(18,19) R(19,3) R(19,18) R(19,22) R(20,3) R(20,21) R(21,4) R(21,20) R(22,19) R(22,23) R(23,5) R(23,22) R(24,0) R(24,8) R(25,0) R(25,26) R(26,25) R(26,27) R(27,11) R(27,26) R(28,0) R(28,29) R(29,28) R(29,30) R(30,29) R(30,31) R(31,12) R(31,30) R(n1,0) R(n1,n3) R(n2,0) R(n2,n6) R(n3,6) R(n3,7) R(n3,n1) R(n4,13) R(n4,15) R(n4,n6) R(n5,15) R(n5,n7) R(n6,n2) R(n6,n4) R(n7,16) R(n7,n5)

-------------------------------------------------------------------------------------------------------------

-> form outgroup SOL2_ALTER - A

n4(mod19)

[ cox1 cox2 nad1 nad3 nad2 cox3 rrnS -nad5 -nad4 -nad4L -cob -nad6 rrnL ]

score = 229:

model 19:

-------------

R(0,1) R(0,9) R(0,10) R(0,17) R(0,18) R(0,24) R(0,25) R(0,28) R(0,n1) R(0,n2) R(1,0) R(2,17) R(3,19) R(3,20) R(4,21) R(5,23) R(6,n1) R(6,n3) R(7,n3) R(8,24) R(9,0) R(10,0) R(11,27) R(12,31) R(13,14) R(13,n4) R(14,13) R(15,n4) R(16,n5) R(17,0) R(17,2) R(18,0) R(18,19) R(19,3) R(19,18) R(19,22) R(20,3) R(20,21) R(21,4) R(21,20) R(22,19) R(22,23) R(23,5) R(23,22) R(24,0) R(24,8) R(25,0) R(25,26) R(26,25) R(26,27) R(27,11) R(27,26) R(28,0) R(28,29) R(29,28) R(29,30) R(30,29) R(30,31) R(31,12) R(31,30) R(n1,0) R(n1,6) R(n2,0) R(n2,n6) R(n3,6) R(n3,7) R(n4,13) R(n4,15) R(n4,n6) R(n4,n7) R(n5,16) R(n5,n7) R(n6,n2) R(n6,n4) R(n7,n4) R(n7,n5)

-------------------------------------------------------------------------------------------------------------

-> form outgroup SOL1_ALTER - A

n3(mod20)

[ cox1 cox2 atp8 atp6 cox3 nad3 -nad5 -nad4 -nad4L nad6 cob rrnS rrnL nad1 nad2 ]

n4(mod20)

[ cox1 cox2 nad1 nad3 nad2 cox3 rrnS -nad5 -nad4 -nad4L -cob -nad6 rrnL ]

score = 229:

model 20:

-------------

R(0,1) R(0,9) R(0,10) R(0,17) R(0,18) R(0,24) R(0,25) R(0,28) R(0,n1) R(0,n2) R(1,0) R(2,17) R(3,19) R(3,20) R(4,21) R(5,23) R(6,n3) R(7,n3) R(8,24) R(9,0) R(10,0) R(11,27) R(12,31) R(13,14) R(13,n4) R(14,13) R(15,n4) R(16,n5) R(17,0) R(17,2) R(18,0) R(18,19) R(19,3) R(19,18) R(19,22) R(20,3) R(20,21) R(21,4) R(21,20) R(22,19) R(22,23) R(23,5) R(23,22) R(24,0) R(24,8) R(25,0) R(25,26) R(26,25) R(26,27) R(27,11) R(27,26) R(28,0) R(28,29) R(29,28) R(29,30) R(30,29) R(30,31) R(31,12) R(31,30) R(n1,0) R(n1,n3) R(n2,0) R(n2,n6) R(n3,6) R(n3,7) R(n3,n1) R(n4,13) R(n4,15) R(n4,n6) R(n4,n7) R(n5,16) R(n5,n7) R(n6,n2) R(n6,n4) R(n7,n4) R(n7,n5)

-------------------------------------------------------------------------------------------------------------

-> form outgroup SOL2_ALTER - A

n5(mod21)

[ cox1 cox2 nad1 nad3 nad2 cox3 rrnS -nad5 -nad4 -nad4L -cob -nad6 rrnL ]

n4(mod21)

[ cox1 cox2 nad1 nad3 nad2 cox3 rrnS nad5 nad4 -nad4L -cob -nad6 rrnL ]

score = 255:

model 21:

-------------

R(0,1) R(0,9) R(0,10) R(0,17) R(0,18) R(0,24) R(0,25) R(0,28) R(0,n1) R(0,n2) R(1,0) R(2,17) R(3,19) R(3,20) R(4,21) R(5,23) R(6,n2) R(6,n3) R(7,n3) R(8,24) R(9,0) R(10,0) R(11,27) R(12,31) R(13,14) R(13,n4) R(14,13) R(15,n5) R(16,n6) R(17,0) R(17,2) R(18,0) R(18,19) R(19,3) R(19,18) R(19,22) R(20,3) R(20,21) R(21,4) R(21,20) R(22,19) R(22,23) R(23,5) R(23,22) R(24,0) R(24,8) R(25,0) R(25,26) R(26,25) R(26,27) R(27,11) R(27,26) R(28,0) R(28,29) R(29,28) R(29,30) R(30,29) R(30,31) R(31,12) R(31,30) R(n1,0) R(n1,n7) R(n2,0) R(n2,6) R(n3,6) R(n3,7) R(n4,13) R(n4,n5) R(n4,n6) R(n5,15) R(n5,n4) R(n5,n7) R(n6,16) R(n6,n4) R(n7,n1) R(n7,n5)

-------------------------------------------------------------------------------------------------------------

-> form outgroup SOL1_ALTER - A

n3(mod22)

[ cox1 cox2 atp8 atp6 cox3 nad3 -nad5 -nad4 -nad4L nad6 cob rrnS rrnL nad1 nad2 ]

n5(mod22)

[ cox1 cox2 nad1 nad3 nad2 cox3 rrnS -nad5 -nad4 -nad4L -cob -nad6 rrnL ]

n4(mod22)

[ cox1 cox2 nad1 nad3 nad2 cox3 rrnS nad5 nad4 -nad4L -cob -nad6 rrnL ]

score = 255:

model 22:

-------------

R(0,1) R(0,9) R(0,10) R(0,17) R(0,18) R(0,24) R(0,25) R(0,28) R(0,n1) R(0,n2) R(1,0) R(2,17) R(3,19) R(3,20) R(4,21) R(5,23) R(6,n3) R(7,n3) R(8,24) R(9,0) R(10,0) R(11,27) R(12,31) R(13,14) R(13,n4) R(14,13) R(15,n5) R(16,n6) R(17,0) R(17,2) R(18,0) R(18,19) R(19,3) R(19,18) R(19,22) R(20,3) R(20,21) R(21,4) R(21,20) R(22,19) R(22,23) R(23,5) R(23,22) R(24,0) R(24,8) R(25,0) R(25,26) R(26,25) R(26,27) R(27,11) R(27,26) R(28,0) R(28,29) R(29,28) R(29,30) R(30,29) R(30,31) R(31,12) R(31,30) R(n1,0) R(n1,n7) R(n2,0) R(n2,n3) R(n3,6) R(n3,7) R(n3,n2) R(n4,13) R(n4,n5) R(n4,n6) R(n5,15) R(n5,n4) R(n5,n7) R(n6,16) R(n6,n4) R(n7,n1) R(n7,n5)

-------------------------------------------------------------------------------------------------------------

-> form outgroup SOL2_ALTER - A

score = 265:

model 23:

-------------

R(0,1) R(0,9) R(0,10) R(0,17) R(0,18) R(0,24) R(0,25) R(0,28) R(0,n1) R(0,n2) R(1,0) R(2,17) R(3,19) R(3,20) R(4,21) R(5,23) R(6,n2) R(6,n3) R(7,n3) R(8,24) R(9,0) R(10,0) R(11,27) R(12,31) R(13,14) R(13,15) R(14,13) R(15,13) R(15,n4) R(15,n5) R(16,n6) R(17,0) R(17,2) R(18,0) R(18,19) R(19,3) R(19,18) R(19,22) R(20,3) R(20,21) R(21,4) R(21,20) R(22,19) R(22,23) R(23,5) R(23,22) R(24,0) R(24,8) R(25,0) R(25,26) R(26,25) R(26,27) R(27,11) R(27,26) R(28,0) R(28,29) R(29,28) R(29,30) R(30,29) R(30,31) R(31,12) R(31,30) R(n1,0) R(n1,n7) R(n2,0) R(n2,6) R(n3,6) R(n3,7) R(n4,15) R(n4,n6) R(n5,15) R(n5,n7) R(n6,16) R(n6,n4) R(n7,n1) R(n7,n5)

-------------------------------------------------------------------------------------------------------------

-> form outgroup SOL1_ALTER - A

n3(mod24)

[ cox1 cox2 atp8 atp6 cox3 nad3 -nad5 -nad4 -nad4L nad6 cob rrnS rrnL nad1 nad2 ]

score = 265:

model 24:

-------------

R(0,1) R(0,9) R(0,10) R(0,17) R(0,18) R(0,24) R(0,25) R(0,28) R(0,n1) R(0,n2) R(1,0) R(2,17) R(3,19) R(3,20) R(4,21) R(5,23) R(6,n3) R(7,n3) R(8,24) R(9,0) R(10,0) R(11,27) R(12,31) R(13,14) R(13,15) R(14,13) R(15,13) R(15,n4) R(15,n5) R(16,n6) R(17,0) R(17,2) R(18,0) R(18,19) R(19,3) R(19,18) R(19,22) R(20,3) R(20,21) R(21,4) R(21,20) R(22,19) R(22,23) R(23,5) R(23,22) R(24,0) R(24,8) R(25,0) R(25,26) R(26,25) R(26,27) R(27,11) R(27,26) R(28,0) R(28,29) R(29,28) R(29,30) R(30,29) R(30,31) R(31,12) R(31,30) R(n1,0) R(n1,n7) R(n2,0) R(n2,n3) R(n3,6) R(n3,7) R(n3,n2) R(n4,15) R(n4,n6) R(n5,15) R(n5,n7) R(n6,16) R(n6,n4) R(n7,n1) R(n7,n5)

-------------------------------------------------------------------------------------------------------------

-> form outgroup SOL2_ALTER - A

n4(mod25)

[ cox1 cox2 nad1 nad3 nad2 cox3 rrnS -nad5 -nad4 -nad4L -cob -nad6 rrnL ]

score = 255:

model 25:

-------------

R(0,1) R(0,9) R(0,10) R(0,17) R(0,18) R(0,24) R(0,25) R(0,28) R(0,n1) R(0,n2) R(1,0) R(2,17) R(3,19) R(3,20) R(4,21) R(5,23) R(6,n1) R(6,n3) R(7,n3) R(8,24) R(9,0) R(10,0) R(11,27) R(12,31) R(13,14) R(13,15) R(14,13) R(15,13) R(15,n4) R(16,n5) R(17,0) R(17,2) R(18,0) R(18,19) R(19,3) R(19,18) R(19,22) R(20,3) R(20,21) R(21,4) R(21,20) R(22,19) R(22,23) R(23,5) R(23,22) R(24,0) R(24,8) R(25,0) R(25,26) R(26,25) R(26,27) R(27,11) R(27,26) R(28,0) R(28,29) R(29,28) R(29,30) R(30,29) R(30,31) R(31,12) R(31,30) R(n1,0) R(n1,6) R(n2,0) R(n2,n6) R(n3,6) R(n3,7) R(n4,15) R(n4,n6) R(n4,n7) R(n5,16) R(n5,n7) R(n6,n2) R(n6,n4) R(n7,n4) R(n7,n5)

-------------------------------------------------------------------------------------------------------------

-> form outgroup SOL1_ALTER - A

n3(mod26)

[ cox1 cox2 atp8 atp6 cox3 nad3 -nad5 -nad4 -nad4L nad6 cob rrnS rrnL nad1 nad2 ]

n4(mod26)

[ cox1 cox2 nad1 nad3 nad2 cox3 rrnS -nad5 -nad4 -nad4L -cob -nad6 rrnL ]

score = 255:

model 26:

-------------

R(0,1) R(0,9) R(0,10) R(0,17) R(0,18) R(0,24) R(0,25) R(0,28) R(0,n1) R(0,n2) R(1,0) R(2,17) R(3,19) R(3,20) R(4,21) R(5,23) R(6,n3) R(7,n3) R(8,24) R(9,0) R(10,0) R(11,27) R(12,31) R(13,14) R(13,15) R(14,13) R(15,13) R(15,n4) R(16,n5) R(17,0) R(17,2) R(18,0) R(18,19) R(19,3) R(19,18) R(19,22) R(20,3) R(20,21) R(21,4) R(21,20) R(22,19) R(22,23) R(23,5) R(23,22) R(24,0) R(24,8) R(25,0) R(25,26) R(26,25) R(26,27) R(27,11) R(27,26) R(28,0) R(28,29) R(29,28) R(29,30) R(30,29) R(30,31) R(31,12) R(31,30) R(n1,0) R(n1,n3) R(n2,0) R(n2,n6) R(n3,6) R(n3,7) R(n3,n1) R(n4,15) R(n4,n6) R(n4,n7) R(n5,16) R(n5,n7) R(n6,n2) R(n6,n4) R(n7,n4) R(n7,n5)

-------------------------------------------------------------------------------------------------------------

-> form outgroup SOL4_ALTER - A

n4(mod27)

[ cox1 cox2 atp8 atp6 cox3 nad3 -nad5 -nad4 -nad4L nad6 cob rrnS rrnL nad1 nad2 ]

n1(mod27)15g

[ cox1 cox2 atp8 atp6 cox3 rrnS rrnL nad1 nad6 cob nad4L nad4 nad5 nad3 nad2 ]

n1(mod27)13g

[ cox1 cox2 cox3 rrnS rrnL nad1 nad6 cob nad4L nad4 nad5 nad3 nad2 ]

score = 279:

model 27:

-------------

R(0,1) R(0,9) R(0,10) R(0,17) R(0,18) R(0,24) R(0,25) R(0,28) R(0,n1) R(1,0) R(2,17) R(3,19) R(3,20) R(4,21) R(5,23) R(6,n4) R(7,n4) R(8,24) R(9,0) R(10,0) R(11,27) R(12,31) R(13,14) R(13,15) R(14,13) R(15,13) R(15,n6) R(15,n7) R(16,n5) R(17,0) R(17,2) R(18,0) R(18,19) R(19,3) R(19,18) R(19,22) R(20,3) R(20,21) R(21,4) R(21,20) R(22,19) R(22,23) R(23,5) R(23,22) R(24,0) R(24,8) R(25,0) R(25,26) R(26,25) R(26,27) R(27,11) R(27,26) R(28,0) R(28,29) R(29,28) R(29,30) R(30,29) R(30,31) R(31,12) R(31,30) R(n1,0) R(n1,n2) R(n1,n3) R(n2,n1) R(n2,n4) R(n3,n1) R(n3,n6) R(n4,6) R(n4,7) R(n4,n2) R(n5,16) R(n5,n7) R(n6,15) R(n6,n3) R(n7,15) R(n7,n5)

-------------------------------------------------------------------------------------------------------------

-> form outgroup SOL4_ALTER - A

n4(mod28)

[ cox1 cox2 atp8 atp6 cox3 nad3 -nad5 -nad4 -nad4L nad6 cob rrnS rrnL nad1 nad2 ]

n1(mod28)15g

[ cox1 cox2 atp8 atp6 cox3 rrnS rrnL nad1 nad6 cob nad4L nad4 nad5 nad3 nad2 ]

n1(mod28)13g

[ cox1 cox2 cox3 rrnS rrnL nad1 nad6 cob nad4L nad4 nad5 nad3 nad2 ]

n6(mod28)

[ cox1 cox2 nad1 nad3 nad2 cox3 rrnS -nad5 -nad4 -nad4L -cob -nad6 rrnL ]

score = 269:

model 28:

-------------

R(0,1) R(0,9) R(0,10) R(0,17) R(0,18) R(0,24) R(0,25) R(0,28) R(0,n1) R(1,0) R(2,17) R(3,19) R(3,20) R(4,21) R(5,23) R(6,n4) R(7,n4) R(8,24) R(9,0) R(10,0) R(11,27) R(12,31) R(13,14) R(13,15) R(14,13) R(15,13) R(15,n6) R(16,n5) R(17,0) R(17,2) R(18,0) R(18,19) R(19,3) R(19,18) R(19,22) R(20,3) R(20,21) R(21,4) R(21,20) R(22,19) R(22,23) R(23,5) R(23,22) R(24,0) R(24,8) R(25,0) R(25,26) R(26,25) R(26,27) R(27,11) R(27,26) R(28,0) R(28,29) R(29,28) R(29,30) R(30,29) R(30,31) R(31,12) R(31,30) R(n1,0) R(n1,n2) R(n1,n3) R(n2,n1) R(n2,n4) R(n3,n1) R(n3,n6) R(n4,6) R(n4,7) R(n4,n2) R(n5,16) R(n5,n7) R(n6,15) R(n6,n3) R(n6,n7) R(n7,n5) R(n7,n6)

-------------------------------------------------------------------------------------------------------------

-> form outgroup SOL4_ALTER - A

n4(mod29)

[ cox1 cox2 atp8 atp6 cox3 nad3 -nad5 -nad4 -nad4L nad6 cob rrnS rrnL nad1 nad2 ]

n1(mod29)15g

[ cox1 cox2 atp8 atp6 cox3 rrnS rrnL nad1 nad6 cob nad4L nad4 nad5 nad3 nad2 ]

n1(mod29)13g

[ cox1 cox2 cox3 rrnS rrnL nad1 nad6 cob nad4L nad4 nad5 nad3 nad2 ]

score = 279:

model 29:

-------------

R(0,1) R(0,9) R(0,10) R(0,17) R(0,18) R(0,24) R(0,25) R(0,28) R(0,n1) R(1,0) R(2,17) R(3,19) R(3,20) R(4,21) R(5,23) R(6,n4) R(7,n4) R(8,24) R(9,0) R(10,0) R(11,27) R(12,31) R(13,14) R(13,15) R(13,n7) R(14,13) R(15,13) R(15,n6) R(16,n5) R(17,0) R(17,2) R(18,0) R(18,19) R(19,3) R(19,18) R(19,22) R(20,3) R(20,21) R(21,4) R(21,20) R(22,19) R(22,23) R(23,5) R(23,22) R(24,0) R(24,8) R(25,0) R(25,26) R(26,25) R(26,27) R(27,11) R(27,26) R(28,0) R(28,29) R(29,28) R(29,30) R(30,29) R(30,31) R(31,12) R(31,30) R(n1,0) R(n1,n2) R(n1,n3) R(n2,n1) R(n2,n4) R(n3,n1) R(n3,n7) R(n4,6) R(n4,7) R(n4,n2) R(n5,16) R(n5,n6) R(n6,15) R(n6,n5) R(n7,13) R(n7,n3)

-------------------------------------------------------------------------------------------------------------

-> form outgroup SOL4_ALTER - A

n4(mod30)

[ cox1 cox2 atp8 atp6 cox3 nad3 -nad5 -nad4 -nad4L nad6 cob rrnS rrnL nad1 nad2 ]

n1(mod30)15g

[ cox1 cox2 atp8 atp6 cox3 rrnS rrnL nad1 nad6 cob nad4L nad4 nad5 nad3 nad2 ]

n1(mod30)13g

[ cox1 cox2 cox3 rrnS rrnL nad1 nad6 cob nad4L nad4 nad5 nad3 nad2 ]

score = 291:

model 30:

-------------

R(0,1) R(0,9) R(0,10) R(0,17) R(0,18) R(0,24) R(0,25) R(0,28) R(0,n1) R(1,0) R(2,17) R(3,19) R(3,20) R(4,21) R(5,23) R(6,n4) R(7,n4) R(8,24) R(9,0) R(10,0) R(11,27) R(12,31) R(13,14) R(13,15) R(13,n7) R(14,13) R(15,13) R(15,n6) R(16,n5) R(17,0) R(17,2) R(18,0) R(18,19) R(19,3) R(19,18) R(19,22) R(20,3) R(20,21) R(21,4) R(21,20) R(22,19) R(22,23) R(23,5) R(23,22) R(24,0) R(24,8) R(25,0) R(25,26) R(26,25) R(26,27) R(27,11) R(27,26) R(28,0) R(28,29) R(29,28) R(29,30) R(30,29) R(30,31) R(31,12) R(31,30) R(n1,0) R(n1,n2) R(n1,n3) R(n2,n1) R(n2,n4) R(n3,n1) R(n3,n6) R(n4,6) R(n4,7) R(n4,n2) R(n5,16) R(n5,n7) R(n6,15) R(n6,n3) R(n7,13) R(n7,n5)

-------------------------------------------------------------------------------------------------------------

-> form outgroup SOL4_ALTER - A

n4(mod31)

[ cox1 cox2 atp8 atp6 cox3 nad3 -nad5 -nad4 -nad4L nad6 cob rrnS rrnL nad1 nad2 ]

n1(mod31)15g

[ cox1 cox2 atp8 atp6 cox3 rrnS rrnL nad1 nad6 cob nad4L nad4 nad5 nad3 nad2 ]

n1(mod31)13g

[ cox1 cox2 cox3 rrnS rrnL nad1 nad6 cob nad4L nad4 nad5 nad3 nad2 ]

score = 265:

model 31:

-------------

R(0,1) R(0,9) R(0,10) R(0,17) R(0,18) R(0,24) R(0,25) R(0,28) R(0,n1) R(1,0) R(2,17) R(3,19) R(3,20) R(4,21) R(5,23) R(6,n4) R(7,n4) R(8,24) R(9,0) R(10,0) R(11,27) R(12,31) R(13,14) R(13,15) R(13,n6) R(13,n7) R(14,13) R(15,13) R(16,n5) R(17,0) R(17,2) R(18,0) R(18,19) R(19,3) R(19,18) R(19,22) R(20,3) R(20,21) R(21,4) R(21,20) R(22,19) R(22,23) R(23,5) R(23,22) R(24,0) R(24,8) R(25,0) R(25,26) R(26,25) R(26,27) R(27,11) R(27,26) R(28,0) R(28,29) R(29,28) R(29,30) R(30,29) R(30,31) R(31,12) R(31,30) R(n1,0) R(n1,n2) R(n1,n3) R(n2,n1) R(n2,n4) R(n3,n1) R(n3,n6) R(n4,6) R(n4,7) R(n4,n2) R(n5,16) R(n5,n7) R(n6,13) R(n6,n3) R(n7,13) R(n7,n5)

-------------------------------------------------------------------------------------------------------------

-> form outgroup SOL4_ALTER - A

n4(mod32)

[ cox1 cox2 atp8 atp6 cox3 nad3 -nad5 -nad4 -nad4L nad6 cob rrnS rrnL nad1 nad2 ]

n1(mod32)15g

[ cox1 cox2 atp8 atp6 cox3 rrnS rrnL nad1 nad6 cob nad4L nad4 nad5 nad3 nad2 ]

n1(mod32)13g

[ cox1 cox2 cox3 rrnS rrnL nad1 nad6 cob nad4L nad4 nad5 nad3 nad2 ]

n6(mod32)

g1: [ cox1 cox2 nad1 nad3 nad2 cox3 rrnS -nad5 -nad4 -nad4L -cob -nad6 rrnL ]

g2: [ cox1 cox2 nad1 nad6 cob nad4L nad5 nad4 nad3 nad2 cox3 rrnS rrnL ]

score = 255:

model 32:

-------------

R(0,1) R(0,9) R(0,10) R(0,17) R(0,18) R(0,24) R(0,25) R(0,28) R(0,n1) R(1,0) R(2,17) R(3,19) R(3,20) R(4,21) R(5,23) R(6,n4) R(7,n4) R(8,24) R(9,0) R(10,0) R(11,27) R(12,31) R(13,14) R(13,15) R(13,n6) R(14,13) R(15,13) R(16,n5) R(17,0) R(17,2) R(18,0) R(18,19) R(19,3) R(19,18) R(19,22) R(20,3) R(20,21) R(21,4) R(21,20) R(22,19) R(22,23) R(23,5) R(23,22) R(24,0) R(24,8) R(25,0) R(25,26) R(26,25) R(26,27) R(27,11) R(27,26) R(28,0) R(28,29) R(29,28) R(29,30) R(30,29) R(30,31) R(31,12) R(31,30) R(n1,0) R(n1,n2) R(n1,n3) R(n2,n1) R(n2,n4) R(n3,n1) R(n3,n6) R(n4,6) R(n4,7) R(n4,n2) R(n5,16) R(n5,n7) R(n6,13) R(n6,n3) R(n6,n7) R(n7,n5) R(n7,n6)

-------------------------------------------------------------------------------------------------------------

-> form outgroup SOL4_ALTER - A

n4(mod33)

[ cox1 cox2 atp8 atp6 cox3 nad3 -nad5 -nad4 -nad4L nad6 cob rrnS rrnL nad1 nad2 ]

n6(mod33)

g1: [ cox1 cox2 nad1 nad3 nad2 cox3 rrnS -nad4 -nad5 -cob -nad6 -nad4L rrnL ]

g2: [ cox1 cox2 nad1 nad3 nad2 cox3 rrnS nad5 nad4 -nad4L -cob -nad6 rrnL ]

g3: [ cox1 cox2 nad1 nad3 nad2 cox3 rrnS rrnL nad6 cob nad4L nad5 nad4 ]

g4: [ cox1 cox2 nad1 nad3 nad2 cox3 rrnS nad6 cob nad4L -nad4 -nad5 rrnL ]

g5: [ cox1 cox2 nad1 nad3 nad2 cox3 rrnS -rrnL nad6 cob nad4L -nad4 -nad5 ]

n1(mod33)15g

for n6-g1:

g1[ cox1 cox2 atp8 atp6 cox3 rrnS rrnL nad1 nad6 cob nad4L nad4 nad5 nad3 nad2 ]

for n6-g2:

g2[ cox1 cox2 atp8 atp6 cox3 rrnS rrnL nad1 nad6 cob nad4L nad4 nad5 nad3 nad2 ]

for n6-g3:

g3[ cox1 cox2 atp8 atp6 cox3 rrnS rrnL nad1 nad6 cob nad4L nad4 nad5 nad3 nad2 ]

g4[ cox1 cox2 atp8 atp6 cox3 nad3 nad2 rrnS rrnL nad1 nad6 cob nad4L nad4 nad5 ]

g5[ cox1 cox2 atp8 atp6 -nad5 -nad4 -nad4L -cob -nad6 -rrnL -rrnS nad1 cox3 nad3 nad2 ]

for n6-g4:

g6[ cox1 cox2 atp8 atp6 cox3 rrnS rrnL nad1 nad6 cob nad4L nad4 nad5 nad3 nad2 ]

for n6-g5:

g7[ cox1 cox2 atp8 atp6 nad6 cob nad4L nad4 nad5 -cox3 rrnS rrnL nad1 nad3 nad2 ]

n1(mod33)13g

for n6-g1:

g1[ cox1 cox2 cox3 rrnS rrnL nad1 nad6 cob nad4L nad4 nad5 nad3 nad2 ]

for n6-g2:

g2[ cox1 cox2 cox3 rrnS rrnL nad1 nad6 cob nad4L nad4 nad5 nad3 nad2 ]

for n6-g3:

g3[ cox1 cox2 cox3 rrnS rrnL nad1 nad6 cob nad4L nad4 nad5 nad3 nad2 ]

g4[ cox1 cox2 cox3 nad3 nad2 rrnS rrnL nad1 nad6 cob nad4L nad4 nad5 ]

g5[ cox1 cox2 -nad5 -nad4 -nad4L -cob -nad6 -rrnL -rrnS nad1 cox3 nad3 nad2 ]

for n6-g4:

g6[ cox1 cox2 cox3 rrnS rrnL nad1 nad6 cob nad4L nad4 nad5 nad3 nad2 ]

for n6-g5:

g7[ cox1 cox2 nad6 cob nad4L nad4 nad5 -cox3 rrnS rrnL nad1 nad3 nad2 ]

score = 291:

model 33:

-------------

R(0,1) R(0,9) R(0,10) R(0,17) R(0,18) R(0,24) R(0,25) R(0,28) R(0,n1) R(1,0) R(2,17) R(3,19) R(3,20) R(4,21) R(5,23) R(6,n4) R(7,n4) R(8,24) R(9,0) R(10,0) R(11,27) R(12,31) R(13,14) R(13,15) R(13,n6) R(14,13) R(15,13) R(16,n5) R(17,0) R(17,2) R(18,0) R(18,19) R(19,3) R(19,18) R(19,22) R(20,3) R(20,21) R(21,4) R(21,20) R(22,19) R(22,23) R(23,5) R(23,22) R(24,0) R(24,8) R(25,0) R(25,26) R(26,25) R(26,27) R(27,11) R(27,26) R(28,0) R(28,29) R(29,28) R(29,30) R(30,29) R(30,31) R(31,12) R(31,30) R(n1,0) R(n1,n2) R(n1,n3) R(n2,n1) R(n2,n4) R(n3,n1) R(n3,n7) R(n4,6) R(n4,7) R(n4,n2) R(n5,16) R(n5,n6) R(n6,13) R(n6,n5) R(n6,n7) R(n7,n3) R(n7,n6)

-------------------------------------------------------------------------------------------------------------

-> form outgroup SOL5_ALTER - A

n1(mod34)15g

[ cox1 cox2 atp8 atp6 cox3 rrnS rrnL nad1 nad6 cob nad4L nad4 nad5 nad3 nad2 ]

n1(mod34)13g

[ cox1 cox2 cox3 rrnS rrnL nad1 nad6 cob nad4L nad4 nad5 nad3 nad2 ]

n6(mod34)

g1: [ cox1 cox2 nad1 nad3 nad2 cox3 rrnS -nad5 -nad4 -nad4L -cob -nad6 rrnL ]

g2: [ cox1 cox2 nad1 nad6 cob nad4L nad5 nad4 nad3 nad2 cox3 rrnS rrnL ]

score = 255:

model 34:

-------------

R(0,1) R(0,9) R(0,10) R(0,17) R(0,18) R(0,24) R(0,25) R(0,28) R(0,n1) R(1,0) R(2,17) R(3,19) R(3,20) R(4,21) R(5,23) R(6,n2) R(6,n4) R(7,n4) R(8,24) R(9,0) R(10,0) R(11,27) R(12,31) R(13,14) R(13,15) R(13,n6) R(14,13) R(15,13) R(16,n5) R(17,0) R(17,2) R(18,0) R(18,19) R(19,3) R(19,18) R(19,22) R(20,3) R(20,21) R(21,4) R(21,20) R(22,19) R(22,23) R(23,5) R(23,22) R(24,0) R(24,8) R(25,0) R(25,26) R(26,25) R(26,27) R(27,11) R(27,26) R(28,0) R(28,29) R(29,28) R(29,30) R(30,29) R(30,31) R(31,12) R(31,30) R(n1,0) R(n1,n2) R(n1,n3) R(n2,6) R(n2,n1) R(n3,n1) R(n3,n6) R(n4,6) R(n4,7) R(n5,16) R(n5,n7) R(n6,13) R(n6,n3) R(n6,n7) R(n7,n5) R(n7,n6)

-------------------------------------------------------------------------------------------------------------

-> form outgroup SOL5_ALTER - A

n1(mod35)15g

[ cox1 cox2 atp8 atp6 cox3 rrnS rrnL nad1 nad6 cob nad4L nad4 nad5 nad3 nad2 ]

n1(mod35)13g

[ cox1 cox2 cox3 rrnS rrnL nad1 nad6 cob nad4L nad4 nad5 nad3 nad2 ]

n6(mod35)

[ cox1 cox2 nad1 nad3 nad2 cox3 rrnS -nad5 -nad4 -nad4L -cob -nad6 rrnL ]

score = 269:

model 35:

-------------

R(0,1) R(0,9) R(0,10) R(0,17) R(0,18) R(0,24) R(0,25) R(0,28) R(0,n1) R(1,0) R(2,17) R(3,19) R(3,20) R(4,21) R(5,23) R(6,n2) R(6,n4) R(7,n4) R(8,24) R(9,0) R(10,0) R(11,27) R(12,31) R(13,14) R(13,15) R(14,13) R(15,13) R(15,n6) R(16,n5) R(17,0) R(17,2) R(18,0) R(18,19) R(19,3) R(19,18) R(19,22) R(20,3) R(20,21) R(21,4) R(21,20) R(22,19) R(22,23) R(23,5) R(23,22) R(24,0) R(24,8) R(25,0) R(25,26) R(26,25) R(26,27) R(27,11) R(27,26) R(28,0) R(28,29) R(29,28) R(29,30) R(30,29) R(30,31) R(31,12) R(31,30) R(n1,0) R(n1,n2) R(n1,n3) R(n2,6) R(n2,n1) R(n3,n1) R(n3,n6) R(n4,6) R(n4,7) R(n5,16) R(n5,n7) R(n6,15) R(n6,n3) R(n6,n7) R(n7,n5) R(n7,n6)

-------------------------------------------------------------------------------------------------------------

-> form outgroup SOL5_ALTER - A

n7(mod36)

g1: [ cox1 cox2 nad1 nad3 nad2 cox3 rrnS -nad4 -nad5 -cob -nad6 -nad4L rrnL ]

g2: [ cox1 cox2 nad1 nad3 nad2 cox3 rrnS nad5 nad4 -nad4L -cob -nad6 rrnL ]

g3: [ cox1 cox2 nad1 nad3 nad2 cox3 rrnS rrnL nad6 cob nad4L nad5 nad4 ]

g4: [ cox1 cox2 nad1 nad3 nad2 cox3 rrnS nad6 cob nad4L -nad4 -nad5 rrnL ]

g5: [ cox1 cox2 nad1 nad3 nad2 cox3 rrnS -nad4 -nad5 -rrnL -nad4L -cob -nad6 ]

n1(mod36)15g

for n7-g1:

g1: [ cox1 cox2 atp8 atp6 cox3 rrnS rrnL nad1 nad6 cob nad4L nad4 nad5 nad3 nad2 ]

for n7-g2:

g2: [ cox1 cox2 atp8 atp6 cox3 rrnS rrnL nad1 nad6 cob nad4L nad4 nad5 nad3 nad2 ]

for n7-g3:

g3: [ cox1 cox2 atp8 atp6 cox3 rrnS rrnL nad1 nad6 cob nad4L nad4 nad5 nad3 nad2 ]

g4: [ cox1 cox2 atp8 atp6 cox3 nad3 nad2 rrnS rrnL nad1 nad6 cob nad4L nad4 nad5 ]

g5: [ cox1 cox2 atp8 atp6 -nad5 -nad4 -nad4L -cob -nad6 -cox3 rrnS rrnL nad1 nad3 nad2 ]

for n7-g4:

g6: [ cox1 cox2 atp8 atp6 cox3 rrnS rrnL nad1 nad6 cob nad4L nad4 nad5 nad3 nad2 ]

for n7-g5:

g7: [ cox1 cox2 atp8 atp6 -nad5 -nad4 -nad4L -cob -nad6 -cox3 rrnS rrnL nad1 nad3 nad2 ]

n1(mod36)(13g)

for n7-g1:

g1: [ cox1 cox2 cox3 rrnS rrnL nad1 nad6 cob nad4L nad4 nad5 nad3 nad2 ]

for n7-g2:

g2: [ cox1 cox2 cox3 rrnS rrnL nad1 nad6 cob nad4L nad4 nad5 nad3 nad2 ]

for n7-g3:

g3: [ cox1 cox2 cox3 rrnS rrnL nad1 nad6 cob nad4L nad4 nad5 nad3 nad2 ]

g4: [ cox1 cox2 cox3 nad3 nad2 rrnS rrnL nad1 nad6 cob nad4L nad4 nad5 ]

g5: [ cox1 cox2 -nad5 -nad4 -nad4L -cob -nad6 -cox3 rrnS rrnL nad1 nad3 nad2 ]

for n7-g4:

g6: [ cox1 cox2 cox3 rrnS rrnL nad1 nad6 cob nad4L nad4 nad5 nad3 nad2 ]

for n7-g5:

g7: [ cox1 cox2 -nad5 -nad4 -nad4L -cob -nad6 -cox3 rrnS rrnL nad1 nad3 nad2 ]

score = 291:

model 36:

-------------

R(0,1) R(0,9) R(0,10) R(0,17) R(0,18) R(0,24) R(0,25) R(0,28) R(0,n1) R(1,0) R(2,17) R(3,19) R(3,20) R(4,21) R(5,23) R(6,n2) R(6,n4) R(7,n4) R(8,24) R(9,0) R(10,0) R(11,27) R(12,31) R(13,14) R(13,15) R(13,n7) R(14,13) R(15,13) R(16,n5) R(17,0) R(17,2) R(18,0) R(18,19) R(19,3) R(19,18) R(19,22) R(20,3) R(20,21) R(21,4) R(21,20) R(22,19) R(22,23) R(23,5) R(23,22) R(24,0) R(24,8) R(25,0) R(25,26) R(26,25) R(26,27) R(27,11) R(27,26) R(28,0) R(28,29) R(29,28) R(29,30) R(30,29) R(30,31) R(31,12) R(31,30) R(n1,0) R(n1,n2) R(n1,n3) R(n2,6) R(n2,n1) R(n3,n1) R(n3,n6) R(n4,6) R(n4,7) R(n5,16) R(n5,n7) R(n6,n3) R(n6,n7) R(n7,13) R(n7,n5) R(n7,n6)

-------------------------------------------------------------------------------------------------------------

-> form outgroup SOL5_ALTER - A

n1(mod37)15g

[ cox1 cox2 atp8 atp6 cox3 rrnS rrnL nad1 nad6 cob nad4L nad4 nad5 nad3 nad2 ]

n1(mod37)13g

[ cox1 cox2 cox3 rrnS rrnL nad1 nad6 cob nad4L nad4 nad5 nad3 nad2 ]

score = 279:

model 37:

-------------

R(0,1) R(0,9) R(0,10) R(0,17) R(0,18) R(0,24) R(0,25) R(0,28) R(0,n1) R(1,0) R(2,17) R(3,19) R(3,20) R(4,21) R(5,23) R(6,n2) R(6,n4) R(7,n4) R(8,24) R(9,0) R(10,0) R(11,27) R(12,31) R(13,14) R(13,15) R(14,13) R(15,13) R(15,n6) R(15,n7) R(16,n5) R(17,0) R(17,2) R(18,0) R(18,19) R(19,3) R(19,18) R(19,22) R(20,3) R(20,21) R(21,4) R(21,20) R(22,19) R(22,23) R(23,5) R(23,22) R(24,0) R(24,8) R(25,0) R(25,26) R(26,25) R(26,27) R(27,11) R(27,26) R(28,0) R(28,29) R(29,28) R(29,30) R(30,29) R(30,31) R(31,12) R(31,30) R(n1,0) R(n1,n2) R(n1,n3) R(n2,6) R(n2,n1) R(n3,n1) R(n3,n6) R(n4,6) R(n4,7) R(n5,16) R(n5,n7) R(n6,15) R(n6,n3) R(n7,15) R(n7,n5)

-------------------------------------------------------------------------------------------------------------

-> form outgroup SOL5_ALTER - A

n1(mod38)15g

[ cox1 cox2 atp8 atp6 cox3 rrnS rrnL nad1 nad6 cob nad4L nad4 nad5 nad3 nad2 ]

n1(mod38)13g

[ cox1 cox2 cox3 rrnS rrnL nad1 nad6 cob nad4L nad4 nad5 nad3 nad2 ]

score = 291:

model 38:

-------------

R(0,1) R(0,9) R(0,10) R(0,17) R(0,18) R(0,24) R(0,25) R(0,28) R(0,n1) R(1,0) R(2,17) R(3,19) R(3,20) R(4,21) R(5,23) R(6,n2) R(6,n4) R(7,n4) R(8,24) R(9,0) R(10,0) R(11,27) R(12,31) R(13,14) R(13,15) R(13,n7) R(14,13) R(15,13) R(15,n6) R(16,n5) R(17,0) R(17,2) R(18,0) R(18,19) R(19,3) R(19,18) R(19,22) R(20,3) R(20,21) R(21,4) R(21,20) R(22,19) R(22,23) R(23,5) R(23,22) R(24,0) R(24,8) R(25,0) R(25,26) R(26,25) R(26,27) R(27,11) R(27,26) R(28,0) R(28,29) R(29,28) R(29,30) R(30,29) R(30,31) R(31,12) R(31,30) R(n1,0) R(n1,n2) R(n1,n3) R(n2,6) R(n2,n1) R(n3,n1) R(n3,n6) R(n4,6) R(n4,7) R(n5,16) R(n5,n7) R(n6,15) R(n6,n3) R(n7,13) R(n7,n5)

-------------------------------------------------------------------------------------------------------------

-> form outgroup SOL5_ALTER - A

n1(mod39)15g

[ cox1 cox2 atp8 atp6 cox3 rrnS rrnL nad1 nad6 cob nad4L nad4 nad5 nad3 nad2 ]

n1(mod39)13g

[ cox1 cox2 cox3 rrnS rrnL nad1 nad6 cob nad4L nad4 nad5 nad3 nad2 ]

score = 265:

model 39:

-------------

R(0,1) R(0,9) R(0,10) R(0,17) R(0,18) R(0,24) R(0,25) R(0,28) R(0,n1) R(1,0) R(2,17) R(3,19) R(3,20) R(4,21) R(5,23) R(6,n2) R(6,n4) R(7,n4) R(8,24) R(9,0) R(10,0) R(11,27) R(12,31) R(13,14) R(13,15) R(13,n6) R(13,n7) R(14,13) R(15,13) R(16,n5) R(17,0) R(17,2) R(18,0) R(18,19) R(19,3) R(19,18) R(19,22) R(20,3) R(20,21) R(21,4) R(21,20) R(22,19) R(22,23) R(23,5) R(23,22) R(24,0) R(24,8) R(25,0) R(25,26) R(26,25) R(26,27) R(27,11) R(27,26) R(28,0) R(28,29) R(29,28) R(29,30) R(30,29) R(30,31) R(31,12) R(31,30) R(n1,0) R(n1,n2) R(n1,n3) R(n2,6) R(n2,n1) R(n3,n1) R(n3,n6) R(n4,6) R(n4,7) R(n5,16) R(n5,n7) R(n6,13) R(n6,n3) R(n7,13) R(n7,n5)

-------------------------------------------------------------------------------------------------------------

-> form outgroup SOL5_ALTER - A

n1(mod40)15g

[ cox1 cox2 atp8 atp6 cox3 rrnS rrnL nad1 nad6 cob nad4L nad4 nad5 nad3 nad2 ]

n1(mod40)13g

[ cox1 cox2 cox3 rrnS rrnL nad1 nad6 cob nad4L nad4 nad5 nad3 nad2 ]

score = 279:

model 40:

-------------

R(0,1) R(0,9) R(0,10) R(0,17) R(0,18) R(0,24) R(0,25) R(0,28) R(0,n1) R(1,0) R(2,17) R(3,19) R(3,20) R(4,21) R(5,23) R(6,n2) R(6,n4) R(7,n4) R(8,24) R(9,0) R(10,0) R(11,27) R(12,31) R(13,14) R(13,15) R(13,n6) R(14,13) R(15,13) R(15,n7) R(16,n5) R(17,0) R(17,2) R(18,0) R(18,19) R(19,3) R(19,18) R(19,22) R(20,3) R(20,21) R(21,4) R(21,20) R(22,19) R(22,23) R(23,5) R(23,22) R(24,0) R(24,8) R(25,0) R(25,26) R(26,25) R(26,27) R(27,11) R(27,26) R(28,0) R(28,29) R(29,28) R(29,30) R(30,29) R(30,31) R(31,12) R(31,30) R(n1,0) R(n1,n2) R(n1,n3) R(n2,6) R(n2,n1) R(n3,n1) R(n3,n6) R(n4,6) R(n4,7) R(n5,16) R(n5,n7) R(n6,13) R(n6,n3) R(n7,15) R(n7,n5)

-------------------------------------------------------------------------------------------------------------

-> form outgroup SOL4_ALTER - A

n4(mod41)

[ cox1 cox2 atp8 atp6 cox3 nad3 -nad5 -nad4 -nad4L nad6 cob rrnS rrnL nad1 nad2 ]

n1(mod41)15g

[ cox1 cox2 atp8 atp6 cox3 rrnS rrnL nad1 nad6 cob nad4L nad4 nad5 nad3 nad2 ]

n1(mod41)13g

[ cox1 cox2 cox3 rrnS rrnL nad1 nad6 cob nad4L nad4 nad5 nad3 nad2 ]

n7(mod41)

[ cox1 cox2 nad1 nad3 nad2 cox3 rrnS -nad4L -nad4 -nad5 -cob -nad6 rrnL ]

score = 305:

model 41:

-------------

R(0,1) R(0,9) R(0,10) R(0,17) R(0,18) R(0,24) R(0,25) R(0,28) R(0,n1) R(1,0) R(2,17) R(3,19) R(3,20) R(4,21) R(5,23) R(6,n4) R(7,n4) R(8,24) R(9,0) R(10,0) R(11,27) R(12,31) R(13,14) R(13,n7) R(14,13) R(15,n6) R(15,n7) R(16,n5) R(17,0) R(17,2) R(18,0) R(18,19) R(19,3) R(19,18) R(19,22) R(20,3) R(20,21) R(21,4) R(21,20) R(22,19) R(22,23) R(23,5) R(23,22) R(24,0) R(24,8) R(25,0) R(25,26) R(26,25) R(26,27) R(27,11) R(27,26) R(28,0) R(28,29) R(29,28) R(29,30) R(30,29) R(30,31) R(31,12) R(31,30) R(n1,0) R(n1,n2) R(n1,n3) R(n2,n1) R(n2,n4) R(n3,n1) R(n3,n6) R(n4,6) R(n4,7) R(n4,n2) R(n5,16) R(n5,n7) R(n6,15) R(n6,n3) R(n7,13) R(n7,15) R(n7,n5)

-------------------------------------------------------------------------------------------------------------

-> form outgroup SOL4_ALTER - A

n4(mod42)

[ cox1 cox2 atp8 atp6 cox3 nad3 -nad5 -nad4 -nad4L nad6 cob rrnS rrnL nad1 nad2 ]

n1(mod42)15g

[ cox1 cox2 atp8 atp6 cox3 rrnS rrnL nad1 nad6 cob nad4L nad4 nad5 nad3 nad2 ]

n1(mod42)13g

[ cox1 cox2 cox3 rrnS rrnL nad1 nad6 cob nad4L nad4 nad5 nad3 nad2 ]

n6(mod42)

[ cox1 cox2 nad1 nad3 nad2 cox3 rrnS -nad5 -nad4 -nad4L -cob -nad6 rrnL ]

score = 257:

model 42:

-------------

R(0,1) R(0,9) R(0,10) R(0,17) R(0,18) R(0,24) R(0,25) R(0,28) R(0,n1) R(1,0) R(2,17) R(3,19) R(3,20) R(4,21) R(5,23) R(6,n4) R(7,n4) R(8,24) R(9,0) R(10,0) R(11,27) R(12,31) R(13,14) R(13,n6) R(14,13) R(15,n6) R(15,n7) R(16,n5) R(17,0) R(17,2) R(18,0) R(18,19) R(19,3) R(19,18) R(19,22) R(20,3) R(20,21) R(21,4) R(21,20) R(22,19) R(22,23) R(23,5) R(23,22) R(24,0) R(24,8) R(25,0) R(25,26) R(26,25) R(26,27) R(27,11) R(27,26) R(28,0) R(28,29) R(29,28) R(29,30) R(30,29) R(30,31) R(31,12) R(31,30) R(n1,0) R(n1,n2) R(n1,n3) R(n2,n1) R(n2,n4) R(n3,n1) R(n3,n6) R(n4,6) R(n4,7) R(n4,n2) R(n5,16) R(n5,n7) R(n6,13) R(n6,15) R(n6,n3) R(n7,15) R(n7,n5)

-------------------------------------------------------------------------------------------------------------

-> form outgroup SOL4_ALTER - A

n4(mod43)

[ cox1 cox2 atp8 atp6 cox3 nad3 -nad5 -nad4 -nad4L nad6 cob rrnS rrnL nad1 nad2 ]

n1(mod43)15g

[ cox1 cox2 atp8 atp6 cox3 rrnS rrnL nad1 nad6 cob nad4L nad4 nad5 nad3 nad2 ]

n1(mod43)13g

[ cox1 cox2 cox3 rrnS rrnL nad1 nad6 cob nad4L nad4 nad5 nad3 nad2 ]

n6(mod43)

[ cox1 cox2 nad1 nad3 nad2 cox3 rrnS -nad5 -nad4 -nad4L -cob -nad6 rrnL ]

score = 243:

model 43:

-------------

R(0,1) R(0,9) R(0,10) R(0,17) R(0,18) R(0,24) R(0,25) R(0,28) R(0,n1) R(1,0) R(2,17) R(3,19) R(3,20) R(4,21) R(5,23) R(6,n4) R(7,n4) R(8,24) R(9,0) R(10,0) R(11,27) R(12,31) R(13,14) R(13,n6) R(14,13) R(15,n6) R(16,n5) R(17,0) R(17,2) R(18,0) R(18,19) R(19,3) R(19,18) R(19,22) R(20,3) R(20,21) R(21,4) R(21,20) R(22,19) R(22,23) R(23,5) R(23,22) R(24,0) R(24,8) R(25,0) R(25,26) R(26,25) R(26,27) R(27,11) R(27,26) R(28,0) R(28,29) R(29,28) R(29,30) R(30,29) R(30,31) R(31,12) R(31,30) R(n1,0) R(n1,n2) R(n1,n3) R(n2,n1) R(n2,n4) R(n3,n1) R(n3,n6) R(n4,6) R(n4,7) R(n4,n2) R(n5,16) R(n5,n7) R(n6,13) R(n6,15) R(n6,n3) R(n6,n7) R(n7,n5) R(n7,n6)

-------------------------------------------------------------------------------------------------------------

-> form outgroup SOL4_ALTER - A

n4(mod44)

[ cox1 cox2 atp8 atp6 cox3 nad3 -nad5 -nad4 -nad4L nad6 cob rrnS rrnL nad1 nad2 ]

n1(mod44)15g

[ cox1 cox2 atp8 atp6 cox3 rrnS rrnL nad1 nad6 cob nad4L nad4 nad5 nad3 nad2 ]

n1(mod44)13g

[ cox1 cox2 cox3 rrnS rrnL nad1 nad6 cob nad4L nad4 nad5 nad3 nad2 ]

n6(mod44)

[ cox1 cox2 nad1 nad3 nad2 cox3 rrnS -nad5 -nad4 -nad4L -cob -nad6 rrnL ]

n7(mod44)

[ cox1 cox2 nad1 nad3 nad2 cox3 rrnS nad5 nad4 -nad4L -cob -nad6 rrnL ]

score = 269:

model 44:

-------------

R(0,1) R(0,9) R(0,10) R(0,17) R(0,18) R(0,24) R(0,25) R(0,28) R(0,n1) R(1,0) R(2,17) R(3,19) R(3,20) R(4,21) R(5,23) R(6,n4) R(7,n4) R(8,24) R(9,0) R(10,0) R(11,27) R(12,31) R(13,14) R(13,n7) R(14,13) R(15,n6) R(16,n5) R(17,0) R(17,2) R(18,0) R(18,19) R(19,3) R(19,18) R(19,22) R(20,3) R(20,21) R(21,4) R(21,20) R(22,19) R(22,23) R(23,5) R(23,22) R(24,0) R(24,8) R(25,0) R(25,26) R(26,25) R(26,27) R(27,11) R(27,26) R(28,0) R(28,29) R(29,28) R(29,30) R(30,29) R(30,31) R(31,12) R(31,30) R(n1,0) R(n1,n2) R(n1,n3) R(n2,n1) R(n2,n4) R(n3,n1) R(n3,n6) R(n4,6) R(n4,7) R(n4,n2) R(n5,16) R(n5,n7) R(n6,15) R(n6,n3) R(n6,n7) R(n7,13) R(n7,n5) R(n7,n6)

-------------------------------------------------------------------------------------------------------------

-> form outgroup SOL4_ALTER - A

n4(mod45)

[ cox1 cox2 atp8 atp6 cox3 nad3 -nad5 -nad4 -nad4L nad6 cob rrnS rrnL nad1 nad2 ]

n1(mod45)15g

[ cox1 cox2 atp8 atp6 cox3 rrnS rrnL nad1 nad6 cob nad4L nad4 nad5 nad3 nad2 ]

n1(mod45)13g

[ cox1 cox2 cox3 rrnS rrnL nad1 nad6 cob nad4L nad4 nad5 nad3 nad2 ]

n6(mod45)

[ cox1 cox2 nad1 nad3 nad2 cox3 rrnS -nad5 -nad4 -nad4L -cob -nad6 rrnL ]

score = 255:

model 45:

-------------

R(0,1) R(0,9) R(0,10) R(0,17) R(0,18) R(0,24) R(0,25) R(0,28) R(0,n1) R(1,0) R(2,17) R(3,19) R(3,20) R(4,21) R(5,23) R(6,n4) R(7,n4) R(8,24) R(9,0) R(10,0) R(11,27) R(12,31) R(13,14) R(13,n6) R(13,n7) R(14,13) R(15,n6) R(16,n5) R(17,0) R(17,2) R(18,0) R(18,19) R(19,3) R(19,18) R(19,22) R(20,3) R(20,21) R(21,4) R(21,20) R(22,19) R(22,23) R(23,5) R(23,22) R(24,0) R(24,8) R(25,0) R(25,26) R(26,25) R(26,27) R(27,11) R(27,26) R(28,0) R(28,29) R(29,28) R(29,30) R(30,29) R(30,31) R(31,12) R(31,30) R(n1,0) R(n1,n2) R(n1,n3) R(n2,n1) R(n2,n4) R(n3,n1) R(n3,n6) R(n4,6) R(n4,7) R(n4,n2) R(n5,16) R(n5,n7) R(n6,13) R(n6,15) R(n6,n3) R(n7,13) R(n7,n5)

-------------------------------------------------------------------------------------------------------------

-> form outgroup SOL4_ALTER - A

n4(mod46)

[ cox1 cox2 atp8 atp6 cox3 nad3 -nad5 -nad4 -nad4L nad6 cob rrnS rrnL nad1 nad2 ]

n1(mod46)15g

[ cox1 cox2 atp8 atp6 cox3 rrnS rrnL nad1 nad6 cob nad4L nad4 nad5 nad3 nad2 ]

n1(mod46)13g

[ cox1 cox2 cox3 rrnS rrnL nad1 nad6 cob nad4L nad4 nad5 nad3 nad2 ]

n6(mod46)

[ cox1 cox2 nad1 nad3 nad2 cox3 rrnS -nad4L -nad4 -nad5 -cob -nad6 rrnL ]

score = 279:

model 46:

-------------

R(0,1) R(0,9) R(0,10) R(0,17) R(0,18) R(0,24) R(0,25) R(0,28) R(0,n1) R(1,0) R(2,17) R(3,19) R(3,20) R(4,21) R(5,23) R(6,n4) R(7,n4) R(8,24) R(9,0) R(10,0) R(11,27) R(12,31) R(13,14) R(13,n6) R(13,n7) R(14,13) R(15,n6) R(16,n5) R(17,0) R(17,2) R(18,0) R(18,19) R(19,3) R(19,18) R(19,22) R(20,3) R(20,21) R(21,4) R(21,20) R(22,19) R(22,23) R(23,5) R(23,22) R(24,0) R(24,8) R(25,0) R(25,26) R(26,25) R(26,27) R(27,11) R(27,26) R(28,0) R(28,29) R(29,28) R(29,30) R(30,29) R(30,31) R(31,12) R(31,30) R(n1,0) R(n1,n2) R(n1,n3) R(n2,n1) R(n2,n4) R(n3,n1) R(n3,n7) R(n4,6) R(n4,7) R(n4,n2) R(n5,16) R(n5,n6) R(n6,13) R(n6,15) R(n6,n5) R(n7,13) R(n7,n3)

-------------------------------------------------------------------------------------------------------------

-> form outgroup SOL5_ALTER - A

n1(mod47)15g

[ cox1 cox2 atp8 atp6 cox3 rrnS rrnL nad1 nad6 cob nad4L nad4 nad5 nad3 nad2 ]

n1(mod47)13g

[ cox1 cox2 cox3 rrnS rrnL nad1 nad6 cob nad4L nad4 nad5 nad3 nad2 ]

n6(mod47)

[ cox1 cox2 nad1 nad3 nad2 cox3 rrnS -nad5 -nad4 -nad4L -cob -nad6 rrnL ]

score = 243:

model 47:

-------------

R(0,1) R(0,9) R(0,10) R(0,17) R(0,18) R(0,24) R(0,25) R(0,28) R(0,n1) R(1,0) R(2,17) R(3,19) R(3,20) R(4,21) R(5,23) R(6,n2) R(6,n4) R(7,n4) R(8,24) R(9,0) R(10,0) R(11,27) R(12,31) R(13,14) R(13,n6) R(14,13) R(15,n6) R(16,n5) R(17,0) R(17,2) R(18,0) R(18,19) R(19,3) R(19,18) R(19,22) R(20,3) R(20,21) R(21,4) R(21,20) R(22,19) R(22,23) R(23,5) R(23,22) R(24,0) R(24,8) R(25,0) R(25,26) R(26,25) R(26,27) R(27,11) R(27,26) R(28,0) R(28,29) R(29,28) R(29,30) R(30,29) R(30,31) R(31,12) R(31,30) R(n1,0) R(n1,n2) R(n1,n3) R(n2,6) R(n2,n1) R(n3,n1) R(n3,n6) R(n4,6) R(n4,7) R(n5,16) R(n5,n7) R(n6,13) R(n6,15) R(n6,n3) R(n6,n7) R(n7,n5) R(n7,n6)

-------------------------------------------------------------------------------------------------------------

-> form outgroup SOL5_ALTER - A

n1(mod48)15g

[ cox1 cox2 atp8 atp6 cox3 rrnS rrnL nad1 nad6 cob nad4L nad4 nad5 nad3 nad2 ]

n1(mod48)13g

[ cox1 cox2 cox3 rrnS rrnL nad1 nad6 cob nad4L nad4 nad5 nad3 nad2 ]

n6(mod48)

[ cox1 cox2 nad1 nad3 nad2 cox3 rrnS -nad5 -nad4 -nad4L -cob -nad6 rrnL ]

n7(mod48)

[ cox1 cox2 nad1 nad3 nad2 cox3 rrnS nad5 nad4 -nad4L -cob -nad6 rrnL ]

score = 269:

model 48:

-------------

R(0,1) R(0,9) R(0,10) R(0,17) R(0,18) R(0,24) R(0,25) R(0,28) R(0,n1) R(1,0) R(2,17) R(3,19) R(3,20) R(4,21) R(5,23) R(6,n2) R(6,n4) R(7,n4) R(8,24) R(9,0) R(10,0) R(11,27) R(12,31) R(13,14) R(13,n7) R(14,13) R(15,n6) R(16,n5) R(17,0) R(17,2) R(18,0) R(18,19) R(19,3) R(19,18) R(19,22) R(20,3) R(20,21) R(21,4) R(21,20) R(22,19) R(22,23) R(23,5) R(23,22) R(24,0) R(24,8) R(25,0) R(25,26) R(26,25) R(26,27) R(27,11) R(27,26) R(28,0) R(28,29) R(29,28) R(29,30) R(30,29) R(30,31) R(31,12) R(31,30) R(n1,0) R(n1,n2) R(n1,n3) R(n2,6) R(n2,n1) R(n3,n1) R(n3,n6) R(n4,6) R(n4,7) R(n5,16) R(n5,n7) R(n6,15) R(n6,n3) R(n6,n7) R(n7,13) R(n7,n5) R(n7,n6)

-------------------------------------------------------------------------------------------------------------

-> form outgroup SOL5_ALTER - A

n1(mod49)15g

[ cox1 cox2 atp8 atp6 cox3 rrnS rrnL nad1 nad6 cob nad4L nad4 nad5 nad3 nad2 ]

n1(mod49)13g

[ cox1 cox2 cox3 rrnS rrnL nad1 nad6 cob nad4L nad4 nad5 nad3 nad2 ]

n6(mod49)

[ cox1 cox2 nad1 nad3 nad2 cox3 rrnS -nad5 -nad4 -nad4L -cob -nad6 rrnL ]

score = 255:

model 49:

-------------

R(0,1) R(0,9) R(0,10) R(0,17) R(0,18) R(0,24) R(0,25) R(0,28) R(0,n1) R(1,0) R(2,17) R(3,19) R(3,20) R(4,21) R(5,23) R(6,n2) R(6,n4) R(7,n4) R(8,24) R(9,0) R(10,0) R(11,27) R(12,31) R(13,14) R(13,n6) R(13,n7) R(14,13) R(15,n6) R(16,n5) R(17,0) R(17,2) R(18,0) R(18,19) R(19,3) R(19,18) R(19,22) R(20,3) R(20,21) R(21,4) R(21,20) R(22,19) R(22,23) R(23,5) R(23,22) R(24,0) R(24,8) R(25,0) R(25,26) R(26,25) R(26,27) R(27,11) R(27,26) R(28,0) R(28,29) R(29,28) R(29,30) R(30,29) R(30,31) R(31,12) R(31,30) R(n1,0) R(n1,n2) R(n1,n3) R(n2,6) R(n2,n1) R(n3,n1) R(n3,n6) R(n4,6) R(n4,7) R(n5,16) R(n5,n7) R(n6,13) R(n6,15) R(n6,n3) R(n7,13) R(n7,n5)

-------------------------------------------------------------------------------------------------------------

-> form outgroup SOL5_ALTER - A

n1(mod50)15g

[ cox1 cox2 atp8 atp6 cox3 rrnS rrnL nad1 nad6 cob nad4L nad4 nad5 nad3 nad2 ]

n1(mod50)13g

[ cox1 cox2 cox3 rrnS rrnL nad1 nad6 cob nad4L nad4 nad5 nad3 nad2 ]

n6(mod50)

[ cox1 cox2 nad1 nad3 nad2 cox3 rrnS -nad5 -nad4 -nad4L -cob -nad6 rrnL ]

score = 257:

model 50:

-------------

R(0,1) R(0,9) R(0,10) R(0,17) R(0,18) R(0,24) R(0,25) R(0,28) R(0,n1) R(1,0) R(2,17) R(3,19) R(3,20) R(4,21) R(5,23) R(6,n2) R(6,n4) R(7,n4) R(8,24) R(9,0) R(10,0) R(11,27) R(12,31) R(13,14) R(13,n6) R(14,13) R(15,n6) R(15,n7) R(16,n5) R(17,0) R(17,2) R(18,0) R(18,19) R(19,3) R(19,18) R(19,22) R(20,3) R(20,21) R(21,4) R(21,20) R(22,19) R(22,23) R(23,5) R(23,22) R(24,0) R(24,8) R(25,0) R(25,26) R(26,25) R(26,27) R(27,11) R(27,26) R(28,0) R(28,29) R(29,28) R(29,30) R(30,29) R(30,31) R(31,12) R(31,30) R(n1,0) R(n1,n2) R(n1,n3) R(n2,6) R(n2,n1) R(n3,n1) R(n3,n6) R(n4,6) R(n4,7) R(n5,16) R(n5,n7) R(n6,13) R(n6,15) R(n6,n3) R(n7,15) R(n7,n5)

-------------------------------------------------------------------------------------------------------------

-> form outgroup SOL5_ALTER - A

n1(mod51)15g

[ cox1 cox2 atp8 atp6 cox3 rrnS rrnL nad1 nad6 cob nad4L nad4 nad5 nad3 nad2 ]

n1(mod51)13g

[ cox1 cox2 cox3 rrnS rrnL nad1 nad6 cob nad4L nad4 nad5 nad3 nad2 ]

n7(mod51)

[ cox1 cox2 nad1 nad3 nad2 cox3 rrnS -nad4L -nad4 -nad5 -cob -nad6 rrnL ]

score = 279:

model 51:

-------------

R(0,1) R(0,9) R(0,10) R(0,17) R(0,18) R(0,24) R(0,25) R(0,28) R(0,n1) R(1,0) R(2,17) R(3,19) R(3,20) R(4,21) R(5,23) R(6,n2) R(6,n4) R(7,n4) R(8,24) R(9,0) R(10,0) R(11,27) R(12,31) R(13,14) R(13,n6) R(13,n7) R(14,13) R(15,n7) R(16,n5) R(17,0) R(17,2) R(18,0) R(18,19) R(19,3) R(19,18) R(19,22) R(20,3) R(20,21) R(21,4) R(21,20) R(22,19) R(22,23) R(23,5) R(23,22) R(24,0) R(24,8) R(25,0) R(25,26) R(26,25) R(26,27) R(27,11) R(27,26) R(28,0) R(28,29) R(29,28) R(29,30) R(30,29) R(30,31) R(31,12) R(31,30) R(n1,0) R(n1,n2) R(n1,n3) R(n2,6) R(n2,n1) R(n3,n1) R(n3,n6) R(n4,6) R(n4,7) R(n5,16) R(n5,n7) R(n6,13) R(n6,n3) R(n7,13) R(n7,15) R(n7,n5)

-------------------------------------------------------------------------------------------------------------

-> form outgroup SOL5_ALTER - A

n1(mod52)15g

[ cox1 cox2 atp8 atp6 cox3 rrnS rrnL nad1 nad6 cob nad4L nad4 nad5 nad3 nad2 ]

n1(mod52)13g

[ cox1 cox2 cox3 rrnS rrnL nad1 nad6 cob nad4L nad4 nad5 nad3 nad2 ]

n7(mod52)

[ cox1 cox2 nad1 nad3 nad2 cox3 rrnS -nad4L -nad4 -nad5 -cob -nad6 rrnL ]

score = 305:

model 52:

-------------

R(0,1) R(0,9) R(0,10) R(0,17) R(0,18) R(0,24) R(0,25) R(0,28) R(0,n1) R(1,0) R(2,17) R(3,19) R(3,20) R(4,21) R(5,23) R(6,n2) R(6,n4) R(7,n4) R(8,24) R(9,0) R(10,0) R(11,27) R(12,31) R(13,14) R(13,n7) R(14,13) R(15,n6) R(15,n7) R(16,n5) R(17,0) R(17,2) R(18,0) R(18,19) R(19,3) R(19,18) R(19,22) R(20,3) R(20,21) R(21,4) R(21,20) R(22,19) R(22,23) R(23,5) R(23,22) R(24,0) R(24,8) R(25,0) R(25,26) R(26,25) R(26,27) R(27,11) R(27,26) R(28,0) R(28,29) R(29,28) R(29,30) R(30,29) R(30,31) R(31,12) R(31,30) R(n1,0) R(n1,n2) R(n1,n3) R(n2,6) R(n2,n1) R(n3,n1) R(n3,n6) R(n4,6) R(n4,7) R(n5,16) R(n5,n7) R(n6,15) R(n6,n3) R(n7,13) R(n7,15) R(n7,n5)

-------------------------------------------------------------------------------------------------------------

-> form outgroup SOL1_ALTER - B

n3(mod53)

[ cox1 cox2 atp8 atp6 cox3 nad3 -nad5 -nad4 -nad4L nad6 cob rrnS rrnL nad1 nad2 ]

n1(mod53)15g

[ cox1 cox2 atp8 atp6 cox3 nad3 -nad5 -nad4 -nad4L -cob -nad6 -nad1 -rrnL -rrnS nad2 ]

n1(mod53)13g

[ cox1 cox2 cox3 nad3 -nad5 -nad4 -nad4L -cob -nad6 -nad1 -rrnL -rrnS nad2 ]

score = 287:

model 53:

-------------

R(0,1) R(0,9) R(0,10) R(0,17) R(0,18) R(0,24) R(0,25) R(0,28) R(0,n1) R(1,0) R(2,17) R(3,19) R(3,20) R(4,21) R(5,23) R(6,n3) R(7,n3) R(8,24) R(9,0) R(10,0) R(11,27) R(12,31) R(13,14) R(13,15) R(13,n4) R(13,n5) R(14,13) R(15,13) R(16,n6) R(17,0) R(17,2) R(18,0) R(18,19) R(19,3) R(19,18) R(19,22) R(20,3) R(20,21) R(21,4) R(21,20) R(22,19) R(22,23) R(23,5) R(23,22) R(24,0) R(24,8) R(25,0) R(25,26) R(26,25) R(26,27) R(27,11) R(27,26) R(28,0) R(28,29) R(29,28) R(29,30) R(30,29) R(30,31) R(31,12) R(31,30) R(n1,0) R(n1,n2) R(n1,n3) R(n2,n1) R(n2,n7) R(n3,6) R(n3,7) R(n3,n1) R(n4,13) R(n4,n6) R(n5,13) R(n5,n7) R(n6,16) R(n6,n4) R(n7,n2) R(n7,n5)

-------------------------------------------------------------------------------------------------------------

-> form outgroup SOL1_ALTER - B

n3(mod54)

[ cox1 cox2 atp8 atp6 cox3 nad3 -nad5 -nad4 -nad4L nad6 cob rrnS rrnL nad1 nad2 ]

n1(mod54)15g

[ cox1 cox2 atp8 atp6 cox3 nad3 -nad5 -nad4 -nad4L -cob -nad6 -nad1 -rrnL -rrnS nad2 ]

n1(mod54)13g

[ cox1 cox2 cox3 nad3 -nad5 -nad4 -nad4L -cob -nad6 -nad1 -rrnL -rrnS nad2 ]

n4(mod54)

[ cox1 cox2 nad1 nad3 nad2 cox3 rrnS -nad4L -nad4 -nad5 -cob -nad6 rrnL ]

score = 301:

model 54:

-------------

R(0,1) R(0,9) R(0,10) R(0,17) R(0,18) R(0,24) R(0,25) R(0,28) R(0,n1) R(1,0) R(2,17) R(3,19) R(3,20) R(4,21) R(5,23) R(6,n3) R(7,n3) R(8,24) R(9,0) R(10,0) R(11,27) R(12,31) R(13,14) R(13,n4) R(13,n5) R(14,13) R(15,n4) R(16,n6) R(17,0) R(17,2) R(18,0) R(18,19) R(19,3) R(19,18) R(19,22) R(20,3) R(20,21) R(21,4) R(21,20) R(22,19) R(22,23) R(23,5) R(23,22) R(24,0) R(24,8) R(25,0) R(25,26) R(26,25) R(26,27) R(27,11) R(27,26) R(28,0) R(28,29) R(29,28) R(29,30) R(30,29) R(30,31) R(31,12) R(31,30) R(n1,0) R(n1,n2) R(n1,n3) R(n2,n1) R(n2,n7) R(n3,6) R(n3,7) R(n3,n1) R(n4,13) R(n4,15) R(n4,n6) R(n5,13) R(n5,n7) R(n6,16) R(n6,n4) R(n7,n2) R(n7,n5)

-------------------------------------------------------------------------------------------------------------

-> form outgroup SOL1_ALTER - B

n3(mod55)

[ cox1 cox2 atp8 atp6 cox3 nad3 -nad5 -nad4 -nad4L nad6 cob rrnS rrnL nad1 nad2 ]

n1(mod55)15g

[ cox1 cox2 atp8 atp6 cox3 nad3 -nad5 -nad4 -nad4L -cob -nad6 -nad1 -rrnL -rrnS nad2 ]

n1(mod55)13g

[ cox1 cox2 cox3 nad3 -nad5 -nad4 -nad4L -cob -nad6 -nad1 -rrnL -rrnS nad2 ]

n5(mod55)

[ cox1 cox2 nad1 nad3 nad2 cox3 rrnS -nad5 -nad4 -nad4L -cob -nad6 rrnL ]

score = 277:

model 55:

-------------

R(0,1) R(0,9) R(0,10) R(0,17) R(0,18) R(0,24) R(0,25) R(0,28) R(0,n1) R(1,0) R(2,17) R(3,19) R(3,20) R(4,21) R(5,23) R(6,n3) R(7,n3) R(8,24) R(9,0) R(10,0) R(11,27) R(12,31) R(13,14) R(13,n4) R(13,n5) R(14,13) R(15,n5) R(16,n6) R(17,0) R(17,2) R(18,0) R(18,19) R(19,3) R(19,18) R(19,22) R(20,3) R(20,21) R(21,4) R(21,20) R(22,19) R(22,23) R(23,5) R(23,22) R(24,0) R(24,8) R(25,0) R(25,26) R(26,25) R(26,27) R(27,11) R(27,26) R(28,0) R(28,29) R(29,28) R(29,30) R(30,29) R(30,31) R(31,12) R(31,30) R(n1,0) R(n1,n2) R(n1,n3) R(n2,n1) R(n2,n7) R(n3,6) R(n3,7) R(n3,n1) R(n4,13) R(n4,n6) R(n5,13) R(n5,15) R(n5,n7) R(n6,16) R(n6,n4) R(n7,n2) R(n7,n5)

-------------------------------------------------------------------------------------------------------------

-> form outgroup SOL1_ALTER - B

n3(mod56)

[ cox1 cox2 atp8 atp6 cox3 nad3 -nad5 -nad4 -nad4L nad6 cob rrnS rrnL nad1 nad2 ]

n1(mod56)15g

[ cox1 cox2 atp8 atp6 cox3 nad3 -nad5 -nad4 -nad4L -cob -nad6 -nad1 -rrnL -rrnS nad2 ]

n1(mod56)13g

[ cox1 cox2 cox3 nad3 -nad5 -nad4 -nad4L -cob -nad6 -nad1 -rrnL -rrnS nad2 ]

n4(mod56)

[ cox1 cox2 nad1 nad3 nad2 cox3 rrnS -nad4L -nad4 -nad5 -cob -nad6 rrnL ]

score = 327:

model 56:

-------------

R(0,1) R(0,9) R(0,10) R(0,17) R(0,18) R(0,24) R(0,25) R(0,28) R(0,n1) R(1,0) R(2,17) R(3,19) R(3,20) R(4,21) R(5,23) R(6,n3) R(7,n3) R(8,24) R(9,0) R(10,0) R(11,27) R(12,31) R(13,14) R(13,n4) R(14,13) R(15,n4) R(15,n6) R(16,n5) R(17,0) R(17,2) R(18,0) R(18,19) R(19,3) R(19,18) R(19,22) R(20,3) R(20,21) R(21,4) R(21,20) R(22,19) R(22,23) R(23,5) R(23,22) R(24,0) R(24,8) R(25,0) R(25,26) R(26,25) R(26,27) R(27,11) R(27,26) R(28,0) R(28,29) R(29,28) R(29,30) R(30,29) R(30,31) R(31,12) R(31,30) R(n1,0) R(n1,n2) R(n1,n3) R(n2,n1) R(n2,n7) R(n3,6) R(n3,7) R(n3,n1) R(n4,13) R(n4,15) R(n4,n5) R(n5,16) R(n5,n4) R(n6,15) R(n6,n7) R(n7,n2) R(n7,n6)

-------------------------------------------------------------------------------------------------------------

-> form outgroup SOL1_ALTER - B

n2(mod57)

[ cox1 cox2 atp8 atp6 cox3 nad3 -nad5 -nad4 -nad4L nad6 cob rrnS rrnL nad1 nad2 ]

n1(mod57)15g

[ cox1 cox2 atp8 atp6 cox3 nad3 -nad5 -nad4 -nad4L -cob -nad6 -nad1 -rrnL -rrnS nad2 ]

n1(mod57)13g

[ cox1 cox2 cox3 nad3 -nad5 -nad4 -nad4L -cob -nad6 -nad1 -rrnL -rrnS nad2 ]

n4(mod57)

[ cox1 cox2 nad1 nad3 nad2 cox3 rrnS -nad5 -nad4 -nad4L -cob -nad6 rrnL ]

score = 279:

model 57:

-------------

R(0,1) R(0,9) R(0,10) R(0,17) R(0,18) R(0,24) R(0,25) R(0,28) R(0,n1) R(1,0) R(2,17) R(3,19) R(3,20) R(4,21) R(5,23) R(6,n2) R(7,n2) R(8,24) R(9,0) R(10,0) R(11,27) R(12,31) R(13,14) R(13,n4) R(14,13) R(15,n4) R(15,n7) R(16,n5) R(17,0) R(17,2) R(18,0) R(18,19) R(19,3) R(19,18) R(19,22) R(20,3) R(20,21) R(21,4) R(21,20) R(22,19) R(22,23) R(23,5) R(23,22) R(24,0) R(24,8) R(25,0) R(25,26) R(26,25) R(26,27) R(27,11) R(27,26) R(28,0) R(28,29) R(29,28) R(29,30) R(30,29) R(30,31) R(31,12) R(31,30) R(n1,0) R(n1,n2) R(n1,n3) R(n2,6) R(n2,7) R(n2,n1) R(n3,n1) R(n3,n6) R(n4,13) R(n4,15) R(n4,n6) R(n5,16) R(n5,n7) R(n6,n3) R(n6,n4) R(n7,15) R(n7,n5)

-------------------------------------------------------------------------------------------------------------

-> form outgroup SOL1_ALTER - B

n2(mod58)

[ cox1 cox2 atp8 atp6 cox3 nad3 -nad5 -nad4 -nad4L nad6 cob rrnS rrnL nad1 nad2 ]

n1(mod58)15g

[ cox1 cox2 atp8 atp6 cox3 nad3 -nad5 -nad4 -nad4L -cob -nad6 -nad1 -rrnL -rrnS nad2 ]

n1(mod58)13g

[ cox1 cox2 cox3 nad3 -nad5 -nad4 -nad4L -cob -nad6 -nad1 -rrnL -rrnS nad2 ]

n4(mod58)

[ cox1 cox2 nad1 nad3 nad2 cox3 rrnS -nad5 -nad4 -nad4L -cob -nad6 rrnL ]

score = 265:

model 58:

-------------

R(0,1) R(0,9) R(0,10) R(0,17) R(0,18) R(0,24) R(0,25) R(0,28) R(0,n1) R(1,0) R(2,17) R(3,19) R(3,20) R(4,21) R(5,23) R(6,n2) R(7,n2) R(8,24) R(9,0) R(10,0) R(11,27) R(12,31) R(13,14) R(13,n4) R(14,13) R(15,n4) R(16,n5) R(17,0) R(17,2) R(18,0) R(18,19) R(19,3) R(19,18) R(19,22) R(20,3) R(20,21) R(21,4) R(21,20) R(22,19) R(22,23) R(23,5) R(23,22) R(24,0) R(24,8) R(25,0) R(25,26) R(26,25) R(26,27) R(27,11) R(27,26) R(28,0) R(28,29) R(29,28) R(29,30) R(30,29) R(30,31) R(31,12) R(31,30) R(n1,0) R(n1,n2) R(n1,n3) R(n2,6) R(n2,7) R(n2,n1) R(n3,n1) R(n3,n6) R(n4,13) R(n4,15) R(n4,n6) R(n4,n7) R(n5,16) R(n5,n7) R(n6,n3) R(n6,n4) R(n7,n4) R(n7,n5)

-------------------------------------------------------------------------------------------------------------

-> form outgroup SOL1_ALTER - B

n3(mod59)

[ cox1 cox2 atp8 atp6 cox3 nad3 -nad5 -nad4 -nad4L nad6 cob rrnS rrnL nad1 nad2 ]

n1(mod59)15g

g1[ cox1 cox2 atp8 atp6 -nad5 -nad4 -nad4L nad6 cob rrnS rrnL nad1 cox3 nad3 nad2 ]

g2[ cox1 cox2 atp8 atp6 nad6 cob nad4L nad4 nad5 -nad3 -cox3 rrnS rrnL nad1 nad2 ]

g3[ cox1 cox2 atp8 atp6 cox3 nad3 -nad5 -nad4 -nad4L -cob -nad6 -nad1 -rrnL -rrnS nad2 ]

n1(mod59)13g

g1[ cox1 cox2 -nad5 -nad4 -nad4L nad6 cob rrnS rrnL nad1 cox3 nad3 nad2 ]

g2[ cox1 cox2 nad6 cob nad4L nad4 nad5 -nad3 -cox3 rrnS rrnL nad1 nad2 ]

g3[ cox1 cox2 cox3 nad3 -nad5 -nad4 -nad4L -cob -nad6 -nad1 -rrnL -rrnS nad2 ]

n4(mod59)

for n1-g1:

g1: [ cox1 cox2 nad1 nad3 nad2 cox3 rrnS -nad4 -nad5 -cob -nad6 rrnL -nad4L ]

g2: [ cox1 cox2 nad1 nad3 nad2 cox3 rrnS rrnL nad6 cob nad4L nad5 nad4 ]

g3: [ cox1 cox2 nad1 nad3 nad2 cox3 rrnS -nad4 -nad5 -cob -nad6 nad4L rrnL ]

g4: [ cox1 cox2 nad1 nad3 nad2 cox3 rrnS -nad4 -nad5 -rrnL -nad4L -cob -nad6 ]

for n1-g2:

g5: [ cox1 cox2 nad1 nad3 nad2 cox3 rrnS rrnL nad6 cob nad4L nad5 nad4 ]

g6: [ cox1 cox2 nad1 nad3 nad2 cox3 rrnS -rrnL nad6 cob nad4L nad5 nad4 ]

g7: [ cox1 cox2 nad1 nad3 nad2 cox3 rrnS -rrnL nad6 cob nad4L -nad4 -nad5 ]

for n1-g3:

g8: [ cox1 cox2 nad1 nad3 nad2 cox3 rrnS -nad4 -nad5 -cob -nad6 -nad4L rrnL ]

g9: [ cox1 cox2 nad1 nad3 nad2 cox3 rrnS nad5 nad4 -nad4L -cob -nad6 rrnL ]

g10: [ cox1 cox2 nad1 nad3 nad2 cox3 rrnS rrnL nad6 cob nad4L nad5 nad4 ]

g11: [ cox1 cox2 nad1 nad3 nad2 cox3 rrnS nad6 cob nad4L -nad4 -nad5 rrnL ]

g12: [ cox1 cox2 nad1 nad3 nad2 cox3 rrnS -nad4 -nad5 nad6 cob nad4L rrnL ]

score = 313:

model 59:

-------------

R(0,1) R(0,9) R(0,10) R(0,17) R(0,18) R(0,24) R(0,25) R(0,28) R(0,n1) R(1,0) R(2,17) R(3,19) R(3,20) R(4,21) R(5,23) R(6,n3) R(7,n3) R(8,24) R(9,0) R(10,0) R(11,27) R(12,31) R(13,14) R(13,15) R(13,n4) R(14,13) R(15,13) R(16,n5) R(17,0) R(17,2) R(18,0) R(18,19) R(19,3) R(19,18) R(19,22) R(20,3) R(20,21) R(21,4) R(21,20) R(22,19) R(22,23) R(23,5) R(23,22) R(24,0) R(24,8) R(25,0) R(25,26) R(26,25) R(26,27) R(27,11) R(27,26) R(28,0) R(28,29) R(29,28) R(29,30) R(30,29) R(30,31) R(31,12) R(31,30) R(n1,0) R(n1,n2) R(n1,n3) R(n2,n1) R(n2,n7) R(n3,6) R(n3,7) R(n3,n1) R(n4,13) R(n4,n5) R(n4,n6) R(n5,16) R(n5,n4) R(n6,n4) R(n6,n7) R(n7,n2) R(n7,n6)

-------------------------------------------------------------------------------------------------------------

-> form outgroup SOL1_ALTER - B

n3(mod60)

[ cox1 cox2 atp8 atp6 cox3 nad3 -nad5 -nad4 -nad4L nad6 cob rrnS rrnL nad1 nad2 ]

n1(mod60)15g

[ cox1 cox2 atp8 atp6 cox3 nad3 -nad5 -nad4 -nad4L -cob -nad6 -nad1 -rrnL -rrnS nad2 ]

n1(mod60)13g

[ cox1 cox2 cox3 nad3 -nad5 -nad4 -nad4L -cob -nad6 -nad1 -rrnL -rrnS nad2 ]

score = 313:

model 60:

-------------

R(0,1) R(0,9) R(0,10) R(0,17) R(0,18) R(0,24) R(0,25) R(0,28) R(0,n1) R(1,0) R(2,17) R(3,19) R(3,20) R(4,21) R(5,23) R(6,n3) R(7,n3) R(8,24) R(9,0) R(10,0) R(11,27) R(12,31) R(13,14) R(13,15) R(13,n4) R(14,13) R(15,13) R(15,n6) R(16,n5) R(17,0) R(17,2) R(18,0) R(18,19) R(19,3) R(19,18) R(19,22) R(20,3) R(20,21) R(21,4) R(21,20) R(22,19) R(22,23) R(23,5) R(23,22) R(24,0) R(24,8) R(25,0) R(25,26) R(26,25) R(26,27) R(27,11) R(27,26) R(28,0) R(28,29) R(29,28) R(29,30) R(30,29) R(30,31) R(31,12) R(31,30) R(n1,0) R(n1,n2) R(n1,n3) R(n2,n1) R(n2,n7) R(n3,6) R(n3,7) R(n3,n1) R(n4,13) R(n4,n5) R(n5,16) R(n5,n4) R(n6,15) R(n6,n7) R(n7,n2) R(n7,n6)

-------------------------------------------------------------------------------------------------------------

-> form outgroup SOL1_ALTER - B

n2(mod61)

[ cox1 cox2 atp8 atp6 cox3 nad3 -nad5 -nad4 -nad4L nad6 cob rrnS rrnL nad1 nad2 ]

n1(mod61)15g

[ cox1 cox2 atp8 atp6 cox3 nad3 -nad5 -nad4 -nad4L -cob -nad6 -nad1 -rrnL -rrnS nad2 ]

n1(mod61)13g

[ cox1 cox2 cox3 nad3 -nad5 -nad4 -nad4L -cob -nad6 -nad1 -rrnL -rrnS nad2 ]

score = 301:

model 61:

-------------

R(0,1) R(0,9) R(0,10) R(0,17) R(0,18) R(0,24) R(0,25) R(0,28) R(0,n1) R(1,0) R(2,17) R(3,19) R(3,20) R(4,21) R(5,23) R(6,n2) R(7,n2) R(8,24) R(9,0) R(10,0) R(11,27) R(12,31) R(13,14) R(13,15) R(13,n4) R(14,13) R(15,13) R(15,n7) R(16,n5) R(17,0) R(17,2) R(18,0) R(18,19) R(19,3) R(19,18) R(19,22) R(20,3) R(20,21) R(21,4) R(21,20) R(22,19) R(22,23) R(23,5) R(23,22) R(24,0) R(24,8) R(25,0) R(25,26) R(26,25) R(26,27) R(27,11) R(27,26) R(28,0) R(28,29) R(29,28) R(29,30) R(30,29) R(30,31) R(31,12) R(31,30) R(n1,0) R(n1,n2) R(n1,n3) R(n2,6) R(n2,7) R(n2,n1) R(n3,n1) R(n3,n6) R(n4,13) R(n4,n6) R(n5,16) R(n5,n7) R(n6,n3) R(n6,n4) R(n7,15) R(n7,n5)

-------------------------------------------------------------------------------------------------------------

-> form outgroup SOL1_ALTER - B

n2(mod62)

[ cox1 cox2 atp8 atp6 cox3 nad3 -nad5 -nad4 -nad4L nad6 cob rrnS rrnL nad1 nad2 ]

n1(mod62)15g

[ cox1 cox2 atp8 atp6 cox3 nad3 -nad5 -nad4 -nad4L -cob -nad6 -nad1 -rrnL -rrnS nad2 ]

n1(mod62)13g

[ cox1 cox2 cox3 nad3 -nad5 -nad4 -nad4L -cob -nad6 -nad1 -rrnL -rrnS nad2 ]

n4(mod62)

g1: [ cox1 cox2 nad1 nad3 -nad4 -nad5 -nad4L -cob -nad6 nad2 cox3 rrnS rrnL ]

g2: [ cox1 cox2 nad1 nad3 nad2 cox3 rrnS -nad5 -nad4 -nad4L -cob -nad6 rrnL ]

g3: [ cox1 cox2 nad1 nad6 cob nad4L nad5 nad4 nad3 nad2 cox3 rrnS rrnL ]

score = 277:

model 62:

-------------

R(0,1) R(0,9) R(0,10) R(0,17) R(0,18) R(0,24) R(0,25) R(0,28) R(0,n1) R(1,0) R(2,17) R(3,19) R(3,20) R(4,21) R(5,23) R(6,n2) R(7,n2) R(8,24) R(9,0) R(10,0) R(11,27) R(12,31) R(13,14) R(13,15) R(13,n4) R(14,13) R(15,13) R(16,n5) R(17,0) R(17,2) R(18,0) R(18,19) R(19,3) R(19,18) R(19,22) R(20,3) R(20,21) R(21,4) R(21,20) R(22,19) R(22,23) R(23,5) R(23,22) R(24,0) R(24,8) R(25,0) R(25,26) R(26,25) R(26,27) R(27,11) R(27,26) R(28,0) R(28,29) R(29,28) R(29,30) R(30,29) R(30,31) R(31,12) R(31,30) R(n1,0) R(n1,n2) R(n1,n3) R(n2,6) R(n2,7) R(n2,n1) R(n3,n1) R(n3,n6) R(n4,13) R(n4,n6) R(n4,n7) R(n5,16) R(n5,n7) R(n6,n3) R(n6,n4) R(n7,n4) R(n7,n5)

-------------------------------------------------------------------------------------------------------------

-> form outgroup SOL1_ALTER - B

n3(mod63)

[ cox1 cox2 atp8 atp6 cox3 nad3 -nad5 -nad4 -nad4L nad6 cob rrnS rrnL nad1 nad2 ]

n1(mod63)15g

[ cox1 cox2 atp8 atp6 cox3 nad3 -nad5 -nad4 -nad4L -cob -nad6 -nad1 -rrnL -rrnS nad2 ]

n1(mod63)13g

[ cox1 cox2 cox3 nad3 -nad5 -nad4 -nad4L -cob -nad6 -nad1 -rrnL -rrnS nad2 ]

n6(mod63)

[ cox1 cox2 nad1 nad3 nad2 cox3 rrnS -nad5 -nad4 -nad4L -cob -nad6 rrnL ]

n4(mod63)

[ cox1 cox2 nad1 nad3 nad2 cox3 rrnS nad5 nad4 -nad4L -cob -nad6 rrnL ]

score = 291:

model 63:

-------------

R(0,1) R(0,9) R(0,10) R(0,17) R(0,18) R(0,24) R(0,25) R(0,28) R(0,n1) R(1,0) R(2,17) R(3,19) R(3,20) R(4,21) R(5,23) R(6,n3) R(7,n3) R(8,24) R(9,0) R(10,0) R(11,27) R(12,31) R(13,14) R(13,n4) R(14,13) R(15,n6) R(16,n5) R(17,0) R(17,2) R(18,0) R(18,19) R(19,3) R(19,18) R(19,22) R(20,3) R(20,21) R(21,4) R(21,20) R(22,19) R(22,23) R(23,5) R(23,22) R(24,0) R(24,8) R(25,0) R(25,26) R(26,25) R(26,27) R(27,11) R(27,26) R(28,0) R(28,29) R(29,28) R(29,30) R(30,29) R(30,31) R(31,12) R(31,30) R(n1,0) R(n1,n2) R(n1,n3) R(n2,n1) R(n2,n7) R(n3,6) R(n3,7) R(n3,n1) R(n4,13) R(n4,n5) R(n4,n6) R(n5,16) R(n5,n4) R(n6,15) R(n6,n4) R(n6,n7) R(n7,n2) R(n7,n6)

-------------------------------------------------------------------------------------------------------------

-> form outgroup SOL1_ALTER - B

n3(mod64)

[ cox1 cox2 atp8 atp6 cox3 nad3 -nad5 -nad4 -nad4L nad6 cob rrnS rrnL nad1 nad2 ]

n1(mod64)15g

[ cox1 cox2 atp8 atp6 cox3 nad3 -nad5 -nad4 -nad4L -cob -nad6 -nad1 -rrnL -rrnS nad2 ]

n1(mod64)13g

[ cox1 cox2 cox3 nad3 -nad5 -nad4 -nad4L -cob -nad6 -nad1 -rrnL -rrnS nad2 ]

score = 301:

model 64:

-------------

R(0,1) R(0,9) R(0,10) R(0,17) R(0,18) R(0,24) R(0,25) R(0,28) R(0,n1) R(1,0) R(2,17) R(3,19) R(3,20) R(4,21) R(5,23) R(6,n3) R(7,n3) R(8,24) R(9,0) R(10,0) R(11,27) R(12,31) R(13,14) R(13,15) R(14,13) R(15,13) R(15,n4) R(15,n5) R(16,n6) R(17,0) R(17,2) R(18,0) R(18,19) R(19,3) R(19,18) R(19,22) R(20,3) R(20,21) R(21,4) R(21,20) R(22,19) R(22,23) R(23,5) R(23,22) R(24,0) R(24,8) R(25,0) R(25,26) R(26,25) R(26,27) R(27,11) R(27,26) R(28,0) R(28,29) R(29,28) R(29,30) R(30,29) R(30,31) R(31,12) R(31,30) R(n1,0) R(n1,n2) R(n1,n3) R(n2,n1) R(n2,n7) R(n3,6) R(n3,7) R(n3,n1) R(n4,15) R(n4,n6) R(n5,15) R(n5,n7) R(n6,16) R(n6,n4) R(n7,n2) R(n7,n5)

-------------------------------------------------------------------------------------------------------------

-> form outgroup SOL1_ALTER - B

n2(mod65)

[ cox1 cox2 atp8 atp6 cox3 nad3 -nad5 -nad4 -nad4L nad6 cob rrnS rrnL nad1 nad2 ]

n1(mod65)15g

[ cox1 cox2 atp8 atp6 cox3 nad3 -nad5 -nad4 -nad4L -cob -nad6 -nad1 -rrnL -rrnS nad2 ]

n1(mod65)13g

[ cox1 cox2 cox3 nad3 -nad5 -nad4 -nad4L -cob -nad6 -nad1 -rrnL -rrnS nad2 ]

n4(mod65)

[ cox1 cox2 nad1 nad3 nad2 cox3 rrnS -nad5 -nad4 -nad4L -cob -nad6 rrnL ]

score = 291:

model 65:

-------------

R(0,1) R(0,9) R(0,10) R(0,17) R(0,18) R(0,24) R(0,25) R(0,28) R(0,n1) R(1,0) R(2,17) R(3,19) R(3,20) R(4,21) R(5,23) R(6,n2) R(7,n2) R(8,24) R(9,0) R(10,0) R(11,27) R(12,31) R(13,14) R(13,15) R(14,13) R(15,13) R(15,n4) R(16,n5) R(17,0) R(17,2) R(18,0) R(18,19) R(19,3) R(19,18) R(19,22) R(20,3) R(20,21) R(21,4) R(21,20) R(22,19) R(22,23) R(23,5) R(23,22) R(24,0) R(24,8) R(25,0) R(25,26) R(26,25) R(26,27) R(27,11) R(27,26) R(28,0) R(28,29) R(29,28) R(29,30) R(30,29) R(30,31) R(31,12) R(31,30) R(n1,0) R(n1,n2) R(n1,n3) R(n2,6) R(n2,7) R(n2,n1) R(n3,n1) R(n3,n6) R(n4,15) R(n4,n6) R(n4,n7) R(n5,16) R(n5,n7) R(n6,n3) R(n6,n4) R(n7,n4) R(n7,n5)

-------------------------------------------------------------------------------------------------------------

-> form outgroup SOL9_ALTER - A

n1(mod66)

[ cox1 cox2 atp8 atp6 cox3 nad3 rrnS rrnL nad1 nad6 cob nad4L nad4 nad5 nad2 ]

n2(mod66)15g

[ cox1 cox2 atp8 atp6 cox3 nad3 rrnS -nad5 -nad4 -nad4L -cob -nad6 rrnL nad1 nad2 ]

n2(mod66)13g

[ cox1 cox2 cox3 nad3 rrnS -nad5 -nad4 -nad4L -cob -nad6 rrnL nad1 nad2 ]

score = 295:

model 66:

-------------

R(0,1) R(0,9) R(0,10) R(0,17) R(0,18) R(0,24) R(0,25) R(0,28) R(0,n1) R(1,0) R(2,17) R(3,19) R(3,20) R(4,21) R(5,23) R(6,n1) R(7,n4) R(8,24) R(9,0) R(10,0) R(11,27) R(12,31) R(13,14) R(13,15) R(14,13) R(15,13) R(15,n6) R(15,n7) R(16,n5) R(17,0) R(17,2) R(18,0) R(18,19) R(19,3) R(19,18) R(19,22) R(20,3) R(20,21) R(21,4) R(21,20) R(22,19) R(22,23) R(23,5) R(23,22) R(24,0) R(24,8) R(25,0) R(25,26) R(26,25) R(26,27) R(27,11) R(27,26) R(28,0) R(28,29) R(29,28) R(29,30) R(30,29) R(30,31) R(31,12) R(31,30) R(n1,0) R(n1,6) R(n1,n2) R(n2,n1) R(n2,n3) R(n2,n4) R(n3,n2) R(n3,n7) R(n4,7) R(n4,n2) R(n5,16) R(n5,n6) R(n6,15) R(n6,n5) R(n7,15) R(n7,n3)

-------------------------------------------------------------------------------------------------------------

-> form outgroup SOL9_ALTER - A

n1(mod67)

[ cox1 cox2 atp8 atp6 cox3 nad3 rrnS rrnL nad1 nad6 cob nad4L nad4 nad5 nad2 ]

n2(mod67)15g

[ cox1 cox2 atp8 atp6 cox3 nad3 rrnS -nad5 -nad4 -nad4L -cob -nad6 rrnL nad1 nad2 ]

n2(mod67)13g

[ cox1 cox2 cox3 nad3 rrnS -nad5 -nad4 -nad4L -cob -nad6 rrnL nad1 nad2 ]

n6(mod67)

[ cox1 cox2 nad1 nad3 nad2 cox3 rrnS -nad5 -nad4 -nad4L -cob -nad6 rrnL ]

score = 285:

model 67:

-------------

R(0,1) R(0,9) R(0,10) R(0,17) R(0,18) R(0,24) R(0,25) R(0,28) R(0,n1) R(1,0) R(2,17) R(3,19) R(3,20) R(4,21) R(5,23) R(6,n1) R(7,n4) R(8,24) R(9,0) R(10,0) R(11,27) R(12,31) R(13,14) R(13,15) R(14,13) R(15,13) R(15,n6) R(16,n5) R(17,0) R(17,2) R(18,0) R(18,19) R(19,3) R(19,18) R(19,22) R(20,3) R(20,21) R(21,4) R(21,20) R(22,19) R(22,23) R(23,5) R(23,22) R(24,0) R(24,8) R(25,0) R(25,26) R(26,25) R(26,27) R(27,11) R(27,26) R(28,0) R(28,29) R(29,28) R(29,30) R(30,29) R(30,31) R(31,12) R(31,30) R(n1,0) R(n1,6) R(n1,n2) R(n2,n1) R(n2,n3) R(n2,n4) R(n3,n2) R(n3,n6) R(n4,7) R(n4,n2) R(n5,16) R(n5,n7) R(n6,15) R(n6,n3) R(n6,n7) R(n7,n5) R(n7,n6)

-------------------------------------------------------------------------------------------------------------

-> form outgroup SOL9_ALTER - A

n1(mod68)

[ cox1 cox2 atp8 atp6 cox3 nad3 rrnS rrnL nad1 nad6 cob nad4L nad4 nad5 nad2 ]

n2(mod68)15g

[ cox1 cox2 atp8 atp6 cox3 nad3 rrnS -nad5 -nad4 -nad4L -cob -nad6 rrnL nad1 nad2 ]

n2(mod68)13g

[ cox1 cox2 cox3 nad3 rrnS -nad5 -nad4 -nad4L -cob -nad6 rrnL nad1 nad2 ]

score = 295:

model 68:

-------------

R(0,1) R(0,9) R(0,10) R(0,17) R(0,18) R(0,24) R(0,25) R(0,28) R(0,n1) R(1,0) R(2,17) R(3,19) R(3,20) R(4,21) R(5,23) R(6,n1) R(7,n3) R(8,24) R(9,0) R(10,0) R(11,27) R(12,31) R(13,14) R(13,15) R(13,n7) R(14,13) R(15,13) R(15,n6) R(16,n5) R(17,0) R(17,2) R(18,0) R(18,19) R(19,3) R(19,18) R(19,22) R(20,3) R(20,21) R(21,4) R(21,20) R(22,19) R(22,23) R(23,5) R(23,22) R(24,0) R(24,8) R(25,0) R(25,26) R(26,25) R(26,27) R(27,11) R(27,26) R(28,0) R(28,29) R(29,28) R(29,30) R(30,29) R(30,31) R(31,12) R(31,30) R(n1,0) R(n1,6) R(n1,n2) R(n2,n1) R(n2,n3) R(n2,n4) R(n3,7) R(n3,n2) R(n4,n2) R(n4,n7) R(n5,16) R(n5,n6) R(n6,15) R(n6,n5) R(n7,13) R(n7,n4)

-------------------------------------------------------------------------------------------------------------

-> form outgroup SOL9_ALTER - A

n1(mod69)

[ cox1 cox2 atp8 atp6 cox3 nad3 rrnS rrnL nad1 nad6 cob nad4L nad4 nad5 nad2 ]

n2(mod69)15g

[ cox1 cox2 atp8 atp6 cox3 nad3 rrnS -nad5 -nad4 -nad4L -cob -nad6 rrnL nad1 nad2 ]

n2(mod69)13g

[ cox1 cox2 cox3 nad3 rrnS -nad5 -nad4 -nad4L -cob -nad6 rrnL nad1 nad2 ]

score = 307:

model 69:

-------------

R(0,1) R(0,9) R(0,10) R(0,17) R(0,18) R(0,24) R(0,25) R(0,28) R(0,n1) R(1,0) R(2,17) R(3,19) R(3,20) R(4,21) R(5,23) R(6,n1) R(7,n4) R(8,24) R(9,0) R(10,0) R(11,27) R(12,31) R(13,14) R(13,15) R(13,n7) R(14,13) R(15,13) R(15,n6) R(16,n5) R(17,0) R(17,2) R(18,0) R(18,19) R(19,3) R(19,18) R(19,22) R(20,3) R(20,21) R(21,4) R(21,20) R(22,19) R(22,23) R(23,5) R(23,22) R(24,0) R(24,8) R(25,0) R(25,26) R(26,25) R(26,27) R(27,11) R(27,26) R(28,0) R(28,29) R(29,28) R(29,30) R(30,29) R(30,31) R(31,12) R(31,30) R(n1,0) R(n1,6) R(n1,n2) R(n2,n1) R(n2,n3) R(n2,n4) R(n3,n2) R(n3,n6) R(n4,7) R(n4,n2) R(n5,16) R(n5,n7) R(n6,15) R(n6,n3) R(n7,13) R(n7,n5)

-------------------------------------------------------------------------------------------------------------

-> form outgroup SOL9_ALTER - A

n1(mod70)

[ cox1 cox2 atp8 atp6 cox3 nad3 rrnS rrnL nad1 nad6 cob nad4L nad4 nad5 nad2 ]

n2(mod70)15g

[ cox1 cox2 atp8 atp6 cox3 nad3 rrnS -nad5 -nad4 -nad4L -cob -nad6 rrnL nad1 nad2 ]

n2(mod70)13g

[ cox1 cox2 cox3 nad3 rrnS -nad5 -nad4 -nad4L -cob -nad6 rrnL nad1 nad2 ]

n6(mod70)

[ cox1 cox2 nad1 nad3 nad2 cox3 rrnS nad5 nad4 -nad4L -cob -nad6 rrnL ]

score = 307:

model 70:

-------------

R(0,1) R(0,9) R(0,10) R(0,17) R(0,18) R(0,24) R(0,25) R(0,28) R(0,n1) R(1,0) R(2,17) R(3,19) R(3,20) R(4,21) R(5,23) R(6,n1) R(7,n4) R(8,24) R(9,0) R(10,0) R(11,27) R(12,31) R(13,14) R(13,15) R(13,n6) R(14,13) R(15,13) R(16,n5) R(17,0) R(17,2) R(18,0) R(18,19) R(19,3) R(19,18) R(19,22) R(20,3) R(20,21) R(21,4) R(21,20) R(22,19) R(22,23) R(23,5) R(23,22) R(24,0) R(24,8) R(25,0) R(25,26) R(26,25) R(26,27) R(27,11) R(27,26) R(28,0) R(28,29) R(29,28) R(29,30) R(30,29) R(30,31) R(31,12) R(31,30) R(n1,0) R(n1,6) R(n1,n2) R(n2,n1) R(n2,n3) R(n2,n4) R(n3,n2) R(n3,n7) R(n4,7) R(n4,n2) R(n5,16) R(n5,n6) R(n6,13) R(n6,n5) R(n6,n7) R(n7,n3) R(n7,n6)

-------------------------------------------------------------------------------------------------------------

-> form outgroup SOL9_ALTER - A

n1(mod71)

[ cox1 cox2 atp8 atp6 cox3 nad3 rrnS rrnL nad1 nad6 cob nad4L nad4 nad5 nad2 ]

n2(mod71)15g

[ cox1 cox2 atp8 atp6 cox3 nad3 rrnS -nad5 -nad4 -nad4L -cob -nad6 rrnL nad1 nad2 ]

n2(mod71)13g

[ cox1 cox2 cox3 nad3 rrnS -nad5 -nad4 -nad4L -cob -nad6 rrnL nad1 nad2 ]

n7(mod71)

[ cox1 cox2 nad1 nad3 nad2 cox3 rrnS -nad5 -nad4 -nad4L -cob -nad6 rrnL ]

score = 271:

model 71:

-------------

R(0,1) R(0,9) R(0,10) R(0,17) R(0,18) R(0,24) R(0,25) R(0,28) R(0,n1) R(1,0) R(2,17) R(3,19) R(3,20) R(4,21) R(5,23) R(6,n1) R(7,n4) R(8,24) R(9,0) R(10,0) R(11,27) R(12,31) R(13,14) R(13,15) R(13,n7) R(14,13) R(15,13) R(16,n5) R(17,0) R(17,2) R(18,0) R(18,19) R(19,3) R(19,18) R(19,22) R(20,3) R(20,21) R(21,4) R(21,20) R(22,19) R(22,23) R(23,5) R(23,22) R(24,0) R(24,8) R(25,0) R(25,26) R(26,25) R(26,27) R(27,11) R(27,26) R(28,0) R(28,29) R(29,28) R(29,30) R(30,29) R(30,31) R(31,12) R(31,30) R(n1,0) R(n1,6) R(n1,n2) R(n2,n1) R(n2,n3) R(n2,n4) R(n3,n2) R(n3,n7) R(n4,7) R(n4,n2) R(n5,16) R(n5,n6) R(n6,n5) R(n6,n7) R(n7,13) R(n7,n3) R(n7,n6)

-------------------------------------------------------------------------------------------------------------

-> form outgroup SOL9_ALTER - A

n1(mod72)

[ cox1 cox2 atp8 atp6 cox3 nad3 rrnS rrnL nad1 nad6 cob nad4L nad4 nad5 nad2 ]

n2(mod72)15g

[ cox1 cox2 atp8 atp6 cox3 nad3 rrnS -nad5 -nad4 -nad4L -cob -nad6 rrnL nad1 nad2 ]

n2(mod72)13g

[ cox1 cox2 cox3 nad3 rrnS -nad5 -nad4 -nad4L -cob -nad6 rrnL nad1 nad2 ]

score = 281:

model 72:

-------------

R(0,1) R(0,9) R(0,10) R(0,17) R(0,18) R(0,24) R(0,25) R(0,28) R(0,n1) R(1,0) R(2,17) R(3,19) R(3,20) R(4,21) R(5,23) R(6,n1) R(7,n3) R(8,24) R(9,0) R(10,0) R(11,27) R(12,31) R(13,14) R(13,15) R(13,n6) R(13,n7) R(14,13) R(15,13) R(16,n5) R(17,0) R(17,2) R(18,0) R(18,19) R(19,3) R(19,18) R(19,22) R(20,3) R(20,21) R(21,4) R(21,20) R(22,19) R(22,23) R(23,5) R(23,22) R(24,0) R(24,8) R(25,0) R(25,26) R(26,25) R(26,27) R(27,11) R(27,26) R(28,0) R(28,29) R(29,28) R(29,30) R(30,29) R(30,31) R(31,12) R(31,30) R(n1,0) R(n1,6) R(n1,n2) R(n2,n1) R(n2,n3) R(n2,n4) R(n3,7) R(n3,n2) R(n4,n2) R(n4,n7) R(n5,16) R(n5,n6) R(n6,13) R(n6,n5) R(n7,13) R(n7,n4)

-------------------------------------------------------------------------------------------------------------

-> form outgroup SOL9_ALTER - A

n1(mod73)

[ cox1 cox2 atp8 atp6 cox3 nad3 rrnS rrnL nad1 nad6 cob nad4L nad4 nad5 nad2 ]

n2(mod73)15g

[ cox1 cox2 atp8 atp6 cox3 nad3 rrnS -nad5 -nad4 -nad4L -cob -nad6 rrnL nad1 nad2 ]

n2(mod73)13g

[ cox1 cox2 cox3 nad3 rrnS -nad5 -nad4 -nad4L -cob -nad6 rrnL nad1 nad2 ]

n6(mod73)

[ cox1 cox2 nad1 nad3 nad2 cox3 rrnS -nad4L -nad4 -nad5 -cob -nad6 rrnL ]

score = 321:

model 73:

-------------

R(0,1) R(0,9) R(0,10) R(0,17) R(0,18) R(0,24) R(0,25) R(0,28) R(0,n1) R(1,0) R(2,17) R(3,19) R(3,20) R(4,21) R(5,23) R(6,n1) R(7,n4) R(8,24) R(9,0) R(10,0) R(11,27) R(12,31) R(13,14) R(13,n6) R(14,13) R(15,n6) R(15,n7) R(16,n5) R(17,0) R(17,2) R(18,0) R(18,19) R(19,3) R(19,18) R(19,22) R(20,3) R(20,21) R(21,4) R(21,20) R(22,19) R(22,23) R(23,5) R(23,22) R(24,0) R(24,8) R(25,0) R(25,26) R(26,25) R(26,27) R(27,11) R(27,26) R(28,0) R(28,29) R(29,28) R(29,30) R(30,29) R(30,31) R(31,12) R(31,30) R(n1,0) R(n1,6) R(n1,n2) R(n2,n1) R(n2,n3) R(n2,n4) R(n3,n2) R(n3,n7) R(n4,7) R(n4,n2) R(n5,16) R(n5,n6) R(n6,13) R(n6,15) R(n6,n5) R(n7,15) R(n7,n3)

-------------------------------------------------------------------------------------------------------------

-> form outgroup SOL9_ALTER - A

n1(mod74)

[ cox1 cox2 atp8 atp6 cox3 nad3 rrnS rrnL nad1 nad6 cob nad4L nad4 nad5 nad2 ]

n2(mod74)15g

[ cox1 cox2 atp8 atp6 cox3 nad3 rrnS -nad5 -nad4 -nad4L -cob -nad6 rrnL nad1 nad2 ]

n2(mod74)13g

[ cox1 cox2 cox3 nad3 rrnS -nad5 -nad4 -nad4L -cob -nad6 rrnL nad1 nad2 ]

n7(mod74)

[ cox1 cox2 nad1 nad3 nad2 cox3 rrnS -nad5 -nad4 -nad4L -cob -nad6 rrnL ]

score = 273:

model 74:

-------------

R(0,1) R(0,9) R(0,10) R(0,17) R(0,18) R(0,24) R(0,25) R(0,28) R(0,n1) R(1,0) R(2,17) R(3,19) R(3,20) R(4,21) R(5,23) R(6,n1) R(7,n3) R(8,24) R(9,0) R(10,0) R(11,27) R(12,31) R(13,14) R(13,n7) R(14,13) R(15,n6) R(15,n7) R(16,n5) R(17,0) R(17,2) R(18,0) R(18,19) R(19,3) R(19,18) R(19,22) R(20,3) R(20,21) R(21,4) R(21,20) R(22,19) R(22,23) R(23,5) R(23,22) R(24,0) R(24,8) R(25,0) R(25,26) R(26,25) R(26,27) R(27,11) R(27,26) R(28,0) R(28,29) R(29,28) R(29,30) R(30,29) R(30,31) R(31,12) R(31,30) R(n1,0) R(n1,6) R(n1,n2) R(n2,n1) R(n2,n3) R(n2,n4) R(n3,7) R(n3,n2) R(n4,n2) R(n4,n7) R(n5,16) R(n5,n6) R(n6,15) R(n6,n5) R(n7,13) R(n7,15) R(n7,n4)

-------------------------------------------------------------------------------------------------------------

-> form outgroup SOL9_ALTER - A

n1(mod75)

[ cox1 cox2 atp8 atp6 cox3 nad3 rrnS rrnL nad1 nad6 cob nad4L nad4 nad5 nad2 ]

n2(mod75)15g

[ cox1 cox2 atp8 atp6 cox3 nad3 rrnS -nad5 -nad4 -nad4L -cob -nad6 rrnL nad1 nad2 ]

n2(mod75)13g

[ cox1 cox2 cox3 nad3 rrnS -nad5 -nad4 -nad4L -cob -nad6 rrnL nad1 nad2 ]

n6(mod75)

[ cox1 cox2 nad1 nad3 nad2 cox3 rrnS -nad5 -nad4 -nad4L -cob -nad6 rrnL ]

score = 259:

model 75:

-------------

R(0,1) R(0,9) R(0,10) R(0,17) R(0,18) R(0,24) R(0,25) R(0,28) R(0,n1) R(1,0) R(2,17) R(3,19) R(3,20) R(4,21) R(5,23) R(6,n1) R(7,n4) R(8,24) R(9,0) R(10,0) R(11,27) R(12,31) R(13,14) R(13,n6) R(14,13) R(15,n6) R(16,n5) R(17,0) R(17,2) R(18,0) R(18,19) R(19,3) R(19,18) R(19,22) R(20,3) R(20,21) R(21,4) R(21,20) R(22,19) R(22,23) R(23,5) R(23,22) R(24,0) R(24,8) R(25,0) R(25,26) R(26,25) R(26,27) R(27,11) R(27,26) R(28,0) R(28,29) R(29,28) R(29,30) R(30,29) R(30,31) R(31,12) R(31,30) R(n1,0) R(n1,6) R(n1,n2) R(n2,n1) R(n2,n3) R(n2,n4) R(n3,n2) R(n3,n6) R(n4,7) R(n4,n2) R(n5,16) R(n5,n7) R(n6,13) R(n6,15) R(n6,n3) R(n6,n7) R(n7,n5) R(n7,n6)

-------------------------------------------------------------------------------------------------------------

-> form outgroup SOL9_ALTER - A

n1(mod76)

[ cox1 cox2 atp8 atp6 cox3 nad3 rrnS rrnL nad1 nad6 cob nad4L nad4 nad5 nad2 ]

n2(mod76)15g

[ cox1 cox2 atp8 atp6 cox3 nad3 rrnS -nad5 -nad4 -nad4L -cob -nad6 rrnL nad1 nad2 ]

n2(mod76)13g

[ cox1 cox2 cox3 nad3 rrnS -nad5 -nad4 -nad4L -cob -nad6 rrnL nad1 nad2 ]

n6(mod76)

[ cox1 cox2 nad1 nad3 nad2 cox3 rrnS -nad5 -nad4 -nad4L -cob -nad6 rrnL ]

n7(mod76)

[ cox1 cox2 nad1 nad3 nad2 cox3 rrnS nad5 nad4 -nad4L -cob -nad6 rrnL ]

score = 285:

model 76:

-------------

R(0,1) R(0,9) R(0,10) R(0,17) R(0,18) R(0,24) R(0,25) R(0,28) R(0,n1) R(1,0) R(2,17) R(3,19) R(3,20) R(4,21) R(5,23) R(6,n1) R(7,n4) R(8,24) R(9,0) R(10,0) R(11,27) R(12,31) R(13,14) R(13,n7) R(14,13) R(15,n6) R(16,n5) R(17,0) R(17,2) R(18,0) R(18,19) R(19,3) R(19,18) R(19,22) R(20,3) R(20,21) R(21,4) R(21,20) R(22,19) R(22,23) R(23,5) R(23,22) R(24,0) R(24,8) R(25,0) R(25,26) R(26,25) R(26,27) R(27,11) R(27,26) R(28,0) R(28,29) R(29,28) R(29,30) R(30,29) R(30,31) R(31,12) R(31,30) R(n1,0) R(n1,6) R(n1,n2) R(n2,n1) R(n2,n3) R(n2,n4) R(n3,n2) R(n3,n6) R(n4,7) R(n4,n2) R(n5,16) R(n5,n7) R(n6,15) R(n6,n3) R(n6,n7) R(n7,13) R(n7,n5) R(n7,n6)

-------------------------------------------------------------------------------------------------------------

-> form outgroup SOL9_ALTER - A

n1(mod77)

[ cox1 cox2 atp8 atp6 cox3 nad3 rrnS rrnL nad1 nad6 cob nad4L nad4 nad5 nad2 ]

n2(mod77)15g

[ cox1 cox2 atp8 atp6 cox3 nad3 rrnS -nad5 -nad4 -nad4L -cob -nad6 rrnL nad1 nad2 ]

n2(mod77)13g

[ cox1 cox2 cox3 nad3 rrnS -nad5 -nad4 -nad4L -cob -nad6 rrnL nad1 nad2 ]

n6(mod77)

[ cox1 cox2 nad1 nad3 nad2 cox3 rrnS -nad4L -nad4 -nad5 -cob -nad6 rrnL ]

score = 295:

model 77:

-------------

R(0,1) R(0,9) R(0,10) R(0,17) R(0,18) R(0,24) R(0,25) R(0,28) R(0,n1) R(1,0) R(2,17) R(3,19) R(3,20) R(4,21) R(5,23) R(6,n1) R(7,n3) R(8,24) R(9,0) R(10,0) R(11,27) R(12,31) R(13,14) R(13,n6) R(13,n7) R(14,13) R(15,n6) R(16,n5) R(17,0) R(17,2) R(18,0) R(18,19) R(19,3) R(19,18) R(19,22) R(20,3) R(20,21) R(21,4) R(21,20) R(22,19) R(22,23) R(23,5) R(23,22) R(24,0) R(24,8) R(25,0) R(25,26) R(26,25) R(26,27) R(27,11) R(27,26) R(28,0) R(28,29) R(29,28) R(29,30) R(30,29) R(30,31) R(31,12) R(31,30) R(n1,0) R(n1,6) R(n1,n2) R(n2,n1) R(n2,n3) R(n2,n4) R(n3,7) R(n3,n2) R(n4,n2) R(n4,n7) R(n5,16) R(n5,n6) R(n6,13) R(n6,15) R(n6,n5) R(n7,13) R(n7,n4)

-------------------------------------------------------------------------------------------------------------

-> form outgroup SOL9_ALTER - A

n1(mod78)

[ cox1 cox2 atp8 atp6 cox3 nad3 rrnS rrnL nad1 nad6 cob nad4L nad4 nad5 nad2 ]

n2(mod78)15g

[ cox1 cox2 atp8 atp6 cox3 nad3 rrnS -nad5 -nad4 -nad4L -cob -nad6 rrnL nad1 nad2 ]

n2(mod78)13g

[ cox1 cox2 cox3 nad3 rrnS -nad5 -nad4 -nad4L -cob -nad6 rrnL nad1 nad2 ]

n6(mod78)

[ cox1 cox2 nad1 nad3 nad2 cox3 rrnS -nad5 -nad4 -nad4L -cob -nad6 rrnL ]

score = 271:

model 78:

-------------

R(0,1) R(0,9) R(0,10) R(0,17) R(0,18) R(0,24) R(0,25) R(0,28) R(0,n1) R(1,0) R(2,17) R(3,19) R(3,20) R(4,21) R(5,23) R(6,n1) R(7,n4) R(8,24) R(9,0) R(10,0) R(11,27) R(12,31) R(13,14) R(13,n6) R(13,n7) R(14,13) R(15,n6) R(16,n5) R(17,0) R(17,2) R(18,0) R(18,19) R(19,3) R(19,18) R(19,22) R(20,3) R(20,21) R(21,4) R(21,20) R(22,19) R(22,23) R(23,5) R(23,22) R(24,0) R(24,8) R(25,0) R(25,26) R(26,25) R(26,27) R(27,11) R(27,26) R(28,0) R(28,29) R(29,28) R(29,30) R(30,29) R(30,31) R(31,12) R(31,30) R(n1,0) R(n1,6) R(n1,n2) R(n2,n1) R(n2,n3) R(n2,n4) R(n3,n2) R(n3,n6) R(n4,7) R(n4,n2) R(n5,16) R(n5,n7) R(n6,13) R(n6,15) R(n6,n3) R(n7,13) R(n7,n5)

-------------------------------------------------------------------------------------------------------------

-> form outgroup SOL4_ALTER - B

n4(mod79)

[ cox1 cox2 atp8 atp6 cox3 nad3 -nad5 -nad4 -nad4L nad6 cob rrnS rrnL nad1 nad2 ]

n2(mod79)15g

[ cox1 cox2 atp8 atp6 cox3 -nad5 -nad4 -nad4L nad6 cob rrnS rrnL nad1 nad3 nad2 ]

n2(mod79)13g

[ cox1 cox2 cox3 -nad5 -nad4 -nad4L nad6 cob rrnS rrnL nad1 nad3 nad2 ]

n6(mod79)

[ cox1 cox2 nad1 nad3 nad2 cox3 rrnS -nad4 -nad5 -cob -nad6 nad4L rrnL ]

score = 327:

model 79:

-------------

R(0,1) R(0,9) R(0,10) R(0,17) R(0,18) R(0,24) R(0,25) R(0,28) R(0,n1) R(1,0) R(2,17) R(3,19) R(3,20) R(4,21) R(5,23) R(6,n4) R(7,n4) R(8,24) R(9,0) R(10,0) R(11,27) R(12,31) R(13,14) R(13,15) R(13,n6) R(14,13) R(15,13) R(16,n5) R(17,0) R(17,2) R(18,0) R(18,19) R(19,3) R(19,18) R(19,22) R(20,3) R(20,21) R(21,4) R(21,20) R(22,19) R(22,23) R(23,5) R(23,22) R(24,0) R(24,8) R(25,0) R(25,26) R(26,25) R(26,27) R(27,11) R(27,26) R(28,0) R(28,29) R(29,28) R(29,30) R(30,29) R(30,31) R(31,12) R(31,30) R(n1,0) R(n1,n2) R(n2,n1) R(n2,n3) R(n2,n4) R(n3,n2) R(n3,n7) R(n4,6) R(n4,7) R(n4,n2) R(n5,16) R(n5,n6) R(n6,13) R(n6,n5) R(n6,n7) R(n7,n3) R(n7,n6)

-------------------------------------------------------------------------------------------------------------

-> form outgroup SOL2_ALTER - B

n1(mod80)15g

[ cox1 cox2 atp8 atp6 cox3 nad3 rrnS rrnL nad1 nad6 cob nad4L nad4 nad5 nad2 ]

n1(mod80)13g

[ cox1 cox2 cox3 nad3 rrnS rrnL nad1 nad6 cob nad4L nad4 nad5 nad2 ]

n4(mod80)

g1: [ cox1 cox2 nad1 nad3 nad2 cox3 rrnS -nad5 -nad4 -nad4L -cob -nad6 rrnL ]

g2: [ cox1 cox2 nad1 nad6 cob nad4L nad5 nad4 nad3 nad2 cox3 rrnS rrnL ]

g3: [ cox1 cox2 nad1 nad3 nad6 cob nad4L nad5 nad4 nad2 cox3 rrnS rrnL ]

score = 277:

model 80:

-------------

R(0,1) R(0,9) R(0,10) R(0,17) R(0,18) R(0,24) R(0,25) R(0,28) R(0,n1) R(1,0) R(2,17) R(3,19) R(3,20) R(4,21) R(5,23) R(6,n1) R(6,n7) R(7,n7) R(8,24) R(9,0) R(10,0) R(11,27) R(12,31) R(13,14) R(13,15) R(13,n4) R(14,13) R(15,13) R(16,n5) R(17,0) R(17,2) R(18,0) R(18,19) R(19,3) R(19,18) R(19,22) R(20,3) R(20,21) R(21,4) R(21,20) R(22,19) R(22,23) R(23,5) R(23,22) R(24,0) R(24,8) R(25,0) R(25,26) R(26,25) R(26,27) R(27,11) R(27,26) R(28,0) R(28,29) R(29,28) R(29,30) R(30,29) R(30,31) R(31,12) R(31,30) R(n1,0) R(n1,6) R(n1,n2) R(n2,n1) R(n2,n3) R(n3,n2) R(n3,n4) R(n4,13) R(n4,n3) R(n4,n6) R(n5,16) R(n5,n6) R(n6,n4) R(n6,n5) R(n7,6) R(n7,7)

-------------------------------------------------------------------------------------------------------------

-> form outgroup SOL2_ALTER - B

n1(mod81)15g

[ cox1 cox2 atp8 atp6 cox3 nad3 rrnS rrnL nad1 nad6 cob nad4L nad4 nad5 nad2 ]

n1(mod81)13g

[ cox1 cox2 cox3 nad3 rrnS rrnL nad1 nad6 cob nad4L nad4 nad5 nad2 ]

score = 287:

model 81:

-------------

R(0,1) R(0,9) R(0,10) R(0,17) R(0,18) R(0,24) R(0,25) R(0,28) R(0,n1) R(1,0) R(2,17) R(3,19) R(3,20) R(4,21) R(5,23) R(6,n1) R(6,n6) R(7,n6) R(8,24) R(9,0) R(10,0) R(11,27) R(12,31) R(13,14) R(13,15) R(13,n4) R(13,n7) R(14,13) R(15,13) R(16,n5) R(17,0) R(17,2) R(18,0) R(18,19) R(19,3) R(19,18) R(19,22) R(20,3) R(20,21) R(21,4) R(21,20) R(22,19) R(22,23) R(23,5) R(23,22) R(24,0) R(24,8) R(25,0) R(25,26) R(26,25) R(26,27) R(27,11) R(27,26) R(28,0) R(28,29) R(29,28) R(29,30) R(30,29) R(30,31) R(31,12) R(31,30) R(n1,0) R(n1,6) R(n1,n2) R(n2,n1) R(n2,n3) R(n3,n2) R(n3,n4) R(n4,13) R(n4,n3) R(n5,16) R(n5,n7) R(n6,6) R(n6,7) R(n7,13) R(n7,n5)

-------------------------------------------------------------------------------------------------------------

-> form outgroup SOL2_ALTER - B

n1(mod82)15g

[ cox1 cox2 atp8 atp6 cox3 nad3 rrnS rrnL nad1 nad6 cob nad4L nad4 nad5 nad2 ]

n1(mod82)13g

[ cox1 cox2 cox3 nad3 rrnS rrnL nad1 nad6 cob nad4L nad4 nad5 nad2 ]

score = 301:

model 82:

-------------

R(0,1) R(0,9) R(0,10) R(0,17) R(0,18) R(0,24) R(0,25) R(0,28) R(0,n1) R(1,0) R(2,17) R(3,19) R(3,20) R(4,21) R(5,23) R(6,n1) R(6,n6) R(7,n6) R(8,24) R(9,0) R(10,0) R(11,27) R(12,31) R(13,14) R(13,15) R(13,n4) R(14,13) R(15,13) R(15,n7) R(16,n5) R(17,0) R(17,2) R(18,0) R(18,19) R(19,3) R(19,18) R(19,22) R(20,3) R(20,21) R(21,4) R(21,20) R(22,19) R(22,23) R(23,5) R(23,22) R(24,0) R(24,8) R(25,0) R(25,26) R(26,25) R(26,27) R(27,11) R(27,26) R(28,0) R(28,29) R(29,28) R(29,30) R(30,29) R(30,31) R(31,12) R(31,30) R(n1,0) R(n1,6) R(n1,n2) R(n2,n1) R(n2,n3) R(n3,n2) R(n3,n4) R(n4,13) R(n4,n3) R(n5,16) R(n5,n7) R(n6,6) R(n6,7) R(n7,15) R(n7,n5)

-------------------------------------------------------------------------------------------------------------

-> form outgroup SOL2_ALTER - B

n1(mod83)15g

[ cox1 cox2 atp8 atp6 cox3 nad3 rrnS rrnL nad1 nad6 cob nad4L nad4 nad5 nad2 ]

n1(mod83)13g

[ cox1 cox2 cox3 nad3 rrnS rrnL nad1 nad6 cob nad4L nad4 nad5 nad2 ]

n4(mod83)

[ cox1 cox2 nad1 nad3 nad2 cox3 rrnS -nad5 -nad4 -nad4L -cob -nad6 rrnL ]

score = 291:

model 83:

-------------

R(0,1) R(0,9) R(0,10) R(0,17) R(0,18) R(0,24) R(0,25) R(0,28) R(0,n1) R(1,0) R(2,17) R(3,19) R(3,20) R(4,21) R(5,23) R(6,n1) R(6,n7) R(7,n7) R(8,24) R(9,0) R(10,0) R(11,27) R(12,31) R(13,14) R(13,15) R(14,13) R(15,13) R(15,n4) R(16,n5) R(17,0) R(17,2) R(18,0) R(18,19) R(19,3) R(19,18) R(19,22) R(20,3) R(20,21) R(21,4) R(21,20) R(22,19) R(22,23) R(23,5) R(23,22) R(24,0) R(24,8) R(25,0) R(25,26) R(26,25) R(26,27) R(27,11) R(27,26) R(28,0) R(28,29) R(29,28) R(29,30) R(30,29) R(30,31) R(31,12) R(31,30) R(n1,0) R(n1,6) R(n1,n2) R(n2,n1) R(n2,n3) R(n3,n2) R(n3,n4) R(n4,15) R(n4,n3) R(n4,n6) R(n5,16) R(n5,n6) R(n6,n4) R(n6,n5) R(n7,6) R(n7,7)

-------------------------------------------------------------------------------------------------------------

-> form outgroup SOL2_ALTER - B

n1(mod84)15g

[ cox1 cox2 atp8 atp6 cox3 nad3 rrnS rrnL nad1 nad6 cob nad4L nad4 nad5 nad2 ]

n1(mod84)13g

[ cox1 cox2 cox3 nad3 rrnS rrnL nad1 nad6 cob nad4L nad4 nad5 nad2 ]

score = 313:

model 84:

-------------

R(0,1) R(0,9) R(0,10) R(0,17) R(0,18) R(0,24) R(0,25) R(0,28) R(0,n1) R(1,0) R(2,17) R(3,19) R(3,20) R(4,21) R(5,23) R(6,n1) R(6,n6) R(7,n6) R(8,24) R(9,0) R(10,0) R(11,27) R(12,31) R(13,14) R(13,15) R(13,n7) R(14,13) R(15,13) R(15,n4) R(16,n5) R(17,0) R(17,2) R(18,0) R(18,19) R(19,3) R(19,18) R(19,22) R(20,3) R(20,21) R(21,4) R(21,20) R(22,19) R(22,23) R(23,5) R(23,22) R(24,0) R(24,8) R(25,0) R(25,26) R(26,25) R(26,27) R(27,11) R(27,26) R(28,0) R(28,29) R(29,28) R(29,30) R(30,29) R(30,31) R(31,12) R(31,30) R(n1,0) R(n1,6) R(n1,n2) R(n2,n1) R(n2,n3) R(n3,n2) R(n3,n4) R(n4,15) R(n4,n3) R(n5,16) R(n5,n7) R(n6,6) R(n6,7) R(n7,13) R(n7,n5)

-------------------------------------------------------------------------------------------------------------

-> form outgroup SOL2_ALTER - B

n1(mod85)15g

[ cox1 cox2 atp8 atp6 cox3 nad3 rrnS rrnL nad1 nad6 cob nad4L nad4 nad5 nad2 ]

n1(mod85)13g

[ cox1 cox2 cox3 nad3 rrnS rrnL nad1 nad6 cob nad4L nad4 nad5 nad2 ]

score = 301:

model 85:

-------------

R(0,1) R(0,9) R(0,10) R(0,17) R(0,18) R(0,24) R(0,25) R(0,28) R(0,n1) R(1,0) R(2,17) R(3,19) R(3,20) R(4,21) R(5,23) R(6,n1) R(6,n6) R(7,n6) R(8,24) R(9,0) R(10,0) R(11,27) R(12,31) R(13,14) R(13,15) R(14,13) R(15,13) R(15,n4) R(15,n7) R(16,n5) R(17,0) R(17,2) R(18,0) R(18,19) R(19,3) R(19,18) R(19,22) R(20,3) R(20,21) R(21,4) R(21,20) R(22,19) R(22,23) R(23,5) R(23,22) R(24,0) R(24,8) R(25,0) R(25,26) R(26,25) R(26,27) R(27,11) R(27,26) R(28,0) R(28,29) R(29,28) R(29,30) R(30,29) R(30,31) R(31,12) R(31,30) R(n1,0) R(n1,6) R(n1,n2) R(n2,n1) R(n2,n3) R(n3,n2) R(n3,n4) R(n4,15) R(n4,n3) R(n5,16) R(n5,n7) R(n6,6) R(n6,7) R(n7,15) R(n7,n5)

-------------------------------------------------------------------------------------------------------------

-> form outgroup SOL2_ALTER - B

-> a new form for chaetognates group (different from the 13 usual forms), possible with the specific value for n1

n1(mod86)15g

[ cox1 cox2 atp8 atp6 -nad5 -nad4 -nad4L nad6 cob -nad1 -rrnL -rrnS cox3 nad3 nad2 ]

n1(mod86)13g

[ cox1 cox2 -nad5 -nad4 -nad4L nad6 cob -nad1 -rrnL -rrnS cox3 nad3 nad2 ]

n4(mod86)

[ cox1 cox2 nad1 nad3 nad2 cox3 -nad4 -nad5 -cob -nad6 rrnS rrnL -nad4L ]

score = 303:

model 86:

-------------

R(0,1) R(0,9) R(0,10) R(0,17) R(0,18) R(0,24) R(0,25) R(0,28) R(0,n1) R(1,0) R(2,17) R(3,19) R(3,20) R(4,21) R(5,23) R(6,n1) R(6,n7) R(7,n7) R(8,24) R(9,0) R(10,0) R(11,27) R(12,31) R(13,14) R(13,15) R(13,n6) R(14,13) R(15,13) R(16,n5) R(17,0) R(17,2) R(18,0) R(18,19) R(19,3) R(19,18) R(19,22) R(20,3) R(20,21) R(21,4) R(21,20) R(22,19) R(22,23) R(23,5) R(23,22) R(24,0) R(24,8) R(25,0) R(25,26) R(26,25) R(26,27) R(27,11) R(27,26) R(28,0) R(28,29) R(29,28) R(29,30) R(30,29) R(30,31) R(31,12) R(31,30) R(n1,0) R(n1,6) R(n1,n2) R(n2,n1) R(n2,n3) R(n3,n2) R(n3,n4) R(n4,n3) R(n4,n5) R(n4,n6) R(n5,16) R(n5,n4) R(n6,13) R(n6,n4) R(n7,6) R(n7,7)

-------------------------------------------------------------------------------------------------------------

-> form outgroup SOL2_ALTER - B

-> a new form for chaetognates group (different from the 13 usual forms), possible with the specific value for n1

n1(mod87)15g

[ cox1 cox2 atp8 atp6 -nad5 -nad4 -nad4L nad6 cob -nad1 -rrnL -rrnS cox3 nad3 nad2 ]

n1(mod87)13g

[ cox1 cox2 -nad5 -nad4 -nad4L nad6 cob -nad1 -rrnL -rrnS cox3 nad3 nad2 ]

n5(mod87)

[ cox1 cox2 nad1 nad3 nad2 cox3 rrnS rrnL nad6 cob nad5 nad4 -nad4L ]

score = 339:

model 87:

-------------

R(0,1) R(0,9) R(0,10) R(0,17) R(0,18) R(0,24) R(0,25) R(0,28) R(0,n1) R(1,0) R(2,17) R(3,19) R(3,20) R(4,21) R(5,23) R(6,n1) R(6,n6) R(7,n6) R(8,24) R(9,0) R(10,0) R(11,27) R(12,31) R(13,14) R(13,15) R(13,n7) R(14,13) R(15,13) R(16,n5) R(17,0) R(17,2) R(18,0) R(18,19) R(19,3) R(19,18) R(19,22) R(20,3) R(20,21) R(21,4) R(21,20) R(22,19) R(22,23) R(23,5) R(23,22) R(24,0) R(24,8) R(25,0) R(25,26) R(26,25) R(26,27) R(27,11) R(27,26) R(28,0) R(28,29) R(29,28) R(29,30) R(30,29) R(30,31) R(31,12) R(31,30) R(n1,0) R(n1,6) R(n1,n2) R(n2,n1) R(n2,n3) R(n3,n2) R(n3,n4) R(n4,n3) R(n4,n5) R(n5,16) R(n5,n4) R(n5,n7) R(n6,6) R(n6,7) R(n7,13) R(n7,n5)

-------------------------------------------------------------------------------------------------------------

-> form outgroup SOL2_ALTER - B

n1(mod88)

g1[ cox1 cox2 cox3 nad3 rrnS rrnL nad1 nad6 cob nad4L nad4 nad5 nad2 ]

g2[ cox1 cox2 -nad5 -nad4 -nad4L nad6 cob -nad1 -rrnL -rrnS cox3 nad3 nad2 ]

n7(mod88)

for n1-g1:

g1: [ cox1 cox2 nad1 nad3 nad2 cox3 rrnS -nad4 -nad5 -cob -nad6 -nad4L rrnL ]

g2: [ cox1 cox2 nad1 nad3 nad2 cox3 rrnS nad5 nad4 -nad4L -cob -nad6 rrnL ]

g3: [ cox1 cox2 nad1 nad3 nad2 cox3 rrnS rrnL nad6 cob nad4L nad5 nad4 ]

g4: [ cox1 cox2 nad1 nad3 nad2 cox3 rrnS nad6 cob nad4L -nad4 -nad5 rrnL ]

for n1-g2:

g1: [ cox1 cox2 nad1 nad3 nad2 cox3 rrnS -nad4 -nad5 -cob -nad6 rrnL -nad4L ]

g2: [ cox1 cox2 nad1 nad3 nad2 cox3 rrnS rrnL nad6 cob nad4L nad5 nad4 ]

g3: [ cox1 cox2 nad1 nad3 nad2 cox3 rrnS -nad4 -nad5 -cob -nad6 nad4L rrnL ]

g4: [ cox1 cox2 nad1 nad3 nad2 cox3 rrnS -nad4 -nad5 -rrnL -nad4L -cob -nad6 ]

score = 313:

model 88:

-------------

R(0,1) R(0,9) R(0,10) R(0,17) R(0,18) R(0,24) R(0,25) R(0,28) R(0,n1) R(1,0) R(2,17) R(3,19) R(3,20) R(4,21) R(5,23) R(6,n1) R(6,n6) R(7,n6) R(8,24) R(9,0) R(10,0) R(11,27) R(12,31) R(13,14) R(13,15) R(13,n7) R(14,13) R(15,13) R(16,n5) R(17,0) R(17,2) R(18,0) R(18,19) R(19,3) R(19,18) R(19,22) R(20,3) R(20,21) R(21,4) R(21,20) R(22,19) R(22,23) R(23,5) R(23,22) R(24,0) R(24,8) R(25,0) R(25,26) R(26,25) R(26,27) R(27,11) R(27,26) R(28,0) R(28,29) R(29,28) R(29,30) R(30,29) R(30,31) R(31,12) R(31,30) R(n1,0) R(n1,6) R(n1,n2) R(n2,n1) R(n2,n3) R(n3,n2) R(n3,n4) R(n4,n3) R(n4,n7) R(n5,16) R(n5,n7) R(n6,6) R(n6,7) R(n7,13) R(n7,n4) R(n7,n5)

-------------------------------------------------------------------------------------------------------------

-> form outgroup SOL2_ALTER - B

n1(mod89)15g

[ cox1 cox2 atp8 atp6 cox3 nad3 rrnS rrnL nad1 nad6 cob nad4L nad4 nad5 nad2 ]

n1(mod89)13g

[ cox1 cox2 cox3 nad3 rrnS rrnL nad1 nad6 cob nad4L nad4 nad5 nad2 ]

n4(mod89)

[ cox1 cox2 nad1 nad3 nad2 cox3 rrnS -nad5 -nad4 -nad4L -cob -nad6 rrnL ]

score = 265:

model 89:

-------------

R(0,1) R(0,9) R(0,10) R(0,17) R(0,18) R(0,24) R(0,25) R(0,28) R(0,n1) R(1,0) R(2,17) R(3,19) R(3,20) R(4,21) R(5,23) R(6,n1) R(6,n7) R(7,n7) R(8,24) R(9,0) R(10,0) R(11,27) R(12,31) R(13,14) R(13,n4) R(14,13) R(15,n4) R(16,n5) R(17,0) R(17,2) R(18,0) R(18,19) R(19,3) R(19,18) R(19,22) R(20,3) R(20,21) R(21,4) R(21,20) R(22,19) R(22,23) R(23,5) R(23,22) R(24,0) R(24,8) R(25,0) R(25,26) R(26,25) R(26,27) R(27,11) R(27,26) R(28,0) R(28,29) R(29,28) R(29,30) R(30,29) R(30,31) R(31,12) R(31,30) R(n1,0) R(n1,6) R(n1,n2) R(n2,n1) R(n2,n3) R(n3,n2) R(n3,n4) R(n4,13) R(n4,15) R(n4,n3) R(n4,n6) R(n5,16) R(n5,n6) R(n6,n4) R(n6,n5) R(n7,6) R(n7,7)

-------------------------------------------------------------------------------------------------------------

-> form outgroup SOL2_ALTER - B

n1(mod90)15g

[ cox1 cox2 atp8 atp6 cox3 nad3 rrnS rrnL nad1 nad6 cob nad4L nad4 nad5 nad2 ]

n1(mod90)13g

[ cox1 cox2 cox3 nad3 rrnS rrnL nad1 nad6 cob nad4L nad4 nad5 nad2 ]

n4(mod90)

[ cox1 cox2 nad1 nad3 nad2 cox3 rrnS -nad5 -nad4 -nad4L -cob -nad6 rrnL ]

score = 277:

model 90:

-------------

R(0,1) R(0,9) R(0,10) R(0,17) R(0,18) R(0,24) R(0,25) R(0,28) R(0,n1) R(1,0) R(2,17) R(3,19) R(3,20) R(4,21) R(5,23) R(6,n1) R(6,n6) R(7,n6) R(8,24) R(9,0) R(10,0) R(11,27) R(12,31) R(13,14) R(13,n4) R(13,n7) R(14,13) R(15,n4) R(16,n5) R(17,0) R(17,2) R(18,0) R(18,19) R(19,3) R(19,18) R(19,22) R(20,3) R(20,21) R(21,4) R(21,20) R(22,19) R(22,23) R(23,5) R(23,22) R(24,0) R(24,8) R(25,0) R(25,26) R(26,25) R(26,27) R(27,11) R(27,26) R(28,0) R(28,29) R(29,28) R(29,30) R(30,29) R(30,31) R(31,12) R(31,30) R(n1,0) R(n1,6) R(n1,n2) R(n2,n1) R(n2,n3) R(n3,n2) R(n3,n4) R(n4,13) R(n4,15) R(n4,n3) R(n5,16) R(n5,n7) R(n6,6) R(n6,7) R(n7,13) R(n7,n5)

-------------------------------------------------------------------------------------------------------------

-> form outgroup SOL2_ALTER - B

n1(mod91)15g

[ cox1 cox2 atp8 atp6 cox3 nad3 rrnS rrnL nad1 nad6 cob nad4L nad4 nad5 nad2 ]

n1(mod91)13g

[ cox1 cox2 cox3 nad3 rrnS rrnL nad1 nad6 cob nad4L nad4 nad5 nad2 ]

n4(mod91)

[ cox1 cox2 nad1 nad3 nad2 cox3 rrnS -nad5 -nad4 -nad4L -cob -nad6 rrnL ]

score = 279:

model 91:

-------------

R(0,1) R(0,9) R(0,10) R(0,17) R(0,18) R(0,24) R(0,25) R(0,28) R(0,n1) R(1,0) R(2,17) R(3,19) R(3,20) R(4,21) R(5,23) R(6,n1) R(6,n6) R(7,n6) R(8,24) R(9,0) R(10,0) R(11,27) R(12,31) R(13,14) R(13,n4) R(14,13) R(15,n4) R(15,n7) R(16,n5) R(17,0) R(17,2) R(18,0) R(18,19) R(19,3) R(19,18) R(19,22) R(20,3) R(20,21) R(21,4) R(21,20) R(22,19) R(22,23) R(23,5) R(23,22) R(24,0) R(24,8) R(25,0) R(25,26) R(26,25) R(26,27) R(27,11) R(27,26) R(28,0) R(28,29) R(29,28) R(29,30) R(30,29) R(30,31) R(31,12) R(31,30) R(n1,0) R(n1,6) R(n1,n2) R(n2,n1) R(n2,n3) R(n3,n2) R(n3,n4) R(n4,13) R(n4,15) R(n4,n3) R(n5,16) R(n5,n7) R(n6,6) R(n6,7) R(n7,15) R(n7,n5)

-------------------------------------------------------------------------------------------------------------

-> form outgroup SOL2_ALTER - B

n1(mod92)15g

[ cox1 cox2 atp8 atp6 cox3 nad3 rrnS rrnL nad1 nad6 cob nad4L nad4 nad5 nad2 ]

n1(mod92)13g

[ cox1 cox2 cox3 nad3 rrnS rrnL nad1 nad6 cob nad4L nad4 nad5 nad2 ]

n7(mod92)

[ cox1 cox2 nad1 nad3 nad2 cox3 rrnS -nad4L -nad4 -nad5 -cob -nad6 rrnL ]

score = 301:

model 92:

-------------

R(0,1) R(0,9) R(0,10) R(0,17) R(0,18) R(0,24) R(0,25) R(0,28) R(0,n1) R(1,0) R(2,17) R(3,19) R(3,20) R(4,21) R(5,23) R(6,n1) R(6,n6) R(7,n6) R(8,24) R(9,0) R(10,0) R(11,27) R(12,31) R(13,14) R(13,n4) R(13,n7) R(14,13) R(15,n7) R(16,n5) R(17,0) R(17,2) R(18,0) R(18,19) R(19,3) R(19,18) R(19,22) R(20,3) R(20,21) R(21,4) R(21,20) R(22,19) R(22,23) R(23,5) R(23,22) R(24,0) R(24,8) R(25,0) R(25,26) R(26,25) R(26,27) R(27,11) R(27,26) R(28,0) R(28,29) R(29,28) R(29,30) R(30,29) R(30,31) R(31,12) R(31,30) R(n1,0) R(n1,6) R(n1,n2) R(n2,n1) R(n2,n3) R(n3,n2) R(n3,n4) R(n4,13) R(n4,n3) R(n5,16) R(n5,n7) R(n6,6) R(n6,7) R(n7,13) R(n7,15) R(n7,n5)

-------------------------------------------------------------------------------------------------------------

-> form outgroup SOL2_ALTER - B

n1(mod93)15g

[ cox1 cox2 atp8 atp6 cox3 nad3 rrnS rrnL nad1 nad6 cob nad4L nad4 nad5 nad2 ]

n1(mod93)13g

[ cox1 cox2 cox3 nad3 rrnS rrnL nad1 nad6 cob nad4L nad4 nad5 nad2 ]

n4(mod93)

[ cox1 cox2 nad1 nad3 nad2 cox3 rrnS -nad5 -nad4 -nad4L -cob -nad6 rrnL ]

n6(mod93)

[ cox1 cox2 nad1 nad3 nad2 cox3 rrnS nad5 nad4 -nad4L -cob -nad6 rrnL ]

score = 291:

model 93:

-------------

R(0,1) R(0,9) R(0,10) R(0,17) R(0,18) R(0,24) R(0,25) R(0,28) R(0,n1) R(1,0) R(2,17) R(3,19) R(3,20) R(4,21) R(5,23) R(6,n1) R(6,n7) R(7,n7) R(8,24) R(9,0) R(10,0) R(11,27) R(12,31) R(13,14) R(13,n6) R(14,13) R(15,n4) R(16,n5) R(17,0) R(17,2) R(18,0) R(18,19) R(19,3) R(19,18) R(19,22) R(20,3) R(20,21) R(21,4) R(21,20) R(22,19) R(22,23) R(23,5) R(23,22) R(24,0) R(24,8) R(25,0) R(25,26) R(26,25) R(26,27) R(27,11) R(27,26) R(28,0) R(28,29) R(29,28) R(29,30) R(30,29) R(30,31) R(31,12) R(31,30) R(n1,0) R(n1,6) R(n1,n2) R(n2,n1) R(n2,n3) R(n3,n2) R(n3,n4) R(n4,15) R(n4,n3) R(n4,n6) R(n5,16) R(n5,n6) R(n6,13) R(n6,n4) R(n6,n5) R(n7,6) R(n7,7)

-------------------------------------------------------------------------------------------------------------

-> form outgroup SOL2_ALTER - B

n1(mod94)15g

[ cox1 cox2 atp8 atp6 cox3 nad3 rrnS rrnL nad1 nad6 cob nad4L nad4 nad5 nad2 ]

n1(mod94)13g

[ cox1 cox2 cox3 nad3 rrnS rrnL nad1 nad6 cob nad4L nad4 nad5 nad2 ]

n6(mod94)

[ cox1 cox2 nad1 nad3 nad2 cox3 rrnS -nad4L -nad4 -nad5 -cob -nad6 rrnL ]

score = 327:

model 94:

-------------

R(0,1) R(0,9) R(0,10) R(0,17) R(0,18) R(0,24) R(0,25) R(0,28) R(0,n1) R(1,0) R(2,17) R(3,19) R(3,20) R(4,21) R(5,23) R(6,n1) R(6,n7) R(7,n7) R(8,24) R(9,0) R(10,0) R(11,27) R(12,31) R(13,14) R(13,n6) R(14,13) R(15,n4) R(15,n6) R(16,n5) R(17,0) R(17,2) R(18,0) R(18,19) R(19,3) R(19,18) R(19,22) R(20,3) R(20,21) R(21,4) R(21,20) R(22,19) R(22,23) R(23,5) R(23,22) R(24,0) R(24,8) R(25,0) R(25,26) R(26,25) R(26,27) R(27,11) R(27,26) R(28,0) R(28,29) R(29,28) R(29,30) R(30,29) R(30,31) R(31,12) R(31,30) R(n1,0) R(n1,6) R(n1,n2) R(n2,n1) R(n2,n3) R(n3,n2) R(n3,n4) R(n4,15) R(n4,n3) R(n5,16) R(n5,n6) R(n6,13) R(n6,15) R(n6,n5) R(n7,6) R(n7,7)

-------------------------------------------------------------------------------------------------------------

-> form outgroup SOL1_ALTER - C

n2(mod95)15g

[ cox1 cox2 atp8 atp6 cox3 nad3 -nad5 -nad4 -nad4L nad6 cob rrnS rrnL nad1 nad2 ]

n2(mod95)13g

[ cox1 cox2 cox3 nad3 -nad5 -nad4 -nad4L nad6 cob rrnS rrnL nad1 nad2 ]

n7(mod95)

[ cox1 cox2 nad1 nad3 nad2 cox3 rrnS -nad4 -nad5 -cob -nad6 nad4L rrnL ]

score = 349:

model 95:

-------------

R(0,1) R(0,9) R(0,10) R(0,17) R(0,18) R(0,24) R(0,25) R(0,28) R(0,n1) R(1,0) R(2,17) R(3,19) R(3,20) R(4,21) R(5,23) R(6,n2) R(7,n2) R(8,24) R(9,0) R(10,0) R(11,27) R(12,31) R(13,14) R(13,15) R(13,n7) R(14,13) R(15,13) R(16,n5) R(17,0) R(17,2) R(18,0) R(18,19) R(19,3) R(19,18) R(19,22) R(20,3) R(20,21) R(21,4) R(21,20) R(22,19) R(22,23) R(23,5) R(23,22) R(24,0) R(24,8) R(25,0) R(25,26) R(26,25) R(26,27) R(27,11) R(27,26) R(28,0) R(28,29) R(29,28) R(29,30) R(30,29) R(30,31) R(31,12) R(31,30) R(n1,0) R(n1,n2) R(n2,6) R(n2,7) R(n2,n1) R(n2,n3) R(n3,n2) R(n3,n4) R(n4,n3) R(n4,n6) R(n5,16) R(n5,n7) R(n6,n4) R(n6,n7) R(n7,13) R(n7,n5) R(n7,n6)

-------------------------------------------------------------------------------------------------------------

-> form outgroup SOL2_ALTER - C

n2(mod96)15g

[ cox1 cox2 atp8 atp6 cox3 nad3 -nad5 -nad4 -nad4L nad6 cob rrnS rrnL nad1 nad2 ]

n2(mod96)13g

[ cox1 cox2 cox3 nad3 -nad5 -nad4 -nad4L nad6 cob rrnS rrnL nad1 nad2 ]

n7(mod96)

[ cox1 cox2 nad1 nad3 nad2 cox3 rrnS -nad4 -nad5 -cob -nad6 nad4L rrnL ]

score = 393:

model 96:

-------------

R(0,1) R(0,9) R(0,10) R(0,17) R(0,18) R(0,24) R(0,25) R(0,28) R(0,n1) R(1,0) R(2,17) R(3,19) R(3,20) R(4,21) R(5,23) R(6,n1) R(6,n2) R(7,n2) R(8,24) R(9,0) R(10,0) R(11,27) R(12,31) R(13,14) R(13,15) R(13,n7) R(14,13) R(15,13) R(16,n5) R(17,0) R(17,2) R(18,0) R(18,19) R(19,3) R(19,18) R(19,22) R(20,3) R(20,21) R(21,4) R(21,20) R(22,19) R(22,23) R(23,5) R(23,22) R(24,0) R(24,8) R(25,0) R(25,26) R(26,25) R(26,27) R(27,11) R(27,26) R(28,0) R(28,29) R(29,28) R(29,30) R(30,29) R(30,31) R(31,12) R(31,30) R(n1,0) R(n1,6) R(n2,6) R(n2,7) R(n2,n3) R(n3,n2) R(n3,n4) R(n4,n3) R(n4,n6) R(n5,16) R(n5,n7) R(n6,n4) R(n6,n7) R(n7,13) R(n7,n5) R(n7,n6)

-------------------------------------------------------------------------------------------------------------

-> no other solutions

chaetognaths_taxC_278sol

================================================================================

================================================================================

AXIOMS

================================================================================

================================================================================

{ the solutions of problem PHYLO are the smallest graphs T (defined on the smallest domain possible but containing at least all the OTUs) which verify properties P1 to P6:

P1- T is simple (the relation R(x, y) which defines graph T is not reflexive)

P2- T is non-oriented (the relation R(x, y) which defines graph T is symmetrical)

P3- T is connected and acyclic (T is a tree)

P4- T respects the minimal distance matrix, i.e.:

for all couple of OTUs x and y, the length of the path x->y in T is always superior or equals to the minimal distance calculated between x and y (encoded in the minimal distance matrix)

P5- T respects other eventual hypothesis (Primary Phylogenetic Hypothesis = PPH)

used to impose the existence of given monophyletic groups

P6- it is possible to calculate all the values for each HTU in the graph T }

{ OTUs: }

katharina_tunicata = 0;

nautilus_macromphallus = 1;

loligo_bleekeri = 2;

platynereis_dumerilii = 3;

urechis_caupo = 4;

sipunculus_nudus = 5;

limulus_polyphemus = 6; { = outgroup1 }

homo_sapiens = 7; { = outgroup2 }

loxocorone_allax = 8;

terebratulina_retusa = 9;

phoronis_architecta = 10;

bugula_neritina = 11;

terebratalia_transversa = 12;

sagitta_enflata = 13;

sagitta_nagae = 14;

paraspadella_gotoi = 15;

spadella_cephaloptera = 16;

priapulus_caudatus = 17; { = outgroup3 }

{ AUXILLIARY CONSTANTS used to fix a part of the solution: }

G1=18; G2=19; G3=20; G4=21;

G5=22; G6=23; G7=24; G8=25; { used to fix the Eutrochozoa group }

G9=26; G10=27; G11=28; G12=29;

G13=30; G14=31; G15=32; { used to fix the Lophophorata group }

{ THE EUTROCHOZOA GROUP IS FIXED: }

{ OTUs (0,1,2): }

R(katharina_tunicata,nautilus_macromphallus);

R(katharina_tunicata,G1);

R(G1,loligo_bleekeri);

Q x ( x<>katharina_tunicata

=>

-R(nautilus_macromphallus,x)

);

Q x ( ( x<>katharina_tunicata et

x<>loligo_bleekeri

)

=>

-R(G1,x)

);

Q x ( x<>G1

=>

-R(loligo_bleekeri,x)

);

{ OTUs (3,4,5,8): }

{ as in the best of the 3 possible forms }

R(katharina_tunicata,G2);

R(G2,G3);

R(G3,platynereis_dumerilii);

R(platynereis_dumerilii,G4);

R(G4,G5);

R(G5,urechis_caupo);

R(G3,G6);

R(G6,G7);

R(G7,sipunculus_nudus);

Q x ( ( x<>katharina_tunicata et

x<>G3

)

=>

-R(G2,x)

);

Q x ( ( x<>G2 et

x<>platynereis_dumerilii et

x<>G6

)

=>

-R(G3,x)

);

Q x ( ( x<>platynereis_dumerilii et

x<>G5

)

=>

-R(G4,x)

);

Q x ( ( x<>G3 et

x<>G4

)

=>

-R(platynereis_dumerilii,x)

);

Q x ( ( x<>G4 et

x<>urechis_caupo

)

=>

-R(G5,x)

);

Q x ( x<>G5

=>

-R(urechis_caupo,x)

);

Q x ( ( x<>G3 et

x<>G7

)

=>

-R(G6,x)

);

Q x ( ( x<>G6 et

x<>sipunculus_nudus

)

=>

-R(G7,x)

);

Q x ( x<>G7

=>

-R(sipunculus_nudus,x)

);

R(katharina_tunicata,G8);

R(G8,loxocorone_allax);

Q x ( ( x<>katharina_tunicata et

x<>loxocorone_allax

)

=>

-R(G8,x)

);

Q x ( x<>G8

=>

-R(loxocorone_allax, x)

);

{ THE LOPHOPHORATA GROUP IS FIXED: }

{ as in the best of the 9 possible forms }

R(katharina_tunicata,terebratulina_retusa);

R(katharina_tunicata,phoronis_architecta);

Q x ( x<>katharina_tunicata

=>

-R(terebratulina_retusa,x)

);

Q x ( x<>katharina_tunicata

=>

-R(phoronis_architecta,x)

);

R(katharina_tunicata,G9);

R(G9,G10);

R(G10,G11);

R(G11,bugula_neritina);

Q x ( ( x<>katharina_tunicata et

x<>G10

)

=>

-R(G9,x)

);

Q x ( ( x<>G9 et

x<>G11

)

=>

-R(G10,x)

);

Q x ( ( x<>G10 et

x<>bugula_neritina

)

=>

-R(G11,x)

);

Q x ( x<>G11

=>

-R(bugula_neritina,x)

);

R(katharina_tunicata,G12);

R(G12,G13);

R(G13,G14);

R(G14,G15);

R(G15,terebratalia_transversa);

Q x ( ( x<>katharina_tunicata et

x<>G13

)

=>

-R(G12,x)

);

Q x ( ( x<>G12 et

x<>G14

)

=>

-R(G13,x)

);

Q x ( ( x<>G13 et

x<>G15

)

=>

-R(G14,x)

);

Q x ( ( x<>G14 et

x<>terebratalia_transversa

)

=>

-R(G15,x)

);

Q x ( x<>G15

=>

-R(terebratalia_transversa,x)

);

{ PROPERTY P1: R(x, y) is not reflexive}

Q x (-R(x, x));

{ PROPERTY P2: R(x, y) is symmetrical}

Q x y (R(x, y) => R(y, x));

{ PROPERTY P3: graph T is connected and acyclic (T is a tree) }

{

This property is verified by a constraint programmed in the model generator, instead of a "heavy" logical formula:

1- it will refuse the partial interpretations in which a connected component of the graph (in construction) is cyclic, i.e. such as: number of edges >= number of vertices

2- it will refuse the complete interpretations in which the constructed graph has more than one connected component

}

{ PROPERTY P4: graph T respects minimal distance matrix }

{

This property is verified by a constraint programmed in the model generator:

it will refuse the partial interpretations in which the graph (in construction) do not respect the minimal distance matrix, i.e. such as:

let x, y a couple of OTUs,

let d= minimal distance calculated between x and y (encoded in the minimal distance matrix), there is a a path of length k between x and y, with: k < d

The minimal distance matrix is encoded directly in the data structure of the model generator:

/* minimal distance matrix chaetognaths taxC: */

DIST[0][0]=0;

DIST[1][0]=1; DIST[1][1]=0;

DIST[2][0]=2; DIST[2][1]=3; DIST[2][2]=0;

DIST[3][0]=3; DIST[3][1]=4; DIST[3][2]=4; DIST[3][3]=0;

DIST[4][0]=5; DIST[4][1]=5; DIST[4][2]=5; DIST[4][3]=3; DIST[4][4]=0;

DIST[5][0]=4; DIST[5][1]=4; DIST[5][2]=5; DIST[5][3]=3; DIST[5][4]=4; DIST[5][5]=0;

DIST[6][0]=2; DIST[6][1]=3; DIST[6][2]=2; DIST[6][3]=4; DIST[6][4]=5; DIST[6][5]=4; DIST[6][6]=0;

DIST[7][0]=3; DIST[7][1]=4; DIST[7][2]=3; DIST[7][3]=4; DIST[7][4]=5; DIST[7][5]=4; DIST[7][6]=2; DIST[7][7]=0;

DIST[8][0]=2; DIST[8][1]=2; DIST[8][2]=4; DIST[8][3]=5; DIST[8][4]=5; DIST[8][5]=5; DIST[8][6]=4; DIST[8][7]=4; DIST[8][8]=0;

DIST[9][0]=1; DIST[9][1]=2; DIST[9][2]=2; DIST[9][3]=3; DIST[9][4]=4; DIST[9][5]=5; DIST[9][6]=2; DIST[9][7]=3; DIST[9][8]=3; DIST[9][9]=0;

DIST[10][0]=1; DIST[10][1]=2; DIST[10][2]=3; DIST[10][3]=4; DIST[10][4]=5; DIST[10][5]=4; DIST[10][6]=3; DIST[10][7]=4; DIST[10][8]=3; DIST[10][9]=2; DIST[10][10]=0;

DIST[11][0]=4; DIST[11][1]=5; DIST[11][2]=5; DIST[11][3]=6; DIST[11][4]=6; DIST[11][5]=6; DIST[11][6]=5; DIST[11][7]=5; DIST[11][8]=5; DIST[11][9]=4; DIST[11][10]=4; DIST[11][11]=0;

DIST[12][0]=5; DIST[12][1]=6; DIST[12][2]=6; DIST[12][3]=5; DIST[12][4]=7; DIST[12][5]=6; DIST[12][6]=6; DIST[12][7]=7; DIST[12][8]=7; DIST[12][9]=5; DIST[12][10]=5; DIST[12][11]=7; DIST[12][12]=0;

DIST[13][0]=4; DIST[13][1]=4; DIST[13][2]=5; DIST[13][3]=3; DIST[13][4]=5; DIST[13][5]=5; DIST[13][6]=5; DIST[13][7]=5; DIST[13][8]=5; DIST[13][9]=4; DIST[13][10]=4; DIST[13][11]=4; DIST[13][12]=6; DIST[13][13]=0;

DIST[14][0]=5; DIST[14][1]=4; DIST[14][2]=6; DIST[14][3]=4; DIST[14][4]=6; DIST[14][5]=6; DIST[14][6]=5; DIST[14][7]=5; DIST[14][8]=5; DIST[14][9]=5; DIST[14][10]=5; DIST[14][11]=4; DIST[14][12]=6; DIST[14][13]=1; DIST[14][14]=0;

DIST[15][0]=4; DIST[15][1]=4; DIST[15][2]=5; DIST[15][3]=4; DIST[15][4]=5; DIST[15][5]=5; DIST[15][6]=5; DIST[15][7]=5; DIST[15][8]=5; DIST[15][9]=4; DIST[15][10]=4; DIST[15][11]=4; DIST[15][12]=5; DIST[15][13]=1; DIST[15][14]=2; DIST[15][15]=0;

DIST[16][0]=6; DIST[16][1]=5; DIST[16][2]=6; DIST[16][3]=4; DIST[16][4]=5; DIST[16][5]=6; DIST[16][6]=6; DIST[16][7]=6; DIST[16][8]=5; DIST[16][9]=5; DIST[16][10]=5; DIST[16][11]=5; DIST[16][12]=6; DIST[16][13]=3; DIST[16][14]=4; DIST[16][15]=3; DIST[16][16]=0;

DIST[17][0]=2; DIST[17][1]=3; DIST[17][2]=2; DIST[17][3]=4; DIST[17][4]=4; DIST[17][5]=4; DIST[17][6]=1; DIST[17][7]=2; DIST[17][8]=4; DIST[17][9]=2; DIST[17][10]=3; DIST[17][11]=5; DIST[17][12]=6; DIST[17][13]=5; DIST[17][14]=5; DIST[17][15]=5; DIST[17][16]=6; DIST[17][17]=0;

}

{ PROPERTY P5: graph T respects eventual Primary Phylogenetic Hypotheses }

{

This property is verified by constraints programmed in the model generator:

- monophyly of Lophotrochozoa = (0,1,2,3,4,5,8,9,10,11,12)

- monophyly of Eutrochozoa = (0,1,2,3,4,5,8)

- monophyly of Mollusca = (0,1,2)

- monophyly of Cephalopoda = (1,2)

- monophyly of Annelida = (3,4)

- monophyly of Lophophorata = (9,10,11,12)

- monophyly of Chaetognatha = (13,14,15,16)

- monophyly of Ecdysozoa = (6,17)

}

{------------------------------------------------------------------------------------------------------------------------}

{ PROPERTY P6: it is possible to calculate all the values for each HTU in the graph T }

{

First we calculate with the model generator the set of tree solutions which verify properties P1 to P5. Property P6 is verified *a posteriori* for each tree solution, with a *feedback* mechanism:

Studying each tree solution for calculating the values of HTUs, we eventually discover "impossible sub-trees": they appear in tree solutions which verify P1 to P5, but they do not verify P6.

For each impossible subtree A, an additional constraint is programmed into the model generator to forbid the solutions containing A. Tree solutions are recalculated and verified, allowing the discovery of new impossible subtrees and the programming of new constraints to recalculate the solutions (feedback mechanism). Finally, the complete set of optimal solutions is determined after iteration of this process and elimination of all the solutions that do not verify P6.

}

================================================================================

================================================================================

SOLUTIONS

================================================================================

================================================================================

OTUs:

katharina_tunicata = 0;

nautilus_macromphallus = 1;

loligo_bleekeri = 2;

platynereis_dumerilii = 3;

urechis_caupo = 4;

sipunculus_nudus = 5;

limulus_polyphemus = 6; { = outgroup1 }

homo_sapiens = 7; { = outgroup2 }

loxocorone_allax = 8;

terebratulina_retusa = 9;

phoronis_architecta = 10;

bugula_neritina=11;

terebratalia_transversa=12;

sagitta_enflata=13;

sagitta_nagae=14;

paraspadella_gotoi=15;

spadella_cephaloptera=16;

priapulus_caudatus = 17; { = outgroup3 }

AUXILLIARY CONSTANTS used to fix a part of the solution:

G1=18; G2=19; G3=20; G4=21;

G5=22; G6=23; G7=24; G8=25; { used to fix the Eutrochozoa group }

G9=26; G10=27; G11=28; G12=29;

G13=30; G14=31; G15=32; { used to fix the Lophophorata group }

HTUs:

n1, n2, n3, n4, n5, n6, n7

NOTE:

In every solution, we have to *insert* in the chaetognaths lineage (OTUs 13,14,15,16) one mutation "loss of the 2 successive genes atp8-atp6" proper to the chaetognaths lineage (common for all chaetognaths): it is the most parsimonious possibility.

In every solution of this file, this "loss" mutation can be inserted at the *beginning* of the chaetognaths lineage, and the possible values for the corresponding ancestral state are given first with 15 genes (before the loss) and then with 13 genes (after the loss of genes atp8-atp6).

But it is possible to insert this loss mutation *at* *any position* proper to the chaetognaths lineage, and easily reconstruct the possible values for ancestral states (with 15 genes) at the beginning of the chaetognaths lineage, before the loss mutation.

D = [0,39]: 278 solutions OK (which verify property P6) (266 impossible sub-trees)

minimal score (best) = 264

maximal score = 448

-------------------------------------------------------------------------------------------------------------

-> form outgroup BILA15 - A

n3(mod1)

[ cox1 cox2 atp8 atp6 cox3 nad3 -nad5 -nad4 -nad4L nad6 cob rrnS rrnL nad1 nad2 ]

score = 301:

model 1:

-------------

R(0,1) R(0,9) R(0,10) R(0,18) R(0,19) R(0,25) R(0,26) R(0,29) R(0,n1) R(0,n2) R(1,0) R(2,18) R(3,20) R(3,21) R(4,22) R(5,24) R(6,n3) R(7,n3) R(8,25) R(9,0) R(10,0) R(11,28) R(12,32) R(13,14) R(13,15) R(13,n4) R(13,n5) R(14,13) R(15,13) R(16,n6) R(17,n3) R(18,0) R(18,2) R(19,0) R(19,20) R(20,3) R(20,19) R(20,23) R(21,3) R(21,22) R(22,4) R(22,21) R(23,20) R(23,24) R(24,5) R(24,23) R(25,0) R(25,8) R(26,0) R(26,27) R(27,26) R(27,28) R(28,11) R(28,27) R(29,0) R(29,30) R(30,29) R(30,31) R(31,30) R(31,32) R(32,12) R(32,31) R(n1,0) R(n1,n7) R(n2,0) R(n2,n3) R(n3,6) R(n3,7) R(n3,17) R(n3,n2) R(n4,13) R(n4,n6) R(n5,13) R(n5,n7) R(n6,16) R(n6,n4) R(n7,n1) R(n7,n5)

-------------------------------------------------------------------------------------------------------------

-> form outgroup BILA15 - A

n3(mod2)

[ cox1 cox2 atp8 atp6 cox3 nad3 -nad5 -nad4 -nad4L nad6 cob rrnS rrnL nad1 nad2 ]

n4(mod2)

[ cox1 cox2 nad1 nad3 nad2 cox3 rrnS -nad4L -nad4 -nad5 -cob -nad6 rrnL ]

score = 316:

model 2:

-------------

R(0,1) R(0,9) R(0,10) R(0,18) R(0,19) R(0,25) R(0,26) R(0,29) R(0,n1) R(0,n2) R(1,0) R(2,18) R(3,20) R(3,21) R(4,22) R(5,24) R(6,n3) R(7,n3) R(8,25) R(9,0) R(10,0) R(11,28) R(12,32) R(13,14) R(13,n4) R(13,n5) R(14,13) R(15,n4) R(16,n6) R(17,n3) R(18,0) R(18,2) R(19,0) R(19,20) R(20,3) R(20,19) R(20,23) R(21,3) R(21,22) R(22,4) R(22,21) R(23,20) R(23,24) R(24,5) R(24,23) R(25,0) R(25,8) R(26,0) R(26,27) R(27,26) R(27,28) R(28,11) R(28,27) R(29,0) R(29,30) R(30,29) R(30,31) R(31,30) R(31,32) R(32,12) R(32,31) R(n1,0) R(n1,n7) R(n2,0) R(n2,n3) R(n3,6) R(n3,7) R(n3,17) R(n3,n2) R(n4,13) R(n4,15) R(n4,n6) R(n5,13) R(n5,n7) R(n6,16) R(n6,n4) R(n7,n1) R(n7,n5)

-------------------------------------------------------------------------------------------------------------

-> form outgroup BILA15 - A

n3(mod3)

[ cox1 cox2 atp8 atp6 cox3 nad3 -nad5 -nad4 -nad4L nad6 cob rrnS rrnL nad1 nad2 ]

n5(mod3)

[ cox1 cox2 nad1 nad3 nad2 cox3 rrnS -nad5 -nad4 -nad4L -cob -nad6 rrnL ]

score = 290:

model 3:

-------------

R(0,1) R(0,9) R(0,10) R(0,18) R(0,19) R(0,25) R(0,26) R(0,29) R(0,n1) R(0,n2) R(1,0) R(2,18) R(3,20) R(3,21) R(4,22) R(5,24) R(6,n3) R(7,n3) R(8,25) R(9,0) R(10,0) R(11,28) R(12,32) R(13,14) R(13,n4) R(13,n5) R(14,13) R(15,n5) R(16,n6) R(17,n3) R(18,0) R(18,2) R(19,0) R(19,20) R(20,3) R(20,19) R(20,23) R(21,3) R(21,22) R(22,4) R(22,21) R(23,20) R(23,24) R(24,5) R(24,23) R(25,0) R(25,8) R(26,0) R(26,27) R(27,26) R(27,28) R(28,11) R(28,27) R(29,0) R(29,30) R(30,29) R(30,31) R(31,30) R(31,32) R(32,12) R(32,31) R(n1,0) R(n1,n7) R(n2,0) R(n2,n3) R(n3,6) R(n3,7) R(n3,17) R(n3,n2) R(n4,13) R(n4,n6) R(n5,13) R(n5,15) R(n5,n7) R(n6,16) R(n6,n4) R(n7,n1) R(n7,n5)

-------------------------------------------------------------------------------------------------------------

-> Ur-ecdysozoa = limulus_polyphemus (solution already obtained in "chaetognaths_taxA")

score = 301:

model 4:

-------------

R(0,1) R(0,9) R(0,10) R(0,18) R(0,19) R(0,25) R(0,26) R(0,29) R(0,n1) R(0,n2) R(1,0) R(2,18) R(3,20) R(3,21) R(4,22) R(5,24) R(6,17) R(6,n2) R(6,n3) R(7,n3) R(8,25) R(9,0) R(10,0) R(11,28) R(12,32) R(13,14) R(13,15) R(13,n4) R(13,n5) R(14,13) R(15,13) R(16,n6) R(17,6) R(18,0) R(18,2) R(19,0) R(19,20) R(20,3) R(20,19) R(20,23) R(21,3) R(21,22) R(22,4) R(22,21) R(23,20) R(23,24) R(24,5) R(24,23) R(25,0) R(25,8) R(26,0) R(26,27) R(27,26) R(27,28) R(28,11) R(28,27) R(29,0) R(29,30) R(30,29) R(30,31) R(31,30) R(31,32) R(32,12) R(32,31) R(n1,0) R(n1,n7) R(n2,0) R(n2,6) R(n3,6) R(n3,7) R(n4,13) R(n4,n6) R(n5,13) R(n5,n7) R(n6,16) R(n6,n4) R(n7,n1) R(n7,n5)

-------------------------------------------------------------------------------------------------------------

-> Ur-ecdysozoa = limulus_polyphemus (solution already obtained in "chaetognaths_taxA")

score = 316:

model 5:

-------------

R(0,1) R(0,9) R(0,10) R(0,18) R(0,19) R(0,25) R(0,26) R(0,29) R(0,n1) R(0,n2) R(1,0) R(2,18) R(3,20) R(3,21) R(4,22) R(5,24) R(6,17) R(6,n3) R(7,n3) R(8,25) R(9,0) R(10,0) R(11,28) R(12,32) R(13,14) R(13,15) R(13,n4) R(13,n5) R(14,13) R(15,13) R(16,n6) R(17,6) R(18,0) R(18,2) R(19,0) R(19,20) R(20,3) R(20,19) R(20,23) R(21,3) R(21,22) R(22,4) R(22,21) R(23,20) R(23,24) R(24,5) R(24,23) R(25,0) R(25,8) R(26,0) R(26,27) R(27,26) R(27,28) R(28,11) R(28,27) R(29,0) R(29,30) R(30,29) R(30,31) R(31,30) R(31,32) R(32,12) R(32,31) R(n1,0) R(n1,n7) R(n2,0) R(n2,n3) R(n3,6) R(n3,7) R(n3,n2) R(n4,13) R(n4,n6) R(n5,13) R(n5,n7) R(n6,16) R(n6,n4) R(n7,n1) R(n7,n5)

-------------------------------------------------------------------------------------------------------------

-> Ur-ecdysozoa = limulus_polyphemus (solution already obtained in "chaetognaths_taxA")

score = 316:

model 6:

-------------

R(0,1) R(0,9) R(0,10) R(0,18) R(0,19) R(0,25) R(0,26) R(0,29) R(0,n1) R(0,n2) R(1,0) R(2,18) R(3,20) R(3,21) R(4,22) R(5,24) R(6,17) R(6,n2) R(6,n3) R(7,n3) R(8,25) R(9,0) R(10,0) R(11,28) R(12,32) R(13,14) R(13,n4) R(13,n5) R(14,13) R(15,n4) R(16,n6) R(17,6) R(18,0) R(18,2) R(19,0) R(19,20) R(20,3) R(20,19) R(20,23) R(21,3) R(21,22) R(22,4) R(22,21) R(23,20) R(23,24) R(24,5) R(24,23) R(25,0) R(25,8) R(26,0) R(26,27) R(27,26) R(27,28) R(28,11) R(28,27) R(29,0) R(29,30) R(30,29) R(30,31) R(31,30) R(31,32) R(32,12) R(32,31) R(n1,0) R(n1,n7) R(n2,0) R(n2,6) R(n3,6) R(n3,7) R(n4,13) R(n4,15) R(n4,n6) R(n5,13) R(n5,n7) R(n6,16) R(n6,n4) R(n7,n1) R(n7,n5)

-------------------------------------------------------------------------------------------------------------

-> Ur-ecdysozoa = limulus_polyphemus (solution already obtained in "chaetognaths_taxA")

score = 331:

model 7:

-------------

R(0,1) R(0,9) R(0,10) R(0,18) R(0,19) R(0,25) R(0,26) R(0,29) R(0,n1) R(0,n2) R(1,0) R(2,18) R(3,20) R(3,21) R(4,22) R(5,24) R(6,17) R(6,n3) R(7,n3) R(8,25) R(9,0) R(10,0) R(11,28) R(12,32) R(13,14) R(13,n4) R(13,n5) R(14,13) R(15,n4) R(16,n6) R(17,6) R(18,0) R(18,2) R(19,0) R(19,20) R(20,3) R(20,19) R(20,23) R(21,3) R(21,22) R(22,4) R(22,21) R(23,20) R(23,24) R(24,5) R(24,23) R(25,0) R(25,8) R(26,0) R(26,27) R(27,26) R(27,28) R(28,11) R(28,27) R(29,0) R(29,30) R(30,29) R(30,31) R(31,30) R(31,32) R(32,12) R(32,31) R(n1,0) R(n1,n7) R(n2,0) R(n2,n3) R(n3,6) R(n3,7) R(n3,n2) R(n4,13) R(n4,15) R(n4,n6) R(n5,13) R(n5,n7) R(n6,16) R(n6,n4) R(n7,n1) R(n7,n5)

-------------------------------------------------------------------------------------------------------------

-> Ur-ecdysozoa = limulus_polyphemus (solution already obtained in "chaetognaths_taxA")

score = 290:

model 8:

-------------

R(0,1) R(0,9) R(0,10) R(0,18) R(0,19) R(0,25) R(0,26) R(0,29) R(0,n1) R(0,n2) R(1,0) R(2,18) R(3,20) R(3,21) R(4,22) R(5,24) R(6,17) R(6,n2) R(6,n3) R(7,n3) R(8,25) R(9,0) R(10,0) R(11,28) R(12,32) R(13,14) R(13,n4) R(13,n5) R(14,13) R(15,n5) R(16,n6) R(17,6) R(18,0) R(18,2) R(19,0) R(19,20) R(20,3) R(20,19) R(20,23) R(21,3) R(21,22) R(22,4) R(22,21) R(23,20) R(23,24) R(24,5) R(24,23) R(25,0) R(25,8) R(26,0) R(26,27) R(27,26) R(27,28) R(28,11) R(28,27) R(29,0) R(29,30) R(30,29) R(30,31) R(31,30) R(31,32) R(32,12) R(32,31) R(n1,0) R(n1,n7) R(n2,0) R(n2,6) R(n3,6) R(n3,7) R(n4,13) R(n4,n6) R(n5,13) R(n5,15) R(n5,n7) R(n6,16) R(n6,n4) R(n7,n1) R(n7,n5)

-------------------------------------------------------------------------------------------------------------

-> Ur-ecdysozoa = limulus_polyphemus (solution already obtained in "chaetognaths_taxA")

score = 305:

model 9:

-------------

R(0,1) R(0,9) R(0,10) R(0,18) R(0,19) R(0,25) R(0,26) R(0,29) R(0,n1) R(0,n2) R(1,0) R(2,18) R(3,20) R(3,21) R(4,22) R(5,24) R(6,17) R(6,n3) R(7,n3) R(8,25) R(9,0) R(10,0) R(11,28) R(12,32) R(13,14) R(13,n4) R(13,n5) R(14,13) R(15,n5) R(16,n6) R(17,6) R(18,0) R(18,2) R(19,0) R(19,20) R(20,3) R(20,19) R(20,23) R(21,3) R(21,22) R(22,4) R(22,21) R(23,20) R(23,24) R(24,5) R(24,23) R(25,0) R(25,8) R(26,0) R(26,27) R(27,26) R(27,28) R(28,11) R(28,27) R(29,0) R(29,30) R(30,29) R(30,31) R(31,30) R(31,32) R(32,12) R(32,31) R(n1,0) R(n1,n7) R(n2,0) R(n2,n3) R(n3,6) R(n3,7) R(n3,n2) R(n4,13) R(n4,n6) R(n5,13) R(n5,15) R(n5,n7) R(n6,16) R(n6,n4) R(n7,n1) R(n7,n5)

-------------------------------------------------------------------------------------------------------------

-> Ur-ecdysozoa = limulus_polyphemus (solution already obtained in "chaetognaths_taxA")

score = 329:

model 10:

-------------

R(0,1) R(0,9) R(0,10) R(0,18) R(0,19) R(0,25) R(0,26) R(0,29) R(0,n1) R(0,n2) R(1,0) R(2,18) R(3,20) R(3,21) R(4,22) R(5,24) R(6,17) R(6,n2) R(6,n3) R(7,n3) R(8,25) R(9,0) R(10,0) R(11,28) R(12,32) R(13,14) R(13,15) R(13,n4) R(14,13) R(15,13) R(15,n5) R(16,n6) R(17,6) R(18,0) R(18,2) R(19,0) R(19,20) R(20,3) R(20,19) R(20,23) R(21,3) R(21,22) R(22,4) R(22,21) R(23,20) R(23,24) R(24,5) R(24,23) R(25,0) R(25,8) R(26,0) R(26,27) R(27,26) R(27,28) R(28,11) R(28,27) R(29,0) R(29,30) R(30,29) R(30,31) R(31,30) R(31,32) R(32,12) R(32,31) R(n1,0) R(n1,n7) R(n2,0) R(n2,6) R(n3,6) R(n3,7) R(n4,13) R(n4,n6) R(n5,15) R(n5,n7) R(n6,16) R(n6,n4) R(n7,n1) R(n7,n5)

-------------------------------------------------------------------------------------------------------------

-> Ur-ecdysozoa = limulus_polyphemus (solution already obtained in "chaetognaths_taxA")

score = 344:

model 11:

-------------

R(0,1) R(0,9) R(0,10) R(0,18) R(0,19) R(0,25) R(0,26) R(0,29) R(0,n1) R(0,n2) R(1,0) R(2,18) R(3,20) R(3,21) R(4,22) R(5,24) R(6,17) R(6,n3) R(7,n3) R(8,25) R(9,0) R(10,0) R(11,28) R(12,32) R(13,14) R(13,15) R(13,n4) R(14,13) R(15,13) R(15,n5) R(16,n6) R(17,6) R(18,0) R(18,2) R(19,0) R(19,20) R(20,3) R(20,19) R(20,23) R(21,3) R(21,22) R(22,4) R(22,21) R(23,20) R(23,24) R(24,5) R(24,23) R(25,0) R(25,8) R(26,0) R(26,27) R(27,26) R(27,28) R(28,11) R(28,27) R(29,0) R(29,30) R(30,29) R(30,31) R(31,30) R(31,32) R(32,12) R(32,31) R(n1,0) R(n1,n7) R(n2,0) R(n2,n3) R(n3,6) R(n3,7) R(n3,n2) R(n4,13) R(n4,n6) R(n5,15) R(n5,n7) R(n6,16) R(n6,n4) R(n7,n1) R(n7,n5)

-------------------------------------------------------------------------------------------------------------

-> Ur-ecdysozoa = limulus_polyphemus (solution already obtained in "chaetognaths_taxA")

score = 316:

model 12:

-------------

R(0,1) R(0,9) R(0,10) R(0,18) R(0,19) R(0,25) R(0,26) R(0,29) R(0,n1) R(0,n2) R(1,0) R(2,18) R(3,20) R(3,21) R(4,22) R(5,24) R(6,17) R(6,n1) R(6,n3) R(7,n3) R(8,25) R(9,0) R(10,0) R(11,28) R(12,32) R(13,14) R(13,15) R(13,n4) R(14,13) R(15,13) R(15,n5) R(16,n7) R(17,6) R(18,0) R(18,2) R(19,0) R(19,20) R(20,3) R(20,19) R(20,23) R(21,3) R(21,22) R(22,4) R(22,21) R(23,20) R(23,24) R(24,5) R(24,23) R(25,0) R(25,8) R(26,0) R(26,27) R(27,26) R(27,28) R(28,11) R(28,27) R(29,0) R(29,30) R(30,29) R(30,31) R(31,30) R(31,32) R(32,12) R(32,31) R(n1,0) R(n1,6) R(n2,0) R(n2,n6) R(n3,6) R(n3,7) R(n4,13) R(n4,n6) R(n5,15) R(n5,n7) R(n6,n2) R(n6,n4) R(n7,16) R(n7,n5)

-------------------------------------------------------------------------------------------------------------

-> Ur-ecdysozoa = limulus_polyphemus (solution already obtained in "chaetognaths_taxA")

score = 331:

model 13:

-------------

R(0,1) R(0,9) R(0,10) R(0,18) R(0,19) R(0,25) R(0,26) R(0,29) R(0,n1) R(0,n2) R(1,0) R(2,18) R(3,20) R(3,21) R(4,22) R(5,24) R(6,17) R(6,n3) R(7,n3) R(8,25) R(9,0) R(10,0) R(11,28) R(12,32) R(13,14) R(13,15) R(13,n4) R(14,13) R(15,13) R(15,n5) R(16,n7) R(17,6) R(18,0) R(18,2) R(19,0) R(19,20) R(20,3) R(20,19) R(20,23) R(21,3) R(21,22) R(22,4) R(22,21) R(23,20) R(23,24) R(24,5) R(24,23) R(25,0) R(25,8) R(26,0) R(26,27) R(27,26) R(27,28) R(28,11) R(28,27) R(29,0) R(29,30) R(30,29) R(30,31) R(31,30) R(31,32) R(32,12) R(32,31) R(n1,0) R(n1,n3) R(n2,0) R(n2,n6) R(n3,6) R(n3,7) R(n3,n1) R(n4,13) R(n4,n6) R(n5,15) R(n5,n7) R(n6,n2) R(n6,n4) R(n7,16) R(n7,n5)

-------------------------------------------------------------------------------------------------------------

-> Ur-ecdysozoa = limulus_polyphemus (solution already obtained in "chaetognaths_taxA")

score = 329:

model 14:

-------------

R(0,1) R(0,9) R(0,10) R(0,18) R(0,19) R(0,25) R(0,26) R(0,29) R(0,n1) R(0,n2) R(1,0) R(2,18) R(3,20) R(3,21) R(4,22) R(5,24) R(6,17) R(6,n2) R(6,n3) R(7,n3) R(8,25) R(9,0) R(10,0) R(11,28) R(12,32) R(13,14) R(13,15) R(13,n4) R(14,13) R(15,13) R(16,n5) R(17,6) R(18,0) R(18,2) R(19,0) R(19,20) R(20,3) R(20,19) R(20,23) R(21,3) R(21,22) R(22,4) R(22,21) R(23,20) R(23,24) R(24,5) R(24,23) R(25,0) R(25,8) R(26,0) R(26,27) R(27,26) R(27,28) R(28,11) R(28,27) R(29,0) R(29,30) R(30,29) R(30,31) R(31,30) R(31,32) R(32,12) R(32,31) R(n1,0) R(n1,n7) R(n2,0) R(n2,6) R(n3,6) R(n3,7) R(n4,13) R(n4,n5) R(n4,n6) R(n5,16) R(n5,n4) R(n6,n4) R(n6,n7) R(n7,n1) R(n7,n6)

-------------------------------------------------------------------------------------------------------------

-> Ur-ecdysozoa = limulus_polyphemus (solution already obtained in "chaetognaths_taxA")

score = 344:

model 15:

-------------

R(0,1) R(0,9) R(0,10) R(0,18) R(0,19) R(0,25) R(0,26) R(0,29) R(0,n1) R(0,n2) R(1,0) R(2,18) R(3,20) R(3,21) R(4,22) R(5,24) R(6,17) R(6,n3) R(7,n3) R(8,25) R(9,0) R(10,0) R(11,28) R(12,32) R(13,14) R(13,15) R(13,n4) R(14,13) R(15,13) R(16,n5) R(17,6) R(18,0) R(18,2) R(19,0) R(19,20) R(20,3) R(20,19) R(20,23) R(21,3) R(21,22) R(22,4) R(22,21) R(23,20) R(23,24) R(24,5) R(24,23) R(25,0) R(25,8) R(26,0) R(26,27) R(27,26) R(27,28) R(28,11) R(28,27) R(29,0) R(29,30) R(30,29) R(30,31) R(31,30) R(31,32) R(32,12) R(32,31) R(n1,0) R(n1,n7) R(n2,0) R(n2,n3) R(n3,6) R(n3,7) R(n3,n2) R(n4,13) R(n4,n5) R(n4,n6) R(n5,16) R(n5,n4) R(n6,n4) R(n6,n7) R(n7,n1) R(n7,n6)

-------------------------------------------------------------------------------------------------------------

-> Ur-ecdysozoa = limulus_polyphemus (solution already obtained in "chaetognaths_taxA")

score = 290:

model 16:

-------------

R(0,1) R(0,9) R(0,10) R(0,18) R(0,19) R(0,25) R(0,26) R(0,29) R(0,n1) R(0,n2) R(1,0) R(2,18) R(3,20) R(3,21) R(4,22) R(5,24) R(6,17) R(6,n1) R(6,n3) R(7,n3) R(8,25) R(9,0) R(10,0) R(11,28) R(12,32) R(13,14) R(13,15) R(13,n4) R(14,13) R(15,13) R(16,n5) R(17,6) R(18,0) R(18,2) R(19,0) R(19,20) R(20,3) R(20,19) R(20,23) R(21,3) R(21,22) R(22,4) R(22,21) R(23,20) R(23,24) R(24,5) R(24,23) R(25,0) R(25,8) R(26,0) R(26,27) R(27,26) R(27,28) R(28,11) R(28,27) R(29,0) R(29,30) R(30,29) R(30,31) R(31,30) R(31,32) R(32,12) R(32,31) R(n1,0) R(n1,6) R(n2,0) R(n2,n6) R(n3,6) R(n3,7) R(n4,13) R(n4,n6) R(n4,n7) R(n5,16) R(n5,n7) R(n6,n2) R(n6,n4) R(n7,n4) R(n7,n5)

-------------------------------------------------------------------------------------------------------------

-> Ur-ecdysozoa = limulus_polyphemus (solution already obtained in "chaetognaths_taxA")

score = 305:

model 17:

-------------

R(0,1) R(0,9) R(0,10) R(0,18) R(0,19) R(0,25) R(0,26) R(0,29) R(0,n1) R(0,n2) R(1,0) R(2,18) R(3,20) R(3,21) R(4,22) R(5,24) R(6,17) R(6,n3) R(7,n3) R(8,25) R(9,0) R(10,0) R(11,28) R(12,32) R(13,14) R(13,15) R(13,n4) R(14,13) R(15,13) R(16,n5) R(17,6) R(18,0) R(18,2) R(19,0) R(19,20) R(20,3) R(20,19) R(20,23) R(21,3) R(21,22) R(22,4) R(22,21) R(23,20) R(23,24) R(24,5) R(24,23) R(25,0) R(25,8) R(26,0) R(26,27) R(27,26) R(27,28) R(28,11) R(28,27) R(29,0) R(29,30) R(30,29) R(30,31) R(31,30) R(31,32) R(32,12) R(32,31) R(n1,0) R(n1,n3) R(n2,0) R(n2,n6) R(n3,6) R(n3,7) R(n3,n1) R(n4,13) R(n4,n6) R(n4,n7) R(n5,16) R(n5,n7) R(n6,n2) R(n6,n4) R(n7,n4) R(n7,n5)

-------------------------------------------------------------------------------------------------------------

-> Ur-ecdysozoa = limulus_polyphemus (solution already obtained in "chaetognaths_taxA")

score = 359:

model 18:

-------------

R(0,1) R(0,9) R(0,10) R(0,18) R(0,19) R(0,25) R(0,26) R(0,29) R(0,n1) R(0,n2) R(1,0) R(2,18) R(3,20) R(3,21) R(4,22) R(5,24) R(6,17) R(6,n3) R(7,n3) R(8,25) R(9,0) R(10,0) R(11,28) R(12,32) R(13,14) R(13,n4) R(14,13) R(15,n4) R(15,n6) R(16,n5) R(17,6) R(18,0) R(18,2) R(19,0) R(19,20) R(20,3) R(20,19) R(20,23) R(21,3) R(21,22) R(22,4) R(22,21) R(23,20) R(23,24) R(24,5) R(24,23) R(25,0) R(25,8) R(26,0) R(26,27) R(27,26) R(27,28) R(28,11) R(28,27) R(29,0) R(29,30) R(30,29) R(30,31) R(31,30) R(31,32) R(32,12) R(32,31) R(n1,0) R(n1,n3) R(n2,0) R(n2,n7) R(n3,6) R(n3,7) R(n3,n1) R(n4,13) R(n4,15) R(n4,n5) R(n5,16) R(n5,n4) R(n6,15) R(n6,n7) R(n7,n2) R(n7,n6)

-------------------------------------------------------------------------------------------------------------

-> Ur-ecdysozoa = limulus_polyphemus (solution already obtained in "chaetognaths_taxA")

score = 344:

model 19:

-------------

R(0,1) R(0,9) R(0,10) R(0,18) R(0,19) R(0,25) R(0,26) R(0,29) R(0,n1) R(0,n2) R(1,0) R(2,18) R(3,20) R(3,21) R(4,22) R(5,24) R(6,17) R(6,n1) R(6,n3) R(7,n3) R(8,25) R(9,0) R(10,0) R(11,28) R(12,32) R(13,14) R(13,n4) R(14,13) R(15,n4) R(15,n7) R(16,n5) R(17,6) R(18,0) R(18,2) R(19,0) R(19,20) R(20,3) R(20,19) R(20,23) R(21,3) R(21,22) R(22,4) R(22,21) R(23,20) R(23,24) R(24,5) R(24,23) R(25,0) R(25,8) R(26,0) R(26,27) R(27,26) R(27,28) R(28,11) R(28,27) R(29,0) R(29,30) R(30,29) R(30,31) R(31,30) R(31,32) R(32,12) R(32,31) R(n1,0) R(n1,6) R(n2,0) R(n2,n6) R(n3,6) R(n3,7) R(n4,13) R(n4,15) R(n4,n5) R(n5,16) R(n5,n4) R(n6,n2) R(n6,n7) R(n7,15) R(n7,n6)

-------------------------------------------------------------------------------------------------------------

-> Ur-ecdysozoa = limulus_polyphemus (solution already obtained in "chaetognaths_taxA")

score = 292:

model 20:

-------------

R(0,1) R(0,9) R(0,10) R(0,18) R(0,19) R(0,25) R(0,26) R(0,29) R(0,n1) R(0,n2) R(1,0) R(2,18) R(3,20) R(3,21) R(4,22) R(5,24) R(6,17) R(6,n1) R(6,n3) R(7,n3) R(8,25) R(9,0) R(10,0) R(11,28) R(12,32) R(13,14) R(13,n4) R(14,13) R(15,n4) R(15,n7) R(16,n5) R(17,6) R(18,0) R(18,2) R(19,0) R(19,20) R(20,3) R(20,19) R(20,23) R(21,3) R(21,22) R(22,4) R(22,21) R(23,20) R(23,24) R(24,5) R(24,23) R(25,0) R(25,8) R(26,0) R(26,27) R(27,26) R(27,28) R(28,11) R(28,27) R(29,0) R(29,30) R(30,29) R(30,31) R(31,30) R(31,32) R(32,12) R(32,31) R(n1,0) R(n1,6) R(n2,0) R(n2,n6) R(n3,6) R(n3,7) R(n4,13) R(n4,15) R(n4,n6) R(n5,16) R(n5,n7) R(n6,n2) R(n6,n4) R(n7,15) R(n7,n5)

-------------------------------------------------------------------------------------------------------------

-> Ur-ecdysozoa = limulus_polyphemus (solution already obtained in "chaetognaths_taxA")

score = 277:

model 21:

-------------

R(0,1) R(0,9) R(0,10) R(0,18) R(0,19) R(0,25) R(0,26) R(0,29) R(0,n1) R(0,n2) R(1,0) R(2,18) R(3,20) R(3,21) R(4,22) R(5,24) R(6,17) R(6,n1) R(6,n3) R(7,n3) R(8,25) R(9,0) R(10,0) R(11,28) R(12,32) R(13,14) R(13,n4) R(14,13) R(15,n4) R(16,n5) R(17,6) R(18,0) R(18,2) R(19,0) R(19,20) R(20,3) R(20,19) R(20,23) R(21,3) R(21,22) R(22,4) R(22,21) R(23,20) R(23,24) R(24,5) R(24,23) R(25,0) R(25,8) R(26,0) R(26,27) R(27,26) R(27,28) R(28,11) R(28,27) R(29,0) R(29,30) R(30,29) R(30,31) R(31,30) R(31,32) R(32,12) R(32,31) R(n1,0) R(n1,6) R(n2,0) R(n2,n6) R(n3,6) R(n3,7) R(n4,13) R(n4,15) R(n4,n6) R(n4,n7) R(n5,16) R(n5,n7) R(n6,n2) R(n6,n4) R(n7,n4) R(n7,n5)

-------------------------------------------------------------------------------------------------------------

-> Ur-ecdysozoa = limulus_polyphemus (solution already obtained in "chaetognaths_taxA")

score = 307:

model 22:

-------------

R(0,1) R(0,9) R(0,10) R(0,18) R(0,19) R(0,25) R(0,26) R(0,29) R(0,n1) R(0,n2) R(1,0) R(2,18) R(3,20) R(3,21) R(4,22) R(5,24) R(6,17) R(6,n3) R(7,n3) R(8,25) R(9,0) R(10,0) R(11,28) R(12,32) R(13,14) R(13,n4) R(14,13) R(15,n4) R(15,n6) R(16,n5) R(17,6) R(18,0) R(18,2) R(19,0) R(19,20) R(20,3) R(20,19) R(20,23) R(21,3) R(21,22) R(22,4) R(22,21) R(23,20) R(23,24) R(24,5) R(24,23) R(25,0) R(25,8) R(26,0) R(26,27) R(27,26) R(27,28) R(28,11) R(28,27) R(29,0) R(29,30) R(30,29) R(30,31) R(31,30) R(31,32) R(32,12) R(32,31) R(n1,0) R(n1,n7) R(n2,0) R(n2,n3) R(n3,6) R(n3,7) R(n3,n2) R(n4,13) R(n4,15) R(n4,n7) R(n5,16) R(n5,n6) R(n6,15) R(n6,n5) R(n7,n1) R(n7,n4)

-------------------------------------------------------------------------------------------------------------

-> Ur-ecdysozoa = limulus_polyphemus (solution already obtained in "chaetognaths_taxA")

score = 292:

model 23:

-------------

R(0,1) R(0,9) R(0,10) R(0,18) R(0,19) R(0,25) R(0,26) R(0,29) R(0,n1) R(0,n2) R(1,0) R(2,18) R(3,20) R(3,21) R(4,22) R(5,24) R(6,17) R(6,n3) R(7,n3) R(8,25) R(9,0) R(10,0) R(11,28) R(12,32) R(13,14) R(13,n4) R(14,13) R(15,n4) R(16,n5) R(17,6) R(18,0) R(18,2) R(19,0) R(19,20) R(20,3) R(20,19) R(20,23) R(21,3) R(21,22) R(22,4) R(22,21) R(23,20) R(23,24) R(24,5) R(24,23) R(25,0) R(25,8) R(26,0) R(26,27) R(27,26) R(27,28) R(28,11) R(28,27) R(29,0) R(29,30) R(30,29) R(30,31) R(31,30) R(31,32) R(32,12) R(32,31) R(n1,0) R(n1,n3) R(n2,0) R(n2,n7) R(n3,6) R(n3,7) R(n3,n1) R(n4,13) R(n4,15) R(n4,n6) R(n4,n7) R(n5,16) R(n5,n6) R(n6,n4) R(n6,n5) R(n7,n2) R(n7,n4)

-------------------------------------------------------------------------------------------------------------

-> Ur-ecdysozoa = limulus_polyphemus (solution already obtained in "chaetognaths_taxA")

score = 305:

model 24:

-------------

R(0,1) R(0,9) R(0,10) R(0,18) R(0,19) R(0,25) R(0,26) R(0,29) R(0,n1) R(0,n2) R(1,0) R(2,18) R(3,20) R(3,21) R(4,22) R(5,24) R(6,17) R(6,n2) R(6,n3) R(7,n3) R(8,25) R(9,0) R(10,0) R(11,28) R(12,32) R(13,14) R(13,n4) R(14,13) R(15,n6) R(16,n5) R(17,6) R(18,0) R(18,2) R(19,0) R(19,20) R(20,3) R(20,19) R(20,23) R(21,3) R(21,22) R(22,4) R(22,21) R(23,20) R(23,24) R(24,5) R(24,23) R(25,0) R(25,8) R(26,0) R(26,27) R(27,26) R(27,28) R(28,11) R(28,27) R(29,0) R(29,30) R(30,29) R(30,31) R(31,30) R(31,32) R(32,12) R(32,31) R(n1,0) R(n1,n7) R(n2,0) R(n2,6) R(n3,6) R(n3,7) R(n4,13) R(n4,n5) R(n4,n6) R(n5,16) R(n5,n4) R(n6,15) R(n6,n4) R(n6,n7) R(n7,n1) R(n7,n6)

-------------------------------------------------------------------------------------------------------------

-> Ur-ecdysozoa = limulus_polyphemus (solution already obtained in "chaetognaths_taxA")

score = 320:

model 25:

-------------

R(0,1) R(0,9) R(0,10) R(0,18) R(0,19) R(0,25) R(0,26) R(0,29) R(0,n1) R(0,n2) R(1,0) R(2,18) R(3,20) R(3,21) R(4,22) R(5,24) R(6,17) R(6,n3) R(7,n3) R(8,25) R(9,0) R(10,0) R(11,28) R(12,32) R(13,14) R(13,n4) R(14,13) R(15,n6) R(16,n5) R(17,6) R(18,0) R(18,2) R(19,0) R(19,20) R(20,3) R(20,19) R(20,23) R(21,3) R(21,22) R(22,4) R(22,21) R(23,20) R(23,24) R(24,5) R(24,23) R(25,0) R(25,8) R(26,0) R(26,27) R(27,26) R(27,28) R(28,11) R(28,27) R(29,0) R(29,30) R(30,29) R(30,31) R(31,30) R(31,32) R(32,12) R(32,31) R(n1,0) R(n1,n7) R(n2,0) R(n2,n3) R(n3,6) R(n3,7) R(n3,n2) R(n4,13) R(n4,n5) R(n4,n6) R(n5,16) R(n5,n4) R(n6,15) R(n6,n4) R(n6,n7) R(n7,n1) R(n7,n6)

-------------------------------------------------------------------------------------------------------------

-> form outgroup BILA15 - A

n3(mod26)

[ cox1 cox2 atp8 atp6 cox3 nad3 -nad5 -nad4 -nad4L nad6 cob rrnS rrnL nad1 nad2 ]

n4(mod26)

[ cox1 cox2 nad1 nad3 nad2 cox3 rrnS -nad4L -nad4 -nad5 -cob -nad6 rrnL ]

score = 344:

model 26:

-------------

R(0,1) R(0,9) R(0,10) R(0,18) R(0,19) R(0,25) R(0,26) R(0,29) R(0,n1) R(0,n2) R(1,0) R(2,18) R(3,20) R(3,21) R(4,22) R(5,24) R(6,n3) R(7,n3) R(8,25) R(9,0) R(10,0) R(11,28) R(12,32) R(13,14) R(13,n4) R(14,13) R(15,n4) R(15,n6) R(16,n5) R(17,n3) R(18,0) R(18,2) R(19,0) R(19,20) R(20,3) R(20,19) R(20,23) R(21,3) R(21,22) R(22,4) R(22,21) R(23,20) R(23,24) R(24,5) R(24,23) R(25,0) R(25,8) R(26,0) R(26,27) R(27,26) R(27,28) R(28,11) R(28,27) R(29,0) R(29,30) R(30,29) R(30,31) R(31,30) R(31,32) R(32,12) R(32,31) R(n1,0) R(n1,n3) R(n2,0) R(n2,n7) R(n3,6) R(n3,7) R(n3,17) R(n3,n1) R(n4,13) R(n4,15) R(n4,n5) R(n5,16) R(n5,n4) R(n6,15) R(n6,n7) R(n7,n2) R(n7,n6)

-------------------------------------------------------------------------------------------------------------

-> form outgroup BILA15 - A

n3(mod27)

[ cox1 cox2 atp8 atp6 cox3 nad3 -nad5 -nad4 -nad4L nad6 cob rrnS rrnL nad1 nad2 ]

n4(mod27)

[ cox1 cox2 nad1 nad3 nad2 cox3 rrnS -nad5 -nad4 -nad4L -cob -nad6 rrnL ]

score = 292:

model 27:

-------------

R(0,1) R(0,9) R(0,10) R(0,18) R(0,19) R(0,25) R(0,26) R(0,29) R(0,n1) R(0,n2) R(1,0) R(2,18) R(3,20) R(3,21) R(4,22) R(5,24) R(6,n3) R(7,n3) R(8,25) R(9,0) R(10,0) R(11,28) R(12,32) R(13,14) R(13,n4) R(14,13) R(15,n4) R(15,n6) R(16,n5) R(17,n3) R(18,0) R(18,2) R(19,0) R(19,20) R(20,3) R(20,19) R(20,23) R(21,3) R(21,22) R(22,4) R(22,21) R(23,20) R(23,24) R(24,5) R(24,23) R(25,0) R(25,8) R(26,0) R(26,27) R(27,26) R(27,28) R(28,11) R(28,27) R(29,0) R(29,30) R(30,29) R(30,31) R(31,30) R(31,32) R(32,12) R(32,31) R(n1,0) R(n1,n7) R(n2,0) R(n2,n3) R(n3,6) R(n3,7) R(n3,17) R(n3,n2) R(n4,13) R(n4,15) R(n4,n7) R(n5,16) R(n5,n6) R(n6,15) R(n6,n5) R(n7,n1) R(n7,n4)

-------------------------------------------------------------------------------------------------------------

-> form outgroup BILA15 - A

n3(mod28)

[ cox1 cox2 atp8 atp6 cox3 nad3 -nad5 -nad4 -nad4L nad6 cob rrnS rrnL nad1 nad2 ]

n4(mod28)

[ cox1 cox2 nad1 nad3 nad2 cox3 rrnS -nad5 -nad4 -nad4L -cob -nad6 rrnL ]

score = 277:

model 28:

-------------

R(0,1) R(0,9) R(0,10) R(0,18) R(0,19) R(0,25) R(0,26) R(0,29) R(0,n1) R(0,n2) R(1,0) R(2,18) R(3,20) R(3,21) R(4,22) R(5,24) R(6,n3) R(7,n3) R(8,25) R(9,0) R(10,0) R(11,28) R(12,32) R(13,14) R(13,n4) R(14,13) R(15,n4) R(16,n5) R(17,n3) R(18,0) R(18,2) R(19,0) R(19,20) R(20,3) R(20,19) R(20,23) R(21,3) R(21,22) R(22,4) R(22,21) R(23,20) R(23,24) R(24,5) R(24,23) R(25,0) R(25,8) R(26,0) R(26,27) R(27,26) R(27,28) R(28,11) R(28,27) R(29,0) R(29,30) R(30,29) R(30,31) R(31,30) R(31,32) R(32,12) R(32,31) R(n1,0) R(n1,n3) R(n2,0) R(n2,n7) R(n3,6) R(n3,7) R(n3,17) R(n3,n1) R(n4,13) R(n4,15) R(n4,n6) R(n4,n7) R(n5,16) R(n5,n6) R(n6,n4) R(n6,n5) R(n7,n2) R(n7,n4)

-------------------------------------------------------------------------------------------------------------

-> form outgroup BILA15 - A

n3(mod29)

[ cox1 cox2 atp8 atp6 cox3 nad3 -nad5 -nad4 -nad4L nad6 cob rrnS rrnL nad1 nad2 ]

n4(mod29)

g1: [ cox1 cox2 nad1 nad3 nad2 cox3 rrnS -nad4 -nad5 -cob -nad6 -nad4L rrnL ]

g2: [ cox1 cox2 nad1 nad3 nad2 cox3 rrnS -nad4 -nad5 -cob -nad6 rrnL -nad4L ]

g3: [ cox1 cox2 nad1 nad3 nad2 cox3 rrnS nad5 nad4 -nad4L -cob -nad6 rrnL ]

g4: [ cox1 cox2 nad1 nad3 nad2 cox3 rrnS rrnL nad6 cob nad4L nad5 nad4 ]

g5: [ cox1 cox2 nad1 nad3 nad2 cox3 rrnS -rrnL nad6 cob nad4L nad5 nad4 ]

g6: [ cox1 cox2 nad1 nad3 nad2 cox3 rrnS -nad4 -nad5 rrnL nad6 cob nad4L ]

g7: [ cox1 cox2 nad1 nad3 nad2 cox3 rrnS nad6 cob nad4L -nad4 -nad5 rrnL ]

g8: [ cox1 cox2 nad1 nad3 nad2 cox3 rrnS -nad4 -nad5 nad6 cob nad4L rrnL ]

g9: [ cox1 cox2 nad1 nad3 nad2 cox3 rrnS -rrnL nad6 cob nad4L -nad4 -nad5 ]

g10: [ cox1 cox2 nad1 nad3 nad2 cox3 rrnS -nad4 -nad5 -rrnL -nad4L -cob -nad6 ]

g11: [ cox1 cox2 nad1 nad3 nad2 cox3 rrnS -nad4 -nad5 -nad4L -cob -nad6 -rrnL ]

score = 329:

model 29:

-------------

R(0,1) R(0,9) R(0,10) R(0,18) R(0,19) R(0,25) R(0,26) R(0,29) R(0,n1) R(0,n2) R(1,0) R(2,18) R(3,20) R(3,21) R(4,22) R(5,24) R(6,n3) R(7,n3) R(8,25) R(9,0) R(10,0) R(11,28) R(12,32) R(13,14) R(13,15) R(13,n4) R(14,13) R(15,13) R(16,n5) R(17,n3) R(18,0) R(18,2) R(19,0) R(19,20) R(20,3) R(20,19) R(20,23) R(21,3) R(21,22) R(22,4) R(22,21) R(23,20) R(23,24) R(24,5) R(24,23) R(25,0) R(25,8) R(26,0) R(26,27) R(27,26) R(27,28) R(28,11) R(28,27) R(29,0) R(29,30) R(30,29) R(30,31) R(31,30) R(31,32) R(32,12) R(32,31) R(n1,0) R(n1,n7) R(n2,0) R(n2,n3) R(n3,6) R(n3,7) R(n3,17) R(n3,n2) R(n4,13) R(n4,n5) R(n4,n6) R(n5,16) R(n5,n4) R(n6,n4) R(n6,n7) R(n7,n1) R(n7,n6)

-------------------------------------------------------------------------------------------------------------

-> form outgroup BILA15 - A

n3(mod30)

[ cox1 cox2 atp8 atp6 cox3 nad3 -nad5 -nad4 -nad4L nad6 cob rrnS rrnL nad1 nad2 ]

score = 329:

model 30:

-------------

R(0,1) R(0,9) R(0,10) R(0,18) R(0,19) R(0,25) R(0,26) R(0,29) R(0,n1) R(0,n2) R(1,0) R(2,18) R(3,20) R(3,21) R(4,22) R(5,24) R(6,n3) R(7,n3) R(8,25) R(9,0) R(10,0) R(11,28) R(12,32) R(13,14) R(13,15) R(13,n4) R(14,13) R(15,13) R(15,n6) R(16,n5) R(17,n3) R(18,0) R(18,2) R(19,0) R(19,20) R(20,3) R(20,19) R(20,23) R(21,3) R(21,22) R(22,4) R(22,21) R(23,20) R(23,24) R(24,5) R(24,23) R(25,0) R(25,8) R(26,0) R(26,27) R(27,26) R(27,28) R(28,11) R(28,27) R(29,0) R(29,30) R(30,29) R(30,31) R(31,30) R(31,32) R(32,12) R(32,31) R(n1,0) R(n1,n3) R(n2,0) R(n2,n7) R(n3,6) R(n3,7) R(n3,17) R(n3,n1) R(n4,13) R(n4,n5) R(n5,16) R(n5,n4) R(n6,15) R(n6,n7) R(n7,n2) R(n7,n6)

-------------------------------------------------------------------------------------------------------------

-> form outgroup BILA15 - A

n3(mod31)

[ cox1 cox2 atp8 atp6 cox3 nad3 -nad5 -nad4 -nad4L nad6 cob rrnS rrnL nad1 nad2 ]

score = 316:

model 31:

-------------

R(0,1) R(0,9) R(0,10) R(0,18) R(0,19) R(0,25) R(0,26) R(0,29) R(0,n1) R(0,n2) R(1,0) R(2,18) R(3,20) R(3,21) R(4,22) R(5,24) R(6,n3) R(7,n3) R(8,25) R(9,0) R(10,0) R(11,28) R(12,32) R(13,14) R(13,15) R(13,n4) R(14,13) R(15,13) R(15,n6) R(16,n5) R(17,n3) R(18,0) R(18,2) R(19,0) R(19,20) R(20,3) R(20,19) R(20,23) R(21,3) R(21,22) R(22,4) R(22,21) R(23,20) R(23,24) R(24,5) R(24,23) R(25,0) R(25,8) R(26,0) R(26,27) R(27,26) R(27,28) R(28,11) R(28,27) R(29,0) R(29,30) R(30,29) R(30,31) R(31,30) R(31,32) R(32,12) R(32,31) R(n1,0) R(n1,n7) R(n2,0) R(n2,n3) R(n3,6) R(n3,7) R(n3,17) R(n3,n2) R(n4,13) R(n4,n7) R(n5,16) R(n5,n6) R(n6,15) R(n6,n5) R(n7,n1) R(n7,n4)

-------------------------------------------------------------------------------------------------------------

-> form outgroup BILA15 - A

n3(mod32)

[ cox1 cox2 atp8 atp6 cox3 nad3 -nad5 -nad4 -nad4L nad6 cob rrnS rrnL nad1 nad2 ]

n4(mod32)

g1: [ cox1 cox2 -nad4 -nad5 -nad4L -cob -nad6 nad1 nad3 nad2 cox3 rrnS rrnL ]

g2: [ cox1 cox2 -nad4 -nad5 -nad4L -cob -nad6 rrnL nad1 nad3 nad2 cox3 rrnS ]

g3: [ cox1 cox2 nad1 nad3 nad2 -nad4 -nad5 -nad4L -cob -nad6 cox3 rrnS rrnL ]

g4: [ cox1 cox2 nad1 nad3 nad2 cox3 rrnS -nad5 -nad4 -nad4L -cob -nad6 rrnL ]

g5: [ cox1 cox2 nad1 nad6 cob nad4L nad5 nad4 nad3 nad2 cox3 rrnS rrnL ]

score = 290:

model 32:

-------------

R(0,1) R(0,9) R(0,10) R(0,18) R(0,19) R(0,25) R(0,26) R(0,29) R(0,n1) R(0,n2) R(1,0) R(2,18) R(3,20) R(3,21) R(4,22) R(5,24) R(6,n3) R(7,n3) R(8,25) R(9,0) R(10,0) R(11,28) R(12,32) R(13,14) R(13,15) R(13,n4) R(14,13) R(15,13) R(16,n5) R(17,n3) R(18,0) R(18,2) R(19,0) R(19,20) R(20,3) R(20,19) R(20,23) R(21,3) R(21,22) R(22,4) R(22,21) R(23,20) R(23,24) R(24,5) R(24,23) R(25,0) R(25,8) R(26,0) R(26,27) R(27,26) R(27,28) R(28,11) R(28,27) R(29,0) R(29,30) R(30,29) R(30,31) R(31,30) R(31,32) R(32,12) R(32,31) R(n1,0) R(n1,n3) R(n2,0) R(n2,n7) R(n3,6) R(n3,7) R(n3,17) R(n3,n1) R(n4,13) R(n4,n6) R(n4,n7) R(n5,16) R(n5,n6) R(n6,n4) R(n6,n5) R(n7,n2) R(n7,n4)

-------------------------------------------------------------------------------------------------------------

-> form outgroup BILA15 - A

n3(mod33)

[ cox1 cox2 atp8 atp6 cox3 nad3 -nad5 -nad4 -nad4L nad6 cob rrnS rrnL nad1 nad2 ]

n6(mod33)

[ cox1 cox2 nad1 nad3 nad2 cox3 rrnS -nad5 -nad4 -nad4L -cob -nad6 rrnL ]

n4(mod33)

[ cox1 cox2 nad1 nad3 nad2 cox3 rrnS nad5 nad4 -nad4L -cob -nad6 rrnL ]

score = 305:

model 33:

-------------

R(0,1) R(0,9) R(0,10) R(0,18) R(0,19) R(0,25) R(0,26) R(0,29) R(0,n1) R(0,n2) R(1,0) R(2,18) R(3,20) R(3,21) R(4,22) R(5,24) R(6,n3) R(7,n3) R(8,25) R(9,0) R(10,0) R(11,28) R(12,32) R(13,14) R(13,n4) R(14,13) R(15,n6) R(16,n5) R(17,n3) R(18,0) R(18,2) R(19,0) R(19,20) R(20,3) R(20,19) R(20,23) R(21,3) R(21,22) R(22,4) R(22,21) R(23,20) R(23,24) R(24,5) R(24,23) R(25,0) R(25,8) R(26,0) R(26,27) R(27,26) R(27,28) R(28,11) R(28,27) R(29,0) R(29,30) R(30,29) R(30,31) R(31,30) R(31,32) R(32,12) R(32,31) R(n1,0) R(n1,n7) R(n2,0) R(n2,n3) R(n3,6) R(n3,7) R(n3,17) R(n3,n2) R(n4,13) R(n4,n5) R(n4,n6) R(n5,16) R(n5,n4) R(n6,15) R(n6,n4) R(n6,n7) R(n7,n1) R(n7,n6)

-------------------------------------------------------------------------------------------------------------

-> form outgroup BILA15 - A

n3(mod34)

[ cox1 cox2 atp8 atp6 cox3 nad3 -nad5 -nad4 -nad4L nad6 cob rrnS rrnL nad1 nad2 ]

score = 316:

model 34:

-------------

R(0,1) R(0,9) R(0,10) R(0,18) R(0,19) R(0,25) R(0,26) R(0,29) R(0,n1) R(0,n2) R(1,0) R(2,18) R(3,20) R(3,21) R(4,22) R(5,24) R(6,n3) R(7,n3) R(8,25) R(9,0) R(10,0) R(11,28) R(12,32) R(13,14) R(13,15) R(14,13) R(15,13) R(15,n4) R(15,n5) R(16,n6) R(17,n3) R(18,0) R(18,2) R(19,0) R(19,20) R(20,3) R(20,19) R(20,23) R(21,3) R(21,22) R(22,4) R(22,21) R(23,20) R(23,24) R(24,5) R(24,23) R(25,0) R(25,8) R(26,0) R(26,27) R(27,26) R(27,28) R(28,11) R(28,27) R(29,0) R(29,30) R(30,29) R(30,31) R(31,30) R(31,32) R(32,12) R(32,31) R(n1,0) R(n1,n7) R(n2,0) R(n2,n3) R(n3,6) R(n3,7) R(n3,17) R(n3,n2) R(n4,15) R(n4,n6) R(n5,15) R(n5,n7) R(n6,16) R(n6,n4) R(n7,n1) R(n7,n5)

-------------------------------------------------------------------------------------------------------------

-> Ur-ecdysozoa = limulus_polyphemus (solution already obtained in "chaetognaths_taxA")

score = 316:

model 35:

-------------

R(0,1) R(0,9) R(0,10) R(0,18) R(0,19) R(0,25) R(0,26) R(0,29) R(0,n1) R(0,n2) R(1,0) R(2,18) R(3,20) R(3,21) R(4,22) R(5,24) R(6,17) R(6,n2) R(6,n3) R(7,n3) R(8,25) R(9,0) R(10,0) R(11,28) R(12,32) R(13,14) R(13,15) R(14,13) R(15,13) R(15,n4) R(15,n5) R(16,n6) R(17,6) R(18,0) R(18,2) R(19,0) R(19,20) R(20,3) R(20,19) R(20,23) R(21,3) R(21,22) R(22,4) R(22,21) R(23,20) R(23,24) R(24,5) R(24,23) R(25,0) R(25,8) R(26,0) R(26,27) R(27,26) R(27,28) R(28,11) R(28,27) R(29,0) R(29,30) R(30,29) R(30,31) R(31,30) R(31,32) R(32,12) R(32,31) R(n1,0) R(n1,n7) R(n2,0) R(n2,6) R(n3,6) R(n3,7) R(n4,15) R(n4,n6) R(n5,15) R(n5,n7) R(n6,16) R(n6,n4) R(n7,n1) R(n7,n5)

-------------------------------------------------------------------------------------------------------------

-> Ur-ecdysozoa = limulus_polyphemus (solution already obtained in "chaetognaths_taxA")

score = 331:

model 36:

-------------

R(0,1) R(0,9) R(0,10) R(0,18) R(0,19) R(0,25) R(0,26) R(0,29) R(0,n1) R(0,n2) R(1,0) R(2,18) R(3,20) R(3,21) R(4,22) R(5,24) R(6,17) R(6,n3) R(7,n3) R(8,25) R(9,0) R(10,0) R(11,28) R(12,32) R(13,14) R(13,15) R(14,13) R(15,13) R(15,n4) R(15,n5) R(16,n6) R(17,6) R(18,0) R(18,2) R(19,0) R(19,20) R(20,3) R(20,19) R(20,23) R(21,3) R(21,22) R(22,4) R(22,21) R(23,20) R(23,24) R(24,5) R(24,23) R(25,0) R(25,8) R(26,0) R(26,27) R(27,26) R(27,28) R(28,11) R(28,27) R(29,0) R(29,30) R(30,29) R(30,31) R(31,30) R(31,32) R(32,12) R(32,31) R(n1,0) R(n1,n7) R(n2,0) R(n2,n3) R(n3,6) R(n3,7) R(n3,n2) R(n4,15) R(n4,n6) R(n5,15) R(n5,n7) R(n6,16) R(n6,n4) R(n7,n1) R(n7,n5)

-------------------------------------------------------------------------------------------------------------

-> Ur-ecdysozoa = limulus_polyphemus (solution already obtained in "chaetognaths_taxA")

score = 305:

model 37:

-------------

R(0,1) R(0,9) R(0,10) R(0,18) R(0,19) R(0,25) R(0,26) R(0,29) R(0,n1) R(0,n2) R(1,0) R(2,18) R(3,20) R(3,21) R(4,22) R(5,24) R(6,17) R(6,n1) R(6,n3) R(7,n3) R(8,25) R(9,0) R(10,0) R(11,28) R(12,32) R(13,14) R(13,15) R(14,13) R(15,13) R(15,n4) R(16,n5) R(17,6) R(18,0) R(18,2) R(19,0) R(19,20) R(20,3) R(20,19) R(20,23) R(21,3) R(21,22) R(22,4) R(22,21) R(23,20) R(23,24) R(24,5) R(24,23) R(25,0) R(25,8) R(26,0) R(26,27) R(27,26) R(27,28) R(28,11) R(28,27) R(29,0) R(29,30) R(30,29) R(30,31) R(31,30) R(31,32) R(32,12) R(32,31) R(n1,0) R(n1,6) R(n2,0) R(n2,n6) R(n3,6) R(n3,7) R(n4,15) R(n4,n6) R(n4,n7) R(n5,16) R(n5,n7) R(n6,n2) R(n6,n4) R(n7,n4) R(n7,n5)

-------------------------------------------------------------------------------------------------------------

-> Ur-ecdysozoa = limulus_polyphemus (solution already obtained in "chaetognaths_taxA")

score = 320:

model 38:

-------------

R(0,1) R(0,9) R(0,10) R(0,18) R(0,19) R(0,25) R(0,26) R(0,29) R(0,n1) R(0,n2) R(1,0) R(2,18) R(3,20) R(3,21) R(4,22) R(5,24) R(6,17) R(6,n3) R(7,n3) R(8,25) R(9,0) R(10,0) R(11,28) R(12,32) R(13,14) R(13,15) R(14,13) R(15,13) R(15,n4) R(16,n5) R(17,6) R(18,0) R(18,2) R(19,0) R(19,20) R(20,3) R(20,19) R(20,23) R(21,3) R(21,22) R(22,4) R(22,21) R(23,20) R(23,24) R(24,5) R(24,23) R(25,0) R(25,8) R(26,0) R(26,27) R(27,26) R(27,28) R(28,11) R(28,27) R(29,0) R(29,30) R(30,29) R(30,31) R(31,30) R(31,32) R(32,12) R(32,31) R(n1,0) R(n1,n3) R(n2,0) R(n2,n6) R(n3,6) R(n3,7) R(n3,n1) R(n4,15) R(n4,n6) R(n4,n7) R(n5,16) R(n5,n7) R(n6,n2) R(n6,n4) R(n7,n4) R(n7,n5)

-------------------------------------------------------------------------------------------------------------

-> form outgroup BILA15 - A

n3(mod39)

[ cox1 cox2 atp8 atp6 cox3 nad3 -nad5 -nad4 -nad4L nad6 cob rrnS rrnL nad1 nad2 ]

n4(mod39)

[ cox1 cox2 nad1 nad3 nad2 cox3 rrnS -nad5 -nad4 -nad4L -cob -nad6 rrnL ]

score = 305:

model 39:

-------------

R(0,1) R(0,9) R(0,10) R(0,18) R(0,19) R(0,25) R(0,26) R(0,29) R(0,n1) R(0,n2) R(1,0) R(2,18) R(3,20) R(3,21) R(4,22) R(5,24) R(6,n3) R(7,n3) R(8,25) R(9,0) R(10,0) R(11,28) R(12,32) R(13,14) R(13,15) R(14,13) R(15,13) R(15,n4) R(16,n5) R(17,n3) R(18,0) R(18,2) R(19,0) R(19,20) R(20,3) R(20,19) R(20,23) R(21,3) R(21,22) R(22,4) R(22,21) R(23,20) R(23,24) R(24,5) R(24,23) R(25,0) R(25,8) R(26,0) R(26,27) R(27,26) R(27,28) R(28,11) R(28,27) R(29,0) R(29,30) R(30,29) R(30,31) R(31,30) R(31,32) R(32,12) R(32,31) R(n1,0) R(n1,n3) R(n2,0) R(n2,n7) R(n3,6) R(n3,7) R(n3,17) R(n3,n1) R(n4,15) R(n4,n6) R(n4,n7) R(n5,16) R(n5,n6) R(n6,n4) R(n6,n5) R(n7,n2) R(n7,n4)

-------------------------------------------------------------------------------------------------------------

-> Ur-ecdysozoa = priapulus_caudatus (solution already obtained in "chaetognaths_taxB")

score = 301:

model 40:

-------------

R(0,1) R(0,9) R(0,10) R(0,18) R(0,19) R(0,25) R(0,26) R(0,29) R(0,n1) R(0,n2) R(1,0) R(2,18) R(3,20) R(3,21) R(4,22) R(5,24) R(6,17) R(7,n3) R(8,25) R(9,0) R(10,0) R(11,28) R(12,32) R(13,14) R(13,15) R(13,n4) R(13,n5) R(14,13) R(15,13) R(16,n6) R(17,6) R(17,n2) R(17,n3) R(18,0) R(18,2) R(19,0) R(19,20) R(20,3) R(20,19) R(20,23) R(21,3) R(21,22) R(22,4) R(22,21) R(23,20) R(23,24) R(24,5) R(24,23) R(25,0) R(25,8) R(26,0) R(26,27) R(27,26) R(27,28) R(28,11) R(28,27) R(29,0) R(29,30) R(30,29) R(30,31) R(31,30) R(31,32) R(32,12) R(32,31) R(n1,0) R(n1,n7) R(n2,0) R(n2,17) R(n3,7) R(n3,17) R(n4,13) R(n4,n6) R(n5,13) R(n5,n7) R(n6,16) R(n6,n4) R(n7,n1) R(n7,n5)

-------------------------------------------------------------------------------------------------------------

-> Ur-ecdysozoa = priapulus_caudatus (solution already obtained in "chaetognaths_taxB")

score = 316:

model 41:

-------------

R(0,1) R(0,9) R(0,10) R(0,18) R(0,19) R(0,25) R(0,26) R(0,29) R(0,n1) R(0,n2) R(1,0) R(2,18) R(3,20) R(3,21) R(4,22) R(5,24) R(6,17) R(7,n3) R(8,25) R(9,0) R(10,0) R(11,28) R(12,32) R(13,14) R(13,15) R(13,n4) R(13,n5) R(14,13) R(15,13) R(16,n6) R(17,6) R(17,n3) R(18,0) R(18,2) R(19,0) R(19,20) R(20,3) R(20,19) R(20,23) R(21,3) R(21,22) R(22,4) R(22,21) R(23,20) R(23,24) R(24,5) R(24,23) R(25,0) R(25,8) R(26,0) R(26,27) R(27,26) R(27,28) R(28,11) R(28,27) R(29,0) R(29,30) R(30,29) R(30,31) R(31,30) R(31,32) R(32,12) R(32,31) R(n1,0) R(n1,n7) R(n2,0) R(n2,n3) R(n3,7) R(n3,17) R(n3,n2) R(n4,13) R(n4,n6) R(n5,13) R(n5,n7) R(n6,16) R(n6,n4) R(n7,n1) R(n7,n5)

-------------------------------------------------------------------------------------------------------------

-> Ur-ecdysozoa = priapulus_caudatus (solution already obtained in "chaetognaths_taxB")

score = 316:

model 42:

-------------

R(0,1) R(0,9) R(0,10) R(0,18) R(0,19) R(0,25) R(0,26) R(0,29) R(0,n1) R(0,n2) R(1,0) R(2,18) R(3,20) R(3,21) R(4,22) R(5,24) R(6,17) R(7,n3) R(8,25) R(9,0) R(10,0) R(11,28) R(12,32) R(13,14) R(13,n4) R(13,n5) R(14,13) R(15,n4) R(16,n6) R(17,6) R(17,n2) R(17,n3) R(18,0) R(18,2) R(19,0) R(19,20) R(20,3) R(20,19) R(20,23) R(21,3) R(21,22) R(22,4) R(22,21) R(23,20) R(23,24) R(24,5) R(24,23) R(25,0) R(25,8) R(26,0) R(26,27) R(27,26) R(27,28) R(28,11) R(28,27) R(29,0) R(29,30) R(30,29) R(30,31) R(31,30) R(31,32) R(32,12) R(32,31) R(n1,0) R(n1,n7) R(n2,0) R(n2,17) R(n3,7) R(n3,17) R(n4,13) R(n4,15) R(n4,n6) R(n5,13) R(n5,n7) R(n6,16) R(n6,n4) R(n7,n1) R(n7,n5)

-------------------------------------------------------------------------------------------------------------

-> Ur-ecdysozoa = priapulus_caudatus (solution already obtained in "chaetognaths_taxB")

score = 331:

model 43:

-------------

R(0,1) R(0,9) R(0,10) R(0,18) R(0,19) R(0,25) R(0,26) R(0,29) R(0,n1) R(0,n2) R(1,0) R(2,18) R(3,20) R(3,21) R(4,22) R(5,24) R(6,17) R(7,n3) R(8,25) R(9,0) R(10,0) R(11,28) R(12,32) R(13,14) R(13,n4) R(13,n5) R(14,13) R(15,n4) R(16,n6) R(17,6) R(17,n3) R(18,0) R(18,2) R(19,0) R(19,20) R(20,3) R(20,19) R(20,23) R(21,3) R(21,22) R(22,4) R(22,21) R(23,20) R(23,24) R(24,5) R(24,23) R(25,0) R(25,8) R(26,0) R(26,27) R(27,26) R(27,28) R(28,11) R(28,27) R(29,0) R(29,30) R(30,29) R(30,31) R(31,30) R(31,32) R(32,12) R(32,31) R(n1,0) R(n1,n7) R(n2,0) R(n2,n3) R(n3,7) R(n3,17) R(n3,n2) R(n4,13) R(n4,15) R(n4,n6) R(n5,13) R(n5,n7) R(n6,16) R(n6,n4) R(n7,n1) R(n7,n5)

-------------------------------------------------------------------------------------------------------------

-> Ur-ecdysozoa = priapulus_caudatus (solution already obtained in "chaetognaths_taxB")

score = 290:

model 44:

-------------

R(0,1) R(0,9) R(0,10) R(0,18) R(0,19) R(0,25) R(0,26) R(0,29) R(0,n1) R(0,n2) R(1,0) R(2,18) R(3,20) R(3,21) R(4,22) R(5,24) R(6,17) R(7,n3) R(8,25) R(9,0) R(10,0) R(11,28) R(12,32) R(13,14) R(13,n4) R(13,n5) R(14,13) R(15,n5) R(16,n6) R(17,6) R(17,n2) R(17,n3) R(18,0) R(18,2) R(19,0) R(19,20) R(20,3) R(20,19) R(20,23) R(21,3) R(21,22) R(22,4) R(22,21) R(23,20) R(23,24) R(24,5) R(24,23) R(25,0) R(25,8) R(26,0) R(26,27) R(27,26) R(27,28) R(28,11) R(28,27) R(29,0) R(29,30) R(30,29) R(30,31) R(31,30) R(31,32) R(32,12) R(32,31) R(n1,0) R(n1,n7) R(n2,0) R(n2,17) R(n3,7) R(n3,17) R(n4,13) R(n4,n6) R(n5,13) R(n5,15) R(n5,n7) R(n6,16) R(n6,n4) R(n7,n1) R(n7,n5)

-------------------------------------------------------------------------------------------------------------

-> Ur-ecdysozoa = priapulus_caudatus (solution already obtained in "chaetognaths_taxB")

score = 305:

model 45:

-------------

R(0,1) R(0,9) R(0,10) R(0,18) R(0,19) R(0,25) R(0,26) R(0,29) R(0,n1) R(0,n2) R(1,0) R(2,18) R(3,20) R(3,21) R(4,22) R(5,24) R(6,17) R(7,n3) R(8,25) R(9,0) R(10,0) R(11,28) R(12,32) R(13,14) R(13,n4) R(13,n5) R(14,13) R(15,n5) R(16,n6) R(17,6) R(17,n3) R(18,0) R(18,2) R(19,0) R(19,20) R(20,3) R(20,19) R(20,23) R(21,3) R(21,22) R(22,4) R(22,21) R(23,20) R(23,24) R(24,5) R(24,23) R(25,0) R(25,8) R(26,0) R(26,27) R(27,26) R(27,28) R(28,11) R(28,27) R(29,0) R(29,30) R(30,29) R(30,31) R(31,30) R(31,32) R(32,12) R(32,31) R(n1,0) R(n1,n7) R(n2,0) R(n2,n3) R(n3,7) R(n3,17) R(n3,n2) R(n4,13) R(n4,n6) R(n5,13) R(n5,15) R(n5,n7) R(n6,16) R(n6,n4) R(n7,n1) R(n7,n5)

-------------------------------------------------------------------------------------------------------------

-> Ur-ecdysozoa = limulus_polyphemus (solution already obtained in "chaetognaths_taxA")

score = 288:

model 46:

-------------

R(0,1) R(0,9) R(0,10) R(0,18) R(0,19) R(0,25) R(0,26) R(0,29) R(0,n1) R(0,n2) R(1,0) R(2,18) R(3,20) R(3,21) R(4,22) R(5,24) R(6,17) R(6,n2) R(7,n3) R(8,25) R(9,0) R(10,0) R(11,28) R(12,32) R(13,14) R(13,15) R(13,n4) R(13,n5) R(14,13) R(15,13) R(16,n6) R(17,6) R(18,0) R(18,2) R(19,0) R(19,20) R(20,3) R(20,19) R(20,23) R(21,3) R(21,22) R(22,4) R(22,21) R(23,20) R(23,24) R(24,5) R(24,23) R(25,0) R(25,8) R(26,0) R(26,27) R(27,26) R(27,28) R(28,11) R(28,27) R(29,0) R(29,30) R(30,29) R(30,31) R(31,30) R(31,32) R(32,12) R(32,31) R(n1,0) R(n1,n7) R(n2,0) R(n2,6) R(n2,n3) R(n3,7) R(n3,n2) R(n4,13) R(n4,n6) R(n5,13) R(n5,n7) R(n6,16) R(n6,n4) R(n7,n1) R(n7,n5)

-------------------------------------------------------------------------------------------------------------

-> Ur-ecdysozoa = limulus_polyphemus (solution already obtained in "chaetognaths_taxA")

score = 303:

model 47:

-------------

R(0,1) R(0,9) R(0,10) R(0,18) R(0,19) R(0,25) R(0,26) R(0,29) R(0,n1) R(0,n2) R(1,0) R(2,18) R(3,20) R(3,21) R(4,22) R(5,24) R(6,17) R(6,n2) R(7,n3) R(8,25) R(9,0) R(10,0) R(11,28) R(12,32) R(13,14) R(13,n4) R(13,n5) R(14,13) R(15,n4) R(16,n6) R(17,6) R(18,0) R(18,2) R(19,0) R(19,20) R(20,3) R(20,19) R(20,23) R(21,3) R(21,22) R(22,4) R(22,21) R(23,20) R(23,24) R(24,5) R(24,23) R(25,0) R(25,8) R(26,0) R(26,27) R(27,26) R(27,28) R(28,11) R(28,27) R(29,0) R(29,30) R(30,29) R(30,31) R(31,30) R(31,32) R(32,12) R(32,31) R(n1,0) R(n1,n7) R(n2,0) R(n2,6) R(n2,n3) R(n3,7) R(n3,n2) R(n4,13) R(n4,15) R(n4,n6) R(n5,13) R(n5,n7) R(n6,16) R(n6,n4) R(n7,n1) R(n7,n5)

-------------------------------------------------------------------------------------------------------------

-> Ur-ecdysozoa = limulus_polyphemus (solution already obtained in "chaetognaths_taxA")

score = 277:

model 48:

-------------

R(0,1) R(0,9) R(0,10) R(0,18) R(0,19) R(0,25) R(0,26) R(0,29) R(0,n1) R(0,n2) R(1,0) R(2,18) R(3,20) R(3,21) R(4,22) R(5,24) R(6,17) R(6,n2) R(7,n3) R(8,25) R(9,0) R(10,0) R(11,28) R(12,32) R(13,14) R(13,n4) R(13,n5) R(14,13) R(15,n5) R(16,n6) R(17,6) R(18,0) R(18,2) R(19,0) R(19,20) R(20,3) R(20,19) R(20,23) R(21,3) R(21,22) R(22,4) R(22,21) R(23,20) R(23,24) R(24,5) R(24,23) R(25,0) R(25,8) R(26,0) R(26,27) R(27,26) R(27,28) R(28,11) R(28,27) R(29,0) R(29,30) R(30,29) R(30,31) R(31,30) R(31,32) R(32,12) R(32,31) R(n1,0) R(n1,n7) R(n2,0) R(n2,6) R(n2,n3) R(n3,7) R(n3,n2) R(n4,13) R(n4,n6) R(n5,13) R(n5,15) R(n5,n7) R(n6,16) R(n6,n4) R(n7,n1) R(n7,n5)

-------------------------------------------------------------------------------------------------------------

-> Ur-ecdysozoa = priapulus_caudatus (solution already obtained in "chaetognaths_taxB")

score = 329:

model 49:

-------------

R(0,1) R(0,9) R(0,10) R(0,18) R(0,19) R(0,25) R(0,26) R(0,29) R(0,n1) R(0,n2) R(1,0) R(2,18) R(3,20) R(3,21) R(4,22) R(5,24) R(6,17) R(7,n3) R(8,25) R(9,0) R(10,0) R(11,28) R(12,32) R(13,14) R(13,15) R(13,n4) R(14,13) R(15,13) R(15,n5) R(16,n6) R(17,6) R(17,n2) R(17,n3) R(18,0) R(18,2) R(19,0) R(19,20) R(20,3) R(20,19) R(20,23) R(21,3) R(21,22) R(22,4) R(22,21) R(23,20) R(23,24) R(24,5) R(24,23) R(25,0) R(25,8) R(26,0) R(26,27) R(27,26) R(27,28) R(28,11) R(28,27) R(29,0) R(29,30) R(30,29) R(30,31) R(31,30) R(31,32) R(32,12) R(32,31) R(n1,0) R(n1,n7) R(n2,0) R(n2,17) R(n3,7) R(n3,17) R(n4,13) R(n4,n6) R(n5,15) R(n5,n7) R(n6,16) R(n6,n4) R(n7,n1) R(n7,n5)

-------------------------------------------------------------------------------------------------------------

-> Ur-ecdysozoa = priapulus_caudatus (solution already obtained in "chaetognaths_taxB")

score = 344:

model 50:

-------------

R(0,1) R(0,9) R(0,10) R(0,18) R(0,19) R(0,25) R(0,26) R(0,29) R(0,n1) R(0,n2) R(1,0) R(2,18) R(3,20) R(3,21) R(4,22) R(5,24) R(6,17) R(7,n3) R(8,25) R(9,0) R(10,0) R(11,28) R(12,32) R(13,14) R(13,15) R(13,n4) R(14,13) R(15,13) R(15,n5) R(16,n6) R(17,6) R(17,n3) R(18,0) R(18,2) R(19,0) R(19,20) R(20,3) R(20,19) R(20,23) R(21,3) R(21,22) R(22,4) R(22,21) R(23,20) R(23,24) R(24,5) R(24,23) R(25,0) R(25,8) R(26,0) R(26,27) R(27,26) R(27,28) R(28,11) R(28,27) R(29,0) R(29,30) R(30,29) R(30,31) R(31,30) R(31,32) R(32,12) R(32,31) R(n1,0) R(n1,n7) R(n2,0) R(n2,n3) R(n3,7) R(n3,17) R(n3,n2) R(n4,13) R(n4,n6) R(n5,15) R(n5,n7) R(n6,16) R(n6,n4) R(n7,n1) R(n7,n5)

-------------------------------------------------------------------------------------------------------------

-> Ur-ecdysozoa = priapulus_caudatus (solution already obtained in "chaetognaths_taxB")

score = 316:

model 51:

-------------

R(0,1) R(0,9) R(0,10) R(0,18) R(0,19) R(0,25) R(0,26) R(0,29) R(0,n1) R(0,n2) R(1,0) R(2,18) R(3,20) R(3,21) R(4,22) R(5,24) R(6,17) R(7,n3) R(8,25) R(9,0) R(10,0) R(11,28) R(12,32) R(13,14) R(13,15) R(13,n4) R(14,13) R(15,13) R(15,n5) R(16,n7) R(17,6) R(17,n1) R(17,n3) R(18,0) R(18,2) R(19,0) R(19,20) R(20,3) R(20,19) R(20,23) R(21,3) R(21,22) R(22,4) R(22,21) R(23,20) R(23,24) R(24,5) R(24,23) R(25,0) R(25,8) R(26,0) R(26,27) R(27,26) R(27,28) R(28,11) R(28,27) R(29,0) R(29,30) R(30,29) R(30,31) R(31,30) R(31,32) R(32,12) R(32,31) R(n1,0) R(n1,17) R(n2,0) R(n2,n6) R(n3,7) R(n3,17) R(n4,13) R(n4,n6) R(n5,15) R(n5,n7) R(n6,n2) R(n6,n4) R(n7,16) R(n7,n5)

-------------------------------------------------------------------------------------------------------------

-> Ur-ecdysozoa = priapulus_caudatus (solution already obtained in "chaetognaths_taxB")

score = 331:

model 52:

-------------

R(0,1) R(0,9) R(0,10) R(0,18) R(0,19) R(0,25) R(0,26) R(0,29) R(0,n1) R(0,n2) R(1,0) R(2,18) R(3,20) R(3,21) R(4,22) R(5,24) R(6,17) R(7,n3) R(8,25) R(9,0) R(10,0) R(11,28) R(12,32) R(13,14) R(13,15) R(13,n4) R(14,13) R(15,13) R(15,n5) R(16,n7) R(17,6) R(17,n3) R(18,0) R(18,2) R(19,0) R(19,20) R(20,3) R(20,19) R(20,23) R(21,3) R(21,22) R(22,4) R(22,21) R(23,20) R(23,24) R(24,5) R(24,23) R(25,0) R(25,8) R(26,0) R(26,27) R(27,26) R(27,28) R(28,11) R(28,27) R(29,0) R(29,30) R(30,29) R(30,31) R(31,30) R(31,32) R(32,12) R(32,31) R(n1,0) R(n1,n3) R(n2,0) R(n2,n6) R(n3,7) R(n3,17) R(n3,n1) R(n4,13) R(n4,n6) R(n5,15) R(n5,n7) R(n6,n2) R(n6,n4) R(n7,16) R(n7,n5)

-------------------------------------------------------------------------------------------------------------

-> Ur-ecdysozoa = priapulus_caudatus (solution already obtained in "chaetognaths_taxB")

score = 329:

model 53:

-------------

R(0,1) R(0,9) R(0,10) R(0,18) R(0,19) R(0,25) R(0,26) R(0,29) R(0,n1) R(0,n2) R(1,0) R(2,18) R(3,20) R(3,21) R(4,22) R(5,24) R(6,17) R(7,n3) R(8,25) R(9,0) R(10,0) R(11,28) R(12,32) R(13,14) R(13,15) R(13,n4) R(14,13) R(15,13) R(16,n5) R(17,6) R(17,n2) R(17,n3) R(18,0) R(18,2) R(19,0) R(19,20) R(20,3) R(20,19) R(20,23) R(21,3) R(21,22) R(22,4) R(22,21) R(23,20) R(23,24) R(24,5) R(24,23) R(25,0) R(25,8) R(26,0) R(26,27) R(27,26) R(27,28) R(28,11) R(28,27) R(29,0) R(29,30) R(30,29) R(30,31) R(31,30) R(31,32) R(32,12) R(32,31) R(n1,0) R(n1,n7) R(n2,0) R(n2,17) R(n3,7) R(n3,17) R(n4,13) R(n4,n5) R(n4,n6) R(n5,16) R(n5,n4) R(n6,n4) R(n6,n7) R(n7,n1) R(n7,n6)

-------------------------------------------------------------------------------------------------------------

-> Ur-ecdysozoa = priapulus_caudatus (solution already obtained in "chaetognaths_taxB")

score = 344:

model 54:

-------------

R(0,1) R(0,9) R(0,10) R(0,18) R(0,19) R(0,25) R(0,26) R(0,29) R(0,n1) R(0,n2) R(1,0) R(2,18) R(3,20) R(3,21) R(4,22) R(5,24) R(6,17) R(7,n3) R(8,25) R(9,0) R(10,0) R(11,28) R(12,32) R(13,14) R(13,15) R(13,n4) R(14,13) R(15,13) R(16,n5) R(17,6) R(17,n3) R(18,0) R(18,2) R(19,0) R(19,20) R(20,3) R(20,19) R(20,23) R(21,3) R(21,22) R(22,4) R(22,21) R(23,20) R(23,24) R(24,5) R(24,23) R(25,0) R(25,8) R(26,0) R(26,27) R(27,26) R(27,28) R(28,11) R(28,27) R(29,0) R(29,30) R(30,29) R(30,31) R(31,30) R(31,32) R(32,12) R(32,31) R(n1,0) R(n1,n7) R(n2,0) R(n2,n3) R(n3,7) R(n3,17) R(n3,n2) R(n4,13) R(n4,n5) R(n4,n6) R(n5,16) R(n5,n4) R(n6,n4) R(n6,n7) R(n7,n1) R(n7,n6)

-------------------------------------------------------------------------------------------------------------

-> Ur-ecdysozoa = priapulus_caudatus (solution already obtained in "chaetognaths_taxB")

score = 290:

model 55:

-------------

R(0,1) R(0,9) R(0,10) R(0,18) R(0,19) R(0,25) R(0,26) R(0,29) R(0,n1) R(0,n2) R(1,0) R(2,18) R(3,20) R(3,21) R(4,22) R(5,24) R(6,17) R(7,n3) R(8,25) R(9,0) R(10,0) R(11,28) R(12,32) R(13,14) R(13,15) R(13,n4) R(14,13) R(15,13) R(16,n5) R(17,6) R(17,n1) R(17,n3) R(18,0) R(18,2) R(19,0) R(19,20) R(20,3) R(20,19) R(20,23) R(21,3) R(21,22) R(22,4) R(22,21) R(23,20) R(23,24) R(24,5) R(24,23) R(25,0) R(25,8) R(26,0) R(26,27) R(27,26) R(27,28) R(28,11) R(28,27) R(29,0) R(29,30) R(30,29) R(30,31) R(31,30) R(31,32) R(32,12) R(32,31) R(n1,0) R(n1,17) R(n2,0) R(n2,n6) R(n3,7) R(n3,17) R(n4,13) R(n4,n6) R(n4,n7) R(n5,16) R(n5,n7) R(n6,n2) R(n6,n4) R(n7,n4) R(n7,n5)

-------------------------------------------------------------------------------------------------------------

-> Ur-ecdysozoa = priapulus_caudatus (solution already obtained in "chaetognaths_taxB")

score = 305:

model 56:

-------------

R(0,1) R(0,9) R(0,10) R(0,18) R(0,19) R(0,25) R(0,26) R(0,29) R(0,n1) R(0,n2) R(1,0) R(2,18) R(3,20) R(3,21) R(4,22) R(5,24) R(6,17) R(7,n3) R(8,25) R(9,0) R(10,0) R(11,28) R(12,32) R(13,14) R(13,15) R(13,n4) R(14,13) R(15,13) R(16,n5) R(17,6) R(17,n3) R(18,0) R(18,2) R(19,0) R(19,20) R(20,3) R(20,19) R(20,23) R(21,3) R(21,22) R(22,4) R(22,21) R(23,20) R(23,24) R(24,5) R(24,23) R(25,0) R(25,8) R(26,0) R(26,27) R(27,26) R(27,28) R(28,11) R(28,27) R(29,0) R(29,30) R(30,29) R(30,31) R(31,30) R(31,32) R(32,12) R(32,31) R(n1,0) R(n1,n3) R(n2,0) R(n2,n6) R(n3,7) R(n3,17) R(n3,n1) R(n4,13) R(n4,n6) R(n4,n7) R(n5,16) R(n5,n7) R(n6,n2) R(n6,n4) R(n7,n4) R(n7,n5)

-------------------------------------------------------------------------------------------------------------

-> Ur-ecdysozoa = limulus_polyphemus (solution already obtained in "chaetognaths_taxA")

score = 277:

model 57:

-------------

R(0,1) R(0,9) R(0,10) R(0,18) R(0,19) R(0,25) R(0,26) R(0,29) R(0,n1) R(0,n2) R(1,0) R(2,18) R(3,20) R(3,21) R(4,22) R(5,24) R(6,17) R(6,n1) R(7,n3) R(8,25) R(9,0) R(10,0) R(11,28) R(12,32) R(13,14) R(13,15) R(13,n4) R(14,13) R(15,13) R(16,n7) R(17,6) R(18,0) R(18,2) R(19,0) R(19,20) R(20,3) R(20,19) R(20,23) R(21,3) R(21,22) R(22,4) R(22,21) R(23,20) R(23,24) R(24,5) R(24,23) R(25,0) R(25,8) R(26,0) R(26,27) R(27,26) R(27,28) R(28,11) R(28,27) R(29,0) R(29,30) R(30,29) R(30,31) R(31,30) R(31,32) R(32,12) R(32,31) R(n1,0) R(n1,6) R(n1,n3) R(n2,0) R(n2,n5) R(n3,7) R(n3,n1) R(n4,13) R(n4,n5) R(n4,n6) R(n5,n2) R(n5,n4) R(n6,n4) R(n6,n7) R(n7,16) R(n7,n6)

-------------------------------------------------------------------------------------------------------------

-> Ur-ecdysozoa = limulus_polyphemus (solution already obtained in "chaetognaths_taxA")

score = 316:

model 58:

-------------

R(0,1) R(0,9) R(0,10) R(0,18) R(0,19) R(0,25) R(0,26) R(0,29) R(0,n1) R(0,n2) R(1,0) R(2,18) R(3,20) R(3,21) R(4,22) R(5,24) R(6,17) R(6,n2) R(7,n3) R(8,25) R(9,0) R(10,0) R(11,28) R(12,32) R(13,14) R(13,15) R(13,n4) R(14,13) R(15,13) R(16,n5) R(17,6) R(18,0) R(18,2) R(19,0) R(19,20) R(20,3) R(20,19) R(20,23) R(21,3) R(21,22) R(22,4) R(22,21) R(23,20) R(23,24) R(24,5) R(24,23) R(25,0) R(25,8) R(26,0) R(26,27) R(27,26) R(27,28) R(28,11) R(28,27) R(29,0) R(29,30) R(30,29) R(30,31) R(31,30) R(31,32) R(32,12) R(32,31) R(n1,0) R(n1,n7) R(n2,0) R(n2,6) R(n2,n3) R(n3,7) R(n3,n2) R(n4,13) R(n4,n5) R(n4,n6) R(n5,16) R(n5,n4) R(n6,n4) R(n6,n7) R(n7,n1) R(n7,n6)

-------------------------------------------------------------------------------------------------------------

-> Ur-ecdysozoa = limulus_polyphemus (solution already obtained in "chaetognaths_taxA")

score = 316:

model 59:

-------------

R(0,1) R(0,9) R(0,10) R(0,18) R(0,19) R(0,25) R(0,26) R(0,29) R(0,n1) R(0,n2) R(1,0) R(2,18) R(3,20) R(3,21) R(4,22) R(5,24) R(6,17) R(6,n1) R(7,n3) R(8,25) R(9,0) R(10,0) R(11,28) R(12,32) R(13,14) R(13,15) R(13,n4) R(14,13) R(15,13) R(15,n7) R(16,n5) R(17,6) R(18,0) R(18,2) R(19,0) R(19,20) R(20,3) R(20,19) R(20,23) R(21,3) R(21,22) R(22,4) R(22,21) R(23,20) R(23,24) R(24,5) R(24,23) R(25,0) R(25,8) R(26,0) R(26,27) R(27,26) R(27,28) R(28,11) R(28,27) R(29,0) R(29,30) R(30,29) R(30,31) R(31,30) R(31,32) R(32,12) R(32,31) R(n1,0) R(n1,6) R(n1,n3) R(n2,0) R(n2,n6) R(n3,7) R(n3,n1) R(n4,13) R(n4,n5) R(n5,16) R(n5,n4) R(n6,n2) R(n6,n7) R(n7,15) R(n7,n6)

-------------------------------------------------------------------------------------------------------------

-> Ur-ecdysozoa = limulus_polyphemus (solution already obtained in "chaetognaths_taxA")

score = 303:

model 60:

-------------

R(0,1) R(0,9) R(0,10) R(0,18) R(0,19) R(0,25) R(0,26) R(0,29) R(0,n1) R(0,n2) R(1,0) R(2,18) R(3,20) R(3,21) R(4,22) R(5,24) R(6,17) R(6,n1) R(7,n3) R(8,25) R(9,0) R(10,0) R(11,28) R(12,32) R(13,14) R(13,15) R(13,n4) R(14,13) R(15,13) R(15,n7) R(16,n6) R(17,6) R(18,0) R(18,2) R(19,0) R(19,20) R(20,3) R(20,19) R(20,23) R(21,3) R(21,22) R(22,4) R(22,21) R(23,20) R(23,24) R(24,5) R(24,23) R(25,0) R(25,8) R(26,0) R(26,27) R(27,26) R(27,28) R(28,11) R(28,27) R(29,0) R(29,30) R(30,29) R(30,31) R(31,30) R(31,32) R(32,12) R(32,31) R(n1,0) R(n1,6) R(n1,n3) R(n2,0) R(n2,n5) R(n3,7) R(n3,n1) R(n4,13) R(n4,n5) R(n5,n2) R(n5,n4) R(n6,16) R(n6,n7) R(n7,15) R(n7,n6)

-------------------------------------------------------------------------------------------------------------

-> Ur-ecdysozoa = priapulus_caudatus (solution already obtained in "chaetognaths_taxB")

score = 359:

model 61:

-------------

R(0,1) R(0,9) R(0,10) R(0,18) R(0,19) R(0,25) R(0,26) R(0,29) R(0,n1) R(0,n2) R(1,0) R(2,18) R(3,20) R(3,21) R(4,22) R(5,24) R(6,17) R(7,n3) R(8,25) R(9,0) R(10,0) R(11,28) R(12,32) R(13,14) R(13,n4) R(14,13) R(15,n4) R(15,n6) R(16,n5) R(17,6) R(17,n3) R(18,0) R(18,2) R(19,0) R(19,20) R(20,3) R(20,19) R(20,23) R(21,3) R(21,22) R(22,4) R(22,21) R(23,20) R(23,24) R(24,5) R(24,23) R(25,0) R(25,8) R(26,0) R(26,27) R(27,26) R(27,28) R(28,11) R(28,27) R(29,0) R(29,30) R(30,29) R(30,31) R(31,30) R(31,32) R(32,12) R(32,31) R(n1,0) R(n1,n3) R(n2,0) R(n2,n7) R(n3,7) R(n3,17) R(n3,n1) R(n4,13) R(n4,15) R(n4,n5) R(n5,16) R(n5,n4) R(n6,15) R(n6,n7) R(n7,n2) R(n7,n6)

-------------------------------------------------------------------------------------------------------------

-> Ur-ecdysozoa = priapulus_caudatus (solution already obtained in "chaetognaths_taxB")

score = 344:

model 62:

-------------

R(0,1) R(0,9) R(0,10) R(0,18) R(0,19) R(0,25) R(0,26) R(0,29) R(0,n1) R(0,n2) R(1,0) R(2,18) R(3,20) R(3,21) R(4,22) R(5,24) R(6,17) R(7,n3) R(8,25) R(9,0) R(10,0) R(11,28) R(12,32) R(13,14) R(13,n4) R(14,13) R(15,n4) R(15,n6) R(16,n5) R(17,6) R(17,n2) R(17,n3) R(18,0) R(18,2) R(19,0) R(19,20) R(20,3) R(20,19) R(20,23) R(21,3) R(21,22) R(22,4) R(22,21) R(23,20) R(23,24) R(24,5) R(24,23) R(25,0) R(25,8) R(26,0) R(26,27) R(27,26) R(27,28) R(28,11) R(28,27) R(29,0) R(29,30) R(30,29) R(30,31) R(31,30) R(31,32) R(32,12) R(32,31) R(n1,0) R(n1,n7) R(n2,0) R(n2,17) R(n3,7) R(n3,17) R(n4,13) R(n4,15) R(n4,n5) R(n5,16) R(n5,n4) R(n6,15) R(n6,n7) R(n7,n1) R(n7,n6)

-------------------------------------------------------------------------------------------------------------

-> Ur-ecdysozoa = priapulus_caudatus (solution already obtained in "chaetognaths_taxB")

score = 292:

model 63:

-------------

R(0,1) R(0,9) R(0,10) R(0,18) R(0,19) R(0,25) R(0,26) R(0,29) R(0,n1) R(0,n2) R(1,0) R(2,18) R(3,20) R(3,21) R(4,22) R(5,24) R(6,17) R(7,n3) R(8,25) R(9,0) R(10,0) R(11,28) R(12,32) R(13,14) R(13,n4) R(14,13) R(15,n4) R(15,n6) R(16,n5) R(17,6) R(17,n2) R(17,n3) R(18,0) R(18,2) R(19,0) R(19,20) R(20,3) R(20,19) R(20,23) R(21,3) R(21,22) R(22,4) R(22,21) R(23,20) R(23,24) R(24,5) R(24,23) R(25,0) R(25,8) R(26,0) R(26,27) R(27,26) R(27,28) R(28,11) R(28,27) R(29,0) R(29,30) R(30,29) R(30,31) R(31,30) R(31,32) R(32,12) R(32,31) R(n1,0) R(n1,n7) R(n2,0) R(n2,17) R(n3,7) R(n3,17) R(n4,13) R(n4,15) R(n4,n7) R(n5,16) R(n5,n6) R(n6,15) R(n6,n5) R(n7,n1) R(n7,n4)

-------------------------------------------------------------------------------------------------------------

-> Ur-ecdysozoa = priapulus_caudatus (solution already obtained in "chaetognaths_taxB")

score = 307:

model 64:

-------------

R(0,1) R(0,9) R(0,10) R(0,18) R(0,19) R(0,25) R(0,26) R(0,29) R(0,n1) R(0,n2) R(1,0) R(2,18) R(3,20) R(3,21) R(4,22) R(5,24) R(6,17) R(7,n3) R(8,25) R(9,0) R(10,0) R(11,28) R(12,32) R(13,14) R(13,n4) R(14,13) R(15,n4) R(15,n6) R(16,n5) R(17,6) R(17,n3) R(18,0) R(18,2) R(19,0) R(19,20) R(20,3) R(20,19) R(20,23) R(21,3) R(21,22) R(22,4) R(22,21) R(23,20) R(23,24) R(24,5) R(24,23) R(25,0) R(25,8) R(26,0) R(26,27) R(27,26) R(27,28) R(28,11) R(28,27) R(29,0) R(29,30) R(30,29) R(30,31) R(31,30) R(31,32) R(32,12) R(32,31) R(n1,0) R(n1,n7) R(n2,0) R(n2,n3) R(n3,7) R(n3,17) R(n3,n2) R(n4,13) R(n4,15) R(n4,n7) R(n5,16) R(n5,n6) R(n6,15) R(n6,n5) R(n7,n1) R(n7,n4)

-------------------------------------------------------------------------------------------------------------

-> Ur-ecdysozoa = priapulus_caudatus (solution already obtained in "chaetognaths_taxB")

score = 292:

model 65:

-------------

R(0,1) R(0,9) R(0,10) R(0,18) R(0,19) R(0,25) R(0,26) R(0,29) R(0,n1) R(0,n2) R(1,0) R(2,18) R(3,20) R(3,21) R(4,22) R(5,24) R(6,17) R(7,n3) R(8,25) R(9,0) R(10,0) R(11,28) R(12,32) R(13,14) R(13,n4) R(14,13) R(15,n4) R(16,n5) R(17,6) R(17,n3) R(18,0) R(18,2) R(19,0) R(19,20) R(20,3) R(20,19) R(20,23) R(21,3) R(21,22) R(22,4) R(22,21) R(23,20) R(23,24) R(24,5) R(24,23) R(25,0) R(25,8) R(26,0) R(26,27) R(27,26) R(27,28) R(28,11) R(28,27) R(29,0) R(29,30) R(30,29) R(30,31) R(31,30) R(31,32) R(32,12) R(32,31) R(n1,0) R(n1,n3) R(n2,0) R(n2,n7) R(n3,7) R(n3,17) R(n3,n1) R(n4,13) R(n4,15) R(n4,n6) R(n4,n7) R(n5,16) R(n5,n6) R(n6,n4) R(n6,n5) R(n7,n2) R(n7,n4)

-------------------------------------------------------------------------------------------------------------

-> Ur-ecdysozoa = priapulus_caudatus (solution already obtained in "chaetognaths_taxB")

score = 277:

model 66:

-------------

R(0,1) R(0,9) R(0,10) R(0,18) R(0,19) R(0,25) R(0,26) R(0,29) R(0,n1) R(0,n2) R(1,0) R(2,18) R(3,20) R(3,21) R(4,22) R(5,24) R(6,17) R(7,n3) R(8,25) R(9,0) R(10,0) R(11,28) R(12,32) R(13,14) R(13,n4) R(14,13) R(15,n4) R(16,n5) R(17,6) R(17,n1) R(17,n3) R(18,0) R(18,2) R(19,0) R(19,20) R(20,3) R(20,19) R(20,23) R(21,3) R(21,22) R(22,4) R(22,21) R(23,20) R(23,24) R(24,5) R(24,23) R(25,0) R(25,8) R(26,0) R(26,27) R(27,26) R(27,28) R(28,11) R(28,27) R(29,0) R(29,30) R(30,29) R(30,31) R(31,30) R(31,32) R(32,12) R(32,31) R(n1,0) R(n1,17) R(n2,0) R(n2,n6) R(n3,7) R(n3,17) R(n4,13) R(n4,15) R(n4,n6) R(n4,n7) R(n5,16) R(n5,n7) R(n6,n2) R(n6,n4) R(n7,n4) R(n7,n5)

-------------------------------------------------------------------------------------------------------------

-> Ur-ecdysozoa = priapulus_caudatus (solution already obtained in "chaetognaths_taxB")

score = 305:

model 67:

-------------

R(0,1) R(0,9) R(0,10) R(0,18) R(0,19) R(0,25) R(0,26) R(0,29) R(0,n1) R(0,n2) R(1,0) R(2,18) R(3,20) R(3,21) R(4,22) R(5,24) R(6,17) R(7,n3) R(8,25) R(9,0) R(10,0) R(11,28) R(12,32) R(13,14) R(13,n4) R(14,13) R(15,n6) R(16,n5) R(17,6) R(17,n2) R(17,n3) R(18,0) R(18,2) R(19,0) R(19,20) R(20,3) R(20,19) R(20,23) R(21,3) R(21,22) R(22,4) R(22,21) R(23,20) R(23,24) R(24,5) R(24,23) R(25,0) R(25,8) R(26,0) R(26,27) R(27,26) R(27,28) R(28,11) R(28,27) R(29,0) R(29,30) R(30,29) R(30,31) R(31,30) R(31,32) R(32,12) R(32,31) R(n1,0) R(n1,n7) R(n2,0) R(n2,17) R(n3,7) R(n3,17) R(n4,13) R(n4,n5) R(n4,n6) R(n5,16) R(n5,n4) R(n6,15) R(n6,n4) R(n6,n7) R(n7,n1) R(n7,n6)

-------------------------------------------------------------------------------------------------------------

-> Ur-ecdysozoa = priapulus_caudatus (solution already obtained in "chaetognaths_taxB")

score = 320:

model 68:

-------------

R(0,1) R(0,9) R(0,10) R(0,18) R(0,19) R(0,25) R(0,26) R(0,29) R(0,n1) R(0,n2) R(1,0) R(2,18) R(3,20) R(3,21) R(4,22) R(5,24) R(6,17) R(7,n3) R(8,25) R(9,0) R(10,0) R(11,28) R(12,32) R(13,14) R(13,n4) R(14,13) R(15,n6) R(16,n5) R(17,6) R(17,n3) R(18,0) R(18,2) R(19,0) R(19,20) R(20,3) R(20,19) R(20,23) R(21,3) R(21,22) R(22,4) R(22,21) R(23,20) R(23,24) R(24,5) R(24,23) R(25,0) R(25,8) R(26,0) R(26,27) R(27,26) R(27,28) R(28,11) R(28,27) R(29,0) R(29,30) R(30,29) R(30,31) R(31,30) R(31,32) R(32,12) R(32,31) R(n1,0) R(n1,n7) R(n2,0) R(n2,n3) R(n3,7) R(n3,17) R(n3,n2) R(n4,13) R(n4,n5) R(n4,n6) R(n5,16) R(n5,n4) R(n6,15) R(n6,n4) R(n6,n7) R(n7,n1) R(n7,n6)

-------------------------------------------------------------------------------------------------------------

-> Ur-ecdysozoa = limulus_polyphemus (solution already obtained in "chaetognaths_taxA")

score = 331:

model 69:

-------------

R(0,1) R(0,9) R(0,10) R(0,18) R(0,19) R(0,25) R(0,26) R(0,29) R(0,n1) R(0,n2) R(1,0) R(2,18) R(3,20) R(3,21) R(4,22) R(5,24) R(6,17) R(6,n1) R(7,n3) R(8,25) R(9,0) R(10,0) R(11,28) R(12,32) R(13,14) R(13,n4) R(14,13) R(15,n4) R(15,n7) R(16,n5) R(17,6) R(18,0) R(18,2) R(19,0) R(19,20) R(20,3) R(20,19) R(20,23) R(21,3) R(21,22) R(22,4) R(22,21) R(23,20) R(23,24) R(24,5) R(24,23) R(25,0) R(25,8) R(26,0) R(26,27) R(27,26) R(27,28) R(28,11) R(28,27) R(29,0) R(29,30) R(30,29) R(30,31) R(31,30) R(31,32) R(32,12) R(32,31) R(n1,0) R(n1,6) R(n1,n3) R(n2,0) R(n2,n6) R(n3,7) R(n3,n1) R(n4,13) R(n4,15) R(n4,n5) R(n5,16) R(n5,n4) R(n6,n2) R(n6,n7) R(n7,15) R(n7,n6)

-------------------------------------------------------------------------------------------------------------

-> Ur-ecdysozoa = limulus_polyphemus (solution already obtained in "chaetognaths_taxA")

score = 279:

model 70:

-------------

R(0,1) R(0,9) R(0,10) R(0,18) R(0,19) R(0,25) R(0,26) R(0,29) R(0,n1) R(0,n2) R(1,0) R(2,18) R(3,20) R(3,21) R(4,22) R(5,24) R(6,17) R(6,n1) R(7,n3) R(8,25) R(9,0) R(10,0) R(11,28) R(12,32) R(13,14) R(13,n4) R(14,13) R(15,n4) R(15,n7) R(16,n5) R(17,6) R(18,0) R(18,2) R(19,0) R(19,20) R(20,3) R(20,19) R(20,23) R(21,3) R(21,22) R(22,4) R(22,21) R(23,20) R(23,24) R(24,5) R(24,23) R(25,0) R(25,8) R(26,0) R(26,27) R(27,26) R(27,28) R(28,11) R(28,27) R(29,0) R(29,30) R(30,29) R(30,31) R(31,30) R(31,32) R(32,12) R(32,31) R(n1,0) R(n1,6) R(n1,n3) R(n2,0) R(n2,n6) R(n3,7) R(n3,n1) R(n4,13) R(n4,15) R(n4,n6) R(n5,16) R(n5,n7) R(n6,n2) R(n6,n4) R(n7,15) R(n7,n5)

-------------------------------------------------------------------------------------------------------------

-> Ur-ecdysozoa = limulus_polyphemus (solution already obtained in "chaetognaths_taxA")

score = 264:

model 71:

-------------

R(0,1) R(0,9) R(0,10) R(0,18) R(0,19) R(0,25) R(0,26) R(0,29) R(0,n1) R(0,n2) R(1,0) R(2,18) R(3,20) R(3,21) R(4,22) R(5,24) R(6,17) R(6,n1) R(7,n3) R(8,25) R(9,0) R(10,0) R(11,28) R(12,32) R(13,14) R(13,n4) R(14,13) R(15,n4) R(16,n5) R(17,6) R(18,0) R(18,2) R(19,0) R(19,20) R(20,3) R(20,19) R(20,23) R(21,3) R(21,22) R(22,4) R(22,21) R(23,20) R(23,24) R(24,5) R(24,23) R(25,0) R(25,8) R(26,0) R(26,27) R(27,26) R(27,28) R(28,11) R(28,27) R(29,0) R(29,30) R(30,29) R(30,31) R(31,30) R(31,32) R(32,12) R(32,31) R(n1,0) R(n1,6) R(n1,n3) R(n2,0) R(n2,n6) R(n3,7) R(n3,n1) R(n4,13) R(n4,15) R(n4,n6) R(n4,n7) R(n5,16) R(n5,n7) R(n6,n2) R(n6,n4) R(n7,n4) R(n7,n5)

-------------------------------------------------------------------------------------------------------------

-> Ur-ecdysozoa = limulus_polyphemus (solution already obtained in "chaetognaths_taxA")

score = 292:

model 72:

-------------

R(0,1) R(0,9) R(0,10) R(0,18) R(0,19) R(0,25) R(0,26) R(0,29) R(0,n1) R(0,n2) R(1,0) R(2,18) R(3,20) R(3,21) R(4,22) R(5,24) R(6,17) R(6,n2) R(7,n3) R(8,25) R(9,0) R(10,0) R(11,28) R(12,32) R(13,14) R(13,n4) R(14,13) R(15,n6) R(16,n5) R(17,6) R(18,0) R(18,2) R(19,0) R(19,20) R(20,3) R(20,19) R(20,23) R(21,3) R(21,22) R(22,4) R(22,21) R(23,20) R(23,24) R(24,5) R(24,23) R(25,0) R(25,8) R(26,0) R(26,27) R(27,26) R(27,28) R(28,11) R(28,27) R(29,0) R(29,30) R(30,29) R(30,31) R(31,30) R(31,32) R(32,12) R(32,31) R(n1,0) R(n1,n7) R(n2,0) R(n2,6) R(n2,n3) R(n3,7) R(n3,n2) R(n4,13) R(n4,n5) R(n4,n6) R(n5,16) R(n5,n4) R(n6,15) R(n6,n4) R(n6,n7) R(n7,n1) R(n7,n6)

-------------------------------------------------------------------------------------------------------------

-> Ur-ecdysozoa = priapulus_caudatus (solution already obtained in "chaetognaths_taxB")

score = 316:

model 73:

-------------

R(0,1) R(0,9) R(0,10) R(0,18) R(0,19) R(0,25) R(0,26) R(0,29) R(0,n1) R(0,n2) R(1,0) R(2,18) R(3,20) R(3,21) R(4,22) R(5,24) R(6,17) R(7,n3) R(8,25) R(9,0) R(10,0) R(11,28) R(12,32) R(13,14) R(13,15) R(14,13) R(15,13) R(15,n4) R(15,n5) R(16,n6) R(17,6) R(17,n2) R(17,n3) R(18,0) R(18,2) R(19,0) R(19,20) R(20,3) R(20,19) R(20,23) R(21,3) R(21,22) R(22,4) R(22,21) R(23,20) R(23,24) R(24,5) R(24,23) R(25,0) R(25,8) R(26,0) R(26,27) R(27,26) R(27,28) R(28,11) R(28,27) R(29,0) R(29,30) R(30,29) R(30,31) R(31,30) R(31,32) R(32,12) R(32,31) R(n1,0) R(n1,n7) R(n2,0) R(n2,17) R(n3,7) R(n3,17) R(n4,15) R(n4,n6) R(n5,15) R(n5,n7) R(n6,16) R(n6,n4) R(n7,n1) R(n7,n5)

-------------------------------------------------------------------------------------------------------------

-> Ur-ecdysozoa = priapulus_caudatus (solution already obtained in "chaetognaths_taxB")

score = 331:

model 74:

-------------

R(0,1) R(0,9) R(0,10) R(0,18) R(0,19) R(0,25) R(0,26) R(0,29) R(0,n1) R(0,n2) R(1,0) R(2,18) R(3,20) R(3,21) R(4,22) R(5,24) R(6,17) R(7,n3) R(8,25) R(9,0) R(10,0) R(11,28) R(12,32) R(13,14) R(13,15) R(14,13) R(15,13) R(15,n4) R(15,n5) R(16,n6) R(17,6) R(17,n3) R(18,0) R(18,2) R(19,0) R(19,20) R(20,3) R(20,19) R(20,23) R(21,3) R(21,22) R(22,4) R(22,21) R(23,20) R(23,24) R(24,5) R(24,23) R(25,0) R(25,8) R(26,0) R(26,27) R(27,26) R(27,28) R(28,11) R(28,27) R(29,0) R(29,30) R(30,29) R(30,31) R(31,30) R(31,32) R(32,12) R(32,31) R(n1,0) R(n1,n7) R(n2,0) R(n2,n3) R(n3,7) R(n3,17) R(n3,n2) R(n4,15) R(n4,n6) R(n5,15) R(n5,n7) R(n6,16) R(n6,n4) R(n7,n1) R(n7,n5)

-------------------------------------------------------------------------------------------------------------

-> Ur-ecdysozoa = limulus_polyphemus (solution already obtained in "chaetognaths_taxA")

score = 303:

model 75:

-------------

R(0,1) R(0,9) R(0,10) R(0,18) R(0,19) R(0,25) R(0,26) R(0,29) R(0,n1) R(0,n2) R(1,0) R(2,18) R(3,20) R(3,21) R(4,22) R(5,24) R(6,17) R(6,n2) R(7,n3) R(8,25) R(9,0) R(10,0) R(11,28) R(12,32) R(13,14) R(13,15) R(14,13) R(15,13) R(15,n4) R(15,n5) R(16,n6) R(17,6) R(18,0) R(18,2) R(19,0) R(19,20) R(20,3) R(20,19) R(20,23) R(21,3) R(21,22) R(22,4) R(22,21) R(23,20) R(23,24) R(24,5) R(24,23) R(25,0) R(25,8) R(26,0) R(26,27) R(27,26) R(27,28) R(28,11) R(28,27) R(29,0) R(29,30) R(30,29) R(30,31) R(31,30) R(31,32) R(32,12) R(32,31) R(n1,0) R(n1,n7) R(n2,0) R(n2,6) R(n2,n3) R(n3,7) R(n3,n2) R(n4,15) R(n4,n6) R(n5,15) R(n5,n7) R(n6,16) R(n6,n4) R(n7,n1) R(n7,n5)

-------------------------------------------------------------------------------------------------------------

-> Ur-ecdysozoa = priapulus_caudatus (solution already obtained in "chaetognaths_taxB")

score = 305:

model 76:

-------------

R(0,1) R(0,9) R(0,10) R(0,18) R(0,19) R(0,25) R(0,26) R(0,29) R(0,n1) R(0,n2) R(1,0) R(2,18) R(3,20) R(3,21) R(4,22) R(5,24) R(6,17) R(7,n3) R(8,25) R(9,0) R(10,0) R(11,28) R(12,32) R(13,14) R(13,15) R(14,13) R(15,13) R(15,n4) R(16,n5) R(17,6) R(17,n1) R(17,n3) R(18,0) R(18,2) R(19,0) R(19,20) R(20,3) R(20,19) R(20,23) R(21,3) R(21,22) R(22,4) R(22,21) R(23,20) R(23,24) R(24,5) R(24,23) R(25,0) R(25,8) R(26,0) R(26,27) R(27,26) R(27,28) R(28,11) R(28,27) R(29,0) R(29,30) R(30,29) R(30,31) R(31,30) R(31,32) R(32,12) R(32,31) R(n1,0) R(n1,17) R(n2,0) R(n2,n6) R(n3,7) R(n3,17) R(n4,15) R(n4,n6) R(n4,n7) R(n5,16) R(n5,n7) R(n6,n2) R(n6,n4) R(n7,n4) R(n7,n5)

-------------------------------------------------------------------------------------------------------------

-> Ur-ecdysozoa = priapulus_caudatus (solution already obtained in "chaetognaths_taxB")

score = 320:

model 77:

-------------

R(0,1) R(0,9) R(0,10) R(0,18) R(0,19) R(0,25) R(0,26) R(0,29) R(0,n1) R(0,n2) R(1,0) R(2,18) R(3,20) R(3,21) R(4,22) R(5,24) R(6,17) R(7,n3) R(8,25) R(9,0) R(10,0) R(11,28) R(12,32) R(13,14) R(13,15) R(14,13) R(15,13) R(15,n4) R(16,n5) R(17,6) R(17,n3) R(18,0) R(18,2) R(19,0) R(19,20) R(20,3) R(20,19) R(20,23) R(21,3) R(21,22) R(22,4) R(22,21) R(23,20) R(23,24) R(24,5) R(24,23) R(25,0) R(25,8) R(26,0) R(26,27) R(27,26) R(27,28) R(28,11) R(28,27) R(29,0) R(29,30) R(30,29) R(30,31) R(31,30) R(31,32) R(32,12) R(32,31) R(n1,0) R(n1,n3) R(n2,0) R(n2,n6) R(n3,7) R(n3,17) R(n3,n1) R(n4,15) R(n4,n6) R(n4,n7) R(n5,16) R(n5,n7) R(n6,n2) R(n6,n4) R(n7,n4) R(n7,n5)

-------------------------------------------------------------------------------------------------------------

-> Ur-ecdysozoa = limulus_polyphemus (solution already obtained in "chaetognaths_taxA")

score = 292:

model 78:

-------------

R(0,1) R(0,9) R(0,10) R(0,18) R(0,19) R(0,25) R(0,26) R(0,29) R(0,n1) R(0,n2) R(1,0) R(2,18) R(3,20) R(3,21) R(4,22) R(5,24) R(6,17) R(6,n1) R(7,n3) R(8,25) R(9,0) R(10,0) R(11,28) R(12,32) R(13,14) R(13,15) R(14,13) R(15,13) R(15,n4) R(16,n7) R(17,6) R(18,0) R(18,2) R(19,0) R(19,20) R(20,3) R(20,19) R(20,23) R(21,3) R(21,22) R(22,4) R(22,21) R(23,20) R(23,24) R(24,5) R(24,23) R(25,0) R(25,8) R(26,0) R(26,27) R(27,26) R(27,28) R(28,11) R(28,27) R(29,0) R(29,30) R(30,29) R(30,31) R(31,30) R(31,32) R(32,12) R(32,31) R(n1,0) R(n1,6) R(n1,n3) R(n2,0) R(n2,n5) R(n3,7) R(n3,n1) R(n4,15) R(n4,n5) R(n4,n6) R(n5,n2) R(n5,n4) R(n6,n4) R(n6,n7) R(n7,16) R(n7,n6)

-------------------------------------------------------------------------------------------------------------

-> Ur-ecdysozoa = limulus_polyphemus (solution already obtained in "chaetognaths_taxA")

score = 322:

model 79:

-------------

R(0,1) R(0,9) R(0,10) R(0,18) R(0,19) R(0,25) R(0,26) R(0,29) R(0,n1) R(1,0) R(2,18) R(3,20) R(3,21) R(4,22) R(5,24) R(6,17) R(6,n2) R(6,n4) R(7,n4) R(8,25) R(9,0) R(10,0) R(11,28) R(12,32) R(13,14) R(13,15) R(13,n5) R(13,n6) R(14,13) R(15,13) R(16,n7) R(17,6) R(18,0) R(18,2) R(19,0) R(19,20) R(20,3) R(20,19) R(20,23) R(21,3) R(21,22) R(22,4) R(22,21) R(23,20) R(23,24) R(24,5) R(24,23) R(25,0) R(25,8) R(26,0) R(26,27) R(27,26) R(27,28) R(28,11) R(28,27) R(29,0) R(29,30) R(30,29) R(30,31) R(31,30) R(31,32) R(32,12) R(32,31) R(n1,0) R(n1,n2) R(n1,n3) R(n2,6) R(n2,n1) R(n3,n1) R(n3,n5) R(n4,6) R(n4,7) R(n5,13) R(n5,n3) R(n6,13) R(n6,n7) R(n7,16) R(n7,n6)

-------------------------------------------------------------------------------------------------------------

-> Ur-ecdysozoa = limulus_polyphemus (solution already obtained in "chaetognaths_taxA")

score = 337:

model 80:

-------------

R(0,1) R(0,9) R(0,10) R(0,18) R(0,19) R(0,25) R(0,26) R(0,29) R(0,n1) R(1,0) R(2,18) R(3,20) R(3,21) R(4,22) R(5,24) R(6,17) R(6,n4) R(7,n4) R(8,25) R(9,0) R(10,0) R(11,28) R(12,32) R(13,14) R(13,15) R(13,n5) R(13,n6) R(14,13) R(15,13) R(16,n7) R(17,6) R(18,0) R(18,2) R(19,0) R(19,20) R(20,3) R(20,19) R(20,23) R(21,3) R(21,22) R(22,4) R(22,21) R(23,20) R(23,24) R(24,5) R(24,23) R(25,0) R(25,8) R(26,0) R(26,27) R(27,26) R(27,28) R(28,11) R(28,27) R(29,0) R(29,30) R(30,29) R(30,31) R(31,30) R(31,32) R(32,12) R(32,31) R(n1,0) R(n1,n2) R(n1,n3) R(n2,n1) R(n2,n6) R(n3,n1) R(n3,n4) R(n4,6) R(n4,7) R(n4,n3) R(n5,13) R(n5,n7) R(n6,13) R(n6,n2) R(n7,16) R(n7,n5)

-------------------------------------------------------------------------------------------------------------

-> Ur-ecdysozoa = limulus_polyphemus (solution already obtained in "chaetognaths_taxA")

score = 326:

model 81:

-------------

R(0,1) R(0,9) R(0,10) R(0,18) R(0,19) R(0,25) R(0,26) R(0,29) R(0,n1) R(1,0) R(2,18) R(3,20) R(3,21) R(4,22) R(5,24) R(6,17) R(6,n4) R(7,n4) R(8,25) R(9,0) R(10,0) R(11,28) R(12,32) R(13,14) R(13,15) R(13,n5) R(14,13) R(15,13) R(16,n6) R(17,6) R(18,0) R(18,2) R(19,0) R(19,20) R(20,3) R(20,19) R(20,23) R(21,3) R(21,22) R(22,4) R(22,21) R(23,20) R(23,24) R(24,5) R(24,23) R(25,0) R(25,8) R(26,0) R(26,27) R(27,26) R(27,28) R(28,11) R(28,27) R(29,0) R(29,30) R(30,29) R(30,31) R(31,30) R(31,32) R(32,12) R(32,31) R(n1,0) R(n1,n2) R(n1,n3) R(n2,n1) R(n2,n4) R(n3,n1) R(n3,n5) R(n4,6) R(n4,7) R(n4,n2) R(n5,13) R(n5,n3) R(n5,n7) R(n6,16) R(n6,n7) R(n7,n5) R(n7,n6)

-------------------------------------------------------------------------------------------------------------

-> Ur-ecdysozoa = limulus_polyphemus (solution already obtained in "chaetognaths_taxA")

score = 352:

model 82:

-------------

R(0,1) R(0,9) R(0,10) R(0,18) R(0,19) R(0,25) R(0,26) R(0,29) R(0,n1) R(1,0) R(2,18) R(3,20) R(3,21) R(4,22) R(5,24) R(6,17) R(6,n4) R(7,n4) R(8,25) R(9,0) R(10,0) R(11,28) R(12,32) R(13,14) R(13,15) R(13,n5) R(14,13) R(15,13) R(15,n7) R(16,n6) R(17,6) R(18,0) R(18,2) R(19,0) R(19,20) R(20,3) R(20,19) R(20,23) R(21,3) R(21,22) R(22,4) R(22,21) R(23,20) R(23,24) R(24,5) R(24,23) R(25,0) R(25,8) R(26,0) R(26,27) R(27,26) R(27,28) R(28,11) R(28,27) R(29,0) R(29,30) R(30,29) R(30,31) R(31,30) R(31,32) R(32,12) R(32,31) R(n1,0) R(n1,n2) R(n1,n3) R(n2,n1) R(n2,n4) R(n3,n1) R(n3,n5) R(n4,6) R(n4,7) R(n4,n2) R(n5,13) R(n5,n3) R(n6,16) R(n6,n7) R(n7,15) R(n7,n6)

-------------------------------------------------------------------------------------------------------------

-> Ur-ecdysozoa = limulus_polyphemus (solution already obtained in "chaetognaths_taxA")

score = 365:

model 83:

-------------

R(0,1) R(0,9) R(0,10) R(0,18) R(0,19) R(0,25) R(0,26) R(0,29) R(0,n1) R(1,0) R(2,18) R(3,20) R(3,21) R(4,22) R(5,24) R(6,17) R(6,n4) R(7,n4) R(8,25) R(9,0) R(10,0) R(11,28) R(12,32) R(13,14) R(13,15) R(13,n5) R(14,13) R(15,13) R(15,n6) R(16,n7) R(17,6) R(18,0) R(18,2) R(19,0) R(19,20) R(20,3) R(20,19) R(20,23) R(21,3) R(21,22) R(22,4) R(22,21) R(23,20) R(23,24) R(24,5) R(24,23) R(25,0) R(25,8) R(26,0) R(26,27) R(27,26) R(27,28) R(28,11) R(28,27) R(29,0) R(29,30) R(30,29) R(30,31) R(31,30) R(31,32) R(32,12) R(32,31) R(n1,0) R(n1,n2) R(n1,n3) R(n2,n1) R(n2,n4) R(n3,n1) R(n3,n6) R(n4,6) R(n4,7) R(n4,n2) R(n5,13) R(n5,n7) R(n6,15) R(n6,n3) R(n7,16) R(n7,n5)

-------------------------------------------------------------------------------------------------------------

-> Ur-ecdysozoa = limulus_polyphemus (solution already obtained in "chaetognaths_taxA")

score = 365:

model 84:

-------------

R(0,1) R(0,9) R(0,10) R(0,18) R(0,19) R(0,25) R(0,26) R(0,29) R(0,n1) R(1,0) R(2,18) R(3,20) R(3,21) R(4,22) R(5,24) R(6,17) R(6,n4) R(7,n4) R(8,25) R(9,0) R(10,0) R(11,28) R(12,32) R(13,14) R(13,15) R(13,n5) R(14,13) R(15,13) R(16,n6) R(17,6) R(18,0) R(18,2) R(19,0) R(19,20) R(20,3) R(20,19) R(20,23) R(21,3) R(21,22) R(22,4) R(22,21) R(23,20) R(23,24) R(24,5) R(24,23) R(25,0) R(25,8) R(26,0) R(26,27) R(27,26) R(27,28) R(28,11) R(28,27) R(29,0) R(29,30) R(30,29) R(30,31) R(31,30) R(31,32) R(32,12) R(32,31) R(n1,0) R(n1,n2) R(n1,n3) R(n2,n1) R(n2,n4) R(n3,n1) R(n3,n7) R(n4,6) R(n4,7) R(n4,n2) R(n5,13) R(n5,n6) R(n5,n7) R(n6,16) R(n6,n5) R(n7,n3) R(n7,n5)

-------------------------------------------------------------------------------------------------------------

-> Ur-ecdysozoa = limulus_polyphemus (solution already obtained in "chaetognaths_taxA")

score = 311:

model 85:

-------------

R(0,1) R(0,9) R(0,10) R(0,18) R(0,19) R(0,25) R(0,26) R(0,29) R(0,n1) R(1,0) R(2,18) R(3,20) R(3,21) R(4,22) R(5,24) R(6,17) R(6,n2) R(6,n4) R(7,n4) R(8,25) R(9,0) R(10,0) R(11,28) R(12,32) R(13,14) R(13,15) R(13,n5) R(14,13) R(15,13) R(16,n6) R(17,6) R(18,0) R(18,2) R(19,0) R(19,20) R(20,3) R(20,19) R(20,23) R(21,3) R(21,22) R(22,4) R(22,21) R(23,20) R(23,24) R(24,5) R(24,23) R(25,0) R(25,8) R(26,0) R(26,27) R(27,26) R(27,28) R(28,11) R(28,27) R(29,0) R(29,30) R(30,29) R(30,31) R(31,30) R(31,32) R(32,12) R(32,31) R(n1,0) R(n1,n2) R(n1,n3) R(n2,6) R(n2,n1) R(n3,n1) R(n3,n5) R(n4,6) R(n4,7) R(n5,13) R(n5,n3) R(n5,n7) R(n6,16) R(n6,n7) R(n7,n5) R(n7,n6)

-------------------------------------------------------------------------------------------------------------

-> Ur-ecdysozoa = limulus_polyphemus (solution already obtained in "chaetognaths_taxA")

score = 337:

model 86:

-------------

R(0,1) R(0,9) R(0,10) R(0,18) R(0,19) R(0,25) R(0,26) R(0,29) R(0,n1) R(1,0) R(2,18) R(3,20) R(3,21) R(4,22) R(5,24) R(6,17) R(6,n2) R(6,n4) R(7,n4) R(8,25) R(9,0) R(10,0) R(11,28) R(12,32) R(13,14) R(13,15) R(13,n5) R(14,13) R(15,13) R(15,n7) R(16,n6) R(17,6) R(18,0) R(18,2) R(19,0) R(19,20) R(20,3) R(20,19) R(20,23) R(21,3) R(21,22) R(22,4) R(22,21) R(23,20) R(23,24) R(24,5) R(24,23) R(25,0) R(25,8) R(26,0) R(26,27) R(27,26) R(27,28) R(28,11) R(28,27) R(29,0) R(29,30) R(30,29) R(30,31) R(31,30) R(31,32) R(32,12) R(32,31) R(n1,0) R(n1,n2) R(n1,n3) R(n2,6) R(n2,n1) R(n3,n1) R(n3,n5) R(n4,6) R(n4,7) R(n5,13) R(n5,n3) R(n6,16) R(n6,n7) R(n7,15) R(n7,n6)

-------------------------------------------------------------------------------------------------------------

-> Ur-ecdysozoa = limulus_polyphemus (solution already obtained in "chaetognaths_taxA")

score = 350:

model 87:

-------------

R(0,1) R(0,9) R(0,10) R(0,18) R(0,19) R(0,25) R(0,26) R(0,29) R(0,n1) R(1,0) R(2,18) R(3,20) R(3,21) R(4,22) R(5,24) R(6,17) R(6,n2) R(6,n4) R(7,n4) R(8,25) R(9,0) R(10,0) R(11,28) R(12,32) R(13,14) R(13,15) R(13,n5) R(14,13) R(15,13) R(15,n6) R(16,n7) R(17,6) R(18,0) R(18,2) R(19,0) R(19,20) R(20,3) R(20,19) R(20,23) R(21,3) R(21,22) R(22,4) R(22,21) R(23,20) R(23,24) R(24,5) R(24,23) R(25,0) R(25,8) R(26,0) R(26,27) R(27,26) R(27,28) R(28,11) R(28,27) R(29,0) R(29,30) R(30,29) R(30,31) R(31,30) R(31,32) R(32,12) R(32,31) R(n1,0) R(n1,n2) R(n1,n3) R(n2,6) R(n2,n1) R(n3,n1) R(n3,n6) R(n4,6) R(n4,7) R(n5,13) R(n5,n7) R(n6,15) R(n6,n3) R(n7,16) R(n7,n5)

-------------------------------------------------------------------------------------------------------------

-> Ur-ecdysozoa = limulus_polyphemus (solution already obtained in "chaetognaths_taxA")

score = 350:

model 88:

-------------

R(0,1) R(0,9) R(0,10) R(0,18) R(0,19) R(0,25) R(0,26) R(0,29) R(0,n1) R(1,0) R(2,18) R(3,20) R(3,21) R(4,22) R(5,24) R(6,17) R(6,n2) R(6,n4) R(7,n4) R(8,25) R(9,0) R(10,0) R(11,28) R(12,32) R(13,14) R(13,15) R(13,n5) R(14,13) R(15,13) R(16,n6) R(17,6) R(18,0) R(18,2) R(19,0) R(19,20) R(20,3) R(20,19) R(20,23) R(21,3) R(21,22) R(22,4) R(22,21) R(23,20) R(23,24) R(24,5) R(24,23) R(25,0) R(25,8) R(26,0) R(26,27) R(27,26) R(27,28) R(28,11) R(28,27) R(29,0) R(29,30) R(30,29) R(30,31) R(31,30) R(31,32) R(32,12) R(32,31) R(n1,0) R(n1,n2) R(n1,n3) R(n2,6) R(n2,n1) R(n3,n1) R(n3,n7) R(n4,6) R(n4,7) R(n5,13) R(n5,n6) R(n5,n7) R(n6,16) R(n6,n5) R(n7,n3) R(n7,n5)

-------------------------------------------------------------------------------------------------------------

-> Ur-ecdysozoa = limulus_polyphemus (solution already obtained in "chaetognaths_taxA")

score = 337:

model 89:

-------------

R(0,1) R(0,9) R(0,10) R(0,18) R(0,19) R(0,25) R(0,26) R(0,29) R(0,n1) R(1,0) R(2,18) R(3,20) R(3,21) R(4,22) R(5,24) R(6,17) R(6,n2) R(6,n4) R(7,n4) R(8,25) R(9,0) R(10,0) R(11,28) R(12,32) R(13,14) R(13,15) R(14,13) R(15,13) R(15,n5) R(15,n6) R(16,n7) R(17,6) R(18,0) R(18,2) R(19,0) R(19,20) R(20,3) R(20,19) R(20,23) R(21,3) R(21,22) R(22,4) R(22,21) R(23,20) R(23,24) R(24,5) R(24,23) R(25,0) R(25,8) R(26,0) R(26,27) R(27,26) R(27,28) R(28,11) R(28,27) R(29,0) R(29,30) R(30,29) R(30,31) R(31,30) R(31,32) R(32,12) R(32,31) R(n1,0) R(n1,n2) R(n1,n3) R(n2,6) R(n2,n1) R(n3,n1) R(n3,n5) R(n4,6) R(n4,7) R(n5,15) R(n5,n3) R(n6,15) R(n6,n7) R(n7,16) R(n7,n6)

-------------------------------------------------------------------------------------------------------------

-> Ur-ecdysozoa = limulus_polyphemus (solution already obtained in "chaetognaths_taxA")

score = 352:

model 90:

-------------

R(0,1) R(0,9) R(0,10) R(0,18) R(0,19) R(0,25) R(0,26) R(0,29) R(0,n1) R(1,0) R(2,18) R(3,20) R(3,21) R(4,22) R(5,24) R(6,17) R(6,n4) R(7,n4) R(8,25) R(9,0) R(10,0) R(11,28) R(12,32) R(13,14) R(13,15) R(14,13) R(15,13) R(15,n5) R(15,n6) R(16,n7) R(17,6) R(18,0) R(18,2) R(19,0) R(19,20) R(20,3) R(20,19) R(20,23) R(21,3) R(21,22) R(22,4) R(22,21) R(23,20) R(23,24) R(24,5) R(24,23) R(25,0) R(25,8) R(26,0) R(26,27) R(27,26) R(27,28) R(28,11) R(28,27) R(29,0) R(29,30) R(30,29) R(30,31) R(31,30) R(31,32) R(32,12) R(32,31) R(n1,0) R(n1,n2) R(n1,n3) R(n2,n1) R(n2,n6) R(n3,n1) R(n3,n4) R(n4,6) R(n4,7) R(n4,n3) R(n5,15) R(n5,n7) R(n6,15) R(n6,n2) R(n7,16) R(n7,n5)

-------------------------------------------------------------------------------------------------------------

-> Ur-ecdysozoa = limulus_polyphemus (solution already obtained in "chaetognaths_taxA")

score = 341:

model 91:

-------------

R(0,1) R(0,9) R(0,10) R(0,18) R(0,19) R(0,25) R(0,26) R(0,29) R(0,n1) R(1,0) R(2,18) R(3,20) R(3,21) R(4,22) R(5,24) R(6,17) R(6,n4) R(7,n4) R(8,25) R(9,0) R(10,0) R(11,28) R(12,32) R(13,14) R(13,15) R(14,13) R(15,13) R(15,n5) R(16,n6) R(17,6) R(18,0) R(18,2) R(19,0) R(19,20) R(20,3) R(20,19) R(20,23) R(21,3) R(21,22) R(22,4) R(22,21) R(23,20) R(23,24) R(24,5) R(24,23) R(25,0) R(25,8) R(26,0) R(26,27) R(27,26) R(27,28) R(28,11) R(28,27) R(29,0) R(29,30) R(30,29) R(30,31) R(31,30) R(31,32) R(32,12) R(32,31) R(n1,0) R(n1,n2) R(n1,n3) R(n2,n1) R(n2,n4) R(n3,n1) R(n3,n5) R(n4,6) R(n4,7) R(n4,n2) R(n5,15) R(n5,n3) R(n5,n7) R(n6,16) R(n6,n7) R(n7,n5) R(n7,n6)

-------------------------------------------------------------------------------------------------------------

-> Ur-ecdysozoa = limulus_polyphemus (solution already obtained in "chaetognaths_taxA")

score = 326:

model 92:

-------------

R(0,1) R(0,9) R(0,10) R(0,18) R(0,19) R(0,25) R(0,26) R(0,29) R(0,n1) R(1,0) R(2,18) R(3,20) R(3,21) R(4,22) R(5,24) R(6,17) R(6,n2) R(6,n4) R(7,n4) R(8,25) R(9,0) R(10,0) R(11,28) R(12,32) R(13,14) R(13,15) R(14,13) R(15,13) R(15,n5) R(16,n6) R(17,6) R(18,0) R(18,2) R(19,0) R(19,20) R(20,3) R(20,19) R(20,23) R(21,3) R(21,22) R(22,4) R(22,21) R(23,20) R(23,24) R(24,5) R(24,23) R(25,0) R(25,8) R(26,0) R(26,27) R(27,26) R(27,28) R(28,11) R(28,27) R(29,0) R(29,30) R(30,29) R(30,31) R(31,30) R(31,32) R(32,12) R(32,31) R(n1,0) R(n1,n2) R(n1,n3) R(n2,6) R(n2,n1) R(n3,n1) R(n3,n5) R(n4,6) R(n4,7) R(n5,15) R(n5,n3) R(n5,n7) R(n6,16) R(n6,n7) R(n7,n5) R(n7,n6)

-------------------------------------------------------------------------------------------------------------

-> Ur-ecdysozoa = limulus_polyphemus (solution already obtained in "chaetognaths_taxA")

score = 311:

model 93:

-------------

R(0,1) R(0,9) R(0,10) R(0,18) R(0,19) R(0,25) R(0,26) R(0,29) R(0,n1) R(1,0) R(2,18) R(3,20) R(3,21) R(4,22) R(5,24) R(6,17) R(6,n2) R(6,n4) R(7,n4) R(8,25) R(9,0) R(10,0) R(11,28) R(12,32) R(13,14) R(13,n5) R(13,n6) R(14,13) R(15,n5) R(16,n7) R(17,6) R(18,0) R(18,2) R(19,0) R(19,20) R(20,3) R(20,19) R(20,23) R(21,3) R(21,22) R(22,4) R(22,21) R(23,20) R(23,24) R(24,5) R(24,23) R(25,0) R(25,8) R(26,0) R(26,27) R(27,26) R(27,28) R(28,11) R(28,27) R(29,0) R(29,30) R(30,29) R(30,31) R(31,30) R(31,32) R(32,12) R(32,31) R(n1,0) R(n1,n2) R(n1,n3) R(n2,6) R(n2,n1) R(n3,n1) R(n3,n5) R(n4,6) R(n4,7) R(n5,13) R(n5,15) R(n5,n3) R(n6,13) R(n6,n7) R(n7,16) R(n7,n6)

-------------------------------------------------------------------------------------------------------------

-> Ur-ecdysozoa = limulus_polyphemus (solution already obtained in "chaetognaths_taxA")

score = 337:

model 94:

-------------

R(0,1) R(0,9) R(0,10) R(0,18) R(0,19) R(0,25) R(0,26) R(0,29) R(0,n1) R(1,0) R(2,18) R(3,20) R(3,21) R(4,22) R(5,24) R(6,17) R(6,n2) R(6,n4) R(7,n4) R(8,25) R(9,0) R(10,0) R(11,28) R(12,32) R(13,14) R(13,n5) R(13,n6) R(14,13) R(15,n5) R(16,n7) R(17,6) R(18,0) R(18,2) R(19,0) R(19,20) R(20,3) R(20,19) R(20,23) R(21,3) R(21,22) R(22,4) R(22,21) R(23,20) R(23,24) R(24,5) R(24,23) R(25,0) R(25,8) R(26,0) R(26,27) R(27,26) R(27,28) R(28,11) R(28,27) R(29,0) R(29,30) R(30,29) R(30,31) R(31,30) R(31,32) R(32,12) R(32,31) R(n1,0) R(n1,n2) R(n1,n3) R(n2,6) R(n2,n1) R(n3,n1) R(n3,n6) R(n4,6) R(n4,7) R(n5,13) R(n5,15) R(n5,n7) R(n6,13) R(n6,n3) R(n7,16) R(n7,n5)

-------------------------------------------------------------------------------------------------------------

-> Ur-ecdysozoa = limulus_polyphemus (solution already obtained in "chaetognaths_taxA")

score = 352:

model 95:

-------------

R(0,1) R(0,9) R(0,10) R(0,18) R(0,19) R(0,25) R(0,26) R(0,29) R(0,n1) R(1,0) R(2,18) R(3,20) R(3,21) R(4,22) R(5,24) R(6,17) R(6,n4) R(7,n4) R(8,25) R(9,0) R(10,0) R(11,28) R(12,32) R(13,14) R(13,n5) R(13,n6) R(14,13) R(15,n5) R(16,n7) R(17,6) R(18,0) R(18,2) R(19,0) R(19,20) R(20,3) R(20,19) R(20,23) R(21,3) R(21,22) R(22,4) R(22,21) R(23,20) R(23,24) R(24,5) R(24,23) R(25,0) R(25,8) R(26,0) R(26,27) R(27,26) R(27,28) R(28,11) R(28,27) R(29,0) R(29,30) R(30,29) R(30,31) R(31,30) R(31,32) R(32,12) R(32,31) R(n1,0) R(n1,n2) R(n1,n3) R(n2,n1) R(n2,n6) R(n3,n1) R(n3,n4) R(n4,6) R(n4,7) R(n4,n3) R(n5,13) R(n5,15) R(n5,n7) R(n6,13) R(n6,n2) R(n7,16) R(n7,n5)

-------------------------------------------------------------------------------------------------------------

-> Ur-ecdysozoa = limulus_polyphemus (solution already obtained in "chaetognaths_taxA")

score = 326:

model 96:

-------------

R(0,1) R(0,9) R(0,10) R(0,18) R(0,19) R(0,25) R(0,26) R(0,29) R(0,n1) R(1,0) R(2,18) R(3,20) R(3,21) R(4,22) R(5,24) R(6,17) R(6,n4) R(7,n4) R(8,25) R(9,0) R(10,0) R(11,28) R(12,32) R(13,14) R(13,n5) R(13,n6) R(14,13) R(15,n6) R(16,n7) R(17,6) R(18,0) R(18,2) R(19,0) R(19,20) R(20,3) R(20,19) R(20,23) R(21,3) R(21,22) R(22,4) R(22,21) R(23,20) R(23,24) R(24,5) R(24,23) R(25,0) R(25,8) R(26,0) R(26,27) R(27,26) R(27,28) R(28,11) R(28,27) R(29,0) R(29,30) R(30,29) R(30,31) R(31,30) R(31,32) R(32,12) R(32,31) R(n1,0) R(n1,n2) R(n1,n3) R(n2,n1) R(n2,n6) R(n3,n1) R(n3,n4) R(n4,6) R(n4,7) R(n4,n3) R(n5,13) R(n5,n7) R(n6,13) R(n6,15) R(n6,n2) R(n7,16) R(n7,n5)

-------------------------------------------------------------------------------------------------------------

-> Ur-ecdysozoa = limulus_polyphemus (solution already obtained in "chaetognaths_taxA")

score = 313:

model 97:

-------------

R(0,1) R(0,9) R(0,10) R(0,18) R(0,19) R(0,25) R(0,26) R(0,29) R(0,n1) R(1,0) R(2,18) R(3,20) R(3,21) R(4,22) R(5,24) R(6,17) R(6,n4) R(7,n4) R(8,25) R(9,0) R(10,0) R(11,28) R(12,32) R(13,14) R(13,n5) R(14,13) R(15,n5) R(16,n6) R(17,6) R(18,0) R(18,2) R(19,0) R(19,20) R(20,3) R(20,19) R(20,23) R(21,3) R(21,22) R(22,4) R(22,21) R(23,20) R(23,24) R(24,5) R(24,23) R(25,0) R(25,8) R(26,0) R(26,27) R(27,26) R(27,28) R(28,11) R(28,27) R(29,0) R(29,30) R(30,29) R(30,31) R(31,30) R(31,32) R(32,12) R(32,31) R(n1,0) R(n1,n2) R(n1,n3) R(n2,n1) R(n2,n4) R(n3,n1) R(n3,n5) R(n4,6) R(n4,7) R(n4,n2) R(n5,13) R(n5,15) R(n5,n3) R(n5,n7) R(n6,16) R(n6,n7) R(n7,n5) R(n7,n6)

-------------------------------------------------------------------------------------------------------------

-> Ur-ecdysozoa = limulus_polyphemus (solution already obtained in "chaetognaths_taxA")

score = 328:

model 98:

-------------

R(0,1) R(0,9) R(0,10) R(0,18) R(0,19) R(0,25) R(0,26) R(0,29) R(0,n1) R(1,0) R(2,18) R(3,20) R(3,21) R(4,22) R(5,24) R(6,17) R(6,n4) R(7,n4) R(8,25) R(9,0) R(10,0) R(11,28) R(12,32) R(13,14) R(13,n5) R(14,13) R(15,n5) R(15,n7) R(16,n6) R(17,6) R(18,0) R(18,2) R(19,0) R(19,20) R(20,3) R(20,19) R(20,23) R(21,3) R(21,22) R(22,4) R(22,21) R(23,20) R(23,24) R(24,5) R(24,23) R(25,0) R(25,8) R(26,0) R(26,27) R(27,26) R(27,28) R(28,11) R(28,27) R(29,0) R(29,30) R(30,29) R(30,31) R(31,30) R(31,32) R(32,12) R(32,31) R(n1,0) R(n1,n2) R(n1,n3) R(n2,n1) R(n2,n4) R(n3,n1) R(n3,n5) R(n4,6) R(n4,7) R(n4,n2) R(n5,13) R(n5,15) R(n5,n3) R(n6,16) R(n6,n7) R(n7,15) R(n7,n6)

-------------------------------------------------------------------------------------------------------------

-> Ur-ecdysozoa = limulus_polyphemus (solution already obtained in "chaetognaths_taxA")

score = 380:

model 99:

-------------

R(0,1) R(0,9) R(0,10) R(0,18) R(0,19) R(0,25) R(0,26) R(0,29) R(0,n1) R(1,0) R(2,18) R(3,20) R(3,21) R(4,22) R(5,24) R(6,17) R(6,n4) R(7,n4) R(8,25) R(9,0) R(10,0) R(11,28) R(12,32) R(13,14) R(13,n5) R(14,13) R(15,n5) R(15,n6) R(16,n7) R(17,6) R(18,0) R(18,2) R(19,0) R(19,20) R(20,3) R(20,19) R(20,23) R(21,3) R(21,22) R(22,4) R(22,21) R(23,20) R(23,24) R(24,5) R(24,23) R(25,0) R(25,8) R(26,0) R(26,27) R(27,26) R(27,28) R(28,11) R(28,27) R(29,0) R(29,30) R(30,29) R(30,31) R(31,30) R(31,32) R(32,12) R(32,31) R(n1,0) R(n1,n2) R(n1,n3) R(n2,n1) R(n2,n4) R(n3,n1) R(n3,n6) R(n4,6) R(n4,7) R(n4,n2) R(n5,13) R(n5,15) R(n5,n7) R(n6,15) R(n6,n3) R(n7,16) R(n7,n5)

-------------------------------------------------------------------------------------------------------------

-> Ur-ecdysozoa = limulus_polyphemus (solution already obtained in "chaetognaths_taxA")

score = 298:

model 100:

-------------
[truncated: 172,719 more chars]
